# Transcriptome Analysis Reveals the Molecular Mechanisms of BR Negative Regulatory Factor StBIN2 Maintaining Tuber Dormancy

**DOI:** 10.3390/ijms25042244

**Published:** 2024-02-13

**Authors:** Shifeng Liu, Chengcheng Cai, Liqin Li, Liping Yu, Qiang Wang, Xiyao Wang

**Affiliations:** 1 College of Agronomy, Sichuan Agriculture University, Chengdu 611130, China; 2020101001@stu.sicau.edu.cn (S.L.); caichengcheng@stu.sicau.edu.cn (C.C.); liliqin88@163.com (L.L.); 15902871886@163.com (L.Y.); qwang@sicau.edu.cn (Q.W.); 2 Potato Research and Development Center, Sichuan Agricultural University, Chengdu 611130, China

**Keywords:** *Solanum tuberosum* L., *StBIN2*, tuber dormancy, RNA-seq, ABA signal, lignin synthesis, BR

## Abstract

Potato is an important food crop. After harvest, these tubers will undergo a period of dormancy. Brassinosteroids (BRs) are a new class of plant hormones that regulate plant growth and seed germination. In this study, 500 nM of BR was able to break the dormancy of tubers. Additionally, exogenous BR also upregulated BR signal transduction genes, except for *StBIN2*. *StBIN2* is a negative regulator of BR, but its specific role in tuber dormancy remains unclear. Transgenic methods were used to regulate the expression level of *StBIN2* in tubers. It was demonstrated that the overexpression of *StBIN2* significantly prolonged tuber dormancy while silencing *StBIN2* led to premature sprouting. To further investigate the effect of *StBIN2* on tuber dormancy, RNA-Seq was used to analyze the differentially expressed genes in *OE*-*StBIN2*, *RNAi*-*StBIN2*, and WT tubers. The results showed that *StBIN2* upregulated the expression of ABA signal transduction genes but inhibited the expression of lignin synthesis key genes. Meanwhile, it was also found that StBIN2 physically interacted with StSnRK2.2 and StCCJ9. These results indicate that *StBIN2* maintains tuber dormancy by mediating ABA signal transduction and lignin synthesis. The findings of this study will help us better understand the molecular mechanisms underlying potato tuber dormancy and provide theoretical support for the development of new varieties using related genes.

## 1. Introduction

Potato (*Solanum tuberosum* L.) is an annual herbaceous plant of the Solanaceae family and the Solanaceae genus, with some advantages, such as high yield, strong adaptability, and tolerance to barren conditions [1]. It is the fourth largest staple food after rice, corn, and wheat [2]. China’s potato planting areas and total yield rank first in the world [3]. Potatoes play an important role in maintaining national stability and ensuring food security. After harvesting, potatoes need to undergo a period of dormancy before they can sprout. Different potato varieties have different dormancy periods [4]. Due to different actual production needs, commercial potatoes need to maintain dormancy without sprouting. Meanwhile, seed potatoes require their dormancy period to match the sowing date to maintain high seed potato vitality. The study of potato dormancy mechanisms can provide a theoretical basis for tuber dormancy regulation and storage preservation and has important practical significance for the development of the potato industry.

Tuber dormancy is a complex physiological mechanism involving multiple factors, and hormones play a pivotal role in determining the dormancy state of potato tubers [5]. There is a close relationship between Gibberellin (GA), Abscisic acid (ABA), and BR and tuber dormancy and sprouting [6]. Recent research has found that multiple hormones, rather than a single hormone, regulate potato dormancy and sprouting. The content and proportion of endogenous hormones significantly influence the state of potato dormancy and sprouting, and thus, maintaining a balance between several hormones is vital for promoting a particular growth state [7]. GA is a commonly used plant hormone for breaking the dormancy of seeds and tubers. GA treatment of potato tubers significantly hastens the rate of breaking dormancy compared to the control. In addition to breaking dormancy, GA promotes the growth of sprouts [8]. Overexpressing the GA biosynthesis gene *GA20ox* in potatoes results in a reduced dormancy period, revealing that GA negatively regulates tuber dormancy [9]. ABA and GA display antagonistic effects during tuber dormancy. ABA is the earliest and most extensively studied type in the study of potato tuber dormancy mechanisms. The ABA content is highest during tuber dormancy and rapidly decreases with the release of dormancy [10].

BR, classified as a major category of plant hormones, holds a pivotal role in influencing the growth and development of plants [11]. It actively participates in critical processes such as cell elongation, cell division, vascular differentiation, pollen development, seed germination, plant aging, and the response of plants to stress [12]. BR exerts an opposing influence on seed germination when compared to ABA. The application of external BR effectively represses the expression of *BRI1*, *BSK1*, *BSU1*, and BR-responsive genes within the leaf BR signaling pathway, ultimately enhancing yield [13]. Studies in quantitative phosphoproteomics suggest that BR facilitates the sprouting of potato tubers by modulating the phosphorylation of proteins [14]. BIN2 (Brassinolide insensitive 2) serves as the negative regulator in BR signal transduction. It inhibits BR signaling by phosphorylating BES1 and BZR1 [15]. BIN2 acts as a mediator between ABA and BR signal transduction. ABI1, ABI2, and ABI5 are members of the PP2C family and are negative regulators of ABA signaling. Research indicates that ABA can enhance the expression of BIN2 through ABI1, ABI2, and ABI5, influencing BR signal transduction and maintaining seed dormancy [16]. In Arabidopsis, the overexpression of *BIN2* strengthens the inhibitory impact of ABA on seed germination and reinforces ABA signaling through the ABI5 signaling factor [17]. SnRK2s is a key protein in the ABA signaling pathway. StBIN2 protein augments ABA signal transduction by phosphorylating SnRK2.3 in potatoes [18]. Furthermore, the overexpression of *StBIN2* was advantageous in promoting the formation of potato tubers and enhancing crop yield [19]. As a crucial kinase, BIN2 plays a significant role in the seed dormancy process. However, considering the distinct mechanism of potato dormancy, further investigation is required to ascertain whether *StBIN2* contributes to maintaining dormancy in potatoes.

Lignin, a crucial secondary metabolite produced in the phenylalanine metabolic pathway of plant cells, is polymerized from phenylpropane monomers [20]. Upon synthesis within the cell, lignin monomers undergo polymerization catalyzed by peroxidase (POX) and lactose (LAC) to form biologically active lignin through a series of chemical reactions. Hydrophobic lignin is found in the seed coats of many species, and the lignin content is positively correlated with the degree of seed coat cracking [21]. This is beneficial for the transpiration and respiration of seeds. Studies have revealed that treating Anacamptis morio (*Orchidaceae*) seeds with laccase results in lignin degradation within the seed coat, leading to a two-fold increase in the seed germination rate compared to the control [22]. Polygonatum cyrtonema, an important medicinal plant, experiences difficulty in seed germination. Transcriptome and metabolome analyses have indicated that compared to non-germinated seeds, germinated seeds promote flavonoid synthesis, inhibit lignin synthesis, and facilitate the germination of *Polygonatum cyrtonema* seeds [23]. In Arabidopsis, a mutation of the lignin synthesis gene *AtLAC15* leads to a reduction in seed coat lignin, promoting seed germination [24]. Furthermore, mutations in the Arabidopsis peroxidases, PRX2 and PRX25, result in a decreased seed germination rate [25]. Fatty alcohol hydroxycinnamoyl transferase (FTH) is an essential enzyme involved in the synthesis of cork resin. The downregulation of *FTH* expression in potatoes led to skin cracking and substantial water loss [26]. *StSN2* played a crucial role in maintaining tuber dormancy, and metabolic studies indicated that it negatively regulated lignin synthesis to promote tuber dormancy [27]. Vulavala revealed that lignin and suberin were involved in potato periderm formation and maturation [28]. In short, potato skin can effectively reduce the respiration and transpiration of potatoes to maintain dormancy. Lignin is an important component of potato skin and also plays a positive role in maintaining tuber dormancy.

RNA-seq is a crucial tool in the field of transcriptomics research, helping to uncover the molecular mechanisms behind specific biological processes by analyzing variations in gene expression [29]. Over the past few years, the maturation of RNA-seq technology has led to its increasingly widespread application. In a study on drought resistance in sweet potatoes, RNA-seq identified the crucial roles played by the ABA, ethylene (ETH), and jasmonic acid (JA) signaling pathways. These pathways contribute significantly to the resilience of sweet potatoes in the face of drought conditions [30]. Similarly, in investigations of low-temperature effects on cassava, RNA-seq has revealed the involvement of certain differentially expressed genes (DEGs) in the metabolic processes of reactive oxygen species (ROS). This potentially explains the plant’s ability to withstand cold temperatures [31]. Furthermore, the dynamic examination of grain development stages through RNA-seq has revealed the molecular mechanisms governing cereal development. This provides a strong foundation for future investigations into the functional aspects of genes involved in millet grain development [32].

Potato tuber dormancy is a complex biological process. In recent years, there has been increasing research on the use of exogenous hormones to break tuber dormancy. However, little is known about the molecular mechanisms behind it. In this study, we found that exogenous BR promoted tuber sprout but inhibited the expression of *StBIN2*. *StBIN2* is a negative regulatory factor of BR and plays an important role in plant growth and development. To explore the role of *StBIN2* in regulating tuber dormancy, we created overexpression and silencing materials for *StBIN2*. We analyzed the dormancy period and germination rate of *StBIN2* transgenic tubers, and the results showed that *StBIN2* was a key gene for maintaining tuber dormancy. The RNA-seq and protein interaction experiments elucidated that *StBIN2* maintains tuber dormancy by promoting ABA signal transduction and inhibiting lignin synthesis. The results of this study have improved our understanding of the molecular mechanism of potato tuber dormancy and provided theoretical support for potato molecular breeding.

## 2. Results

### 2.1. BR Promotes Tuber Sprout by Inhibiting StBIN2

**Figure 1:**
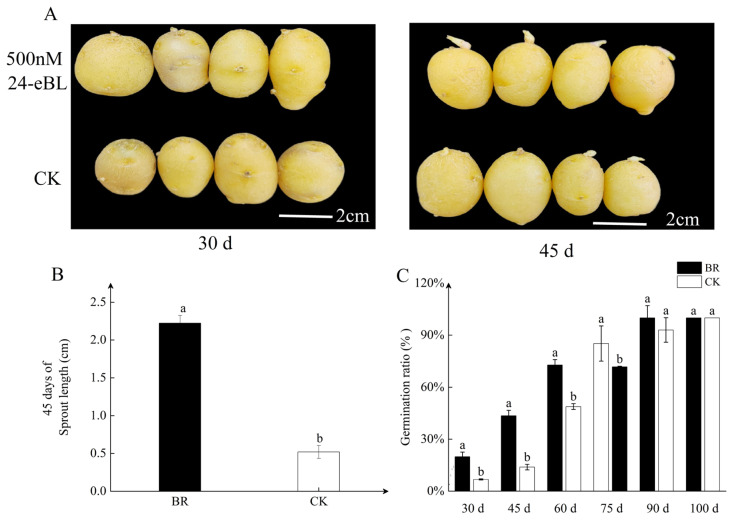
BR promotes potato tuber sprouts. (**A**) Germination of tubers at different stages after BR treatment. (**B**) The length of potato sprouts after 45 days of BR treatment. (**C**) Potato tuber germination rate. CK represents distilled water treatment, and d represents the number of days processed. Tubers were stored at 20 °C in the dark prior to being analyzed. Data are the means ± SD of three biological replicates. Different small letters represent significant differences (*p* < 0.05).

BR plays an important role in promoting seed germination, and some studies have shown that BR can break the dormancy of tubers and promote sprouting [14]. In order to further investigate the role of BR in promoting tuber sprouting, this experiment treated the tubers with 500 nM of 24-eBL (analogs of artificially synthesized BR) and stored them at 20 ± 2 °C. The results showed that 500 nM of 24-eBL significantly promoted the germination of tubers, and the bud length under 24-eBL treatment was significantly higher than the control at 30 d and 45 d (Figure 1A). The spout length at 45 days was 4.32 times that of the control (Figure 1B). Subsequently, we conducted a statistical analysis of the germination rate during storage (a bud length ≥2 mm is defined as a sprout). The results showed that 24-eBL significantly improved the sprouting rate of tubers, with the treatment group fully germinating about 10 days earlier than the control (Figure 1C). From this, it can be seen that BR can break the dormancy of tubers and promote sprouting.

**Figure 2:**
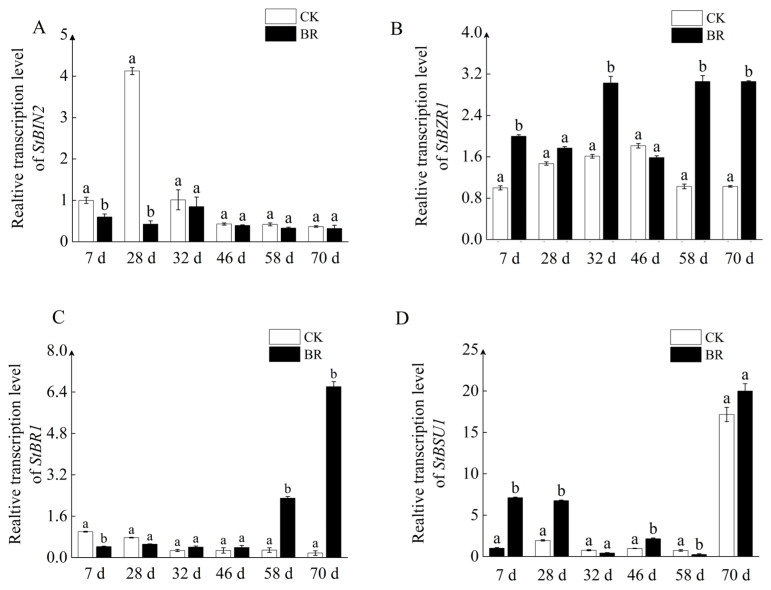
qPCR detection of the key genes involved in the BR signaling pathway during tuber storage. (**A**) Expression of the *StBIN2* gene. (**B**) Expression of the *StBZR1* gene. (**C**) Expression of the *StBR1* gene. (**D**) Expression of the *StBSU1* gene. CK represents distilled water treatment, and d represents the number of days processed. Tubers were stored at 20 °C in the dark for 70 days prior to being analyzed. Data are the means ± SD of three biological replicates. Different small letters represent significant differences (*p* < 0.05).

It has been verified that BR can enhance the sprouting of tubers. The transcriptomic and proteomic analysis of early dormancy and sprouting has revealed that BR signaling genes play a crucial role in tuber sprouting [33]. In order to confirm the involvement of BR signaling genes in tuber sprout, the expression of key genes in the BR signaling pathway was examined. qPCR results demonstrated that BR treatment inhibited the expression of *StBIN2* compared to WT and delayed its peak time (Figure 2A). On the other hand, *BSU1*, *BR1*, and *BZR1* act as positive regulatory factors in the BR signaling pathway. qPCR results showed that BR promoted the expression of *StBSU1*, *StBR1*, and *StBZR1* during storage, particularly in the late dormancy stage, being significantly higher than the control, suggesting a positive role in tuber sprout (Figure 2B–D). It can be inferred that *StBIN2* may play a major role in tuber dormancy.

### 2.2. StBIN2 Maintains Tuber Dormancy

**Figure 3:**
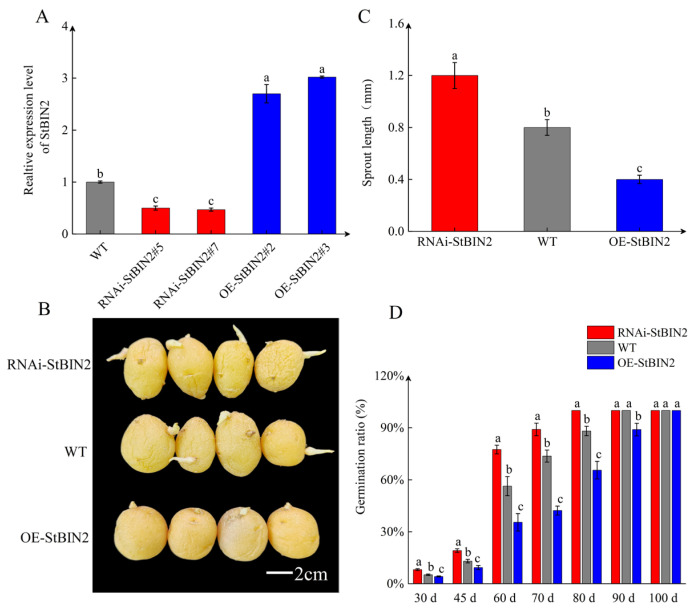
*StBIN2* maintains tuber dormancy. (**A**) qPCR detection of *StBIN2* gene expression in WT, *OE-StBIN2*, and *RNAi-StBIN2*. (**B**) Sprout length of WT, *OE-StBIN2*, and *RNAi-StBIN2*. (**C**) The potato tuber phenotype is stored for 60 days. (**D**) Germination rate of potato tubers. d represents the number of days processed. Tubers were stored at 20 °C in the dark prior to being analyzed. Data are the means ± SD of three biological replicates. Different small letters represent significant differences (*p* < 0.05).

To investigate the function of *StBIN2* in tuber dormancy, we created overexpression and silencing materials of *StBIN2* through potato genetic transformation. The qPCR results showed that compared to WT, the transcription levels of *StBIN2* in *OE-StBIN2#2* and *OE-StBIN2#3* were 2.8 and 3.04-fold higher, respectively, while the two silent lines were 0.40 and 0.38-fold higher, respectively (Figure 3A). We stored the harvested tubers for statistical purposes. After 60 days of storage, we observed and measured the length of the sprout. We found that overexpression of *StBIN2* significantly prolonged the dormancy period of the tubers (Figure 3B), leading to slow bud growth. Specifically, the bud length of WT was two-fold higher than that of *OE-StBIN2* and 0.6-fold higher than that of *RNAi-StBIN2*, respectively (Figure 3C). Throughout the storage period, the germination rate of *RNAi-StBIN2* was higher than that of WT and *OE-StBIN2*. Complete sprouting occurred one week earlier than in WT and 2 weeks earlier than in *OE-StBIN2* (Figure 3D). Therefore, *StBIN2* has the function of maintaining tuber dormancy.

### 2.3. Analysis and Identification of Differentially Expressed Genes

**Figure 4:**
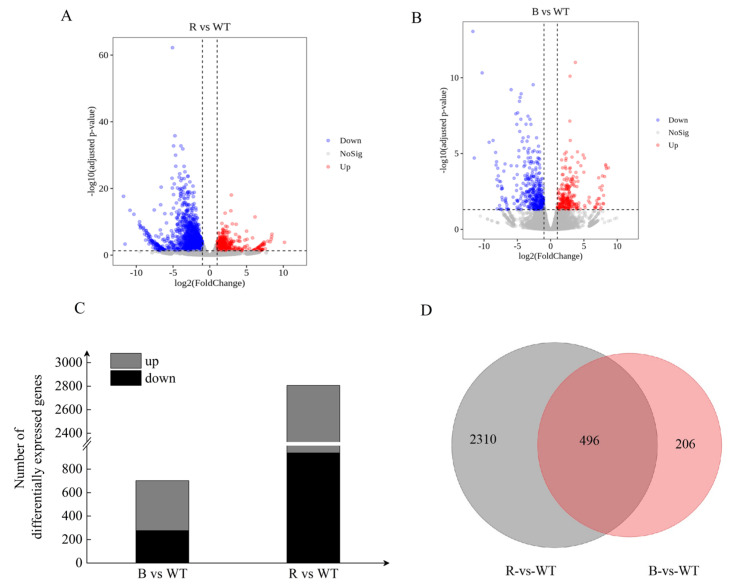
Identification of differentially expressed genes (DEGs). (**A**,**B**) Volcano plot showing the DEGs revealed via RNA-seq analysis. Red, green, and black dots represent upregulated, downregulated, and insignificant DEGs, respectively. (**C**) Statistics on the number of differentially expressed genes. (**D**) The genes that are identified in both the B vs WT and R vs WT comparisons, as indicated by their intersection, are those that are common to both comparisons. B represents *OE-StBIN2*, R represents *RNAi-StBIN2*, and WT represents wild-type.

In order to investigate the molecular mechanism of *StBIN2* in tuber dormancy, we conducted RNA-seq analysis on potato tuber budding eyes stored for 45 days (the critical period from dormancy to germination). The Illumina NovaSeq6000 sequencing platform was used for PE150 mode sequencing. RNA-seq analysis was conducted on three biological replicas of WT (referred to as W), *OE-StBIN2* (referred to as B), and *RNAi-StBIN2* (referred to as R) potatoes. The Illumina NovaSeq6000 sequencing platform was utilized for PE150 pattern sequencing. This study completed the transcriptome analysis of nine samples, with 382218628 clean data retained after removing adapters and low-quality sequences (Supplementary Table S1). The Q30 base percentage of each sample was 95% or above, and the GC content ranged from 43.84% to 45.045% (Supplementary Table S2). The RNA-seq reads for each library ranged from 84.93% to 88.79% and were successfully mapped to the potato genome (Supplementary Table S3). The Clean Reads of each sample were aligned against the S. tuberosum DM reference genome (V6.1). Using DESeq2 for differential analysis. We established two comparative groups, B vs. W and R vs. WT, to investigate the gene expression differences caused by different levels of *StBIN2*. Differential gene screening was performed using criteria of Fold Change ≥ 2 and FDR < 0.01; the resulting differentially expressed gene list underwent functional enrichment analysis. We identified a total of 3508 differentially expressed genes as follows: 2806 (938 upregulated, 1868 downregulated) and 702 (277 upregulated, 425 downregulated) in B vs. WT and R vs. WT, respectively (Supplementary Table S4) (Figure 4C); the 496 genes were identified in both B vs WT and R vs WT (Figure 4D).

### 2.4. Gene Ontology (GO) Analysis

**Figure 5:**
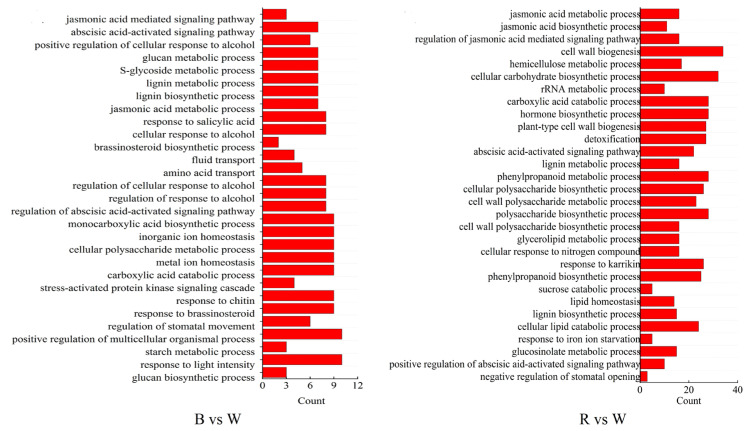
GO enrichment analysis of B vs W and R vs W genes. The vertical axis represents different biological processes, and the horizontal axis represents the number of genes enriched. B represents *OE-StBIN2*, R represents *RNAi-StBIN2*, and W represents wild-type.

In the analysis of the biological processes of GO, we found that the number of GO terms in B vs W was 1200 (Supplementary Table S5), while that in R vs W was 2578 (Supplementary Table S6). This data strongly suggests that the physiological metabolism inside the tuber became more intense after the expression of *StBIN2* was suppressed, providing more possibility for the supply of substances and energy required for sprouting. ABA is a hormone that plays an important role in tuber dormancy [34]. In B vs W and R vs W, we screened out the ABA activation signal pathway (GO: 0009787) (Figure 5). In this pathway, genes such as *StSnRK2.2* (Soltu.DM.08G023690), *StABI5* (Soltu.DM.11G016910), *StBZIP* (Soltu.DM.09G003280), and *StABI3* (Soltu.DM.04G033590) showed significant differences in their TPM values (Supplementary Figure S1). This suggests that *StBIN2* may maintain tuber dormancy by regulating the ABA signal pathway. Furthermore, peroxidase is a key substance that promotes lignin synthesis [35]. Studies have shown that the content of lignin is directly related to tuber dormancy [27]. In the GO analysis, we found that both B vs W and R vs W were associated with the lignin synthesis pathway (GO: 0009809) (Figure 5). Among them, B vs W had seven related genes enriched, while R vs W had 15. Among these genes, *StCCJ9* (Soltu.DM.08G023690) and *StPOD* (Soltu.DM.01G044300) showed significant differences in their TPM values (Supplementary Figure S1). This suggests that *StBIN2* may inhibit lignin synthesis by affecting peroxidase, thereby affecting tuber dormancy and germination. Additionally, GO analysis also involved the JA signal metabolism pathway (GO:0009694) (Figure 5) and the starch decomposition metabolism process (GO:0005983) (Figure 5). Studies have shown that jasmonic acid can help break dormancy in pear buds [36], while starch decomposition can provide energy for tuber sprouting, accelerating the processes [37]. These findings further demonstrate the complexity of tuber dormancy, which involves multiple biological processes. *StBIN2* may maintain tuber dormancy by regulating multiple pathways, providing us with further directions for exploration.

### 2.5. RT-qPCR of the Selected DEGs

**Figure 6:**
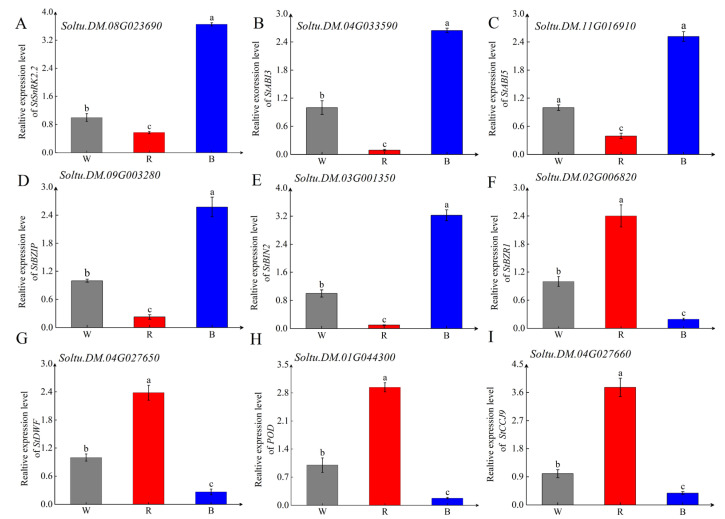
qPCR detection of genes in different tubers stored for 45 days. (**A**) The expression level of *StSnRK2.2*. (**B**) The expression level of *StABI3*. (**C**) The expression level of *StABI5*. (**D**) The expression level of *StBZIP.* (**E**) The expression level of *StBIN2*. (**F**) The expression level of *StBZR1*. (**G**) The expression level of *StDWF*. (**H**) The expression level of *StPOD.* (**I**) The expression level of *StCCJ9.* Tubers were stored at 20 °C in the dark prior to being analyzed. B represents *OE-StBIN2*, R represents *RNAi-StBIN2*, and W represents wild-type. Data are the means ± SD of three biological replicates. Different small letters represent significant differences (*p* < 0.05).

To ensure the reliability of our RNA-seq data, we selected nine differentially expressed genes for validation using qPCR. We extracted RNA from the tuber buds of OE-StBIN2, RNAi-StBIN2, and WT that had been stored for 45 days. We reversed transcribed the RNA into cDNA for qPCR experiments. The qPCR results confirmed that StBIN2 indeed promoted the expression of StSnRK2.2, StAB13, StABI5, and StBZIP. These are key genes in the ABA signaling pathway (Figure 6A–D). Furthermore, in the silencing material, due to the reduced inhibitory effect of StBIN2, the expression levels of BR signal transduction and synthesis genes StBZR1 and StDWF were found to increase (Figure 6F,G). Additionally, we observed that the overexpression of StBIN2 suppressed the expression of StPOD and StCCJ9 (a homolog of peroxidase AtPER12) (Figure 6H,I). When we compared the qPCR results of the selected genes with TPM values (Supplementary Table S7), we observed a consistent expression trend. Therefore, we can confidently infer that our transcriptome data is reliable, and the differentially expressed genes screened can be used for further analysis in subsequent experiments.

### 2.6. Physical Interactions of StBIN2 Protein

Protein interaction plays a pivotal role in cellular signal transduction [38]. Previous studies have shown that BIN2 can regulate various life activities by interacting with multiple proteins in plants [39]. In Arabidopsis, the interaction between BIN2 and SnRK2.2 positively regulates the ABA signaling pathway [40]. CONSTANS (CO) is a key regulator of floral initiation in response to the photoperiod, and GhBIN2 delays flowering by phosphorylating the Thr280 residue of CO in Arabidopsis [41]. ICE1 (Inducer of CBF expression 1) is the first identified transcription factor that enhances plant cold resistance by positively regulating CBF3 [42]. The interaction between HbBIN2 and HbICE1 negatively regulates rubber tolerance to cold stress by inhibiting the transcriptional activity of HbICE1 [43]. BIN2 phosphorylates JAZ1, an inhibitor of the jasmonic acid pathway, leading to the degradation of JAZ protein and negatively regulating plant defense against *Verticillium dahliae* in Arabidopsis and cotton [44]. 

**Figure 7:**
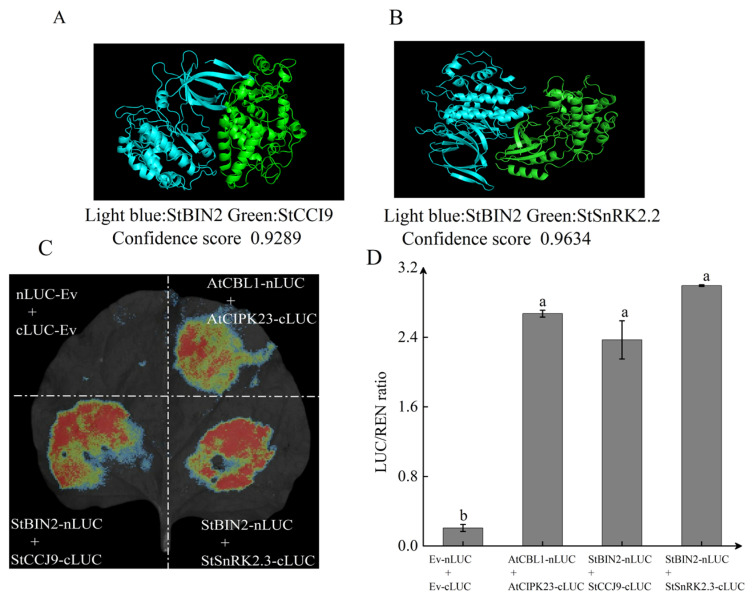
Verification of interaction between StBIN2 and StCCJ9, StSnRK2.2. (**A**) Y2H-AOS analysis of the interaction between StBIN2 and StCCJ9. Light blue represents StBIN2 protein, green represents StCCJ9 protein. (**B**) Y2H-AOS analysis of the interaction between StBIN2 and StSnrk2.2. Light blue represents StBIN2 protein, green represents StSnrk2.2 protein. (**C**) Verification of dual luciferase interaction. The area shaded in red indicates interaction. (**D**) LUC enzyme activity detection. Data are the means ± SD of three biological replicates. Different small letters represent significant differences (*p* < 0.05).

We employed the yeast two-hybrid antibody optimization system (Y2H-AOS) [45] and luciferase complementary assays to further investigate the interaction between StSnRK2.2 and StCCJ9. The Y2H-AOS results revealed a distinct protein binding pattern between StBIN2 + StCCJ9 and StBIN2 + StSnRK2.2, with confidence scores exceeding 0.9 (confidence scores > 0.8 indicating a high likelihood of interaction) (Figure 7A,B). The results from the dual luciferase complementary experiment were also promising. Fluorescence (red area in tobacco) was observed in tobacco infiltrated with StBIN2 + StCCJ9, StBIN2 + StSnRK2.2, and a positive control, while no fluorescence was observed in the negative control (Figure 7C). The LUC enzyme activity detection revealed that the enzyme activity of StBIN2 + StCCJ9 and StBIN2 + StSnRK2.2 was more than five times higher than that of the negative control (Figure 7D). Through bioinformatics analysis and dual luciferase complementary experiments, we have strong evidence supporting the existence of a protein interaction between StBIN2 and both StSnRK2.2 and StCCJ9.

## 3. Discussion

BR, a vital growth regulator in plants, has been shown to play a positive role in seed germination of species such as Arabidopsis, rice, and oilseed rape [46,47]. Earlier reports indicated that exogenous BR could promote potato tuber sprouting by regulating protein phosphorylation [14]. In this study, we observed a four-fold increase in sprout length in tubers treated with 500 nM of BR compared to the control after 45 days (Figure 1B). Additionally, the dormancy period of these tubers was shortened by approximately 10 days (Figure 1C). Subsequently, we examined the expression of BR signaling genes during storage and found that BR treatment promoted the expression of signaling genes except for *StBIN2*. This indicates that BR can promote tuber sprouting not only through protein phosphorylation but also by enhancing the expression of responsive genes (Figure 2A). Surprisingly, another study reported a decrease in the expression of BR synthesis and signaling genes in potato leaves treated with 300 nM of BR, except for *StBIN2* [13]. This discrepancy could be attributed to the varying sensitivities of different tissues to BR. Nevertheless, under 500 nM of BR treatment, the tuber dormancy was indeed shortened, confirming that BR has the ability to break tuber dormancy.

As a negative regulator of BR, BIN2 can bind to proteins in the ABA signaling pathway to positively regulate the ABA signaling pathway [40]. Abscisic acid insensitive 5 (ABI5) is an important transcription factor in the ABA signaling pathway. In Arabidopsis, BIN2 phosphorylates ABI5, thereby positively regulating the ABA response to inhibit seed germination [17]. SnRK2s are key proteins in ABA signal transduction [48]. In Arabidopsis, BIN2 interacts with SnRK2.2/2.3/2.6 and phosphorylates them, thereby positively regulating the ABA signaling pathway [40]. In this study, we confirmed that StBIN2 interacted with StSnrk2.2 in tobacco, and RNA-seq results indicated that *StBIN2* promoted the expression of *StSnRK2.2*. Additionally, the expression levels of *ABI3* and *ABI5* were also elevated in the overexpressed tubers, indicating that StBIN2 interacted with StSnRK2.2 to positively regulate ABA signal transduction and maintain tuber dormancy. Fortunately, previous studies have found that StBIN2 interacts with ABI1, PP2C2s, ABI5, and SnRK2.2 in yeast [19]. This further confirms that *StBIN2* mediates the ABA signaling pathway to maintain tuber dormancy.

In previous studies, it was found that the roughness and cracking of the pericarp are correlated with lignin content. When the pericarp cracks, water loss becomes severe, leading to a shorter fruit lifespan [49]. The skin of potato tubers serves as the primary barrier for water retention. Previous research found that the overexpression of *StSN2* resulted in smoother skin with slower water loss, leading to a prolonged dormancy period. Silencing *StSN2* had the opposite effect. Further exploration revealed that silencing *StSN2* elevated lignin content, leading to the collapse of the periderm and skin cracking [27]. My previous research has shown that StSN2 interacts with StBIN2 to enhance StBIN2 expression [18]. It can be inferred that *StBIN2* may function similarly to *StSN2* in regulating lignin synthesis. In this study, RNA-seq and qPCR results both indicated that *StBIN2* inhibited the expression of key peroxidase genes *StPOD* and *StCCJ9*. There is an interaction between StBIN2 and StCCJ9 in tobacco. Peroxidases catalyze the synthesis of lignin monomers. In Arabidopsis, the prx2 and prx25 mutant seeds have reduced lignin levels but higher germination rates than WT [25]. In citrus, overexpression of *CsPrx25* enhances cell wall lignification [46]. RNAi-mediated silencing of *StSN2* in potatoes results in elevated peroxidase levels, including Prx1, Prx2, Prx4, Prx5, and Prx6 [27]. These findings suggest that *StBIN2* can regulate lignin synthesis through peroxidases, ultimately suppressing tuber sprouting.

In summary, this study has revealed that 500 nM of BR can break the dormancy of potato tubers. *StBIN2* has been identified as the key gene that maintains tuber dormancy. *StBIN2* primarily maintains tuber dormancy through two mechanisms. First, it interacts with StSnRK2.2 to activate the ABA signaling pathway and maintain tuber dormancy. Additionally, StBIN2 interacts with StCCJ9 to downregulate peroxidase-related gene expression and negatively regulate lignin synthesis, ultimately inhibiting tuber sprouting. In addition, the other differentially expressed genes identified by RNA-seq provide theoretical support for the functional analysis of *StBIN2*, which can further explore other functions of *StBIN2*.

## 4. Material and Method

### 4.1. Plant Materials and Growth Conditions

‘Chuanyu 10’ (WT) is a common tetraploid potato variety widely used in the southwest region of China. It is a medium and early maturing variety cultivated by the Crop Research Institute of Sichuan Academy of Agricultural Sciences. The virus-free tissue culture seedlings of ‘Chuanyu 10’ were provided by the Potato Research and Development Center of Sichuan Agricultural University.

We used plant tissue culture technology to expand sterile potato seedlings. We inserted the seedlings into MS medium for growth and placed them in a tissue culture room at 20 °C with a 16 h/8 h light and dark cycle. After 25 days of tissue culture, we planted the tissue culture seedlings in a substrate containing coconut shells. We regularly watered them. After 90–100 days, we harvested the tubers. Following the harvest, the tubers underwent a cleaning and drying process. Subsequently, tubers of consistent size were chosen and stored in a shaded area at a temperature of 20 °C for the next experiment.

We planted Nicotiana benthamiana seeds in a substrate and cultured them for one month under a temperature of 20 °C and a 16 h light/8 h dark cycle. Then, we used them for dual luciferase experiments.

### 4.2. Generation of StBIN2 Transgenic Potato Lines

After sequence alignment analysis, we selected the 40 bp to 324 bp segments within the open reading frame (ORF) region of *StBIN2* (Soltu.DM.03G001350.2) as the most suitable target sequence for RNA interference (RNAi). Subsequently, we inserted it into the *pBWA(V)KS* vector, creating an interference vector for the *StBIN2* gene. To achieve an overexpression of *StBIN2*, we inserted the full-length *StBIN2* (1149 bp) into the *pBI121* vector with the 35S promoter, resulting in the construction of the overexpression vector. The constructed vectors were introduced into *Agrobacterium tumefaciens* GV3101 and cultured in a YEB liquid medium. Potato stem segments (0.5–1 cm) from the ‘Chuanyu 10’ variety were immersed in the bacterial suspension for 3–5 min. Subsequently, the treated segments were transferred to a differentiating culture set to induce shoot development [50]. Finally, we obtained sterile plants with different levels of expression of *SBIN2*.

### 4.3. Hormone Treatment

To conduct hormone treatment, the 60 tubers were immersed in 1.5 L of distilled water (with the addition of 100 μL of DMSO) and 500 nM of 24-epibrassinolide (a highly active BR dissolved in DMSO, Sigma Aldrich, Shanghai, China) for a duration of 30 min. Following this, the tubers were removed and allowed to dry [14]. Subsequently, the dried tubers were placed in a square paper box measuring 15 cm × 15 cm × 15 cm. This box was positioned in an environment maintained at 20 °C. Periodically, at intervals of one week, the tubers were examined for signs of sprouting.

### 4.4. RNA-Seq Analysis

Total RNA from tuber budding eyes was extracted using the MolPure® Plant RNA Kit (YEASEN, Shanghai, China). The quality and quantity of the prepared samples were estimated using a NanoPhotometer® spectrophotometer (IMPLEN, Westlake Village, CA, USA). Next, the samples were mailed to APExBIO Technology LLC (Shanghai, China) for pair-end RNA sequencing on a PE150 Sequencing System. The raw data obtained from sequencing was subjected to adapter removal and low-quality filtering to obtain filtered data. HISAT2 was used to map the trimmed reads to the S. tuberosum DM reference genome (V6.1). The Stringtie software was employed for transcript reconstruction, assembling accurate transcripts, and quantifying the expression levels of each gene or transcript. Differential expression analysis was performed using DESeq2 [51]. DEGs were identified via pairwise sample comparisons in adjacent sampling points (B vs WT and R vs WT). Genes with an adjusted |log2 (fold-change)| > 1 and *p*-value < 0.05 were defined as differentially expressed genes [52]. These DEGs were subjected to GO enrichment analyses using the GOseq R package, with *p*-values of < 0.05 as the cut-off for the significantly enriched GO [53]. The KEGG database (http://www.genome.jp/kegg/; accessed on 16 November 2023) was used to test the statistically enriched KEGG pathways, with *p*-values < 0.05 as the threshold value of the significantly enriched pathways [54].

### 4.5. Validation of RNA-Seq Using RT-qPCR

To further validate the transcriptome data, nine genes were selected for qRT-PCR. We extracted RNA from the tuber buds and used 1 μg of RNA for reverse transcription into cDNA using Hifair II 1st Strand cDNA Synthesis SuperMix (YEASEN, Shanghai, China). qPCR was carried out using the Hieff® qPCR SYBR Green Master Mix (YEASEN, Shanghai, China) and Bio-Rad CFX96 (Bio-Rad CFX96, Hercules, CA, USA). The primers are listed in Supplemental Table S8. The gene expression was normalized to the internal reference gene EF-1αL. In this experiment, we conducted three biological replicates.

### 4.6. Dual-Luciferase Assay

To obtain the coding sequence (CDS) of *StBIN2*, *StCCJ9* (Soltu.DM.04G027660), and *StSnRK2.2* (Soltu.DM.08G023690), we performed PCR amplification. The *StBIN2* gene was cloned into the nLUC vector, and the *StSnRK2.2* and *StCCJ9* genes were inserted into the cLUC vector. Subsequently, the constructed plasmids were separately transformed into *Agrobacterium tumefaciens* GV3101. The transformed strains were cultured overnight in the YEB medium. Next, the StBIN2 bacterial solution was mixed with the StSnRK2.2 and StCCJ9 bacterial solutions, and the mixtures were infiltrated into tobacco leaves. After 36 h, the leaves were subjected to a photo recording using a molecular imaging system (Bio-Rad, Hercules, CA, USA). LUC enzyme activity detection was performed using a dual luciferase reporter assay kit (Vazyme, Nanjing, China) [18] for this experiment. The experiment was repeated three times biologically. The primers are listed in Supplemental Table S8.

### 4.7. Protein Interaction Bioinformatics Analysis

Y2H-AOS is a suite that integrates homology search, template-based modeling, structural prediction, molecular docking, biological information integration, and work management for powerful and fast protein docking [45]. By inputting the amino acid sequences of proteins, the HDOCK server automatically predicts their interactions through a hybrid algorithm based on template and template-free docking [54].

### 4.8. Statistical Analysis

All experiments in this study were independently repeated three times, and the data are presented as the mean ± SD (*n* = 3). The statistical analysis was initially performed using SPSS 14.0 software (IBM, New York, NY, USA), while the figures were originally created using Origin 2021 software (Origin Lab, Northampton, MA, USA). The significance of differences was denoted using different lowercase letters.

## Data Availability

The data presented in this study are available within the article and Supplementary Materials.

## Supplementary Materials

The following supporting information can be downloaded at: https://www.mdpi.com/article/10.3390/ijms25042244/s1.
