# Supplementary material for: Transcriptome Analysis Reveals the Molecular Mechanisms of BR Negative Regulatory Factor StBIN2 Maintaining Tuber Dormancy
Source: Int J Mol Sci. 2024 Feb 13;25(4):2244. doi: 10.3390/ijms25042244 (PMC10889842; doi:10.3390/ijms25042244)
Supplement: Supplementary file 1 [file ijms-25-02244-s001.zip › ijms-2787968-supplementary.pdf]

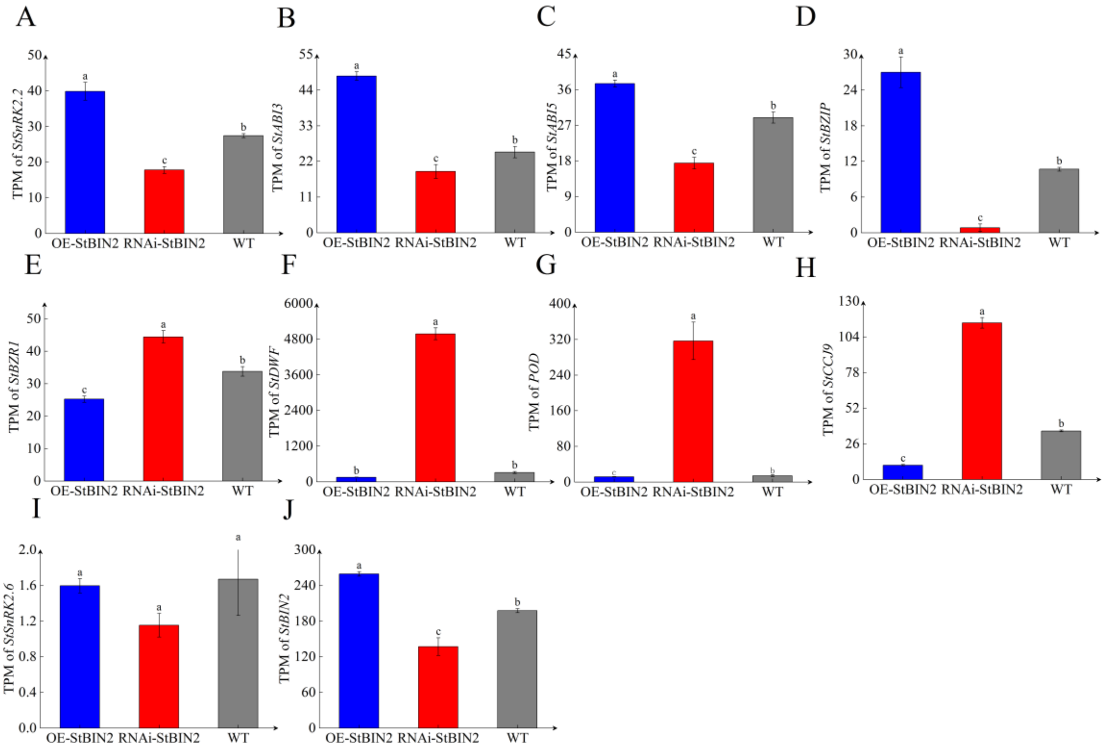

Supplementary Figure1. TPM value of differentially expressed genes.

Sample	Total_Reads
B-1	44727750
B-2	41715860
B-3	41133672
R-1	27336452
R-2	39958154
R-3	60885768
WT-1	43593164
WT-2	44235482
WT-3	38632326
Total	382218628

Sample	Q20	Q30	GC
B-1	97.49%	95.51%	44.14%
B-2	97.36%	95.18%	45.05%
B-3	97.33%	95.14%	44.10%
R-1	97.76%	95.70%	43.52%
R-2	97.30%	95.10%	43.53%
R-3	97.30%	95.12%	43.43%
WT-1	97.36%	95.23%	43.84%
WT-2	97.42%	95.36%	44.61%
WT-3	97.45%	95.37%	44.43%

Sample	Total_Reads	Total_Mapped	Match Ratio
B-1	44727750	39402409	88.09%
B-2	41715860	36707344	87.99%
B-3	41133672	35637226	86.64%
R-1	27336452	23690803	86.66%
R-2	39958154	34181791	85.54%
R-3	60885768	51709328	84.93%
WT-1	43593164	37206553	85.35%
WT-2	44235482	39275915	88.79%
WT-3	38632326	33632699	87.06%

Comparision	Total	# Significantly up-regulated genes	# Significantly down-regulated genes	显著差异基因信息表
B-vs-WT	702	277	425	DiffResult_Annot_B-vs-WT_0.05_1_info.xls
R-vs-WT	2806	938	1868	DiffResult_Annot_R-vs-WT_0.05_1_info.xls
Total	3508			

Number	ID	Description	GeneRatio	BgRatio	pvalue	p.adjust	qvalue	geneID	Count
1	GO:0009787	regulation of abscisic acid-activated signaling pathway	8/295	131/12756	0.011103671	0.209041265	0.196938876	Soltu.DM.08G023690/Soltu.DM.04G005970/Soltu.DM.04G033590/Soltu.DM.07G017190/Soltu.DM.04G024100/Soltu.DM.07G017220/Soltu.DM.07G017210/Soltu.DM.09G018310	8
2	GO:0009789	positive regulation of abscisic acid-activated signaling pathway	6/295	55/12756	0.001627115	0.092273812	0.086931644	Soltu.DM.07G017180/Soltu.DM.04G033590/Soltu.DM.07G017190/Soltu.DM.07G017220/Soltu.DM.07G017210/Soltu.DM.09G018310	6
3	GO:0009738	abscisic acid-activated signaling pathway	7/295	130/12756	0.031316037	0.37579244	0.354036036	Soltu.DM.09G019250/Soltu.DM.07G017180/Soltu.DM.04G033590/Soltu.DM.06G026960/Soltu.DM.07G017190/Soltu.DM.07G017220/Soltu.DM.07G017210	7
4	GO:0071215	cellular response to abscisic acid stimulus	8/295	177/12756	0.05345215	0.482275034	0.454353847	Soltu.DM.09G019250/Soltu.DM.07G017180/Soltu.DM.04G033590/Soltu.DM.06G026960/Soltu.DM.07G017190/Soltu.DM.08G002280/Soltu.DM.07G017220/Soltu.DM.07G017210	8
5	GO:0046345	abscisic acid catabolic process	2/295	17/12756	0.057693519	0.492605257	0.464086005	Soltu.DM.07G013900/Soltu.DM.08G020150	2
6	GO:0009687	abscisic acid metabolic	2/295	40/12756	0.236298065	0.634729845	0.597982328	Soltu.DM.07G013900/Soltu.DM.08G020150	2

		process							
7	GO:0009788	negative regulation of abscisic acid-activated signaling pathway	2/295	61/12756	0.413832802	0.715145237	0.673742092	Soltu.DM.04G005970/Soltu.DM.04G024100	2
8	GO:0009809	lignin biosynthetic process	7/295	73/12756	0.001456522	0.092273812	0.086931644	Soltu.DM.04G027660/Soltu.DM.10G000640/Soltu.DM.11G002650/Soltu.DM.03G011790/Soltu.DM.03G002800/Soltu.DM.02G024380/Soltu.DM.01G044300	7
9	GO:0009694	jasmonic acid metabolic process	8/295	31/12756	3.70E-07	0.000443542	0.000417863	Soltu.DM.03G024680/Soltu.DM.03G024660/Soltu.DM.04G034690/Soltu.DM.10G027770/Soltu.DM.03G024670/Soltu.DM.12G004930/Soltu.DM.02G025590/Soltu.DM.09G018310	8
10	GO:1902457	negative regulation of stomatal opening	4/295	6/12756	4.05E-06	0.002432265	0.00229145	Soltu.DM.07G017180/Soltu.DM.07G017190/Soltu.DM.07G017220/Soltu.DM.07G017210	4
11	GO:0009741	response to brassinosteroid	11/295	112/12756	5.55E-05	0.022181418	0.02089723	Soltu.DM.04G029270/Soltu.DM.03G017780/Soltu.DM.04G030010/Soltu.DM.08G022190/Soltu.DM.04G030060/Soltu.DM.12G028730/Soltu.DM.04G030020/Soltu.DM.04G030040/Soltu.DM.10G026020/Soltu.DM.03G017800/Soltu.DM.04G030050	11
12	GO:0048480	stigma development	3/295	5/12756	0.000118293	0.035487864	0.033433304	Soltu.DM.03G024680/Soltu.DM.03G024660/Soltu.DM.03G024670	3
13	GO:2000038	regulation of stomatal complex development	4/295	14/12756	0.000233585	0.056060283	0.052814688	Soltu.DM.07G017180/Soltu.DM.07G017190/Soltu.DM.07G017220/Soltu.DM.07G017210	4
14	GO:1902456	regulation of stomatal opening	4/295	17/12756	0.000525745	0.090285582	0.085058522	Soltu.DM.07G017180/Soltu.DM.07G017190/Soltu.DM.07G017220/Soltu.DM.07G017210	4
15	GO:0042537	benzene-containing compound metabolic process	7/295	63/12756	0.000601741	0.090285582	0.085058522	Soltu.DM.07G022460/Soltu.DM.06G028410/Soltu.DM.08G002280/Soltu.DM.05G007640/Soltu.DM.07G022490/Soltu.DM.02G008550/Soltu.DM.07G022510	7
16	GO:0009808	lignin metabolic process	8/295	82/12756	0.000601904	0.090285582	0.085058522	Soltu.DM.07G028550/Soltu.DM.10G000640/Soltu.DM.06G032850/Soltu.DM.11G002650/Soltu.DM.03G011790/Soltu.DM.03G002800/Soltu.DM.02G024380/Soltu.DM.03G031830	8
17	GO:0071462	cellular response to water	6/295	52/12756	0.001210645	0.092273812	0.086931644	Soltu.DM.01G040570/Soltu.DM.10G005360/Soltu.DM.07G022460/Soltu.DM.08G002280/Soltu.DM.07G0224	6

18	GO:0009635	response to herbicide	4/295	21/12756	0.001229133	0.092273812	0.086931644	Soltu.DM.07G024910/Soltu.DM.07G022460/Soltu.DM.07G022490/Soltu.DM.07G022510	4
19	GO:0080167	response to karrikin	11/295	161/12756	0.001296858	0.092273812	0.086931644	Soltu.DM.03G027330/Soltu.DM.12G007510/Soltu.DM.03G020490/Soltu.DM.01G025080/Soltu.DM.06G032850/Soltu.DM.06G012630/Soltu.DM.07G022050/Soltu.DM.07G022460/Soltu.DM.08G002280/Soltu.DM.07G022490/Soltu.DM.07G022510	11
20	GO:0009698	phenylpropanoid metabolic process	11/295	163/12756	0.00143271	0.092273812	0.086931644	Soltu.DM.07G028550/Soltu.DM.05G021610/Soltu.DM.10G000640/Soltu.DM.06G032850/Soltu.DM.11G002650/Soltu.DM.03G011790/Soltu.DM.03G002800/Soltu.DM.10G026580/Soltu.DM.02G024380/Soltu.DM.05G025440/Soltu.DM.03G031830	11
21	GO:1901421	positive regulation of response to alcohol	6/295	55/12756	0.001627115	0.092273812	0.086931644	Soltu.DM.07G017180/Soltu.DM.04G033590/Soltu.DM.07G017190/Soltu.DM.07G017220/Soltu.DM.07G017210/Soltu.DM.09G018310	6
22	GO:1905959	positive regulation of cellular response to alcohol	6/295	55/12756	0.001627115	0.092273812	0.086931644	Soltu.DM.07G017180/Soltu.DM.04G033590/Soltu.DM.07G017190/Soltu.DM.07G017220/Soltu.DM.07G017210/Soltu.DM.09G018310	6
23	GO:0042447	hormone catabolic process	4/295	23/12756	0.00175359	0.092273812	0.086931644	Soltu.DM.07G022710/Soltu.DM.07G022720/Soltu.DM.04G011550/Soltu.DM.06G028410	4
24	GO:0009695	jasmonic acid biosynthetic process	4/295	24/12756	0.002066433	0.092273812	0.086931644	Soltu.DM.04G034690/Soltu.DM.12G004930/Soltu.DM.02G025590/Soltu.DM.09G018310	4
25	GO:1900057	positive regulation of leaf senescence	4/295	24/12756	0.002066433	0.092273812	0.086931644	Soltu.DM.07G017180/Soltu.DM.07G017190/Soltu.DM.07G017220/Soltu.DM.07G017210	4
26	GO:1905623	positive regulation of leaf development	4/295	24/12756	0.002066433	0.092273812	0.086931644	Soltu.DM.07G017180/Soltu.DM.07G017190/Soltu.DM.07G017220/Soltu.DM.07G017210	4
27	GO:0009699	phenylpropanoid biosynthetic process	10/295	147/12756	0.002198461	0.092273812	0.086931644	Soltu.DM.07G028550/Soltu.DM.05G021610/Soltu.DM.10G000640/Soltu.DM.11G002650/Soltu.DM.03G011790/Soltu.DM.03G002800/Soltu.DM.10G026580/Soltu.DM.02G024380/Soltu.DM.05G025440/Soltu.DM.03G031830	10
28	GO:0018973	trinitrotoluene metabolic process	3/295	12/12756	0.002306845	0.092273812	0.086931644	Soltu.DM.07G022460/Soltu.DM.07G022490/Soltu.DM.07G022510	3

29	GO:0018974	2,4,6-trinitrotoluene metabolic process	3/295	12/12756	0.002306845	0.092273812	0.086931644	Soltu.DM.07G022460/Soltu.DM.07G022490/Soltu.DM.07G022510	3
30	GO:0019326	nitrotoluene metabolic process	3/295	12/12756	0.002306845	0.092273812	0.086931644	Soltu.DM.07G022460/Soltu.DM.07G022490/Soltu.DM.07G022510	3
31	GO:0046256	2,4,6-trinitrotoluene catabolic process	3/295	12/12756	0.002306845	0.092273812	0.086931644	Soltu.DM.07G022460/Soltu.DM.07G022490/Soltu.DM.07G022510	3
32	GO:0046260	trinitrotoluene catabolic process	3/295	12/12756	0.002306845	0.092273812	0.086931644	Soltu.DM.07G022460/Soltu.DM.07G022490/Soltu.DM.07G022510	3
33	GO:0046263	nitrotoluene catabolic process	3/295	12/12756	0.002306845	0.092273812	0.086931644	Soltu.DM.07G022460/Soltu.DM.07G022490/Soltu.DM.07G022510	3
34	GO:0072490	toluene-containing compound metabolic process	3/295	12/12756	0.002306845	0.092273812	0.086931644	Soltu.DM.07G022460/Soltu.DM.07G022490/Soltu.DM.07G022510	3
35	GO:0072491	toluene-containing compound catabolic process	3/295	12/12756	0.002306845	0.092273812	0.086931644	Soltu.DM.07G022460/Soltu.DM.07G022490/Soltu.DM.07G022510	3
36	GO:1901141	regulation of lignin biosynthetic process	3/295	12/12756	0.002306845	0.092273812	0.086931644	Soltu.DM.03G035710/Soltu.DM.07G003530/Soltu.DM.07G003550	3
37	GO:0016143	S-glycoside metabolic process	7/295	82/12756	0.002850291	0.103646962	0.097646348	Soltu.DM.03G016740/Soltu.DM.03G035710/Soltu.DM.02G027330/Soltu.DM.03G016750/Soltu.DM.04G005970/Soltu.DM.05G007640/Soltu.DM.05G019830	7
38	GO:0019757	glycosinolate metabolic process	7/295	82/12756	0.002850291	0.103646962	0.097646348	Soltu.DM.03G016740/Soltu.DM.03G035710/Soltu.DM.02G027330/Soltu.DM.03G016750/Soltu.DM.04G005970/Soltu.DM.05G007640/Soltu.DM.05G019830	7
39	GO:0019760	glucosinolate metabolic process	7/295	82/12756	0.002850291	0.103646962	0.097646348	Soltu.DM.03G016740/Soltu.DM.03G035710/Soltu.DM.02G027330/Soltu.DM.03G016750/Soltu.DM.04G005970/Soltu.DM.05G007640/Soltu.DM.05G019830	7
40	GO:0009642	response to light intensity	10/295	157/12756	0.00354386	0.125077402	0.117836078	Soltu.DM.01G034240/Soltu.DM.07G028550/Soltu.DM.10G000640/Soltu.DM.07G024910/Soltu.DM.07G0035	10

								30/Soltu.DM.07G003550/Soltu.DM.05G026160/Soltu.DM.02G025590/Soltu.DM.07G000550/Soltu.DM.01G008290	
41	GO:0010200	response to chitin	9/295	133/12756	0.003736176	0.128097472	0.120681302	Soltu.DM.08G028970/Soltu.DM.03G014570/Soltu.DM.03G014580/Soltu.DM.03G014560/Soltu.DM.07G019030/Soltu.DM.10G026020/Soltu.DM.03G027640/Soltu.DM.01G046560/Soltu.DM.07G019630	9
42	GO:0009646	response to absence of light	5/295	47/12756	0.004431988	0.146985551	0.138475862	Soltu.DM.07G028550/Soltu.DM.10G000640/Soltu.DM.07G003530/Soltu.DM.07G003550/Soltu.DM.02G025590	5
43	GO:0010411	xyloglucan metabolic process	3/295	15/12756	0.004532055	0.146985551	0.138475862	Soltu.DM.01G003570/Soltu.DM.08G029290/Soltu.DM.09G018910	3
44	GO:0006833	water transport	5/295	48/12756	0.004855674	0.149405366	0.140755582	Soltu.DM.05G026690/Soltu.DM.10G029590/Soltu.DM.03G012810/Soltu.DM.06G018020/Soltu.DM.06G006130	5
45	GO:0042044	fluid transport	5/295	48/12756	0.004855674	0.149405366	0.140755582	Soltu.DM.05G026690/Soltu.DM.10G029590/Soltu.DM.03G012810/Soltu.DM.06G018020/Soltu.DM.06G006130	5
46	GO:0015706	nitrate transmembrane transport	4/295	31/12756	0.005390617	0.157774167	0.148639874	Soltu.DM.03G027330/Soltu.DM.02G010790/Soltu.DM.10G010160/Soltu.DM.01G031830	4
47	GO:1902025	nitrate import	4/295	31/12756	0.005390617	0.157774167	0.148639874	Soltu.DM.03G027330/Soltu.DM.02G010790/Soltu.DM.10G010160/Soltu.DM.01G031830	4
48	GO:0042631	cellular response to water deprivation	5/295	50/12756	0.00578791	0.165368859	0.155794873	Soltu.DM.01G040570/Soltu.DM.07G022460/Soltu.DM.08G002280/Soltu.DM.07G022490/Soltu.DM.07G022510	5
49	GO:1902600	proton transmembrane transport	7/295	95/12756	0.006432266	0.179505085	0.169112685	Soltu.DM.03G027330/Soltu.DM.02G010790/Soltu.DM.10G010160/Soltu.DM.09G024150/Soltu.DM.01G031830/Soltu.DM.01G037640/Soltu.DM.02G006700	7
50	GO:0071365	cellular response to auxin stimulus	7/295	97/12756	0.007193069	0.196174596	0.18481712	Soltu.DM.12G023260/Soltu.DM.04G029270/Soltu.DM.03G035710/Soltu.DM.11G011180/Soltu.DM.06G026960/Soltu.DM.02G025590/Soltu.DM.12G022190	7
51	GO:0009825	multidimensional cell growth	5/295	53/12756	0.007412567	0.196351757	0.184984024	Soltu.DM.04G027320/Soltu.DM.07G019030/Soltu.DM.05G009050/Soltu.DM.03G011300/Soltu.DM.08G014180	5
52	GO:0009696	salicylic acid metabolic process	4/295	34/12756	0.007526817	0.196351757	0.184984024	Soltu.DM.06G028410/Soltu.DM.08G002280/Soltu.DM.05G007640/Soltu.DM.02G008550	4
53	GO:00	cellular	8/295	124/1275	0.00810	0.206976	0.194993511	Soltu.DM.09G027770/Soltu.DM.01G	8

	06073	glucan metabolic process		6	6574	353		040570/Soltu.DM.04G027320/Soltu.DM.05G006330/Soltu.DM.04G037250/Soltu.DM.01G003570/Soltu.DM.08G029290/Soltu.DM.09G018910	
54	GO:1900376	regulation of secondary metabolite biosynthetic process	4/295	35/12756	0.008346413	0.20735008	0.195345602	Soltu.DM.03G035710/Soltu.DM.07G003530/Soltu.DM.07G003550/Soltu.DM.12G022190	4
55	GO:0030587	sorocarp development	5/295	55/12756	0.008657261	0.20735008	0.195345602	Soltu.DM.07G017180/Soltu.DM.07G017190/Soltu.DM.07G017220/Soltu.DM.07G017210/Soltu.DM.01G028770	5
56	GO:0099120	socially cooperative development	5/295	55/12756	0.008657261	0.20735008	0.195345602	Soltu.DM.07G017180/Soltu.DM.07G017190/Soltu.DM.07G017220/Soltu.DM.07G017210/Soltu.DM.01G028770	5
57	GO:0055065	metal ion homeostasis	9/295	153/12756	0.009192003	0.20735008	0.195345602	Soltu.DM.05G021830/Soltu.DM.07G028550/Soltu.DM.10G010160/Soltu.DM.03G017590/Soltu.DM.10G000640/Soltu.DM.01G031830/Soltu.DM.07G002440/Soltu.DM.11G022440/Soltu.DM.01G037640	9
58	GO:0010167	response to nitrate	4/295	36/12756	0.009222339	0.20735008	0.195345602	Soltu.DM.02G010790/Soltu.DM.10G010160/Soltu.DM.01G031830/Soltu.DM.06G026960	4
59	GO:0010218	response to far red light	5/295	56/12756	0.009330754	0.20735008	0.195345602	Soltu.DM.12G007510/Soltu.DM.08G011110/Soltu.DM.09G021320/Soltu.DM.06G021700/Soltu.DM.02G025590	5
60	GO:0043455	regulation of secondary metabolic process	5/295	56/12756	0.009330754	0.20735008	0.195345602	Soltu.DM.03G035710/Soltu.DM.10G005360/Soltu.DM.07G003530/Soltu.DM.07G003550/Soltu.DM.12G022190	5
61	GO:0009734	auxin-activated signaling pathway	6/295	79/12756	0.009854521	0.209041265	0.196938876	Soltu.DM.12G023260/Soltu.DM.04G029270/Soltu.DM.11G011180/Soltu.DM.06G026960/Soltu.DM.02G025590/Soltu.DM.12G022190	6
62	GO:0097501	stress response to metal ion	2/295	7/12756	0.010365902	0.209041265	0.196938876	Soltu.DM.07G028550/Soltu.DM.10G000640	2
63	GO:0002213	defense response to insect	3/295	20/12756	0.010427439	0.209041265	0.196938876	Soltu.DM.03G024680/Soltu.DM.03G024660/Soltu.DM.03G024670	3
64	GO:2000762	regulation of phenylpropanoid metabolic process	3/295	20/12756	0.010427439	0.209041265	0.196938876	Soltu.DM.03G035710/Soltu.DM.07G003530/Soltu.DM.07G003550	3
65	GO:00	glucan	8/295	130/1275	0.01063	0.209041	0.196938876	Soltu.DM.09G027770/Soltu.DM.01G	8

	44042	metabolic process		6	1863	265		040570/Soltu.DM.04G027320/Soltu.DM.05G006330/Soltu.DM.04G037250/Soltu.DM.01G003570/Soltu.DM.08G029290/Soltu.DM.09G018910	
66	GO:0010114	response to red light	6/295	81/12756	0.011076289	0.209041265	0.196938876	Soltu.DM.08G011110/Soltu.DM.10G005360/Soltu.DM.09G021320/Soltu.DM.06G021700/Soltu.DM.02G025590/Soltu.DM.03G027640	6
67	GO:1901419	regulation of response to alcohol	8/295	131/12756	0.011103671	0.209041265	0.196938876	Soltu.DM.07G017180/Soltu.DM.04G005970/Soltu.DM.04G033590/Soltu.DM.07G017190/Soltu.DM.04G024100/Soltu.DM.07G017220/Soltu.DM.07G017210/Soltu.DM.09G018310	8
68	GO:1905957	regulation of cellular response to alcohol	8/295	131/12756	0.011103671	0.209041265	0.196938876	Soltu.DM.07G017180/Soltu.DM.04G005970/Soltu.DM.04G033590/Soltu.DM.07G017190/Soltu.DM.04G024100/Soltu.DM.07G017220/Soltu.DM.07G017210/Soltu.DM.09G018310	8
69	GO:0009631	cold acclimation	4/295	38/12756	0.011148867	0.209041265	0.196938876	Soltu.DM.02G024660/Soltu.DM.03G016740/Soltu.DM.03G016750/Soltu.DM.04G037380	4
70	GO:0055088	lipid homeostasis	5/295	59/12756	0.011566035	0.213526807	0.201164729	Soltu.DM.03G017780/Soltu.DM.06G004470/Soltu.DM.08G029860/Soltu.DM.08G020150/Soltu.DM.03G017800	5
71	GO:0010252	auxin homeostasis	4/295	39/12756	0.012202136	0.221857026	0.209012672	Soltu.DM.12G023260/Soltu.DM.10G026500/Soltu.DM.12G022190/Soltu.DM.03G031830	4
72	GO:0007623	circadian rhythm	7/295	108/12756	0.012617985	0.222670316	0.209778876	Soltu.DM.12G007510/Soltu.DM.06G026960/Soltu.DM.02G025590/Soltu.DM.03G027640/Soltu.DM.07G026780/Soltu.DM.10G027680/Soltu.DM.12G002630	7
73	GO:0048511	rhythmic process	7/295	108/12756	0.012617985	0.222670316	0.209778876	Soltu.DM.12G007510/Soltu.DM.06G026960/Soltu.DM.02G025590/Soltu.DM.03G027640/Soltu.DM.07G026780/Soltu.DM.10G027680/Soltu.DM.12G002630	7
74	GO:0005987	sucrose catabolic process	2/295	8/12756	0.013611534	0.23672233	0.223017353	Soltu.DM.05G006330/Soltu.DM.04G037250	2
75	GO:0016115	terpenoid catabolic process	4/295	42/12756	0.01573646	0.269767893	0.254149752	Soltu.DM.07G022710/Soltu.DM.07G013900/Soltu.DM.07G022720/Soltu.DM.08G020150	4
76	GO:0098661	inorganic anion transmembrane transport	5/295	64/12756	0.016055337	0.271357801	0.255647613	Soltu.DM.03G027330/Soltu.DM.02G010790/Soltu.DM.10G010160/Soltu.DM.01G031830/Soltu.DM.09G020160	5
77	GO:0008300	isoprenoid catabolic process	4/295	43/12756	0.017043012	0.275769175	0.259803592	Soltu.DM.07G022710/Soltu.DM.07G013900/Soltu.DM.07G022720/Soltu.DM.08G020150	4
78	GO:0010268	brassinosteroid homeostasis	4/295	43/12756	0.017043012	0.275769175	0.259803592	Soltu.DM.03G017780/Soltu.DM.06G004470/Soltu.DM.08G020150/Soltu.DM.03G017800	4

79	GO:0046886	positive regulation of hormone biosynthetic process	2/295	9/12756	0.017235573	0.275769175	0.259803592	Soltu.DM.06G009270/Soltu.DM.10G005360	2
80	GO:0090480	purine nucleotide-sugar transmembrane transport	2/295	9/12756	0.017235573	0.275769175	0.259803592	Soltu.DM.12G004580/Soltu.DM.12G007520	2
81	GO:0010119	regulation of stomatal movement	6/295	90/12756	0.01791425	0.282856574	0.266480667	Soltu.DM.07G017180/Soltu.DM.07G024910/Soltu.DM.07G017190/Soltu.DM.01G037640/Soltu.DM.07G017220/Soltu.DM.07G017210	6
82	GO:0016131	brassinosteroid metabolic process	4/295	44/12756	0.018415405	0.28699332	0.270377918	Soltu.DM.03G017780/Soltu.DM.06G004470/Soltu.DM.08G020150/Soltu.DM.03G017800	4
83	GO:1901264	carbohydrate derivative transport	6/295	92/12756	0.019755163	0.289681078	0.272910068	Soltu.DM.03G027330/Soltu.DM.12G004580/Soltu.DM.12G007520/Soltu.DM.03G032350/Soltu.DM.04G030440/Soltu.DM.01G008290	6
84	GO:0034614	cellular response to reactive oxygen species	5/295	68/12756	0.020386378	0.289681078	0.272910068	Soltu.DM.07G028550/Soltu.DM.06G012170/Soltu.DM.10G000640/Soltu.DM.08G002280/Soltu.DM.02G012210	5
85	GO:1901657	glycosyl compound metabolic process	9/295	175/12756	0.020564617	0.289681078	0.272910068	Soltu.DM.03G016740/Soltu.DM.03G035710/Soltu.DM.02G027330/Soltu.DM.03G016750/Soltu.DM.04G005970/Soltu.DM.03G002800/Soltu.DM.05G007640/Soltu.DM.02G008550/Soltu.DM.05G019830	9
86	GO:0006415	translational termination	2/295	10/12756	0.021218904	0.289681078	0.272910068	Soltu.DM.08G019530/Soltu.DM.07G001240	2
87	GO:0009969	xyloglucan biosynthetic process	2/295	10/12756	0.021218904	0.289681078	0.272910068	Soltu.DM.08G029290/Soltu.DM.09G018910	2
88	GO:0032352	positive regulation of hormone metabolic process	2/295	10/12756	0.021218904	0.289681078	0.272910068	Soltu.DM.06G009270/Soltu.DM.10G005360	2
89	GO:0034311	diol metabolic process	2/295	10/12756	0.021218904	0.289681078	0.272910068	Soltu.DM.06G033540/Soltu.DM.08G014180	2
90	GO:00	diol	2/295	10/12756	0.02121	0.289681	0.272910068	Soltu.DM.06G033540/Soltu.DM.08G	2

	34312	biosynthetic process			8904	078		014180	
91	GO:1990641	response to iron ion starvation	2/295	10/12756	0.021218904	0.289681078	0.272910068	Soltu.DM.07G028550/Soltu.DM.10G000640	2
92	GO:0005983	starch catabolic process	3/295	26/12756	0.02148468	0.289681078	0.272910068	Soltu.DM.09G027770/Soltu.DM.05G006330/Soltu.DM.04G037250	3
93	GO:0071577	zinc ion transmembrane transport	3/295	26/12756	0.02148468	0.289681078	0.272910068	Soltu.DM.07G002440/Soltu.DM.07G027100/Soltu.DM.02G012210	3
94	GO:0071732	cellular response to nitric oxide	3/295	26/12756	0.02148468	0.289681078	0.272910068	Soltu.DM.07G028550/Soltu.DM.10G000640/Soltu.DM.02G012210	3
95	GO:0051703	biological process involved in intraspecies interaction between organisms	5/295	70/12756	0.022812486	0.302451317	0.284940978	Soltu.DM.07G017180/Soltu.DM.07G017190/Soltu.DM.07G017220/Soltu.DM.07G017210/Soltu.DM.01G028770	5
96	GO:0018958	phenol-containing compound metabolic process	4/295	47/12756	0.022935892	0.302451317	0.284940978	Soltu.DM.06G028410/Soltu.DM.08G002280/Soltu.DM.05G007640/Soltu.DM.02G008550	4
97	GO:0009407	toxin catabolic process	3/295	27/12756	0.023766237	0.309994393	0.292047349	Soltu.DM.07G022460/Soltu.DM.07G022490/Soltu.DM.07G022510	3
98	GO:0016128	phytosteroid metabolic process	4/295	48/12756	0.024579548	0.317155453	0.298793822	Soltu.DM.03G017780/Soltu.DM.06G004470/Soltu.DM.08G020150/Soltu.DM.03G017800	4
99	GO:0016103	diterpenoid catabolic process	2/295	11/12756	0.025543085	0.322649495	0.303969787	Soltu.DM.07G022710/Soltu.DM.07G022720	2
100	GO:0045487	gibberellin catabolic process	2/295	11/12756	0.025543085	0.322649495	0.303969787	Soltu.DM.07G022710/Soltu.DM.07G022720	2
101	GO:0006829	zinc ion transport	3/295	28/12756	0.026173793	0.32717241	0.308230849	Soltu.DM.07G002440/Soltu.DM.07G027100/Soltu.DM.02G012210	3
102	GO:0010030	positive regulation of seed germination	4/295	50/12756	0.028075653	0.347327666	0.327219222	Soltu.DM.03G033490/Soltu.DM.10G005360/Soltu.DM.08G002280/Soltu.DM.05G007640	4
103	GO:0055075	potassium ion	3/295	29/12756	0.028707064	0.351515065	0.331164193	Soltu.DM.10G010160/Soltu.DM.01G031830/Soltu.DM.01G037640	3

		homeostasis							
104	GO:0008202	steroid metabolic process	6/295	101/12756	0.029629658	0.359147364	0.338354622	Soltu.DM.03G017780/Soltu.DM.04G034690/Soltu.DM.06G004470/Soltu.DM.01G003630/Soltu.DM.08G020150/Soltu.DM.03G017800	6
105	GO:0009404	toxin metabolic process	4/295	52/12756	0.03185316	0.378453383	0.356542924	Soltu.DM.07G022460/Soltu.DM.12G022190/Soltu.DM.07G022490/Soltu.DM.07G022510	4
106	GO:0051347	positive regulation of transferase activity	6/295	103/12756	0.032195055	0.378765355	0.356836834	Soltu.DM.07G017180/Soltu.DM.06G026960/Soltu.DM.07G017190/Soltu.DM.07G017220/Soltu.DM.07G015530/Soltu.DM.07G017210	6
107	GO:0009682	induced systemic resistance	3/295	31/12756	0.034148586	0.383383624	0.36118773	Soltu.DM.07G028550/Soltu.DM.10G000640/Soltu.DM.02G025590	3
108	GO:0071731	response to nitric oxide	3/295	31/12756	0.034148586	0.383383624	0.36118773	Soltu.DM.07G028550/Soltu.DM.10G000640/Soltu.DM.02G012210	3
109	GO:0097366	response to bronchodilator	3/295	31/12756	0.034148586	0.383383624	0.36118773	Soltu.DM.07G028550/Soltu.DM.10G000640/Soltu.DM.02G012210	3
110	GO:0018874	benzoate metabolic process	2/295	13/12756	0.035143499	0.383383624	0.36118773	Soltu.DM.08G002280/Soltu.DM.05G007640	2
111	GO:0042547	cell wall modification involved in multidimensional cell growth	2/295	13/12756	0.035143499	0.383383624	0.36118773	Soltu.DM.05G009050/Soltu.DM.03G011300	2
112	GO:0046352	disaccharide catabolic process	2/295	13/12756	0.035143499	0.383383624	0.36118773	Soltu.DM.05G006330/Soltu.DM.04G037250	2
113	GO:0070413	trehalose metabolism in response to stress	2/295	13/12756	0.035143499	0.383383624	0.36118773	Soltu.DM.04G012960/Soltu.DM.07G001730	2
114	GO:2000652	regulation of secondary cell wall biogenesis	2/295	13/12756	0.035143499	0.383383624	0.36118773	Soltu.DM.07G028550/Soltu.DM.10G000640	2
115	GO:1900055	regulation of leaf senescence	4/295	54/12756	0.035914722	0.38826727	0.365788638	Soltu.DM.07G017180/Soltu.DM.07G017190/Soltu.DM.07G017220/Soltu.DM.07G017210	4
116	GO:0055080	monoatomic cation	9/295	194/12756	0.036565376	0.391771891	0.36909036	Soltu.DM.05G021830/Soltu.DM.07G028550/Soltu.DM.10G010160/Soltu.	9

		homeostasis						DM.03G017590/Soltu.DM.10G000640/Soltu.DM.01G031830/Soltu.DM.07G002440/Soltu.DM.11G022440/Soltu.DM.01G037640	
117	GO:0031098	stress-activated protein kinase signaling cascade	4/295	55/12756	0.038052563	0.40409801	0.380702862	Soltu.DM.07G017180/Soltu.DM.07G017190/Soltu.DM.07G017220/Soltu.DM.07G017210	4
118	GO:0009313	oligosaccharide catabolic process	2/295	14/12756	0.04038605	0.421419649	0.397021669	Soltu.DM.05G006330/Soltu.DM.04G037250	2
119	GO:0009866	induced systemic resistance, ethylene mediated signaling pathway	2/295	14/12756	0.04038605	0.421419649	0.397021669	Soltu.DM.07G028550/Soltu.DM.10G000640	2
120	GO:0009751	response to salicylic acid	9/295	198/12756	0.04078989	0.421964384	0.397534867	Soltu.DM.07G028550/Soltu.DM.01G021910/Soltu.DM.10G000640/Soltu.DM.06G026960/Soltu.DM.06G028410/Soltu.DM.10G026020/Soltu.DM.02G025590/Soltu.DM.08G006060/Soltu.DM.09G018910	9
121	GO:0005984	disaccharide metabolic process	4/295	58/12756	0.044895357	0.460465203	0.433806691	Soltu.DM.04G012960/Soltu.DM.05G006330/Soltu.DM.04G037250/Soltu.DM.07G001730	4
122	GO:0046482	para-aminobenzoic acid metabolic process	2/295	15/12756	0.045902049	0.462877804	0.436079616	Soltu.DM.08G002280/Soltu.DM.05G007640	2
123	GO:0051365	cellular response to potassium ion starvation	2/295	15/12756	0.045902049	0.462877804	0.436079616	Soltu.DM.07G028550/Soltu.DM.10G000640	2
124	GO:0016999	antibiotic metabolic process	5/295	85/12756	0.046959106	0.469282393	0.442113412	Soltu.DM.06G028410/Soltu.DM.08G002280/Soltu.DM.01G019990/Soltu.DM.05G007640/Soltu.DM.02G008550	5
125	GO:0009072	aromatic amino acid metabolic process	4/295	59/12756	0.047319308	0.469282393	0.442113412	Soltu.DM.08G002280/Soltu.DM.04G018630/Soltu.DM.04G023360/Soltu.DM.05G007640	4
126	GO:0009627	systemic acquired resistance	5/295	86/12756	0.04895704	0.473369204	0.445963618	Soltu.DM.12G006650/Soltu.DM.03G035710/Soltu.DM.01G021910/Soltu.DM.04G030790/Soltu.DM.02G008550	5
127	GO:0032147	activation of protein	4/295	60/12756	0.049814583	0.473369204	0.445963618	Soltu.DM.07G017180/Soltu.DM.07G017190/Soltu.DM.07G017220/Soltu.	4

		kinase activity						DM.07G017210	
128	GO:0046916	cellular transition metal ion homeostasis	4/295	60/12756	0.049814583	0.473369204	0.445963618	Soltu.DM.05G021830/Soltu.DM.03G017590/Soltu.DM.07G002440/Soltu.DM.11G022440	4
129	GO:0016998	cell wall macromolecule catabolic process	3/295	36/12756	0.04989564	0.473369204	0.445963618	Soltu.DM.06G006580/Soltu.DM.09G005280/Soltu.DM.06G006590	3
130	GO:1902170	cellular response to reactive nitrogen species	3/295	36/12756	0.04989564	0.473369204	0.445963618	Soltu.DM.07G028550/Soltu.DM.10G000640/Soltu.DM.02G012210	3
131	GO:0009269	response to desiccation	2/295	16/12756	0.051676138	0.473369204	0.445963618	Soltu.DM.01G040570/Soltu.DM.05G018810	2
132	GO:0010065	primary meristem tissue development	2/295	16/12756	0.051676138	0.473369204	0.445963618	Soltu.DM.04G019530/Soltu.DM.07G020980	2
133	GO:0015691	cadmium ion transport	2/295	16/12756	0.051676138	0.473369204	0.445963618	Soltu.DM.03G035710/Soltu.DM.02G012210	2
134	GO:0052482	defense response by cell wall thickening	2/295	16/12756	0.051676138	0.473369204	0.445963618	Soltu.DM.03G035710/Soltu.DM.05G007640	2
135	GO:0052544	defense response by callose deposition in cell wall	2/295	16/12756	0.051676138	0.473369204	0.445963618	Soltu.DM.03G035710/Soltu.DM.05G007640	2
136	GO:0034599	cellular response to oxidative stress	6/295	116/12756	0.052377556	0.476159604	0.448592469	Soltu.DM.07G028550/Soltu.DM.06G012170/Soltu.DM.10G000640/Soltu.DM.03G032350/Soltu.DM.08G002280/Soltu.DM.02G012210	6
137	GO:0002239	response to oomycetes	4/295	62/12756	0.055018261	0.489051207	0.460737716	Soltu.DM.07G003530/Soltu.DM.07G003550/Soltu.DM.06G028410/Soltu.DM.01G044600	4
138	GO:0042545	cell wall modification	4/295	62/12756	0.055018261	0.489051207	0.460737716	Soltu.DM.03G035710/Soltu.DM.05G009050/Soltu.DM.03G011300/Soltu.DM.05G007640	4
139	GO:0097306	cellular response to alcohol	8/295	179/12756	0.05637406	0.492605257	0.464086005	Soltu.DM.09G019250/Soltu.DM.07G017180/Soltu.DM.04G033590/Soltu.DM.06G026960/Soltu.DM.07G017190/Soltu.DM.08G002280/Soltu.DM.	8

								07G017220/Soltu.DM.07G017210	
140	GO:0016107	sesquiterpenoid catabolic process	2/295	17/12756	0.057693519	0.492605257	0.464086005	Soltu.DM.07G013900/Soltu.DM.08G020150	2
141	GO:0043290	apocarotenoid catabolic process	2/295	17/12756	0.057693519	0.492605257	0.464086005	Soltu.DM.07G013900/Soltu.DM.08G020150	2
142	GO:0046940	nucleoside monophosphate phosphorylation	2/295	17/12756	0.057693519	0.492605257	0.464086005	Soltu.DM.09G006670/Soltu.DM.05G011440	2
143	GO:0009967	positive regulation of signal transduction	7/295	149/12756	0.057881118	0.492605257	0.464086005	Soltu.DM.07G017180/Soltu.DM.04G033590/Soltu.DM.07G017190/Soltu.DM.10G026020/Soltu.DM.07G017220/Soltu.DM.07G017210/Soltu.DM.09G018310	7
144	GO:0048582	positive regulation of post-embryonic development	6/295	120/12756	0.059848737	0.505763974	0.476482902	Soltu.DM.12G007510/Soltu.DM.03G033490/Soltu.DM.10G005360/Soltu.DM.06G019760/Soltu.DM.08G002280/Soltu.DM.05G007640	6
145	GO:2000022	regulation of jasmonic acid mediated signaling pathway	3/295	39/12756	0.060758917	0.509865035	0.480346533	Soltu.DM.03G032770/Soltu.DM.10G026020/Soltu.DM.10G022640	3
146	GO:0044036	cell wall macromolecule metabolic process	6/295	121/12756	0.061810783	0.515089858	0.485268866	Soltu.DM.06G006580/Soltu.DM.09G005280/Soltu.DM.06G006590/Soltu.DM.01G003570/Soltu.DM.08G029290/Soltu.DM.09G018910	6
147	GO:0042542	response to hydrogen peroxide	6/295	122/12756	0.063810588	0.521958645	0.491739987	Soltu.DM.12G007510/Soltu.DM.06G026960/Soltu.DM.08G002280/Soltu.DM.02G025590/Soltu.DM.08G006060/Soltu.DM.01G028770	6
148	GO:0052542	defense response by callose deposition	2/295	18/12756	0.063939934	0.521958645	0.491739987	Soltu.DM.03G035710/Soltu.DM.05G007640	2
149	GO:0071474	cellular hyperosmotic response	2/295	18/12756	0.063939934	0.521958645	0.491739987	Soltu.DM.08G002280/Soltu.DM.02G020550	2
150	GO:0009251	glucan catabolic process	3/295	40/12756	0.064605553	0.523828809	0.493501878	Soltu.DM.09G027770/Soltu.DM.05G006330/Soltu.DM.04G037250	3
151	GO:0009637	response to blue	5/295	95/12756	0.069165874	0.557040599	0.52479088	Soltu.DM.12G007510/Soltu.DM.06G021700/Soltu.DM.02G025590/Soltu.	5

		light						DM.10G012930/Soltu.DM.05G019830	
152	GO:0062197	cellular response to chemical stress	7/295	156/12756	0.070371979	0.562975829	0.530382492	Soltu.DM.07G028550/Soltu.DM.06G012170/Soltu.DM.10G000640/Soltu.DM.03G032350/Soltu.DM.08G002280/Soltu.DM.02G020550/Soltu.DM.02G012210	7
153	GO:0006972	hyperosmotic response	5/295	96/12756	0.071657698	0.569465153	0.536496118	Soltu.DM.08G019590/Soltu.DM.08G002280/Soltu.DM.05G018810/Soltu.DM.02G020550/Soltu.DM.01G046560	5
154	GO:0010029	regulation of seed germination	6/295	126/12756	0.072187232	0.5698992	0.536905035	Soltu.DM.09G019250/Soltu.DM.03G033490/Soltu.DM.10G005360/Soltu.DM.04G037380/Soltu.DM.08G002280/Soltu.DM.05G007640	6
155	GO:0071555	cell wall organization	8/295	190/12756	0.074243309	0.582300466	0.548588333	Soltu.DM.10G029580/Soltu.DM.03G035710/Soltu.DM.02G020130/Soltu.DM.12G028730/Soltu.DM.05G018810/Soltu.DM.05G009050/Soltu.DM.03G011300/Soltu.DM.05G007640	8
156	GO:0023056	positive regulation of signaling	7/295	159/12756	0.076181318	0.59362066	0.559253148	Soltu.DM.07G017180/Soltu.DM.04G033590/Soltu.DM.07G017190/Soltu.DM.10G026020/Soltu.DM.07G017220/Soltu.DM.07G017210/Soltu.DM.09G018310	7
157	GO:0006749	glutathione metabolic process	3/295	43/12756	0.076793801	0.594532652	0.56011234	Soltu.DM.07G022460/Soltu.DM.07G022490/Soltu.DM.07G022510	3
158	GO:0010051	xylem and phloem pattern formation	4/295	70/12756	0.078620535	0.600921285	0.566131106	Soltu.DM.08G003320/Soltu.DM.04G029270/Soltu.DM.12G005490/Soltu.DM.08G022670	4
159	GO:0048440	carpel development	4/295	70/12756	0.078620535	0.600921285	0.566131106	Soltu.DM.03G024680/Soltu.DM.03G024660/Soltu.DM.03G024670/Soltu.DM.10G026020	4
160	GO:0015698	inorganic anion transport	5/295	99/12756	0.079424978	0.603227679	0.568303971	Soltu.DM.03G027330/Soltu.DM.02G010790/Soltu.DM.10G010160/Soltu.DM.01G031830/Soltu.DM.09G020160	5
161	GO:0010043	response to zinc ion	3/295	44/12756	0.081065391	0.607990431	0.572790985	Soltu.DM.07G028550/Soltu.DM.10G000640/Soltu.DM.07G002440	3
162	GO:1901616	organic hydroxy compound catabolic process	3/295	44/12756	0.081065391	0.607990431	0.572790985	Soltu.DM.07G013900/Soltu.DM.06G028410/Soltu.DM.08G020150	3
163	GO:0034219	carbohydrate transmembrane transport	4/295	72/12756	0.085196973	0.626834574	0.590544151	Soltu.DM.09G024150/Soltu.DM.02G014590/Soltu.DM.02G006700/Soltu.DM.01G008290	4
164	GO:1900140	regulation of seedling developm	6/295	132/12756	0.085878766	0.626834574	0.590544151	Soltu.DM.09G019250/Soltu.DM.03G033490/Soltu.DM.10G005360/Soltu.DM.04G037380/Soltu.DM.08G002280/Soltu.DM.05G007640	6

		ent							
165	GO:0003333	amino acid transmembrane transport	4/295	73/12756	0.088582902	0.626834574	0.590544151	Soltu.DM.03G003280/Soltu.DM.11G011180/Soltu.DM.05G003990/Soltu.DM.03G011160	4
166	GO:0006865	amino acid transport	4/295	73/12756	0.088582902	0.626834574	0.590544151	Soltu.DM.03G003280/Soltu.DM.11G011180/Soltu.DM.05G003990/Soltu.DM.03G011160	4
167	GO:0009311	oligosaccharide metabolic process	4/295	73/12756	0.088582902	0.626834574	0.590544151	Soltu.DM.04G012960/Soltu.DM.05G006330/Soltu.DM.04G037250/Soltu.DM.07G001730	4
168	GO:0090627	plant epidermal cell differentiation	7/295	165/12756	0.088619579	0.626834574	0.590544151	Soltu.DM.05G021390/Soltu.DM.12G005490/Soltu.DM.09G026810/Soltu.DM.11G023760/Soltu.DM.04G001370/Soltu.DM.08G029290/Soltu.DM.08G023170	7
169	GO:0010647	positive regulation of cell communication	7/295	166/12756	0.090798292	0.626834574	0.590544151	Soltu.DM.07G017180/Soltu.DM.04G033590/Soltu.DM.07G017190/Soltu.DM.10G026020/Soltu.DM.07G017220/Soltu.DM.07G017210/Soltu.DM.09G018310	7
170	GO:0051017	actin filament bundle assembly	2/295	22/12756	0.090948864	0.626834574	0.590544151	Soltu.DM.07G026780/Soltu.DM.01G028770	2
171	GO:0061572	actin filament bundle organization	2/295	22/12756	0.090948864	0.626834574	0.590544151	Soltu.DM.07G026780/Soltu.DM.01G028770	2
172	GO:0120256	olefinic compound catabolic process	2/295	22/12756	0.090948864	0.626834574	0.590544151	Soltu.DM.07G013900/Soltu.DM.08G020150	2
173	GO:1903338	regulation of cell wall organization or biogenesis	2/295	22/12756	0.090948864	0.626834574	0.590544151	Soltu.DM.07G028550/Soltu.DM.10G000640	2
174	GO:0044247	cellular polysaccharide catabolic process	3/295	47/12756	0.094474335	0.626834574	0.590544151	Soltu.DM.09G027770/Soltu.DM.05G006330/Soltu.DM.04G037250	3
175	GO:0008643	carbohydrate transport	4/295	75/12756	0.095546218	0.626834574	0.590544151	Soltu.DM.09G024150/Soltu.DM.02G014590/Soltu.DM.02G006700/Soltu.DM.01G008290	4
176	GO:0009250	glucan biosynthetic process	4/295	75/12756	0.095546218	0.626834574	0.590544151	Soltu.DM.01G040570/Soltu.DM.04G027320/Soltu.DM.08G029290/Soltu.DM.09G018910	4
177	GO:00	cellular	4/295	75/12756	0.09554	0.626834	0.590544151	Soltu.DM.07G028550/Soltu.DM.10G	4

	71281	response to iron ion			6218	574		000640/Soltu.DM.08G027110/Soltu.DM.02G012210	
178	GO:0071248	cellular response to metal ion	5/295	105/12756	0.096249085	0.626834574	0.590544151	Soltu.DM.07G028550/Soltu.DM.10G000640/Soltu.DM.08G027110/Soltu.DM.03G032350/Soltu.DM.02G012210	5
179	GO:0005992	trehalose biosynthetic process	2/295	23/12756	0.098144429	0.626834574	0.590544151	Soltu.DM.04G012960/Soltu.DM.07G001730	2
180	GO:0034620	cellular response to unfolded protein	2/295	23/12756	0.098144429	0.626834574	0.590544151	Soltu.DM.08G019590/Soltu.DM.08G029860	2
181	GO:0052386	cell wall thickening	2/295	23/12756	0.098144429	0.626834574	0.590544151	Soltu.DM.03G035710/Soltu.DM.05G007640	2
182	GO:0052543	callose deposition in cell wall	2/295	23/12756	0.098144429	0.626834574	0.590544151	Soltu.DM.03G035710/Soltu.DM.05G007640	2
183	GO:2000573	positive regulation of DNA biosynthetic process	2/295	23/12756	0.098144429	0.626834574	0.590544151	Soltu.DM.06G026960/Soltu.DM.07G015530	2
184	GO:0000041	transition metal ion transport	4/295	76/12756	0.099121915	0.626834574	0.590544151	Soltu.DM.03G035710/Soltu.DM.07G002440/Soltu.DM.07G027100/Soltu.DM.02G012210	4
185	GO:0009813	flavonoid biosynthetic process	4/295	76/12756	0.099121915	0.626834574	0.590544151	Soltu.DM.09G028560/Soltu.DM.09G028570/Soltu.DM.06G028410/Soltu.DM.04G001370	4
186	GO:0015804	neutral amino acid transport	2/295	24/12756	0.105493961	0.626834574	0.590544151	Soltu.DM.03G003280/Soltu.DM.05G003990	2
187	GO:0055082	cellular chemical homeostasis	7/295	173/12756	0.10688325	0.626834574	0.590544151	Soltu.DM.05G021830/Soltu.DM.03G017590/Soltu.DM.11G011180/Soltu.DM.07G002440/Soltu.DM.11G022440/Soltu.DM.08G029860/Soltu.DM.01G037640	7
188	GO:0048236	plant-type sporogenesis	3/295	50/12756	0.108723373	0.626834574	0.590544151	Soltu.DM.06G009270/Soltu.DM.11G009620/Soltu.DM.08G022670	3
189	GO:2000024	regulation of leaf development	4/295	79/12756	0.11021437	0.626834574	0.590544151	Soltu.DM.07G017180/Soltu.DM.07G017190/Soltu.DM.07G017220/Soltu.DM.07G017210	4
190	GO:0002188	translation reinitiation	1/295	5/12756	0.110422345	0.626834574	0.590544151	Soltu.DM.08G019530	1
191	GO:0006004	fucose metabolic process	1/295	5/12756	0.110422345	0.626834574	0.590544151	Soltu.DM.09G018910	1

192	GO:0006083	acetate metabolic process	1/295	5/12756	0.110422345	0.626834574	0.590544151	Soltu.DM.01G019990	1
193	GO:0008277	regulation of G protein-coupled receptor signaling pathway	1/295	5/12756	0.110422345	0.626834574	0.590544151	Soltu.DM.06G033540	1
194	GO:0009643	photosynthetic acclimation	1/295	5/12756	0.110422345	0.626834574	0.590544151	Soltu.DM.01G008290	1
195	GO:0010068	protoderm histogenesis	1/295	5/12756	0.110422345	0.626834574	0.590544151	Soltu.DM.07G020980	1
196	GO:0010196	nonphotochemical quenching	1/295	5/12756	0.110422345	0.626834574	0.590544151	Soltu.DM.06G021700	1
197	GO:0015713	phosphoglycerate transmembrane transport	1/295	5/12756	0.110422345	0.626834574	0.590544151	Soltu.DM.01G008290	1
198	GO:0015837	amine transport	1/295	5/12756	0.110422345	0.626834574	0.590544151	Soltu.DM.06G018020	1
199	GO:0015843	methylammonium transport	1/295	5/12756	0.110422345	0.626834574	0.590544151	Soltu.DM.06G018020	1
200	GO:0019605	butyrate metabolic process	1/295	5/12756	0.110422345	0.626834574	0.590544151	Soltu.DM.01G019990	1
201	GO:0051764	actin crosslink formation	1/295	5/12756	0.110422345	0.626834574	0.590544151	Soltu.DM.07G026780	1
202	GO:0061088	regulation of sequestering of zinc ion	1/295	5/12756	0.110422345	0.626834574	0.590544151	Soltu.DM.07G002440	1
203	GO:0070574	cadmium ion transmembrane transport	1/295	5/12756	0.110422345	0.626834574	0.590544151	Soltu.DM.03G035710	1
204	GO:0071366	cellular response to indolebutyric acid stimulus	1/295	5/12756	0.110422345	0.626834574	0.590544151	Soltu.DM.03G035710	1
205	GO:1904502	regulation of lipophagy	1/295	5/12756	0.110422345	0.626834574	0.590544151	Soltu.DM.08G014180	1
206	GO:19	positive	1/295	5/12756	0.11042	0.626834	0.590544151	Soltu.DM.08G014180	1

	04504	regulation of lipophagy			2345	574			
207	GO:1990066	energy quenching	1/295	5/12756	0.110422345	0.626834574	0.590544151	Soltu.DM.06G021700	1
208	GO:0005991	trehalose metabolic process	2/295	25/12756	0.11298655	0.626834574	0.590544151	Soltu.DM.04G012960/Soltu.DM.07G001730	2
209	GO:0005982	starch metabolic process	3/295	51/12756	0.113648019	0.626834574	0.590544151	Soltu.DM.09G027770/Soltu.DM.05G006330/Soltu.DM.04G037250	3
210	GO:0045860	positive regulation of protein kinase activity	4/295	80/12756	0.114030476	0.626834574	0.590544151	Soltu.DM.07G017180/Soltu.DM.07G017190/Soltu.DM.07G017220/Soltu.DM.07G017210	4
211	GO:0051338	regulation of transferase activity	6/295	143/12756	0.114406911	0.626834574	0.590544151	Soltu.DM.07G017180/Soltu.DM.06G026960/Soltu.DM.07G017190/Soltu.DM.07G017220/Soltu.DM.07G015530/Soltu.DM.07G017210	6
212	GO:0033674	positive regulation of kinase activity	4/295	81/12756	0.117904243	0.626834574	0.590544151	Soltu.DM.07G017180/Soltu.DM.07G017190/Soltu.DM.07G017220/Soltu.DM.07G017210	4
213	GO:0042538	hyperosmotic salinity response	4/295	81/12756	0.117904243	0.626834574	0.590544151	Soltu.DM.08G019590/Soltu.DM.08G002280/Soltu.DM.05G018810/Soltu.DM.01G046560	4
214	GO:0010025	wax biosynthetic process	2/295	26/12756	0.120611706	0.626834574	0.590544151	Soltu.DM.10G005470/Soltu.DM.10G005260	2
215	GO:0010305	leaf vascular tissue pattern formation	2/295	26/12756	0.120611706	0.626834574	0.590544151	Soltu.DM.04G029270/Soltu.DM.08G022670	2
216	GO:0010345	suberin biosynthetic process	2/295	26/12756	0.120611706	0.626834574	0.590544151	Soltu.DM.10G026580/Soltu.DM.05G025440	2
217	GO:0019432	triglyceride biosynthetic process	2/295	26/12756	0.120611706	0.626834574	0.590544151	Soltu.DM.12G002120/Soltu.DM.10G005470	2
218	GO:0042335	cuticle development	2/295	26/12756	0.120611706	0.626834574	0.590544151	Soltu.DM.07G019030/Soltu.DM.07G020980	2
219	GO:0051445	regulation of meiotic cell cycle	2/295	26/12756	0.120611706	0.626834574	0.590544151	Soltu.DM.11G009620/Soltu.DM.11G023760	2
220	GO:0016125	sterol metabolic process	4/295	82/12756	0.121834644	0.626834574	0.590544151	Soltu.DM.04G034690/Soltu.DM.06G004470/Soltu.DM.01G003630/Soltu.DM.08G020150	4
221	GO:0070417	cellular response	3/295	53/12756	0.123744321	0.626834574	0.590544151	Soltu.DM.01G040570/Soltu.DM.07G003530/Soltu.DM.07G003550	3

		to cold							
222	GO:1901568	fatty acid derivative metabolic process	3/295	53/12756	0.123744321	0.626834574	0.590544151	Soltu.DM.04G034690/Soltu.DM.10G005470/Soltu.DM.10G005260	3
223	GO:2000030	regulation of response to red or far red light	3/295	53/12756	0.123744321	0.626834574	0.590544151	Soltu.DM.07G022460/Soltu.DM.07G022490/Soltu.DM.07G022510	3
224	GO:0055076	transition metal ion homeostasis	4/295	83/12756	0.125820631	0.626834574	0.590544151	Soltu.DM.05G021830/Soltu.DM.03G017590/Soltu.DM.07G002440/Soltu.DM.11G022440	4
225	GO:0080147	root hair cell development	4/295	83/12756	0.125820631	0.626834574	0.590544151	Soltu.DM.12G005490/Soltu.DM.09G026810/Soltu.DM.04G001370/Soltu.DM.08G023170	4
226	GO:0071466	cellular response to xenobiotic stimulus	6/295	148/12756	0.12877789	0.626834574	0.590544151	Soltu.DM.07G028550/Soltu.DM.01G021910/Soltu.DM.10G000640/Soltu.DM.08G002280/Soltu.DM.02G012210/Soltu.DM.09G018910	6
227	GO:0006097	glyoxylate cycle	1/295	6/12756	0.131003116	0.626834574	0.590544151	Soltu.DM.01G019990	1
228	GO:0015675	nickel cation transport	1/295	6/12756	0.131003116	0.626834574	0.590544151	Soltu.DM.02G012210	1
229	GO:0015717	triose phosphate transport	1/295	6/12756	0.131003116	0.626834574	0.590544151	Soltu.DM.01G008290	1
230	GO:0016239	positive regulation of macroautophagy	1/295	6/12756	0.131003116	0.626834574	0.590544151	Soltu.DM.08G014180	1
231	GO:0019373	epoxygenase P450 pathway	1/295	6/12756	0.131003116	0.626834574	0.590544151	Soltu.DM.04G034690	1
232	GO:0030038	contractile actin filament bundle assembly	1/295	6/12756	0.131003116	0.626834574	0.590544151	Soltu.DM.01G028770	1
233	GO:0031270	pseudopodium retraction	1/295	6/12756	0.131003116	0.626834574	0.590544151	Soltu.DM.01G028770	1
234	GO:0032060	bleb assembly	1/295	6/12756	0.131003116	0.626834574	0.590544151	Soltu.DM.01G028770	1
235	GO:0033298	contractile vacuole organization	1/295	6/12756	0.131003116	0.626834574	0.590544151	Soltu.DM.01G028770	1
236	GO:0035436	triose phosphat	1/295	6/12756	0.131003116	0.626834574	0.590544151	Soltu.DM.01G008290	1

		e transmembrane transport							
237	GO:0042344	indole glucosinolate catabolic process	1/295	6/12756	0.131003116	0.626834574	0.590544151	Soltu.DM.03G035710	1
238	GO:0042873	aldonate transmembrane transport	1/295	6/12756	0.131003116	0.626834574	0.590544151	Soltu.DM.01G008290	1
239	GO:0046847	filopodium assembly	1/295	6/12756	0.131003116	0.626834574	0.590544151	Soltu.DM.01G028770	1
240	GO:0051639	actin filament network formation	1/295	6/12756	0.131003116	0.626834574	0.590544151	Soltu.DM.07G026780	1
241	GO:0052390	induction by symbiont of host innate immune response	1/295	6/12756	0.131003116	0.626834574	0.590544151	Soltu.DM.01G024680	1
242	GO:0052559	induction by symbiont of host immune response	1/295	6/12756	0.131003116	0.626834574	0.590544151	Soltu.DM.01G024680	1
243	GO:0060327	cytoplasmic actin-based contraction involved in cell motility	1/295	6/12756	0.131003116	0.626834574	0.590544151	Soltu.DM.01G028770	1
244	GO:0060328	cytoplasmic actin-based contraction involved in forward cell motility	1/295	6/12756	0.131003116	0.626834574	0.590544151	Soltu.DM.01G028770	1
245	GO:0098719	sodium ion import across plasma membrane	1/295	6/12756	0.131003116	0.626834574	0.590544151	Soltu.DM.01G037640	1

246	GO:1900036	positive regulation of cellular response to heat	1/295	6/12756	0.131003116	0.626834574	0.590544151	Soltu.DM.07G019630	1
247	GO:1900262	regulation of DNA-directed DNA polymerase activity	1/295	6/12756	0.131003116	0.626834574	0.590544151	Soltu.DM.07G015530	1
248	GO:1900264	positive regulation of DNA-directed DNA polymerase activity	1/295	6/12756	0.131003116	0.626834574	0.590544151	Soltu.DM.07G015530	1
249	GO:1902022	L-lysine transport	1/295	6/12756	0.131003116	0.626834574	0.590544151	Soltu.DM.05G003990	1
250	GO:1903401	L-lysine transmembrane transport	1/295	6/12756	0.131003116	0.626834574	0.590544151	Soltu.DM.05G003990	1
251	GO:0000160	phosphorelay signal transduction system	4/295	85/12756	0.13395506	0.626834574	0.590544151	Soltu.DM.07G028550/Soltu.DM.10G000640/Soltu.DM.03G027640/Soltu.DM.10G027680	4
252	GO:0048467	gynoecium development	4/295	85/12756	0.13395506	0.626834574	0.590544151	Soltu.DM.03G024680/Soltu.DM.03G024660/Soltu.DM.03G024670/Soltu.DM.10G026020	4
253	GO:0010193	response to ozone	3/295	55/12756	0.134152588	0.626834574	0.590544151	Soltu.DM.11G024590/Soltu.DM.06G029230/Soltu.DM.11G024600	3
254	GO:0034293	sexual sporulation	3/295	55/12756	0.134152588	0.626834574	0.590544151	Soltu.DM.06G009270/Soltu.DM.11G009620/Soltu.DM.08G022670	3
255	GO:0005985	sucrose metabolic process	2/295	28/12756	0.13621977	0.626834574	0.590544151	Soltu.DM.05G006330/Soltu.DM.04G037250	2
256	GO:0009612	response to mechanical stimulus	2/295	28/12756	0.13621977	0.626834574	0.590544151	Soltu.DM.12G028730/Soltu.DM.01G028770	2
257	GO:0010166	wax metabolic process	2/295	28/12756	0.13621977	0.626834574	0.590544151	Soltu.DM.10G005470/Soltu.DM.10G005260	2
258	GO:0010039	response to iron ion	4/295	86/12756	0.138101304	0.626834574	0.590544151	Soltu.DM.07G028550/Soltu.DM.10G000640/Soltu.DM.08G027110/Soltu.DM.02G012210	4
259	GO:0009644	response to high light intensity	4/295	87/12756	0.142298742	0.626834574	0.590544151	Soltu.DM.01G034240/Soltu.DM.07G024910/Soltu.DM.05G026160/Soltu.DM.07G000550	4
260	GO:0009685	gibberellin	4/295	87/12756	0.142298742	0.626834574	0.590544151	Soltu.DM.07G022710/Soltu.DM.07G022720/Soltu.DM.08G027110/Soltu.	4

		metabolic process						DM.06G004470	
261	GO:0072329	monocarboxylic acid catabolic process	4/295	87/12756	0.142298742	0.626834574	0.590544151	Soltu.DM.07G013900/Soltu.DM.06G028410/Soltu.DM.08G020150/Soltu.DM.09G018310	4
262	GO:0009768	photosynthesis, light harvesting in photosystem I	2/295	29/12756	0.144183674	0.626834574	0.590544151	Soltu.DM.07G024910/Soltu.DM.06G021700	2
263	GO:0015780	nucleotide-sugar transmembrane transport	2/295	29/12756	0.144183674	0.626834574	0.590544151	Soltu.DM.12G004580/Soltu.DM.12G007520	2
264	GO:0033037	polysaccharide localization	2/295	29/12756	0.144183674	0.626834574	0.590544151	Soltu.DM.03G035710/Soltu.DM.05G007640	2
265	GO:0046351	disaccharide biosynthetic process	2/295	29/12756	0.144183674	0.626834574	0.590544151	Soltu.DM.04G012960/Soltu.DM.07G001730	2
266	GO:0052545	callose localization	2/295	29/12756	0.144183674	0.626834574	0.590544151	Soltu.DM.03G035710/Soltu.DM.05G007640	2
267	GO:0010410	hemicellulose metabolic process	3/295	57/12756	0.144851836	0.626834574	0.590544151	Soltu.DM.01G003570/Soltu.DM.08G029290/Soltu.DM.09G018910	3
268	GO:0034637	cellular carbohydrate biosynthetic process	6/295	154/12756	0.14710984	0.626834574	0.590544151	Soltu.DM.04G012960/Soltu.DM.01G040570/Soltu.DM.04G027320/Soltu.DM.08G029290/Soltu.DM.07G001730/Soltu.DM.09G018910	6
269	GO:0030162	regulation of proteolysis	5/295	121/12756	0.148940208	0.626834574	0.590544151	Soltu.DM.06G026960/Soltu.DM.08G027150/Soltu.DM.04G003450/Soltu.DM.12G005510/Soltu.DM.08G006060	5
270	GO:0010235	guard mother cell cytokinesis	1/295	7/12756	0.151109319	0.626834574	0.590544151	Soltu.DM.11G023760	1
271	GO:0014074	response to purine-containing compound	1/295	7/12756	0.151109319	0.626834574	0.590544151	Soltu.DM.01G028770	1
272	GO:0015808	L-alanine transport	1/295	7/12756	0.151109319	0.626834574	0.590544151	Soltu.DM.05G003990	1

273	GO:0019336	phenol-containing compound catabolic process	1/295	7/12756	0.151109319	0.626834574	0.590544151	Soltu.DM.06G028410	1
274	GO:0019632	shikimate metabolic process	1/295	7/12756	0.151109319	0.626834574	0.590544151	Soltu.DM.04G023360	1
275	GO:0031034	myosin filament assembly	1/295	7/12756	0.151109319	0.626834574	0.590544151	Soltu.DM.01G028770	1
276	GO:0032328	alanine transport	1/295	7/12756	0.151109319	0.626834574	0.590544151	Soltu.DM.05G003990	1
277	GO:0046459	short-chain fatty acid metabolic process	1/295	7/12756	0.151109319	0.626834574	0.590544151	Soltu.DM.01G019990	1
278	GO:0051016	barbed-end actin filament capping	1/295	7/12756	0.151109319	0.626834574	0.590544151	Soltu.DM.12G019570	1
279	GO:0051591	response to cAMP	1/295	7/12756	0.151109319	0.626834574	0.590544151	Soltu.DM.01G028770	1
280	GO:0110126	phloem loading	1/295	7/12756	0.151109319	0.626834574	0.590544151	Soltu.DM.03G027330	1
281	GO:1900367	positive regulation of defense response to insect	1/295	7/12756	0.151109319	0.626834574	0.590544151	Soltu.DM.02G025590	1
282	GO:2000014	regulation of endosperm development	1/295	7/12756	0.151109319	0.626834574	0.590544151	Soltu.DM.05G021010	1
283	GO:0051301	cell division	7/295	190/12756	0.151764369	0.626834574	0.590544151	Soltu.DM.03G014570/Soltu.DM.03G014580/Soltu.DM.03G014560/Soltu.DM.04G027320/Soltu.DM.01G036010/Soltu.DM.11G023760/Soltu.DM.01G028770	7
284	GO:0140115	export across plasma membrane	2/295	30/12756	0.152242111	0.626834574	0.590544151	Soltu.DM.11G011180/Soltu.DM.10G026500	2
285	GO:1901658	glycosyl compound catabolic process	2/295	30/12756	0.152242111	0.626834574	0.590544151	Soltu.DM.03G035710/Soltu.DM.02G008550	2
286	GO:0016054	organic acid catabolic	6/295	156/12756	0.153471622	0.626834574	0.590544151	Soltu.DM.07G022710/Soltu.DM.07G013900/Soltu.DM.07G022720/Soltu.DM.06G028410/Soltu.DM.08G0201	6

		process						50/Soltu.DM.09G018310	
287	GO:0046395	carboxylic acid catabolic process	6/295	156/12756	0.153471622	0.626834574	0.590544151	Soltu.DM.07G022710/Soltu.DM.07G013900/Soltu.DM.07G022720/Soltu.DM.06G028410/Soltu.DM.08G020150/Soltu.DM.09G018310	6
288	GO:0098754	detoxification	6/295	156/12756	0.153471622	0.626834574	0.590544151	Soltu.DM.06G012170/Soltu.DM.02G020130/Soltu.DM.07G022460/Soltu.DM.05G018810/Soltu.DM.07G022490/Soltu.DM.07G022510	6
289	GO:0009073	aromatic amino acid family biosynthetic process	2/295	31/12756	0.160386492	0.626834574	0.590544151	Soltu.DM.04G018630/Soltu.DM.04G023360	2
290	GO:0046460	neutral lipid biosynthetic process	2/295	31/12756	0.160386492	0.626834574	0.590544151	Soltu.DM.12G002120/Soltu.DM.10G005470	2
291	GO:0046463	acylglycerol biosynthetic process	2/295	31/12756	0.160386492	0.626834574	0.590544151	Soltu.DM.12G002120/Soltu.DM.10G005470	2
292	GO:0044275	cellular carbohydrate catabolic process	3/295	61/12756	0.167039264	0.626834574	0.590544151	Soltu.DM.09G027770/Soltu.DM.05G006330/Soltu.DM.04G037250	3
293	GO:0006855	xenobiotic transmembrane transport	5/295	126/12756	0.167493563	0.626834574	0.590544151	Soltu.DM.05G026690/Soltu.DM.03G035710/Soltu.DM.05G003990/Soltu.DM.03G032350/Soltu.DM.11G003270	5
294	GO:0046173	polyol biosynthetic process	2/295	32/12756	0.168608573	0.626834574	0.590544151	Soltu.DM.06G033540/Soltu.DM.08G014180	2
295	GO:0070988	demethylation	2/295	32/12756	0.168608573	0.626834574	0.590544151	Soltu.DM.08G022190/Soltu.DM.01G003630	2
296	GO:0006734	NADH metabolic process	1/295	8/12756	0.170751859	0.626834574	0.590544151	Soltu.DM.02G018700	1
297	GO:0006949	syncytium formation	1/295	8/12756	0.170751859	0.626834574	0.590544151	Soltu.DM.07G019710	1
298	GO:0009819	drought recovery	1/295	8/12756	0.170751859	0.626834574	0.590544151	Soltu.DM.05G026160	1
299	GO:0009823	cytokinin catabolic process	1/295	8/12756	0.170751859	0.626834574	0.590544151	Soltu.DM.04G011550	1
300	GO:0009961	response to 1-aminocyclopropane-1-carboxylic acid	1/295	8/12756	0.170751859	0.626834574	0.590544151	Soltu.DM.02G024660	1

301	GO:0010205	photoinhibition	1/295	8/12756	0.170751859	0.626834574	0.590544151	Soltu.DM.07G000550	1
302	GO:0016094	polyprenol biosynthetic process	1/295	8/12756	0.170751859	0.626834574	0.590544151	Soltu.DM.10G022710	1
303	GO:0016145	S-glycoside catabolic process	1/295	8/12756	0.170751859	0.626834574	0.590544151	Soltu.DM.03G035710	1
304	GO:0019759	glycosinolate catabolic process	1/295	8/12756	0.170751859	0.626834574	0.590544151	Soltu.DM.03G035710	1
305	GO:0019762	glucosinolate catabolic process	1/295	8/12756	0.170751859	0.626834574	0.590544151	Soltu.DM.03G035710	1
306	GO:0031407	oxylipin metabolic process	1/295	8/12756	0.170751859	0.626834574	0.590544151	Soltu.DM.04G034690	1
307	GO:0033120	positive regulation of RNA splicing	1/295	8/12756	0.170751859	0.626834574	0.590544151	Soltu.DM.12G005490	1
308	GO:0043155	negative regulation of photosynthesis, light reaction	1/295	8/12756	0.170751859	0.626834574	0.590544151	Soltu.DM.07G000550	1
309	GO:0046487	glyoxylate metabolic process	1/295	8/12756	0.170751859	0.626834574	0.590544151	Soltu.DM.01G019990	1
310	GO:0046683	response to organophosphorus	1/295	8/12756	0.170751859	0.626834574	0.590544151	Soltu.DM.01G028770	1
311	GO:0051176	positive regulation of sulfur metabolic process	1/295	8/12756	0.170751859	0.626834574	0.590544151	Soltu.DM.12G022190	1
312	GO:0051290	protein heterotetramerization	1/295	8/12756	0.170751859	0.626834574	0.590544151	Soltu.DM.03G012810	1
313	GO:0051552	flavone metabolic process	1/295	8/12756	0.170751859	0.626834574	0.590544151	Soltu.DM.06G028410	1
314	GO:0051553	flavone biosynthetic process	1/295	8/12756	0.170751859	0.626834574	0.590544151	Soltu.DM.06G028410	1
315	GO:00	cellular	1/295	8/12756	0.17075	0.626834	0.590544151	Soltu.DM.03G032350	1

	71277	response to calcium ion			1859	574			
316	GO:1902476	chloride transmembrane transport	1/295	8/12756	0.170751859	0.626834574	0.590544151	Soltu.DM.02G010790	1
317	GO:0001934	positive regulation of protein phosphorylation	4/295	94/12756	0.173016754	0.626834574	0.590544151	Soltu.DM.07G017180/Soltu.DM.07G017190/Soltu.DM.07G017220/Soltu.DM.07G017210	4
318	GO:0018107	peptidyl-threonine phosphorylation	2/295	33/12756	0.176900442	0.626834574	0.590544151	Soltu.DM.06G002800/Soltu.DM.10G022340	2
319	GO:0018210	peptidyl-threonine modification	2/295	33/12756	0.176900442	0.626834574	0.590544151	Soltu.DM.06G002800/Soltu.DM.10G022340	2
320	GO:1901570	fatty acid derivative biosynthetic process	2/295	33/12756	0.176900442	0.626834574	0.590544151	Soltu.DM.10G005470/Soltu.DM.10G005260	2
321	GO:2000278	regulation of DNA biosynthetic process	2/295	33/12756	0.176900442	0.626834574	0.590544151	Soltu.DM.06G026960/Soltu.DM.07G015530	2
322	GO:0042327	positive regulation of phosphorylation	4/295	95/12756	0.177581628	0.626834574	0.590544151	Soltu.DM.07G017180/Soltu.DM.07G017190/Soltu.DM.07G017220/Soltu.DM.07G017210	4
323	GO:0048767	root hair elongation	3/295	63/12756	0.178485704	0.626834574	0.590544151	Soltu.DM.12G005490/Soltu.DM.09G026810/Soltu.DM.08G023170	3
324	GO:0030003	cellular monoatomic cation homeostasis	5/295	129/12756	0.179042669	0.626834574	0.590544151	Soltu.DM.05G021830/Soltu.DM.03G017590/Soltu.DM.07G002440/Soltu.DM.11G022440/Soltu.DM.01G037640	5
325	GO:0010562	positive regulation of phosphorus metabolic process	4/295	96/12756	0.182187017	0.626834574	0.590544151	Soltu.DM.07G017180/Soltu.DM.07G017190/Soltu.DM.07G017220/Soltu.DM.07G017210	4
326	GO:0045937	positive regulation of phosphate metabolic process	4/295	96/12756	0.182187017	0.626834574	0.590544151	Soltu.DM.07G017180/Soltu.DM.07G017190/Soltu.DM.07G017220/Soltu.DM.07G017210	4

327	GO:0010053	root epidermal cell differentiation	5/295	130/12756	0.182957751	0.626834574	0.590544151	Soltu.DM.12G005490/Soltu.DM.09G026810/Soltu.DM.04G001370/Soltu.DM.08G029290/Soltu.DM.08G023170	5
328	GO:0046496	nicotinamide nucleotide metabolic process	3/295	64/12756	0.184288075	0.626834574	0.590544151	Soltu.DM.02G018700/Soltu.DM.08G014620/Soltu.DM.12G004480	3
329	GO:0006986	response to unfolded protein	2/295	34/12756	0.185254509	0.626834574	0.590544151	Soltu.DM.08G019590/Soltu.DM.08G029860	2
330	GO:0008645	hexose transmembrane transport	2/295	34/12756	0.185254509	0.626834574	0.590544151	Soltu.DM.09G024150/Soltu.DM.02G006700	2
331	GO:0030837	negative regulation of actin filament polymerization	2/295	34/12756	0.185254509	0.626834574	0.590544151	Soltu.DM.12G019570/Soltu.DM.01G028770	2
332	GO:0046323	glucose import	2/295	34/12756	0.185254509	0.626834574	0.590544151	Soltu.DM.09G024150/Soltu.DM.02G006700	2
333	GO:1904659	glucose transmembrane transport	2/295	34/12756	0.185254509	0.626834574	0.590544151	Soltu.DM.09G024150/Soltu.DM.02G006700	2
334	GO:0002792	negative regulation of peptide secretion	1/295	9/12756	0.189941395	0.626834574	0.590544151	Soltu.DM.08G027150	1
335	GO:0006670	sphingosine metabolic process	1/295	9/12756	0.189941395	0.626834574	0.590544151	Soltu.DM.08G014180	1
336	GO:0009831	plant-type cell wall modification involved in multidimensional cell growth	1/295	9/12756	0.189941395	0.626834574	0.590544151	Soltu.DM.05G009050	1
337	GO:0010344	seed oilbody biogenesis	1/295	9/12756	0.189941395	0.626834574	0.590544151	Soltu.DM.02G033270	1
338	GO:0010371	regulation of gibberellin	1/295	9/12756	0.189941395	0.626834574	0.590544151	Soltu.DM.10G005360	1

		biosynthetic process							
339	GO:0015801	aromatic amino acid transport	1/295	9/12756	0.189941395	0.626834574	0.590544151	Soltu.DM.03G003280	1
340	GO:0019430	removal of superoxide radicals	1/295	9/12756	0.189941395	0.626834574	0.590544151	Soltu.DM.06G012170	1
341	GO:0031268	pseudopodium organization	1/295	9/12756	0.189941395	0.626834574	0.590544151	Soltu.DM.01G028770	1
342	GO:0032796	uropod organization	1/295	9/12756	0.189941395	0.626834574	0.590544151	Soltu.DM.01G028770	1
343	GO:0033273	response to vitamin	1/295	9/12756	0.189941395	0.626834574	0.590544151	Soltu.DM.02G025590	1
344	GO:0033356	UDP-L-arabinose metabolic process	1/295	9/12756	0.189941395	0.626834574	0.590544151	Soltu.DM.04G000320	1
345	GO:0034461	uropod retraction	1/295	9/12756	0.189941395	0.626834574	0.590544151	Soltu.DM.01G028770	1
346	GO:0042343	indole glucosinolate metabolic process	1/295	9/12756	0.189941395	0.626834574	0.590544151	Soltu.DM.03G035710	1
347	GO:0042436	indole-containing compound catabolic process	1/295	9/12756	0.189941395	0.626834574	0.590544151	Soltu.DM.03G035710	1
348	GO:0046512	sphingosine biosynthetic process	1/295	9/12756	0.189941395	0.626834574	0.590544151	Soltu.DM.08G014180	1
349	GO:0048578	positive regulation of long-day photoperiodism, flowering	1/295	9/12756	0.189941395	0.626834574	0.590544151	Soltu.DM.12G007510	1
350	GO:0050709	negative regulation of protein secretion	1/295	9/12756	0.189941395	0.626834574	0.590544151	Soltu.DM.08G027150	1
351	GO:0051048	negative regulation of secretion	1/295	9/12756	0.189941395	0.626834574	0.590544151	Soltu.DM.08G027150	1

352	GO:0051055	negative regulation of lipid biosynthetic process	1/295	9/12756	0.189941395	0.626834574	0.590544151	Soltu.DM.08G029860	1
353	GO:0051924	regulation of calcium ion transport	1/295	9/12756	0.189941395	0.626834574	0.590544151	Soltu.DM.02G020550	1
354	GO:0052031	modulation by symbiont of host defense response	1/295	9/12756	0.189941395	0.626834574	0.590544151	Soltu.DM.01G024680	1
355	GO:0052167	modulation by symbiont of host innate immune response	1/295	9/12756	0.189941395	0.626834574	0.590544151	Soltu.DM.01G024680	1
356	GO:0052173	response to defenses of other organism	1/295	9/12756	0.189941395	0.626834574	0.590544151	Soltu.DM.01G024680	1
357	GO:0052200	response to host defenses	1/295	9/12756	0.189941395	0.626834574	0.590544151	Soltu.DM.01G024680	1
358	GO:0052553	modulation by symbiont of host immune response	1/295	9/12756	0.189941395	0.626834574	0.590544151	Soltu.DM.01G024680	1
359	GO:0052572	response to host immune response	1/295	9/12756	0.189941395	0.626834574	0.590544151	Soltu.DM.01G024680	1
360	GO:0071450	cellular response to oxygen radical	1/295	9/12756	0.189941395	0.626834574	0.590544151	Soltu.DM.06G012170	1
361	GO:0071451	cellular response to superoxide	1/295	9/12756	0.189941395	0.626834574	0.590544151	Soltu.DM.06G012170	1
362	GO:0075136	response to host	1/295	9/12756	0.189941395	0.626834574	0.590544151	Soltu.DM.01G024680	1
363	GO:1903531	negative regulation of secretion by cell	1/295	9/12756	0.189941395	0.626834574	0.590544151	Soltu.DM.08G027150	1

364	GO:2000068	regulation of defense response to insect	1/295	9/12756	0.189941395	0.626834574	0.590544151	Soltu.DM.02G025590	1
365	GO:0019362	pyridine nucleotide metabolic process	3/295	65/12756	0.190139821	0.626834574	0.590544151	Soltu.DM.02G018700/Soltu.DM.08G014620/Soltu.DM.12G004480	3
366	GO:0022411	cellular component disassembly	3/295	65/12756	0.190139821	0.626834574	0.590544151	Soltu.DM.08G019530/Soltu.DM.07G001240/Soltu.DM.01G028770	3
367	GO:0006633	fatty acid biosynthetic process	4/295	98/12756	0.191514425	0.627916148	0.591563108	Soltu.DM.06G009560/Soltu.DM.04G008710/Soltu.DM.10G026580/Soltu.DM.07G000550	4
368	GO:0016101	diterpenoid metabolic process	4/295	98/12756	0.191514425	0.627916148	0.591563108	Soltu.DM.07G022710/Soltu.DM.07G022720/Soltu.DM.08G027110/Soltu.DM.06G004470	4
369	GO:0015749	monosaccharide transmembrane transport	2/295	35/12756	0.193663498	0.628097831	0.591734272	Soltu.DM.09G024150/Soltu.DM.02G006700	2
370	GO:0032272	negative regulation of protein polymerization	2/295	35/12756	0.193663498	0.628097831	0.591734272	Soltu.DM.12G019570/Soltu.DM.01G028770	2
371	GO:0046164	alcohol catabolic process	2/295	35/12756	0.193663498	0.628097831	0.591734272	Soltu.DM.07G013900/Soltu.DM.08G020150	2
372	GO:0072348	sulfur compound transport	2/295	35/12756	0.193663498	0.628097831	0.591734272	Soltu.DM.03G027330/Soltu.DM.09G020160	2
373	GO:0071241	cellular response to inorganic substance	5/295	133/12756	0.194889182	0.630369323	0.593874257	Soltu.DM.07G028550/Soltu.DM.10G000640/Soltu.DM.08G027110/Soltu.DM.03G032350/Soltu.DM.02G012210	5
374	GO:0006641	triglyceride metabolic process	2/295	36/12756	0.202120431	0.634729845	0.597982328	Soltu.DM.12G002120/Soltu.DM.10G005470	2
375	GO:0010150	leaf senescence	5/295	135/12756	0.202991378	0.634729845	0.597982328	Soltu.DM.10G010160/Soltu.DM.01G031830/Soltu.DM.12G027330/Soltu.DM.06G028410/Soltu.DM.05G026160	5
376	GO:0009664	plant-type cell wall organization	5/295	136/12756	0.207084648	0.634729845	0.597982328	Soltu.DM.10G029580/Soltu.DM.02G020130/Soltu.DM.12G028730/Soltu.DM.05G018810/Soltu.DM.05G009050	5

377	GO:0048653	anther development	3/295	68/12756	0.20796624	0.634729845	0.597982328	Soltu.DM.03G024680/Soltu.DM.03G024660/Soltu.DM.03G024670	3
378	GO:0072524	pyridine-containing compound metabolic process	3/295	68/12756	0.20796624	0.634729845	0.597982328	Soltu.DM.02G018700/Soltu.DM.08G014620/Soltu.DM.12G004480	3
379	GO:0000303	response to superoxide	1/295	10/12756	0.208688339	0.634729845	0.597982328	Soltu.DM.06G012170	1
380	GO:0006971	hypotonic response	1/295	10/12756	0.208688339	0.634729845	0.597982328	Soltu.DM.01G028770	1
381	GO:0009051	pentose-phosphate shunt, oxidative branch	1/295	10/12756	0.208688339	0.634729845	0.597982328	Soltu.DM.08G014620	1
382	GO:0010358	leaf shaping	1/295	10/12756	0.208688339	0.634729845	0.597982328	Soltu.DM.10G025140	1
383	GO:0015800	acidic amino acid transport	1/295	10/12756	0.208688339	0.634729845	0.597982328	Soltu.DM.05G003990	1
384	GO:0015813	L-glutamate transmembrane transport	1/295	10/12756	0.208688339	0.634729845	0.597982328	Soltu.DM.05G003990	1
385	GO:0045833	negative regulation of lipid metabolic process	1/295	10/12756	0.208688339	0.634729845	0.597982328	Soltu.DM.08G029860	1
386	GO:0051938	L-glutamate import	1/295	10/12756	0.208688339	0.634729845	0.597982328	Soltu.DM.05G003990	1
387	GO:0061615	glycolytic process through fructose-6-phosphate	1/295	10/12756	0.208688339	0.634729845	0.597982328	Soltu.DM.12G004480	1
388	GO:0080060	integument development	1/295	10/12756	0.208688339	0.634729845	0.597982328	Soltu.DM.10G026020	1
389	GO:1900034	regulation of cellular response to heat	1/295	10/12756	0.208688339	0.634729845	0.597982328	Soltu.DM.07G019630	1
390	GO:1990573	potassium ion import across plasma	1/295	10/12756	0.208688339	0.634729845	0.597982328	Soltu.DM.01G037640	1

391	GO:0006875	membrane cellular metal ion homeostasis	4/295	102/12756	0.21060122	0.634729845	0.597982328	Soltu.DM.05G021830/Soltu.DM.03G017590/Soltu.DM.07G002440/Soltu.DM.11G022440	4
392	GO:0009812	flavonoid metabolic process	4/295	102/12756	0.21060122	0.634729845	0.597982328	Soltu.DM.09G028560/Soltu.DM.09G028570/Soltu.DM.06G028410/Soltu.DM.04G001370	4
393	GO:0009962	regulation of flavonoid biosynthetic process	2/295	37/12756	0.210618622	0.634729845	0.597982328	Soltu.DM.05G026160/Soltu.DM.03G031830	2
394	GO:0031333	negative regulation of protein-containing complex assembly	2/295	37/12756	0.210618622	0.634729845	0.597982328	Soltu.DM.12G019570/Soltu.DM.01G028770	2
395	GO:0042908	xenobiotic transport	5/295	137/12756	0.211205046	0.634729845	0.597982328	Soltu.DM.05G026690/Soltu.DM.03G035710/Soltu.DM.05G003990/Soltu.DM.03G032350/Soltu.DM.11G003270	5
396	GO:0016132	brassinosteroid biosynthetic process	2/295	38/12756	0.219151669	0.634729845	0.597982328	Soltu.DM.06G004470/Soltu.DM.08G020150	2
397	GO:0048586	regulation of long-day photoperiodism, flowering	2/295	38/12756	0.219151669	0.634729845	0.597982328	Soltu.DM.12G007510/Soltu.DM.10G026020	2
398	GO:0090693	plant organ senescence	5/295	139/12756	0.219524239	0.634729845	0.597982328	Soltu.DM.10G010160/Soltu.DM.01G031830/Soltu.DM.12G027330/Soltu.DM.06G028410/Soltu.DM.05G026160	5
399	GO:0006694	steroid biosynthetic process	3/295	70/12756	0.22005189	0.634729845	0.597982328	Soltu.DM.06G004470/Soltu.DM.01G003630/Soltu.DM.08G020150	3
400	GO:1901926	cadinene metabolic process	3/295	70/12756	0.22005189	0.634729845	0.597982328	Soltu.DM.06G017120/Soltu.DM.06G017230/Soltu.DM.06G017100	3
401	GO:1901928	cadinene biosynthetic process	3/295	70/12756	0.22005189	0.634729845	0.597982328	Soltu.DM.06G017120/Soltu.DM.06G017230/Soltu.DM.06G017100	3
402	GO:0045338	farnesyl diphosphate metabolic process	4/295	104/12756	0.220340817	0.634729845	0.597982328	Soltu.DM.06G017120/Soltu.DM.06G017230/Soltu.DM.06G017100/Soltu.DM.01G040980	4

403	GO:0043934	sporulation	3/295	71/12756	0.226147889	0.634729845	0.597982328	Soltu.DM.06G009270/Soltu.DM.11G009620/Soltu.DM.08G022670	3
404	GO:0006098	pentose-phosphate shunt	1/295	11/12756	0.227002864	0.634729845	0.597982328	Soltu.DM.08G014620	1
405	GO:0006743	ubiquinone metabolic process	1/295	11/12756	0.227002864	0.634729845	0.597982328	Soltu.DM.06G032850	1
406	GO:0006744	ubiquinone biosynthetic process	1/295	11/12756	0.227002864	0.634729845	0.597982328	Soltu.DM.06G032850	1
407	GO:0006882	cellular zinc ion homeostasis	1/295	11/12756	0.227002864	0.634729845	0.597982328	Soltu.DM.07G002440	1
408	GO:0008216	spermidine metabolic process	1/295	11/12756	0.227002864	0.634729845	0.597982328	Soltu.DM.06G014480	1
409	GO:0008295	spermidine biosynthetic process	1/295	11/12756	0.227002864	0.634729845	0.597982328	Soltu.DM.06G014480	1
410	GO:0010067	procambium histogenesis	1/295	11/12756	0.227002864	0.634729845	0.597982328	Soltu.DM.04G019530	1
411	GO:0010184	cytokinin transport	1/295	11/12756	0.227002864	0.634729845	0.597982328	Soltu.DM.04G030440	1
412	GO:0010508	positive regulation of autophagy	1/295	11/12756	0.227002864	0.634729845	0.597982328	Soltu.DM.08G014180	1
413	GO:0015802	basic amino acid transport	1/295	11/12756	0.227002864	0.634729845	0.597982328	Soltu.DM.05G003990	1
414	GO:0015860	purine nucleoside transmembrane transport	1/295	11/12756	0.227002864	0.634729845	0.597982328	Soltu.DM.04G030440	1
415	GO:0030866	cortical actin cytoskeleton organization	1/295	11/12756	0.227002864	0.634729845	0.597982328	Soltu.DM.01G028770	1
416	GO:0031152	aggregation involved	1/295	11/12756	0.227002864	0.634729845	0.597982328	Soltu.DM.01G028770	1

		in sorocarp development							
417	GO:0033169	histone H3-K9 demethylation	1/295	11/12756	0.227002864	0.634729845	0.597982328	Soltu.DM.08G022190	1
418	GO:0046513	ceramide biosynthetic process	1/295	11/12756	0.227002864	0.634729845	0.597982328	Soltu.DM.08G014180	1
419	GO:0080144	cellular amino acid homeostasis	1/295	11/12756	0.227002864	0.634729845	0.597982328	Soltu.DM.11G011180	1
420	GO:0098630	aggregation of unicellular organisms	1/295	11/12756	0.227002864	0.634729845	0.597982328	Soltu.DM.01G028770	1
421	GO:0098743	cell aggregation	1/295	11/12756	0.227002864	0.634729845	0.597982328	Soltu.DM.01G028770	1
422	GO:1903826	L-arginine transmembrane transport	1/295	11/12756	0.227002864	0.634729845	0.597982328	Soltu.DM.05G003990	1
423	GO:1905156	negative regulation of photosynthesis	1/295	11/12756	0.227002864	0.634729845	0.597982328	Soltu.DM.07G000550	1
424	GO:1990822	basic amino acid transmembrane transport	1/295	11/12756	0.227002864	0.634729845	0.597982328	Soltu.DM.05G003990	1
425	GO:0010208	pollen wall assembly	2/295	39/12756	0.22771344	0.634729845	0.597982328	Soltu.DM.07G000410/Soltu.DM.05G025440	2
426	GO:0051494	negative regulation of cytoskeleton organization	2/295	39/12756	0.22771344	0.634729845	0.597982328	Soltu.DM.12G019570/Soltu.DM.01G028770	2
427	GO:0071367	cellular response to brassinosteroid stimulus	2/295	39/12756	0.22771344	0.634729845	0.597982328	Soltu.DM.04G029270/Soltu.DM.10G026020	2
428	GO:0085029	extracellular matrix	2/295	39/12756	0.22771344	0.634729845	0.597982328	Soltu.DM.07G000410/Soltu.DM.05G025440	2

		assembly							
429	GO:1902904	negative regulation of supramolecular fiber organization	2/295	39/12756	0.22771344	0.634729845	0.597982328	Soltu.DM.12G019570/Soltu.DM.01G028770	2
430	GO:0006873	cellular monoatomic ion homeostasis	5/295	141/12756	0.227942962	0.634729845	0.597982328	Soltu.DM.05G021830/Soltu.DM.03G017590/Soltu.DM.07G002440/Soltu.DM.11G022440/Soltu.DM.01G037640	5
431	GO:1903825	organic acid transmembrane transport	5/295	141/12756	0.227942962	0.634729845	0.597982328	Soltu.DM.03G003280/Soltu.DM.11G011180/Soltu.DM.05G003990/Soltu.DM.03G011160/Soltu.DM.01G008290	5
432	GO:1905039	carboxylic acid transmembrane transport	5/295	141/12756	0.227942962	0.634729845	0.597982328	Soltu.DM.03G003280/Soltu.DM.11G011180/Soltu.DM.05G003990/Soltu.DM.03G011160/Soltu.DM.01G008290	5
433	GO:0046165	alcohol biosynthetic process	3/295	72/12756	0.232276189	0.634729845	0.597982328	Soltu.DM.10G022710/Soltu.DM.06G033540/Soltu.DM.08G014180	3
434	GO:1901617	organic hydroxy compound biosynthetic process	6/295	179/12756	0.234391993	0.634729845	0.597982328	Soltu.DM.06G004470/Soltu.DM.01G003630/Soltu.DM.10G022710/Soltu.DM.06G033540/Soltu.DM.08G020150/Soltu.DM.08G014180	6
435	GO:0009225	nucleotide-sugar metabolic process	2/295	40/12756	0.236298065	0.634729845	0.597982328	Soltu.DM.11G021140/Soltu.DM.04G000320	2
436	GO:0009312	oligosaccharide biosynthetic process	2/295	40/12756	0.236298065	0.634729845	0.597982328	Soltu.DM.04G012960/Soltu.DM.07G001730	2
437	GO:0009556	microsporogenesis	2/295	40/12756	0.236298065	0.634729845	0.597982328	Soltu.DM.06G009270/Soltu.DM.11G009620	2
438	GO:0009668	plastid membrane organization	2/295	40/12756	0.236298065	0.634729845	0.597982328	Soltu.DM.08G001900/Soltu.DM.10G022710	2
439	GO:0030148	sphingolipid biosynthetic process	2/295	40/12756	0.236298065	0.634729845	0.597982328	Soltu.DM.04G008710/Soltu.DM.08G014180	2
440	GO:00	protein-co	2/295	40/12756	0.23629	0.634729	0.597982328	Soltu.DM.08G019530/Soltu.DM.07G	2

	32984	ntaining complex disassembly			8065	845		001240	
441	GO:0043288	apocarotenoid metabolic process	2/295	40/12756	0.236298065	0.634729845	0.597982328	Soltu.DM.07G013900/Soltu.DM.08G020150	2
442	GO:1902644	tertiary alcohol metabolic process	2/295	40/12756	0.236298065	0.634729845	0.597982328	Soltu.DM.07G013900/Soltu.DM.08G020150	2
443	GO:0009411	response to UV	5/295	143/12756	0.236455187	0.634729845	0.597982328	Soltu.DM.07G028550/Soltu.DM.06G012170/Soltu.DM.10G000640/Soltu.DM.02G024380/Soltu.DM.07G011660	5
444	GO:0045859	regulation of protein kinase activity	4/295	108/12756	0.240163063	0.634729845	0.597982328	Soltu.DM.07G017180/Soltu.DM.07G017190/Soltu.DM.07G017220/Soltu.DM.07G017210	4
445	GO:0044242	cellular lipid catabolic process	5/295	144/12756	0.240744472	0.634729845	0.597982328	Soltu.DM.07G022710/Soltu.DM.07G013900/Soltu.DM.07G022720/Soltu.DM.08G020150/Soltu.DM.09G018310	5
446	GO:0007015	actin filament organization	3/295	74/12756	0.244620454	0.634729845	0.597982328	Soltu.DM.07G026780/Soltu.DM.12G019570/Soltu.DM.01G028770	3
447	GO:0000305	response to oxygen radical	1/295	12/12756	0.244894912	0.634729845	0.597982328	Soltu.DM.06G012170	1
448	GO:0006740	NADPH regeneration	1/295	12/12756	0.244894912	0.634729845	0.597982328	Soltu.DM.08G014620	1
449	GO:0006783	heme biosynthetic process	1/295	12/12756	0.244894912	0.634729845	0.597982328	Soltu.DM.08G013640	1
450	GO:0009423	chorismate biosynthetic process	1/295	12/12756	0.244894912	0.634729845	0.597982328	Soltu.DM.04G018630	1
451	GO:0009700	indole phytoalexin biosynthetic process	1/295	12/12756	0.244894912	0.634729845	0.597982328	Soltu.DM.12G022190	1
452	GO:0009821	alkaloid biosynthetic process	1/295	12/12756	0.244894912	0.634729845	0.597982328	Soltu.DM.08G001740	1
453	GO:0010120	camalexin biosynthetic process	1/295	12/12756	0.244894912	0.634729845	0.597982328	Soltu.DM.12G022190	1

454	GO:0010207	photosystem II assembly	1/295	12/12756	0.244894912	0.634729845	0.597982328	Soltu.DM.06G021700	1
455	GO:0010342	endosperm cellularization	1/295	12/12756	0.244894912	0.634729845	0.597982328	Soltu.DM.01G036010	1
456	GO:0016139	glycoside catabolic process	1/295	12/12756	0.244894912	0.634729845	0.597982328	Soltu.DM.02G008550	1
457	GO:0031033	myosin filament organization	1/295	12/12756	0.244894912	0.634729845	0.597982328	Soltu.DM.01G028770	1
458	GO:0031154	culmination involved in sorocarp development	1/295	12/12756	0.244894912	0.634729845	0.597982328	Soltu.DM.01G028770	1
459	GO:0045815	transcription initiation-coupled chromatin remodeling	1/295	12/12756	0.244894912	0.634729845	0.597982328	Soltu.DM.08G022190	1
460	GO:0045828	positive regulation of isoprenoid metabolic process	1/295	12/12756	0.244894912	0.634729845	0.597982328	Soltu.DM.10G005360	1
461	GO:0046217	indole phytoalexin metabolic process	1/295	12/12756	0.244894912	0.634729845	0.597982328	Soltu.DM.12G022190	1
462	GO:0052317	camalexin metabolic process	1/295	12/12756	0.244894912	0.634729845	0.597982328	Soltu.DM.12G022190	1
463	GO:0015807	L-amino acid transport	2/295	41/12756	0.244899932	0.634729845	0.597982328	Soltu.DM.11G011180/Soltu.DM.05G003990	2
464	GO:1902475	L-alpha-amino acid transmembrane transport	2/295	41/12756	0.244899932	0.634729845	0.597982328	Soltu.DM.11G011180/Soltu.DM.05G003990	2
465	GO:0043549	regulation of kinase activity	4/295	110/12756	0.250226131	0.642838363	0.605621406	Soltu.DM.07G017180/Soltu.DM.07G017190/Soltu.DM.07G017220/Soltu.DM.07G017210	4
466	GO:0000272	polysaccharide catabolic	3/295	75/12756	0.25083188	0.642838363	0.605621406	Soltu.DM.09G027770/Soltu.DM.05G006330/Soltu.DM.04G037250	3

		process							
467	GO:0006081	cellular aldehyde metabolic process	2/295	42/12756	0.253513671	0.642838363	0.605621406	Soltu.DM.08G014620/Soltu.DM.01G019990	2
468	GO:0016129	phytosteroid biosynthetic process	2/295	42/12756	0.253513671	0.642838363	0.605621406	Soltu.DM.06G004470/Soltu.DM.08G020150	2
469	GO:0009123	nucleoside monophosphate metabolic process	4/295	111/12756	0.255291409	0.642838363	0.605621406	Soltu.DM.02G018700/Soltu.DM.12G004480/Soltu.DM.09G006670/Soltu.DM.05G011440	4
470	GO:0010087	phloem or xylem histogenesis	4/295	112/12756	0.260377583	0.642838363	0.605621406	Soltu.DM.03G014570/Soltu.DM.03G014580/Soltu.DM.03G014560/Soltu.DM.04G019530	4
471	GO:0048764	trichoblast maturation	4/295	112/12756	0.260377583	0.642838363	0.605621406	Soltu.DM.12G005490/Soltu.DM.09G026810/Soltu.DM.04G001370/Soltu.DM.08G023170	4
472	GO:0048765	root hair cell differentiation	4/295	112/12756	0.260377583	0.642838363	0.605621406	Soltu.DM.12G005490/Soltu.DM.09G026810/Soltu.DM.04G001370/Soltu.DM.08G023170	4
473	GO:0009765	photosynthesis, light harvesting	2/295	43/12756	0.262134153	0.642838363	0.605621406	Soltu.DM.07G024910/Soltu.DM.06G021700	2
474	GO:0048870	cell motility	2/295	43/12756	0.262134153	0.642838363	0.605621406	Soltu.DM.08G027150/Soltu.DM.01G028770	2
475	GO:0051674	localization of cell	2/295	43/12756	0.262134153	0.642838363	0.605621406	Soltu.DM.08G027150/Soltu.DM.01G028770	2
476	GO:0006108	malate metabolic process	1/295	13/12756	0.262374197	0.642838363	0.605621406	Soltu.DM.08G014620	1
477	GO:0006672	ceramide metabolic process	1/295	13/12756	0.262374197	0.642838363	0.605621406	Soltu.DM.08G014180	1
478	GO:0006821	chloride transport	1/295	13/12756	0.262374197	0.642838363	0.605621406	Soltu.DM.02G010790	1
479	GO:0009717	isoflavonoid biosynthetic process	1/295	13/12756	0.262374197	0.642838363	0.605621406	Soltu.DM.05G021610	1
480	GO:0009820	alkaloid metabolic process	1/295	13/12756	0.262374197	0.642838363	0.605621406	Soltu.DM.08G001740	1
481	GO:0009963	positive regulation of flavonoid biosynthe	1/295	13/12756	0.262374197	0.642838363	0.605621406	Soltu.DM.03G031830	1

		tic process							
482	GO:0016093	polyprenol metabolic process	1/295	13/12756	0.262374197	0.642838363	0.605621406	Soltu.DM.10G022710	1
483	GO:0033275	actin-myosin filament sliding	1/295	13/12756	0.262374197	0.642838363	0.605621406	Soltu.DM.01G028770	1
484	GO:0034755	iron ion transmembrane transport	1/295	13/12756	0.262374197	0.642838363	0.605621406	Soltu.DM.02G012210	1
485	GO:0042168	heme metabolic process	1/295	13/12756	0.262374197	0.642838363	0.605621406	Soltu.DM.08G013640	1
486	GO:0042559	pteridine-containing compound biosynthetic process	1/295	13/12756	0.262374197	0.642838363	0.605621406	Soltu.DM.06G033540	1
487	GO:0046287	isoflavonoid metabolic process	1/295	13/12756	0.262374197	0.642838363	0.605621406	Soltu.DM.05G021610	1
488	GO:0046417	chorismate metabolic process	1/295	13/12756	0.262374197	0.642838363	0.605621406	Soltu.DM.04G018630	1
489	GO:0080026	response to indolebutyric acid	1/295	13/12756	0.262374197	0.642838363	0.605621406	Soltu.DM.03G035710	1
490	GO:1902358	sulfate transmembrane transport	1/295	13/12756	0.262374197	0.642838363	0.605621406	Soltu.DM.09G020160	1
491	GO:0016042	lipid catabolic process	5/295	149/12756	0.262492332	0.642838363	0.605621406	Soltu.DM.07G022710/Soltu.DM.07G013900/Soltu.DM.07G022720/Soltu.DM.08G020150/Soltu.DM.09G018310	5
492	GO:0010054	trichoblast differentiation	4/295	113/12756	0.265483464	0.648839423	0.611275035	Soltu.DM.12G005490/Soltu.DM.09G026810/Soltu.DM.04G001370/Soltu.DM.08G023170	4
493	GO:0006066	alcohol metabolic process	5/295	150/12756	0.266896895	0.649647614	0.612036437	Soltu.DM.07G013900/Soltu.DM.10G022710/Soltu.DM.06G033540/Soltu.DM.08G020150/Soltu.DM.08G014180	5
494	GO:0009553	embryo sac development	5/295	150/12756	0.266896895	0.649647614	0.612036437	Soltu.DM.06G009270/Soltu.DM.06G026960/Soltu.DM.08G022670/Soltu.DM.08G006060/Soltu.DM.12G002630	5

495	GO:0048508	embryonic meristem development	2/295	44/12756	0.270756475	0.654961425	0.617042605	Soltu.DM.04G019530/Soltu.DM.07G020980	2
496	GO:0015931	nucleobase-containing compound transport	5/295	151/12756	0.271318028	0.654961425	0.617042605	Soltu.DM.12G004580/Soltu.DM.12G005490/Soltu.DM.12G007520/Soltu.DM.03G032350/Soltu.DM.04G030440	5
497	GO:0002252	immune effector process	4/295	115/12756	0.275749631	0.654961425	0.617042605	Soltu.DM.07G028550/Soltu.DM.10G000640/Soltu.DM.10G025390/Soltu.DM.02G025590	4
498	GO:0042430	indole-containing compound metabolic process	3/295	79/12756	0.275888269	0.654961425	0.617042605	Soltu.DM.03G035710/Soltu.DM.08G002280/Soltu.DM.12G022190	3
499	GO:0006730	one-carbon metabolic process	1/295	14/12756	0.279450208	0.654961425	0.617042605	Soltu.DM.12G002630	1
500	GO:0009864	induced systemic resistance, jasmonic acid mediated signaling pathway	1/295	14/12756	0.279450208	0.654961425	0.617042605	Soltu.DM.02G025590	1
501	GO:0010112	regulation of systemic acquired resistance	1/295	14/12756	0.279450208	0.654961425	0.617042605	Soltu.DM.10G027770	1
502	GO:0015693	magnesium ion transport	1/295	14/12756	0.279450208	0.654961425	0.617042605	Soltu.DM.12G002670	1
503	GO:0015866	ADP transport	1/295	14/12756	0.279450208	0.654961425	0.617042605	Soltu.DM.03G032350	1
504	GO:0042058	regulation of epidermal growth factor receptor signaling pathway	1/295	14/12756	0.279450208	0.654961425	0.617042605	Soltu.DM.08G027150	1
505	GO:0043090	amino acid import	1/295	14/12756	0.279450208	0.654961425	0.617042605	Soltu.DM.11G011180	1
506	GO:0048768	root hair cell tip growth	1/295	14/12756	0.279450208	0.654961425	0.617042605	Soltu.DM.09G026810	1
507	GO:00	detection	1/295	14/12756	0.27945	0.654961	0.617042605	Soltu.DM.01G028770	1

	50982	of mechanical stimulus			0208	425			
508	GO:0051156	glucose 6-phosphate metabolic process	1/295	14/12756	0.279450208	0.654961425	0.617042605	Soltu.DM.08G014620	1
509	GO:0051973	positive regulation of telomerase activity	1/295	14/12756	0.279450208	0.654961425	0.617042605	Soltu.DM.06G026960	1
510	GO:0052314	phytoalexin metabolic process	1/295	14/12756	0.279450208	0.654961425	0.617042605	Soltu.DM.12G022190	1
511	GO:0052315	phytoalexin biosynthetic process	1/295	14/12756	0.279450208	0.654961425	0.617042605	Soltu.DM.12G022190	1
512	GO:1901184	regulation of ERBB signaling pathway	1/295	14/12756	0.279450208	0.654961425	0.617042605	Soltu.DM.08G027150	1
513	GO:1901642	nucleoside transmembrane transport	1/295	14/12756	0.279450208	0.654961425	0.617042605	Soltu.DM.04G030440	1
514	GO:0007178	transmembrane receptor protein serine/threonine kinase signaling pathway	5/295	153/12756	0.280207005	0.655444748	0.617497947	Soltu.DM.01G031880/Soltu.DM.08G023130/Soltu.DM.12G001970/Soltu.DM.09G026810/Soltu.DM.07G020980	5
515	GO:0048532	anatomical structure arrangement	3/295	80/12756	0.282194417	0.655444748	0.617497947	Soltu.DM.10G025140/Soltu.DM.10G026020/Soltu.DM.08G022670	3
516	GO:0043200	response to amino acid	2/295	46/12756	0.287988135	0.655444748	0.617497947	Soltu.DM.02G024660/Soltu.DM.01G051900	2
517	GO:0046885	regulation of hormone biosynthetic process	2/295	46/12756	0.287988135	0.655444748	0.617497947	Soltu.DM.06G009270/Soltu.DM.10G005360	2
518	GO:0071805	potassium ion	3/295	81/12756	0.288513269	0.655444748	0.617497947	Soltu.DM.10G010160/Soltu.DM.01G031830/Soltu.DM.01G037640	3

		transmembrane transport							
519	GO:0045089	positive regulation of innate immune response	4/295	118/12756	0.29126745	0.655444748	0.617497947	Soltu.DM.07G028550/Soltu.DM.10G000640/Soltu.DM.01G024680/Soltu.DM.02G025590	4
520	GO:0002218	activation of innate immune response	3/295	82/12756	0.294842838	0.655444748	0.617497947	Soltu.DM.07G028550/Soltu.DM.10G000640/Soltu.DM.02G025590	3
521	GO:0048439	flower morphogenesis	3/295	82/12756	0.294842838	0.655444748	0.617497947	Soltu.DM.08G022190/Soltu.DM.10G028070/Soltu.DM.11G021100	3
522	GO:0006801	superoxide metabolic process	1/295	15/12756	0.296132219	0.655444748	0.617497947	Soltu.DM.06G012170	1
523	GO:0009554	megasporogenesis	1/295	15/12756	0.296132219	0.655444748	0.617497947	Soltu.DM.08G022670	1
524	GO:0009833	plant-type primary cell wall biogenesis	1/295	15/12756	0.296132219	0.655444748	0.617497947	Soltu.DM.04G027320	1
525	GO:0010161	red light signaling pathway	1/295	15/12756	0.296132219	0.655444748	0.617497947	Soltu.DM.10G005360	1
526	GO:0010232	vascular transport	1/295	15/12756	0.296132219	0.655444748	0.617497947	Soltu.DM.03G027330	1
527	GO:0010233	phloem transport	1/295	15/12756	0.296132219	0.655444748	0.617497947	Soltu.DM.03G027330	1
528	GO:0010929	positive regulation of auxin mediated signaling pathway	1/295	15/12756	0.296132219	0.655444748	0.617497947	Soltu.DM.10G026020	1
529	GO:0015858	nucleoside transport	1/295	15/12756	0.296132219	0.655444748	0.617497947	Soltu.DM.04G030440	1
530	GO:0019369	arachidonic acid metabolic process	1/295	15/12756	0.296132219	0.655444748	0.617497947	Soltu.DM.04G034690	1
531	GO:0019674	NAD metabolic process	1/295	15/12756	0.296132219	0.655444748	0.617497947	Soltu.DM.02G018700	1
532	GO:0019755	one-carbon compound transport	1/295	15/12756	0.296132219	0.655444748	0.617497947	Soltu.DM.06G018020	1
533	GO:0030835	negative regulation of actin	1/295	15/12756	0.296132219	0.655444748	0.617497947	Soltu.DM.12G019570	1

		filament depolymerization							
534	GO:0035067	negative regulation of histone acetylation	1/295	15/12756	0.296132219	0.655444748	0.617497947	Soltu.DM.08G022190	1
535	GO:0046520	sphingoid biosynthetic process	1/295	15/12756	0.296132219	0.655444748	0.617497947	Soltu.DM.08G014180	1
536	GO:0051693	actin filament capping	1/295	15/12756	0.296132219	0.655444748	0.617497947	Soltu.DM.12G019570	1
537	GO:1901984	negative regulation of protein acetylation	1/295	15/12756	0.296132219	0.655444748	0.617497947	Soltu.DM.08G022190	1
538	GO:1902806	regulation of cell cycle G1/S phase transition	1/295	15/12756	0.296132219	0.655444748	0.617497947	Soltu.DM.11G023760	1
539	GO:2000757	negative regulation of peptidyl-lysine acetylation	1/295	15/12756	0.296132219	0.655444748	0.617497947	Soltu.DM.08G022190	1
540	GO:0051761	sesquiterpene metabolic process	4/295	119/12756	0.296467094	0.655444748	0.617497947	Soltu.DM.06G017120/Soltu.DM.06G017230/Soltu.DM.06G017100/Soltu.DM.01G040980	4
541	GO:0051762	sesquiterpene biosynthetic process	4/295	119/12756	0.296467094	0.655444748	0.617497947	Soltu.DM.06G017120/Soltu.DM.06G017230/Soltu.DM.06G017100/Soltu.DM.01G040980	4
542	GO:0006413	translational initiation	2/295	47/12756	0.296588749	0.655444748	0.617497947	Soltu.DM.08G019530/Soltu.DM.12G027350	2
543	GO:0046890	regulation of lipid biosynthetic process	2/295	47/12756	0.296588749	0.655444748	0.617497947	Soltu.DM.10G005360/Soltu.DM.08G029860	2
544	GO:0080050	regulation of seed development	2/295	47/12756	0.296588749	0.655444748	0.617497947	Soltu.DM.05G021010/Soltu.DM.10G026020	2
545	GO:1901699	cellular response to nitrogen	6/295	196/12756	0.301062807	0.661817285	0.623501547	Soltu.DM.07G028550/Soltu.DM.03G035710/Soltu.DM.10G000640/Soltu.DM.01G051900/Soltu.DM.12G005490/Soltu.DM.02G012210	6

		compound							
546	GO:0006813	potassium ion transport	3/295	83/12756	0.30118117	0.661817285	0.623501547	Soltu.DM.10G010160/Soltu.DM.01G031830/Soltu.DM.01G037640	3
547	GO:0071369	cellular response to ethylene stimulus	3/295	83/12756	0.30118117	0.661817285	0.623501547	Soltu.DM.07G028550/Soltu.DM.10G000640/Soltu.DM.02G012210	3
548	GO:0033692	cellular polysaccharide biosynthetic process	4/295	120/12756	0.301678379	0.661817285	0.623501547	Soltu.DM.01G040570/Soltu.DM.04G027320/Soltu.DM.08G029290/Soltu.DM.09G018910	4
549	GO:0009965	leaf morphogenesis	5/295	158/12756	0.302667173	0.662774831	0.624403656	Soltu.DM.08G003320/Soltu.DM.12G023260/Soltu.DM.10G025140/Soltu.DM.10G028070/Soltu.DM.11G021100	5
550	GO:0007346	regulation of mitotic cell cycle	5/295	159/12756	0.307194062	0.668297949	0.629607015	Soltu.DM.07G017180/Soltu.DM.07G017190/Soltu.DM.11G023760/Soltu.DM.07G017220/Soltu.DM.07G017210	5
551	GO:0008272	sulfate transport	1/295	16/12756	0.312429291	0.668297949	0.629607015	Soltu.DM.09G020160	1
552	GO:0009268	response to pH	1/295	16/12756	0.312429291	0.668297949	0.629607015	Soltu.DM.06G002800	1
553	GO:0010332	response to gamma radiation	1/295	16/12756	0.312429291	0.668297949	0.629607015	Soltu.DM.10G029860	1
554	GO:0016241	regulation of macroautophagy	1/295	16/12756	0.312429291	0.668297949	0.629607015	Soltu.DM.08G014180	1
555	GO:0019319	hexose biosynthetic process	1/295	16/12756	0.312429291	0.668297949	0.629607015	Soltu.DM.09G018910	1
556	GO:0051224	negative regulation of protein transport	1/295	16/12756	0.312429291	0.668297949	0.629607015	Soltu.DM.08G027150	1
557	GO:0051782	negative regulation of cell division	1/295	16/12756	0.312429291	0.668297949	0.629607015	Soltu.DM.10G026020	1
558	GO:0070252	actin-mediated cell contraction	1/295	16/12756	0.312429291	0.668297949	0.629607015	Soltu.DM.01G028770	1
559	GO:0071475	cellular hyperosmotic salinity response	1/295	16/12756	0.312429291	0.668297949	0.629607015	Soltu.DM.08G002280	1
560	GO:00	cellular	1/295	16/12756	0.31242	0.668297	0.629607015	Soltu.DM.10G005360	1

	71491	response to red light			9291	949			
561	GO:0090333	regulation of stomatal closure	1/295	16/12756	0.312429291	0.668297949	0.629607015	Soltu.DM.01G037640	1
562	GO:1904950	negative regulation of establishment of protein localization	1/295	16/12756	0.312429291	0.668297949	0.629607015	Soltu.DM.08G027150	1
563	GO:0009867	jasmonic acid mediated signaling pathway	3/295	85/12756	0.313876526	0.670198989	0.631397994	Soltu.DM.03G032770/Soltu.DM.02G025590/Soltu.DM.07G024710	3
564	GO:0048469	cell maturation	4/295	123/12756	0.317371054	0.676456954	0.637293656	Soltu.DM.12G005490/Soltu.DM.09G026810/Soltu.DM.04G001370/Soltu.DM.08G023170	4
565	GO:0002253	activation of immune response	3/295	86/12756	0.320229848	0.677010881	0.637815514	Soltu.DM.07G028550/Soltu.DM.10G000640/Soltu.DM.02G025590	3
566	GO:0006714	sesquiterpenoid metabolic process	3/295	86/12756	0.320229848	0.677010881	0.637815514	Soltu.DM.07G013900/Soltu.DM.08G020150/Soltu.DM.01G040980	3
567	GO:0120254	olefinic compound metabolic process	3/295	86/12756	0.320229848	0.677010881	0.637815514	Soltu.DM.07G013900/Soltu.DM.04G034690/Soltu.DM.08G020150	3
568	GO:0007167	enzyme-linked receptor protein signaling pathway	5/295	162/12756	0.320831366	0.677010881	0.637815514	Soltu.DM.01G031880/Soltu.DM.08G023130/Soltu.DM.12G001970/Soltu.DM.09G026810/Soltu.DM.07G020980	5
569	GO:0009863	salicylic acid mediated signaling pathway	2/295	50/12756	0.322281565	0.677010881	0.637815514	Soltu.DM.01G021910/Soltu.DM.09G018910	2
570	GO:0030198	extracellular matrix organization	2/295	50/12756	0.322281565	0.677010881	0.637815514	Soltu.DM.07G000410/Soltu.DM.05G025440	2
571	GO:0043062	extracellular structure organization	2/295	50/12756	0.322281565	0.677010881	0.637815514	Soltu.DM.07G000410/Soltu.DM.05G025440	2
572	GO:19	carbohydr	2/295	50/12756	0.32228	0.677010	0.637815514	Soltu.DM.03G035710/Soltu.DM.02G	2

	01136	ate derivative catabolic process			1565	881		008550	
573	GO:0005978	glycogen biosynthetic process	1/295	17/12756	0.328350277	0.677010881	0.637815514	Soltu.DM.01G040570	1
574	GO:0006836	neurotransmitter transport	1/295	17/12756	0.328350277	0.677010881	0.637815514	Soltu.DM.05G003990	1
575	GO:0007349	cellularization	1/295	17/12756	0.328350277	0.677010881	0.637815514	Soltu.DM.01G036010	1
576	GO:0009638	phototropism	1/295	17/12756	0.328350277	0.677010881	0.637815514	Soltu.DM.10G012930	1
577	GO:0015867	ATP transport	1/295	17/12756	0.328350277	0.677010881	0.637815514	Soltu.DM.03G032350	1
578	GO:0030834	regulation of actin filament depolymerization	1/295	17/12756	0.328350277	0.677010881	0.637815514	Soltu.DM.12G019570	1
579	GO:0040020	regulation of meiotic nuclear division	1/295	17/12756	0.328350277	0.677010881	0.637815514	Soltu.DM.11G023760	1
580	GO:0042775	mitochondrial ATP synthesis coupled electron transport	1/295	17/12756	0.328350277	0.677010881	0.637815514	Soltu.DM.02G018700	1
581	GO:0046889	positive regulation of lipid biosynthetic process	1/295	17/12756	0.328350277	0.677010881	0.637815514	Soltu.DM.10G005360	1
582	GO:0051291	protein heterooligomerization	1/295	17/12756	0.328350277	0.677010881	0.637815514	Soltu.DM.03G012810	1
583	GO:1901880	negative regulation of protein depolymerization	1/295	17/12756	0.328350277	0.677010881	0.637815514	Soltu.DM.12G019570	1
584	GO:0045088	regulation of innate immune response	5/295	164/12756	0.329962959	0.67916904	0.639848727	Soltu.DM.07G028550/Soltu.DM.10G000640/Soltu.DM.01G024680/Soltu.DM.10G027770/Soltu.DM.02G025590	5
585	GO:0006665	sphingolipid metabolic process	2/295	51/12756	0.33079722	0.679720315	0.640368086	Soltu.DM.04G008710/Soltu.DM.08G014180	2
586	GO:0007568	aging	5/295	165/12756	0.334538657	0.685062097	0.645400607	Soltu.DM.10G010160/Soltu.DM.01G031830/Soltu.DM.12G027330/Soltu.	5

								DM.06G028410/Soltu.DM.05G026160	
587	GO:0048588	developmental cell growth	5/295	165/12756	0.334538657	0.685062097	0.645400607	Soltu.DM.10G024410/Soltu.DM.12G005490/Soltu.DM.09G005320/Soltu.DM.09G026810/Soltu.DM.08G023170	5
588	GO:0030833	regulation of actin filament polymerization	2/295	52/12756	0.339282874	0.688955913	0.649068992	Soltu.DM.12G019570/Soltu.DM.01G028770	2
589	GO:0051054	positive regulation of DNA metabolic process	2/295	52/12756	0.339282874	0.688955913	0.649068992	Soltu.DM.06G026960/Soltu.DM.07G015530	2
590	GO:0046246	terpene biosynthetic process	4/295	128/12756	0.343657285	0.688955913	0.649068992	Soltu.DM.06G017120/Soltu.DM.06G017230/Soltu.DM.06G017100/Soltu.DM.01G040980	4
591	GO:0006012	galactose metabolic process	1/295	18/12756	0.343903827	0.688955913	0.649068992	Soltu.DM.01G040570	1
592	GO:0009862	systemic acquired resistance, salicylic acid mediated signaling pathway	1/295	18/12756	0.343903827	0.688955913	0.649068992	Soltu.DM.01G021910	1
593	GO:0009934	regulation of meristem structural organization	1/295	18/12756	0.343903827	0.688955913	0.649068992	Soltu.DM.08G022670	1
594	GO:0009956	radial pattern formation	1/295	18/12756	0.343903827	0.688955913	0.649068992	Soltu.DM.08G022670	1
595	GO:0010222	stem vascular tissue pattern formation	1/295	18/12756	0.343903827	0.688955913	0.649068992	Soltu.DM.08G003320	1
596	GO:0010444	guard mother cell differentiation	1/295	18/12756	0.343903827	0.688955913	0.649068992	Soltu.DM.11G023760	1
597	GO:0031053	primary miRNA processing	1/295	18/12756	0.343903827	0.688955913	0.649068992	Soltu.DM.12G005490	1
598	GO:0042548	regulation of photosynt	1/295	18/12756	0.343903827	0.688955913	0.649068992	Soltu.DM.07G000550	1

		hesis, light reaction							
599	GO:0080113	regulation of seed growth	1/295	18/12756	0.343903827	0.688955913	0.649068992	Soltu.DM.10G026020	1
600	GO:1902074	response to salt	1/295	18/12756	0.343903827	0.688955913	0.649068992	Soltu.DM.04G024100	1
601	GO:0030036	actin cytoskeleton organization	3/295	90/12756	0.345639439	0.690864966	0.65086752	Soltu.DM.07G026780/Soltu.DM.12G019570/Soltu.DM.01G028770	3
602	GO:0010374	stomatal complex development	2/295	53/12756	0.347735366	0.690864966	0.65086752	Soltu.DM.05G021390/Soltu.DM.11G023760	2
603	GO:0010927	cellular component assembly involved in morphogenesis	2/295	53/12756	0.347735366	0.690864966	0.65086752	Soltu.DM.07G000410/Soltu.DM.05G025440	2
604	GO:0019318	hexose metabolic process	2/295	53/12756	0.347735366	0.690864966	0.65086752	Soltu.DM.01G040570/Soltu.DM.09G018910	2
605	GO:0019751	polyol metabolic process	2/295	53/12756	0.347735366	0.690864966	0.65086752	Soltu.DM.06G033540/Soltu.DM.08G014180	2
606	GO:0050778	positive regulation of immune response	4/295	129/12756	0.348924953	0.691682296	0.651637531	Soltu.DM.07G028550/Soltu.DM.10G000640/Soltu.DM.01G024680/Soltu.DM.02G025590	4
607	GO:0046148	pigment biosynthetic process	3/295	91/12756	0.351982463	0.691682296	0.651637531	Soltu.DM.06G028410/Soltu.DM.08G013640/Soltu.DM.04G001370	3
608	GO:0071236	cellular response to antibiotic	3/295	91/12756	0.351982463	0.691682296	0.651637531	Soltu.DM.01G021910/Soltu.DM.08G002280/Soltu.DM.09G018910	3
609	GO:0061136	regulation of proteasomal protein catabolic process	2/295	54/12756	0.356151697	0.691682296	0.651637531	Soltu.DM.08G027150/Soltu.DM.12G005510	2
610	GO:1903050	regulation of proteolysis involved in protein catabolic	2/295	54/12756	0.356151697	0.691682296	0.651637531	Soltu.DM.08G027150/Soltu.DM.12G005510	2

		process							
611	GO:0015849	organic acid transport	5/295	170/12756	0.357488603	0.691682296	0.651637531	Soltu.DM.03G003280/Soltu.DM.11G011180/Soltu.DM.05G003990/Soltu.DM.03G011160/Soltu.DM.01G008290	5
612	GO:0046942	carboxylic acid transport	5/295	170/12756	0.357488603	0.691682296	0.651637531	Soltu.DM.03G003280/Soltu.DM.11G011180/Soltu.DM.05G003990/Soltu.DM.03G011160/Soltu.DM.01G008290	5
613	GO:0006575	cellular modified amino acid metabolic process	3/295	92/12756	0.358318498	0.691682296	0.651637531	Soltu.DM.07G022460/Soltu.DM.07G022490/Soltu.DM.07G022510	3
614	GO:0002183	cytoplasmic translational initiation	1/295	19/12756	0.359098392	0.691682296	0.651637531	Soltu.DM.08G019530	1
615	GO:0016577	histone demethylation	1/295	19/12756	0.359098392	0.691682296	0.651637531	Soltu.DM.08G022190	1
616	GO:0019682	glyceraldehyde-3-phosphate metabolic process	1/295	19/12756	0.359098392	0.691682296	0.651637531	Soltu.DM.08G014620	1
617	GO:0030968	endoplasmic reticulum unfolded protein response	1/295	19/12756	0.359098392	0.691682296	0.651637531	Soltu.DM.08G019590	1
618	GO:0035725	sodium ion transmembrane transport	1/295	19/12756	0.359098392	0.691682296	0.651637531	Soltu.DM.01G037640	1
619	GO:0045010	actin nucleation	1/295	19/12756	0.359098392	0.691682296	0.651637531	Soltu.DM.12G019570	1
620	GO:0055069	zinc ion homeostasis	1/295	19/12756	0.359098392	0.691682296	0.651637531	Soltu.DM.07G002440	1
621	GO:0062013	positive regulation of small molecule metabolic process	1/295	19/12756	0.359098392	0.691682296	0.651637531	Soltu.DM.10G005360	1
622	GO:0070076	histone lysine demethylation	1/295	19/12756	0.359098392	0.691682296	0.651637531	Soltu.DM.08G022190	1
623	GO:0072488	ammonium	1/295	19/12756	0.359098392	0.691682296	0.651637531	Soltu.DM.06G018020	1

		transmembrane transport						
624	GO:1900378	positive regulation of secondary metabolite biosynthetic process	1/295	19/12756	0.359098392	0.691682296	0.651637531	Soltu.DM.12G022190	1
625	GO:0006090	pyruvate metabolic process	2/295	55/12756	0.364529022	0.692485612	0.65239434	Soltu.DM.08G014620/Soltu.DM.12G004480	2
626	GO:0032271	regulation of protein polymerization	2/295	55/12756	0.364529022	0.692485612	0.65239434	Soltu.DM.12G019570/Soltu.DM.01G028770	2
627	GO:0002684	positive regulation of immune system process	4/295	133/12756	0.369997276	0.692485612	0.65239434	Soltu.DM.07G028550/Soltu.DM.10G000640/Soltu.DM.01G024680/Soltu.DM.02G025590	4
628	GO:0046467	membrane lipid biosynthetic process	3/295	94/12756	0.370963442	0.692485612	0.65239434	Soltu.DM.04G008710/Soltu.DM.08G001900/Soltu.DM.08G014180	3
629	GO:0008064	regulation of actin polymerization or depolymerization	2/295	56/12756	0.372864646	0.692485612	0.65239434	Soltu.DM.12G019570/Soltu.DM.01G028770	2
630	GO:0009736	cytokinin-activated signaling pathway	2/295	56/12756	0.372864646	0.692485612	0.65239434	Soltu.DM.03G027640/Soltu.DM.10G027680	2
631	GO:0019722	calcium-mediated signaling	2/295	56/12756	0.372864646	0.692485612	0.65239434	Soltu.DM.01G051900/Soltu.DM.10G029310	2
632	GO:0030832	regulation of actin filament length	2/295	56/12756	0.372864646	0.692485612	0.65239434	Soltu.DM.12G019570/Soltu.DM.01G028770	2
633	GO:0048825	cotyledon development	2/295	56/12756	0.372864646	0.692485612	0.65239434	Soltu.DM.08G003320/Soltu.DM.06G019760	2
634	GO:0071470	cellular response to osmotic stress	2/295	56/12756	0.372864646	0.692485612	0.65239434	Soltu.DM.08G002280/Soltu.DM.02G020550	2
635	GO:0003018	vascular process in	1/295	20/12756	0.373942231	0.692485612	0.65239434	Soltu.DM.03G027330	1

636	GO:0006482	circulatory system protein demethylation	1/295	20/12756	0.373942231	0.692485612	0.65239434	Soltu.DM.08G022190	1
637	GO:0006690	icosanoid metabolic process	1/295	20/12756	0.373942231	0.692485612	0.65239434	Soltu.DM.04G034690	1
638	GO:0006739	NADP metabolic process	1/295	20/12756	0.373942231	0.692485612	0.65239434	Soltu.DM.08G014620	1
639	GO:0008214	protein dealkylation	1/295	20/12756	0.373942231	0.692485612	0.65239434	Soltu.DM.08G022190	1
640	GO:0009641	shade avoidance	1/295	20/12756	0.373942231	0.692485612	0.65239434	Soltu.DM.04G036140	1
641	GO:0010052	guard cell differentiation	1/295	20/12756	0.373942231	0.692485612	0.65239434	Soltu.DM.05G021390	1
642	GO:0030048	actin filament-based movement	1/295	20/12756	0.373942231	0.692485612	0.65239434	Soltu.DM.01G028770	1
643	GO:0043242	negative regulation of protein-containing complex disassembly	1/295	20/12756	0.373942231	0.692485612	0.65239434	Soltu.DM.12G019570	1
644	GO:0045834	positive regulation of lipid metabolic process	1/295	20/12756	0.373942231	0.692485612	0.65239434	Soltu.DM.10G005360	1
645	GO:0050826	response to freezing	1/295	20/12756	0.373942231	0.692485612	0.65239434	Soltu.DM.02G033270	1
646	GO:0051972	regulation of telomerase activity	1/295	20/12756	0.373942231	0.692485612	0.65239434	Soltu.DM.06G026960	1
647	GO:0098659	inorganic cation import across plasma membrane	1/295	20/12756	0.373942231	0.692485612	0.65239434	Soltu.DM.01G037640	1
648	GO:0099587	inorganic ion import across plasma membran	1/295	20/12756	0.373942231	0.692485612	0.65239434	Soltu.DM.01G037640	1

649	GO:1901879	e regulation of protein depolymerization	1/295	20/12756	0.373942231	0.692485612	0.65239434	Soltu.DM.12G019570	1
650	GO:0000271	polysaccharide biosynthetic process	4/295	135/12756	0.380521149	0.69952228	0.659023622	Soltu.DM.01G040570/Soltu.DM.04G027320/Soltu.DM.08G029290/Soltu.DM.09G018910	4
651	GO:0002833	positive regulation of response to biotic stimulus	4/295	135/12756	0.380521149	0.69952228	0.659023622	Soltu.DM.07G028550/Soltu.DM.10G000640/Soltu.DM.01G024680/Soltu.DM.02G025590	4
652	GO:0042214	terpene metabolic process	4/295	135/12756	0.380521149	0.69952228	0.659023622	Soltu.DM.06G017120/Soltu.DM.06G017230/Soltu.DM.06G017100/Soltu.DM.01G040980	4
653	GO:0032350	regulation of hormone metabolic process	2/295	57/12756	0.381156021	0.69952228	0.659023622	Soltu.DM.06G009270/Soltu.DM.10G005360	2
654	GO:0097164	ammonium ion metabolic process	2/295	57/12756	0.381156021	0.69952228	0.659023622	Soltu.DM.06G014480/Soltu.DM.08G014180	2
655	GO:0010600	regulation of auxin biosynthetic process	1/295	21/12756	0.388443412	0.69952228	0.659023622	Soltu.DM.06G009270	1
656	GO:0010959	regulation of metal ion transport	1/295	21/12756	0.388443412	0.69952228	0.659023622	Soltu.DM.02G020550	1
657	GO:0031057	negative regulation of histone modification	1/295	21/12756	0.388443412	0.69952228	0.659023622	Soltu.DM.08G022190	1
658	GO:0032886	regulation of microtubule-based process	1/295	21/12756	0.388443412	0.69952228	0.659023622	Soltu.DM.08G010920	1
659	GO:0035065	regulation of histone acetylation	1/295	21/12756	0.388443412	0.69952228	0.659023622	Soltu.DM.08G022190	1
660	GO:0042558	pteridine-containing compound metabolic	1/295	21/12756	0.388443412	0.69952228	0.659023622	Soltu.DM.06G033540	1

		process							
661	GO:0046519	sphingoid metabolic process	1/295	21/12756	0.388443412	0.69952228	0.659023622	Soltu.DM.08G014180	1
662	GO:0080024	indolebutyric acid metabolic process	1/295	21/12756	0.388443412	0.69952228	0.659023622	Soltu.DM.08G002280	1
663	GO:0080186	developmental vegetative growth	1/295	21/12756	0.388443412	0.69952228	0.659023622	Soltu.DM.02G025590	1
664	GO:1901983	regulation of protein acetylation	1/295	21/12756	0.388443412	0.69952228	0.659023622	Soltu.DM.08G022190	1
665	GO:1903828	negative regulation of protein localization	1/295	21/12756	0.388443412	0.69952228	0.659023622	Soltu.DM.08G027150	1
666	GO:2000035	regulation of stem cell division	1/295	21/12756	0.388443412	0.69952228	0.659023622	Soltu.DM.08G027110	1
667	GO:2000756	regulation of peptidyl-lysine acetylation	1/295	21/12756	0.388443412	0.69952228	0.659023622	Soltu.DM.08G022190	1
668	GO:0071446	cellular response to salicylic acid stimulus	2/295	58/12756	0.389400736	0.69952228	0.659023622	Soltu.DM.01G021910/Soltu.DM.09G018910	2
669	GO:0110053	regulation of actin filament organization	2/295	58/12756	0.389400736	0.69952228	0.659023622	Soltu.DM.12G019570/Soltu.DM.01G028770	2
670	GO:0016052	carbohydrate catabolic process	4/295	137/12756	0.391027079	0.701393864	0.660786851	Soltu.DM.09G027770/Soltu.DM.12G004480/Soltu.DM.05G006330/Soltu.DM.04G037250	4
671	GO:0071417	cellular response to organonitrogen compound	2/295	59/12756	0.397596515	0.712113161	0.670885557	Soltu.DM.03G035710/Soltu.DM.01G051900	2
672	GO:0032103	positive regulation of response to	4/295	139/12756	0.401508061	0.71258375	0.671328901	Soltu.DM.07G028550/Soltu.DM.10G000640/Soltu.DM.01G024680/Soltu.DM.02G025590	4

673	GO:0071395	external stimulus cellular response to jasmonic acid stimulus	3/295	99/12756	0.402349867	0.71258375	0.671328901	Soltu.DM.03G032770/Soltu.DM.02G025590/Soltu.DM.07G024710	3
674	GO:0006596	polyamine biosynthetic process	1/295	22/12756	0.402609819	0.71258375	0.671328901	Soltu.DM.06G014480	1
675	GO:0006814	sodium ion transport	1/295	22/12756	0.402609819	0.71258375	0.671328901	Soltu.DM.01G037640	1
676	GO:0006826	iron ion transport	1/295	22/12756	0.402609819	0.71258375	0.671328901	Soltu.DM.02G012210	1
677	GO:0010588	cotyledon vascular tissue pattern formation	1/295	22/12756	0.402609819	0.71258375	0.671328901	Soltu.DM.08G003320	1
678	GO:0030838	positive regulation of actin filament polymerization	1/295	22/12756	0.402609819	0.71258375	0.671328901	Soltu.DM.12G019570	1
679	GO:0048579	negative regulation of long-day photoperiodism, flowering	1/295	22/12756	0.402609819	0.71258375	0.671328901	Soltu.DM.10G026020	1
680	GO:0009743	response to carbohydrate	5/295	180/12756	0.403481624	0.713075035	0.671791743	Soltu.DM.03G035710/Soltu.DM.06G026960/Soltu.DM.08G027110/Soltu.DM.04G024100/Soltu.DM.05G007640	5
681	GO:0015748	organophosphate ester transport	2/295	60/12756	0.405741211	0.715145237	0.673742092	Soltu.DM.03G032350/Soltu.DM.01G008290	2
682	GO:0031349	positive regulation of defense response	4/295	141/12756	0.411957343	0.715145237	0.673742092	Soltu.DM.07G028550/Soltu.DM.10G000640/Soltu.DM.01G024680/Soltu.DM.02G025590	4
683	GO:0002237	response to molecule of bacterial origin	2/295	61/12756	0.413832802	0.715145237	0.673742092	Soltu.DM.07G028550/Soltu.DM.10G000640	2
684	GO:0009873	ethylene-activated	2/295	61/12756	0.413832802	0.715145237	0.673742092	Soltu.DM.07G028550/Soltu.DM.10G000640	2

		signaling pathway							
685	GO:0070592	cell wall polysaccharide biosynthetic process	2/295	61/12756	0.413832802	0.715145237	0.673742092	Soltu.DM.08G029290/Soltu.DM.09G018910	2
686	GO:1901420	negative regulation of response to alcohol	2/295	61/12756	0.413832802	0.715145237	0.673742092	Soltu.DM.04G005970/Soltu.DM.04G024100	2
687	GO:1905958	negative regulation of cellular response to alcohol	2/295	61/12756	0.413832802	0.715145237	0.673742092	Soltu.DM.04G005970/Soltu.DM.04G024100	2
688	GO:0010143	cutin biosynthetic process	1/295	23/12756	0.416449155	0.715145237	0.673742092	Soltu.DM.07G022050	1
689	GO:0010440	stomatal lineage progression	1/295	23/12756	0.416449155	0.715145237	0.673742092	Soltu.DM.11G023760	1
690	GO:0017001	antibiotic catabolic process	1/295	23/12756	0.416449155	0.715145237	0.673742092	Soltu.DM.06G028410	1
691	GO:0019747	regulation of isoprenoid metabolic process	1/295	23/12756	0.416449155	0.715145237	0.673742092	Soltu.DM.10G005360	1
692	GO:0030497	fatty acid elongation	1/295	23/12756	0.416449155	0.715145237	0.673742092	Soltu.DM.04G008710	1
693	GO:0042761	very long-chain fatty acid biosynthetic process	1/295	23/12756	0.416449155	0.715145237	0.673742092	Soltu.DM.10G026580	1
694	GO:0042773	ATP synthesis coupled electron transport	1/295	23/12756	0.416449155	0.715145237	0.673742092	Soltu.DM.02G018700	1
695	GO:0043650	dicarboxylic acid biosynthetic process	1/295	23/12756	0.416449155	0.715145237	0.673742092	Soltu.DM.04G018630	1
696	GO:1901661	quinone metabolic process	1/295	23/12756	0.416449155	0.715145237	0.673742092	Soltu.DM.06G032850	1

697	GO:1901663	quinone biosynthetic process	1/295	23/12756	0.416449155	0.715145237	0.673742092	Soltu.DM.06G032850	1
698	GO:1901679	nucleotide transmembrane transport	1/295	23/12756	0.416449155	0.715145237	0.673742092	Soltu.DM.03G032350	1
699	GO:0048443	stamen development	4/295	142/12756	0.417168055	0.715145237	0.673742092	Soltu.DM.03G024680/Soltu.DM.03G024660/Soltu.DM.12G008000/Soltu.DM.03G024670	4
700	GO:0048466	androecium development	4/295	142/12756	0.417168055	0.715145237	0.673742092	Soltu.DM.03G024680/Soltu.DM.03G024660/Soltu.DM.12G008000/Soltu.DM.03G024670	4
701	GO:0071398	cellular response to fatty acid	3/295	103/12756	0.427138577	0.727733053	0.68560114	Soltu.DM.03G032770/Soltu.DM.02G025590/Soltu.DM.07G024710	3
702	GO:0010928	regulation of auxin mediated signaling pathway	2/295	63/12756	0.42984918	0.727733053	0.68560114	Soltu.DM.10G026500/Soltu.DM.10G026020	2
703	GO:0006119	oxidative phosphorylation	1/295	24/12756	0.429968946	0.727733053	0.68560114	Soltu.DM.02G018700	1
704	GO:0010229	inflorescence development	1/295	24/12756	0.429968946	0.727733053	0.68560114	Soltu.DM.06G019760	1
705	GO:0032273	positive regulation of protein polymerization	1/295	24/12756	0.429968946	0.727733053	0.68560114	Soltu.DM.12G019570	1
706	GO:0032436	positive regulation of proteasomal ubiquitin-dependent protein catabolic process	1/295	24/12756	0.429968946	0.727733053	0.68560114	Soltu.DM.12G005510	1
707	GO:0042181	ketone biosynthetic process	1/295	24/12756	0.429968946	0.727733053	0.68560114	Soltu.DM.06G032850	1
708	GO:0042762	regulation of sulfur metabolic process	1/295	24/12756	0.429968946	0.727733053	0.68560114	Soltu.DM.12G022190	1
709	GO:0090354	regulation of auxin	1/295	24/12756	0.429968946	0.727733053	0.68560114	Soltu.DM.06G009270	1

		metabolic process							
710	GO:0001932	regulation of protein phosphorylation	4/295	145/12756	0.432735066	0.731276092	0.688939055	Soltu.DM.07G017180/Soltu.DM.07G017190/Soltu.DM.07G017220/Soltu.DM.07G017210	4
711	GO:0010383	cell wall polysaccharide metabolic process	3/295	104/12756	0.433281084	0.731276092	0.688939055	Soltu.DM.01G003570/Soltu.DM.08G029290/Soltu.DM.09G018910	3
712	GO:0019932	second-messenger-mediated signaling	2/295	64/12756	0.437770506	0.733910346	0.6914208	Soltu.DM.01G051900/Soltu.DM.10G029310	2
713	GO:0035967	cellular response to topologically incorrect protein	2/295	64/12756	0.437770506	0.733910346	0.6914208	Soltu.DM.08G019590/Soltu.DM.08G029860	2
714	GO:0044038	cell wall macromolecule biosynthetic process	2/295	64/12756	0.437770506	0.733910346	0.6914208	Soltu.DM.08G029290/Soltu.DM.09G018910	2
715	GO:0070589	cellular component macromolecule biosynthetic process	2/295	64/12756	0.437770506	0.733910346	0.6914208	Soltu.DM.08G029290/Soltu.DM.09G018910	2
716	GO:0031401	positive regulation of protein modification process	4/295	146/12756	0.43789984	0.733910346	0.6914208	Soltu.DM.07G017180/Soltu.DM.07G017190/Soltu.DM.07G017220/Soltu.DM.07G017210	4
717	GO:0009403	toxin biosynthetic process	1/295	25/12756	0.443176546	0.737603127	0.694899788	Soltu.DM.12G022190	1
718	GO:0010315	auxin export across the plasma membrane	1/295	25/12756	0.443176546	0.737603127	0.694899788	Soltu.DM.10G026500	1
719	GO:0043467	regulation of generation of precursor	1/295	25/12756	0.443176546	0.737603127	0.694899788	Soltu.DM.07G000550	1

		metabolites and energy							
720	GO:2000031	regulation of salicylic acid mediated signaling pathway	1/295	25/12756	0.443176546	0.737603127	0.694899788	Soltu.DM.10G026020	1
721	GO:2000060	positive regulation of ubiquitin-dependent protein catabolic process	1/295	25/12756	0.443176546	0.737603127	0.694899788	Soltu.DM.12G005510	1
722	GO:0006638	neutral lipid metabolic process	2/295	65/12756	0.445631798	0.738616239	0.695854247	Soltu.DM.12G002120/Soltu.DM.10G005470	2
723	GO:0006639	acylglycerol metabolic process	2/295	65/12756	0.445631798	0.738616239	0.695854247	Soltu.DM.12G002120/Soltu.DM.10G005470	2
724	GO:1902903	regulation of supramolecular fiber organization	2/295	65/12756	0.445631798	0.738616239	0.695854247	Soltu.DM.12G019570/Soltu.DM.01G028770	2
725	GO:0009860	pollen tube growth	3/295	107/12756	0.451559803	0.744619003	0.701509481	Soltu.DM.10G024410/Soltu.DM.09G005320/Soltu.DM.09G026810	3
726	GO:0030029	actin filament-based process	3/295	107/12756	0.451559803	0.744619003	0.701509481	Soltu.DM.07G026780/Soltu.DM.12G019570/Soltu.DM.01G028770	3
727	GO:0120251	hydrocarbon biosynthetic process	4/295	149/12756	0.453311329	0.744619003	0.701509481	Soltu.DM.06G017120/Soltu.DM.06G017230/Soltu.DM.06G017100/Soltu.DM.01G040980	4
728	GO:0000038	very long-chain fatty acid metabolic process	1/295	26/12756	0.456079139	0.744619003	0.701509481	Soltu.DM.10G026580	1
729	GO:0000741	karyogamy	1/295	26/12756	0.456079139	0.744619003	0.701509481	Soltu.DM.12G002630	1
730	GO:0002791	regulation of peptide secretion	1/295	26/12756	0.456079139	0.744619003	0.701509481	Soltu.DM.08G027150	1
731	GO:0009226	nucleotide-sugar biosynthe	1/295	26/12756	0.456079139	0.744619003	0.701509481	Soltu.DM.11G021140	1

		tic process							
732	GO:0009559	embryo sac central cell differentiation	1/295	26/12756	0.456079139	0.744619003	0.701509481	Soltu.DM.12G002630	1
733	GO:0010197	polar nucleus fusion	1/295	26/12756	0.456079139	0.744619003	0.701509481	Soltu.DM.12G002630	1
734	GO:0010951	negative regulation of endopeptidase activity	1/295	26/12756	0.456079139	0.744619003	0.701509481	Soltu.DM.04G003450	1
735	GO:0050708	regulation of protein secretion	1/295	26/12756	0.456079139	0.744619003	0.701509481	Soltu.DM.08G027150	1
736	GO:0006643	membrane lipid metabolic process	3/295	108/12756	0.45759971	0.745074155	0.701938283	Soltu.DM.04G008710/Soltu.DM.08G001900/Soltu.DM.08G014180	3
737	GO:0010639	negative regulation of organelle organization	3/295	108/12756	0.45759971	0.745074155	0.701938283	Soltu.DM.08G022190/Soltu.DM.12G019570/Soltu.DM.01G028770	3
738	GO:0015718	monocarboxylic acid transport	2/295	67/12756	0.46116852	0.747664084	0.704378268	Soltu.DM.05G003990/Soltu.DM.01G008290	2
739	GO:0050776	regulation of immune response	5/295	193/12756	0.462611039	0.747664084	0.704378268	Soltu.DM.07G028550/Soltu.DM.10G000640/Soltu.DM.01G024680/Soltu.DM.10G027770/Soltu.DM.02G025590	5
740	GO:0097237	cellular response to toxic substance	4/295	152/12756	0.468584199	0.747664084	0.704378268	Soltu.DM.06G012170/Soltu.DM.02G020130/Soltu.DM.08G002280/Soltu.DM.05G018810	4
741	GO:0006595	polyamine metabolic process	1/295	27/12756	0.468683747	0.747664084	0.704378268	Soltu.DM.06G014480	1
742	GO:0010584	pollen exine formation	1/295	27/12756	0.468683747	0.747664084	0.704378268	Soltu.DM.07G000410	1
743	GO:0015695	organic cation transport	1/295	27/12756	0.468683747	0.747664084	0.704378268	Soltu.DM.06G018020	1
744	GO:0016137	glycoside metabolic process	1/295	27/12756	0.468683747	0.747664084	0.704378268	Soltu.DM.02G008550	1
745	GO:0043244	regulation of protein-co	1/295	27/12756	0.468683747	0.747664084	0.704378268	Soltu.DM.12G019570	1

		ntaining complex disassembly							
746	GO:0044273	sulfur compound catabolic process	1/295	27/12756	0.468683747	0.747664084	0.704378268	Soltu.DM.03G035710	1
747	GO:0051592	response to calcium ion	1/295	27/12756	0.468683747	0.747664084	0.704378268	Soltu.DM.03G032350	1
748	GO:1905268	negative regulation of chromatin organization	1/295	27/12756	0.468683747	0.747664084	0.704378268	Soltu.DM.08G022190	1
749	GO:2000012	regulation of auxin polar transport	1/295	27/12756	0.468683747	0.747664084	0.704378268	Soltu.DM.07G022680	1
750	GO:0009850	auxin metabolic process	2/295	68/12756	0.468841317	0.747664084	0.704378268	Soltu.DM.06G019760/Soltu.DM.08G002280	2
751	GO:0071368	cellular response to cytokinin stimulus	2/295	68/12756	0.468841317	0.747664084	0.704378268	Soltu.DM.03G027640/Soltu.DM.10G027680	2
752	GO:0000281	mitotic cytokinesis	3/295	110/12756	0.469594273	0.747664084	0.704378268	Soltu.DM.04G027320/Soltu.DM.11G023760/Soltu.DM.01G028770	3
753	GO:0019216	regulation of lipid metabolic process	2/295	69/12756	0.476448802	0.747664084	0.704378268	Soltu.DM.10G005360/Soltu.DM.08G029860	2
754	GO:0042176	regulation of protein catabolic process	2/295	69/12756	0.476448802	0.747664084	0.704378268	Soltu.DM.08G027150/Soltu.DM.12G005510	2
755	GO:0051262	protein tetramerization	2/295	69/12756	0.476448802	0.747664084	0.704378268	Soltu.DM.03G012810/Soltu.DM.02G020550	2
756	GO:0044087	regulation of cellular component biogenesis	5/295	197/12756	0.480488132	0.747664084	0.704378268	Soltu.DM.07G028550/Soltu.DM.10G000640/Soltu.DM.08G029860/Soltu.DM.12G019570/Soltu.DM.01G028770	5
757	GO:0007135	meiosis II	1/295	28/12756	0.480997227	0.747664084	0.704378268	Soltu.DM.11G009620	1
758	GO:0009718	anthocyanin-containing compound	1/295	28/12756	0.480997227	0.747664084	0.704378268	Soltu.DM.04G001370	1

		biosynthetic process							
759	GO:0009750	response to fructose	1/295	28/12756	0.480997227	0.747664084	0.704378268	Soltu.DM.08G027110	1
760	GO:0010466	negative regulation of peptidase activity	1/295	28/12756	0.480997227	0.747664084	0.704378268	Soltu.DM.04G003450	1
761	GO:0010492	maintenance of shoot apical meristem identity	1/295	28/12756	0.480997227	0.747664084	0.704378268	Soltu.DM.02G027330	1
762	GO:0015868	purine ribonucleotide transport	1/295	28/12756	0.480997227	0.747664084	0.704378268	Soltu.DM.03G032350	1
763	GO:0016144	S-glycoside biosynthetic process	1/295	28/12756	0.480997227	0.747664084	0.704378268	Soltu.DM.05G007640	1
764	GO:0019758	glycosinolate biosynthetic process	1/295	28/12756	0.480997227	0.747664084	0.704378268	Soltu.DM.05G007640	1
765	GO:0019761	glucosinolate biosynthetic process	1/295	28/12756	0.480997227	0.747664084	0.704378268	Soltu.DM.05G007640	1
766	GO:0030641	regulation of cellular pH	1/295	28/12756	0.480997227	0.747664084	0.704378268	Soltu.DM.01G037640	1
767	GO:0033559	unsaturated fatty acid metabolic process	1/295	28/12756	0.480997227	0.747664084	0.704378268	Soltu.DM.04G034690	1
768	GO:0050821	protein stabilization	1/295	28/12756	0.480997227	0.747664084	0.704378268	Soltu.DM.04G037380	1
769	GO:0051453	regulation of intracellular pH	1/295	28/12756	0.480997227	0.747664084	0.704378268	Soltu.DM.01G037640	1
770	GO:0061983	meiosis II cell cycle process	1/295	28/12756	0.480997227	0.747664084	0.704378268	Soltu.DM.11G009620	1
771	GO:0070301	cellular response to	1/295	28/12756	0.480997227	0.747664084	0.704378268	Soltu.DM.08G002280	1

772	GO:1902905	hydrogen peroxide positive regulation of supramolecular fiber organization	1/295	28/12756	0.480997227	0.747664084	0.704378268	Soltu.DM.12G019570	1
773	GO:0030865	cortical cytoskeleton organization	2/295	70/12756	0.483989888	0.749900088	0.706484819	Soltu.DM.04G027320/Soltu.DM.01G028770	2
774	GO:0009746	response to hexose	2/295	71/12756	0.491463569	0.749900088	0.706484819	Soltu.DM.08G027110/Soltu.DM.04G024100	2
775	GO:0010083	regulation of vegetative meristem growth	2/295	71/12756	0.491463569	0.749900088	0.706484819	Soltu.DM.10G028070/Soltu.DM.11G021100	2
776	GO:0043648	dicarboxylic acid metabolic process	2/295	71/12756	0.491463569	0.749900088	0.706484819	Soltu.DM.08G014620/Soltu.DM.04G018630	2
777	GO:1905613	regulation of developmental vegetative growth	2/295	71/12756	0.491463569	0.749900088	0.706484819	Soltu.DM.10G028070/Soltu.DM.11G021100	2
778	GO:2000243	positive regulation of reproductive process	2/295	71/12756	0.491463569	0.749900088	0.706484819	Soltu.DM.12G007510/Soltu.DM.06G019760	2
779	GO:0006334	nucleosome assembly	1/295	29/12756	0.493026283	0.749900088	0.706484819	Soltu.DM.05G025150	1
780	GO:0009625	response to insect	1/295	29/12756	0.493026283	0.749900088	0.706484819	Soltu.DM.02G025590	1
781	GO:0009827	plant-type cell wall modification	1/295	29/12756	0.493026283	0.749900088	0.706484819	Soltu.DM.05G009050	1
782	GO:0009960	endosperm development	1/295	29/12756	0.493026283	0.749900088	0.706484819	Soltu.DM.01G036010	1
783	GO:0016477	cell migration	1/295	29/12756	0.493026283	0.749900088	0.706484819	Soltu.DM.08G027150	1
784	GO:0048826	cotyledon morphogenesis	1/295	29/12756	0.493026283	0.749900088	0.706484819	Soltu.DM.08G003320	1
785	GO:20	regulation	1/295	29/12756	0.49302	0.749900	0.706484819	Soltu.DM.10G026020	1

	01023	of response to drug			6283	088			
786	GO:2001038	regulation of cellular response to drug	1/295	29/12756	0.493026283	0.749900088	0.706484819	Soltu.DM.10G026020	1
787	GO:0042440	pigment metabolic process	3/295	114/12756	0.493217908	0.749900088	0.706484819	Soltu.DM.06G028410/Soltu.DM.08G013640/Soltu.DM.04G001370	3
788	GO:0061640	cytoskeleton-dependent cytokinesis	3/295	114/12756	0.493217908	0.749900088	0.706484819	Soltu.DM.04G027320/Soltu.DM.11G023760/Soltu.DM.01G028770	3
789	GO:0010941	regulation of cell death	4/295	157/12756	0.493684224	0.749900088	0.706484819	Soltu.DM.12G006650/Soltu.DM.03G032350/Soltu.DM.04G030790/Soltu.DM.08G014180	4
790	GO:0042325	regulation of phosphorylation	4/295	157/12756	0.493684224	0.749900088	0.706484819	Soltu.DM.07G017180/Soltu.DM.07G017190/Soltu.DM.07G017220/Soltu.DM.07G017210	4
791	GO:0009686	gibberellin biosynthetic process	2/295	72/12756	0.498868923	0.755862004	0.712101572	Soltu.DM.08G027110/Soltu.DM.06G004470	2
792	GO:0032956	regulation of actin cytoskeleton organization	2/295	72/12756	0.498868923	0.755862004	0.712101572	Soltu.DM.12G019570/Soltu.DM.01G028770	2
793	GO:0006863	purine nucleobase transport	1/295	30/12756	0.504777461	0.760016253	0.716015312	Soltu.DM.04G030440	1
794	GO:0022904	respiratory electron transport chain	1/295	30/12756	0.504777461	0.760016253	0.716015312	Soltu.DM.02G018700	1
795	GO:0032434	regulation of proteasomal ubiquitin-dependent protein catabolic process	1/295	30/12756	0.504777461	0.760016253	0.716015312	Soltu.DM.12G005510	1
796	GO:0048235	pollen sperm cell differentiation	1/295	30/12756	0.504777461	0.760016253	0.716015312	Soltu.DM.07G022050	1
797	GO:1904823	purine nucleobase	1/295	30/12756	0.504777461	0.760016253	0.716015312	Soltu.DM.04G030440	1

		transmembrane transport							
798	GO:0015979	photosynthesis	3/295	117/12756	0.510590829	0.762941616	0.718771312	Soltu.DM.07G024910/Soltu.DM.10G018880/Soltu.DM.06G021700	3
799	GO:0120252	hydrocarbon metabolic process	4/295	161/12756	0.513403317	0.762941616	0.718771312	Soltu.DM.06G017120/Soltu.DM.06G017230/Soltu.DM.06G017100/Soltu.DM.01G040980	4
800	GO:0009756	carbohydrate mediated signaling	2/295	74/12756	0.513471345	0.762941616	0.718771312	Soltu.DM.06G026960/Soltu.DM.08G027110	2
801	GO:0010182	sugar mediated signaling pathway	2/295	74/12756	0.513471345	0.762941616	0.718771312	Soltu.DM.06G026960/Soltu.DM.08G027110	2
802	GO:0034284	response to monosaccharide	2/295	74/12756	0.513471345	0.762941616	0.718771312	Soltu.DM.08G027110/Soltu.DM.04G024100	2
803	GO:0006779	porphyrin-containing compound biosynthetic process	1/295	31/12756	0.51625716	0.762941616	0.718771312	Soltu.DM.08G013640	1
804	GO:0006835	dicarboxylic acid transport	1/295	31/12756	0.51625716	0.762941616	0.718771312	Soltu.DM.05G003990	1
805	GO:0015851	nucleobase transport	1/295	31/12756	0.51625716	0.762941616	0.718771312	Soltu.DM.04G030440	1
806	GO:0015865	purine nucleotide transport	1/295	31/12756	0.51625716	0.762941616	0.718771312	Soltu.DM.03G032350	1
807	GO:0016126	sterol biosynthetic process	1/295	31/12756	0.51625716	0.762941616	0.718771312	Soltu.DM.01G003630	1
808	GO:0030244	cellulose biosynthetic process	1/295	31/12756	0.51625716	0.762941616	0.718771312	Soltu.DM.04G027320	1
809	GO:0051051	negative regulation of transport	1/295	31/12756	0.51625716	0.762941616	0.718771312	Soltu.DM.08G027150	1
810	GO:0051503	adenine nucleotide transport	1/295	31/12756	0.51625716	0.762941616	0.718771312	Soltu.DM.03G032350	1
811	GO:0052548	regulation of	1/295	31/12756	0.51625716	0.762941616	0.718771312	Soltu.DM.04G003450	1

812	GO:2000058	endopeptidase activity regulation of ubiquitin-dependent protein catabolic process	1/295	31/12756	0.51625716	0.762941616	0.718771312	Soltu.DM.12G005510	1
813	GO:0010224	response to UV-B	2/295	75/12756	0.520666946	0.76756798	0.723129834	Soltu.DM.02G024380/Soltu.DM.07G011660	2
814	GO:0032970	regulation of actin filament-based process	2/295	75/12756	0.520666946	0.76756798	0.723129834	Soltu.DM.12G019570/Soltu.DM.01G028770	2
815	GO:0000910	cytokinesis	3/295	119/12756	0.52199796	0.767644059	0.723201508	Soltu.DM.04G027320/Soltu.DM.11G023760/Soltu.DM.01G028770	3
816	GO:0098869	cellular oxidant detoxification	3/295	119/12756	0.52199796	0.767644059	0.723201508	Soltu.DM.06G012170/Soltu.DM.02G020130/Soltu.DM.05G018810	3
817	GO:0003013	circulatory system process	1/295	32/12756	0.527471631	0.768874091	0.724360328	Soltu.DM.03G027330	1
818	GO:0006096	glycolytic process	1/295	32/12756	0.527471631	0.768874091	0.724360328	Soltu.DM.12G004480	1
819	GO:0006757	ATP generation from ADP	1/295	32/12756	0.527471631	0.768874091	0.724360328	Soltu.DM.12G004480	1
820	GO:0010103	stomatal complex morphogenesis	1/295	32/12756	0.527471631	0.768874091	0.724360328	Soltu.DM.05G021390	1
821	GO:0045454	cell redox homeostasis	1/295	32/12756	0.527471631	0.768874091	0.724360328	Soltu.DM.01G047270	1
822	GO:0071230	cellular response to amino acid stimulus	1/295	32/12756	0.527471631	0.768874091	0.724360328	Soltu.DM.01G051900	1
823	GO:0009832	plant-type cell wall biogenesis	3/295	120/12756	0.52764718	0.768874091	0.724360328	Soltu.DM.04G027320/Soltu.DM.04G000320/Soltu.DM.08G029290	3
824	GO:0051259	protein complex oligomerization	4/295	164/12756	0.527960209	0.768874091	0.724360328	Soltu.DM.08G014620/Soltu.DM.03G012810/Soltu.DM.02G020550/Soltu.DM.12G026330	4
825	GO:0016102	diterpenoid biosynthetic process	2/295	77/12756	0.53484379	0.772521929	0.727796975	Soltu.DM.08G027110/Soltu.DM.06G004470	2

826	GO:0046034	ATP metabolic process	2/295	77/12756	0.53484379	0.772521929	0.727796975	Soltu.DM.02G018700/Soltu.DM.12G004480	2
827	GO:0001676	long-chain fatty acid metabolic process	1/295	33/12756	0.53842698	0.772521929	0.727796975	Soltu.DM.04G034690	1
828	GO:0006862	nucleotide transport	1/295	33/12756	0.53842698	0.772521929	0.727796975	Soltu.DM.03G032350	1
829	GO:0007140	male meiotic nuclear division	1/295	33/12756	0.53842698	0.772521929	0.727796975	Soltu.DM.11G009620	1
830	GO:0010091	trichome branching	1/295	33/12756	0.53842698	0.772521929	0.727796975	Soltu.DM.10G018290	1
831	GO:0031647	regulation of protein stability	1/295	33/12756	0.53842698	0.772521929	0.727796975	Soltu.DM.04G037380	1
832	GO:0033014	tetrapyrrole biosynthetic process	1/295	33/12756	0.53842698	0.772521929	0.727796975	Soltu.DM.08G013640	1
833	GO:0046031	ADP metabolic process	1/295	33/12756	0.53842698	0.772521929	0.727796975	Soltu.DM.12G004480	1
834	GO:0051274	beta-glucan biosynthetic process	1/295	33/12756	0.53842698	0.772521929	0.727796975	Soltu.DM.04G027320	1
835	GO:0051495	positive regulation of cytoskeleton organization	1/295	33/12756	0.53842698	0.772521929	0.727796975	Soltu.DM.12G019570	1
836	GO:2000070	regulation of response to water deprivation	1/295	33/12756	0.53842698	0.772521929	0.727796975	Soltu.DM.07G019630	1
837	GO:1990748	cellular detoxification	3/295	122/12756	0.538834046	0.772521929	0.727796975	Soltu.DM.06G012170/Soltu.DM.02G020130/Soltu.DM.05G018810	3
838	GO:0008283	cell population proliferation	2/295	78/12756	0.541823972	0.774956813	0.730090893	Soltu.DM.10G026020/Soltu.DM.08G027150	2
839	GO:0035966	response to topologically	2/295	78/12756	0.541823972	0.774956813	0.730090893	Soltu.DM.08G019590/Soltu.DM.08G029860	2

840	GO:0097435	incorrect protein supramolecular fiber organization	4/295	168/12756	0.54703579	0.777987027	0.732945672	Soltu.DM.04G027320/Soltu.DM.07G026780/Soltu.DM.12G019570/Soltu.DM.01G028770	4
841	GO:0008356	asymmetric cell division	1/295	34/12756	0.549129176	0.777987027	0.732945672	Soltu.DM.11G023760	1
842	GO:0009851	auxin biosynthetic process	1/295	34/12756	0.549129176	0.777987027	0.732945672	Soltu.DM.06G019760	1
843	GO:0030243	cellulose metabolic process	1/295	34/12756	0.549129176	0.777987027	0.732945672	Soltu.DM.04G027320	1
844	GO:0046835	carbohydrate phosphorylation	1/295	34/12756	0.549129176	0.777987027	0.732945672	Soltu.DM.12G004480	1
845	GO:0051289	protein homotetramerization	1/295	34/12756	0.549129176	0.777987027	0.732945672	Soltu.DM.03G012810	1
846	GO:0052547	regulation of peptidase activity	1/295	34/12756	0.549129176	0.777987027	0.732945672	Soltu.DM.04G003450	1
847	GO:2000069	regulation of post-embryonic root development	1/295	34/12756	0.549129176	0.777987027	0.732945672	Soltu.DM.08G027110	1
848	GO:0043067	regulation of programmed cell death	3/295	125/12756	0.555327578	0.785840912	0.740344859	Soltu.DM.12G006650/Soltu.DM.04G030790/Soltu.DM.08G014180	3
849	GO:0000165	MAPK cascade	1/295	35/12756	0.559584049	0.786300772	0.740778095	Soltu.DM.07G023200	1
850	GO:0019915	lipid storage	1/295	35/12756	0.559584049	0.786300772	0.740778095	Soltu.DM.02G033270	1
851	GO:0042866	pyruvate biosynthetic process	1/295	35/12756	0.559584049	0.786300772	0.740778095	Soltu.DM.12G004480	1
852	GO:0045861	negative regulation of proteolysis	1/295	35/12756	0.559584049	0.786300772	0.740778095	Soltu.DM.04G003450	1
853	GO:0051046	regulation of secretion	1/295	35/12756	0.559584049	0.786300772	0.740778095	Soltu.DM.08G027150	1

854	GO:1903530	regulation of secretion by cell	1/295	35/12756	0.559584049	0.786300772	0.740778095	Soltu.DM.08G027150	1
855	GO:0009205	purine ribonucleoside triphosphate metabolic process	2/295	81/12756	0.562326495	0.787388324	0.741802684	Soltu.DM.02G018700/Soltu.DM.12G004480	2
856	GO:0019827	stem cell population maintenance	2/295	81/12756	0.562326495	0.787388324	0.741802684	Soltu.DM.02G027330/Soltu.DM.03G027640	2
857	GO:0098727	maintenance of cell number	2/295	81/12756	0.562326495	0.787388324	0.741802684	Soltu.DM.02G027330/Soltu.DM.03G027640	2
858	GO:0009144	purine nucleoside triphosphate metabolic process	2/295	82/12756	0.569013586	0.789557453	0.743846232	Soltu.DM.02G018700/Soltu.DM.12G004480	2
859	GO:0048232	male gamete generation	2/295	82/12756	0.569013586	0.789557453	0.743846232	Soltu.DM.07G022050/Soltu.DM.11G009620	2
860	GO:0007584	response to nutrient	1/295	36/12756	0.569797295	0.789557453	0.743846232	Soltu.DM.02G025590	1
861	GO:0008360	regulation of cell shape	1/295	36/12756	0.569797295	0.789557453	0.743846232	Soltu.DM.01G028770	1
862	GO:0009135	purine nucleoside diphosphate metabolic process	1/295	36/12756	0.569797295	0.789557453	0.743846232	Soltu.DM.12G004480	1
863	GO:0009179	purine ribonucleoside diphosphate metabolic process	1/295	36/12756	0.569797295	0.789557453	0.743846232	Soltu.DM.12G004480	1
864	GO:0009185	ribonucleoside diphosphate metabolic process	1/295	36/12756	0.569797295	0.789557453	0.743846232	Soltu.DM.12G004480	1
865	GO:00	anthocya	1/295	36/12756	0.56979	0.789557	0.743846232	Soltu.DM.04G001370	1

	46283	nin-containing compound metabolic process			7295	453			
866	GO:0051273	beta-glucan metabolic process	1/295	36/12756	0.569797295	0.789557453	0.743846232	Soltu.DM.04G027320	1
867	GO:0009267	cellular response to starvation	4/295	174/12756	0.57488213	0.794767922	0.748755042	Soltu.DM.07G028550/Soltu.DM.10G000640/Soltu.DM.04G001370/Soltu.DM.01G028770	4
868	GO:0071695	anatomical structure maturation	4/295	174/12756	0.57488213	0.794767922	0.748755042	Soltu.DM.12G005490/Soltu.DM.09G026810/Soltu.DM.04G001370/Soltu.DM.08G023170	4
869	GO:0000413	protein peptidyl-prolyl isomerization	1/295	37/12756	0.579774481	0.796029035	0.749943144	Soltu.DM.03G034400	1
870	GO:0005977	glycogen metabolic process	1/295	37/12756	0.579774481	0.796029035	0.749943144	Soltu.DM.01G040570	1
871	GO:0006112	energy reserve metabolic process	1/295	37/12756	0.579774481	0.796029035	0.749943144	Soltu.DM.01G040570	1
872	GO:0009742	brassinosteroid mediated signaling pathway	1/295	37/12756	0.579774481	0.796029035	0.749943144	Soltu.DM.04G029270	1
873	GO:0010027	thylakoid membrane organization	1/295	37/12756	0.579774481	0.796029035	0.749943144	Soltu.DM.08G001900	1
874	GO:0098739	import across plasma membrane	1/295	37/12756	0.579774481	0.796029035	0.749943144	Soltu.DM.01G037640	1
875	GO:0009914	hormone transport	3/295	130/12756	0.582022045	0.798201661	0.751989986	Soltu.DM.10G026500/Soltu.DM.12G008000/Soltu.DM.04G030440	3
876	GO:0006163	purine nucleotide metabolic process	4/295	177/12756	0.588439858	0.805723522	0.75907637	Soltu.DM.02G018700/Soltu.DM.08G014620/Soltu.DM.12G004480/Soltu.DM.05G011440	4
877	GO:0010506	regulation of autophagy	1/295	38/12756	0.589521043	0.805723522	0.75907637	Soltu.DM.08G014180	1

878	GO:0120031	plasma membrane bounded cell projection assembly	1/295	38/12756	0.589521043	0.805723522	0.75907637	Soltu.DM.01G028770	1
879	GO:0009126	purine nucleoside monophosphate metabolic process	2/295	86/12756	0.59502106	0.811392355	0.764417008	Soltu.DM.02G018700/Soltu.DM.12G004480	2
880	GO:0009167	purine ribonucleoside monophosphate metabolic process	2/295	86/12756	0.59502106	0.811392355	0.764417008	Soltu.DM.02G018700/Soltu.DM.12G004480	2
881	GO:0000470	maturation of LSU-rRNA	1/295	39/12756	0.599042296	0.814100516	0.76696838	Soltu.DM.10G015180	1
882	GO:0006165	nucleoside diphosphate phosphorylation	1/295	39/12756	0.599042296	0.814100516	0.76696838	Soltu.DM.12G004480	1
883	GO:0042023	DNA endoreduplication	1/295	39/12756	0.599042296	0.814100516	0.76696838	Soltu.DM.11G023760	1
884	GO:0071322	cellular response to carbohydrate stimulus	2/295	87/12756	0.601337214	0.815655996	0.768433807	Soltu.DM.06G026960/Soltu.DM.08G027110	2
885	GO:0003002	regionalization	4/295	180/12756	0.601743352	0.815655996	0.768433807	Soltu.DM.08G003320/Soltu.DM.04G029270/Soltu.DM.12G005490/Soltu.DM.08G022670	4
886	GO:0009199	ribonucleoside triphosphate metabolic process	2/295	88/12756	0.607579037	0.815655996	0.768433807	Soltu.DM.02G018700/Soltu.DM.12G004480	2
887	GO:2000028	regulation of photoperiodism, flowering	2/295	88/12756	0.607579037	0.815655996	0.768433807	Soltu.DM.12G007510/Soltu.DM.10G026020	2
888	GO:2000242	negative regulation of reproduct	2/295	88/12756	0.607579037	0.815655996	0.768433807	Soltu.DM.11G022440/Soltu.DM.10G026020	2

		ive process							
889	GO:0009932	cell tip growth	3/295	135/12756	0.607684088	0.815655996	0.768433807	Soltu.DM.10G024410/Soltu.DM.09G005320/Soltu.DM.09G026810	3
890	GO:0009166	nucleotide catabolic process	1/295	40/12756	0.60834343	0.815655996	0.768433807	Soltu.DM.12G004480	1
891	GO:0009309	amine biosynthetic process	1/295	40/12756	0.60834343	0.815655996	0.768433807	Soltu.DM.06G014480	1
892	GO:0042401	biogenic amine biosynthetic process	1/295	40/12756	0.60834343	0.815655996	0.768433807	Soltu.DM.06G014480	1
893	GO:0044772	mitotic cell cycle phase transition	1/295	40/12756	0.60834343	0.815655996	0.768433807	Soltu.DM.11G023760	1
894	GO:1901800	positive regulation of proteasomal protein catabolic process	1/295	40/12756	0.60834343	0.815655996	0.768433807	Soltu.DM.12G005510	1
895	GO:1903052	positive regulation of proteolysis involved in protein catabolic process	1/295	40/12756	0.60834343	0.815655996	0.768433807	Soltu.DM.12G005510	1
896	GO:0019953	sexual reproduction	4/295	183/12756	0.61478591	0.820504342	0.773001459	Soltu.DM.06G009270/Soltu.DM.07G022050/Soltu.DM.11G009620/Soltu.DM.08G022670	4
897	GO:1903046	meiotic cell cycle process	4/295	183/12756	0.61478591	0.820504342	0.773001459	Soltu.DM.06G009270/Soltu.DM.12G023260/Soltu.DM.11G009620/Soltu.DM.08G022670	4
898	GO:0008284	positive regulation of cell population proliferation	1/295	41/12756	0.617429518	0.820504342	0.773001459	Soltu.DM.11G023760	1
899	GO:0010109	regulation of photosynthesis	1/295	41/12756	0.617429518	0.820504342	0.773001459	Soltu.DM.07G000550	1
900	GO:0019359	nicotinamide nucleotid	1/295	41/12756	0.617429518	0.820504342	0.773001459	Soltu.DM.12G004480	1

		e biosynthetic process							
901	GO:0035196	miRNA processing	1/295	41/12756	0.617429518	0.820504342	0.773001459	Soltu.DM.12G005490	1
902	GO:0042180	cellular ketone metabolic process	1/295	41/12756	0.617429518	0.820504342	0.773001459	Soltu.DM.06G032850	1
903	GO:0090329	regulation of DNA-templated DNA replication	1/295	41/12756	0.617429518	0.820504342	0.773001459	Soltu.DM.07G015530	1
904	GO:0019220	regulation of phosphate metabolic process	4/295	184/12756	0.619074423	0.820872164	0.773347986	Soltu.DM.07G017180/Soltu.DM.07G017190/Soltu.DM.07G017220/Soltu.DM.07G017210	4
905	GO:0051174	regulation of phosphorus metabolic process	4/295	184/12756	0.619074423	0.820872164	0.773347986	Soltu.DM.07G017180/Soltu.DM.07G017190/Soltu.DM.07G017220/Soltu.DM.07G017210	4
906	GO:0051493	regulation of cytoskeleton organization	2/295	90/12756	0.619839922	0.820980029	0.773449606	Soltu.DM.12G019570/Soltu.DM.01G028770	2
907	GO:0005996	monosaccharide metabolic process	2/295	91/12756	0.62585919	0.825897381	0.778082269	Soltu.DM.01G040570/Soltu.DM.09G018910	2
908	GO:0019363	pyridine nucleotide biosynthetic process	1/295	42/12756	0.626305514	0.825897381	0.778082269	Soltu.DM.12G004480	1
909	GO:0072530	purine-containing compound transmembrane transport	1/295	42/12756	0.626305514	0.825897381	0.778082269	Soltu.DM.04G030440	1
910	GO:1901292	nucleoside phosphate	1/295	42/12756	0.626305514	0.825897381	0.778082269	Soltu.DM.12G004480	1

		catabolic process							
911	GO:0042594	response to starvation	4/295	187/12756	0.631760278	0.830411223	0.782334784	Soltu.DM.07G028550/Soltu.DM.10G000640/Soltu.DM.04G001370/Soltu.DM.01G028770	4
912	GO:0009624	response to nematode	2/295	92/12756	0.631804539	0.830411223	0.782334784	Soltu.DM.05G026690/Soltu.DM.05G025440	2
913	GO:0051260	protein homooligomerization	2/295	92/12756	0.631804539	0.830411223	0.782334784	Soltu.DM.08G014620/Soltu.DM.03G012810	2
914	GO:0030031	cell projection assembly	1/295	43/12756	0.634976261	0.832755752	0.784543576	Soltu.DM.01G028770	1
915	GO:0031124	mRNA 3'-end processing	1/295	43/12756	0.634976261	0.832755752	0.784543576	Soltu.DM.12G005490	1
916	GO:0009161	ribonucleoside monophosphate metabolic process	2/295	93/12756	0.637676155	0.834472613	0.786161041	Soltu.DM.02G018700/Soltu.DM.12G004480	2
917	GO:0010380	regulation of chlorophyll biosynthetic process	2/295	93/12756	0.637676155	0.834472613	0.786161041	Soltu.DM.10G028070/Soltu.DM.11G021100	2
918	GO:0044770	cell cycle phase transition	1/295	44/12756	0.643446488	0.836870239	0.788419856	Soltu.DM.11G023760	1
919	GO:0071472	cellular response to salt stress	1/295	44/12756	0.643446488	0.836870239	0.788419856	Soltu.DM.08G002280	1
920	GO:0080027	response to herbivore	1/295	44/12756	0.643446488	0.836870239	0.788419856	Soltu.DM.01G040980	1
921	GO:0019684	photosynthesis, light reaction	2/295	94/12756	0.643474258	0.836870239	0.788419856	Soltu.DM.07G024910/Soltu.DM.06G021700	2
922	GO:1901463	regulation of tetrapyrrole biosynthetic process	2/295	94/12756	0.643474258	0.836870239	0.788419856	Soltu.DM.10G028070/Soltu.DM.11G021100	2
923	GO:0051129	negative regulation of cellular compone	3/295	143/12756	0.646519396	0.836870239	0.788419856	Soltu.DM.08G022190/Soltu.DM.12G019570/Soltu.DM.01G028770	3

		nt organization							
924	GO:0009141	nucleoside triphosphate metabolic process	2/295	95/12756	0.649199094	0.836870239	0.788419856	Soltu.DM.02G018700/Soltu.DM.12G004480	2
925	GO:0006275	regulation of DNA replication	1/295	45/12756	0.651720818	0.836870239	0.788419856	Soltu.DM.07G015530	1
926	GO:0006885	regulation of pH	1/295	45/12756	0.651720818	0.836870239	0.788419856	Soltu.DM.01G037640	1
927	GO:0009132	nucleoside diphosphate metabolic process	1/295	45/12756	0.651720818	0.836870239	0.788419856	Soltu.DM.12G004480	1
928	GO:0009911	positive regulation of flower development	1/295	45/12756	0.651720818	0.836870239	0.788419856	Soltu.DM.06G019760	1
929	GO:0055081	monoatomic anion homeostasis	1/295	45/12756	0.651720818	0.836870239	0.788419856	Soltu.DM.11G011180	1
930	GO:0072525	pyridine-containing compound biosynthetic process	1/295	45/12756	0.651720818	0.836870239	0.788419856	Soltu.DM.12G004480	1
931	GO:0090056	regulation of chlorophyll metabolic process	2/295	96/12756	0.65485094	0.836870239	0.788419856	Soltu.DM.10G028070/Soltu.DM.11G021100	2
932	GO:0051321	meiotic cell cycle	4/295	193/12756	0.656309405	0.836870239	0.788419856	Soltu.DM.06G009270/Soltu.DM.12G023260/Soltu.DM.11G009620/Soltu.DM.08G022670	4
933	GO:0006635	fatty acid beta-oxidation	1/295	46/12756	0.659803767	0.836870239	0.788419856	Soltu.DM.09G018310	1
934	GO:0009060	aerobic respiration	1/295	46/12756	0.659803767	0.836870239	0.788419856	Soltu.DM.02G018700	1
935	GO:0009581	detection of external stimulus	1/295	46/12756	0.659803767	0.836870239	0.788419856	Soltu.DM.01G028770	1
936	GO:00	detection	1/295	46/12756	0.65980	0.836870	0.788419856	Soltu.DM.01G028770	1

	09582	of abiotic stimulus			3767	239			
937	GO:0010105	negative regulation of ethylene-activated signaling pathway	1/295	46/12756	0.659803767	0.836870239	0.788419856	Soltu.DM.08G027110	1
938	GO:0043401	steroid hormone mediated signaling pathway	1/295	46/12756	0.659803767	0.836870239	0.788419856	Soltu.DM.04G029270	1
939	GO:0043484	regulation of RNA splicing	1/295	46/12756	0.659803767	0.836870239	0.788419856	Soltu.DM.12G005490	1
940	GO:0046364	monosaccharide biosynthetic process	1/295	46/12756	0.659803767	0.836870239	0.788419856	Soltu.DM.09G018910	1
941	GO:0046939	nucleotide phosphorylation	1/295	46/12756	0.659803767	0.836870239	0.788419856	Soltu.DM.12G004480	1
942	GO:0070298	negative regulation of phosphorelay signal transduction system	1/295	46/12756	0.659803767	0.836870239	0.788419856	Soltu.DM.08G027110	1
943	GO:0071383	cellular response to steroid hormone stimulus	1/295	46/12756	0.659803767	0.836870239	0.788419856	Soltu.DM.04G029270	1
944	GO:2000377	regulation of reactive oxygen species metabolic process	1/295	46/12756	0.659803767	0.836870239	0.788419856	Soltu.DM.05G026160	1
945	GO:0009165	nucleotide biosynthetic process	3/295	146/12756	0.660364339	0.836870239	0.788419856	Soltu.DM.12G004480/Soltu.DM.09G006670/Soltu.DM.05G011440	3
946	GO:1901293	nucleoside phosphate biosynthetic process	3/295	146/12756	0.660364339	0.836870239	0.788419856	Soltu.DM.12G004480/Soltu.DM.09G006670/Soltu.DM.05G011440	3

947	GO:0009615	response to virus	2/295	97/12756	0.660430097	0.836870239	0.788419856	Soltu.DM.10G025390/Soltu.DM.01G035490	2
948	GO:0000209	protein polyubiquitination	2/295	98/12756	0.665936892	0.839873894	0.791249616	Soltu.DM.09G006890/Soltu.DM.12G005510	2
949	GO:0032535	regulation of cellular component size	2/295	98/12756	0.665936892	0.839873894	0.791249616	Soltu.DM.12G019570/Soltu.DM.01G028770	2
950	GO:1901401	regulation of tetrapyrrole metabolic process	2/295	98/12756	0.665936892	0.839873894	0.791249616	Soltu.DM.10G028070/Soltu.DM.11G021100	2
951	GO:0009749	response to glucose	1/295	47/12756	0.667699746	0.839873894	0.791249616	Soltu.DM.04G024100	1
952	GO:0015850	organic hydroxy compound transport	1/295	47/12756	0.667699746	0.839873894	0.791249616	Soltu.DM.03G035710	1
953	GO:0048545	response to steroid hormone	1/295	47/12756	0.667699746	0.839873894	0.791249616	Soltu.DM.04G029270	1
954	GO:0080086	stamen filament development	1/295	47/12756	0.667699746	0.839873894	0.791249616	Soltu.DM.12G008000	1
955	GO:0015980	energy derivation by oxidation of organic compounds	2/295	99/12756	0.671371676	0.8425111	0.793734141	Soltu.DM.02G018700/Soltu.DM.01G040570	2
956	GO:0018105	peptidyl-serine phosphorylation	2/295	99/12756	0.671371676	0.8425111	0.793734141	Soltu.DM.06G002800/Soltu.DM.10G022340	2
957	GO:0044703	multi-organism reproductive process	4/295	197/12756	0.672055759	0.8425111	0.793734141	Soltu.DM.06G009270/Soltu.DM.07G022050/Soltu.DM.11G009620/Soltu.DM.08G022670	4
958	GO:0006109	regulation of carbohydrate metabolic process	1/295	48/12756	0.675413065	0.8425111	0.793734141	Soltu.DM.07G019030	1
959	GO:0035195	miRNA-mediated gene silencing	1/295	48/12756	0.675413065	0.8425111	0.793734141	Soltu.DM.12G005490	1
960	GO:0042435	indole-containing	1/295	48/12756	0.675413065	0.8425111	0.793734141	Soltu.DM.12G022190	1

		compound biosynthetic process							
961	GO:0048481	plant ovule development	1/295	48/12756	0.675413065	0.8425111	0.793734141	Soltu.DM.10G026020	1
962	GO:0070588	calcium ion transmembrane transport	1/295	48/12756	0.675413065	0.8425111	0.793734141	Soltu.DM.01G051900	1
963	GO:0018209	peptidyl-serine modification	2/295	100/12756	0.676734822	0.843283267	0.794461604	Soltu.DM.06G002800/Soltu.DM.10G022340	2
964	GO:0048868	pollen tube development	3/295	150/12756	0.678210717	0.844245706	0.795368323	Soltu.DM.10G024410/Soltu.DM.09G005320/Soltu.DM.09G026810	3
965	GO:0006754	ATP biosynthetic process	1/295	49/12756	0.682947936	0.846484738	0.797477727	Soltu.DM.12G004480	1
966	GO:0042752	regulation of circadian rhythm	1/295	49/12756	0.682947936	0.846484738	0.797477727	Soltu.DM.12G007510	1
967	GO:0045732	positive regulation of protein catabolic process	1/295	49/12756	0.682947936	0.846484738	0.797477727	Soltu.DM.12G005510	1
968	GO:0045862	positive regulation of proteolysis	1/295	49/12756	0.682947936	0.846484738	0.797477727	Soltu.DM.12G005510	1
969	GO:0021700	developmental maturation	4/295	200/12756	0.683536426	0.846484738	0.797477727	Soltu.DM.12G005490/Soltu.DM.09G026810/Soltu.DM.04G001370/Soltu.DM.08G023170	4
970	GO:0042127	regulation of cell population proliferation	2/295	102/12756	0.687247798	0.846970612	0.797935471	Soltu.DM.02G027330/Soltu.DM.11G023760	2
971	GO:0034728	nucleosome organization	1/295	50/12756	0.690308474	0.846970612	0.797935471	Soltu.DM.05G025150	1
972	GO:0035670	plant-type ovary developm	1/295	50/12756	0.690308474	0.846970612	0.797935471	Soltu.DM.10G026020	1

		ent modulation by symbiont of host process							
973	GO:0044003		1/295	50/12756	0.690308474	0.846970612	0.797935471	Soltu.DM.01G024680	1
974	GO:0006486	protein glycosylation	3/295	153/12756	0.691135652	0.846970612	0.797935471	Soltu.DM.10G028070/Soltu.DM.11G021100/Soltu.DM.04G000320	3
975	GO:0008652	amino acid biosynthetic process	3/295	153/12756	0.691135652	0.846970612	0.797935471	Soltu.DM.04G018630/Soltu.DM.09G006670/Soltu.DM.04G023360	3
976	GO:0043413	macromolecule glycosylation	3/295	153/12756	0.691135652	0.846970612	0.797935471	Soltu.DM.10G028070/Soltu.DM.11G021100/Soltu.DM.04G000320	3
977	GO:0001666	response to hypoxia	2/295	103/12756	0.692398475	0.846970612	0.797935471	Soltu.DM.04G005970/Soltu.DM.08G027110	2
978	GO:0009606	tropism	2/295	103/12756	0.692398475	0.846970612	0.797935471	Soltu.DM.09G006890/Soltu.DM.10G012930	2
979	GO:0040011	locomotion	2/295	103/12756	0.692398475	0.846970612	0.797935471	Soltu.DM.08G027150/Soltu.DM.01G028770	2
980	GO:0051302	regulation of cell division	2/295	103/12756	0.692398475	0.846970612	0.797935471	Soltu.DM.08G027110/Soltu.DM.10G026020	2
981	GO:0090066	regulation of anatomical structure size	2/295	103/12756	0.692398475	0.846970612	0.797935471	Soltu.DM.12G019570/Soltu.DM.01G028770	2
982	GO:0042546	cell wall biogenesis	3/295	154/12756	0.695356616	0.848882799	0.799736952	Soltu.DM.04G027320/Soltu.DM.04G000320/Soltu.DM.08G029290	3
983	GO:0009926	auxin polar transport	2/295	104/12756	0.697479208	0.848882799	0.799736952	Soltu.DM.10G026500/Soltu.DM.12G008000	2
984	GO:0009145	purine nucleoside triphosphate biosynthetic process	1/295	51/12756	0.6974987	0.848882799	0.799736952	Soltu.DM.12G004480	1
985	GO:0009206	purine ribonucleoside triphosphate biosynthetic process	1/295	51/12756	0.6974987	0.848882799	0.799736952	Soltu.DM.12G004480	1
986	GO:00	microgam	1/295	51/12756	0.69749	0.848882	0.799736952	Soltu.DM.07G022050	1

	55046	etogenesis			87	799			
987	GO:0006406	mRNA export from nucleus	1/295	52/12756	0.704522539	0.851386754	0.802095942	Soltu.DM.12G005490	1
988	GO:0006816	calcium ion transport	1/295	52/12756	0.704522539	0.851386754	0.802095942	Soltu.DM.01G051900	1
989	GO:0009247	glycolipid biosynthetic process	1/295	52/12756	0.704522539	0.851386754	0.802095942	Soltu.DM.08G001900	1
990	GO:0010104	regulation of ethylene-activated signaling pathway	1/295	52/12756	0.704522539	0.851386754	0.802095942	Soltu.DM.08G027110	1
991	GO:0046620	regulation of organ growth	1/295	52/12756	0.704522539	0.851386754	0.802095942	Soltu.DM.12G008000	1
992	GO:0051028	mRNA transport	1/295	52/12756	0.704522539	0.851386754	0.802095942	Soltu.DM.12G005490	1
993	GO:0070297	regulation of phosphorelay signal transduction system	1/295	52/12756	0.704522539	0.851386754	0.802095942	Soltu.DM.08G027110	1
994	GO:0007276	gamete generation	2/295	106/12756	0.707432729	0.852328589	0.80298325	Soltu.DM.07G022050/Soltu.DM.11G009620	2
995	GO:0044272	sulfur compound biosynthetic process	2/295	106/12756	0.707432729	0.852328589	0.80298325	Soltu.DM.12G022190/Soltu.DM.05G007640	2
996	GO:0072522	purine-containing compound biosynthetic process	2/295	106/12756	0.707432729	0.852328589	0.80298325	Soltu.DM.12G004480/Soltu.DM.02G025740	2
997	GO:0044786	cell cycle DNA replication	1/295	53/12756	0.711383831	0.85537134	0.805849841	Soltu.DM.11G023760	1
998	GO:2001251	negative regulation of chromosome organization	1/295	53/12756	0.711383831	0.85537134	0.805849841	Soltu.DM.08G022190	1

999	GO:0010075	regulation of meristem growth	2/295	108/12756	0.717112288	0.856564204	0.806973645	Soltu.DM.10G028070/Soltu.DM.11G021100	2
1000	GO:0022412	cellular process involved in reproduction in multicellular organism	2/295	108/12756	0.717112288	0.856564204	0.806973645	Soltu.DM.07G022050/Soltu.DM.11G009620	2
1001	GO:0045017	glycerolipid biosynthetic process	2/295	108/12756	0.717112288	0.856564204	0.806973645	Soltu.DM.12G002120/Soltu.DM.10G005470	2
1002	GO:0002229	defense response to oomycetes	1/295	54/12756	0.718086324	0.856564204	0.806973645	Soltu.DM.06G028410	1
1003	GO:0007031	peroxisome organization	1/295	54/12756	0.718086324	0.856564204	0.806973645	Soltu.DM.09G018310	1
1004	GO:0010017	red or far-red light signaling pathway	1/295	54/12756	0.718086324	0.856564204	0.806973645	Soltu.DM.10G005360	1
1005	GO:0018208	peptidyl-proline modification	1/295	54/12756	0.718086324	0.856564204	0.806973645	Soltu.DM.03G034400	1
1006	GO:0043622	cortical microtubule organization	1/295	54/12756	0.718086324	0.856564204	0.806973645	Soltu.DM.04G027320	1
1007	GO:0006778	porphyrin-containing compound metabolic process	1/295	55/12756	0.724633682	0.859249425	0.809503405	Soltu.DM.08G013640	1
1008	GO:0010311	lateral root formation	1/295	55/12756	0.724633682	0.859249425	0.809503405	Soltu.DM.02G006700	1
1009	GO:0019395	fatty acid oxidation	1/295	55/12756	0.724633682	0.859249425	0.809503405	Soltu.DM.09G018310	1
1010	GO:0035821	modulation of process of another	1/295	55/12756	0.724633682	0.859249425	0.809503405	Soltu.DM.01G024680	1

1011	GO:0051346	organism negative regulation of hydrolase activity	1/295	55/12756	0.724633682	0.859249425	0.809503405	Soltu.DM.04G003450	1
1012	GO:0071489	cellular response to red or far red light	1/295	55/12756	0.724633682	0.859249425	0.809503405	Soltu.DM.10G005360	1
1013	GO:0090626	plant epidermis morphogenesis	2/295	110/12756	0.726522034	0.860638145	0.810811726	Soltu.DM.05G021390/Soltu.DM.10G018290	2
1014	GO:0009062	fatty acid catabolic process	1/295	56/12756	0.731029482	0.863535919	0.813541734	Soltu.DM.09G018310	1
1015	GO:0033013	tetrapyrrole metabolic process	1/295	56/12756	0.731029482	0.863535919	0.813541734	Soltu.DM.08G013640	1
1016	GO:0022604	regulation of cell morphogenesis	2/295	111/12756	0.731127078	0.863535919	0.813541734	Soltu.DM.02G030780/Soltu.DM.01G028770	2
1017	GO:0006935	chemotaxis	1/295	57/12756	0.737277223	0.866535423	0.816367583	Soltu.DM.01G028770	1
1018	GO:0009127	purine nucleoside monophosphate biosynthetic process	1/295	57/12756	0.737277223	0.866535423	0.816367583	Soltu.DM.12G004480	1
1019	GO:0009168	purine ribonucleoside monophosphate biosynthetic process	1/295	57/12756	0.737277223	0.866535423	0.816367583	Soltu.DM.12G004480	1
1020	GO:0031056	regulation of histone modification	1/295	57/12756	0.737277223	0.866535423	0.816367583	Soltu.DM.08G022190	1
1021	GO:0043269	regulation of monoatomic ion transport	1/295	57/12756	0.737277223	0.866535423	0.816367583	Soltu.DM.02G020550	1
1022	GO:0009201	ribonucleoside triphosphate	1/295	58/12756	0.743380319	0.872000374	0.821516142	Soltu.DM.12G004480	1

		biosynthetic process							
1023	GO:0042330	taxis	1/295	58/12756	0.743380319	0.872000374	0.821516142	Soltu.DM.01G028770	1
1024	GO:0031331	positive regulation of cellular catabolic process	2/295	115/12756	0.748894883	0.872860266	0.82232625	Soltu.DM.12G005510/Soltu.DM.08G014180	2
1025	GO:0000911	cytokinesis by cell plate formation	1/295	59/12756	0.749342109	0.872860266	0.82232625	Soltu.DM.11G023760	1
1026	GO:0009910	negative regulation of flower development	1/295	59/12756	0.749342109	0.872860266	0.82232625	Soltu.DM.11G022440	1
1027	GO:0010074	maintenance of meristem identity	1/295	59/12756	0.749342109	0.872860266	0.82232625	Soltu.DM.02G027330	1
1028	GO:0042178	xenobiotic catabolic process	1/295	59/12756	0.749342109	0.872860266	0.82232625	Soltu.DM.06G028410	1
1029	GO:0051783	regulation of nuclear division	1/295	59/12756	0.749342109	0.872860266	0.82232625	Soltu.DM.11G023760	1
1030	GO:1901659	glycosyl compound biosynthetic process	1/295	59/12756	0.749342109	0.872860266	0.82232625	Soltu.DM.05G007640	1
1031	GO:0009101	glycoprotein biosynthetic process	3/295	168/12756	0.749932445	0.872860266	0.82232625	Soltu.DM.10G028070/Soltu.DM.11G021100/Soltu.DM.04G000320	3
1032	GO:0006261	DNA-templated DNA replication	2/295	116/12756	0.75317665	0.874709482	0.824068407	Soltu.DM.11G023760/Soltu.DM.07G015530	2
1033	GO:0034976	response to endoplasmic reticulum stress	2/295	116/12756	0.75317665	0.874709482	0.824068407	Soltu.DM.08G019590/Soltu.DM.03G035420	2
1034	GO:0006664	glycolipid metabolic process	1/295	60/12756	0.755165853	0.874709482	0.824068407	Soltu.DM.08G001900	1
1035	GO:1902410	mitotic cytokinetic process	1/295	60/12756	0.755165853	0.874709482	0.824068407	Soltu.DM.11G023760	1

1036	GO:1903509	liposaccharide metabolic process	1/295	60/12756	0.755165853	0.874709482	0.824068407	Soltu.DM.08G001900	1
1037	GO:0006576	biogenic amine metabolic process	1/295	61/12756	0.760854737	0.877869904	0.827045857	Soltu.DM.06G014480	1
1038	GO:0010212	response to ionizing radiation	1/295	61/12756	0.760854737	0.877869904	0.827045857	Soltu.DM.10G029860	1
1039	GO:0045333	cellular respiration	1/295	61/12756	0.760854737	0.877869904	0.827045857	Soltu.DM.02G018700	1
1040	GO:0051510	regulation of unidimensional cell growth	1/295	61/12756	0.760854737	0.877869904	0.827045857	Soltu.DM.02G030780	1
1041	GO:0060918	auxin transport	2/295	118/12756	0.761552142	0.877869904	0.827045857	Soltu.DM.10G026500/Soltu.DM.12G008000	2
1042	GO:0000226	microtubule cytoskeleton organization	3/295	172/12756	0.764013183	0.879018044	0.828127525	Soltu.DM.04G027320/Soltu.DM.01G036010/Soltu.DM.11G023760	3
1043	GO:0019693	ribose phosphate metabolic process	3/295	172/12756	0.764013183	0.879018044	0.828127525	Soltu.DM.02G018700/Soltu.DM.08G014620/Soltu.DM.12G004480	3
1044	GO:0009845	seed germination	2/295	119/12756	0.765647085	0.879248803	0.828344925	Soltu.DM.10G005360/Soltu.DM.02G033270	2
1045	GO:0009142	nucleoside triphosphate biosynthetic process	1/295	62/12756	0.766411874	0.879248803	0.828344925	Soltu.DM.12G004480	1
1046	GO:0048598	embryonic morphogenesis	1/295	62/12756	0.766411874	0.879248803	0.828344925	Soltu.DM.08G003320	1
1047	GO:0010026	trichome differentiation	2/295	120/12756	0.769680982	0.881263906	0.830243364	Soltu.DM.07G003440/Soltu.DM.10G018290	2
1048	GO:0009561	megagametogenesis	1/295	63/12756	0.771840304	0.881263906	0.830243364	Soltu.DM.12G002630	1
1049	GO:0010565	regulation of cellular ketone metabolic process	1/295	63/12756	0.771840304	0.881263906	0.830243364	Soltu.DM.10G005360	1

1050	GO:0031122	cytoplasmic microtubule organization	1/295	63/12756	0.771840304	0.881263906	0.830243364	Soltu.DM.04G027320	1
1051	GO:0031334	positive regulation of protein-containing complex assembly	1/295	63/12756	0.771840304	0.881263906	0.830243364	Soltu.DM.12G019570	1
1052	GO:0036293	response to decreased oxygen levels	2/295	121/12756	0.773654454	0.882219493	0.831143627	Soltu.DM.04G005970/Soltu.DM.08G027110	2
1053	GO:0070085	glycosylation	3/295	175/12756	0.774147605	0.882219493	0.831143627	Soltu.DM.10G028070/Soltu.DM.11G021100/Soltu.DM.04G000320	3
1054	GO:0009156	ribonucleoside monophosphate biosynthetic process	1/295	64/12756	0.777142999	0.884792789	0.833567943	Soltu.DM.12G004480	1
1055	GO:0009896	positive regulation of catabolic process	2/295	123/12756	0.781422631	0.885648523	0.834374135	Soltu.DM.12G005510/Soltu.DM.08G014180	2
1056	GO:0045926	negative regulation of growth	2/295	123/12756	0.781422631	0.885648523	0.834374135	Soltu.DM.10G028070/Soltu.DM.11G021100	2
1057	GO:0070482	response to oxygen levels	2/295	123/12756	0.781422631	0.885648523	0.834374135	Soltu.DM.04G005970/Soltu.DM.08G027110	2
1058	GO:0016106	sesquiterpenoid biosynthetic process	1/295	65/12756	0.782322862	0.885648523	0.834374135	Soltu.DM.01G040980	1
1059	GO:0072593	reactive oxygen species metabolic process	1/295	65/12756	0.782322862	0.885648523	0.834374135	Soltu.DM.06G012170	1
1060	GO:0120036	plasma membrane bounded cell projection organization	1/295	65/12756	0.782322862	0.885648523	0.834374135	Soltu.DM.01G028770	1
1061	GO:00	glycoprot	3/295	179/1275	0.78710	0.888025	0.836613622	Soltu.DM.10G028070/Soltu.DM.11G	3

	09100	ein metabolic process		6	4487	633		021100/Soltu.DM.04G000320	
1062	GO:0002181	cytoplasmic translation	1/295	66/12756	0.787382728	0.888025633	0.836613622	Soltu.DM.08G019530	1
1063	GO:0009933	meristem structural organization	1/295	66/12756	0.787382728	0.888025633	0.836613622	Soltu.DM.08G022670	1
1064	GO:0034440	lipid oxidation	1/295	66/12756	0.787382728	0.888025633	0.836613622	Soltu.DM.09G018310	1
1065	GO:0031400	negative regulation of protein modification process	1/295	67/12756	0.792325367	0.89192349	0.840285814	Soltu.DM.08G022190	1
1066	GO:0032506	cytokinetic process	1/295	67/12756	0.792325367	0.89192349	0.840285814	Soltu.DM.11G023760	1
1067	GO:0009124	nucleoside monophosphate biosynthetic process	1/295	68/12756	0.797153487	0.892335993	0.840674436	Soltu.DM.12G004480	1
1068	GO:0009640	photomorphogenesis	1/295	68/12756	0.797153487	0.892335993	0.840674436	Soltu.DM.04G036140	1
1069	GO:0010101	post-embryonic root morphogenesis	1/295	68/12756	0.797153487	0.892335993	0.840674436	Soltu.DM.02G006700	1
1070	GO:0010102	lateral root morphogenesis	1/295	68/12756	0.797153487	0.892335993	0.840674436	Soltu.DM.02G006700	1
1071	GO:0034404	nucleobase-containing small molecule biosynthetic process	1/295	68/12756	0.797153487	0.892335993	0.840674436	Soltu.DM.12G004480	1
1072	GO:1902532	negative regulation of intracellular signal transduction	1/295	68/12756	0.797153487	0.892335993	0.840674436	Soltu.DM.08G027110	1
1073	GO:0001558	regulation of cell growth	2/295	128/12756	0.799829825	0.892833293	0.841142944	Soltu.DM.10G029860/Soltu.DM.02G030780	2

1074	GO:0043254	regulation of protein-containing complex assembly	2/295	128/12756	0.799829825	0.892833293	0.841142944	Soltu.DM.12G019570/Soltu.DM.01G028770	2
1075	GO:0090351	seedling development	2/295	128/12756	0.799829825	0.892833293	0.841142944	Soltu.DM.10G005360/Soltu.DM.02G033270	2
1076	GO:0051235	maintenance of location	1/295	69/12756	0.801869733	0.894278512	0.842504493	Soltu.DM.02G033270	1
1077	GO:0022900	electron transport chain	1/295	70/12756	0.806476687	0.894428858	0.842646135	Soltu.DM.02G018700	1
1078	GO:0030030	cell projection organization	1/295	70/12756	0.806476687	0.894428858	0.842646135	Soltu.DM.01G028770	1
1079	GO:0048506	regulation of timing of meristematic phase transition	1/295	70/12756	0.806476687	0.894428858	0.842646135	Soltu.DM.08G027110	1
1080	GO:0048510	regulation of timing of transition from vegetative to reproductive phase	1/295	70/12756	0.806476687	0.894428858	0.842646135	Soltu.DM.08G027110	1
1081	GO:0051607	defense response to virus	1/295	70/12756	0.806476687	0.894428858	0.842646135	Soltu.DM.10G025390	1
1082	GO:0140546	defense response to symbiont	1/295	70/12756	0.806476687	0.894428858	0.842646135	Soltu.DM.10G025390	1
1083	GO:0031329	regulation of cellular catabolic process	3/295	186/12756	0.808298434	0.895621533	0.84376976	Soltu.DM.08G027150/Soltu.DM.12G005510/Soltu.DM.08G014180	3
1084	GO:0006470	protein dephosphorylation	1/295	71/12756	0.810976874	0.896932949	0.845005252	Soltu.DM.02G015080	1
1085	GO:0031123	RNA 3'-end processing	1/295	71/12756	0.810976874	0.896932949	0.845005252	Soltu.DM.12G005490	1
1086	GO:0003008	system process	1/295	72/12756	0.815372761	0.900964377	0.848803281	Soltu.DM.03G027330	1
1087	GO:0046434	organophosphate catabolic	1/295	73/12756	0.819666756	0.904139501	0.851794583	Soltu.DM.12G004480	1

		process							
1088	GO:0006260	DNA replication	2/295	134/12756	0.820099096	0.904139501	0.851794583	Soltu.DM.11G023760/Soltu.DM.07G015530	2
1089	GO:0043086	negative regulation of catalytic activity	2/295	135/12756	0.823293306	0.904139501	0.851794583	Soltu.DM.06G012620/Soltu.DM.04G003450	2
1090	GO:0140013	meiotic nuclear division	2/295	135/12756	0.823293306	0.904139501	0.851794583	Soltu.DM.12G023260/Soltu.DM.11G009620	2
1091	GO:0051223	regulation of protein transport	1/295	74/12756	0.823861214	0.904139501	0.851794583	Soltu.DM.08G027150	1
1092	GO:0071166	ribonucleoprotein complex localization	1/295	74/12756	0.823861214	0.904139501	0.851794583	Soltu.DM.12G005490	1
1093	GO:0090087	regulation of peptide transport	1/295	74/12756	0.823861214	0.904139501	0.851794583	Soltu.DM.08G027150	1
1094	GO:0016114	terpenoid biosynthetic process	3/295	192/12756	0.825027295	0.904139501	0.851794583	Soltu.DM.08G027110/Soltu.DM.06G004470/Soltu.DM.01G040980	3
1095	GO:0022603	regulation of anatomical structure morphogenesis	3/295	192/12756	0.825027295	0.904139501	0.851794583	Soltu.DM.08G022670/Soltu.DM.02G030780/Soltu.DM.01G028770	3
1096	GO:0031348	negative regulation of defense response	1/295	75/12756	0.827958434	0.906523833	0.854040874	Soltu.DM.03G035710	1
1097	GO:0006611	protein export from nucleus	1/295	76/12756	0.831960662	0.908419286	0.85582659	Soltu.DM.12G005490	1
1098	GO:0051701	biological process involved in interaction with host	1/295	76/12756	0.831960662	0.908419286	0.85582659	Soltu.DM.01G024680	1
1099	GO:0070201	regulation of establishment of protein localization	1/295	76/12756	0.831960662	0.908419286	0.85582659	Soltu.DM.08G027150	1

1100	GO:0048284	organelle fusion	1/295	77/12756	0.835870095	0.909346738	0.856700348	Soltu.DM.12G002630	1
1101	GO:0009968	negative regulation of signal transduction	3/295	197/12756	0.838003481	0.909346738	0.856700348	Soltu.DM.04G005970/Soltu.DM.08G027110/Soltu.DM.04G024100	3
1102	GO:0140352	export from cell	2/295	140/12756	0.838513962	0.909346738	0.856700348	Soltu.DM.11G011180/Soltu.DM.10G026500	2
1103	GO:0006405	RNA export from nucleus	1/295	78/12756	0.839688876	0.909346738	0.856700348	Soltu.DM.12G005490	1
1104	GO:0016036	cellular response to phosphate starvation	1/295	78/12756	0.839688876	0.909346738	0.856700348	Soltu.DM.04G001370	1
1105	GO:1905393	plant organ formation	2/295	141/12756	0.841412425	0.909346738	0.856700348	Soltu.DM.02G006700/Soltu.DM.07G020980	2
1106	GO:0009856	pollination	3/295	199/12756	0.842957718	0.909346738	0.856700348	Soltu.DM.10G024410/Soltu.DM.09G005320/Soltu.DM.09G026810	3
1107	GO:0044706	multi-multicellular organism process	3/295	199/12756	0.842957718	0.909346738	0.856700348	Soltu.DM.10G024410/Soltu.DM.09G005320/Soltu.DM.09G026810	3
1108	GO:0007018	microtubule-based movement	1/295	79/12756	0.8434191	0.909346738	0.856700348	Soltu.DM.12G012260	1
1109	GO:0007131	reciprocal meiotic recombination	1/295	79/12756	0.8434191	0.909346738	0.856700348	Soltu.DM.12G023260	1
1110	GO:0050657	nucleic acid transport	1/295	79/12756	0.8434191	0.909346738	0.856700348	Soltu.DM.12G005490	1
1111	GO:0050658	RNA transport	1/295	79/12756	0.8434191	0.909346738	0.856700348	Soltu.DM.12G005490	1
1112	GO:0051236	establishment of RNA localization	1/295	79/12756	0.8434191	0.909346738	0.856700348	Soltu.DM.12G005490	1
1113	GO:0140527	reciprocal homologous recombination	1/295	79/12756	0.8434191	0.909346738	0.856700348	Soltu.DM.12G023260	1
1114	GO:0009150	purine ribonucleotide metabolic process	2/295	142/12756	0.844263789	0.909440347	0.856788538	Soltu.DM.02G018700/Soltu.DM.12G004480	2
1115	GO:00	nucleus	1/295	81/12756	0.85062	0.913011	0.860152573	Soltu.DM.12G002630	1

	06997	organization			2018	111			
1116	GO:0010090	trichome morphogenesis	1/295	81/12756	0.850622018	0.913011111	0.860152573	Soltu.DM.10G018290	1
1117	GO:0031503	protein-containing complex localization	1/295	81/12756	0.850622018	0.913011111	0.860152573	Soltu.DM.12G005490	1
1118	GO:0034249	negative regulation of amide metabolic process	1/295	81/12756	0.850622018	0.913011111	0.860152573	Soltu.DM.08G029860	1
1119	GO:0051168	nuclear export	1/295	82/12756	0.854098665	0.915923502	0.862896352	Soltu.DM.12G005490	1
1120	GO:0035825	homologous recombination	1/295	83/12756	0.857494665	0.916289936	0.863241571	Soltu.DM.12G023260	1
1121	GO:0048573	photoperiodism, flowering	1/295	83/12756	0.857494665	0.916289936	0.863241571	Soltu.DM.02G025590	1
1122	GO:0051606	detection of stimulus	1/295	83/12756	0.857494665	0.916289936	0.863241571	Soltu.DM.01G028770	1
1123	GO:2000280	regulation of root development	1/295	83/12756	0.857494665	0.916289936	0.863241571	Soltu.DM.08G027110	1
1124	GO:0048581	negative regulation of post-embryonic development	2/295	148/12756	0.860417331	0.918199339	0.86504043	Soltu.DM.11G022440/Soltu.DM.10G026020	2
1125	GO:1902275	regulation of chromatin organization	1/295	84/12756	0.86081188	0.918199339	0.86504043	Soltu.DM.08G022190	1
1126	GO:0009152	purine ribonucleotide biosynthetic process	1/295	86/12756	0.867217206	0.923390103	0.869930676	Soltu.DM.12G004480	1
1127	GO:0040034	regulation of development, heterochronic	1/295	86/12756	0.867217206	0.923390103	0.869930676	Soltu.DM.08G027110	1
1128	GO:0009744	response to sucrose	1/295	87/12756	0.870308833	0.92504039	0.87148542	Soltu.DM.08G027110	1

1129	GO:0034285	response to disaccharide	1/295	87/12756	0.870308833	0.92504039	0.87148542	Soltu.DM.08G027110	1
1130	GO:0006403	RNA localization	1/295	88/12756	0.873328716	0.926608717	0.872962949	Soltu.DM.12G005490	1
1131	GO:0009630	gravitropism	1/295	88/12756	0.873328716	0.926608717	0.872962949	Soltu.DM.09G006890	1
1132	GO:0044089	positive regulation of cellular component biogenesis	1/295	89/12756	0.876278513	0.928917152	0.875137738	Soltu.DM.12G019570	1
1133	GO:0044092	negative regulation of molecular function	2/295	155/12756	0.877322177	0.929131084	0.875339284	Soltu.DM.06G012620/Soltu.DM.04G003450	2
1134	GO:0010256	endomembrane system organization	1/295	90/12756	0.879159846	0.929131084	0.875339284	Soltu.DM.09G018910	1
1135	GO:1901987	regulation of cell cycle phase transition	1/295	90/12756	0.879159846	0.929131084	0.875339284	Soltu.DM.11G023760	1
1136	GO:0051052	regulation of DNA metabolic process	2/295	156/12756	0.879577426	0.929131084	0.875339284	Soltu.DM.06G026960/Soltu.DM.07G015530	2
1137	GO:0009648	photoperiodism	1/295	91/12756	0.881974297	0.930025621	0.876182032	Soltu.DM.02G025590	1
1138	GO:0065004	protein-DNA complex assembly	1/295	91/12756	0.881974297	0.930025621	0.876182032	Soltu.DM.05G025150	1
1139	GO:0071359	cellular response to dsRNA	1/295	93/12756	0.887408712	0.934688931	0.880575361	Soltu.DM.12G005490	1
1140	GO:0032880	regulation of protein localization	1/295	94/12756	0.890031663	0.934688931	0.880575361	Soltu.DM.08G027150	1
1141	GO:0070918	regulatory ncRNA processing	1/295	94/12756	0.890031663	0.934688931	0.880575361	Soltu.DM.12G005490	1
1142	GO:0009259	ribonucleotide metabolic process	2/295	161/12756	0.890291207	0.934688931	0.880575361	Soltu.DM.02G018700/Soltu.DM.12G004480	2
1143	GO:00	nucleobas	2/295	161/1275	0.89029	0.934688	0.880575361	Soltu.DM.12G004480/Soltu.DM.05G	2

	34655	e-containing compound catabolic process		6	1207	931		003030	
1144	GO:0007033	vacuole organization	1/295	95/12756	0.892593712	0.93628711	0.882081014	Soltu.DM.01G028770	1
1145	GO:0009629	response to gravity	1/295	96/12756	0.895096267	0.937273578	0.883010371	Soltu.DM.09G006890	1
1146	GO:0043331	response to dsRNA	1/295	96/12756	0.895096267	0.937273578	0.883010371	Soltu.DM.12G005490	1
1147	GO:0006338	chromatin remodeling	2/295	164/12756	0.896288998	0.93770427	0.883416128	Soltu.DM.08G022190/Soltu.DM.05G025150	2
1148	GO:0006164	purine nucleotide biosynthetic process	1/295	97/12756	0.897540706	0.93819586	0.883879258	Soltu.DM.12G004480	1
1149	GO:0007127	meiosis I	1/295	98/12756	0.899928375	0.939039638	0.884674185	Soltu.DM.12G023260	1
1150	GO:0062012	regulation of small molecule metabolic process	1/295	98/12756	0.899928375	0.939039638	0.884674185	Soltu.DM.10G005360	1
1151	GO:0006839	mitochondrial transport	1/295	99/12756	0.902260586	0.939039638	0.884674185	Soltu.DM.03G032350	1
1152	GO:0035194	RNA-mediated post-transcriptional gene silencing	1/295	99/12756	0.902260586	0.939039638	0.884674185	Soltu.DM.12G005490	1
1153	GO:0042273	ribosomal large subunit biogenesis	1/295	99/12756	0.902260586	0.939039638	0.884674185	Soltu.DM.10G015180	1
1154	GO:0010638	positive regulation of organelle organization	1/295	101/12756	0.906763743	0.942908571	0.888319128	Soltu.DM.12G019570	1
1155	GO:0061982	meiosis I cell cycle process	1/295	103/12756	0.911060096	0.945436097	0.890700323	Soltu.DM.12G023260	1
1156	GO:0046486	glycerolipid metabolic process	2/295	173/12756	0.912503601	0.945436097	0.890700323	Soltu.DM.12G002120/Soltu.DM.10G005470	2
1157	GO:00	ribonucle	1/295	104/1275	0.91313	0.945436	0.890700323	Soltu.DM.12G004480	1

	09260	otide biosynthetic process		6	3697	097			
1158	GO:0016050	vesicle organization	1/295	104/12756	0.913133697	0.945436097	0.890700323	Soltu.DM.01G028770	1
1159	GO:0046390	ribose phosphate biosynthetic process	1/295	104/12756	0.913133697	0.945436097	0.890700323	Soltu.DM.12G004480	1
1160	GO:0016441	post-transcriptional gene silencing	1/295	108/12756	0.920957281	0.952714428	0.897557277	Soltu.DM.12G005490	1
1161	GO:0048527	lateral root development	1/295	109/12756	0.922800861	0.953799339	0.898579378	Soltu.DM.02G006700	1
1162	GO:0061024	membrane organization	2/295	181/12756	0.924895807	0.955141969	0.899844276	Soltu.DM.08G001900/Soltu.DM.10G022710	2
1163	GO:0090696	post-embryonic plant organ development	2/295	183/12756	0.927725385	0.956781864	0.90138923	Soltu.DM.02G024210/Soltu.DM.02G006700	2
1164	GO:0071824	protein-DNA complex subunit organization	1/295	112/12756	0.928078408	0.956781864	0.90138923	Soltu.DM.05G025150	1
1165	GO:0071482	cellular response to light stimulus	1/295	113/12756	0.929756427	0.957689024	0.90224387	Soltu.DM.10G005360	1
1166	GO:0051248	negative regulation of protein metabolic process	2/295	189/12756	0.935623415	0.962905744	0.90715857	Soltu.DM.08G022190/Soltu.DM.04G003450	2
1167	GO:0080135	regulation of cellular response to stress	1/295	118/12756	0.937578989	0.963266084	0.907498048	Soltu.DM.07G019630	1
1168	GO:0098657	import into cell	1/295	118/12756	0.937578989	0.963266084	0.907498048	Soltu.DM.01G037640	1
1169	GO:0009909	regulation of flower development	2/295	196/12756	0.943806259	0.966775058	0.91080387	Soltu.DM.11G022440/Soltu.DM.06G019760	2
1170	GO:00	multicellul	2/295	196/1275	0.94380	0.966775	0.91080387	Soltu.DM.07G022050/Soltu.DM.11G	2

	48609	ar organismal reproductive process		6	6259	058		009620	
1171	GO:0009691	cytokinin biosynthetic process	1/295	123/12756	0.944533026	0.966775058	0.91080387	Soltu.DM.02G025740	1
1172	GO:0008654	phospholipid biosynthetic process	1/295	124/12756	0.945828265	0.966775058	0.91080387	Soltu.DM.08G014180	1
1173	GO:0071478	cellular response to radiation	1/295	124/12756	0.945828265	0.966775058	0.91080387	Soltu.DM.10G005360	1
1174	GO:0090697	post-embryonic plant organ morphogenesis	1/295	124/12756	0.945828265	0.966775058	0.91080387	Soltu.DM.02G006700	1
1175	GO:0048528	post-embryonic root development	1/295	126/12756	0.948329006	0.968506219	0.912434806	Soltu.DM.02G006700	1
1176	GO:0006913	nucleocytoplasmic transport	1/295	130/12756	0.952990558	0.971613143	0.915361856	Soltu.DM.12G005490	1
1177	GO:0051169	nuclear transport	1/295	130/12756	0.952990558	0.971613143	0.915361856	Soltu.DM.12G005490	1
1178	GO:0060341	regulation of cellular localization	1/295	131/12756	0.954088909	0.971907208	0.915638896	Soltu.DM.08G027150	1
1179	GO:0033044	regulation of chromosome organization	1/295	132/12756	0.955161683	0.972174741	0.915890941	Soltu.DM.08G022190	1
1180	GO:0030258	lipid modification	1/295	133/12756	0.956209474	0.972416414	0.916118622	Soltu.DM.09G018310	1
1181	GO:0034050	programmed cell death induced by symbiont	1/295	135/12756	0.958232409	0.97364851	0.917279386	Soltu.DM.12G027350	1
1182	GO:0034248	regulation of amide metabolic	1/295	139/12756	0.962003108	0.975827329	0.919332062	Soltu.DM.08G029860	1

		process							
1183	GO:0051702	biological process involved in interaction with symbiont	1/295	139/12756	0.962003108	0.975827329	0.919332062	Soltu.DM.12G027350	1
1184	GO:0006457	protein folding	1/295	141/12756	0.963759227	0.975993301	0.919488426	Soltu.DM.03G035420	1
1185	GO:0010876	lipid localization	1/295	142/12756	0.964606712	0.975993301	0.919488426	Soltu.DM.02G033270	1
1186	GO:0031047	RNA-mediated gene silencing	1/295	142/12756	0.964606712	0.975993301	0.919488426	Soltu.DM.12G005490	1
1187	GO:0000398	mRNA splicing, via spliceosome	1/295	150/12756	0.970712734	0.98134396	0.924529309	Soltu.DM.12G005490	1
1188	GO:0071103	DNA conformation change	1/295	153/12756	0.972721251	0.982364779	0.925491028	Soltu.DM.05G025150	1
1189	GO:0010564	regulation of cell cycle process	1/295	154/12756	0.973359768	0.982364779	0.925491028	Soltu.DM.11G023760	1
1190	GO:0022618	ribonucleoprotein complex assembly	1/295	157/12756	0.975187319	0.982874113	0.925970875	Soltu.DM.08G019530	1
1191	GO:0045944	positive regulation of transcription by RNA polymerase II	1/295	158/12756	0.975768297	0.982874113	0.925970875	Soltu.DM.08G019590	1
1192	GO:0009739	response to gibberellin	1/295	160/12756	0.976889893	0.982874113	0.925970875	Soltu.DM.10G026020	1
1193	GO:0071826	ribonucleoprotein complex subunit organization	1/295	161/12756	0.977431135	0.982874113	0.925970875	Soltu.DM.08G019530	1
1194	GO:0051336	regulation of hydrolase activity	1/295	162/12756	0.977959743	0.982874113	0.925970875	Soltu.DM.04G003450	1
1195	GO:1902531	regulation of intracellul	1/295	165/12756	0.979472667	0.983570879	0.926627302	Soltu.DM.08G027110	1

		ar signal transduction							
1196	GO:0000377	RNA splicing, via transesterification reactions with bulged adenosine as nucleophile	1/295	167/12756	0.980423324	0.983702332	0.926751144	Soltu.DM.12G005490	1
1197	GO:0000375	RNA splicing, via transesterification reactions	1/295	169/12756	0.981330096	0.98378957	0.926833332	Soltu.DM.12G005490	1
1198	GO:0051130	positive regulation of cellular component organization	1/295	171/12756	0.982195004	0.983834728	0.926875876	Soltu.DM.12G019570	1
1199	GO:0006310	DNA recombination	1/295	177/12756	0.984557364	0.985378513	0.928330283	Soltu.DM.12G023260	1
1200	GO:0006364	rRNA processing	1/295	198/12756	0.990621905	0.990621905	0.93327011	Soltu.DM.10G015180	1

Number	ID	Description	BgRatio	pvalue	p.adjust	qvalue	geneID	Count
1	GO:0009787	regulation of abscisic acid-activated signaling pathway	131/12756	0.035375003	0.402716375	0.380336848	Soltu.DM.08G023690/Soltu.DM.07G017210/Soltu.DM.07G017190/Soltu.DM.07G017200/Soltu.DM.04G033590/Soltu.DM.07G017180/Soltu.DM.07G012130/Soltu.DM.01G046820/Soltu.DM.09G028490/Soltu.DM.04G037130/Soltu.DM.06G017300/Soltu.DM.01G000060/Soltu.DM.04G024100/Soltu.DM.08G011890/Soltu.DM.09G026500/Soltu.DM.04G005970/Soltu.DM.02G026820/Soltu.DM.10G001460/Soltu.DM.07G024500	19
2	GO:0009738	abscisic acid-activated signaling pathway	130/12756	0.000884924	0.054317453	0.051298954	Soltu.DM.09G019250/Soltu.DM.06G026960/Soltu.DM.07G017210/Soltu.DM.04G000490/Soltu.DM.09G031320/Soltu.DM.07G017190/Soltu.DM.07G017200/Soltu.DM.04G033590/Soltu.DM.03G017570/Soltu.DM.07G017180/Soltu.DM.06G034820/Soltu.DM.08G028440/Soltu.DM.01G000060/Soltu.DM.06G003060/Soltu.DM.05G000860/Soltu.DM.03G021780/Soltu.DM.08G011890/Soltu.DM.02G023840/Soltu.DM.07G026270/Soltu.DM.09G031340/Soltu.DM.04G000670/Soltu.DM.03G016650/Soltu.DM.11G004950/Soltu.DM.01G024340	24
3	GO:0071215	cellular response to abscisic acid stimulus	177/12756	0.00196284	0.08295412 1	0.07834424 2	Soltu.DM.09G019250/Soltu.DM.06G026960/Soltu.DM.07G017210/Soltu.DM.04G000490/Soltu.DM.09G031320/Soltu.DM.07G017190/Soltu.DM.07G017200/Soltu.DM.04G033590/Soltu.DM.03G017570/Soltu.DM.02G018520/Soltu.DM.07G017180/Soltu.DM.08G008380/Soltu.DM.06G034820/Soltu.DM.08G028440/Soltu.DM.01G000060/Soltu.DM.09G016850/Soltu.DM.06G003060/Soltu.DM.05G000860/Soltu.DM.03G021780/Soltu.DM.05G011970/Soltu.DM.08G011890/Soltu.DM.02G023840/Soltu.DM.07G026270/Soltu.DM.12G010960/Soltu.DM.09G031340/Soltu.DM.04G000670/Soltu.DM.03G016650/Soltu.DM.11G004950/Soltu.DM.01G024340	29
4	GO:0009789	positive regulation of abscisic acid-activated signaling pathway	55/12756	0.011657812	0.231576996	0.218707931	Soltu.DM.09G018310/Soltu.DM.07G017210/Soltu.DM.07G017190/Soltu.DM.07G017200/Soltu.DM.04G033590/Soltu.DM.07G017180/Soltu.DM.09G028490/Soltu.DM.04G037130/Soltu.DM.08G011890/Soltu.DM.09G026500/Soltu.DM.02G026820	11

5	GO:0046345	abscisic acid catabolic process	17/12756	0.208016893	0.893779252	0.844110657	Soltu.DM.08G020150/Soltu.DM.07G013940/Soltu.DM.07G013900	3
6	GO:0009687	abscisic acid metabolic process	40/12756	0.317266558	0.999994594	0.94442346	Soltu.DM.08G020150/Soltu.DM.11G024450/Soltu.DM.07G013940/Soltu.DM.06G029640/Soltu.DM.07G013900	5
7	GO:0009788	negative regulation of abscisic acid-activated signaling pathway	61/12756	0.342973763	0.999994594	0.94442346	Soltu.DM.07G012130/Soltu.DM.01G046820/Soltu.DM.06G017300/Soltu.DM.01G000060/Soltu.DM.04G024100/Soltu.DM.08G011890/Soltu.DM.04G005970	7
8	GO:0010115	regulation of abscisic acid biosynthetic process	8/12756	0.543910229	0.999994594	0.94442346	Soltu.DM.02G020950	1
9	GO:0009688	abscisic acid biosynthetic process	20/12756	0.569840814	0.999994594	0.94442346	Soltu.DM.11G024450/Soltu.DM.06G029640	2
10	GO:0080168	abscisic acid transport	20/12756	0.569840814	0.999994594	0.94442346	Soltu.DM.09G028710/Soltu.DM.11G011430	2
11	GO:0009809	lignin biosynthetic process	73/12756	0.002694848	0.095168739	0.089880076	Soltu.DM.04G027660/Soltu.DM.03G031830/Soltu.DM.11G002020/Soltu.DM.07G028550/Soltu.DM.09G000560/Soltu.DM.10G029960/Soltu.DM.10G000640/Soltu.DM.01G044300/Soltu.DM.02G019030/Soltu.DM.03G021440/Soltu.DM.09G000850/Soltu.DM.02G024380/Soltu.DM.03G002800/Soltu.DM.11G002250/Soltu.DM.11G002650	15
12	GO:0009694	jasmonic acid metabolic process	31/12756	2.11E-10	5.44E-07	5.14E-07	Soltu.DM.02G025590/Soltu.DM.08G024620/Soltu.DM.09G018310/Soltu.DM.04G034690/Soltu.DM.03G024660/Soltu.DM.03G024680/Soltu.DM.10G027770/Soltu.DM.07G003270/Soltu.DM.12G004930/Soltu.DM.11G023180/Soltu.DM.03G024670/Soltu.DM.09G028490/Soltu.DM.01G035830/Soltu.DM.03G024690/Soltu.DM.05G011130/Soltu.DM.11G001520/Soltu.DM.02G019940	17
13	GO:2000022	regulation of jasmonic acid mediated signaling pathway	39/12756	1.45E-07	0.000186632	0.00017626	Soltu.DM.07G012950/Soltu.DM.08G007190/Soltu.DM.08G007150/Soltu.DM.04G033180/Soltu.DM.08G007100/Soltu.DM.03G032770/Soltu.DM.12G026270/Soltu.DM.03G036980/Soltu.DM.05G012690/Soltu.DM.10G019650/Soltu.DM.07G014680/Soltu.DM.08G007110/Soltu.DM.10G019640/Soltu.DM.01G000760/Soltu.DM.10G022640/Soltu.DM.03G013090	16
14	GO:0042546	cell wall biogenesis	154/12756	1.54E-06	0.001327502	0.001253731	Soltu.DM.02G028360/Soltu.DM.04G027320/Soltu.DM.12G007610/Soltu.DM.02G009140/Soltu.DM.10G001690/Soltu.DM.02G031090/Soltu.DM.08G027650/Soltu.DM.09G000560/Soltu.DM.08G029290/Soltu.DM.06G009650/Soltu.DM.02G025970/Soltu.DM.07G000930/Soltu.DM.12G008450/Soltu.DM.10G019900/Soltu.DM.02G015410/Soltu.DM.06G010280/Soltu.DM.03G013120/Soltu.DM.02G030660/Soltu.DM.04G020370/Soltu.DM.04G029850/Soltu.DM.02G012570/Soltu.DM.07G025450/Soltu.DM.10G000250/Soltu.DM.04G000320/Soltu.DM.04G022240/Soltu.DM.05G001020/Soltu.DM.06G021870/Soltu.DM.09G006300/Soltu.DM.08G005070/Soltu.DM.09G007590/Soltu.DM.01G027520/Soltu.DM.11G008240/Soltu.DM.03G006820/Soltu.DM.02G014140	34
15	GO:0009695	jasmonic acid biosynthetic process	24/12756	3.60E-06	0.002318414	0.002189577	Soltu.DM.02G025590/Soltu.DM.08G024620/Soltu.DM.09G018310/Soltu.DM.04G034690/Soltu.DM.07G003270/Soltu.DM.12G004930/Soltu.DM.11G023180/Soltu.DM.09G028490/Soltu.DM.05G011130/Soltu.DM.11G001520/Soltu.DM.02G019940	11
16	GO:0000271	polysaccharide biosynthetic process	135/12756	5.46E-06	0.002814869	0.002658443	Soltu.DM.09G018910/Soltu.DM.02G028360/Soltu.DM.01G040570/Soltu.DM.04G027320/Soltu.DM.12G007610/Soltu.DM.10G001690/Soltu.DM.02G031090/Soltu.DM.08G027650/Soltu.DM.01G022940/Soltu.DM.08G029290/Soltu.DM.06G009650/Soltu.DM.07G000930/Soltu.DM.12G008450/Soltu.DM.02G015410/Soltu.DM.02G027540/Soltu.DM.03G013120/Soltu.DM.04G029850/Soltu.DM.02G024820/Soltu.DM.09G031790/Soltu.DM.07G025450/Soltu.DM.04G022240/Soltu.DM.06G009750/Soltu.DM.05G009320/Soltu.DM.06G021870/Soltu.DM.09G006300/Soltu.DM.01G051470/Soltu.DM.05G005090/Soltu.DM.09G007590/Soltu.DM.11G008240/Soltu.DM.02G014140	30
17	GO:0034637	cellular carbohydrate biosynthetic process	154/12756	1.19E-05	0.004592395	0.004337189	Soltu.DM.04G012960/Soltu.DM.09G018910/Soltu.DM.02G015310/Soltu.DM.02G028360/Soltu.DM.01G040570/Soltu.DM.04G027320/Soltu.DM.12G007610/Soltu.DM.10G001690/Soltu.DM.02G031090/Soltu.DM.08G029290/Soltu.DM.07G001730/Soltu.DM.06G009650/Soltu.DM.07G000930/Soltu.DM.12G008450/Soltu.DM.02G015410/Soltu.DM.03G013120/Soltu.DM.04G029850/Soltu.DM.02G024820/Soltu.DM.09G031790/Soltu.DM.07G025450/Soltu.DM.05G008060/Soltu.DM.04G022240/Soltu.DM.06G009750/Soltu.DM.05G009320/Soltu.DM.06G021870/Soltu.DM.09G006300/Soltu.DM.01G051470/Soltu.DM.05G005090/Soltu.DM.09G007590/Soltu.DM.11G008240/Soltu.DM.02G014140/Soltu.DM.07G014750	32
18	GO:0033692	cellular polysaccharide biosynthetic process	120/12756	1.25E-05	0.004592395	0.004337189	Soltu.DM.09G018910/Soltu.DM.02G028360/Soltu.DM.01G040570/Soltu.DM.04G027320/Soltu.DM.12G007610/Soltu.DM.10G001690/Soltu.DM.02G031090/Soltu.DM.08G029290/Soltu.DM.06G009650/Soltu.DM.07G000930/Soltu.DM.12G008450/Soltu.DM.02G015410/Soltu.DM.03G013120/Soltu.DM.04G029850/Soltu.DM.02G024820/Soltu.DM.09G031790/Soltu.DM.07G025450/Soltu.DM.04G022240/Soltu.DM.06G009750/Soltu.DM.05G009320/Soltu.D	27

							M.06G021870/Soltu.DM.09G006300/Soltu.DM.01G051470/Soltu.DM.05G005090/Soltu.DM.09G007590/Soltu.DM.11G008240/Soltu.DM.02G014140	
19	GO:0080167	response to karrikin	161/12756	3.05E-05	0.009842477	0.009295516	Soltu.DM.06G032850/Soltu.DM.03G020490/Soltu.DM.06G032860/Soltu.DM.12G007510/Soltu.DM.01G035900/Soltu.DM.07G022460/Soltu.DM.07G022500/Soltu.DM.07G022050/Soltu.DM.07G022530/Soltu.DM.04G027760/Soltu.DM.09G025070/Soltu.DM.07G022490/Soltu.DM.05G022850/Soltu.DM.10G003300/Soltu.DM.03G027330/Soltu.DM.06G012790/Soltu.DM.10G016030/Soltu.DM.01G035170/Soltu.DM.11G021810/Soltu.DM.01G050280/Soltu.DM.08G001820/Soltu.DM.01G025080/Soltu.DM.07G022450/Soltu.DM.10G005400/Soltu.DM.11G008180/Soltu.DM.01G035910/Soltu.DM.07G026100/Soltu.DM.03G027340/Soltu.DM.03G021710/Soltu.DM.03G013090/Soltu.DM.01G029270/Soltu.DM.04G031480	32
20	GO:0009832	plant-type cell wall biogenesis	120/12756	3.61E-05	0.010161459	0.009596772	Soltu.DM.02G028360/Soltu.DM.04G027320/Soltu.DM.12G007610/Soltu.DM.02G009140/Soltu.DM.02G031090/Soltu.DM.09G000560/Soltu.DM.08G029290/Soltu.DM.07G000930/Soltu.DM.12G008450/Soltu.DM.10G019900/Soltu.DM.02G015410/Soltu.DM.06G010280/Soltu.DM.02G030660/Soltu.DM.04G020370/Soltu.DM.04G029850/Soltu.DM.02G012570/Soltu.DM.04G000320/Soltu.DM.04G022240/Soltu.DM.05G001020/Soltu.DM.06G021870/Soltu.DM.09G006300/Soltu.DM.08G005070/Soltu.DM.01G027520/Soltu.DM.11G008240/Soltu.DM.03G006820/Soltu.DM.02G014140	26
21	GO:0009698	phenylpropanoid metabolic process	163/12756	3.94E-05	0.010161459	0.009596772	Soltu.DM.06G032850/Soltu.DM.06G032860/Soltu.DM.06G024250/Soltu.DM.03G031830/Soltu.DM.05G021610/Soltu.DM.10G020990/Soltu.DM.11G002020/Soltu.DM.07G028550/Soltu.DM.03G020790/Soltu.DM.05G026870/Soltu.DM.09G000560/Soltu.DM.03G035070/Soltu.DM.10G029960/Soltu.DM.10G000640/Soltu.DM.05G025440/Soltu.DM.10G019900/Soltu.DM.02G019030/Soltu.DM.03G021440/Soltu.DM.06G024540/Soltu.DM.09G000850/Soltu.DM.03G032090/Soltu.DM.02G024380/Soltu.DM.12G024890/Soltu.DM.07G007400/Soltu.DM.03G035080/Soltu.DM.03G002800/Soltu.DM.06G020450/Soltu.DM.10G026590/Soltu.DM.08G026700/Soltu.DM.11G002250/Soltu.DM.03G000410/Soltu.DM.11G002650	32
22	GO:0010410	hemicellulose metabolic process	57/12756	4.40E-05	0.01032119	0.009747626	Soltu.DM.09G018910/Soltu.DM.02G028360/Soltu.DM.10G001690/Soltu.DM.01G022940/Soltu.DM.08G029290/Soltu.DM.06G009650/Soltu.DM.12G008450/Soltu.DM.06G010280/Soltu.DM.03G013120/Soltu.DM.01G003570/Soltu.DM.07G025450/Soltu.DM.11G011390/Soltu.DM.09G006300/Soltu.DM.09G007590/Soltu.DM.11G008240/Soltu.DM.02G014140	16
23	GO:1900140	regulation of seedling development	132/12756	7.44E-05	0.015994043	0.015105231	Soltu.DM.09G019250/Soltu.DM.04G037380/Soltu.DM.01G018690/Soltu.DM.02G016290/Soltu.DM.05G007640/Soltu.DM.09G025070/Soltu.DM.06G034820/Soltu.DM.05G007630/Soltu.DM.01G045040/Soltu.DM.05G011970/Soltu.DM.02G016300/Soltu.DM.01G035240/Soltu.DM.01G045030/Soltu.DM.03G017660/Soltu.DM.02G016770/Soltu.DM.02G016380/Soltu.DM.10G005360/Soltu.DM.01G024340/Soltu.DM.01G020640/Soltu.DM.07G020080/Soltu.DM.02G016780/Soltu.DM.06G013150/Soltu.DM.10G001460/Soltu.DM.06G002140/Soltu.DM.04G033440/Soltu.DM.01G045020/Soltu.DM.07G022640	27
24	GO:0010029	regulation of seed germination	126/12756	8.62E-05	0.017095872	0.01614583	Soltu.DM.09G019250/Soltu.DM.04G037380/Soltu.DM.01G018690/Soltu.DM.02G016290/Soltu.DM.05G007640/Soltu.DM.09G025070/Soltu.DM.06G034820/Soltu.DM.05G007630/Soltu.DM.01G045040/Soltu.DM.05G011970/Soltu.DM.02G016300/Soltu.DM.01G035240/Soltu.DM.01G045030/Soltu.DM.03G017660/Soltu.DM.02G016770/Soltu.DM.02G016380/Soltu.DM.10G005360/Soltu.DM.01G024340/Soltu.DM.01G020640/Soltu.DM.07G020080/Soltu.DM.02G016780/Soltu.DM.06G013150/Soltu.DM.10G001460/Soltu.DM.06G002140/Soltu.DM.04G033440/Soltu.DM.01G045020	26
25	GO:0044036	cell wall macromolecule metabolic process	121/12756	0.000114575	0.021098229	0.01992577	Soltu.DM.09G018910/Soltu.DM.02G028360/Soltu.DM.10G001690/Soltu.DM.02G031090/Soltu.DM.01G022940/Soltu.DM.08G029290/Soltu.DM.06G009650/Soltu.DM.02G025970/Soltu.DM.07G000930/Soltu.DM.12G008450/Soltu.DM.06G006590/Soltu.DM.06G010280/Soltu.DM.03G013120/Soltu.DM.11G000740/Soltu.DM.01G003570/Soltu.DM.09G005280/Soltu.DM.04G029850/Soltu.DM.07G025450/Soltu.DM.11G011390/Soltu.DM.06G021870/Soltu.DM.09G006300/Soltu.DM.06G006580/Soltu.DM.09G007590/Soltu.DM.11G008240/Soltu.DM.02G014140	25
26	GO:0044038	cell wall macromolecule biosynthetic process	64/12756	0.000198637	0.030471748	0.028778389	Soltu.DM.09G018910/Soltu.DM.02G028360/Soltu.DM.10G001690/Soltu.DM.02G031090/Soltu.DM.08G029290/Soltu.DM.06G009650/Soltu.DM.02G025970/Soltu.DM.12G008450/Soltu.DM.03G013120/Soltu.DM.04G029850/Soltu.DM.07G025450/Soltu.DM.06G021870/Soltu.DM.09G006300/Soltu.DM.09G007590/Soltu.DM.11G008240/Soltu.DM.02G014140	16
27	GO:0070589	cellular component macromolecule biosynthetic process	64/12756	0.000198637	0.030471748	0.028778389	Soltu.DM.09G018910/Soltu.DM.02G028360/Soltu.DM.10G001690/Soltu.DM.02G031090/Soltu.DM.08G029290/Soltu.DM.06G009650/Soltu.DM.02G025970/Soltu.DM.12G008450/Soltu.DM.03G013120/Soltu.DM.04G029850/Soltu.DM.07G025450/Soltu.DM.06G021870/Soltu.DM.09G006300/Soltu.DM.09G007590/Soltu.DM.11G008240/Soltu.DM.02G014140	16
28	GO:0098754	detoxification	156/12756	0.000240671	0.030471748	0.028778389	Soltu.DM.04G027640/Soltu.DM.04G027650/Soltu.DM.01G044300/Soltu.DM.02G031330/Soltu.DM.02G023420/Soltu.DM.07G022460/Soltu.DM.07G022500/Soltu.DM.09G021720/Soltu.DM.01G047440/Soltu.DM.07G022530/Soltu.DM.02G022700/Soltu.DM.09G003770/Soltu.DM.02G024520/Soltu.DM.07G022490/Soltu.DM.10G003300/Soltu.DM.06G012170/Soltu.DM.02G019520/Soltu.DM.02G023590/Soltu.DM.05G018830/Soltu.DM.08G011330/Soltu.DM.07G022450/Soltu.DM.02G023580/Soltu.DM.09G001260/Soltu.DM.12G028960/Soltu.DM.05G018810/Soltu.DM.01G028030/Soltu.DM.12G003880/Soltu.DM.09G005310/Soltu.DM.03G013100	29
29	GO:0055088	lipid homeostasis	59/12756	0.000255551	0.030471748	0.028778389	Soltu.DM.08G020150/Soltu.DM.08G029860/Soltu.DM.03G017780/Soltu.DM.01G030110/Soltu.DM.05G015440/Soltu.DM.06G004460/Soltu.DM.03G017800/Soltu.DM.10G004300/Soltu.DM.02G030630/Soltu.DM.12G024720/Soltu.DM.06G005440/Soltu.DM.10G003570/Soltu.DM.10G001460/Soltu.DM.08G027080/Soltu.DM.10G003550	15
30	GO:00	sucrose catabolic process	8/1	0.0	0.0	0.0	Soltu.DM.05G006330/Soltu.DM.01G018690/Soltu.DM.01G040550/Soltu.DM.04G037250/S	5

	05987		2756	00310985	30471748	28778389	oltu.DM.06G020260	
31	GO:0003333	amino acid transmembrane transport	73/12756	0.000318449	0.030471748	0.028778389	Soltu.DM.05G003990/Soltu.DM.09G002620/Soltu.DM.04G031760/Soltu.DM.03G011160/Soltu.DM.03G003280/Soltu.DM.12G006380/Soltu.DM.02G030410/Soltu.DM.11G011180/Soltu.DM.05G022850/Soltu.DM.06G015630/Soltu.DM.10G028240/Soltu.DM.08G003390/Soltu.DM.01G001450/Soltu.DM.06G018560/Soltu.DM.06G017010/Soltu.DM.04G034490/Soltu.DM.11G021200	17
32	GO:0006865	amino acid transport	73/12756	0.000318449	0.030471748	0.028778389	Soltu.DM.05G003990/Soltu.DM.09G002620/Soltu.DM.04G031760/Soltu.DM.03G011160/Soltu.DM.03G003280/Soltu.DM.12G006380/Soltu.DM.02G030410/Soltu.DM.11G011180/Soltu.DM.05G022850/Soltu.DM.06G015630/Soltu.DM.10G028240/Soltu.DM.08G003390/Soltu.DM.01G001450/Soltu.DM.06G018560/Soltu.DM.06G017010/Soltu.DM.04G034490/Soltu.DM.11G021200	17
33	GO:0009834	plant-type secondary cell wall biogenesis	48/12756	0.000333389	0.030471748	0.028778389	Soltu.DM.02G028360/Soltu.DM.09G000560/Soltu.DM.07G000930/Soltu.DM.10G019900/Soltu.DM.02G015410/Soltu.DM.06G010280/Soltu.DM.02G030660/Soltu.DM.09G006300/Soltu.DM.08G005070/Soltu.DM.01G027520/Soltu.DM.11G008240/Soltu.DM.03G006820/Soltu.DM.02G014140	13
34	GO:0048480	stigma development	5/12756	0.000351233	0.030471748	0.028778389	Soltu.DM.03G024660/Soltu.DM.03G024680/Soltu.DM.03G024670/Soltu.DM.03G024690	4
35	GO:0018973	trinitrotoluene metabolic process	12/12756	0.000370314	0.030471748	0.028778389	Soltu.DM.07G022460/Soltu.DM.07G022500/Soltu.DM.07G022530/Soltu.DM.07G022490/Soltu.DM.10G003300/Soltu.DM.07G022450	6
36	GO:0018974	2,4,6-trinitrotoluene metabolic process	12/12756	0.000370314	0.030471748	0.028778389	Soltu.DM.07G022460/Soltu.DM.07G022500/Soltu.DM.07G022530/Soltu.DM.07G022490/Soltu.DM.10G003300/Soltu.DM.07G022450	6
37	GO:0019326	nitrotoluene metabolic process	12/12756	0.000370314	0.030471748	0.028778389	Soltu.DM.07G022460/Soltu.DM.07G022500/Soltu.DM.07G022530/Soltu.DM.07G022490/Soltu.DM.10G003300/Soltu.DM.07G022450	6
38	GO:0046256	2,4,6-trinitrotoluene catabolic process	12/12756	0.000370314	0.030471748	0.028778389	Soltu.DM.07G022460/Soltu.DM.07G022500/Soltu.DM.07G022530/Soltu.DM.07G022490/Soltu.DM.10G003300/Soltu.DM.07G022450	6
39	GO:0046260	trinitrotoluene catabolic process	12/12756	0.000370314	0.030471748	0.028778389	Soltu.DM.07G022460/Soltu.DM.07G022500/Soltu.DM.07G022530/Soltu.DM.07G022490/Soltu.DM.10G003300/Soltu.DM.07G022450	6
40	GO:0046263	nitrotoluene catabolic process	12/12756	0.000370314	0.030471748	0.028778389	Soltu.DM.07G022460/Soltu.DM.07G022500/Soltu.DM.07G022530/Soltu.DM.07G022490/Soltu.DM.10G003300/Soltu.DM.07G022450	6
41	GO:0072490	toluene-containing compound metabolic process	12/12756	0.000370314	0.030471748	0.028778389	Soltu.DM.07G022460/Soltu.DM.07G022500/Soltu.DM.07G022530/Soltu.DM.07G022490/Soltu.DM.10G003300/Soltu.DM.07G022450	6
42	GO:0072491	toluene-containing compound catabolic process	12/12756	0.000370314	0.030471748	0.028778389	Soltu.DM.07G022460/Soltu.DM.07G022500/Soltu.DM.07G022530/Soltu.DM.07G022490/Soltu.DM.10G003300/Soltu.DM.07G022450	6
43	GO:0070592	cell wall polysaccharide biosynthetic process	61/12756	0.000378237	0.030471748	0.028778389	Soltu.DM.09G018910/Soltu.DM.02G028360/Soltu.DM.10G001690/Soltu.DM.02G031090/Soltu.DM.08G029290/Soltu.DM.06G009650/Soltu.DM.12G008450/Soltu.DM.03G013120/Soltu.DM.04G029850/Soltu.DM.07G025450/Soltu.DM.06G021870/Soltu.DM.09G006300/Soltu.DM.09G007590/Soltu.DM.11G008240/Soltu.DM.02G014140	15
44	GO:0009407	toxin catabolic process	27/12756	0.000522104	0.040386365	0.038142036	Soltu.DM.07G022460/Soltu.DM.07G022500/Soltu.DM.07G022530/Soltu.DM.07G022490/Soltu.DM.10G003300/Soltu.DM.07G022450/Soltu.DM.09G001260/Soltu.DM.12G028960/Soltu.DM.01G028030	9
45	GO:0010383	cell wall polysaccharide metabolic process	104/12756	0.00054243	0.040386365	0.038142036	Soltu.DM.09G018910/Soltu.DM.02G028360/Soltu.DM.10G001690/Soltu.DM.02G031090/Soltu.DM.01G022940/Soltu.DM.08G029290/Soltu.DM.06G009650/Soltu.DM.07G000930/Soltu.DM.12G008450/Soltu.DM.06G010280/Soltu.DM.03G013120/Soltu.DM.11G000740/Soltu.DM.01G003570/Soltu.DM.04G029850/Soltu.DM.07G025450/Soltu.DM.11G011390/Soltu.DM.06G021870/Soltu.DM.09G006300/Soltu.DM.09G007590/Soltu.DM.11G008240/Soltu.DM.02G014140	21
46	GO:0042537	benzene-containing compound metabolic process	63/12756	0.000548302	0.040386365	0.038142036	Soltu.DM.06G028410/Soltu.DM.07G022460/Soltu.DM.07G022500/Soltu.DM.05G007640/Soltu.DM.07G022530/Soltu.DM.07G022490/Soltu.DM.10G003300/Soltu.DM.05G007630/Soltu.DM.10G004300/Soltu.DM.07G022450/Soltu.DM.06G020450/Soltu.DM.04G009170/Soltu.DM.02G008560/Soltu.DM.09G023400/Soltu.DM.02G008550	15

47	GO:0009310	amine catabolic process	9/12756	0.000645822	0.044998075	0.042497467	Soltu.DM.07G014310/Soltu.DM.03G035070/Soltu.DM.02G020890/Soltu.DM.03G035080/Soltu.DM.01G027080	5
48	GO:0042402	cellular biogenic amine catabolic process	9/12756	0.000645822	0.044998075	0.042497467	Soltu.DM.07G014310/Soltu.DM.03G035070/Soltu.DM.02G020890/Soltu.DM.03G035080/Soltu.DM.01G027080	5
49	GO:1901264	carbohydrate derivative transport	92/12756	0.000733476	0.048912757	0.046194605	Soltu.DM.04G030440/Soltu.DM.12G004580/Soltu.DM.10G000530/Soltu.DM.01G038470/Soltu.DM.11G022930/Soltu.DM.01G008290/Soltu.DM.12G004060/Soltu.DM.12G007520/Soltu.DM.10G022360/Soltu.DM.03G027330/Soltu.DM.06G024610/Soltu.DM.03G032350/Soltu.DM.12G024350/Soltu.DM.06G012690/Soltu.DM.03G034530/Soltu.DM.03G032030/Soltu.DM.03G027340/Soltu.DM.06G025410/Soltu.DM.11G004080	19
50	GO:0030162	regulation of proteolysis	121/12756	0.000739952	0.048912757	0.046194605	Soltu.DM.06G026960/Soltu.DM.09G031320/Soltu.DM.03G020450/Soltu.DM.04G034360/Soltu.DM.11G011280/Soltu.DM.08G027150/Soltu.DM.03G034960/Soltu.DM.04G000940/Soltu.DM.12G005510/Soltu.DM.04G034390/Soltu.DM.04G034380/Soltu.DM.03G037730/Soltu.DM.08G006060/Soltu.DM.04G003450/Soltu.DM.06G015770/Soltu.DM.07G014680/Soltu.DM.04G034740/Soltu.DM.09G031340/Soltu.DM.09G005140/Soltu.DM.09G002090/Soltu.DM.04G034280/Soltu.DM.06G018040/Soltu.DM.06G024530	23
51	GO:0044242	cellular lipid catabolic process	144/12756	0.000781136	0.050344241	0.047546539	Soltu.DM.07G022720/Soltu.DM.08G024620/Soltu.DM.02G034460/Soltu.DM.07G022710/Soltu.DM.09G018310/Soltu.DM.01G033530/Soltu.DM.02G018520/Soltu.DM.08G020150/Soltu.DM.02G016290/Soltu.DM.01G038470/Soltu.DM.09G028490/Soltu.DM.07G013940/Soltu.DM.07G022700/Soltu.DM.07G013900/Soltu.DM.10G003240/Soltu.DM.06G018150/Soltu.DM.05G012150/Soltu.DM.02G016300/Soltu.DM.06G034310/Soltu.DM.01G005590/Soltu.DM.02G016770/Soltu.DM.03G024130/Soltu.DM.02G016380/Soltu.DM.02G016780/Soltu.DM.10G003920/Soltu.DM.02G019940	26
52	GO:0009734	auxin-activated signaling pathway	79/12756	0.000842421	0.05296978	0.050026173	Soltu.DM.02G025590/Soltu.DM.09G002620/Soltu.DM.06G026960/Soltu.DM.10G026390/Soltu.DM.12G022190/Soltu.DM.11G011180/Soltu.DM.12G023260/Soltu.DM.06G018130/Soltu.DM.09G020190/Soltu.DM.02G004510/Soltu.DM.04G029270/Soltu.DM.01G040670/Soltu.DM.02G032660/Soltu.DM.04G002510/Soltu.DM.02G027780/Soltu.DM.04G021540/Soltu.DM.06G034230	17
53	GO:0045492	xylan biosynthetic process	29/12756	0.000940136	0.056364431	0.053232179	Soltu.DM.02G028360/Soltu.DM.10G001690/Soltu.DM.06G009650/Soltu.DM.03G013120/Soltu.DM.07G025450/Soltu.DM.09G006300/Soltu.DM.09G007590/Soltu.DM.11G008240/Soltu.DM.02G014140	9
54	GO:1902457	negative regulation of stomatal opening	6/12756	0.000975427	0.056465539	0.053327668	Soltu.DM.07G017210/Soltu.DM.07G017190/Soltu.DM.07G017200/Soltu.DM.07G017180	4
55	GO:0015866	ADP transport	14/12756	0.001021346	0.056465539	0.053327668	Soltu.DM.01G038470/Soltu.DM.06G024610/Soltu.DM.03G032350/Soltu.DM.12G024350/Soltu.DM.03G034530/Soltu.DM.06G025410	6
56	GO:0015807	L-amino acid transport	41/12756	0.001029434	0.056465539	0.053327668	Soltu.DM.05G003990/Soltu.DM.04G031760/Soltu.DM.12G006380/Soltu.DM.11G011180/Soltu.DM.05G022850/Soltu.DM.06G015630/Soltu.DM.10G028240/Soltu.DM.08G003390/Soltu.DM.01G001450/Soltu.DM.06G018560/Soltu.DM.06G017010	11
57	GO:1902475	L-alpha-amino acid transmembrane transport	41/12756	0.001029434	0.056465539	0.053327668	Soltu.DM.05G003990/Soltu.DM.04G031760/Soltu.DM.12G006380/Soltu.DM.11G011180/Soltu.DM.05G022850/Soltu.DM.06G015630/Soltu.DM.10G028240/Soltu.DM.08G003390/Soltu.DM.01G001450/Soltu.DM.06G018560/Soltu.DM.06G017010	11
58	GO:0009699	phenylpropanoid biosynthetic process	147/12756	0.001071552	0.057551297	0.054353088	Soltu.DM.06G024250/Soltu.DM.03G031830/Soltu.DM.05G021610/Soltu.DM.10G020990/Soltu.DM.11G002020/Soltu.DM.07G028550/Soltu.DM.05G026870/Soltu.DM.09G000560/Soltu.DM.10G029960/Soltu.DM.10G000640/Soltu.DM.05G025440/Soltu.DM.10G019900/Soltu.DM.02G019030/Soltu.DM.03G021440/Soltu.DM.06G024540/Soltu.DM.09G000850/Soltu.DM.03G032090/Soltu.DM.02G024380/Soltu.DM.12G024890/Soltu.DM.03G002800/Soltu.DM.06G020450/Soltu.DM.10G026590/Soltu.DM.08G026700/Soltu.DM.11G002250/Soltu.DM.03G000410/Soltu.DM.11G002650	26
59	GO:1990641	response to iron ion starvation	10/12756	0.001192405	0.061445276	0.058030673	Soltu.DM.07G028550/Soltu.DM.10G000640/Soltu.DM.07G017750/Soltu.DM.07G015200/Soltu.DM.02G027940	5
60	GO:0016054	organic acid catabolic process	156/12756	0.001215558	0.061445276	0.058030673	Soltu.DM.07G022720/Soltu.DM.06G028410/Soltu.DM.08G024620/Soltu.DM.07G022710/Soltu.DM.09G018310/Soltu.DM.04G025250/Soltu.DM.01G033530/Soltu.DM.08G013400/Soltu.DM.08G020150/Soltu.DM.01G038470/Soltu.DM.03G035070/Soltu.DM.08G030020/Soltu.DM.09G028490/Soltu.DM.07G013940/Soltu.DM.07G019380/Soltu.DM.07G022700/Soltu.DM.01G047450/Soltu.DM.12G024030/Soltu.DM.07G013900/Soltu.DM.10G003240/Soltu.DM.06G018150/Soltu.DM.03G035080/Soltu.DM.03G017660/Soltu.DM.08G007450/Soltu.DM.04G009170/Soltu.DM.06G013150/Soltu.DM.10G003920	27
61	GO:0046395	carboxylic acid catabolic process	156/1	0.0012	0.0614	0.0580	Soltu.DM.07G022720/Soltu.DM.06G028410/Soltu.DM.08G024620/Soltu.DM.07G022710/Soltu.DM.09G018310/Soltu.DM.04G025250/Soltu.DM.01G033530/Soltu.DM.08G013400/Sol	27

			2756	15558	45276	30673	tu.DM.08G020150/Soltu.DM.01G038470/Soltu.DM.03G035070/Soltu.DM.08G030020/Soltu.DM.09G028490/Soltu.DM.07G013940/Soltu.DM.07G019380/Soltu.DM.07G022700/Soltu.DM.01G047450/Soltu.DM.12G024030/Soltu.DM.07G013900/Soltu.DM.10G003240/Soltu.DM.06G018150/Soltu.DM.03G035080/Soltu.DM.03G017660/Soltu.DM.08G007450/Soltu.DM.04G009170/Soltu.DM.06G013150/Soltu.DM.10G003920	
62	GO:0009250	glucan biosynthetic process	75/12756	0.001305196	0.062706876	0.059222165	Soltu.DM.09G018910/Soltu.DM.01G040570/Soltu.DM.04G027320/Soltu.DM.12G007610/Soltu.DM.08G029290/Soltu.DM.07G000930/Soltu.DM.12G008450/Soltu.DM.02G015410/Soltu.DM.02G024820/Soltu.DM.09G031790/Soltu.DM.04G022240/Soltu.DM.06G009750/Soltu.DM.05G009320/Soltu.DM.01G051470/Soltu.DM.09G007590/Soltu.DM.11G008240	16
63	GO:0009808	lignin metabolic process	82/12756	0.001307201	0.062706876	0.059222165	Soltu.DM.06G032850/Soltu.DM.06G032860/Soltu.DM.06G024250/Soltu.DM.03G031830/Soltu.DM.11G002020/Soltu.DM.07G028550/Soltu.DM.09G000560/Soltu.DM.10G029960/Soltu.DM.10G000640/Soltu.DM.10G019900/Soltu.DM.02G019030/Soltu.DM.03G021440/Soltu.DM.09G000850/Soltu.DM.02G024380/Soltu.DM.03G002800/Soltu.DM.11G002250/Soltu.DM.11G002650	17
64	GO:0016042	lipid catabolic process	149/12756	0.001313488	0.062706876	0.059222165	Soltu.DM.07G022720/Soltu.DM.08G024620/Soltu.DM.02G034460/Soltu.DM.07G022710/Soltu.DM.09G018310/Soltu.DM.01G033530/Soltu.DM.02G018520/Soltu.DM.08G020150/Soltu.DM.02G016290/Soltu.DM.01G038470/Soltu.DM.09G028490/Soltu.DM.07G013940/Soltu.DM.07G022700/Soltu.DM.07G013900/Soltu.DM.10G003240/Soltu.DM.06G018150/Soltu.DM.05G012150/Soltu.DM.02G016300/Soltu.DM.06G034310/Soltu.DM.01G005590/Soltu.DM.02G016770/Soltu.DM.03G024130/Soltu.DM.02G016380/Soltu.DM.02G016780/Soltu.DM.10G003920/Soltu.DM.02G019940	26
65	GO:0009642	response to light intensity	157/12756	0.001340618	0.062838443	0.05934642	Soltu.DM.02G025590/Soltu.DM.04G025250/Soltu.DM.07G011880/Soltu.DM.01G034240/Soltu.DM.11G001010/Soltu.DM.05G026160/Soltu.DM.07G003530/Soltu.DM.01G034250/Soltu.DM.03G037120/Soltu.DM.07G003550/Soltu.DM.07G028550/Soltu.DM.07G012130/Soltu.DM.10G000640/Soltu.DM.02G025970/Soltu.DM.01G008290/Soltu.DM.09G025070/Soltu.DM.04G007430/Soltu.DM.03G021360/Soltu.DM.02G030630/Soltu.DM.08G011890/Soltu.DM.01G032120/Soltu.DM.02G032340/Soltu.DM.07G000550/Soltu.DM.04G037460/Soltu.DM.07G024910/Soltu.DM.01G024940/Soltu.DM.02G013580	27
66	GO:0071365	cellular response to auxin stimulus	97/12756	0.001430438	0.065251572	0.061625449	Soltu.DM.02G025590/Soltu.DM.09G002620/Soltu.DM.06G026960/Soltu.DM.03G035710/Soltu.DM.10G026390/Soltu.DM.12G022190/Soltu.DM.11G011180/Soltu.DM.12G023260/Soltu.DM.06G018130/Soltu.DM.09G020190/Soltu.DM.02G004510/Soltu.DM.11G011390/Soltu.DM.04G029270/Soltu.DM.01G040670/Soltu.DM.02G032660/Soltu.DM.04G002510/Soltu.DM.02G027780/Soltu.DM.04G021540/Soltu.DM.06G034230	19
67	GO:0042743	hydrogen peroxide metabolic process	25/12756	0.001442723	0.065251572	0.061625449	Soltu.DM.02G022700/Soltu.DM.09G003770/Soltu.DM.08G028440/Soltu.DM.02G023590/Soltu.DM.08G011330/Soltu.DM.02G023580/Soltu.DM.07G022590/Soltu.DM.07G022640	8
68	GO:0010268	brassinosteroid homeostasis	43/12756	0.001575635	0.069711057	0.065837114	Soltu.DM.08G020150/Soltu.DM.03G017780/Soltu.DM.01G030110/Soltu.DM.05G015440/Soltu.DM.06G004460/Soltu.DM.03G017800/Soltu.DM.02G030630/Soltu.DM.12G024720/Soltu.DM.10G003570/Soltu.DM.08G027080/Soltu.DM.10G003550	11
69	GO:0030244	cellulose biosynthetic process	31/12756	0.001595404	0.069711057	0.065837114	Soltu.DM.04G027320/Soltu.DM.12G007610/Soltu.DM.07G000930/Soltu.DM.02G015410/Soltu.DM.09G031790/Soltu.DM.04G022240/Soltu.DM.06G009750/Soltu.DM.05G009320/Soltu.DM.09G007590	9
70	GO:0016131	brassinosteroid metabolic process	44/12756	0.001926519	0.082776103	0.078176116	Soltu.DM.08G020150/Soltu.DM.03G017780/Soltu.DM.01G030110/Soltu.DM.05G015440/Soltu.DM.06G004460/Soltu.DM.03G017800/Soltu.DM.02G030630/Soltu.DM.12G024720/Soltu.DM.10G003570/Soltu.DM.08G027080/Soltu.DM.10G003550	11
71	GO:0015849	organic acid transport	170/12756	0.002148514	0.087918555	0.083032795	Soltu.DM.05G003990/Soltu.DM.09G002620/Soltu.DM.04G031760/Soltu.DM.09G028710/Soltu.DM.03G011160/Soltu.DM.03G003280/Soltu.DM.01G008290/Soltu.DM.12G006380/Soltu.DM.06G005370/Soltu.DM.11G000570/Soltu.DM.02G030410/Soltu.DM.11G011180/Soltu.DM.05G022850/Soltu.DM.10G022360/Soltu.DM.06G015630/Soltu.DM.10G028240/Soltu.DM.08G003390/Soltu.DM.01G034820/Soltu.DM.01G001450/Soltu.DM.06G018560/Soltu.DM.06G017010/Soltu.DM.04G034490/Soltu.DM.03G024040/Soltu.DM.11G011430/Soltu.DM.05G021160/Soltu.DM.02G019940/Soltu.DM.11G004080/Soltu.DM.11G021200	28
72	GO:0046942	carboxylic acid transport	170/12756	0.002148514	0.087918555	0.083032795	Soltu.DM.05G003990/Soltu.DM.09G002620/Soltu.DM.04G031760/Soltu.DM.09G028710/Soltu.DM.03G011160/Soltu.DM.03G003280/Soltu.DM.01G008290/Soltu.DM.12G006380/Soltu.DM.06G005370/Soltu.DM.11G000570/Soltu.DM.02G030410/Soltu.DM.11G011180/Soltu.DM.05G022850/Soltu.DM.10G022360/Soltu.DM.06G015630/Soltu.DM.10G028240/Soltu.DM.08G003390/Soltu.DM.01G034820/Soltu.DM.01G001450/Soltu.DM.06G018560/Soltu.DM.06G017010/Soltu.DM.04G034490/Soltu.DM.03G024040/Soltu.DM.11G011430/Soltu.DM.05G021160/Soltu.DM.02G019940/Soltu.DM.11G004080/Soltu.DM.11G021200	28
73	GO:0009635	response to herbicide	21/12756	0.002196385	0.088473144	0.083556564	Soltu.DM.07G022460/Soltu.DM.07G022500/Soltu.DM.07G022530/Soltu.DM.07G022490/Soltu.DM.10G003300/Soltu.DM.07G022450/Soltu.DM.07G024910	7
74	GO:0097306	cellular response to alcohol	179/12756	0.002333254	0.091340017	0.086264121	Soltu.DM.09G019250/Soltu.DM.06G026960/Soltu.DM.07G017210/Soltu.DM.04G000490/Soltu.DM.09G031320/Soltu.DM.07G017190/Soltu.DM.07G017200/Soltu.DM.04G033590/Soltu.DM.03G017570/Soltu.DM.02G018520/Soltu.DM.07G017180/Soltu.DM.08G008380/Soltu.DM.06G034820/Soltu.DM.08G028440/Soltu.DM.01G000060/Soltu.DM.09G016850/Soltu.	29

						DM.06G003060/Soltu.DM.05G000860/Soltu.DM.03G021780/Soltu.DM.05G011970/Soltu.DM.08G011890/Soltu.DM.02G023840/Soltu.DM.07G026270/Soltu.DM.12G010960/Soltu.DM.09G031340/Soltu.DM.04G000670/Soltu.DM.03G016650/Soltu.DM.11G004950/Soltu.DM.01G024340	
75	GO:0045491	xylan metabolic process	45/12756	0.002338418	0.091340017	0.086264121	Soltu.DM.02G028360/Soltu.DM.10G001690/Soltu.DM.06G009650/Soltu.DM.06G010280/Soltu.DM.03G013120/Soltu.DM.07G025450/Soltu.DM.11G011390/Soltu.DM.09G006300/Soltu.DM.09G007590/Soltu.DM.11G008240/Soltu.DM.02G014140	11
76	GO:0006073	cellular glucan metabolic process	124/12756	0.002406654	0.092165519	0.087043749	Soltu.DM.09G027770/Soltu.DM.09G018910/Soltu.DM.01G040570/Soltu.DM.04G027320/Soltu.DM.05G006330/Soltu.DM.12G007610/Soltu.DM.01G022940/Soltu.DM.08G029290/Soltu.DM.07G000930/Soltu.DM.12G008450/Soltu.DM.02G015410/Soltu.DM.01G003570/Soltu.DM.12G007130/Soltu.DM.04G037250/Soltu.DM.02G024820/Soltu.DM.09G031790/Soltu.DM.04G022240/Soltu.DM.06G009750/Soltu.DM.05G009320/Soltu.DM.01G051470/Soltu.DM.09G007590/Soltu.DM.11G008240	22
77	GO:0010252	auxin homeostasis	39/12756	0.002498483	0.092165519	0.087043749	Soltu.DM.03G031830/Soltu.DM.03G035070/Soltu.DM.12G022190/Soltu.DM.12G023260/Soltu.DM.03G035080/Soltu.DM.02G032050/Soltu.DM.10G026500/Soltu.DM.07G026690/Soltu.DM.02G022410/Soltu.DM.04G002690	10
78	GO:0051592	response to calcium ion	27/12756	0.002502033	0.092165519	0.087043749	Soltu.DM.12G020370/Soltu.DM.10G026220/Soltu.DM.12G020350/Soltu.DM.06G024610/Soltu.DM.03G032350/Soltu.DM.04G022240/Soltu.DM.12G021010/Soltu.DM.12G020340	8
79	GO:0009404	toxin metabolic process	52/12756	0.002545413	0.092165519	0.087043749	Soltu.DM.07G022460/Soltu.DM.07G022500/Soltu.DM.12G022190/Soltu.DM.07G022530/Soltu.DM.06G018840/Soltu.DM.07G022490/Soltu.DM.10G003300/Soltu.DM.07G022450/Soltu.DM.09G001260/Soltu.DM.12G028960/Soltu.DM.01G028030/Soltu.DM.07G014750	12
80	GO:0001676	long-chain fatty acid metabolic process	33/12756	0.002574056	0.092165519	0.087043749	Soltu.DM.08G024620/Soltu.DM.04G034690/Soltu.DM.01G047750/Soltu.DM.02G017970/Soltu.DM.10G004300/Soltu.DM.03G033450/Soltu.DM.06G034310/Soltu.DM.02G031030/Soltu.DM.02G019940	9
81	GO:0051274	beta-glucan biosynthetic process	33/12756	0.002574056	0.092165519	0.087043749	Soltu.DM.04G027320/Soltu.DM.12G007610/Soltu.DM.07G000930/Soltu.DM.02G015410/Soltu.DM.09G031790/Soltu.DM.04G022240/Soltu.DM.06G009750/Soltu.DM.05G009320/Soltu.DM.09G007590	9
82	GO:0071555	cell wall organization	190/12756	0.002955992	0.102980384	0.097257617	Soltu.DM.04G027640/Soltu.DM.04G027650/Soltu.DM.01G044300/Soltu.DM.03G035710/Soltu.DM.02G023420/Soltu.DM.02G009140/Soltu.DM.10G001690/Soltu.DM.10G029580/Soltu.DM.02G031090/Soltu.DM.05G007640/Soltu.DM.09G021720/Soltu.DM.02G025970/Soltu.DM.07G000930/Soltu.DM.01G041220/Soltu.DM.02G031050/Soltu.DM.05G007630/Soltu.DM.04G029850/Soltu.DM.07G025450/Soltu.DM.05G026200/Soltu.DM.05G018830/Soltu.DM.04G022240/Soltu.DM.06G021870/Soltu.DM.02G029580/Soltu.DM.09G007590/Soltu.DM.08G012010/Soltu.DM.01G025270/Soltu.DM.05G018810/Soltu.DM.12G003880/Soltu.DM.01G040720/Soltu.DM.07G022640	30
83	GO:0009225	nucleotide-sugar metabolic process	40/12756	0.003057443	0.105094495	0.099254243	Soltu.DM.11G021140/Soltu.DM.02G025970/Soltu.DM.02G031050/Soltu.DM.04G020370/Soltu.DM.09G023470/Soltu.DM.06G030030/Soltu.DM.04G000320/Soltu.DM.05G001020/Soltu.DM.10G002320/Soltu.DM.03G027720	10
84	GO:0030243	cellulose metabolic process	34/12756	0.003215427	0.107818599	0.101826965	Soltu.DM.04G027320/Soltu.DM.12G007610/Soltu.DM.07G000930/Soltu.DM.02G015410/Soltu.DM.09G031790/Soltu.DM.04G022240/Soltu.DM.06G009750/Soltu.DM.05G009320/Soltu.DM.09G007590	9
85	GO:0010466	negative regulation of peptidase activity	28/12756	0.003220338	0.107818599	0.101826965	Soltu.DM.09G031320/Soltu.DM.03G020450/Soltu.DM.04G034360/Soltu.DM.04G034390/Soltu.DM.04G034380/Soltu.DM.04G003450/Soltu.DM.09G031340/Soltu.DM.04G034280	8
86	GO:0009646	response to absence of light	47/12756	0.00337558	0.111567258	0.105367305	Soltu.DM.02G025590/Soltu.DM.04G025250/Soltu.DM.11G001010/Soltu.DM.07G003530/Soltu.DM.07G003550/Soltu.DM.07G028550/Soltu.DM.10G000640/Soltu.DM.02G025970/Soltu.DM.02G030630/Soltu.DM.01G032120/Soltu.DM.01G024940	11
87	GO:0010119	regulation of stomatal movement	90/12756	0.0037022	0.120813556	0.114099774	Soltu.DM.07G017210/Soltu.DM.11G001010/Soltu.DM.07G017190/Soltu.DM.07G017200/Soltu.DM.01G035900/Soltu.DM.07G017180/Soltu.DM.07G012130/Soltu.DM.04G037130/Soltu.DM.12G024710/Soltu.DM.08G028440/Soltu.DM.05G024870/Soltu.DM.08G011890/Soltu.DM.12G026560/Soltu.DM.01G035910/Soltu.DM.11G004950/Soltu.DM.07G024910/Soltu.DM.07G022640	17
88	GO:0071482	cellular response to light stimulus	113/12756	0.003812614	0.122861497	0.116033907	Soltu.DM.09G031320/Soltu.DM.09G019220/Soltu.DM.09G025070/Soltu.DM.06G018130/Soltu.DM.06G028040/Soltu.DM.11G021810/Soltu.DM.03G022850/Soltu.DM.07G015980/Soltu.DM.02G001620/Soltu.DM.02G001630/Soltu.DM.08G011890/Soltu.DM.01G035240/Soltu.DM.06G021830/Soltu.DM.09G031340/Soltu.DM.07G028470/Soltu.DM.12G026560/Soltu.DM.10G005360/Soltu.DM.10G000040/Soltu.DM.02G011380/Soltu.DM.06G002140	20
89	GO:0042447	hormone catabolic process	23/1275	0.0039230	0.1233381	0.1164840	Soltu.DM.07G022720/Soltu.DM.06G028410/Soltu.DM.07G022710/Soltu.DM.04G011550/Soltu.DM.02G011120/Soltu.DM.07G022700/Soltu.DM.10G003240	7

			6	91	68	89		
90	GO:0071483	cellular response to blue light	23/12756	0.003923091	0.123338168	0.116484089	Soltu.DM.09G031320/Soltu.DM.09G025070/Soltu.DM.11G021810/Soltu.DM.03G022850/Soltu.DM.08G011890/Soltu.DM.06G021830/Soltu.DM.09G031340	7
91	GO:0016128	phytosteroid metabolic process	48/12756	0.004017122	0.124772768	0.117838966	Soltu.DM.08G020150/Soltu.DM.03G017780/Soltu.DM.01G030110/Soltu.DM.05G015440/Soltu.DM.06G004460/Soltu.DM.03G017800/Soltu.DM.02G030630/Soltu.DM.12G024720/Soltu.DM.10G003570/Soltu.DM.08G027080/Soltu.DM.10G003550	11
92	GO:0044042	glucan metabolic process	130/12756	0.004375665	0.134291242	0.126828485	Soltu.DM.09G027770/Soltu.DM.09G018910/Soltu.DM.01G040570/Soltu.DM.04G027320/Soltu.DM.05G006330/Soltu.DM.12G007610/Soltu.DM.01G022940/Soltu.DM.08G029290/Soltu.DM.07G000930/Soltu.DM.12G008450/Soltu.DM.02G015410/Soltu.DM.01G003570/Soltu.DM.12G007130/Soltu.DM.04G037250/Soltu.DM.02G024820/Soltu.DM.09G031790/Soltu.DM.04G022240/Soltu.DM.06G009750/Soltu.DM.05G009320/Soltu.DM.01G051470/Soltu.DM.09G007590/Soltu.DM.11G008240	22
93	GO:0046352	disaccharide catabolic process	13/12756	0.004797315	0.144325624	0.136305242	Soltu.DM.05G006330/Soltu.DM.01G018690/Soltu.DM.01G040550/Soltu.DM.04G037250/Soltu.DM.06G020260	5
94	GO:0010218	response to far red light	56/12756	0.004865819	0.144325624	0.136305242	Soltu.DM.02G025590/Soltu.DM.12G007510/Soltu.DM.04G027760/Soltu.DM.09G025070/Soltu.DM.06G018130/Soltu.DM.01G002850/Soltu.DM.03G022850/Soltu.DM.08G011110/Soltu.DM.02G013430/Soltu.DM.07G028470/Soltu.DM.03G021710/Soltu.DM.02G011380	12
95	GO:0051273	beta-glucan metabolic process	36/12756	0.00487057	0.144325624	0.136305242	Soltu.DM.04G027320/Soltu.DM.12G007610/Soltu.DM.07G000930/Soltu.DM.02G015410/Soltu.DM.09G031790/Soltu.DM.04G022240/Soltu.DM.06G009750/Soltu.DM.05G009320/Soltu.DM.09G007590	9
96	GO:0010030	positive regulation of seed germination	50/12756	0.005589879	0.163712039	0.154614326	Soltu.DM.05G007640/Soltu.DM.09G025070/Soltu.DM.05G007630/Soltu.DM.01G045040/Soltu.DM.01G035240/Soltu.DM.01G045030/Soltu.DM.03G017660/Soltu.DM.10G005360/Soltu.DM.01G020640/Soltu.DM.06G013150/Soltu.DM.01G045020	11
97	GO:0032350	regulation of hormone metabolic process	57/12756	0.005651812	0.163712039	0.154614326	Soltu.DM.06G009270/Soltu.DM.03G035070/Soltu.DM.08G013580/Soltu.DM.09G031790/Soltu.DM.03G035080/Soltu.DM.02G004510/Soltu.DM.05G022790/Soltu.DM.01G035240/Soltu.DM.11G018040/Soltu.DM.10G005360/Soltu.DM.07G024240/Soltu.DM.09G023400	12
98	GO:1901657	glycosyl compound metabolic process	175/12756	0.006372489	0.174638901	0.164933967	Soltu.DM.03G035710/Soltu.DM.02G027330/Soltu.DM.05G019830/Soltu.DM.01G042210/Soltu.DM.11G024450/Soltu.DM.05G007640/Soltu.DM.01G008310/Soltu.DM.03G037170/Soltu.DM.06G018090/Soltu.DM.07G020090/Soltu.DM.05G015440/Soltu.DM.02G020870/Soltu.DM.05G007630/Soltu.DM.09G020830/Soltu.DM.10G027910/Soltu.DM.12G003790/Soltu.DM.09G025040/Soltu.DM.12G002620/Soltu.DM.01G017170/Soltu.DM.03G002800/Soltu.DM.01G041980/Soltu.DM.04G005970/Soltu.DM.02G008560/Soltu.DM.02G009820/Soltu.DM.07G024690/Soltu.DM.02G008550/Soltu.DM.03G016750	27
99	GO:0046886	positive regulation of hormone biosynthetic process	9/12756	0.006510545	0.174638901	0.164933967	Soltu.DM.06G009270/Soltu.DM.05G022790/Soltu.DM.01G035240/Soltu.DM.10G005360	4
100	GO:0009313	oligosaccharide catabolic process	14/12756	0.006895076	0.174638901	0.164933967	Soltu.DM.05G006330/Soltu.DM.01G018690/Soltu.DM.01G040550/Soltu.DM.04G037250/Soltu.DM.06G020260	5
101	GO:0043090	amino acid import	14/12756	0.006895076	0.174638901	0.164933967	Soltu.DM.09G002620/Soltu.DM.04G031760/Soltu.DM.12G006380/Soltu.DM.11G011180/Soltu.DM.05G022850	5
102	GO:0043086	negative regulation of catalytic activity	135/12756	0.006905075	0.174638901	0.164933967	Soltu.DM.09G031320/Soltu.DM.03G020450/Soltu.DM.06G012620/Soltu.DM.04G034360/Soltu.DM.07G012130/Soltu.DM.03G015630/Soltu.DM.11G010230/Soltu.DM.03G015540/Soltu.DM.11G010220/Soltu.DM.04G034390/Soltu.DM.04G034380/Soltu.DM.06G028580/Soltu.DM.03G037430/Soltu.DM.04G003450/Soltu.DM.07G006430/Soltu.DM.04G038280/Soltu.DM.08G011890/Soltu.DM.11G008180/Soltu.DM.09G031340/Soltu.DM.04G034280/Soltu.DM.09G023660/Soltu.DM.02G013390	22
103	GO:0098869	cellular oxidant detoxification	119/12756	0.006911607	0.174638901	0.164933967	Soltu.DM.04G027640/Soltu.DM.04G027650/Soltu.DM.01G044300/Soltu.DM.02G031330/Soltu.DM.02G023420/Soltu.DM.09G021720/Soltu.DM.01G047440/Soltu.DM.02G022700/Soltu.DM.09G003770/Soltu.DM.02G024520/Soltu.DM.06G012170/Soltu.DM.02G019520/Soltu.DM.02G023590/Soltu.DM.05G018830/Soltu.DM.08G011330/Soltu.DM.02G023580/Soltu.DM.05G018810/Soltu.DM.12G003880/Soltu.DM.09G005310/Soltu.DM.03G013100	20
104	GO:0006598	polyamine catabolic process	5/12756	0.007045169	0.174638901	0.164933967	Soltu.DM.07G014310/Soltu.DM.02G020890/Soltu.DM.01G027080	3
105	GO:0010770	positive regulation of cell morphogenesis involved in	5/127	0.0070	0.1746	0.1649	Soltu.DM.12G020370/Soltu.DM.12G020350/Soltu.DM.12G020340	3

		differentiation	56	45169	38901	33967		
106	GO:0010771	negative regulation of cell morphogenesis involved in differentiation	5/12756	0.007045169	0.174638901	0.164933967	Soltu.DM.12G020370/Soltu.DM.12G020350/Soltu.DM.12G020340	3
107	GO:0030516	regulation of axon extension	5/12756	0.007045169	0.174638901	0.164933967	Soltu.DM.12G020370/Soltu.DM.12G020350/Soltu.DM.12G020340	3
108	GO:0032482	Rab protein signal transduction	5/12756	0.007045169	0.174638901	0.164933967	Soltu.DM.12G020370/Soltu.DM.12G020350/Soltu.DM.12G020340	3
109	GO:0050665	hydrogen peroxide biosynthetic process	5/12756	0.007045169	0.174638901	0.164933967	Soltu.DM.08G028440/Soltu.DM.07G022590/Soltu.DM.07G022640	3
110	GO:0050771	negative regulation of axonogenesis	5/12756	0.007045169	0.174638901	0.164933967	Soltu.DM.12G020370/Soltu.DM.12G020350/Soltu.DM.12G020340	3
111	GO:0090059	protoxylem development	5/12756	0.007045169	0.174638901	0.164933967	Soltu.DM.09G024260/Soltu.DM.11G011390/Soltu.DM.09G024270	3
112	GO:0090315	negative regulation of protein targeting to membrane	5/12756	0.007045169	0.174638901	0.164933967	Soltu.DM.12G020370/Soltu.DM.12G020350/Soltu.DM.12G020340	3
113	GO:0071462	cellular response to water stimulus	52/12756	0.007611307	0.186875711	0.176490796	Soltu.DM.01G040570/Soltu.DM.07G022460/Soltu.DM.07G022500/Soltu.DM.08G030020/Soltu.DM.07G022530/Soltu.DM.07G022490/Soltu.DM.10G003300/Soltu.DM.07G025290/Soltu.DM.07G022450/Soltu.DM.01G035240/Soltu.DM.10G005360	11
114	GO:2000762	regulation of phenylpropanoid metabolic process	20/12756	0.008090151	0.196758567	0.185824412	Soltu.DM.03G035710/Soltu.DM.07G003530/Soltu.DM.07G003550/Soltu.DM.03G034960/Soltu.DM.02G019030/Soltu.DM.10G017480	6
115	GO:0009226	nucleotide-sugar biosynthetic process	26/12756	0.008217495	0.197987869	0.186985399	Soltu.DM.11G021140/Soltu.DM.02G025970/Soltu.DM.02G031050/Soltu.DM.09G023470/Soltu.DM.06G030030/Soltu.DM.10G002320/Soltu.DM.03G027720	7
116	GO:0046885	regulation of hormone biosynthetic process	46/12756	0.008805863	0.208480908	0.196895325	Soltu.DM.06G009270/Soltu.DM.03G035070/Soltu.DM.08G013580/Soltu.DM.03G035080/Soltu.DM.02G004510/Soltu.DM.05G022790/Soltu.DM.01G035240/Soltu.DM.11G018040/Soltu.DM.10G005360/Soltu.DM.07G024240	10
117	GO:2000030	regulation of response to red or far red light	53/12756	0.008814747	0.208480908	0.196895325	Soltu.DM.07G022460/Soltu.DM.10G026220/Soltu.DM.07G022500/Soltu.DM.07G022530/Soltu.DM.07G022490/Soltu.DM.10G003300/Soltu.DM.07G022450/Soltu.DM.02G013430/Soltu.DM.02G002480/Soltu.DM.01G024340/Soltu.DM.06G002140	11
118	GO:1990748	cellular detoxification	122/12756	0.009098347	0.213232165	0.201382547	Soltu.DM.04G027640/Soltu.DM.04G027650/Soltu.DM.01G044300/Soltu.DM.02G031330/Soltu.DM.02G023420/Soltu.DM.09G021720/Soltu.DM.01G047440/Soltu.DM.02G022700/Soltu.DM.09G003770/Soltu.DM.02G024520/Soltu.DM.06G012170/Soltu.DM.02G019520/Soltu.DM.02G023590/Soltu.DM.05G018830/Soltu.DM.08G011330/Soltu.DM.02G023580/Soltu.DM.05G018810/Soltu.DM.12G003880/Soltu.DM.09G005310/Soltu.DM.03G013100	20
119	GO:0046503	glycerolipid catabolic process	33/12756	0.009466086	0.218065767	0.205947539	Soltu.DM.02G034460/Soltu.DM.02G016290/Soltu.DM.02G016300/Soltu.DM.06G034310/Soltu.DM.02G016770/Soltu.DM.02G016380/Soltu.DM.02G016780/Soltu.DM.02G019940	8
120	GO:0009833	plant-type primary cell wall biogenesis	15/12756	0.009558352	0.218065767	0.205947539	Soltu.DM.04G027320/Soltu.DM.12G007610/Soltu.DM.07G000930/Soltu.DM.02G015410/Soltu.DM.04G022240	5
121	GO:0010411	xyloglucan metabolic process	15/12756	0.009558352	0.218065767	0.205947539	Soltu.DM.09G018910/Soltu.DM.01G022940/Soltu.DM.08G029290/Soltu.DM.12G008450/Soltu.DM.01G003570	5
122	GO:0006558	L-phenylalanine metabolic process	10/12756	0.010055972	0.223485314	0.211065914	Soltu.DM.03G035070/Soltu.DM.02G020220/Soltu.DM.03G035080/Soltu.DM.08G011890	4

123	GO:0032352	positive regulation of hormone metabolic process	10/12756	0.010055972	0.223485314	0.211065914	Soltu.DM.06G009270/Soltu.DM.05G022790/Soltu.DM.01G035240/Soltu.DM.10G005360	4
124	GO:1902221	erythrose 4-phosphate/phosphoenolpyruvate family amino acid metabolic process	10/12756	0.010055972	0.223485314	0.211065914	Soltu.DM.03G035070/Soltu.DM.02G020220/Soltu.DM.03G035080/Soltu.DM.08G011890	4
125	GO:0071478	cellular response to radiation	124/12756	0.010843557	0.231576996	0.218707931	Soltu.DM.09G031320/Soltu.DM.09G019220/Soltu.DM.09G025070/Soltu.DM.06G018130/Soltu.DM.06G028040/Soltu.DM.11G021810/Soltu.DM.03G022850/Soltu.DM.07G015980/Soltu.DM.02G001620/Soltu.DM.02G001630/Soltu.DM.08G011890/Soltu.DM.01G035240/Soltu.DM.06G021830/Soltu.DM.09G031340/Soltu.DM.07G028470/Soltu.DM.12G026560/Soltu.DM.10G005360/Soltu.DM.10G000040/Soltu.DM.02G011380/Soltu.DM.06G002140	20
126	GO:0007623	circadian rhythm	108/12756	0.010946459	0.231576996	0.218707931	Soltu.DM.02G025590/Soltu.DM.06G026960/Soltu.DM.06G024250/Soltu.DM.07G026780/Soltu.DM.12G007510/Soltu.DM.01G018690/Soltu.DM.03G027640/Soltu.DM.03G017570/Soltu.DM.05G023990/Soltu.DM.10G027680/Soltu.DM.04G027760/Soltu.DM.10G016030/Soltu.DM.02G004510/Soltu.DM.12G002630/Soltu.DM.10G000040/Soltu.DM.10G027810/Soltu.DM.04G033440/Soltu.DM.02G013580	18
127	GO:0048511	rhythmic process	108/12756	0.010946459	0.231576996	0.218707931	Soltu.DM.02G025590/Soltu.DM.06G026960/Soltu.DM.06G024250/Soltu.DM.07G026780/Soltu.DM.12G007510/Soltu.DM.01G018690/Soltu.DM.03G027640/Soltu.DM.03G017570/Soltu.DM.05G023990/Soltu.DM.10G027680/Soltu.DM.04G027760/Soltu.DM.10G016030/Soltu.DM.02G004510/Soltu.DM.12G002630/Soltu.DM.10G000040/Soltu.DM.10G027810/Soltu.DM.04G033440/Soltu.DM.02G013580	18
128	GO:0052547	regulation of peptidase activity	34/12756	0.011389359	0.231576996	0.218707931	Soltu.DM.09G031320/Soltu.DM.03G020450/Soltu.DM.04G034360/Soltu.DM.04G034390/Soltu.DM.04G034380/Soltu.DM.04G003450/Soltu.DM.09G031340/Soltu.DM.04G034280	8
129	GO:1903825	organic acid transmembrane transport	141/12756	0.011404585	0.231576996	0.218707931	Soltu.DM.05G003990/Soltu.DM.09G002620/Soltu.DM.04G031760/Soltu.DM.03G011160/Soltu.DM.03G003280/Soltu.DM.01G008290/Soltu.DM.12G006380/Soltu.DM.11G000570/Soltu.DM.02G030410/Soltu.DM.11G011180/Soltu.DM.05G022850/Soltu.DM.06G015630/Soltu.DM.10G028240/Soltu.DM.08G003390/Soltu.DM.01G001450/Soltu.DM.06G018560/Soltu.DM.06G017010/Soltu.DM.04G034490/Soltu.DM.03G024040/Soltu.DM.05G021160/Soltu.DM.11G004080/Soltu.DM.11G021200	22
130	GO:1905039	carboxylic acid transmembrane transport	141/12756	0.011404585	0.231576996	0.218707931	Soltu.DM.05G003990/Soltu.DM.09G002620/Soltu.DM.04G031760/Soltu.DM.03G011160/Soltu.DM.03G003280/Soltu.DM.01G008290/Soltu.DM.12G006380/Soltu.DM.11G000570/Soltu.DM.02G030410/Soltu.DM.11G011180/Soltu.DM.05G022850/Soltu.DM.06G015630/Soltu.DM.10G028240/Soltu.DM.08G003390/Soltu.DM.01G001450/Soltu.DM.06G018560/Soltu.DM.06G017010/Soltu.DM.04G034490/Soltu.DM.03G024040/Soltu.DM.05G021160/Soltu.DM.11G004080/Soltu.DM.11G021200	22
131	GO:0000160	phosphorelay signal transduction system	85/12756	0.011614326	0.231576996	0.218707931	Soltu.DM.01G006210/Soltu.DM.03G027640/Soltu.DM.07G028550/Soltu.DM.10G000640/Soltu.DM.10G027680/Soltu.DM.07G020090/Soltu.DM.08G028440/Soltu.DM.05G024870/Soltu.DM.01G002310/Soltu.DM.09G026500/Soltu.DM.02G017280/Soltu.DM.06G011930/Soltu.DM.10G027810/Soltu.DM.06G011620/Soltu.DM.07G022640	15
132	GO:0016999	antibiotic metabolic process	85/12756	0.011614326	0.231576996	0.218707931	Soltu.DM.06G028410/Soltu.DM.05G007640/Soltu.DM.02G022700/Soltu.DM.09G003770/Soltu.DM.07G017750/Soltu.DM.08G028440/Soltu.DM.05G007630/Soltu.DM.02G023590/Soltu.DM.08G011330/Soltu.DM.02G023580/Soltu.DM.07G022590/Soltu.DM.02G008560/Soltu.DM.09G023400/Soltu.DM.02G008550/Soltu.DM.07G022640	15
133	GO:1901421	positive regulation of response to alcohol	55/12756	0.011657812	0.231576996	0.218707931	Soltu.DM.09G018310/Soltu.DM.07G017210/Soltu.DM.07G017190/Soltu.DM.07G017200/Soltu.DM.04G033590/Soltu.DM.07G017180/Soltu.DM.09G028490/Soltu.DM.04G037130/Soltu.DM.08G011890/Soltu.DM.09G026500/Soltu.DM.02G026820	11
134	GO:1905959	positive regulation of cellular response to alcohol	55/12756	0.011657812	0.231576996	0.218707931	Soltu.DM.09G018310/Soltu.DM.07G017210/Soltu.DM.07G017190/Soltu.DM.07G017200/Soltu.DM.04G033590/Soltu.DM.07G017180/Soltu.DM.09G028490/Soltu.DM.04G037130/Soltu.DM.08G011890/Soltu.DM.09G026500/Soltu.DM.02G026820	11
135	GO:0008202	steroid metabolic process	101/12756	0.011982305	0.231576996	0.218707931	Soltu.DM.04G034690/Soltu.DM.01G042210/Soltu.DM.08G020150/Soltu.DM.11G023180/Soltu.DM.03G017780/Soltu.DM.01G030110/Soltu.DM.05G015440/Soltu.DM.06G004460/Soltu.DM.07G017000/Soltu.DM.03G017800/Soltu.DM.02G030630/Soltu.DM.12G024720/Soltu.DM.01G048780/Soltu.DM.02G007460/Soltu.DM.10G003570/Soltu.DM.08G027080/Soltu.DM.10G003550	17
136	GO:0051224	negative regulation of protein transport	16/12756	0.012851746	0.231576996	0.218707931	Soltu.DM.12G020370/Soltu.DM.12G020350/Soltu.DM.08G027150/Soltu.DM.09G002090/Soltu.DM.12G020340	5
137	GO:1904950	negative regulation of establishment of protein localization	16/12756	0.012851746	0.231576996	0.218707931	Soltu.DM.12G020370/Soltu.DM.12G020350/Soltu.DM.08G027150/Soltu.DM.09G002090/Soltu.DM.12G020340	5
138	GO:0001682	tRNA 5'-leader removal	6/12756	0.0131149	0.2315769	0.2187079	Soltu.DM.12G016740/Soltu.DM.11G004920/Soltu.DM.06G029830	3

				11	96	31		
139	GO:0009099	valine biosynthetic process	6/12756	0.013114911	0.231576996	0.218707931	Soltu.DM.03G005810/Soltu.DM.07G023080/Soltu.DM.11G003850	3
140	GO:0010366	negative regulation of ethylene biosynthetic process	6/12756	0.013114911	0.231576996	0.218707931	Soltu.DM.03G035070/Soltu.DM.03G035080/Soltu.DM.11G018040	3
141	GO:0015824	proline transport	6/12756	0.013114911	0.231576996	0.218707931	Soltu.DM.04G031760/Soltu.DM.12G006380/Soltu.DM.05G022850	3
142	GO:0031336	negative regulation of sulfur amino acid metabolic process	6/12756	0.013114911	0.231576996	0.218707931	Soltu.DM.03G035070/Soltu.DM.03G035080/Soltu.DM.11G018040	3
143	GO:0032387	negative regulation of intracellular transport	6/12756	0.013114911	0.231576996	0.218707931	Soltu.DM.12G020370/Soltu.DM.12G020350/Soltu.DM.12G020340	3
144	GO:0033239	negative regulation of amine metabolic process	6/12756	0.013114911	0.231576996	0.218707931	Soltu.DM.03G035070/Soltu.DM.03G035080/Soltu.DM.11G018040	3
145	GO:0035524	proline transmembrane transport	6/12756	0.013114911	0.231576996	0.218707931	Soltu.DM.04G031760/Soltu.DM.12G006380/Soltu.DM.05G022850	3
146	GO:0040009	regulation of growth rate	6/12756	0.013114911	0.231576996	0.218707931	Soltu.DM.04G006870/Soltu.DM.02G022410/Soltu.DM.06G001230	3
147	GO:0045763	negative regulation of cellular amino acid metabolic process	6/12756	0.013114911	0.231576996	0.218707931	Soltu.DM.03G035070/Soltu.DM.03G035080/Soltu.DM.11G018040	3
148	GO:0050770	regulation of axonogenesis	6/12756	0.013114911	0.231576996	0.218707931	Soltu.DM.12G020370/Soltu.DM.12G020350/Soltu.DM.12G020340	3
149	GO:0051175	negative regulation of sulfur metabolic process	6/12756	0.013114911	0.231576996	0.218707931	Soltu.DM.03G035070/Soltu.DM.03G035080/Soltu.DM.11G018040	3
150	GO:0090317	negative regulation of intracellular protein transport	6/12756	0.013114911	0.231576996	0.218707931	Soltu.DM.12G020370/Soltu.DM.12G020350/Soltu.DM.12G020340	3
151	GO:1900909	negative regulation of olefin metabolic process	6/12756	0.013114911	0.231576996	0.218707931	Soltu.DM.03G035070/Soltu.DM.03G035080/Soltu.DM.11G018040	3
152	GO:1900912	negative regulation of olefin biosynthetic process	6/12756	0.013114911	0.231576996	0.218707931	Soltu.DM.03G035070/Soltu.DM.03G035080/Soltu.DM.11G018040	3
153	GO:1905476	negative regulation of protein localization to membrane	6/12756	0.013114911	0.231576996	0.218707931	Soltu.DM.12G020370/Soltu.DM.12G020350/Soltu.DM.12G020340	3
154	GO:0043455	regulation of secondary metabolic process	56/12756	0.013318077	0.233564651	0.220585128	Soltu.DM.03G035710/Soltu.DM.07G003530/Soltu.DM.07G003550/Soltu.DM.03G034960/Soltu.DM.12G022190/Soltu.DM.02G019030/Soltu.DM.02G020950/Soltu.DM.10G017480/Soltu.DM.08G013580/Soltu.DM.01G035240/Soltu.DM.10G005360	11
155	GO:0045861	negative regulation of proteolysis	35/12756	0.013585864	0.23665107	0.223500031	Soltu.DM.09G031320/Soltu.DM.03G020450/Soltu.DM.04G034360/Soltu.DM.04G034390/Soltu.DM.04G034380/Soltu.DM.04G003450/Soltu.DM.09G031340/Soltu.DM.04G034280	8
156	GO:0009685	gibberellin metabolic process	87/12	0.0142	0.2452	0.2315	Soltu.DM.07G022720/Soltu.DM.07G022710/Soltu.DM.07G020920/Soltu.DM.02G011120/Soltu.DM.10G024780/Soltu.DM.07G022700/Soltu.DM.06G012790/Soltu.DM.08G013420/Sol	15

			756	73099	24562	97081	tu.DM.06G004460/Soltu.DM.10G003240/Soltu.DM.02G019740/Soltu.DM.10G003570/Soltu.DM.10G022070/Soltu.DM.06G023440/Soltu.DM.10G003550	
157	GO:0016103	diterpenoid catabolic process	11/12756	0.014648791	0.245224562	0.231597081	Soltu.DM.07G022720/Soltu.DM.07G022710/Soltu.DM.07G022700/Soltu.DM.10G003240	4
158	GO:0043649	dicarboxylic acid catabolic process	11/12756	0.014648791	0.245224562	0.231597081	Soltu.DM.01G033530/Soltu.DM.12G024030/Soltu.DM.03G017660/Soltu.DM.06G013150	4
159	GO:0045487	gibberellin catabolic process	11/12756	0.014648791	0.245224562	0.231597081	Soltu.DM.07G022720/Soltu.DM.07G022710/Soltu.DM.07G022700/Soltu.DM.10G003240	4
160	GO:0080144	cellular amino acid homeostasis	11/12756	0.014648791	0.245224562	0.231597081	Soltu.DM.09G002620/Soltu.DM.11G011180/Soltu.DM.10G000600/Soltu.DM.10G025890	4
161	GO:1905156	negative regulation of photosynthesis	11/12756	0.014648791	0.245224562	0.231597081	Soltu.DM.07G012130/Soltu.DM.01G024860/Soltu.DM.07G000550/Soltu.DM.04G037460	4
162	GO:0033037	polysaccharide localization	29/12756	0.015242707	0.2518955	0.237897306	Soltu.DM.03G035710/Soltu.DM.05G007640/Soltu.DM.02G020800/Soltu.DM.08G028440/Soltu.DM.03G019120/Soltu.DM.05G007630/Soltu.DM.07G022640	7
163	GO:0052545	callose localization	29/12756	0.015242707	0.2518955	0.237897306	Soltu.DM.03G035710/Soltu.DM.05G007640/Soltu.DM.02G020800/Soltu.DM.08G028440/Soltu.DM.03G019120/Soltu.DM.05G007630/Soltu.DM.07G022640	7
164	GO:0072593	reactive oxygen species metabolic process	65/12756	0.016141346	0.263817942	0.2491572	Soltu.DM.02G022700/Soltu.DM.09G003770/Soltu.DM.08G028440/Soltu.DM.06G012170/Soltu.DM.10G004300/Soltu.DM.02G023590/Soltu.DM.08G011890/Soltu.DM.08G011330/Soltu.DM.02G023580/Soltu.DM.03G028360/Soltu.DM.07G022590/Soltu.DM.07G022640	12
165	GO:0006749	glutathione metabolic process	43/12756	0.016168827	0.263817942	0.2491572	Soltu.DM.07G022460/Soltu.DM.07G022500/Soltu.DM.07G022530/Soltu.DM.07G022490/Soltu.DM.10G003300/Soltu.DM.07G022450/Soltu.DM.09G001260/Soltu.DM.12G028960/Soltu.DM.01G028030	9
166	GO:0017001	antibiotic catabolic process	23/12756	0.016546884	0.264619216	0.249913946	Soltu.DM.06G028410/Soltu.DM.02G022700/Soltu.DM.09G003770/Soltu.DM.02G023590/Soltu.DM.08G011330/Soltu.DM.02G023580	6
167	GO:0046340	diacylglycerol catabolic process	23/12756	0.016546884	0.264619216	0.249913946	Soltu.DM.02G016290/Soltu.DM.02G016300/Soltu.DM.06G034310/Soltu.DM.02G016770/Soltu.DM.02G016380/Soltu.DM.02G016780	6
168	GO:0009311	oligosaccharide metabolic process	73/12756	0.01680187	0.264619216	0.249913946	Soltu.DM.04G012960/Soltu.DM.02G015310/Soltu.DM.05G006330/Soltu.DM.01G018690/Soltu.DM.01G040550/Soltu.DM.07G001730/Soltu.DM.09G022610/Soltu.DM.04G037250/Soltu.DM.12G024030/Soltu.DM.05G027000/Soltu.DM.05G008060/Soltu.DM.06G020260/Soltu.DM.07G014750	13
169	GO:0006836	neurotransmitter transport	17/12756	0.016833806	0.264619216	0.249913946	Soltu.DM.05G003990/Soltu.DM.04G031760/Soltu.DM.12G006380/Soltu.DM.11G026460/Soltu.DM.11G021200	5
170	GO:0015867	ATP transport	17/12756	0.016833806	0.264619216	0.249913946	Soltu.DM.01G038470/Soltu.DM.06G024610/Soltu.DM.03G032350/Soltu.DM.03G034530/Soltu.DM.06G025410	5
171	GO:1902456	regulation of stomatal opening	17/12756	0.016833806	0.264619216	0.249913946	Soltu.DM.07G017210/Soltu.DM.07G017190/Soltu.DM.07G017200/Soltu.DM.07G017180/Soltu.DM.07G012130	5
172	GO:0005984	disaccharide metabolic process	58/12756	0.017167838	0.268234466	0.253328292	Soltu.DM.04G012960/Soltu.DM.02G015310/Soltu.DM.05G006330/Soltu.DM.01G018690/Soltu.DM.01G040550/Soltu.DM.07G001730/Soltu.DM.04G037250/Soltu.DM.12G024030/Soltu.DM.05G008060/Soltu.DM.06G020260/Soltu.DM.07G014750	11
173	GO:0030522	intracellular receptor signaling pathway	30/12756	0.018321248	0.284531188	0.268719382	Soltu.DM.09G031320/Soltu.DM.12G025260/Soltu.DM.11G021810/Soltu.DM.04G038280/Soltu.DM.08G011890/Soltu.DM.06G021830/Soltu.DM.09G031340	7
17	GO:00	S-glycoside metabolic process	82/	0.0	0.2	0.2	Soltu.DM.03G035710/Soltu.DM.02G027330/Soltu.DM.05G019830/Soltu.DM.11G024450/S	14

4	16143		12756	19014242	88855385	72803277	oltu.DM.05G007640/Soltu.DM.06G018090/Soltu.DM.07G020090/Soltu.DM.02G020870/Soltu.DM.05G007630/Soltu.DM.09G020830/Soltu.DM.01G041980/Soltu.DM.04G005970/Soltu.DM.02G009820/Soltu.DM.03G016750	
175	GO:0019757	glycosinolate metabolic process	82/12756	0.019014242	0.288855385	0.272803277	Soltu.DM.03G035710/Soltu.DM.02G027330/Soltu.DM.05G019830/Soltu.DM.11G024450/Soltu.DM.05G007640/Soltu.DM.06G018090/Soltu.DM.07G020090/Soltu.DM.02G020870/Soltu.DM.05G007630/Soltu.DM.09G020830/Soltu.DM.01G041980/Soltu.DM.04G005970/Soltu.DM.02G009820/Soltu.DM.03G016750	14
176	GO:0019760	glucosinolate metabolic process	82/12756	0.019014242	0.288855385	0.272803277	Soltu.DM.03G035710/Soltu.DM.02G027330/Soltu.DM.05G019830/Soltu.DM.11G024450/Soltu.DM.05G007640/Soltu.DM.06G018090/Soltu.DM.07G020090/Soltu.DM.02G020870/Soltu.DM.05G007630/Soltu.DM.09G020830/Soltu.DM.01G041980/Soltu.DM.04G005970/Soltu.DM.02G009820/Soltu.DM.03G016750	14
177	GO:0016101	diterpenoid metabolic process	98/12756	0.019047873	0.288855385	0.272803277	Soltu.DM.07G022720/Soltu.DM.07G022710/Soltu.DM.07G020920/Soltu.DM.02G011120/Soltu.DM.03G018850/Soltu.DM.10G024780/Soltu.DM.07G022700/Soltu.DM.06G012790/Soltu.DM.08G013420/Soltu.DM.06G004460/Soltu.DM.10G003240/Soltu.DM.02G019740/Soltu.DM.10G003570/Soltu.DM.10G022070/Soltu.DM.06G023440/Soltu.DM.10G003550	16
178	GO:0015804	neutral amino acid transport	24/12756	0.020362872	0.301881789	0.285105785	Soltu.DM.05G003990/Soltu.DM.04G031760/Soltu.DM.03G003280/Soltu.DM.12G006380/Soltu.DM.05G022850/Soltu.DM.06G015630	6
179	GO:1900057	positive regulation of leaf senescence	24/12756	0.020362872	0.301881789	0.285105785	Soltu.DM.07G017210/Soltu.DM.07G017190/Soltu.DM.07G017200/Soltu.DM.07G017180/Soltu.DM.04G033430/Soltu.DM.06G017300	6
180	GO:1905623	positive regulation of leaf development	24/12756	0.020362872	0.301881789	0.285105785	Soltu.DM.07G017210/Soltu.DM.07G017190/Soltu.DM.07G017200/Soltu.DM.07G017180/Soltu.DM.04G033430/Soltu.DM.06G017300	6
181	GO:1901141	regulation of lignin biosynthetic process	12/12756	0.020375264	0.301881789	0.285105785	Soltu.DM.03G035710/Soltu.DM.07G003530/Soltu.DM.07G003550/Soltu.DM.02G019030	4
182	GO:0015808	L-alanine transport	7/12756	0.021370475	0.308950577	0.291781751	Soltu.DM.05G003990/Soltu.DM.04G031760/Soltu.DM.12G006380	3
183	GO:0032328	alanine transport	7/12756	0.021370475	0.308950577	0.291781751	Soltu.DM.05G003990/Soltu.DM.04G031760/Soltu.DM.12G006380	3
184	GO:0061387	regulation of extent of cell growth	7/12756	0.021370475	0.308950577	0.291781751	Soltu.DM.12G020370/Soltu.DM.12G020350/Soltu.DM.12G020340	3
185	GO:2000014	regulation of endosperm development	7/12756	0.021370475	0.308950577	0.291781751	Soltu.DM.11G002420/Soltu.DM.05G021010/Soltu.DM.01G024940	3
186	GO:0052542	defense response by callose deposition	18/12756	0.021555906	0.308950577	0.291781751	Soltu.DM.03G035710/Soltu.DM.05G007640/Soltu.DM.08G028440/Soltu.DM.05G007630/Soltu.DM.07G022640	5
187	GO:0015706	nitrate transmembrane transport	31/12756	0.021811096	0.308950577	0.291781751	Soltu.DM.02G010790/Soltu.DM.08G025260/Soltu.DM.10G010160/Soltu.DM.03G035250/Soltu.DM.03G027330/Soltu.DM.06G017770/Soltu.DM.03G035260	7
188	GO:0046339	diacylglycerol metabolic process	31/12756	0.021811096	0.308950577	0.291781751	Soltu.DM.02G016290/Soltu.DM.02G024810/Soltu.DM.02G016300/Soltu.DM.06G034310/Soltu.DM.02G016770/Soltu.DM.02G016380/Soltu.DM.02G016780	7
189	GO:1902025	nitrate import	31/12756	0.021811096	0.308950577	0.291781751	Soltu.DM.02G010790/Soltu.DM.08G025260/Soltu.DM.10G010160/Soltu.DM.03G035250/Soltu.DM.03G027330/Soltu.DM.06G017770/Soltu.DM.03G035260	7
190	GO:0009624	response to nematode	92/12756	0.022978522	0.323708337	0.305719401	Soltu.DM.04G031760/Soltu.DM.01G035900/Soltu.DM.04G023400/Soltu.DM.06G010750/Soltu.DM.05G025440/Soltu.DM.12G006380/Soltu.DM.02G032470/Soltu.DM.05G026690/Soltu.DM.01G025110/Soltu.DM.12G000550/Soltu.DM.09G004090/Soltu.DM.01G035910/Soltu.DM.02G014120/Soltu.DM.06G010530/Soltu.DM.10G024900	15
191	GO:0010150	leaf senescence	135/12756	0.025731588	0.360521808	0.340487094	Soltu.DM.06G028410/Soltu.DM.05G026160/Soltu.DM.03G037070/Soltu.DM.12G027330/Soltu.DM.06G022310/Soltu.DM.03G017570/Soltu.DM.10G010160/Soltu.DM.03G037170/Soltu.DM.02G018270/Soltu.DM.03G016080/Soltu.DM.02G014410/Soltu.DM.05G024870/Soltu.DM.12G003790/Soltu.DM.05G011970/Soltu.DM.11G022310/Soltu.DM.03G033450/Soltu.	20

							DM.01G007500/Soltu.DM.02G014440/Soltu.DM.07G014750/Soltu.DM.06G010530	
192	GO:0009812	flavonoid metabolic process	102/12756	0.026880795	0.3684623	0.347986321	Soltu.DM.06G028410/Soltu.DM.11G001010/Soltu.DM.01G024670/Soltu.DM.10G029960/Soltu.DM.04G001370/Soltu.DM.04G018070/Soltu.DM.03G021440/Soltu.DM.09G028560/Soltu.DM.03G019900/Soltu.DM.03G020570/Soltu.DM.09G025040/Soltu.DM.10G003940/Soltu.DM.03G018210/Soltu.DM.12G026560/Soltu.DM.08G026700/Soltu.DM.07G014750	16
193	GO:0006469	negative regulation of protein kinase activity	19/12756	0.027061421	0.3684623	0.347986321	Soltu.DM.07G012130/Soltu.DM.11G010230/Soltu.DM.11G010220/Soltu.DM.06G028580/Soltu.DM.04G038280	5
194	GO:0009785	blue light signaling pathway	19/12756	0.027061421	0.3684623	0.347986321	Soltu.DM.09G031320/Soltu.DM.11G021810/Soltu.DM.08G011890/Soltu.DM.06G021830/Soltu.DM.09G031340	5
195	GO:0033673	negative regulation of kinase activity	19/12756	0.027061421	0.3684623	0.347986321	Soltu.DM.07G012130/Soltu.DM.11G010230/Soltu.DM.11G010220/Soltu.DM.06G028580/Soltu.DM.04G038280	5
196	GO:0042168	heme metabolic process	13/12756	0.027298797	0.3684623	0.347986321	Soltu.DM.08G013640/Soltu.DM.04G031570/Soltu.DM.12G026560/Soltu.DM.06G002140	4
197	GO:0070413	trehalose metabolism in response to stress	13/12756	0.027298797	0.3684623	0.347986321	Soltu.DM.04G012960/Soltu.DM.02G015310/Soltu.DM.07G001730/Soltu.DM.05G008060	4
198	GO:2000652	regulation of secondary cell wall biogenesis	13/12756	0.027298797	0.3684623	0.347986321	Soltu.DM.07G028550/Soltu.DM.10G000640/Soltu.DM.11G011390/Soltu.DM.01G027520	4
199	GO:0009664	plant-type cell wall organization	136/12756	0.027618453	0.370826102	0.350218764	Soltu.DM.04G027640/Soltu.DM.04G027650/Soltu.DM.01G044300/Soltu.DM.02G023420/Soltu.DM.02G009140/Soltu.DM.10G029580/Soltu.DM.02G031090/Soltu.DM.09G021720/Soltu.DM.07G000930/Soltu.DM.02G031050/Soltu.DM.04G029850/Soltu.DM.05G018830/Soltu.DM.04G022240/Soltu.DM.06G021870/Soltu.DM.09G007590/Soltu.DM.08G012010/Soltu.DM.01G025270/Soltu.DM.05G018810/Soltu.DM.12G003880/Soltu.DM.01G040720	20
200	GO:0120254	olefinic compound metabolic process	86/12756	0.027761613	0.370826102	0.350218764	Soltu.DM.04G034690/Soltu.DM.08G020150/Soltu.DM.11G024450/Soltu.DM.07G013940/Soltu.DM.03G018850/Soltu.DM.11G009500/Soltu.DM.02G004480/Soltu.DM.06G029640/Soltu.DM.02G017970/Soltu.DM.07G013900/Soltu.DM.10G004300/Soltu.DM.06G034310/Soltu.DM.06G020450/Soltu.DM.02G019940	14
201	GO:0009743	response to carbohydrate	180/12756	0.028722902	0.381688874	0.360477876	Soltu.DM.06G026960/Soltu.DM.04G025250/Soltu.DM.03G035710/Soltu.DM.07G013360/Soltu.DM.01G006210/Soltu.DM.03G017570/Soltu.DM.05G007640/Soltu.DM.08G008380/Soltu.DM.02G025970/Soltu.DM.07G020920/Soltu.DM.10G024780/Soltu.DM.05G006190/Soltu.DM.12G025260/Soltu.DM.08G028440/Soltu.DM.05G007630/Soltu.DM.08G013420/Soltu.DM.04G024100/Soltu.DM.05G011970/Soltu.DM.08G011890/Soltu.DM.01G024860/Soltu.DM.02G002480/Soltu.DM.10G022070/Soltu.DM.12G029710/Soltu.DM.01G047090/Soltu.DM.07G022640	25
202	GO:0001666	response to hypoxia	103/12756	0.029168503	0.383130295	0.361839195	Soltu.DM.07G011880/Soltu.DM.07G013360/Soltu.DM.01G006210/Soltu.DM.07G020920/Soltu.DM.10G024780/Soltu.DM.10G019450/Soltu.DM.03G037290/Soltu.DM.08G013420/Soltu.DM.06G026400/Soltu.DM.01G051770/Soltu.DM.08G001690/Soltu.DM.04G005970/Soltu.DM.10G022070/Soltu.DM.11G013880/Soltu.DM.11G011650/Soltu.DM.11G011740	16
203	GO:0071489	cellular response to red or far red light	55/12756	0.029497937	0.383130295	0.361839195	Soltu.DM.09G025070/Soltu.DM.06G018130/Soltu.DM.07G015980/Soltu.DM.01G035240/Soltu.DM.06G021830/Soltu.DM.07G028470/Soltu.DM.10G005360/Soltu.DM.10G000040/Soltu.DM.02G011380/Soltu.DM.06G002140	10
204	GO:0010951	negative regulation of endopeptidase activity	26/12756	0.029689031	0.383130295	0.361839195	Soltu.DM.03G020450/Soltu.DM.04G034360/Soltu.DM.04G034390/Soltu.DM.04G034380/Soltu.DM.04G003450/Soltu.DM.04G034280	6
205	GO:0048582	positive regulation of post-embryonic development	120/12756	0.029691371	0.383130295	0.361839195	Soltu.DM.12G007510/Soltu.DM.05G007640/Soltu.DM.04G027760/Soltu.DM.09G025070/Soltu.DM.05G007630/Soltu.DM.06G019760/Soltu.DM.01G045040/Soltu.DM.01G040220/Soltu.DM.01G035240/Soltu.DM.01G045030/Soltu.DM.03G017660/Soltu.DM.10G005360/Soltu.DM.01G024340/Soltu.DM.01G020640/Soltu.DM.08G001470/Soltu.DM.06G013150/Soltu.DM.03G024000/Soltu.DM.01G045020	18
206	GO:0009637	response to blue light	95/12756	0.029832927	0.383130295	0.361839195	Soltu.DM.02G025590/Soltu.DM.12G007510/Soltu.DM.09G031320/Soltu.DM.05G019830/Soltu.DM.04G027760/Soltu.DM.09G025070/Soltu.DM.04G001110/Soltu.DM.11G021810/Soltu.DM.03G022850/Soltu.DM.05G006430/Soltu.DM.08G011890/Soltu.DM.02G013430/Soltu.DM.06G021830/Soltu.DM.09G031340/Soltu.DM.10G001460	15
207	GO:0009644	response to high light intensity	87/12756	0.030348451	0.383130295	0.361839195	Soltu.DM.07G011880/Soltu.DM.01G034240/Soltu.DM.05G026160/Soltu.DM.01G034250/Soltu.DM.03G037120/Soltu.DM.07G012130/Soltu.DM.09G025070/Soltu.DM.04G007430/Soltu.DM.03G021360/Soltu.DM.02G032340/Soltu.DM.07G000550/Soltu.DM.04G037460/Soltu.DM.07G024910/Soltu.DM.02G013580	14
20	GO:00	phloem or xylem histogenesis	11	0.0	0.3	0.3	Soltu.DM.09G024260/Soltu.DM.05G010240/Soltu.DM.02G019030/Soltu.DM.07G010930/S	17

8	10087		2/12756	30676639	83130295	61839195	oltu.DM.05G011970/Soltu.DM.09G020190/Soltu.DM.06G019150/Soltu.DM.01G047760/Soltu.DM.11G011390/Soltu.DM.01G007500/Soltu.DM.03G014570/Soltu.DM.12G030150/Soltu.DM.05G010260/Soltu.DM.08G012010/Soltu.DM.09G024270/Soltu.DM.03G014580/Soltu.DM.07G022640	
209	GO:0044092	negative regulation of molecular function	155/12756	0.031048743	0.383130295	0.361839195	Soltu.DM.09G031320/Soltu.DM.03G020450/Soltu.DM.06G012620/Soltu.DM.04G034360/Soltu.DM.07G012130/Soltu.DM.03G015630/Soltu.DM.11G010230/Soltu.DM.03G015540/Soltu.DM.11G010220/Soltu.DM.04G034390/Soltu.DM.04G034380/Soltu.DM.06G028580/Soltu.DM.03G037430/Soltu.DM.04G003450/Soltu.DM.07G006430/Soltu.DM.04G038280/Soltu.DM.08G011890/Soltu.DM.11G008180/Soltu.DM.09G031340/Soltu.DM.04G034280/Soltu.DM.09G023660/Soltu.DM.02G013390	22
210	GO:0006833	water transport	48/12756	0.031662799	0.383130295	0.361839195	Soltu.DM.10G029590/Soltu.DM.03G012810/Soltu.DM.06G006130/Soltu.DM.12G010360/Soltu.DM.06G018020/Soltu.DM.06G031120/Soltu.DM.05G026690/Soltu.DM.12G015670/Soltu.DM.08G012010	9
211	GO:0042044	fluid transport	48/12756	0.031662799	0.383130295	0.361839195	Soltu.DM.10G029590/Soltu.DM.03G012810/Soltu.DM.06G006130/Soltu.DM.12G010360/Soltu.DM.06G018020/Soltu.DM.06G031120/Soltu.DM.05G026690/Soltu.DM.12G015670/Soltu.DM.08G012010	9
212	GO:0009819	drought recovery	8/12756	0.031850359	0.383130295	0.361839195	Soltu.DM.05G026160/Soltu.DM.01G039760/Soltu.DM.01G000710	3
213	GO:0010205	photoinhibition	8/12756	0.031850359	0.383130295	0.361839195	Soltu.DM.07G012130/Soltu.DM.07G000550/Soltu.DM.04G037460	3
214	GO:0015825	L-serine transport	8/12756	0.031850359	0.383130295	0.361839195	Soltu.DM.04G031760/Soltu.DM.12G006380/Soltu.DM.06G015630	3
215	GO:0032329	serine transport	8/12756	0.031850359	0.383130295	0.361839195	Soltu.DM.04G031760/Soltu.DM.12G006380/Soltu.DM.06G015630	3
216	GO:0032351	negative regulation of hormone metabolic process	8/12756	0.031850359	0.383130295	0.361839195	Soltu.DM.03G035070/Soltu.DM.03G035080/Soltu.DM.11G018040	3
217	GO:0032353	negative regulation of hormone biosynthetic process	8/12756	0.031850359	0.383130295	0.361839195	Soltu.DM.03G035070/Soltu.DM.03G035080/Soltu.DM.11G018040	3
218	GO:0043155	negative regulation of photosynthesis, light reaction	8/12756	0.031850359	0.383130295	0.361839195	Soltu.DM.07G012130/Soltu.DM.07G000550/Soltu.DM.04G037460	3
219	GO:0051552	flavone metabolic process	8/12756	0.031850359	0.383130295	0.361839195	Soltu.DM.06G028410/Soltu.DM.01G024670/Soltu.DM.03G021440	3
220	GO:0051553	flavone biosynthetic process	8/12756	0.031850359	0.383130295	0.361839195	Soltu.DM.06G028410/Soltu.DM.01G024670/Soltu.DM.03G021440	3
221	GO:0090313	regulation of protein targeting to membrane	8/12756	0.031850359	0.383130295	0.361839195	Soltu.DM.12G020370/Soltu.DM.12G020350/Soltu.DM.12G020340	3
222	GO:0036293	response to decreased oxygen levels	121/12756	0.031952294	0.383130295	0.361839195	Soltu.DM.07G011880/Soltu.DM.07G013360/Soltu.DM.01G006210/Soltu.DM.07G020920/Soltu.DM.10G024780/Soltu.DM.10G019450/Soltu.DM.03G037290/Soltu.DM.08G013420/Soltu.DM.06G026400/Soltu.DM.03G027730/Soltu.DM.01G051770/Soltu.DM.01G024860/Soltu.DM.08G001690/Soltu.DM.04G005970/Soltu.DM.10G022070/Soltu.DM.11G013880/Soltu.DM.11G011650/Soltu.DM.11G011740	18
223	GO:0009641	shade avoidance	20/12756	0.033385178	0.391213589	0.369473289	Soltu.DM.11G001010/Soltu.DM.04G036140/Soltu.DM.03G035070/Soltu.DM.06G018130/Soltu.DM.03G035080	5
224	GO:0042744	hydrogen peroxide catabolic process	20/12756	0.033385178	0.391213589	0.369473289	Soltu.DM.02G022700/Soltu.DM.09G003770/Soltu.DM.02G023590/Soltu.DM.08G011330/Soltu.DM.02G023580	5

225	GO:0046462	monoacylglycerol metabolic process	20/12756	0.033385178	0.391213589	0.369473289	Soltu.DM.02G016290/Soltu.DM.02G016300/Soltu.DM.02G016770/Soltu.DM.02G016380/Soltu.DM.02G016780	5
226	GO:0050826	response to freezing	20/12756	0.033385178	0.391213589	0.369473289	Soltu.DM.03G030480/Soltu.DM.06G012350/Soltu.DM.06G009650/Soltu.DM.03G013120/Soltu.DM.12G028510	5
227	GO:0052651	monoacylglycerol catabolic process	20/12756	0.033385178	0.391213589	0.369473289	Soltu.DM.02G016290/Soltu.DM.02G016300/Soltu.DM.02G016770/Soltu.DM.02G016380/Soltu.DM.02G016780	5
228	GO:0010187	negative regulation of seed germination	41/12756	0.033589161	0.391822885	0.370048725	Soltu.DM.02G016290/Soltu.DM.02G016300/Soltu.DM.02G016770/Soltu.DM.02G016380/Soltu.DM.07G020080/Soltu.DM.02G016780/Soltu.DM.06G002140/Soltu.DM.04G033440	8
229	GO:0090693	plant organ senescence	139/12756	0.033907755	0.393757626	0.37187595	Soltu.DM.06G028410/Soltu.DM.05G026160/Soltu.DM.03G037070/Soltu.DM.12G027330/Soltu.DM.06G022310/Soltu.DM.03G017570/Soltu.DM.10G010160/Soltu.DM.03G037170/Soltu.DM.02G018270/Soltu.DM.03G016080/Soltu.DM.02G014410/Soltu.DM.05G024870/Soltu.DM.12G003790/Soltu.DM.05G011970/Soltu.DM.11G022310/Soltu.DM.03G033450/Soltu.DM.01G007500/Soltu.DM.02G014440/Soltu.DM.07G014750/Soltu.DM.06G010530	20
230	GO:1901419	regulation of response to alcohol	131/12756	0.035375003	0.402716375	0.380336848	Soltu.DM.09G018310/Soltu.DM.07G017210/Soltu.DM.07G017190/Soltu.DM.07G017200/Soltu.DM.04G033590/Soltu.DM.07G017180/Soltu.DM.07G012130/Soltu.DM.01G046820/Soltu.DM.09G028490/Soltu.DM.04G037130/Soltu.DM.06G017300/Soltu.DM.01G000060/Soltu.DM.04G024100/Soltu.DM.08G011890/Soltu.DM.09G026500/Soltu.DM.04G005970/Soltu.DM.02G026820/Soltu.DM.10G001460/Soltu.DM.07G024500	19
231	GO:1905957	regulation of cellular response to alcohol	131/12756	0.035375003	0.402716375	0.380336848	Soltu.DM.09G018310/Soltu.DM.07G017210/Soltu.DM.07G017190/Soltu.DM.07G017200/Soltu.DM.04G033590/Soltu.DM.07G017180/Soltu.DM.07G012130/Soltu.DM.01G046820/Soltu.DM.09G028490/Soltu.DM.04G037130/Soltu.DM.06G017300/Soltu.DM.01G000060/Soltu.DM.04G024100/Soltu.DM.08G011890/Soltu.DM.09G026500/Soltu.DM.04G005970/Soltu.DM.02G026820/Soltu.DM.10G001460/Soltu.DM.07G024500	19
232	GO:0006730	one-carbon metabolic process	14/12756	0.035460286	0.402716375	0.380336848	Soltu.DM.11G003760/Soltu.DM.05G023990/Soltu.DM.01G033180/Soltu.DM.12G002630	4
233	GO:2000038	regulation of stomatal complex development	14/12756	0.035460286	0.402716375	0.380336848	Soltu.DM.07G017210/Soltu.DM.07G017190/Soltu.DM.07G017200/Soltu.DM.07G017180	4
234	GO:0042440	pigment metabolic process	114/12756	0.035637318	0.402951776	0.380559168	Soltu.DM.06G028410/Soltu.DM.11G001010/Soltu.DM.01G024670/Soltu.DM.04G001370/Soltu.DM.04G018070/Soltu.DM.03G021440/Soltu.DM.08G013640/Soltu.DM.07G010570/Soltu.DM.08G028310/Soltu.DM.06G029640/Soltu.DM.03G020570/Soltu.DM.09G025040/Soltu.DM.04G031570/Soltu.DM.12G026560/Soltu.DM.08G026700/Soltu.DM.06G002140/Soltu.DM.07G014750	17
235	GO:0070482	response to oxygen levels	123/12756	0.036853149	0.414879553	0.391824101	Soltu.DM.07G011880/Soltu.DM.07G013360/Soltu.DM.01G006210/Soltu.DM.07G020920/Soltu.DM.10G024780/Soltu.DM.10G019450/Soltu.DM.03G037290/Soltu.DM.08G013420/Soltu.DM.06G026400/Soltu.DM.03G027730/Soltu.DM.01G051770/Soltu.DM.01G024860/Soltu.DM.08G001690/Soltu.DM.04G005970/Soltu.DM.10G022070/Soltu.DM.11G013880/Soltu.DM.11G011650/Soltu.DM.11G011740	18
236	GO:0042631	cellular response to water deprivation	50/12756	0.040057877	0.446777908	0.421949819	Soltu.DM.01G040570/Soltu.DM.07G022460/Soltu.DM.07G022500/Soltu.DM.08G030020/Soltu.DM.07G022530/Soltu.DM.07G022490/Soltu.DM.10G003300/Soltu.DM.07G025290/Soltu.DM.07G022450	9
237	GO:0019915	lipid storage	35/12756	0.04031697	0.446777908	0.421949819	Soltu.DM.02G016290/Soltu.DM.06G012350/Soltu.DM.02G016300/Soltu.DM.02G016770/Soltu.DM.02G016380/Soltu.DM.12G028510/Soltu.DM.02G016780	7
238	GO:0010200	response to chitin	133/12756	0.040449329	0.446777908	0.421949819	Soltu.DM.07G011880/Soltu.DM.08G028970/Soltu.DM.02G032550/Soltu.DM.03G027640/Soltu.DM.04G033180/Soltu.DM.06G018840/Soltu.DM.07G019030/Soltu.DM.07G019630/Soltu.DM.03G021360/Soltu.DM.08G002290/Soltu.DM.10G024950/Soltu.DM.01G046560/Soltu.DM.01G047760/Soltu.DM.06G021830/Soltu.DM.09G010330/Soltu.DM.11G026620/Soltu.DM.03G014570/Soltu.DM.09G003520/Soltu.DM.03G014580	19
239	GO:0010600	regulation of auxin biosynthetic process	21/12756	0.040553154	0.446777908	0.421949819	Soltu.DM.06G009270/Soltu.DM.03G035070/Soltu.DM.03G035080/Soltu.DM.02G004510/Soltu.DM.05G022790	5
240	GO:1903828	negative regulation of protein localization	21/12756	0.040553154	0.446777908	0.421949819	Soltu.DM.12G020370/Soltu.DM.12G020350/Soltu.DM.08G027150/Soltu.DM.09G002090/Soltu.DM.12G020340	5
24	GO:00	sucrose metabolic process	28/	0.0	0.4	0.4	Soltu.DM.05G006330/Soltu.DM.01G018690/Soltu.DM.01G040550/Soltu.DM.04G037250/S	6

1	05985		12756	41443604	50808483	25756409	oltu.DM.12G024030/Soltu.DM.06G020260	
242	GO:0015868	purine ribonucleotide transport	28/12756	0.041443604	0.450808483	0.425756409	Soltu.DM.01G038470/Soltu.DM.06G024610/Soltu.DM.03G032350/Soltu.DM.12G024350/Soltu.DM.03G034530/Soltu.DM.06G025410	6
243	GO:0033559	unsaturated fatty acid metabolic process	28/12756	0.041443604	0.450808483	0.425756409	Soltu.DM.04G034690/Soltu.DM.02G017970/Soltu.DM.10G004300/Soltu.DM.06G034310/Soltu.DM.07G026100/Soltu.DM.02G019940	6
244	GO:0046148	pigment biosynthetic process	91/12756	0.042466287	0.452686473	0.427530037	Soltu.DM.06G028410/Soltu.DM.01G024670/Soltu.DM.04G001370/Soltu.DM.03G021440/Soltu.DM.08G013640/Soltu.DM.08G028310/Soltu.DM.06G029640/Soltu.DM.03G020570/Soltu.DM.09G025040/Soltu.DM.04G031570/Soltu.DM.12G026560/Soltu.DM.08G026700/Soltu.DM.06G002140/Soltu.DM.07G014750	14
245	GO:0009411	response to UV	143/12756	0.043871861	0.452686473	0.427530037	Soltu.DM.07G028550/Soltu.DM.03G019450/Soltu.DM.01G024670/Soltu.DM.10G000640/Soltu.DM.09G025070/Soltu.DM.02G006310/Soltu.DM.02G024380/Soltu.DM.07G011650/Soltu.DM.07G011670/Soltu.DM.10G017480/Soltu.DM.07G011660/Soltu.DM.06G012170/Soltu.DM.05G007660/Soltu.DM.02G001620/Soltu.DM.02G001630/Soltu.DM.12G010860/Soltu.DM.12G026560/Soltu.DM.05G003920/Soltu.DM.02G033290/Soltu.DM.12G021450	20
246	GO:0097237	cellular response to toxic substance	152/12756	0.044395338	0.452686473	0.427530037	Soltu.DM.04G027640/Soltu.DM.04G027650/Soltu.DM.01G044300/Soltu.DM.02G031330/Soltu.DM.02G023420/Soltu.DM.09G021720/Soltu.DM.01G047440/Soltu.DM.02G022700/Soltu.DM.09G003770/Soltu.DM.02G024520/Soltu.DM.06G012170/Soltu.DM.02G019520/Soltu.DM.02G023590/Soltu.DM.05G018830/Soltu.DM.08G011330/Soltu.DM.02G023580/Soltu.DM.05G018810/Soltu.DM.12G003880/Soltu.DM.03G013090/Soltu.DM.09G005310/Soltu.DM.03G013100	21
247	GO:0010364	regulation of ethylene biosynthetic process	9/12756	0.044520266	0.452686473	0.427530037	Soltu.DM.03G035070/Soltu.DM.03G035080/Soltu.DM.11G018040	3
248	GO:0010371	regulation of gibberellin biosynthetic process	9/12756	0.044520266	0.452686473	0.427530037	Soltu.DM.08G013580/Soltu.DM.01G035240/Soltu.DM.10G005360	3
249	GO:0015801	aromatic amino acid transport	9/12756	0.044520266	0.452686473	0.427530037	Soltu.DM.04G031760/Soltu.DM.03G003280/Soltu.DM.12G006380	3
250	GO:0031335	regulation of sulfur amino acid metabolic process	9/12756	0.044520266	0.452686473	0.427530037	Soltu.DM.03G035070/Soltu.DM.03G035080/Soltu.DM.11G018040	3
251	GO:0033273	response to vitamin	9/12756	0.044520266	0.452686473	0.427530037	Soltu.DM.02G025590/Soltu.DM.02G025970/Soltu.DM.06G003240	3
252	GO:0033356	UDP-L-arabinose metabolic process	9/12756	0.044520266	0.452686473	0.427530037	Soltu.DM.04G020370/Soltu.DM.04G000320/Soltu.DM.05G001020	3
253	GO:0042436	indole-containing compound catabolic process	9/12756	0.044520266	0.452686473	0.427530037	Soltu.DM.03G035710/Soltu.DM.03G035070/Soltu.DM.03G035080	3
254	GO:0090480	purine nucleotide-sugar transmembrane transport	9/12756	0.044520266	0.452686473	0.427530037	Soltu.DM.12G004580/Soltu.DM.12G007520/Soltu.DM.12G024350	3
255	GO:1900908	regulation of olefin metabolic process	9/12756	0.044520266	0.452686473	0.427530037	Soltu.DM.03G035070/Soltu.DM.03G035080/Soltu.DM.11G018040	3
256	GO:1900911	regulation of olefin biosynthetic process	9/12756	0.044520266	0.452686473	0.427530037	Soltu.DM.03G035070/Soltu.DM.03G035080/Soltu.DM.11G018040	3
257	GO:0071281	cellular response to iron ion	75/12756	0.044551022	0.452686473	0.427530037	Soltu.DM.01G006210/Soltu.DM.07G028550/Soltu.DM.10G000640/Soltu.DM.07G020920/Soltu.DM.04G003430/Soltu.DM.10G024780/Soltu.DM.02G030410/Soltu.DM.08G013420/Soltu.DM.04G034620/Soltu.DM.01G002310/Soltu.DM.10G022070/Soltu.DM.07G022640	12
25	GO:00	glucuronoxylan metabolic process	15/	0.0	0.4	0.4	Soltu.DM.10G001690/Soltu.DM.07G025450/Soltu.DM.09G006300/Soltu.DM.11G008240	4

8	10413		12756	44879171	52686473	27530037		
259	GO:0010417	glucuronoxylan biosynthetic process	15/12756	0.044879171	0.452686473	0.427530037	Soltu.DM.10G001690/Soltu.DM.07G025450/Soltu.DM.09G006300/Soltu.DM.11G008240	4
260	GO:0019369	arachidonic acid metabolic process	15/12756	0.044879171	0.452686473	0.427530037	Soltu.DM.04G034690/Soltu.DM.10G004300/Soltu.DM.06G034310/Soltu.DM.02G019940	4
261	GO:0019755	one-carbon compound transport	15/12756	0.044879171	0.452686473	0.427530037	Soltu.DM.12G010360/Soltu.DM.06G018020/Soltu.DM.06G031120/Soltu.DM.03G031200	4
262	GO:0006855	xenobiotic transmembrane transport	126/12756	0.045203423	0.452686473	0.427530037	Soltu.DM.05G003990/Soltu.DM.03G035710/Soltu.DM.04G031760/Soltu.DM.09G028710/Soltu.DM.01G038470/Soltu.DM.12G006380/Soltu.DM.05G022850/Soltu.DM.06G024610/Soltu.DM.05G026690/Soltu.DM.03G032350/Soltu.DM.10G028240/Soltu.DM.11G003270/Soltu.DM.03G034530/Soltu.DM.05G013430/Soltu.DM.06G018560/Soltu.DM.03G031200/Soltu.DM.05G021160/Soltu.DM.06G025410	18
263	GO:0009072	aromatic amino acid metabolic process	59/12756	0.045303767	0.452686473	0.427530037	Soltu.DM.04G023360/Soltu.DM.04G018630/Soltu.DM.02G031330/Soltu.DM.05G007640/Soltu.DM.03G035070/Soltu.DM.05G007630/Soltu.DM.02G020220/Soltu.DM.03G035080/Soltu.DM.08G011890/Soltu.DM.04G009170	10
264	GO:0042178	xenobiotic catabolic process	59/12756	0.045303767	0.452686473	0.427530037	Soltu.DM.06G028410/Soltu.DM.02G017810/Soltu.DM.08G013400/Soltu.DM.02G022700/Soltu.DM.09G003770/Soltu.DM.10G012990/Soltu.DM.03G019550/Soltu.DM.02G023590/Soltu.DM.08G011330/Soltu.DM.02G023580	10
265	GO:0007584	response to nutrient	36/12756	0.046168251	0.456022036	0.430680237	Soltu.DM.02G025590/Soltu.DM.01G008040/Soltu.DM.02G025970/Soltu.DM.06G026400/Soltu.DM.06G003240/Soltu.DM.05G001260/Soltu.DM.06G026560	7
266	GO:0010167	response to nitrate	36/12756	0.046168251	0.456022036	0.430680237	Soltu.DM.02G010790/Soltu.DM.06G026960/Soltu.DM.08G025260/Soltu.DM.10G010160/Soltu.DM.09G031790/Soltu.DM.06G017770/Soltu.DM.09G023400	7
267	GO:0046283	anthocyanin-containing compound metabolic process	36/12756	0.046168251	0.456022036	0.430680237	Soltu.DM.11G001010/Soltu.DM.04G001370/Soltu.DM.04G018070/Soltu.DM.03G020570/Soltu.DM.09G025040/Soltu.DM.08G026700/Soltu.DM.07G014750	7
268	GO:0015780	nucleotide-sugar transmembrane transport	29/12756	0.048278662	0.4739746	0.447635151	Soltu.DM.12G004580/Soltu.DM.11G022930/Soltu.DM.12G004060/Soltu.DM.12G007520/Soltu.DM.12G024350/Soltu.DM.03G032030	6
269	GO:1903338	regulation of cell wall organization or biogenesis	22/12756	0.048582399	0.4739746	0.447635151	Soltu.DM.07G028550/Soltu.DM.10G000640/Soltu.DM.11G011390/Soltu.DM.01G027520/Soltu.DM.03G035510	5
270	GO:0009813	flavonoid biosynthetic process	76/12756	0.048598804	0.4739746	0.447635151	Soltu.DM.06G028410/Soltu.DM.01G024670/Soltu.DM.10G029960/Soltu.DM.04G001370/Soltu.DM.03G021440/Soltu.DM.09G028560/Soltu.DM.03G020570/Soltu.DM.09G025040/Soltu.DM.03G018210/Soltu.DM.12G026560/Soltu.DM.08G026700/Soltu.DM.07G014750	12
271	GO:0010043	response to zinc ion	44/12756	0.048721206	0.4739746	0.447635151	Soltu.DM.07G002440/Soltu.DM.01G035900/Soltu.DM.07G028550/Soltu.DM.03G034540/Soltu.DM.10G000640/Soltu.DM.02G025970/Soltu.DM.09G003770/Soltu.DM.01G035910	8
272	GO:0051235	maintenance of location	69/12756	0.054159443	0.519024584	0.490181644	Soltu.DM.02G016290/Soltu.DM.06G012350/Soltu.DM.04G003430/Soltu.DM.07G009580/Soltu.DM.06G022970/Soltu.DM.02G016300/Soltu.DM.02G016770/Soltu.DM.02G016380/Soltu.DM.12G028510/Soltu.DM.12G009990/Soltu.DM.02G016780	11
273	GO:0009873	ethylene-activated signaling pathway	61/12756	0.055023516	0.519024584	0.490181644	Soltu.DM.01G006210/Soltu.DM.07G028550/Soltu.DM.10G000640/Soltu.DM.07G020090/Soltu.DM.08G028440/Soltu.DM.05G024870/Soltu.DM.01G002310/Soltu.DM.09G026500/Soltu.DM.02G017280/Soltu.DM.07G022640	10
274	GO:0009825	multidimensional cell growth	53/12756	0.055277213	0.519024584	0.490181644	Soltu.DM.04G027320/Soltu.DM.12G007610/Soltu.DM.02G009140/Soltu.DM.08G014180/Soltu.DM.07G019030/Soltu.DM.09G031790/Soltu.DM.05G026200/Soltu.DM.01G025270/Soltu.DM.01G040720	9
275	GO:0009269	response to desiccation	16/1275	0.0555549	0.5190245	0.4901816	Soltu.DM.01G040570/Soltu.DM.05G018830/Soltu.DM.05G018810/Soltu.DM.03G013100	4

			6	79	84	44		
276	GO:0017000	antibiotic biosynthetic process	16/12756	0.055554979	0.519024584	0.490181644	Soltu.DM.08G028440/Soltu.DM.07G022590/Soltu.DM.09G023400/Soltu.DM.07G022640	4
277	GO:0052482	defense response by cell wall thickening	16/12756	0.055554979	0.519024584	0.490181644	Soltu.DM.03G035710/Soltu.DM.05G007640/Soltu.DM.05G007630/Soltu.DM.07G022640	4
278	GO:0052544	defense response by callose deposition in cell wall	16/12756	0.055554979	0.519024584	0.490181644	Soltu.DM.03G035710/Soltu.DM.05G007640/Soltu.DM.05G007630/Soltu.DM.07G022640	4
279	GO:0071491	cellular response to red light	16/12756	0.055554979	0.519024584	0.490181644	Soltu.DM.09G025070/Soltu.DM.01G035240/Soltu.DM.10G005360/Soltu.DM.06G002140	4
280	GO:0090333	regulation of stomatal closure	16/12756	0.055554979	0.519024584	0.490181644	Soltu.DM.01G035900/Soltu.DM.04G037130/Soltu.DM.12G024710/Soltu.DM.01G035910	4
281	GO:0046461	neutral lipid catabolic process	30/12756	0.055767963	0.519024584	0.490181644	Soltu.DM.02G016290/Soltu.DM.02G016300/Soltu.DM.06G034310/Soltu.DM.02G016770/Soltu.DM.02G016380/Soltu.DM.02G016780	6
282	GO:0046464	acylglycerol catabolic process	30/12756	0.055767963	0.519024584	0.490181644	Soltu.DM.02G016290/Soltu.DM.02G016300/Soltu.DM.06G034310/Soltu.DM.02G016770/Soltu.DM.02G016380/Soltu.DM.02G016780	6
283	GO:0140115	export across plasma membrane	30/12756	0.055767963	0.519024584	0.490181644	Soltu.DM.09G002620/Soltu.DM.11G011180/Soltu.DM.02G032050/Soltu.DM.10G026500/Soltu.DM.02G022410/Soltu.DM.04G002690	6
284	GO:0005992	trehalose biosynthetic process	23/12756	0.057481153	0.528289203	0.498931414	Soltu.DM.04G012960/Soltu.DM.02G015310/Soltu.DM.07G001730/Soltu.DM.05G008060/Soltu.DM.07G014750	5
285	GO:1901679	nucleotide transmembrane transport	23/12756	0.057481153	0.528289203	0.498931414	Soltu.DM.01G038470/Soltu.DM.06G024610/Soltu.DM.03G032350/Soltu.DM.03G034530/Soltu.DM.06G025410	5
286	GO:0009969	xyloglucan biosynthetic process	10/12756	0.059290678	0.528289203	0.498931414	Soltu.DM.09G018910/Soltu.DM.08G029290/Soltu.DM.12G008450	3
287	GO:0009970	cellular response to sulfate starvation	10/12756	0.059290678	0.528289203	0.498931414	Soltu.DM.08G030020/Soltu.DM.03G000340/Soltu.DM.07G020410	3
288	GO:0010977	negative regulation of neuron projection development	10/12756	0.059290678	0.528289203	0.498931414	Soltu.DM.12G020370/Soltu.DM.12G020350/Soltu.DM.12G020340	3
289	GO:0015800	acidic amino acid transport	10/12756	0.059290678	0.528289203	0.498931414	Soltu.DM.05G003990/Soltu.DM.04G031760/Soltu.DM.12G006380	3
290	GO:0015813	L-glutamate transmembrane transport	10/12756	0.059290678	0.528289203	0.498931414	Soltu.DM.05G003990/Soltu.DM.04G031760/Soltu.DM.12G006380	3
291	GO:0031345	negative regulation of cell projection organization	10/12756	0.059290678	0.528289203	0.498931414	Soltu.DM.12G020370/Soltu.DM.12G020350/Soltu.DM.12G020340	3
292	GO:0045665	negative regulation of neuron differentiation	10/12756	0.059290678	0.528289203	0.498931414	Soltu.DM.12G020370/Soltu.DM.12G020350/Soltu.DM.12G020340	3
293	GO:0046470	phosphatidylcholine metabolic process	10/12	0.0592	0.5282	0.4989	Soltu.DM.03G030800/Soltu.DM.06G018040/Soltu.DM.02G019940	3

			756	90678	89203	31414		
294	GO:0051938	L-glutamate import	10/12756	0.059290678	0.528289203	0.498931414	Soltu.DM.05G003990/Soltu.DM.04G031760/Soltu.DM.12G006380	3
295	GO:1905475	regulation of protein localization to membrane	10/12756	0.059290678	0.528289203	0.498931414	Soltu.DM.12G020370/Soltu.DM.12G020350/Soltu.DM.12G020340	3
296	GO:0016132	brassinosteroid biosynthetic process	38/12756	0.059427412	0.528289203	0.498931414	Soltu.DM.08G020150/Soltu.DM.05G015440/Soltu.DM.06G004460/Soltu.DM.02G030630/Soltu.DM.10G003570/Soltu.DM.08G027080/Soltu.DM.10G003550	7
297	GO:0072329	monocarboxylic acid catabolic process	87/12756	0.059989996	0.531457761	0.501923891	Soltu.DM.06G028410/Soltu.DM.08G024620/Soltu.DM.09G018310/Soltu.DM.01G033530/Soltu.DM.08G020150/Soltu.DM.01G038470/Soltu.DM.09G028490/Soltu.DM.07G013940/Soltu.DM.12G024030/Soltu.DM.07G013900/Soltu.DM.06G018150/Soltu.DM.04G009170/Soltu.DM.10G003920	13
298	GO:0010105	negative regulation of ethylene-activated signaling pathway	46/12756	0.060932462	0.533792809	0.504129177	Soltu.DM.07G020920/Soltu.DM.10G024780/Soltu.DM.06G014700/Soltu.DM.08G013420/Soltu.DM.08G013440/Soltu.DM.10G022070/Soltu.DM.07G022640/Soltu.DM.05G025620	8
299	GO:0070298	negative regulation of phosphorelay signal transduction system	46/12756	0.060932462	0.533792809	0.504129177	Soltu.DM.07G020920/Soltu.DM.10G024780/Soltu.DM.06G014700/Soltu.DM.08G013420/Soltu.DM.08G013440/Soltu.DM.10G022070/Soltu.DM.07G022640/Soltu.DM.05G025620	8
300	GO:0010017	red or far-red light signaling pathway	54/12756	0.061081799	0.533792809	0.504129177	Soltu.DM.06G018130/Soltu.DM.07G015980/Soltu.DM.01G035240/Soltu.DM.06G021830/Soltu.DM.07G028470/Soltu.DM.10G005360/Soltu.DM.10G000040/Soltu.DM.02G011380/Soltu.DM.06G002140	9
301	GO:0045927	positive regulation of growth	54/12756	0.061081799	0.533792809	0.504129177	Soltu.DM.12G020370/Soltu.DM.02G018520/Soltu.DM.12G020350/Soltu.DM.09G023300/Soltu.DM.08G011890/Soltu.DM.04G006870/Soltu.DM.12G026380/Soltu.DM.12G020340/Soltu.DM.06G001230	9
302	GO:0007267	cell-cell signaling	31/12756	0.063917612	0.547440541	0.517018485	Soltu.DM.01G039230/Soltu.DM.02G028750/Soltu.DM.02G022460/Soltu.DM.11G026460/Soltu.DM.07G002580/Soltu.DM.08G026840	6
303	GO:0015865	purine nucleotide transport	31/12756	0.063917612	0.547440541	0.517018485	Soltu.DM.01G038470/Soltu.DM.06G024610/Soltu.DM.03G032350/Soltu.DM.12G024350/Soltu.DM.03G034530/Soltu.DM.06G025410	6
304	GO:0051051	negative regulation of transport	31/12756	0.063917612	0.547440541	0.517018485	Soltu.DM.12G020370/Soltu.DM.12G020350/Soltu.DM.08G027150/Soltu.DM.05G011970/Soltu.DM.09G002090/Soltu.DM.12G020340	6
305	GO:0051348	negative regulation of transferase activity	31/12756	0.063917612	0.547440541	0.517018485	Soltu.DM.07G012130/Soltu.DM.11G010230/Soltu.DM.11G010220/Soltu.DM.06G028580/Soltu.DM.04G038280/Soltu.DM.02G013390	6
306	GO:0051503	adenine nucleotide transport	31/12756	0.063917612	0.547440541	0.517018485	Soltu.DM.01G038470/Soltu.DM.06G024610/Soltu.DM.03G032350/Soltu.DM.12G024350/Soltu.DM.03G034530/Soltu.DM.06G025410	6
307	GO:0052548	regulation of endopeptidase activity	31/12756	0.063917612	0.547440541	0.517018485	Soltu.DM.03G020450/Soltu.DM.04G034360/Soltu.DM.04G034390/Soltu.DM.04G034380/Soltu.DM.04G003450/Soltu.DM.04G034280	6
308	GO:0010565	regulation of cellular ketone metabolic process	63/12756	0.066023785	0.562898261	0.531617198	Soltu.DM.03G035070/Soltu.DM.02G020950/Soltu.DM.08G013580/Soltu.DM.09G031790/Soltu.DM.03G035080/Soltu.DM.01G035240/Soltu.DM.11G018040/Soltu.DM.10G005360/Soltu.DM.07G024240/Soltu.DM.09G023400	10
309	GO:0006515	protein quality control for misfolded or incompletely synthesized proteins	24/12756	0.067249122	0.562898261	0.531617198	Soltu.DM.03G016820/Soltu.DM.06G012970/Soltu.DM.10G004220/Soltu.DM.05G005120/Soltu.DM.06G009790	5
310	GO:0032436	positive regulation of proteasomal ubiquitin-dependent protein catabolic process	24/12756	0.067249122	0.562898261	0.531617198	Soltu.DM.12G005510/Soltu.DM.06G015770/Soltu.DM.04G034740/Soltu.DM.09G005140/Soltu.DM.06G024530	5
31	GO:00	regulation of sulfur metabolic process	24/	0.0	0.5	0.5	Soltu.DM.03G035070/Soltu.DM.12G022190/Soltu.DM.03G035080/Soltu.DM.11G018040/S	5

1	42762		12756	67249122	62898261	31617198	oltu.DM.01G002310	
312	GO:0090354	regulation of auxin metabolic process	24/12756	0.067249122	0.562898261	0.531617198	Soltu.DM.06G009270/Soltu.DM.03G035070/Soltu.DM.03G035080/Soltu.DM.02G004510/Soltu.DM.05G022790	5
313	GO:0010193	response to ozone	55/12756	0.067261251	0.562898261	0.531617198	Soltu.DM.06G029230/Soltu.DM.03G037120/Soltu.DM.07G003270/Soltu.DM.09G028710/Soltu.DM.03G019450/Soltu.DM.06G009750/Soltu.DM.11G024620/Soltu.DM.07G024240/Soltu.DM.06G010530	9
314	GO:0051346	negative regulation of hydrolase activity	55/12756	0.067261251	0.562898261	0.531617198	Soltu.DM.09G031320/Soltu.DM.03G020450/Soltu.DM.04G034360/Soltu.DM.04G034390/Soltu.DM.04G034380/Soltu.DM.04G003450/Soltu.DM.08G011890/Soltu.DM.09G031340/Soltu.DM.04G034280	9
315	GO:0006570	tyrosine metabolic process	17/12756	0.067469186	0.562898261	0.531617198	Soltu.DM.03G035070/Soltu.DM.03G035080/Soltu.DM.08G011890/Soltu.DM.04G009170	4
316	GO:0018958	phenol-containing compound metabolic process	47/12756	0.067695837	0.562967317	0.531682415	Soltu.DM.06G028410/Soltu.DM.05G007640/Soltu.DM.05G007630/Soltu.DM.04G009170/Soltu.DM.08G026700/Soltu.DM.02G008560/Soltu.DM.09G023400/Soltu.DM.02G008550	8
317	GO:0015931	nucleobase-containing compound transport	151/12756	0.069845406	0.570032361	0.538354845	Soltu.DM.04G030440/Soltu.DM.12G004580/Soltu.DM.10G000530/Soltu.DM.01G038470/Soltu.DM.11G022930/Soltu.DM.12G004060/Soltu.DM.12G005490/Soltu.DM.03G003730/Soltu.DM.12G007520/Soltu.DM.06G024610/Soltu.DM.03G032350/Soltu.DM.12G024350/Soltu.DM.06G012690/Soltu.DM.03G034530/Soltu.DM.09G005370/Soltu.DM.11G024760/Soltu.DM.04G011330/Soltu.DM.03G032030/Soltu.DM.07G006510/Soltu.DM.06G025410	20
318	GO:0098661	inorganic anion transmembrane transport	64/12756	0.072014978	0.570032361	0.538354845	Soltu.DM.02G010790/Soltu.DM.08G025260/Soltu.DM.09G020160/Soltu.DM.10G010160/Soltu.DM.03G035250/Soltu.DM.03G027330/Soltu.DM.06G017770/Soltu.DM.03G031200/Soltu.DM.05G021160/Soltu.DM.03G035260	10
319	GO:0006687	glycosphingolipid metabolic process	5/12756	0.072083223	0.570032361	0.538354845	Soltu.DM.01G042210/Soltu.DM.10G022360	2
320	GO:0015810	aspartate transmembrane transport	5/12756	0.072083223	0.570032361	0.538354845	Soltu.DM.04G031760/Soltu.DM.12G006380	2
321	GO:0015822	ornithine transport	5/12756	0.072083223	0.570032361	0.538354845	Soltu.DM.10G028240/Soltu.DM.06G018560	2
322	GO:0015837	amine transport	5/12756	0.072083223	0.570032361	0.538354845	Soltu.DM.12G010360/Soltu.DM.06G018020	2
323	GO:0015843	methylammonium transport	5/12756	0.072083223	0.570032361	0.538354845	Soltu.DM.12G010360/Soltu.DM.06G018020	2
324	GO:0018175	protein nucleotidylation	5/12756	0.072083223	0.570032361	0.538354845	Soltu.DM.01G008040/Soltu.DM.05G001260	2
325	GO:0018177	protein uridylylation	5/12756	0.072083223	0.570032361	0.538354845	Soltu.DM.01G008040/Soltu.DM.05G001260	2
326	GO:0044380	protein localization to cytoskeleton	5/12756	0.072083223	0.570032361	0.538354845	Soltu.DM.08G015350/Soltu.DM.04G022240	2
327	GO:0070932	histone H3 deacetylation	5/12756	0.072083223	0.570032361	0.538354845	Soltu.DM.02G001620/Soltu.DM.02G001630	2
328	GO:0072698	protein localization to microtubule cytoskeleton	5/12756	0.0720832	0.5700323	0.5383548	Soltu.DM.08G015350/Soltu.DM.04G022240	2

				23	61	45		
329	GO:0090293	nitrogen catabolite regulation of transcription	5/12756	0.072083223	0.570032361	0.538354845	Soltu.DM.01G008040/Soltu.DM.05G001260	2
330	GO:1901070	guanosine-containing compound biosynthetic process	5/12756	0.072083223	0.570032361	0.538354845	Soltu.DM.03G037170/Soltu.DM.10G027910	2
331	GO:1903352	L-ornithine transmembrane transport	5/12756	0.072083223	0.570032361	0.538354845	Soltu.DM.10G028240/Soltu.DM.06G018560	2
332	GO:1990937	xylan acetylation	5/12756	0.072083223	0.570032361	0.538354845	Soltu.DM.06G010280/Soltu.DM.02G014140	2
333	GO:0010114	response to red light	81/12756	0.072592976	0.571631125	0.539864763	Soltu.DM.02G025590/Soltu.DM.03G027640/Soltu.DM.09G025070/Soltu.DM.03G022850/Soltu.DM.08G011110/Soltu.DM.02G013430/Soltu.DM.01G035240/Soltu.DM.10G005360/Soltu.DM.03G021710/Soltu.DM.06G002140/Soltu.DM.04G033440/Soltu.DM.06G023440	12
334	GO:0036260	RNA capping	32/12756	0.072728863	0.571631125	0.539864763	Soltu.DM.11G014610/Soltu.DM.12G016740/Soltu.DM.11G014620/Soltu.DM.01G051600/Soltu.DM.11G004920/Soltu.DM.06G029830	6
335	GO:1901606	alpha-amino acid catabolic process	56/12756	0.073818986	0.578435704	0.546291202	Soltu.DM.08G013400/Soltu.DM.03G035070/Soltu.DM.08G030020/Soltu.DM.07G019380/Soltu.DM.01G047450/Soltu.DM.12G024030/Soltu.DM.03G035080/Soltu.DM.08G007450/Soltu.DM.04G009170	9
336	GO:0009751	response to salicylic acid	198/12756	0.0743071063	0.579100187	0.546918759	Soltu.DM.06G028410/Soltu.DM.02G025590/Soltu.DM.06G026960/Soltu.DM.10G000560/Soltu.DM.09G018910/Soltu.DM.09G028710/Soltu.DM.07G028550/Soltu.DM.10G022380/Soltu.DM.04G033180/Soltu.DM.09G029050/Soltu.DM.02G030600/Soltu.DM.10G000640/Soltu.DM.02G006310/Soltu.DM.10G017480/Soltu.DM.08G006060/Soltu.DM.05G027000/Soltu.DM.07G025540/Soltu.DM.04G028540/Soltu.DM.02G004510/Soltu.DM.11G026620/Soltu.DM.06G020450/Soltu.DM.02G029580/Soltu.DM.07G024240/Soltu.DM.01G027520/Soltu.DM.09G023400	25
337	GO:0036294	cellular response to decreased oxygen levels	40/12756	0.074802313	0.579100187	0.546918759	Soltu.DM.07G011880/Soltu.DM.03G027730/Soltu.DM.01G051770/Soltu.DM.01G024860/Soltu.DM.11G013880/Soltu.DM.11G011650/Soltu.DM.11G011740	7
338	GO:0071453	cellular response to oxygen levels	40/12756	0.074802313	0.579100187	0.546918759	Soltu.DM.07G011880/Soltu.DM.03G027730/Soltu.DM.01G051770/Soltu.DM.01G024860/Soltu.DM.11G013880/Soltu.DM.11G011650/Soltu.DM.11G011740	7
339	GO:0071897	DNA biosynthetic process	40/12756	0.074802313	0.579100187	0.546918759	Soltu.DM.08G027160/Soltu.DM.05G007660/Soltu.DM.06G015770/Soltu.DM.03G032560/Soltu.DM.02G033290/Soltu.DM.05G023970/Soltu.DM.02G013390	7
340	GO:0009647	skotomorphogenesis	11/12756	0.076031385	0.583359856	0.550941712	Soltu.DM.11G001010/Soltu.DM.02G030630/Soltu.DM.01G032120	3
341	GO:0010337	regulation of salicylic acid metabolic process	11/12756	0.076031385	0.583359856	0.550941712	Soltu.DM.09G031790/Soltu.DM.07G024240/Soltu.DM.09G023400	3
342	GO:1903409	reactive oxygen species biosynthetic process	11/12756	0.076031385	0.583359856	0.550941712	Soltu.DM.08G028440/Soltu.DM.07G022590/Soltu.DM.07G022640	3
343	GO:0005991	trehalose metabolic process	25/12756	0.077877887	0.588785808	0.556066136	Soltu.DM.04G012960/Soltu.DM.02G015310/Soltu.DM.07G001730/Soltu.DM.05G008060/Soltu.DM.07G014750	5
344	GO:0052546	cell wall pectin metabolic process	25/12756	0.077877887	0.588785808	0.556066136	Soltu.DM.02G031090/Soltu.DM.07G000930/Soltu.DM.02G031050/Soltu.DM.04G029850/Soltu.DM.06G021870	5
345	GO:2000060	positive regulation of ubiquitin-dependent protein catabolic process	25/1275	0.0778778	0.5887858	0.5606661	Soltu.DM.12G005510/Soltu.DM.06G015770/Soltu.DM.04G034740/Soltu.DM.09G005140/Soltu.DM.06G024530	5

			6	87	08	36		
346	GO:0016125	sterol metabolic process	82/12756	0.078168026	0.588785808	0.556066136	Soltu.DM.04G034690/Soltu.DM.08G020150/Soltu.DM.11G023180/Soltu.DM.05G015440/Soltu.DM.06G004460/Soltu.DM.07G017000/Soltu.DM.02G030630/Soltu.DM.01G048780/Soltu.DM.02G007460/Soltu.DM.10G003570/Soltu.DM.08G027080/Soltu.DM.10G003550	12
347	GO:0006638	neutral lipid metabolic process	65/12756	0.078337289	0.588785808	0.556066136	Soltu.DM.02G016290/Soltu.DM.02G024810/Soltu.DM.12G002120/Soltu.DM.01G034820/Soltu.DM.02G016300/Soltu.DM.06G034310/Soltu.DM.02G016770/Soltu.DM.02G016380/Soltu.DM.02G016780/Soltu.DM.10G005430	10
348	GO:0006639	acylglycerol metabolic process	65/12756	0.078337289	0.588785808	0.556066136	Soltu.DM.02G016290/Soltu.DM.02G024810/Soltu.DM.12G002120/Soltu.DM.01G034820/Soltu.DM.02G016300/Soltu.DM.06G034310/Soltu.DM.02G016770/Soltu.DM.02G016380/Soltu.DM.02G016780/Soltu.DM.10G005430	10
349	GO:0009063	amino acid catabolic process	65/12756	0.078337289	0.588785808	0.556066136	Soltu.DM.04G025250/Soltu.DM.08G013400/Soltu.DM.03G035070/Soltu.DM.08G030020/Soltu.DM.07G019380/Soltu.DM.01G047450/Soltu.DM.12G024030/Soltu.DM.03G035080/Soltu.DM.08G007450/Soltu.DM.04G009170	10
350	GO:0006012	galactose metabolic process	18/12756	0.080587267	0.596994177	0.563818354	Soltu.DM.01G040570/Soltu.DM.02G031920/Soltu.DM.02G031890/Soltu.DM.02G024820	4
351	GO:0050769	positive regulation of neurogenesis	18/12756	0.080587267	0.596994177	0.563818354	Soltu.DM.12G020370/Soltu.DM.12G020350/Soltu.DM.12G025260/Soltu.DM.12G020340	4
352	GO:0051962	positive regulation of nervous system development	18/12756	0.080587267	0.596994177	0.563818354	Soltu.DM.12G020370/Soltu.DM.12G020350/Soltu.DM.12G025260/Soltu.DM.12G020340	4
353	GO:1902074	response to salt	18/12756	0.080587267	0.596994177	0.563818354	Soltu.DM.03G017570/Soltu.DM.03G022540/Soltu.DM.02G020950/Soltu.DM.04G024100	4
354	GO:2000032	regulation of secondary shoot formation	18/12756	0.080587267	0.596994177	0.563818354	Soltu.DM.06G025210/Soltu.DM.02G023840/Soltu.DM.02G017390/Soltu.DM.03G034300	4
355	GO:0006862	nucleotide transport	33/12756	0.082198442	0.607185055	0.57344291	Soltu.DM.01G038470/Soltu.DM.06G024610/Soltu.DM.03G032350/Soltu.DM.12G024350/Soltu.DM.03G034530/Soltu.DM.06G025410	6
356	GO:0034440	lipid oxidation	66/12756	0.084992211	0.626028343	0.59123905	Soltu.DM.08G024620/Soltu.DM.09G018310/Soltu.DM.03G037120/Soltu.DM.01G038470/Soltu.DM.09G028490/Soltu.DM.05G011130/Soltu.DM.06G018150/Soltu.DM.11G001520/Soltu.DM.10G003920/Soltu.DM.08G026840	10
357	GO:0042908	xenobiotic transport	137/12756	0.087240938	0.640761078	0.605153066	Soltu.DM.05G003990/Soltu.DM.03G035710/Soltu.DM.04G031760/Soltu.DM.09G028710/Soltu.DM.01G038470/Soltu.DM.12G006380/Soltu.DM.05G022850/Soltu.DM.06G024610/Soltu.DM.05G026690/Soltu.DM.03G032350/Soltu.DM.10G028240/Soltu.DM.11G003270/Soltu.DM.03G034530/Soltu.DM.05G013430/Soltu.DM.06G018560/Soltu.DM.03G031200/Soltu.DM.05G021160/Soltu.DM.06G025410	18
358	GO:0001933	negative regulation of protein phosphorylation	26/12756	0.089351424	0.643464333	0.607706097	Soltu.DM.07G012130/Soltu.DM.11G010230/Soltu.DM.11G010220/Soltu.DM.06G028580/Soltu.DM.04G038280	5
359	GO:0062197	cellular response to chemical stress	156/12756	0.090548346	0.643464333	0.607706097	Soltu.DM.07G028550/Soltu.DM.02G020550/Soltu.DM.10G000640/Soltu.DM.01G047440/Soltu.DM.07G020410/Soltu.DM.02G030410/Soltu.DM.02G024520/Soltu.DM.06G024610/Soltu.DM.08G028440/Soltu.DM.07G015200/Soltu.DM.03G032350/Soltu.DM.06G012170/Soltu.DM.03G022850/Soltu.DM.01G008180/Soltu.DM.02G019520/Soltu.DM.02G023590/Soltu.DM.08G011330/Soltu.DM.02G023580/Soltu.DM.06G026560/Soltu.DM.09G005310	20
360	GO:0044003	modulation by symbiont of host process	50/12756	0.090647757	0.643464333	0.607706097	Soltu.DM.01G024680/Soltu.DM.02G012280/Soltu.DM.05G026810/Soltu.DM.07G014680/Soltu.DM.01G005590/Soltu.DM.03G024130/Soltu.DM.01G010020/Soltu.DM.11G004150	8
361	GO:0016115	terpenoid catabolic process	42/12756	0.092300671	0.643464333	0.607706097	Soltu.DM.07G022720/Soltu.DM.07G022710/Soltu.DM.08G020150/Soltu.DM.07G013940/Soltu.DM.07G022700/Soltu.DM.07G013900/Soltu.DM.10G003240	7
362	GO:0016129	phytosteroid biosynthetic process	42/12756	0.092300671	0.643464333	0.607706097	Soltu.DM.08G020150/Soltu.DM.05G015440/Soltu.DM.06G004460/Soltu.DM.02G030630/Soltu.DM.10G003570/Soltu.DM.08G027080/Soltu.DM.10G003550	7

363	GO:0009696	salicylic acid metabolic process	34/12756	0.092318662	0.643464333	0.607706097	Soltu.DM.06G028410/Soltu.DM.05G007640/Soltu.DM.05G007630/Soltu.DM.02G008560/Soltu.DM.09G023400/Soltu.DM.02G008550	6
364	GO:0000055	ribosomal large subunit export from nucleus	12/12756	0.094583426	0.643464333	0.607706097	Soltu.DM.12G024350/Soltu.DM.09G005370/Soltu.DM.07G006510	3
365	GO:0006521	regulation of cellular amino acid metabolic process	12/12756	0.094583426	0.643464333	0.607706097	Soltu.DM.03G035070/Soltu.DM.03G035080/Soltu.DM.11G018040	3
366	GO:0006783	heme biosynthetic process	12/12756	0.094583426	0.643464333	0.607706097	Soltu.DM.08G013640/Soltu.DM.04G031570/Soltu.DM.06G002140	3
367	GO:0009423	chorismate biosynthetic process	12/12756	0.094583426	0.643464333	0.607706097	Soltu.DM.04G018630/Soltu.DM.01G031840/Soltu.DM.01G022690	3
368	GO:0009700	indole phytoalexin biosynthetic process	12/12756	0.094583426	0.643464333	0.607706097	Soltu.DM.12G022190/Soltu.DM.06G018840/Soltu.DM.07G014750	3
369	GO:0010120	camalexin biosynthetic process	12/12756	0.094583426	0.643464333	0.607706097	Soltu.DM.12G022190/Soltu.DM.06G018840/Soltu.DM.07G014750	3
370	GO:0010555	response to mannitol	12/12756	0.094583426	0.643464333	0.607706097	Soltu.DM.07G013360/Soltu.DM.03G017570/Soltu.DM.12G025260	3
371	GO:0010976	positive regulation of neuron projection development	12/12756	0.094583426	0.643464333	0.607706097	Soltu.DM.12G020370/Soltu.DM.12G020350/Soltu.DM.12G020340	3
372	GO:0016139	glycoside catabolic process	12/12756	0.094583426	0.643464333	0.607706097	Soltu.DM.01G042210/Soltu.DM.02G008560/Soltu.DM.02G008550	3
373	GO:0033238	regulation of amine metabolic process	12/12756	0.094583426	0.643464333	0.607706097	Soltu.DM.03G035070/Soltu.DM.03G035080/Soltu.DM.11G018040	3
374	GO:0045828	positive regulation of isoprenoid metabolic process	12/12756	0.094583426	0.643464333	0.607706097	Soltu.DM.02G020950/Soltu.DM.01G035240/Soltu.DM.10G005360	3
375	GO:0046217	indole phytoalexin metabolic process	12/12756	0.094583426	0.643464333	0.607706097	Soltu.DM.12G022190/Soltu.DM.06G018840/Soltu.DM.07G014750	3
376	GO:0048766	root hair initiation	12/12756	0.094583426	0.643464333	0.607706097	Soltu.DM.01G042120/Soltu.DM.03G018740/Soltu.DM.04G002690	3
377	GO:0050768	negative regulation of neurogenesis	12/12756	0.094583426	0.643464333	0.607706097	Soltu.DM.12G020370/Soltu.DM.12G020350/Soltu.DM.12G020340	3
378	GO:0052317	camalexin metabolic process	12/12756	0.094583426	0.643464333	0.607706097	Soltu.DM.12G022190/Soltu.DM.06G018840/Soltu.DM.07G014750	3
379	GO:1903533	regulation of protein targeting	12/12756	0.094583426	0.643464333	0.607706097	Soltu.DM.12G020370/Soltu.DM.12G020350/Soltu.DM.12G020340	3
380	GO:0000731	DNA synthesis involved in DNA repair	19/1275	0.0948608	0.6434643	0.6077060	Soltu.DM.08G027160/Soltu.DM.06G015770/Soltu.DM.03G032560/Soltu.DM.02G033290	4

			6	25	33	97		
381	GO:0009395	phospholipid catabolic process	19/12756	0.094860825	0.643464333	0.607706097	Soltu.DM.02G034460/Soltu.DM.01G005590/Soltu.DM.03G024130/Soltu.DM.02G019940	4
382	GO:0055069	zinc ion homeostasis	19/12756	0.094860825	0.643464333	0.607706097	Soltu.DM.07G002440/Soltu.DM.01G035900/Soltu.DM.07G009580/Soltu.DM.01G035910	4
383	GO:0062013	positive regulation of small molecule metabolic process	19/12756	0.094860825	0.643464333	0.607706097	Soltu.DM.02G020950/Soltu.DM.01G035240/Soltu.DM.03G008510/Soltu.DM.10G005360	4
384	GO:1901659	glycosyl compound biosynthetic process	59/12756	0.095774935	0.643464333	0.607706097	Soltu.DM.05G007640/Soltu.DM.03G037170/Soltu.DM.06G018090/Soltu.DM.05G015440/Soltu.DM.02G020870/Soltu.DM.05G007630/Soltu.DM.10G027910/Soltu.DM.09G025040/Soltu.DM.01G017170	9
385	GO:0006665	sphingolipid metabolic process	51/12756	0.099182707	0.643464333	0.607706097	Soltu.DM.04G008710/Soltu.DM.01G042210/Soltu.DM.02G018520/Soltu.DM.08G014180/Soltu.DM.01G047750/Soltu.DM.10G022360/Soltu.DM.10G020020/Soltu.DM.05G012150	8
386	GO:0009640	photomorphogenesis	68/12756	0.099300518	0.643464333	0.607706097	Soltu.DM.11G001010/Soltu.DM.04G036140/Soltu.DM.01G046820/Soltu.DM.08G013580/Soltu.DM.06G023410/Soltu.DM.07G015980/Soltu.DM.01G032120/Soltu.DM.06G021830/Soltu.DM.02G002480/Soltu.DM.02G027780	10
387	GO:1902532	negative regulation of intracellular signal transduction	68/12756	0.099300518	0.643464333	0.607706097	Soltu.DM.11G010230/Soltu.DM.07G020920/Soltu.DM.10G024780/Soltu.DM.11G010220/Soltu.DM.06G014700/Soltu.DM.08G013420/Soltu.DM.08G013440/Soltu.DM.10G022070/Soltu.DM.07G022640/Soltu.DM.05G025620	10
388	GO:0000729	DNA double-strand break processing	6/12756	0.101567379	0.643464333	0.607706097	Soltu.DM.02G001620/Soltu.DM.02G001630	2
389	GO:0001887	selenium compound metabolic process	6/12756	0.101567379	0.643464333	0.607706097	Soltu.DM.03G000340/Soltu.DM.01G041940	2
390	GO:0007064	mitotic sister chromatid cohesion	6/12756	0.101567379	0.643464333	0.607706097	Soltu.DM.06G019850/Soltu.DM.09G014080	2
391	GO:0009094	L-phenylalanine biosynthetic process	6/12756	0.101567379	0.643464333	0.607706097	Soltu.DM.02G020220/Soltu.DM.08G011890	2
392	GO:0019373	epoxygenase P450 pathway	6/12756	0.101567379	0.643464333	0.607706097	Soltu.DM.04G034690/Soltu.DM.10G004300	2
393	GO:0019673	GDP-mannose metabolic process	6/12756	0.101567379	0.643464333	0.607706097	Soltu.DM.02G025970/Soltu.DM.03G027720	2
394	GO:0034720	histone H3-K4 demethylation	6/12756	0.101567379	0.643464333	0.607706097	Soltu.DM.04G006870/Soltu.DM.06G001230	2
395	GO:0046466	membrane lipid catabolic process	6/12756	0.101567379	0.643464333	0.607706097	Soltu.DM.02G018520/Soltu.DM.05G012150	2
396	GO:0046500	S-adenosylmethionine metabolic process	6/12756	0.101567379	0.643464333	0.607706097	Soltu.DM.01G041940/Soltu.DM.12G002620	2
397	GO:0051554	flavonol metabolic process	6/12756	0.101567379	0.643464333	0.607706097	Soltu.DM.01G024670/Soltu.DM.03G021440	2
398	GO:0051555	flavonol biosynthetic process	6/127	0.1015	0.6434	0.6077	Soltu.DM.01G024670/Soltu.DM.03G021440	2

			56	67379	64333	06097		
399	GO:0052390	induction by symbiont of host innate immune response	6/12756	0.101567379	0.643464333	0.607706097	Soltu.DM.01G024680/Soltu.DM.07G014680	2
400	GO:0052559	induction by symbiont of host immune response	6/12756	0.101567379	0.643464333	0.607706097	Soltu.DM.01G024680/Soltu.DM.07G014680	2
401	GO:0061062	regulation of nematode larval development	6/12756	0.101567379	0.643464333	0.607706097	Soltu.DM.09G004090/Soltu.DM.10G024900	2
402	GO:0070070	proton-transporting V-type ATPase complex assembly	6/12756	0.101567379	0.643464333	0.607706097	Soltu.DM.07G009580/Soltu.DM.08G001690	2
403	GO:0070072	vacuolar proton-transporting V-type ATPase complex assembly	6/12756	0.101567379	0.643464333	0.607706097	Soltu.DM.07G009580/Soltu.DM.08G001690	2
404	GO:0097184	response to azide	6/12756	0.101567379	0.643464333	0.607706097	Soltu.DM.09G024260/Soltu.DM.09G024270	2
405	GO:0097185	cellular response to azide	6/12756	0.101567379	0.643464333	0.607706097	Soltu.DM.09G024260/Soltu.DM.09G024270	2
406	GO:1900262	regulation of DNA-directed DNA polymerase activity	6/12756	0.101567379	0.643464333	0.607706097	Soltu.DM.11G009630/Soltu.DM.08G027160	2
407	GO:1900264	positive regulation of DNA-directed DNA polymerase activity	6/12756	0.101567379	0.643464333	0.607706097	Soltu.DM.11G009630/Soltu.DM.08G027160	2
408	GO:1900384	regulation of flavonol biosynthetic process	6/12756	0.101567379	0.643464333	0.607706097	Soltu.DM.01G024670/Soltu.DM.10G017480	2
409	GO:1902223	erythrose 4-phosphate/phosphoenolpyruvate family amino acid biosynthetic process	6/12756	0.101567379	0.643464333	0.607706097	Soltu.DM.02G020220/Soltu.DM.08G011890	2
410	GO:1905034	regulation of antifungal innate immune response	6/12756	0.101567379	0.643464333	0.607706097	Soltu.DM.12G010960/Soltu.DM.03G008510	2
411	GO:0006595	polyamine metabolic process	27/12756	0.1016467	0.643464333	0.607706097	Soltu.DM.07G014310/Soltu.DM.06G014480/Soltu.DM.02G020890/Soltu.DM.01G050280/Soltu.DM.01G027080	5
412	GO:0016137	glycoside metabolic process	27/12756	0.1016467	0.643464333	0.607706097	Soltu.DM.01G042210/Soltu.DM.05G015440/Soltu.DM.09G025040/Soltu.DM.02G008560/Soltu.DM.02G008550	5
413	GO:0008285	negative regulation of cell population proliferation	43/12756	0.101836093	0.643464333	0.607706097	Soltu.DM.01G024670/Soltu.DM.04G033430/Soltu.DM.08G013580/Soltu.DM.05G003100/Soltu.DM.11G016820/Soltu.DM.09G004090/Soltu.DM.10G024900	7
414	GO:0008300	isoprenoid catabolic process	43/12756	0.101836093	0.643464333	0.607706097	Soltu.DM.07G022720/Soltu.DM.07G022710/Soltu.DM.08G020150/Soltu.DM.07G013940/Soltu.DM.07G022700/Soltu.DM.07G013900/Soltu.DM.10G003240	7
415	GO:1900376	regulation of secondary metabolite biosynthetic process	35/12756	0.103077633	0.648388103	0.612356246	Soltu.DM.03G035710/Soltu.DM.07G003530/Soltu.DM.07G003550/Soltu.DM.12G022190/Soltu.DM.02G019030/Soltu.DM.10G017480	6
41	GO:00	response to iron ion	86/	0.1	0.6	0.6	Soltu.DM.01G006210/Soltu.DM.07G028550/Soltu.DM.10G000640/Soltu.DM.07G020920/S	12

6	10039		12756	03118356	48388103	12356246	oltu.DM.04G003430/Soltu.DM.10G024780/Soltu.DM.02G030410/Soltu.DM.08G013420/Soltu.DM.04G034620/Soltu.DM.01G002310/Soltu.DM.10G022070/Soltu.DM.07G022640	
417	GO:0010104	regulation of ethylene-activated signaling pathway	52/12756	0.108153695	0.676748119	0.639140256	Soltu.DM.07G020920/Soltu.DM.10G024780/Soltu.DM.06G014700/Soltu.DM.08G013420/Soltu.DM.08G013440/Soltu.DM.10G022070/Soltu.DM.07G022640/Soltu.DM.05G025620	8
418	GO:0070297	regulation of phosphorelay signal transduction system	52/12756	0.108153695	0.676748119	0.639140256	Soltu.DM.07G020920/Soltu.DM.10G024780/Soltu.DM.06G014700/Soltu.DM.08G013420/Soltu.DM.08G013440/Soltu.DM.10G022070/Soltu.DM.07G022640/Soltu.DM.05G025620	8
419	GO:0009744	response to sucrose	87/12756	0.110018894	0.678042055	0.640362286	Soltu.DM.04G025250/Soltu.DM.07G013360/Soltu.DM.02G025970/Soltu.DM.07G020920/Soltu.DM.10G024780/Soltu.DM.05G006190/Soltu.DM.08G013420/Soltu.DM.05G011970/Soltu.DM.01G024860/Soltu.DM.10G022070/Soltu.DM.12G029710/Soltu.DM.01G047090	12
420	GO:0034285	response to disaccharide	87/12756	0.110018894	0.678042055	0.640362286	Soltu.DM.04G025250/Soltu.DM.07G013360/Soltu.DM.02G025970/Soltu.DM.07G020920/Soltu.DM.10G024780/Soltu.DM.05G006190/Soltu.DM.08G013420/Soltu.DM.05G011970/Soltu.DM.01G024860/Soltu.DM.10G022070/Soltu.DM.12G029710/Soltu.DM.01G047090	12
421	GO:0002213	defense response to insect	20/12756	0.110229737	0.678042055	0.640362286	Soltu.DM.03G024660/Soltu.DM.03G024680/Soltu.DM.03G024670/Soltu.DM.03G024690	4
422	GO:0006690	icosanoid metabolic process	20/12756	0.110229737	0.678042055	0.640362286	Soltu.DM.04G034690/Soltu.DM.10G004300/Soltu.DM.06G034310/Soltu.DM.02G019940	4
423	GO:0007265	Ras protein signal transduction	20/12756	0.110229737	0.678042055	0.640362286	Soltu.DM.12G020370/Soltu.DM.12G020350/Soltu.DM.12G020340/Soltu.DM.12G023230	4
424	GO:0046128	purine ribonucleoside metabolic process	20/12756	0.110229737	0.678042055	0.640362286	Soltu.DM.03G037170/Soltu.DM.10G027910/Soltu.DM.12G003790/Soltu.DM.12G002620	4
425	GO:0060688	regulation of morphogenesis of a branching structure	20/12756	0.110229737	0.678042055	0.640362286	Soltu.DM.06G025210/Soltu.DM.02G023840/Soltu.DM.02G017390/Soltu.DM.03G034300	4
426	GO:0071248	cellular response to metal ion	105/12756	0.110464571	0.678042055	0.640362286	Soltu.DM.01G006210/Soltu.DM.07G028550/Soltu.DM.10G000640/Soltu.DM.07G020920/Soltu.DM.04G003430/Soltu.DM.10G024780/Soltu.DM.02G030410/Soltu.DM.06G024610/Soltu.DM.03G032350/Soltu.DM.08G013420/Soltu.DM.04G034620/Soltu.DM.01G002310/Soltu.DM.10G022070/Soltu.DM.07G022640	14
427	GO:0009718	anthocyanin-containing compound biosynthetic process	28/12756	0.114734329	0.689682992	0.651356319	Soltu.DM.04G001370/Soltu.DM.03G020570/Soltu.DM.09G025040/Soltu.DM.08G026700/Soltu.DM.07G014750	5
428	GO:0043562	cellular response to nitrogen levels	28/12756	0.114734329	0.689682992	0.651356319	Soltu.DM.02G017810/Soltu.DM.10G019110/Soltu.DM.10G012990/Soltu.DM.03G019550/Soltu.DM.11G022310	5
429	GO:0071456	cellular response to hypoxia	28/12756	0.114734329	0.689682992	0.651356319	Soltu.DM.07G011880/Soltu.DM.01G051770/Soltu.DM.11G013880/Soltu.DM.11G011650/Soltu.DM.11G011740	5
430	GO:0009074	aromatic amino acid family catabolic process	13/12756	0.114768814	0.689682992	0.651356319	Soltu.DM.03G035070/Soltu.DM.03G035080/Soltu.DM.04G009170	3
431	GO:0010225	response to UV-C	13/12756	0.114768814	0.689682992	0.651356319	Soltu.DM.02G001620/Soltu.DM.02G001630/Soltu.DM.12G026560	3
432	GO:0019985	translesion synthesis	13/12756	0.114768814	0.689682992	0.651356319	Soltu.DM.08G027160/Soltu.DM.06G015770/Soltu.DM.02G033290	3
433	GO:0042547	cell wall modification involved in multidimensional cell growth	13/12756	0.114768814	0.689682992	0.651356319	Soltu.DM.05G026200/Soltu.DM.01G025270/Soltu.DM.01G040720	3

434	GO:0045666	positive regulation of neuron differentiation	13/12756	0.114768814	0.689682992	0.651356319	Soltu.DM.12G020370/Soltu.DM.12G020350/Soltu.DM.12G020340	3
435	GO:0046417	chorismate metabolic process	13/12756	0.114768814	0.689682992	0.651356319	Soltu.DM.04G018630/Soltu.DM.01G031840/Soltu.DM.01G022690	3
436	GO:2000028	regulation of photoperiodism, flowering	88/12756	0.171824047	0.702549648	0.663507956	Soltu.DM.12G007510/Soltu.DM.04G027760/Soltu.DM.12G025260/Soltu.DM.01G045040/Soltu.DM.04G022240/Soltu.DM.04G006870/Soltu.DM.01G045030/Soltu.DM.05G012040/Soltu.DM.01G024340/Soltu.DM.01G020640/Soltu.DM.01G024940/Soltu.DM.01G045020	12
437	GO:0042545	cell wall modification	62/12756	0.121123084	0.724490208	0.684229316	Soltu.DM.03G035710/Soltu.DM.05G007640/Soltu.DM.05G007630/Soltu.DM.05G026200/Soltu.DM.02G029580/Soltu.DM.09G007590/Soltu.DM.01G025270/Soltu.DM.01G040720/Soltu.DM.07G022640	9
438	GO:0055081	monoatomic anion homeostasis	45/12756	0.122435623	0.729957101	0.689392338	Soltu.DM.02G034460/Soltu.DM.09G002620/Soltu.DM.11G011180/Soltu.DM.10G000600/Soltu.DM.06G005440/Soltu.DM.10G025890/Soltu.DM.10G001460	7
439	GO:0034599	cellular response to oxidative stress	116/12756	0.122647514	0.729957101	0.689392338	Soltu.DM.07G028550/Soltu.DM.10G000640/Soltu.DM.01G047440/Soltu.DM.02G030410/Soltu.DM.02G024520/Soltu.DM.06G024610/Soltu.DM.07G015200/Soltu.DM.03G032350/Soltu.DM.06G012170/Soltu.DM.02G019520/Soltu.DM.02G023590/Soltu.DM.08G011330/Soltu.DM.02G023580/Soltu.DM.06G026560/Soltu.DM.09G005310	15
440	GO:0043648	dicarboxylic acid metabolic process	71/12756	0.123233544	0.729957101	0.689392338	Soltu.DM.08G014620/Soltu.DM.04G018630/Soltu.DM.01G033530/Soltu.DM.01G031840/Soltu.DM.01G022690/Soltu.DM.12G024030/Soltu.DM.03G017660/Soltu.DM.12G026560/Soltu.DM.08G007450/Soltu.DM.06G013150	10
441	GO:0007264	small GTPase mediated signal transduction	21/12756	0.126624227	0.729957101	0.689392338	Soltu.DM.12G020370/Soltu.DM.12G020350/Soltu.DM.12G020340/Soltu.DM.12G023230	4
442	GO:0033866	nucleoside bisphosphate biosynthetic process	21/12756	0.126624227	0.729957101	0.689392338	Soltu.DM.03G037170/Soltu.DM.02G031030/Soltu.DM.11G010590/Soltu.DM.01G019520	4
443	GO:0034030	ribonucleoside bisphosphate biosynthetic process	21/12756	0.126624227	0.729957101	0.689392338	Soltu.DM.03G037170/Soltu.DM.02G031030/Soltu.DM.11G010590/Soltu.DM.01G019520	4
444	GO:0034033	purine nucleoside bisphosphate biosynthetic process	21/12756	0.126624227	0.729957101	0.689392338	Soltu.DM.03G037170/Soltu.DM.02G031030/Soltu.DM.11G010590/Soltu.DM.01G019520	4
445	GO:0034471	ncRNA 5'-end processing	21/12756	0.126624227	0.729957101	0.689392338	Soltu.DM.12G016740/Soltu.DM.01G051600/Soltu.DM.11G004920/Soltu.DM.06G029830	4
446	GO:0034644	cellular response to UV	21/12756	0.126624227	0.729957101	0.689392338	Soltu.DM.09G025070/Soltu.DM.02G001620/Soltu.DM.02G001630/Soltu.DM.12G026560	4
447	GO:0048639	positive regulation of developmental growth	21/12756	0.126624227	0.729957101	0.689392338	Soltu.DM.12G020370/Soltu.DM.12G020350/Soltu.DM.08G011890/Soltu.DM.12G020340	4
448	GO:2000035	regulation of stem cell division	21/12756	0.126624227	0.729957101	0.689392338	Soltu.DM.07G020920/Soltu.DM.10G024780/Soltu.DM.08G013420/Soltu.DM.10G022070	4
449	GO:0042326	negative regulation of phosphorylation	29/12756	0.128579269	0.729957101	0.689392338	Soltu.DM.07G012130/Soltu.DM.11G010230/Soltu.DM.11G010220/Soltu.DM.06G028580/Soltu.DM.04G038280	5
450	GO:0046351	disaccharide biosynthetic process	29/12756	0.128579269	0.729957101	0.689392338	Soltu.DM.04G012960/Soltu.DM.02G015310/Soltu.DM.07G001730/Soltu.DM.05G008060/Soltu.DM.07G014750	5
451	GO:0045088	regulation of innate immune response	164/127	0.1312116	0.7299571	0.6893923	Soltu.DM.02G025590/Soltu.DM.10G027770/Soltu.DM.02G014980/Soltu.DM.07G028550/Soltu.DM.01G024680/Soltu.DM.03G036010/Soltu.DM.02G015010/Soltu.DM.10G000640/Soltu.DM.04G018070/Soltu.DM.08G022900/Soltu.DM.02G014950/Soltu.DM.05G012690/Solt	20

			56	77	01	38	u.DM.08G011890/Soltu.DM.02G022460/Soltu.DM.01G051770/Soltu.DM.12G010960/Soltu.DM.03G008510/Soltu.DM.08G020460/Soltu.DM.02G026820/Soltu.DM.05G023030	
452	GO:0009686	gibberellin biosynthetic process	72/12756	0.131855883	0.729957101	0.689392338	Soltu.DM.07G020920/Soltu.DM.02G011120/Soltu.DM.10G024780/Soltu.DM.06G012790/Soltu.DM.08G013420/Soltu.DM.06G004460/Soltu.DM.10G003570/Soltu.DM.10G022070/Soltu.DM.06G023440/Soltu.DM.10G003550	10
453	GO:0046364	monosaccharide biosynthetic process	46/12756	0.133474652	0.729957101	0.689392338	Soltu.DM.09G018910/Soltu.DM.02G025970/Soltu.DM.04G031580/Soltu.DM.02G012570/Soltu.DM.12G002640/Soltu.DM.06G009750/Soltu.DM.01G045760	7
454	GO:0001504	neurotransmitter uptake	7/12756	0.133646141	0.729957101	0.689392338	Soltu.DM.04G031760/Soltu.DM.12G006380	2
455	GO:0003376	sphingosine-1-phosphate receptor signaling pathway	7/12756	0.133646141	0.729957101	0.689392338	Soltu.DM.02G018520/Soltu.DM.08G011890	2
456	GO:0006208	pyrimidine nucleobase catabolic process	7/12756	0.133646141	0.729957101	0.689392338	Soltu.DM.10G012990/Soltu.DM.03G019550	2
457	GO:0006212	uracil catabolic process	7/12756	0.133646141	0.729957101	0.689392338	Soltu.DM.10G012990/Soltu.DM.03G019550	2
458	GO:0006954	inflammatory response	7/12756	0.133646141	0.729957101	0.689392338	Soltu.DM.02G017970/Soltu.DM.10G004300	2
459	GO:0007231	osmosensory signaling pathway	7/12756	0.133646141	0.729957101	0.689392338	Soltu.DM.07G020410/Soltu.DM.08G028440	2
460	GO:0009095	aromatic amino acid family biosynthetic process, prephenate pathway	7/12756	0.133646141	0.729957101	0.689392338	Soltu.DM.02G020220/Soltu.DM.08G011890	2
461	GO:0010231	maintenance of seed dormancy	7/12756	0.133646141	0.729957101	0.689392338	Soltu.DM.03G013100/Soltu.DM.07G024500	2
462	GO:0010304	PSII associated light-harvesting complex II catabolic process	7/12756	0.133646141	0.729957101	0.689392338	Soltu.DM.07G010570/Soltu.DM.04G037460	2
463	GO:0016233	telomere capping	7/12756	0.133646141	0.729957101	0.689392338	Soltu.DM.05G023970/Soltu.DM.02G013390	2
464	GO:0019336	phenol-containing compound catabolic process	7/12756	0.133646141	0.729957101	0.689392338	Soltu.DM.06G028410/Soltu.DM.04G009170	2
465	GO:0019632	shikimate metabolic process	7/12756	0.133646141	0.729957101	0.689392338	Soltu.DM.04G023360/Soltu.DM.01G022690	2
466	GO:0019860	uracil metabolic process	7/12756	0.133646141	0.729957101	0.689392338	Soltu.DM.10G012990/Soltu.DM.03G019550	2
467	GO:0042793	plastid transcription	7/12756	0.133646141	0.729957101	0.689392338	Soltu.DM.02G013430/Soltu.DM.02G027620	2
468	GO:0043407	negative regulation of MAP kinase activity	7/12756	0.133646141	0.729957101	0.689392338	Soltu.DM.11G010230/Soltu.DM.11G010220	2
46	GO:00	linoleic acid metabolic process	7/1	0.1	0.7	0.6	Soltu.DM.02G017970/Soltu.DM.02G019940	2

9	43651		27 56	336 461 41	299 571 01	893 923 38		
470	GO:0071918	urea transmembrane transport	7/127 56	0.1336 461 41	0.7299 571 01	0.6893 923 38	Soltu.DM.06G031120/Soltu.DM.03G031200	2
471	GO:0089718	amino acid import across plasma membrane	7/127 56	0.1336 461 41	0.7299 571 01	0.6893 923 38	Soltu.DM.04G031760/Soltu.DM.12G006380	2
472	GO:0090520	sphingolipid mediated signaling pathway	7/127 56	0.1336 461 41	0.7299 571 01	0.6893 923 38	Soltu.DM.02G018520/Soltu.DM.08G011890	2
473	GO:0097437	maintenance of dormancy	7/127 56	0.1336 461 41	0.7299 571 01	0.6893 923 38	Soltu.DM.03G013100/Soltu.DM.07G024500	2
474	GO:0097501	stress response to metal ion	7/127 56	0.1336 461 41	0.7299 571 01	0.6893 923 38	Soltu.DM.07G028550/Soltu.DM.10G000640	2
475	GO:0098712	L-glutamate import across plasma membrane	7/127 56	0.1336 461 41	0.7299 571 01	0.6893 923 38	Soltu.DM.04G031760/Soltu.DM.12G006380	2
476	GO:0110126	phloem loading	7/127 56	0.1336 461 41	0.7299 571 01	0.6893 923 38	Soltu.DM.03G027330/Soltu.DM.03G027340	2
477	GO:1900367	positive regulation of defense response to insect	7/127 56	0.1336 461 41	0.7299 571 01	0.6893 923 38	Soltu.DM.02G025590/Soltu.DM.03G008510	2
478	GO:2000010	positive regulation of protein localization to cell surface	7/127 56	0.1336 461 41	0.7299 571 01	0.6893 923 38	Soltu.DM.07G002400/Soltu.DM.08G001690	2
479	GO:0010112	regulation of systemic acquired resistance	14/12756	0.1363 984 13	0.7341 025 24	0.6933 073 93	Soltu.DM.10G027770/Soltu.DM.03G036010/Soltu.DM.05G012690	3
480	GO:0016559	peroxisome fission	14/12756	0.1363 984 13	0.7341 025 24	0.6933 073 93	Soltu.DM.01G033530/Soltu.DM.12G001180/Soltu.DM.10G007000	3
481	GO:0052314	phytoalexin metabolic process	14/12756	0.1363 984 13	0.7341 025 24	0.6933 073 93	Soltu.DM.12G022190/Soltu.DM.06G018840/Soltu.DM.07G014750	3
482	GO:0052315	phytoalexin biosynthetic process	14/12756	0.1363 984 13	0.7341 025 24	0.6933 073 93	Soltu.DM.12G022190/Soltu.DM.06G018840/Soltu.DM.07G014750	3
483	GO:0071490	cellular response to far red light	14/12756	0.1363 984 13	0.7341 025 24	0.6933 073 93	Soltu.DM.09G025070/Soltu.DM.07G028470/Soltu.DM.02G011380	3
484	GO:0099116	tRNA 5'-end processing	14/12756	0.1363 984 13	0.7341 025 24	0.6933 073 93	Soltu.DM.12G016740/Soltu.DM.11G004920/Soltu.DM.06G029830	3
485	GO:1901642	nucleoside transmembrane transport	14/12756	0.1363 984 13	0.7341 025 24	0.6933 073 93	Soltu.DM.04G030440/Soltu.DM.10G000530/Soltu.DM.06G012690	3
486	GO:0007568	aging	165/127 56	0.1369 483 7	0.7355 268 7	0.6946 525 86	Soltu.DM.06G028410/Soltu.DM.05G026160/Soltu.DM.03G037070/Soltu.DM.12G027330/Soltu.DM.06G022310/Soltu.DM.03G017570/Soltu.DM.10G010160/Soltu.DM.03G037170/Soltu.DM.02G018270/Soltu.DM.03G016080/Soltu.DM.02G014410/Soltu.DM.05G024870/Soltu.DM.12G003790/Soltu.DM.05G011970/Soltu.DM.11G022310/Soltu.DM.03G033450/Soltu.	20

							DM.01G007500/Soltu.DM.02G014440/Soltu.DM.07G014750/Soltu.DM.06G010530	
487	GO:0006778	porphyrin-containing compound metabolic process	55/12756	0.137607365	0.735999561	0.695099009	Soltu.DM.02G034170/Soltu.DM.08G013640/Soltu.DM.07G010570/Soltu.DM.08G028310/Soltu.DM.04G031570/Soltu.DM.12G026560/Soltu.DM.09G027920/Soltu.DM.06G002140	8
488	GO:0035821	modulation of process of another organism	55/12756	0.137607365	0.735999561	0.695099009	Soltu.DM.01G024680/Soltu.DM.02G012280/Soltu.DM.05G026810/Soltu.DM.07G014680/Soltu.DM.01G005590/Soltu.DM.03G024130/Soltu.DM.01G010020/Soltu.DM.11G004150	8
489	GO:0005996	monosaccharide metabolic process	91/12756	0.140225474	0.748449838	0.706857407	Soltu.DM.09G018910/Soltu.DM.01G040570/Soltu.DM.02G031920/Soltu.DM.02G025970/Soltu.DM.04G031580/Soltu.DM.02G031890/Soltu.DM.09G023470/Soltu.DM.02G012570/Soltu.DM.02G024820/Soltu.DM.12G002640/Soltu.DM.06G009750/Soltu.DM.01G045760	12
490	GO:0032434	regulation of proteasomal ubiquitin-dependent protein catabolic process	30/12756	0.143141563	0.760863637	0.718581354	Soltu.DM.12G005510/Soltu.DM.06G015770/Soltu.DM.04G034740/Soltu.DM.09G005140/Soltu.DM.06G024530	5
491	GO:1901658	glycosyl compound catabolic process	30/12756	0.143141563	0.760863637	0.718581354	Soltu.DM.03G035710/Soltu.DM.01G042210/Soltu.DM.12G003790/Soltu.DM.02G008560/Soltu.DM.02G008550	5
492	GO:0120256	olefinic compound catabolic process	22/12756	0.143966856	0.763676039	0.721237466	Soltu.DM.08G020150/Soltu.DM.07G013940/Soltu.DM.07G013900/Soltu.DM.10G004300	4
493	GO:0042364	water-soluble vitamin biosynthetic process	47/12756	0.144985945	0.765929848	0.723366028	Soltu.DM.04G025250/Soltu.DM.09G006800/Soltu.DM.02G025970/Soltu.DM.06G009750/Soltu.DM.01G045760/Soltu.DM.06G003240/Soltu.DM.01G044760	7
494	GO:0080050	regulation of seed development	47/12756	0.144985945	0.765929848	0.723366028	Soltu.DM.05G024870/Soltu.DM.07G010930/Soltu.DM.11G002420/Soltu.DM.01G040220/Soltu.DM.05G021010/Soltu.DM.03G036780/Soltu.DM.01G024940	7
495	GO:0033013	tetrapyrrole metabolic process	56/12756	0.148238762	0.780829579	0.73743776	Soltu.DM.02G034170/Soltu.DM.08G013640/Soltu.DM.07G010570/Soltu.DM.08G028310/Soltu.DM.04G031570/Soltu.DM.12G026560/Soltu.DM.09G027920/Soltu.DM.06G002140	8
496	GO:0006575	cellular modified amino acid metabolic process	92/12756	0.148412139	0.780829579	0.73743776	Soltu.DM.04G025250/Soltu.DM.07G022460/Soltu.DM.07G022500/Soltu.DM.09G006800/Soltu.DM.07G022530/Soltu.DM.07G022490/Soltu.DM.10G003300/Soltu.DM.12G002620/Soltu.DM.07G022450/Soltu.DM.09G001260/Soltu.DM.12G028960/Soltu.DM.01G028030	12
497	GO:0071369	cellular response to ethylene stimulus	83/12756	0.149514812	0.785028891	0.741403711	Soltu.DM.01G006210/Soltu.DM.07G028550/Soltu.DM.10G000640/Soltu.DM.07G020090/Soltu.DM.02G030410/Soltu.DM.08G028440/Soltu.DM.05G024870/Soltu.DM.01G002310/Soltu.DM.09G026500/Soltu.DM.02G017280/Soltu.DM.07G022640	11
498	GO:0010208	pollen wall assembly	39/12756	0.152130834	0.795523915	0.751315551	Soltu.DM.07G000410/Soltu.DM.03G020790/Soltu.DM.05G025440/Soltu.DM.04G022240/Soltu.DM.02G011180/Soltu.DM.02G031030	6
499	GO:0085029	extracellular matrix assembly	39/12756	0.152130834	0.795523915	0.751315551	Soltu.DM.07G000410/Soltu.DM.03G020790/Soltu.DM.05G025440/Soltu.DM.04G022240/Soltu.DM.02G011180/Soltu.DM.02G031030	6
500	GO:0009967	positive regulation of signal transduction	149/12756	0.155335863	0.810639383	0.76559099	Soltu.DM.09G018310/Soltu.DM.07G017210/Soltu.DM.07G017190/Soltu.DM.07G017200/Soltu.DM.04G033590/Soltu.DM.02G018520/Soltu.DM.07G017180/Soltu.DM.04G033180/Soltu.DM.09G028490/Soltu.DM.04G037130/Soltu.DM.12G025260/Soltu.DM.09G022610/Soltu.DM.03G021360/Soltu.DM.08G011890/Soltu.DM.02G013430/Soltu.DM.09G026500/Soltu.DM.03G036780/Soltu.DM.02G026820	18
501	GO:0001505	regulation of neurotransmitter levels	48/12756	0.15695142	0.8156193	0.770294165	Soltu.DM.04G031760/Soltu.DM.08G013400/Soltu.DM.12G006380/Soltu.DM.08G013620/Soltu.DM.12G024030/Soltu.DM.06G034310/Soltu.DM.11G026460	7
502	GO:0006835	dicarboxylic acid transport	31/12756	0.158376899	0.8156193	0.770294165	Soltu.DM.05G003990/Soltu.DM.04G031760/Soltu.DM.12G006380/Soltu.DM.11G000570/Soltu.DM.05G021160	5
503	GO:0009073	aromatic amino acid family biosynthetic process	31/12756	0.158376899	0.8156193	0.770294165	Soltu.DM.04G023360/Soltu.DM.04G018630/Soltu.DM.02G031330/Soltu.DM.02G020220/Soltu.DM.08G011890	5
50	GO:20	regulation of ubiquitin-dependent	31/	0.1	0.8	0.7	Soltu.DM.12G005510/Soltu.DM.06G015770/Soltu.DM.04G034740/Soltu.DM.09G005140/S	5

4	00058	protein catabolic process	12756	58376899	156193	70294165	oltu.DM.06G024530	
505	GO:0010161	red light signaling pathway	15/12756	0.159278224	0.8156193	0.770294165	Soltu.DM.01G035240/Soltu.DM.10G005360/Soltu.DM.06G002140	3
506	GO:0015858	nucleoside transport	15/12756	0.159278224	0.8156193	0.770294165	Soltu.DM.04G030440/Soltu.DM.10G000530/Soltu.DM.06G012690	3
507	GO:0031346	positive regulation of cell projection organization	15/12756	0.159278224	0.8156193	0.770294165	Soltu.DM.12G020370/Soltu.DM.12G020350/Soltu.DM.12G020340	3
508	GO:0010224	response to UV-B	75/12756	0.159575777	0.8156193	0.770294165	Soltu.DM.03G019450/Soltu.DM.01G024670/Soltu.DM.09G025070/Soltu.DM.02G024380/Soltu.DM.07G011650/Soltu.DM.07G011670/Soltu.DM.10G017480/Soltu.DM.07G011660/Soltu.DM.05G007660/Soltu.DM.05G003920	10
509	GO:0019747	regulation of isoprenoid metabolic process	23/12756	0.162174372	0.8156193	0.770294165	Soltu.DM.02G020950/Soltu.DM.08G013580/Soltu.DM.01G035240/Soltu.DM.10G005360	4
510	GO:0043650	dicarboxylic acid biosynthetic process	23/12756	0.162174372	0.8156193	0.770294165	Soltu.DM.04G018630/Soltu.DM.01G031840/Soltu.DM.01G022690/Soltu.DM.12G026560	4
511	GO:0052386	cell wall thickening	23/12756	0.162174372	0.8156193	0.770294165	Soltu.DM.03G035710/Soltu.DM.05G007640/Soltu.DM.05G007630/Soltu.DM.07G022640	4
512	GO:0052543	callose deposition in cell wall	23/12756	0.162174372	0.8156193	0.770294165	Soltu.DM.03G035710/Soltu.DM.05G007640/Soltu.DM.05G007630/Soltu.DM.07G022640	4
513	GO:2000573	positive regulation of DNA biosynthetic process	23/12756	0.162174372	0.8156193	0.770294165	Soltu.DM.06G026960/Soltu.DM.11G009630/Soltu.DM.08G027160/Soltu.DM.02G013390	4
514	GO:0009739	response to gibberellin	160/12756	0.165082531	0.8156193	0.770294165	Soltu.DM.10G020990/Soltu.DM.12G001040/Soltu.DM.05G026870/Soltu.DM.04G033180/Soltu.DM.02G011120/Soltu.DM.09G022610/Soltu.DM.06G012790/Soltu.DM.08G011890/Soltu.DM.02G004510/Soltu.DM.09G001260/Soltu.DM.11G026620/Soltu.DM.06G020450/Soltu.DM.04G033700/Soltu.DM.01G024340/Soltu.DM.01G028030/Soltu.DM.02G009820/Soltu.DM.06G002140/Soltu.DM.06G023440/Soltu.DM.07G022640	19
515	GO:0009312	oligosaccharide biosynthetic process	40/12756	0.165777044	0.8156193	0.770294165	Soltu.DM.04G012960/Soltu.DM.02G015310/Soltu.DM.07G001730/Soltu.DM.09G022610/Soltu.DM.05G008060/Soltu.DM.07G014750	6
516	GO:0000304	response to singlet oxygen	8/12756	0.167578068	0.8156193	0.770294165	Soltu.DM.06G026400/Soltu.DM.06G026560	2
517	GO:0005513	detection of calcium ion	8/12756	0.167578068	0.8156193	0.770294165	Soltu.DM.10G026220/Soltu.DM.12G021010	2
518	GO:0006656	phosphatidylcholine biosynthetic process	8/12756	0.167578068	0.8156193	0.770294165	Soltu.DM.03G030800/Soltu.DM.06G018040	2
519	GO:0006808	regulation of nitrogen utilization	8/12756	0.167578068	0.8156193	0.770294165	Soltu.DM.01G008040/Soltu.DM.05G001260	2
520	GO:0006949	syncytium formation	8/12756	0.167578068	0.8156193	0.770294165	Soltu.DM.07G019710/Soltu.DM.06G011420	2
521	GO:0009961	response to 1-aminocyclopropane-1-carboxylic acid	8/12756	0.1675780	0.8156193	0.7702941	Soltu.DM.02G024660/Soltu.DM.10G000560	2

				68		65		
522	GO:0010215	cellulose microfibril organization	8/12756	0.167578068	0.8156193	0.770294165	Soltu.DM.02G009140/Soltu.DM.04G022240	2
523	GO:0010469	regulation of signaling receptor activity	8/12756	0.167578068	0.8156193	0.770294165	Soltu.DM.01G039230/Soltu.DM.02G028750	2
524	GO:0031407	oxylipin metabolic process	8/12756	0.167578068	0.8156193	0.770294165	Soltu.DM.04G034690/Soltu.DM.07G003270	2
525	GO:0033120	positive regulation of RNA splicing	8/12756	0.167578068	0.8156193	0.770294165	Soltu.DM.12G005490/Soltu.DM.12G025260	2
526	GO:0033499	galactose catabolic process via UDP-galactose	8/12756	0.167578068	0.8156193	0.770294165	Soltu.DM.02G031920/Soltu.DM.02G031890	2
527	GO:0042135	neurotransmitter catabolic process	8/12756	0.167578068	0.8156193	0.770294165	Soltu.DM.08G013400/Soltu.DM.12G024030	2
528	GO:0042451	purine nucleoside biosynthetic process	8/12756	0.167578068	0.8156193	0.770294165	Soltu.DM.03G037170/Soltu.DM.10G027910	2
529	GO:0043409	negative regulation of MAPK cascade	8/12756	0.167578068	0.8156193	0.770294165	Soltu.DM.11G010230/Soltu.DM.11G010220	2
530	GO:0046129	purine ribonucleoside biosynthetic process	8/12756	0.167578068	0.8156193	0.770294165	Soltu.DM.03G037170/Soltu.DM.10G027910	2
531	GO:0070071	proton-transporting two-sector ATPase complex assembly	8/12756	0.167578068	0.8156193	0.770294165	Soltu.DM.07G009580/Soltu.DM.08G001690	2
532	GO:0071277	cellular response to calcium ion	8/12756	0.167578068	0.8156193	0.770294165	Soltu.DM.06G024610/Soltu.DM.03G032350	2
533	GO:1901348	positive regulation of secondary cell wall biogenesis	8/12756	0.167578068	0.8156193	0.770294165	Soltu.DM.11G011390/Soltu.DM.01G027520	2
534	GO:1903340	positive regulation of cell wall organization or biogenesis	8/12756	0.167578068	0.8156193	0.770294165	Soltu.DM.11G011390/Soltu.DM.01G027520	2
535	GO:0009867	jasmonic acid mediated signaling pathway	85/12756	0.167679685	0.8156193	0.770294165	Soltu.DM.02G025590/Soltu.DM.07G024710/Soltu.DM.07G012950/Soltu.DM.09G024260/Soltu.DM.03G032770/Soltu.DM.12G029330/Soltu.DM.02G022460/Soltu.DM.01G000760/Soltu.DM.07G024240/Soltu.DM.01G017870/Soltu.DM.09G024270	11
536	GO:0048467	gynoecium development	85/12756	0.167679685	0.8156193	0.770294165	Soltu.DM.03G024660/Soltu.DM.03G024680/Soltu.DM.03G024670/Soltu.DM.10G023790/Soltu.DM.06G018130/Soltu.DM.02G003130/Soltu.DM.05G003100/Soltu.DM.03G024690/Soltu.DM.01G000910/Soltu.DM.04G022240/Soltu.DM.04G033440	11
537	GO:0042752	regulation of circadian rhythm	49/12756	0.169351094	0.822197969	0.765072749	Soltu.DM.06G024250/Soltu.DM.11G001010/Soltu.DM.12G007510/Soltu.DM.04G027760/Soltu.DM.12G025260/Soltu.DM.03G005580/Soltu.DM.02G011380	7
538	GO:0015718	monocarboxylic acid transport	67/12756	0.170499111	0.826215615	0.780301628	Soltu.DM.05G003990/Soltu.DM.09G028710/Soltu.DM.01G008290/Soltu.DM.06G005370/Soltu.DM.01G034820/Soltu.DM.03G024040/Soltu.DM.11G011430/Soltu.DM.02G019940/Soltu.DM.11G021200	9
539	GO:0045454	cell redox homeostasis	32/12	0.1742	0.8427	0.7959	Soltu.DM.01G047440/Soltu.DM.02G024520/Soltu.DM.02G019520/Soltu.DM.01G047270/Soltu.DM.09G005310	5

			756	37646	47939	15228		
540	GO:0071241	cellular response to inorganic substance	133/12756	0.17680498	0.853564115	0.806130332	Soltu.DM.01G006210/Soltu.DM.07G028550/Soltu.DM.09G024260/Soltu.DM.10G000640/Soltu.DM.07G020920/Soltu.DM.04G003430/Soltu.DM.10G024780/Soltu.DM.02G030410/Soltu.DM.06G024610/Soltu.DM.03G032350/Soltu.DM.08G013420/Soltu.DM.04G034620/Soltu.DM.01G002310/Soltu.DM.10G022070/Soltu.DM.09G024270/Soltu.DM.07G022640	16
541	GO:0009627	systemic acquired resistance	86/12756	0.17714868	0.853624856	0.806187698	Soltu.DM.03G035710/Soltu.DM.08G015610/Soltu.DM.06G026400/Soltu.DM.03G028930/Soltu.DM.04G028540/Soltu.DM.07G014680/Soltu.DM.01G051770/Soltu.DM.02G008560/Soltu.DM.07G006510/Soltu.DM.09G023400/Soltu.DM.02G008550	11
542	GO:0016102	diterpenoid biosynthetic process	77/12756	0.179520916	0.861909876	0.814012308	Soltu.DM.07G020920/Soltu.DM.02G011120/Soltu.DM.10G024780/Soltu.DM.06G012790/Soltu.DM.08G013420/Soltu.DM.06G004460/Soltu.DM.10G003570/Soltu.DM.10G022070/Soltu.DM.06G023440/Soltu.DM.10G003550	10
543	GO:0045488	pectin metabolic process	41/12756	0.17991813	0.861909876	0.814012308	Soltu.DM.02G031090/Soltu.DM.07G000930/Soltu.DM.02G031050/Soltu.DM.04G029850/Soltu.DM.06G021870/Soltu.DM.09G007590	6
544	GO:0000054	ribosomal subunit export from nucleus	24/12756	0.181159423	0.861909876	0.814012308	Soltu.DM.12G024350/Soltu.DM.09G005370/Soltu.DM.11G024760/Soltu.DM.07G006510	4
545	GO:0010229	inflorescence development	24/12756	0.181159423	0.861909876	0.814012308	Soltu.DM.06G019760/Soltu.DM.12G026560/Soltu.DM.08G001470/Soltu.DM.08G012010	4
546	GO:0033750	ribosome localization	24/12756	0.181159423	0.861909876	0.814012308	Soltu.DM.12G024350/Soltu.DM.09G005370/Soltu.DM.11G024760/Soltu.DM.07G006510	4
547	GO:0006767	water-soluble vitamin metabolic process	50/12756	0.182163266	0.861909876	0.814012308	Soltu.DM.04G025250/Soltu.DM.09G006800/Soltu.DM.02G025970/Soltu.DM.06G009750/Soltu.DM.01G045760/Soltu.DM.06G003240/Soltu.DM.01G044760	7
548	GO:0030198	extracellular matrix organization	50/12756	0.182163266	0.861909876	0.814012308	Soltu.DM.07G000410/Soltu.DM.02G009140/Soltu.DM.03G020790/Soltu.DM.05G025440/Soltu.DM.04G022240/Soltu.DM.02G011180/Soltu.DM.02G031030	7
549	GO:0043062	extracellular structure organization	50/12756	0.182163266	0.861909876	0.814012308	Soltu.DM.07G000410/Soltu.DM.02G009140/Soltu.DM.03G020790/Soltu.DM.05G025440/Soltu.DM.04G022240/Soltu.DM.02G011180/Soltu.DM.02G031030	7
550	GO:0000082	G1/S transition of mitotic cell cycle	16/12756	0.183214357	0.861909876	0.814012308	Soltu.DM.03G003730/Soltu.DM.02G029740/Soltu.DM.01G047090	3
551	GO:0008272	sulfate transport	16/12756	0.183214357	0.861909876	0.814012308	Soltu.DM.09G020160/Soltu.DM.07G020410/Soltu.DM.05G021160	3
552	GO:0009268	response to pH	16/12756	0.183214357	0.861909876	0.814012308	Soltu.DM.06G002800/Soltu.DM.06G032750/Soltu.DM.06G020020	3
553	GO:0019852	L-ascorbic acid metabolic process	16/12756	0.183214357	0.861909876	0.814012308	Soltu.DM.02G025970/Soltu.DM.06G009750/Soltu.DM.01G045760	3
554	GO:0019853	L-ascorbic acid biosynthetic process	16/12756	0.183214357	0.861909876	0.814012308	Soltu.DM.02G025970/Soltu.DM.06G009750/Soltu.DM.01G045760	3
555	GO:0033014	tetrapyrrole biosynthetic process	33/12756	0.190673218	0.893737376	0.844071108	Soltu.DM.08G013640/Soltu.DM.08G028310/Soltu.DM.04G031570/Soltu.DM.12G026560/Soltu.DM.06G002140	5
556	GO:2000278	regulation of DNA biosynthetic process	33/12756	0.190673218	0.893737376	0.844071108	Soltu.DM.06G026960/Soltu.DM.11G009630/Soltu.DM.08G027160/Soltu.DM.05G006310/Soltu.DM.02G013390	5
55	GO:00	regulation of immune response	19	0.1	0.8	0.8	Soltu.DM.02G025590/Soltu.DM.10G027770/Soltu.DM.02G014980/Soltu.DM.07G028550/S	22

7	50776		3/12756	914466	93779252	44110657	oltu.DM.01G024680/Soltu.DM.03G036010/Soltu.DM.02G015010/Soltu.DM.10G000640/Soltu.DM.04G018070/Soltu.DM.08G022900/Soltu.DM.02G014950/Soltu.DM.05G012690/Soltu.DM.08G011890/Soltu.DM.04G022240/Soltu.DM.07G014680/Soltu.DM.02G022460/Soltu.DM.01G051770/Soltu.DM.12G010960/Soltu.DM.03G008510/Soltu.DM.08G020460/Soltu.DM.02G026820/Soltu.DM.05G023030	
558	GO:0015748	organophosphate ester transport	60/12756	0.194472064	0.893779252	0.844110657	Soltu.DM.01G038470/Soltu.DM.01G008290/Soltu.DM.06G024610/Soltu.DM.06G022490/Soltu.DM.03G032350/Soltu.DM.12G024350/Soltu.DM.03G034530/Soltu.DM.06G025410	8
559	GO:0015994	chlorophyll metabolic process	42/12756	0.194521044	0.893779252	0.844110657	Soltu.DM.02G034170/Soltu.DM.07G010570/Soltu.DM.08G028310/Soltu.DM.04G031570/Soltu.DM.09G027920/Soltu.DM.06G002140	6
560	GO:0009082	branched-chain amino acid biosynthetic process	25/12756	0.200832096	0.893779252	0.844110657	Soltu.DM.03G005810/Soltu.DM.06G018090/Soltu.DM.07G023080/Soltu.DM.11G003850	4
561	GO:0010315	auxin export across the plasma membrane	25/12756	0.200832096	0.893779252	0.844110657	Soltu.DM.02G032050/Soltu.DM.10G026500/Soltu.DM.02G022410/Soltu.DM.04G002690	4
562	GO:1900618	regulation of shoot system morphogenesis	25/12756	0.200832096	0.893779252	0.844110657	Soltu.DM.06G025210/Soltu.DM.02G023840/Soltu.DM.02G017390/Soltu.DM.03G034300	4
563	GO:0062012	regulation of small molecule metabolic process	98/12756	0.202476976	0.893779252	0.844110657	Soltu.DM.03G035070/Soltu.DM.02G020950/Soltu.DM.08G013580/Soltu.DM.09G031790/Soltu.DM.10G004300/Soltu.DM.03G035080/Soltu.DM.01G035240/Soltu.DM.03G008510/Soltu.DM.11G018040/Soltu.DM.10G005360/Soltu.DM.07G024240/Soltu.DM.09G023400	12
564	GO:0002792	negative regulation of peptide secretion	9/12756	0.20273744	0.893779252	0.844110657	Soltu.DM.08G027150/Soltu.DM.09G002090	2
565	GO:0006670	sphingosine metabolic process	9/12756	0.20273744	0.893779252	0.844110657	Soltu.DM.02G018520/Soltu.DM.08G014180	2
566	GO:0009831	plant-type cell wall modification involved in multidimensional cell growth	9/12756	0.20273744	0.893779252	0.844110657	Soltu.DM.01G025270/Soltu.DM.01G040720	2
567	GO:0010344	seed oilbody biogenesis	9/12756	0.20273744	0.893779252	0.844110657	Soltu.DM.06G012350/Soltu.DM.12G028510	2
568	GO:0015940	pantothenate biosynthetic process	9/12756	0.20273744	0.893779252	0.844110657	Soltu.DM.04G025250/Soltu.DM.09G006800	2
569	GO:0033539	fatty acid beta-oxidation using acyl-CoA dehydrogenase	9/12756	0.20273744	0.893779252	0.844110657	Soltu.DM.08G024620/Soltu.DM.10G003920	2
570	GO:0045292	mRNA cis splicing, via spliceosome	9/12756	0.20273744	0.893779252	0.844110657	Soltu.DM.02G021990/Soltu.DM.10G001400	2
571	GO:0046512	sphingosine biosynthetic process	9/12756	0.20273744	0.893779252	0.844110657	Soltu.DM.02G018520/Soltu.DM.08G014180	2
572	GO:0048578	positive regulation of long-day photoperiodism, flowering	9/12756	0.20273744	0.893779252	0.844110657	Soltu.DM.12G007510/Soltu.DM.04G027760	2
573	GO:0050709	negative regulation of protein secretion	9/12756	0.20273744	0.893779252	0.844110657	Soltu.DM.08G027150/Soltu.DM.09G002090	2
574	GO:0051048	negative regulation of secretion	9/127	0.2027	0.8937	0.8441	Soltu.DM.08G027150/Soltu.DM.09G002090	2

			56	3744	79252	10657		
575	GO:0052031	modulation by symbiont of host defense response	9/12756	0.20273744	0.893779252	0.844110657	Soltu.DM.01G024680/Soltu.DM.07G014680	2
576	GO:0052167	modulation by symbiont of host innate immune response	9/12756	0.20273744	0.893779252	0.844110657	Soltu.DM.01G024680/Soltu.DM.07G014680	2
577	GO:0052173	response to defenses of other organism	9/12756	0.20273744	0.893779252	0.844110657	Soltu.DM.01G024680/Soltu.DM.07G014680	2
578	GO:0052200	response to host defenses	9/12756	0.20273744	0.893779252	0.844110657	Soltu.DM.01G024680/Soltu.DM.07G014680	2
579	GO:0052553	modulation by symbiont of host immune response	9/12756	0.20273744	0.893779252	0.844110657	Soltu.DM.01G024680/Soltu.DM.07G014680	2
580	GO:0052572	response to host immune response	9/12756	0.20273744	0.893779252	0.844110657	Soltu.DM.01G024680/Soltu.DM.07G014680	2
581	GO:0070206	protein trimerization	9/12756	0.20273744	0.893779252	0.844110657	Soltu.DM.03G000340/Soltu.DM.02G032340	2
582	GO:0070861	regulation of protein exit from endoplasmic reticulum	9/12756	0.20273744	0.893779252	0.844110657	Soltu.DM.07G002400/Soltu.DM.08G001690	2
583	GO:0070863	positive regulation of protein exit from endoplasmic reticulum	9/12756	0.20273744	0.893779252	0.844110657	Soltu.DM.07G002400/Soltu.DM.08G001690	2
584	GO:0070987	error-free translesion synthesis	9/12756	0.20273744	0.893779252	0.844110657	Soltu.DM.08G027160/Soltu.DM.06G015770	2
585	GO:0075136	response to host	9/12756	0.20273744	0.893779252	0.844110657	Soltu.DM.01G024680/Soltu.DM.07G014680	2
586	GO:1901068	guanosine-containing compound metabolic process	9/12756	0.20273744	0.893779252	0.844110657	Soltu.DM.03G037170/Soltu.DM.10G027910	2
587	GO:1903531	negative regulation of secretion by cell	9/12756	0.20273744	0.893779252	0.844110657	Soltu.DM.08G027150/Soltu.DM.09G002090	2
588	GO:2000068	regulation of defense response to insect	9/12756	0.20273744	0.893779252	0.844110657	Soltu.DM.02G025590/Soltu.DM.03G008510	2
589	GO:0030865	cortical cytoskeleton organization	70/12756	0.203968972	0.893779252	0.844110657	Soltu.DM.04G027320/Soltu.DM.12G007610/Soltu.DM.02G028740/Soltu.DM.01G028770/Soltu.DM.01G008180/Soltu.DM.04G022240/Soltu.DM.05G009320/Soltu.DM.11G018040/Soltu.DM.09G027230	9
590	GO:0048440	carpel development	70/12756	0.203968972	0.893779252	0.844110657	Soltu.DM.03G024660/Soltu.DM.03G024680/Soltu.DM.03G024670/Soltu.DM.10G023790/Soltu.DM.02G003130/Soltu.DM.05G003100/Soltu.DM.03G024690/Soltu.DM.01G000910/Soltu.DM.04G033440	9
591	GO:0048506	regulation of timing of meristematic phase transition	70/12756	0.203968972	0.893779252	0.844110657	Soltu.DM.04G035890/Soltu.DM.01G018690/Soltu.DM.07G020920/Soltu.DM.10G024780/Soltu.DM.04G033160/Soltu.DM.06G020280/Soltu.DM.08G013420/Soltu.DM.06G020260/Soltu.DM.10G022070	9
59	GO:00	regulation of timing of transition from	70/	0.2	0.8	0.8	Soltu.DM.04G035890/Soltu.DM.01G018690/Soltu.DM.07G020920/Soltu.DM.10G024780/S	9

2	48510	vegetative to reproductive phase	12756	03968972	93779252	44110657	oltu.DM.04G033160/Soltu.DM.06G020280/Soltu.DM.08G013420/Soltu.DM.06G020260/Soltu.DM.10G022070	
593	GO:0010647	positive regulation of cell communication	166/12756	0.207459504	0.893779252	0.844110657	Soltu.DM.09G018310/Soltu.DM.07G017210/Soltu.DM.07G017190/Soltu.DM.07G017200/Soltu.DM.04G033590/Soltu.DM.02G018520/Soltu.DM.07G017180/Soltu.DM.04G033180/Soltu.DM.09G028490/Soltu.DM.04G037130/Soltu.DM.01G047440/Soltu.DM.12G025260/Soltu.DM.09G022610/Soltu.DM.03G021360/Soltu.DM.08G011890/Soltu.DM.02G013430/Soltu.DM.09G026500/Soltu.DM.03G036780/Soltu.DM.02G026820	19
594	GO:0030307	positive regulation of cell growth	34/12756	0.2076309	0.893779252	0.844110657	Soltu.DM.12G020370/Soltu.DM.02G018520/Soltu.DM.12G020350/Soltu.DM.12G026380/Soltu.DM.12G020340	5
595	GO:0030837	negative regulation of actin filament polymerization	34/12756	0.2076309	0.893779252	0.844110657	Soltu.DM.06G022970/Soltu.DM.01G028770/Soltu.DM.09G015150/Soltu.DM.12G009990/Soltu.DM.10G004310	5
596	GO:0005978	glycogen biosynthetic process	17/12756	0.208016893	0.893779252	0.844110657	Soltu.DM.01G040570/Soltu.DM.02G024820/Soltu.DM.11G008240	3
597	GO:0006573	valine metabolic process	17/12756	0.208016893	0.893779252	0.844110657	Soltu.DM.03G005810/Soltu.DM.07G023080/Soltu.DM.11G003850	3
598	GO:0010721	negative regulation of cell development	17/12756	0.208016893	0.893779252	0.844110657	Soltu.DM.12G020370/Soltu.DM.12G020350/Soltu.DM.12G020340	3
599	GO:0016107	sesquiterpenoid catabolic process	17/12756	0.208016893	0.893779252	0.844110657	Soltu.DM.08G020150/Soltu.DM.07G013940/Soltu.DM.07G013900	3
600	GO:0031670	cellular response to nutrient	17/12756	0.208016893	0.893779252	0.844110657	Soltu.DM.01G008040/Soltu.DM.05G001260/Soltu.DM.06G026560	3
601	GO:0043290	apocarotenoid catabolic process	17/12756	0.208016893	0.893779252	0.844110657	Soltu.DM.08G020150/Soltu.DM.07G013940/Soltu.DM.07G013900	3
602	GO:0046113	nucleobase catabolic process	17/12756	0.208016893	0.893779252	0.844110657	Soltu.DM.02G017810/Soltu.DM.10G012990/Soltu.DM.03G019550	3
603	GO:0046889	positive regulation of lipid biosynthetic process	17/12756	0.208016893	0.893779252	0.844110657	Soltu.DM.02G020950/Soltu.DM.01G035240/Soltu.DM.10G005360	3
604	GO:0046940	nucleoside monophosphate phosphorylation	17/12756	0.208016893	0.893779252	0.844110657	Soltu.DM.05G011440/Soltu.DM.09G006670/Soltu.DM.05G023970	3
605	GO:0090481	pyrimidine nucleotide-sugar transmembrane transport	17/12756	0.208016893	0.893779252	0.844110657	Soltu.DM.11G022930/Soltu.DM.12G004060/Soltu.DM.03G032030	3
606	GO:0071395	cellular response to jasmonic acid stimulus	99/12756	0.212249064	0.910446068	0.859851274	Soltu.DM.02G025590/Soltu.DM.07G024710/Soltu.DM.07G012950/Soltu.DM.09G024260/Soltu.DM.09G029050/Soltu.DM.03G032770/Soltu.DM.12G029330/Soltu.DM.02G022460/Soltu.DM.01G000760/Soltu.DM.07G024240/Soltu.DM.01G017870/Soltu.DM.09G024270	12
607	GO:0042594	response to starvation	187/12756	0.21770709	0.929851836	0.878178635	Soltu.DM.02G017810/Soltu.DM.04G025250/Soltu.DM.01G049900/Soltu.DM.01G049890/Soltu.DM.07G028550/Soltu.DM.03G037170/Soltu.DM.08G030020/Soltu.DM.04G001370/Soltu.DM.10G000640/Soltu.DM.03G000340/Soltu.DM.07G020410/Soltu.DM.04G031580/Soltu.DM.07G017750/Soltu.DM.07G015200/Soltu.DM.11G007630/Soltu.DM.02G027940/Soltu.DM.01G028770/Soltu.DM.05G011970/Soltu.DM.11G022310/Soltu.DM.01G040220/Soltu.DM.10G002820	21
608	GO:0009965	leaf morphogenesis	158/12756	0.221071685	0.929851836	0.878178635	Soltu.DM.10G025140/Soltu.DM.09G002750/Soltu.DM.04G011110/Soltu.DM.09G019220/Soltu.DM.04G001110/Soltu.DM.05G006190/Soltu.DM.10G027470/Soltu.DM.12G023260/Soltu.DM.04G011320/Soltu.DM.05G006430/Soltu.DM.04G011240/Soltu.DM.10G028070/Soltu.DM.04G011370/Soltu.DM.03G024040/Soltu.DM.01G001590/Soltu.DM.08G012010/Soltu.DM.03G024000/Soltu.DM.11G021090	18

609	GO:0000741	karyogamy	26/12756	0.221101309	0.929851836	0.878178635	Soltu.DM.05G023990/Soltu.DM.02G012680/Soltu.DM.01G002850/Soltu.DM.12G002630	4
610	GO:0002791	regulation of peptide secretion	26/12756	0.221101309	0.929851836	0.878178635	Soltu.DM.08G027150/Soltu.DM.01G002690/Soltu.DM.09G002090/Soltu.DM.02G022620	4
611	GO:0009559	embryo sac central cell differentiation	26/12756	0.221101309	0.929851836	0.878178635	Soltu.DM.05G023990/Soltu.DM.02G012680/Soltu.DM.01G002850/Soltu.DM.12G002630	4
612	GO:0010197	polar nucleus fusion	26/12756	0.221101309	0.929851836	0.878178635	Soltu.DM.05G023990/Soltu.DM.02G012680/Soltu.DM.01G002850/Soltu.DM.12G002630	4
613	GO:0010305	leaf vascular tissue pattern formation	26/12756	0.221101309	0.929851836	0.878178635	Soltu.DM.09G020190/Soltu.DM.04G029270/Soltu.DM.04G021540/Soltu.DM.04G031030	4
614	GO:0042278	purine nucleoside metabolic process	26/12756	0.221101309	0.929851836	0.878178635	Soltu.DM.03G037170/Soltu.DM.10G027910/Soltu.DM.12G003790/Soltu.DM.12G002620	4
615	GO:0042335	cuticle development	26/12756	0.221101309	0.929851836	0.878178635	Soltu.DM.07G019030/Soltu.DM.06G020510/Soltu.DM.05G013430/Soltu.DM.07G020980	4
616	GO:0045489	pectin biosynthetic process	26/12756	0.221101309	0.929851836	0.878178635	Soltu.DM.02G031090/Soltu.DM.04G029850/Soltu.DM.06G021870/Soltu.DM.09G007590	4
617	GO:0050708	regulation of protein secretion	26/12756	0.221101309	0.929851836	0.878178635	Soltu.DM.08G027150/Soltu.DM.01G002690/Soltu.DM.09G002090/Soltu.DM.02G022620	4
618	GO:1905428	regulation of plant organ formation	26/12756	0.221101309	0.929851836	0.878178635	Soltu.DM.06G025210/Soltu.DM.02G023840/Soltu.DM.02G017390/Soltu.DM.03G034300	4
619	GO:1901568	fatty acid derivative metabolic process	53/12756	0.228360347	0.935621059	0.883627255	Soltu.DM.04G034690/Soltu.DM.10G005260/Soltu.DM.10G004300/Soltu.DM.06G034310/Soltu.DM.02G031030/Soltu.DM.02G019940/Soltu.DM.10G005430	7
620	GO:0032272	negative regulation of protein polymerization	35/12756	0.225056445	0.941875836	0.889534445	Soltu.DM.06G022970/Soltu.DM.01G028770/Soltu.DM.09G015150/Soltu.DM.12G009990/Soltu.DM.10G004310	5
621	GO:0072348	sulfur compound transport	35/12756	0.225056445	0.941875836	0.889534445	Soltu.DM.09G020160/Soltu.DM.07G020410/Soltu.DM.03G027330/Soltu.DM.05G021160/Soltu.DM.03G027340	5
622	GO:0023056	positive regulation of signaling	159/12756	0.229058617	0.9570715	0.903885664	Soltu.DM.09G018310/Soltu.DM.07G017210/Soltu.DM.07G017190/Soltu.DM.07G017200/Soltu.DM.04G033590/Soltu.DM.02G018520/Soltu.DM.07G017180/Soltu.DM.04G033180/Soltu.DM.09G028490/Soltu.DM.04G037130/Soltu.DM.12G025260/Soltu.DM.09G022610/Soltu.DM.03G021360/Soltu.DM.08G011890/Soltu.DM.02G013430/Soltu.DM.09G026500/Soltu.DM.03G036780/Soltu.DM.02G026820	18
623	GO:0051248	negative regulation of protein metabolic process	189/12756	0.232342181	0.959607738	0.90628096	Soltu.DM.09G031320/Soltu.DM.08G022190/Soltu.DM.01G033530/Soltu.DM.03G020450/Soltu.DM.05G004270/Soltu.DM.04G034360/Soltu.DM.07G012130/Soltu.DM.11G010230/Soltu.DM.11G010220/Soltu.DM.04G034390/Soltu.DM.04G034380/Soltu.DM.04G001110/Soltu.DM.08G013620/Soltu.DM.06G028580/Soltu.DM.04G003450/Soltu.DM.04G038280/Soltu.DM.05G006430/Soltu.DM.05G026810/Soltu.DM.09G031340/Soltu.DM.04G034280/Soltu.DM.04G031030	21
624	GO:0006551	leucine metabolic process	18/12756	0.233502819	0.959607738	0.90628096	Soltu.DM.06G018090/Soltu.DM.01G047450/Soltu.DM.11G003850	3
625	GO:0031053	primary miRNA processing	18/12756	0.233502819	0.959607738	0.90628096	Soltu.DM.12G005490/Soltu.DM.11G025410/Soltu.DM.04G031030	3

626	GO:0042548	regulation of photosynthesis, light reaction	18/12756	0.233502819	0.959607738	0.90628096	Soltu.DM.07G012130/Soltu.DM.07G000550/Soltu.DM.04G037460	3
627	GO:0044843	cell cycle G1/S phase transition	18/12756	0.233502819	0.959607738	0.90628096	Soltu.DM.03G003730/Soltu.DM.02G029740/Soltu.DM.01G047090	3
628	GO:0052325	cell wall pectin biosynthetic process	18/12756	0.233502819	0.959607738	0.90628096	Soltu.DM.02G031090/Soltu.DM.04G029850/Soltu.DM.06G021870	3
629	GO:0007031	peroxisome organization	54/12756	0.23705362	0.959607738	0.90628096	Soltu.DM.09G018310/Soltu.DM.01G033530/Soltu.DM.09G028490/Soltu.DM.12G001180/Soltu.DM.08G023320/Soltu.DM.10G004300/Soltu.DM.10G007000	7
630	GO:0043622	cortical microtubule organization	54/12756	0.23705362	0.959607738	0.90628096	Soltu.DM.04G027320/Soltu.DM.12G007610/Soltu.DM.02G028740/Soltu.DM.01G008180/Soltu.DM.04G022240/Soltu.DM.05G009320/Soltu.DM.11G018040	7
631	GO:0061136	regulation of proteasomal protein catabolic process	54/12756	0.23705362	0.959607738	0.90628096	Soltu.DM.08G027150/Soltu.DM.12G005510/Soltu.DM.06G015770/Soltu.DM.04G034740/Soltu.DM.09G005140/Soltu.DM.09G002090/Soltu.DM.06G024530	7
632	GO:1900055	regulation of leaf senescence	54/12756	0.23705362	0.959607738	0.90628096	Soltu.DM.07G017210/Soltu.DM.07G017190/Soltu.DM.07G017200/Soltu.DM.07G017180/Soltu.DM.04G033430/Soltu.DM.06G017300/Soltu.DM.07G014750	7
633	GO:1903050	regulation of proteolysis involved in protein catabolic process	54/12756	0.23705362	0.959607738	0.90628096	Soltu.DM.08G027150/Soltu.DM.12G005510/Soltu.DM.06G015770/Soltu.DM.04G034740/Soltu.DM.09G005140/Soltu.DM.09G002090/Soltu.DM.06G024530	7
634	GO:0006370	7-methylguanosine mRNA capping	10/12756	0.23859913	0.959607738	0.90628096	Soltu.DM.11G014610/Soltu.DM.11G014620	2
635	GO:0006415	translational termination	10/12756	0.23859913	0.959607738	0.90628096	Soltu.DM.07G001240/Soltu.DM.08G019530	2
636	GO:0006971	hypotonic response	10/12756	0.23859913	0.959607738	0.90628096	Soltu.DM.01G028770/Soltu.DM.01G040220	2
637	GO:0009452	7-methylguanosine RNA capping	10/12756	0.23859913	0.959607738	0.90628096	Soltu.DM.11G014610/Soltu.DM.11G014620	2
638	GO:0010358	leaf shaping	10/12756	0.23859913	0.959607738	0.90628096	Soltu.DM.10G025140/Soltu.DM.09G002750	2
639	GO:0015840	urea transport	10/12756	0.23859913	0.959607738	0.90628096	Soltu.DM.06G031120/Soltu.DM.03G031200	2
640	GO:0034311	diol metabolic process	10/12756	0.23859913	0.959607738	0.90628096	Soltu.DM.02G018520/Soltu.DM.08G014180	2
641	GO:0034312	diol biosynthetic process	10/12756	0.23859913	0.959607738	0.90628096	Soltu.DM.02G018520/Soltu.DM.08G014180	2
642	GO:0035384	thioester biosynthetic process	10/12756	0.23859913	0.959607738	0.90628096	Soltu.DM.02G031030/Soltu.DM.01G019520	2
643	GO:0042759	long-chain fatty acid biosynthetic process	10/1275	0.2385991	0.9596077	0.9062809	Soltu.DM.01G047750/Soltu.DM.10G004300	2

			6	31	38	6		
644	GO:0045176	apical protein localization	10/12756	0.238599131	0.959607738	0.90628096	Soltu.DM.06G005370/Soltu.DM.08G001470	2
645	GO:0071616	acyl-CoA biosynthetic process	10/12756	0.238599131	0.959607738	0.90628096	Soltu.DM.02G031030/Soltu.DM.01G019520	2
646	GO:1902047	polyamine transmembrane transport	10/12756	0.238599131	0.959607738	0.90628096	Soltu.DM.01G001470/Soltu.DM.08G003390	2
647	GO:0033865	nucleoside bisphosphate metabolic process	45/12756	0.240746835	0.962278379	0.908803189	Soltu.DM.01G033530/Soltu.DM.03G037170/Soltu.DM.08G026810/Soltu.DM.02G031030/Soltu.DM.11G010590/Soltu.DM.01G019520	6
648	GO:0033875	ribonucleoside bisphosphate metabolic process	45/12756	0.240746835	0.962278379	0.908803189	Soltu.DM.01G033530/Soltu.DM.03G037170/Soltu.DM.08G026810/Soltu.DM.02G031030/Soltu.DM.11G010590/Soltu.DM.01G019520	6
649	GO:0034032	purine nucleoside bisphosphate metabolic process	45/12756	0.240746835	0.962278379	0.908803189	Soltu.DM.01G033530/Soltu.DM.03G037170/Soltu.DM.08G026810/Soltu.DM.02G031030/Soltu.DM.11G010590/Soltu.DM.01G019520	6
650	GO:0007186	G protein-coupled receptor signaling pathway	27/12756	0.241876024	0.962278379	0.908803189	Soltu.DM.02G018520/Soltu.DM.10G022360/Soltu.DM.06G022970/Soltu.DM.08G011890	4
651	GO:0010584	pollen exine formation	27/12756	0.241876024	0.962278379	0.908803189	Soltu.DM.07G000410/Soltu.DM.03G020790/Soltu.DM.02G011180/Soltu.DM.02G031030	4
652	GO:0015740	C4-dicarboxylate transport	27/12756	0.241876024	0.962278379	0.908803189	Soltu.DM.04G031760/Soltu.DM.12G006380/Soltu.DM.11G000570/Soltu.DM.05G021160	4
653	GO:0034059	response to anoxia	27/12756	0.241876024	0.962278379	0.908803189	Soltu.DM.07G013360/Soltu.DM.10G019450/Soltu.DM.03G027730/Soltu.DM.01G024860	4
654	GO:0042127	regulation of cell population proliferation	102/12756	0.242709193	0.964105237	0.910528526	Soltu.DM.02G027330/Soltu.DM.12G026600/Soltu.DM.02G018520/Soltu.DM.01G024670/Soltu.DM.04G033430/Soltu.DM.08G013580/Soltu.DM.05G003100/Soltu.DM.08G011890/Soltu.DM.11G016820/Soltu.DM.09G004090/Soltu.DM.02G016680/Soltu.DM.10G024900	12
655	GO:0035967	cellular response to topologically incorrect protein	64/12756	0.245809666	0.974918953	0.920741308	Soltu.DM.07G011880/Soltu.DM.03G016820/Soltu.DM.08G029860/Soltu.DM.08G019590/Soltu.DM.06G012970/Soltu.DM.10G004220/Soltu.DM.05G005120/Soltu.DM.06G009790	8
656	GO:0010089	xylem development	55/12756	0.251556199	0.98857923	0.933642464	Soltu.DM.09G024260/Soltu.DM.02G019030/Soltu.DM.07G010930/Soltu.DM.06G019150/Soltu.DM.11G011390/Soltu.DM.01G007500/Soltu.DM.09G024270	7
657	GO:0019395	fatty acid oxidation	55/12756	0.251556199	0.98857923	0.933642464	Soltu.DM.08G024620/Soltu.DM.09G018310/Soltu.DM.01G038470/Soltu.DM.09G028490/Soltu.DM.06G018150/Soltu.DM.10G003920/Soltu.DM.08G026840	7
658	GO:0030587	sorocarp development	55/12756	0.251556199	0.98857923	0.933642464	Soltu.DM.07G017210/Soltu.DM.07G017190/Soltu.DM.07G017200/Soltu.DM.07G017180/Soltu.DM.02G028740/Soltu.DM.01G028770/Soltu.DM.10G004310	7
659	GO:0099120	socially cooperative development	55/12756	0.251556199	0.98857923	0.933642464	Soltu.DM.07G017210/Soltu.DM.07G017190/Soltu.DM.07G017200/Soltu.DM.07G017180/Soltu.DM.02G028740/Soltu.DM.01G028770/Soltu.DM.10G004310	7
660	GO:0010876	lipid localization	142/12756	0.251684738	0.98857923	0.933642464	Soltu.DM.09G028710/Soltu.DM.02G016290/Soltu.DM.08G016090/Soltu.DM.06G012350/Soltu.DM.06G005370/Soltu.DM.10G022360/Soltu.DM.06G022490/Soltu.DM.01G034820/Soltu.DM.02G016300/Soltu.DM.02G016770/Soltu.DM.02G016380/Soltu.DM.12G028510/Soltu.DM.03G024040/Soltu.DM.11G011430/Soltu.DM.02G016780/Soltu.DM.02G019940	16
661	GO:0034284	response to monosaccharide	74/12	0.2523	0.9885	0.9336	Soltu.DM.07G013360/Soltu.DM.08G008380/Soltu.DM.02G025970/Soltu.DM.07G020920/Soltu.DM.10G024780/Soltu.DM.08G013420/Soltu.DM.04G024100/Soltu.DM.08G011890/Sol	9

			756	21619	7923	42464	tu.DM.10G022070	
662	GO:0051223	regulation of protein transport	74/12756	0.252321619	0.98857923	0.933642464	Soltu.DM.12G020370/Soltu.DM.12G020350/Soltu.DM.08G027150/Soltu.DM.07G002400/Soltu.DM.01G002690/Soltu.DM.08G001690/Soltu.DM.09G002090/Soltu.DM.12G020340/Soltu.DM.02G022620	9
663	GO:0090087	regulation of peptide transport	74/12756	0.252321619	0.98857923	0.933642464	Soltu.DM.12G020370/Soltu.DM.12G020350/Soltu.DM.08G027150/Soltu.DM.07G002400/Soltu.DM.01G002690/Soltu.DM.08G001690/Soltu.DM.09G002090/Soltu.DM.12G020340/Soltu.DM.02G022620	9
664	GO:0071398	cellular response to fatty acid	103/12756	0.253213242	0.98906627	0.934102439	Soltu.DM.02G025590/Soltu.DM.07G024710/Soltu.DM.07G012950/Soltu.DM.09G024260/Soltu.DM.09G029050/Soltu.DM.03G032770/Soltu.DM.12G029330/Soltu.DM.02G022460/Soltu.DM.01G000760/Soltu.DM.07G024240/Soltu.DM.01G017870/Soltu.DM.09G024270	12
665	GO:0090066	regulation of anatomical structure size	103/12756	0.253213242	0.98906627	0.934102439	Soltu.DM.12G020370/Soltu.DM.12G020350/Soltu.DM.06G022970/Soltu.DM.01G028770/Soltu.DM.05G027000/Soltu.DM.09G015150/Soltu.DM.10G004300/Soltu.DM.12G009990/Soltu.DM.10G004310/Soltu.DM.12G020340/Soltu.DM.09G027230/Soltu.DM.10G007000	12
666	GO:0006635	fatty acid beta-oxidation	46/12756	0.256837662	0.99472165	0.939443542	Soltu.DM.08G024620/Soltu.DM.09G018310/Soltu.DM.01G038470/Soltu.DM.09G028490/Soltu.DM.06G018150/Soltu.DM.10G003920	6
667	GO:0043484	regulation of RNA splicing	46/12756	0.256837662	0.99472165	0.939443542	Soltu.DM.12G015690/Soltu.DM.12G005490/Soltu.DM.12G025260/Soltu.DM.11G016820/Soltu.DM.10G001400/Soltu.DM.11G019980	6
668	GO:0030258	lipid modification	133/12756	0.259494291	0.99472165	0.939443542	Soltu.DM.08G024620/Soltu.DM.09G018310/Soltu.DM.03G037120/Soltu.DM.02G018520/Soltu.DM.01G038470/Soltu.DM.09G028490/Soltu.DM.02G017970/Soltu.DM.05G011130/Soltu.DM.10G004300/Soltu.DM.06G018150/Soltu.DM.11G001520/Soltu.DM.04G034770/Soltu.DM.05G001470/Soltu.DM.10G003920/Soltu.DM.08G026840	15
669	GO:0010540	basipetal auxin transport	19/12756	0.259498192	0.99472165	0.939443542	Soltu.DM.01G035900/Soltu.DM.01G035910/Soltu.DM.08G001470	3
670	GO:0015908	fatty acid transport	19/12756	0.259498192	0.99472165	0.939443542	Soltu.DM.01G034820/Soltu.DM.03G024040/Soltu.DM.02G019940	3
671	GO:0016577	histone demethylation	19/12756	0.259498192	0.99472165	0.939443542	Soltu.DM.08G022190/Soltu.DM.04G006870/Soltu.DM.06G001230	3
672	GO:0031398	positive regulation of protein ubiquitination	19/12756	0.259498192	0.99472165	0.939443542	Soltu.DM.02G018520/Soltu.DM.06G015770/Soltu.DM.09G005140	3
673	GO:0070076	histone lysine demethylation	19/12756	0.259498192	0.99472165	0.939443542	Soltu.DM.08G022190/Soltu.DM.04G006870/Soltu.DM.06G001230	3
674	GO:0072488	ammonium transmembrane transport	19/12756	0.259498192	0.99472165	0.939443542	Soltu.DM.12G010360/Soltu.DM.06G018020/Soltu.DM.08G003390	3
675	GO:1903322	positive regulation of protein modification by small protein conjugation or removal	19/12756	0.259498192	0.99472165	0.939443542	Soltu.DM.02G018520/Soltu.DM.06G015770/Soltu.DM.09G005140	3
676	GO:0009962	regulation of flavonoid biosynthetic process	37/12756	0.261089955	0.99472165	0.939443542	Soltu.DM.03G031830/Soltu.DM.05G026160/Soltu.DM.01G024670/Soltu.DM.10G017480/Soltu.DM.03G021360	5
677	GO:0010214	seed coat development	37/12756	0.261089955	0.99472165	0.939443542	Soltu.DM.02G027540/Soltu.DM.03G017660/Soltu.DM.06G013150/Soltu.DM.10G023760/Soltu.DM.03G020440	5
678	GO:0031333	negative regulation of protein-containing complex assembly	37/12756	0.261089955	0.99472165	0.939443542	Soltu.DM.06G022970/Soltu.DM.01G028770/Soltu.DM.09G015150/Soltu.DM.12G009990/Soltu.DM.10G004310	5
67	GO:00	glycerolipid metabolic process	17	0.2	0.9	0.9	Soltu.DM.02G034460/Soltu.DM.02G016290/Soltu.DM.04G037130/Soltu.DM.02G024810/S	19

9	46486		3/12756	62583473	94721651	39443542	oltu.DM.03G030800/Soltu.DM.12G002120/Soltu.DM.02G010490/Soltu.DM.01G034820/Soltu.DM.04G034770/Soltu.DM.02G016300/Soltu.DM.05G001470/Soltu.DM.06G034310/Soltu.DM.02G016770/Soltu.DM.02G016380/Soltu.DM.07G026100/Soltu.DM.02G016780/Soltu.DM.06G018040/Soltu.DM.02G019940/Soltu.DM.10G005430	
680	GO:0009750	response to fructose	28/12756	0.263066306	0.994721651	0.939443542	Soltu.DM.07G020920/Soltu.DM.10G024780/Soltu.DM.08G013420/Soltu.DM.10G022070	4
681	GO:0016144	S-glycoside biosynthetic process	28/12756	0.263066306	0.994721651	0.939443542	Soltu.DM.05G007640/Soltu.DM.06G018090/Soltu.DM.02G020870/Soltu.DM.05G007630	4
682	GO:0019758	glycosinolate biosynthetic process	28/12756	0.263066306	0.994721651	0.939443542	Soltu.DM.05G007640/Soltu.DM.06G018090/Soltu.DM.02G020870/Soltu.DM.05G007630	4
683	GO:0019761	glucosinolate biosynthetic process	28/12756	0.263066306	0.994721651	0.939443542	Soltu.DM.05G007640/Soltu.DM.06G018090/Soltu.DM.02G020870/Soltu.DM.05G007630	4
684	GO:0030641	regulation of cellular pH	28/12756	0.263066306	0.994721651	0.939443542	Soltu.DM.02G024200/Soltu.DM.11G000570/Soltu.DM.07G009580/Soltu.DM.08G001690	4
685	GO:0051453	regulation of intracellular pH	28/12756	0.263066306	0.994721651	0.939443542	Soltu.DM.02G024200/Soltu.DM.11G000570/Soltu.DM.07G009580/Soltu.DM.08G001690	4
686	GO:0009260	ribonucleotide biosynthetic process	104/12756	0.263876494	0.994721651	0.939443542	Soltu.DM.12G004480/Soltu.DM.03G037170/Soltu.DM.07G009580/Soltu.DM.11G007630/Soltu.DM.10G027910/Soltu.DM.06G013720/Soltu.DM.03G021730/Soltu.DM.01G017170/Soltu.DM.02G031030/Soltu.DM.11G025570/Soltu.DM.11G010590/Soltu.DM.01G019520	12
687	GO:0046390	ribose phosphate biosynthetic process	104/12756	0.263876494	0.994721651	0.939443542	Soltu.DM.12G004480/Soltu.DM.03G037170/Soltu.DM.07G009580/Soltu.DM.11G007630/Soltu.DM.10G027910/Soltu.DM.06G013720/Soltu.DM.03G021730/Soltu.DM.01G017170/Soltu.DM.02G031030/Soltu.DM.11G025570/Soltu.DM.11G010590/Soltu.DM.01G019520	12
688	GO:0009062	fatty acid catabolic process	56/12756	0.266315833	0.994721651	0.939443542	Soltu.DM.08G024620/Soltu.DM.09G018310/Soltu.DM.01G033530/Soltu.DM.01G038470/Soltu.DM.09G028490/Soltu.DM.06G018150/Soltu.DM.10G003920	7
689	GO:0009736	cytokinin-activated signaling pathway	56/12756	0.266315833	0.994721651	0.939443542	Soltu.DM.03G027640/Soltu.DM.10G027680/Soltu.DM.05G024870/Soltu.DM.05G011970/Soltu.DM.06G011930/Soltu.DM.10G027810/Soltu.DM.06G011620	7
690	GO:0048825	cotyledon development	56/12756	0.266315833	0.994721651	0.939443542	Soltu.DM.05G006190/Soltu.DM.06G019760/Soltu.DM.11G024760/Soltu.DM.02G002480/Soltu.DM.03G024040/Soltu.DM.08G001470/Soltu.DM.02G016680	7
691	GO:1902600	proton transmembrane transport	95/12756	0.272339453	0.994721651	0.939443542	Soltu.DM.02G010790/Soltu.DM.09G024150/Soltu.DM.10G010160/Soltu.DM.02G024200/Soltu.DM.02G006700/Soltu.DM.07G009580/Soltu.DM.03G027330/Soltu.DM.02G018010/Soltu.DM.11G025570/Soltu.DM.05G021160/Soltu.DM.03G027340	11
692	GO:0010393	galacturonan metabolic process	47/12756	0.273206206	0.994721651	0.939443542	Soltu.DM.02G031090/Soltu.DM.07G000930/Soltu.DM.02G031050/Soltu.DM.04G029850/Soltu.DM.06G021870/Soltu.DM.09G007590	6
693	GO:0043069	negative regulation of programmed cell death	47/12756	0.273206206	0.994721651	0.939443542	Soltu.DM.02G018520/Soltu.DM.05G022160/Soltu.DM.08G022900/Soltu.DM.08G028440/Soltu.DM.04G022240/Soltu.DM.06G024530	6
694	GO:0046890	regulation of lipid biosynthetic process	47/12756	0.273206206	0.994721651	0.939443542	Soltu.DM.08G029860/Soltu.DM.02G020950/Soltu.DM.08G013580/Soltu.DM.01G035240/Soltu.DM.10G005360/Soltu.DM.07G024240	6
695	GO:0006743	ubiquinone metabolic process	11/12756	0.274725297	0.994721651	0.939443542	Soltu.DM.06G032850/Soltu.DM.06G032860	2
696	GO:0006744	ubiquinone biosynthetic process	11/1275	0.2747252	0.9947216	0.9394435	Soltu.DM.06G032850/Soltu.DM.06G032860	2

			6	97	51	42		
697	GO:0006882	cellular zinc ion homeostasis	11/12756	0.274725297	0.994721651	0.939443542	Soltu.DM.07G002440/Soltu.DM.07G009580	2
698	GO:0007004	telomere maintenance via telomerase	11/12756	0.274725297	0.994721651	0.939443542	Soltu.DM.05G023970/Soltu.DM.02G013390	2
699	GO:0009065	glutamine family amino acid catabolic process	11/12756	0.274725297	0.994721651	0.939443542	Soltu.DM.12G024030/Soltu.DM.08G007450	2
700	GO:0009901	anther dehiscence	11/12756	0.274725297	0.994721651	0.939443542	Soltu.DM.03G037120/Soltu.DM.04G022240	2
701	GO:0010184	cytokinin transport	11/12756	0.274725297	0.994721651	0.939443542	Soltu.DM.04G030440/Soltu.DM.06G012690	2
702	GO:0010508	positive regulation of autophagy	11/12756	0.274725297	0.994721651	0.939443542	Soltu.DM.08G014180/Soltu.DM.06G018840	2
703	GO:0010833	telomere maintenance via telomere lengthening	11/12756	0.274725297	0.994721651	0.939443542	Soltu.DM.05G023970/Soltu.DM.02G013390	2
704	GO:0015802	basic amino acid transport	11/12756	0.274725297	0.994721651	0.939443542	Soltu.DM.05G003990/Soltu.DM.06G017010	2
705	GO:0015846	polyamine transport	11/12756	0.274725297	0.994721651	0.939443542	Soltu.DM.01G001470/Soltu.DM.08G003390	2
706	GO:0015860	purine nucleoside transmembrane transport	11/12756	0.274725297	0.994721651	0.939443542	Soltu.DM.04G030440/Soltu.DM.06G012690	2
707	GO:0015939	pantothenate metabolic process	11/12756	0.274725297	0.994721651	0.939443542	Soltu.DM.04G025250/Soltu.DM.09G006800	2
708	GO:0030866	cortical actin cytoskeleton organization	11/12756	0.274725297	0.994721651	0.939443542	Soltu.DM.01G028770/Soltu.DM.09G027230	2
709	GO:0044804	autophagy of nucleus	11/12756	0.274725297	0.994721651	0.939443542	Soltu.DM.01G039130/Soltu.DM.11G022310	2
710	GO:0046513	ceramide biosynthetic process	11/12756	0.274725297	0.994721651	0.939443542	Soltu.DM.08G014180/Soltu.DM.01G047750	2
711	GO:0051238	sequestering of metal ion	11/12756	0.274725297	0.994721651	0.939443542	Soltu.DM.04G003430/Soltu.DM.07G009580	2
712	GO:0070370	cellular heat acclimation	11/12756	0.274725297	0.994721651	0.939443542	Soltu.DM.06G018840/Soltu.DM.08G012010	2
713	GO:0071668	plant-type cell wall assembly	11/12756	0.274725297	0.994721651	0.939443542	Soltu.DM.02G009140/Soltu.DM.04G022240	2
714	GO:1903826	L-arginine transmembrane transport	11/12	0.2747	0.9947	0.9394	Soltu.DM.05G003990/Soltu.DM.06G017010	2

			756	25297	21651	43542		
715	GO:1990822	basic amino acid transmembrane transport	11/12756	0.274725297	0.994721651	0.939443542	Soltu.DM.05G003990/Soltu.DM.06G017010	2
716	GO:2000008	regulation of protein localization to cell surface	11/12756	0.274725297	0.994721651	0.939443542	Soltu.DM.07G002400/Soltu.DM.08G001690	2
717	GO:2000279	negative regulation of DNA biosynthetic process	11/12756	0.274725297	0.994721651	0.939443542	Soltu.DM.05G006310/Soltu.DM.02G013390	2
718	GO:0070201	regulation of establishment of protein localization	76/12756	0.277805328	0.999994594	0.944423446	Soltu.DM.12G020370/Soltu.DM.12G020350/Soltu.DM.08G027150/Soltu.DM.07G002400/Soltu.DM.01G002690/Soltu.DM.08G001690/Soltu.DM.09G002090/Soltu.DM.12G020340/Soltu.DM.02G022620	9
719	GO:0009631	cold acclimation	38/12756	0.279586839	0.999994594	0.944423446	Soltu.DM.02G024660/Soltu.DM.04G037380/Soltu.DM.02G024670/Soltu.DM.08G024250/Soltu.DM.03G016750	5
720	GO:0009152	purine ribonucleotide biosynthetic process	86/12756	0.281297295	0.999994594	0.944423446	Soltu.DM.12G004480/Soltu.DM.03G037170/Soltu.DM.07G009580/Soltu.DM.10G027910/Soltu.DM.06G013720/Soltu.DM.03G021730/Soltu.DM.02G031030/Soltu.DM.11G025570/Soltu.DM.11G010590/Soltu.DM.01G019520	10
721	GO:0040034	regulation of development, heterochronic	86/12756	0.281297295	0.999994594	0.944423446	Soltu.DM.04G035890/Soltu.DM.01G018690/Soltu.DM.07G020920/Soltu.DM.10G024780/Soltu.DM.04G033160/Soltu.DM.06G020280/Soltu.DM.08G013420/Soltu.DM.06G020260/Soltu.DM.10G022070/Soltu.DM.01G010020	10
722	GO:0097164	ammonium ion metabolic process	57/12756	0.281304183	0.999994594	0.944423446	Soltu.DM.02G018520/Soltu.DM.08G014180/Soltu.DM.06G014480/Soltu.DM.03G030800/Soltu.DM.01G050280/Soltu.DM.06G018040/Soltu.DM.02G019940	7
723	GO:0044272	sulfur compound biosynthetic process	106/12756	0.285639944	0.999994594	0.944423446	Soltu.DM.05G007640/Soltu.DM.08G030020/Soltu.DM.12G022190/Soltu.DM.06G018090/Soltu.DM.06G018840/Soltu.DM.02G020870/Soltu.DM.05G007630/Soltu.DM.12G025770/Soltu.DM.06G003240/Soltu.DM.02G031030/Soltu.DM.07G014750/Soltu.DM.01G019520	12
724	GO:0003018	vascular process in circulatory system	20/12756	0.285839685	0.999994594	0.944423446	Soltu.DM.03G027330/Soltu.DM.10G004300/Soltu.DM.03G027340	3
725	GO:0006301	postreplication repair	20/12756	0.285839685	0.999994594	0.944423446	Soltu.DM.08G027160/Soltu.DM.06G015770/Soltu.DM.02G033290	3
726	GO:0006482	protein demethylation	20/12756	0.285839685	0.999994594	0.944423446	Soltu.DM.08G022190/Soltu.DM.04G006870/Soltu.DM.06G001230	3
727	GO:0008214	protein dealkylation	20/12756	0.285839685	0.999994594	0.944423446	Soltu.DM.08G022190/Soltu.DM.04G006870/Soltu.DM.06G001230	3
728	GO:0009608	response to symbiont	20/12756	0.285839685	0.999994594	0.944423446	Soltu.DM.03G017570/Soltu.DM.09G003770/Soltu.DM.01G002850	3
729	GO:0009900	dehiscence	20/12756	0.285839685	0.999994594	0.944423446	Soltu.DM.03G037120/Soltu.DM.04G022240/Soltu.DM.08G005070	3
730	GO:0015995	chlorophyll biosynthetic process	20/12756	0.285839685	0.999994594	0.944423446	Soltu.DM.08G028310/Soltu.DM.04G031570/Soltu.DM.06G002140	3
731	GO:0030048	actin filament-based movement	20/12756	0.285839685	0.999994594	0.944423446	Soltu.DM.09G018720/Soltu.DM.01G028770/Soltu.DM.10G001460	3
73	GO:00	positive regulation of lipid metabolic	20/	0.2	0.9	0.9	Soltu.DM.02G020950/Soltu.DM.01G035240/Soltu.DM.10G005360	3

2	45834	process	12756	85839685	99994594	4442346		
733	GO:0045931	positive regulation of mitotic cell cycle	20/12756	0.285839685	0.999994594	0.94442346	Soltu.DM.02G018520/Soltu.DM.03G003730/Soltu.DM.12G023230	3
734	GO:0072583	clathrin-dependent endocytosis	20/12756	0.285839685	0.999994594	0.94442346	Soltu.DM.09G000440/Soltu.DM.10G023680/Soltu.DM.01G042120	3
735	GO:1901334	lactone metabolic process	20/12756	0.285839685	0.999994594	0.94442346	Soltu.DM.02G025970/Soltu.DM.06G009750/Soltu.DM.01G045760	3
736	GO:1901336	lactone biosynthetic process	20/12756	0.285839685	0.999994594	0.94442346	Soltu.DM.02G025970/Soltu.DM.06G009750/Soltu.DM.01G045760	3
737	GO:0071322	cellular response to carbohydrate stimulus	87/12756	0.293561251	0.999994594	0.94442346	Soltu.DM.06G026960/Soltu.DM.01G006210/Soltu.DM.08G008380/Soltu.DM.07G020920/Soltu.DM.10G024780/Soltu.DM.08G013420/Soltu.DM.05G011970/Soltu.DM.02G002480/Soltu.DM.10G022070/Soltu.DM.07G022640	10
738	GO:0051494	negative regulation of cytoskeleton organization	39/12756	0.298330369	0.999994594	0.94442346	Soltu.DM.06G022970/Soltu.DM.01G028770/Soltu.DM.09G015150/Soltu.DM.12G009990/Soltu.DM.10G004310	5
739	GO:1902904	negative regulation of supramolecular fiber organization	39/12756	0.298330369	0.999994594	0.94442346	Soltu.DM.06G022970/Soltu.DM.01G028770/Soltu.DM.09G015150/Soltu.DM.12G009990/Soltu.DM.10G004310	5
740	GO:0048653	anther development	68/12756	0.300966333	0.999994594	0.94442346	Soltu.DM.03G024660/Soltu.DM.03G024680/Soltu.DM.03G037120/Soltu.DM.03G024670/Soltu.DM.03G024690/Soltu.DM.10G000600/Soltu.DM.04G022240/Soltu.DM.03G000400	8
741	GO:0035966	response to topologically incorrect protein	78/12756	0.303975551	0.999994594	0.94442346	Soltu.DM.07G011880/Soltu.DM.03G016820/Soltu.DM.04G037150/Soltu.DM.08G029860/Soltu.DM.08G019590/Soltu.DM.06G012970/Soltu.DM.10G004220/Soltu.DM.05G005120/Soltu.DM.06G009790	9
742	GO:0006863	purine nucleobase transport	30/12756	0.306344648	0.999994594	0.94442346	Soltu.DM.04G030440/Soltu.DM.07G000710/Soltu.DM.06G012690/Soltu.DM.02G014120	4
743	GO:0051651	maintenance of location in cell	30/12756	0.306344648	0.999994594	0.94442346	Soltu.DM.04G003430/Soltu.DM.07G009580/Soltu.DM.06G022970/Soltu.DM.12G009990	4
744	GO:1904823	purine nucleobase transmembrane transport	30/12756	0.306344648	0.999994594	0.94442346	Soltu.DM.04G030440/Soltu.DM.07G000710/Soltu.DM.06G012690/Soltu.DM.02G014120	4
745	GO:0045862	positive regulation of proteolysis	49/12756	0.306622814	0.999994594	0.94442346	Soltu.DM.12G005510/Soltu.DM.06G015770/Soltu.DM.04G034740/Soltu.DM.09G005140/Soltu.DM.06G018040/Soltu.DM.06G024530	6
746	GO:0032535	regulation of cellular component size	98/12756	0.307214562	0.999994594	0.94442346	Soltu.DM.12G020370/Soltu.DM.12G020350/Soltu.DM.06G022970/Soltu.DM.01G028770/Soltu.DM.05G027000/Soltu.DM.09G015150/Soltu.DM.12G009990/Soltu.DM.10G004310/Soltu.DM.12G020340/Soltu.DM.09G027230/Soltu.DM.10G007000	11
747	GO:0010075	regulation of meristem growth	108/12756	0.307916087	0.999994594	0.94442346	Soltu.DM.04G011110/Soltu.DM.10G027470/Soltu.DM.05G024870/Soltu.DM.04G011320/Soltu.DM.04G011240/Soltu.DM.10G028070/Soltu.DM.05G012760/Soltu.DM.04G011370/Soltu.DM.12G026560/Soltu.DM.02G016680/Soltu.DM.11G021090/Soltu.DM.10G024000	12
748	GO:0000002	mitochondrial genome maintenance	12/12756	0.310753671	0.999994594	0.94442346	Soltu.DM.04G034330/Soltu.DM.08G023320	2
749	GO:0000422	autophagy of mitochondrion	12/12756	0.310753671	0.999994594	0.94442346	Soltu.DM.01G039130/Soltu.DM.11G022310	2

750	GO:0000447	endonucleolytic cleavage in ITS1 to separate SSU-rRNA from 5.8S rRNA and LSU-rRNA from tricistronic rRNA transcript (SSU-rRNA, 5.8S rRNA, LSU-rRNA)	12/12756	0.310753671	0.999994594	0.94442346	Soltu.DM.12G024350/Soltu.DM.01G051600	2
751	GO:0006278	RNA-templated DNA biosynthetic process	12/12756	0.310753671	0.999994594	0.94442346	Soltu.DM.05G023970/Soltu.DM.02G013390	2
752	GO:0006561	proline biosynthetic process	12/12756	0.310753671	0.999994594	0.94442346	Soltu.DM.08G007450/Soltu.DM.07G014750	2
753	GO:0009610	response to symbiotic fungus	12/12756	0.310753671	0.999994594	0.94442346	Soltu.DM.09G003770/Soltu.DM.01G002850	2
754	GO:0009821	alkaloid biosynthetic process	12/12756	0.310753671	0.999994594	0.94442346	Soltu.DM.02G016870/Soltu.DM.08G001740	2
755	GO:0015786	UDP-glucose transmembrane transport	12/12756	0.310753671	0.999994594	0.94442346	Soltu.DM.12G004060/Soltu.DM.03G032030	2
756	GO:0018106	peptidyl-histidine phosphorylation	12/12756	0.310753671	0.999994594	0.94442346	Soltu.DM.05G011970/Soltu.DM.07G022640	2
757	GO:0043144	sno(s)RNA processing	12/12756	0.310753671	0.999994594	0.94442346	Soltu.DM.02G021990/Soltu.DM.06G029830	2
758	GO:0045815	transcription initiation-coupled chromatin remodeling	12/12756	0.310753671	0.999994594	0.94442346	Soltu.DM.08G022190/Soltu.DM.10G024770	2
759	GO:0061726	mitochondrion disassembly	12/12756	0.310753671	0.999994594	0.94442346	Soltu.DM.01G039130/Soltu.DM.11G022310	2
760	GO:0070726	cell wall assembly	12/12756	0.310753671	0.999994594	0.94442346	Soltu.DM.02G009140/Soltu.DM.04G022240	2
761	GO:0071454	cellular response to anoxia	12/12756	0.310753671	0.999994594	0.94442346	Soltu.DM.03G027730/Soltu.DM.01G024860	2
762	GO:0010072	primary shoot apical meristem specification	21/12756	0.312375594	0.999994594	0.94442346	Soltu.DM.02G003130/Soltu.DM.05G026810/Soltu.DM.06G034230	3
763	GO:0032886	regulation of microtubule-based process	21/12756	0.312375594	0.999994594	0.94442346	Soltu.DM.03G003730/Soltu.DM.04G022240/Soltu.DM.08G010920	3
764	GO:1901617	organic hydroxy compound biosynthetic process	179/12756	0.313739873	0.999994594	0.94442346	Soltu.DM.02G018520/Soltu.DM.08G020150/Soltu.DM.11G024450/Soltu.DM.01G024670/Soltu.DM.08G014180/Soltu.DM.03G021440/Soltu.DM.05G015440/Soltu.DM.06G004460/Soltu.DM.06G029640/Soltu.DM.02G030630/Soltu.DM.03G008510/Soltu.DM.02G007460/Soltu.DM.10G003570/Soltu.DM.06G003240/Soltu.DM.08G026700/Soltu.DM.10G022710/Soltu.DM.08G027080/Soltu.DM.09G023400/Soltu.DM.10G003550	19
765	GO:0019216	regulation of lipid metabolic process	69/12756	0.31518865	0.999994594	0.94442346	Soltu.DM.08G029860/Soltu.DM.02G020950/Soltu.DM.08G013580/Soltu.DM.10G022360/Soltu.DM.10G004300/Soltu.DM.01G035240/Soltu.DM.10G005360/Soltu.DM.07G024240	8
766	GO:0000966	RNA 5'-end processing	40/12756	0.317266558	0.999994594	0.94442346	Soltu.DM.12G016740/Soltu.DM.01G051600/Soltu.DM.11G004920/Soltu.DM.06G002320/Soltu.DM.06G029830	5
76	GO:00	adaxial/abaxial pattern specification	40/	0.3	0.9	0.9	Soltu.DM.02G003130/Soltu.DM.04G006620/Soltu.DM.11G007750/Soltu.DM.07G026690/S	5

7	09955		12756	17266558	99994594	4442346	oltu.DM.08G012010	
768	GO:0030148	sphingolipid biosynthetic process	40/12756	0.317266558	0.999994594	0.94442346	Soltu.DM.04G008710/Soltu.DM.02G018520/Soltu.DM.08G014180/Soltu.DM.01G047750/Soltu.DM.10G020020	5
769	GO:0043288	apocarotenoid metabolic process	40/12756	0.317266558	0.999994594	0.94442346	Soltu.DM.08G020150/Soltu.DM.11G024450/Soltu.DM.07G013940/Soltu.DM.06G029640/Soltu.DM.07G013900	5
770	GO:1901800	positive regulation of proteasomal protein catabolic process	40/12756	0.317266558	0.999994594	0.94442346	Soltu.DM.12G005510/Soltu.DM.06G015770/Soltu.DM.04G034740/Soltu.DM.09G005140/Soltu.DM.06G024530	5
771	GO:1902644	tertiary alcohol metabolic process	40/12756	0.317266558	0.999994594	0.94442346	Soltu.DM.08G020150/Soltu.DM.11G024450/Soltu.DM.07G013940/Soltu.DM.06G029640/Soltu.DM.07G013900	5
772	GO:1903052	positive regulation of proteolysis involved in protein catabolic process	40/12756	0.317266558	0.999994594	0.94442346	Soltu.DM.12G005510/Soltu.DM.06G015770/Soltu.DM.04G034740/Soltu.DM.09G005140/Soltu.DM.06G024530	5
773	GO:2000024	regulation of leaf development	79/12756	0.317273248	0.999994594	0.94442346	Soltu.DM.07G017210/Soltu.DM.07G017190/Soltu.DM.07G017200/Soltu.DM.12G026600/Soltu.DM.07G017180/Soltu.DM.04G033430/Soltu.DM.03G018850/Soltu.DM.06G017300/Soltu.DM.07G014750	9
774	GO:0015698	inorganic anion transport	99/12756	0.319085249	0.999994594	0.94442346	Soltu.DM.02G010790/Soltu.DM.08G025260/Soltu.DM.09G020160/Soltu.DM.10G010160/Soltu.DM.03G035250/Soltu.DM.07G020410/Soltu.DM.03G027330/Soltu.DM.06G017770/Soltu.DM.03G031200/Soltu.DM.05G021160/Soltu.DM.03G035260	11
775	GO:0009081	branched-chain amino acid metabolic process	50/12756	0.323594571	0.999994594	0.94442346	Soltu.DM.04G025250/Soltu.DM.03G005810/Soltu.DM.06G018090/Soltu.DM.07G023080/Soltu.DM.01G047450/Soltu.DM.11G003850	6
776	GO:0048236	plant-type sporogenesis	50/12756	0.323594571	0.999994594	0.94442346	Soltu.DM.06G009270/Soltu.DM.09G031520/Soltu.DM.05G022790/Soltu.DM.02G011180/Soltu.DM.09G031510/Soltu.DM.12G023840	6
777	GO:0006555	methionine metabolic process	31/12756	0.328265796	0.999994594	0.94442346	Soltu.DM.03G035070/Soltu.DM.08G030020/Soltu.DM.03G035080/Soltu.DM.12G002620	4
778	GO:0006779	porphyrin-containing compound biosynthetic process	31/12756	0.328265796	0.999994594	0.94442346	Soltu.DM.08G013640/Soltu.DM.08G028310/Soltu.DM.04G031570/Soltu.DM.06G002140	4
779	GO:0009682	induced systemic resistance	31/12756	0.328265796	0.999994594	0.94442346	Soltu.DM.02G025590/Soltu.DM.07G028550/Soltu.DM.10G000640/Soltu.DM.02G022460	4
780	GO:0015851	nucleobase transport	31/12756	0.328265796	0.999994594	0.94442346	Soltu.DM.04G030440/Soltu.DM.07G000710/Soltu.DM.06G012690/Soltu.DM.02G014120	4
781	GO:0046460	neutral lipid biosynthetic process	31/12756	0.328265796	0.999994594	0.94442346	Soltu.DM.02G024810/Soltu.DM.12G002120/Soltu.DM.01G034820/Soltu.DM.10G005430	4
782	GO:0046463	acylglycerol biosynthetic process	31/12756	0.328265796	0.999994594	0.94442346	Soltu.DM.02G024810/Soltu.DM.12G002120/Soltu.DM.01G034820/Soltu.DM.10G005430	4
783	GO:0010053	root epidermal cell differentiation	130/12756	0.3289786	0.999994594	0.94442346	Soltu.DM.08G027650/Soltu.DM.08G029290/Soltu.DM.04G001370/Soltu.DM.12G008450/Soltu.DM.12G004060/Soltu.DM.12G005490/Soltu.DM.08G023170/Soltu.DM.12G024710/Soltu.DM.09G026810/Soltu.DM.09G031790/Soltu.DM.03G001740/Soltu.DM.01G042120/Soltu.DM.03G018740/Soltu.DM.04G002690	14
784	GO:0006694	steroid biosynthetic process	70/12756	0.329539576	0.999994594	0.94442346	Soltu.DM.08G020150/Soltu.DM.05G015440/Soltu.DM.06G004460/Soltu.DM.02G030630/Soltu.DM.02G007460/Soltu.DM.10G003570/Soltu.DM.08G027080/Soltu.DM.10G003550	8

785	GO:0051703	biological process involved in intraspecies interaction between organisms	70/12756	0.329539576	0.999994594	0.94442346	Soltu.DM.07G017210/Soltu.DM.07G017190/Soltu.DM.07G017200/Soltu.DM.07G017180/Soltu.DM.02G028740/Soltu.DM.12G024030/Soltu.DM.01G028770/Soltu.DM.10G004310	8
786	GO:0010109	regulation of photosynthesis	41/12756	0.336342729	0.999994594	0.94442346	Soltu.DM.07G012130/Soltu.DM.06G028040/Soltu.DM.01G024860/Soltu.DM.07G000550/Soltu.DM.04G037460	5
787	GO:0010162	seed dormancy process	22/12756	0.338966447	0.999994594	0.94442346	Soltu.DM.01G001590/Soltu.DM.03G013100/Soltu.DM.07G024500	3
788	GO:0022611	dormancy process	22/12756	0.338966447	0.999994594	0.94442346	Soltu.DM.01G001590/Soltu.DM.03G013100/Soltu.DM.07G024500	3
789	GO:0006576	biogenic amine metabolic process	61/12756	0.342973763	0.999994594	0.94442346	Soltu.DM.07G014310/Soltu.DM.03G035070/Soltu.DM.06G014480/Soltu.DM.02G020890/Soltu.DM.01G050280/Soltu.DM.03G035080/Soltu.DM.01G027080	7
790	GO:0044275	cellular carbohydrate catabolic process	61/12756	0.342973763	0.999994594	0.94442346	Soltu.DM.09G027770/Soltu.DM.05G006330/Soltu.DM.01G018690/Soltu.DM.01G040550/Soltu.DM.11G000740/Soltu.DM.04G037250/Soltu.DM.06G020260	7
791	GO:1901420	negative regulation of response to alcohol	61/12756	0.342973763	0.999994594	0.94442346	Soltu.DM.07G012130/Soltu.DM.01G046820/Soltu.DM.06G017300/Soltu.DM.01G000060/Soltu.DM.04G024100/Soltu.DM.08G011890/Soltu.DM.04G005970	7
792	GO:1905958	negative regulation of cellular response to alcohol	61/12756	0.342973763	0.999994594	0.94442346	Soltu.DM.07G012130/Soltu.DM.01G046820/Soltu.DM.06G017300/Soltu.DM.01G000060/Soltu.DM.04G024100/Soltu.DM.08G011890/Soltu.DM.04G005970	7
793	GO:0006470	protein dephosphorylation	71/12756	0.343996839	0.999994594	0.94442346	Soltu.DM.01G039580/Soltu.DM.04G020850/Soltu.DM.07G012130/Soltu.DM.11G010230/Soltu.DM.01G039760/Soltu.DM.11G010220/Soltu.DM.06G003060/Soltu.DM.02G023840	8
794	GO:0009746	response to hexose	71/12756	0.343996839	0.999994594	0.94442346	Soltu.DM.07G013360/Soltu.DM.08G008380/Soltu.DM.07G020920/Soltu.DM.10G024780/Soltu.DM.08G013420/Soltu.DM.04G024100/Soltu.DM.08G011890/Soltu.DM.10G022070	8
795	GO:0090502	RNA phosphodiester bond hydrolysis, endonucleolytic	81/12756	0.344203298	0.999994594	0.94442346	Soltu.DM.02G018270/Soltu.DM.09G014740/Soltu.DM.12G024350/Soltu.DM.01G051600/Soltu.DM.11G004920/Soltu.DM.07G006530/Soltu.DM.04G031030/Soltu.DM.06G029830/Soltu.DM.11G004150	9
796	GO:0006672	ceramide metabolic process	13/12756	0.346387281	0.999994594	0.94442346	Soltu.DM.08G014180/Soltu.DM.01G047750	2
797	GO:0007187	G protein-coupled receptor signaling pathway, coupled to cyclic nucleotide second messenger	13/12756	0.346387281	0.999994594	0.94442346	Soltu.DM.10G022360/Soltu.DM.08G011890	2
798	GO:0007188	adenylate cyclase-modulating G protein-coupled receptor signaling pathway	13/12756	0.346387281	0.999994594	0.94442346	Soltu.DM.10G022360/Soltu.DM.08G011890	2
799	GO:0008217	regulation of blood pressure	13/12756	0.346387281	0.999994594	0.94442346	Soltu.DM.02G017970/Soltu.DM.10G004300	2
800	GO:0009820	alkaloid metabolic process	13/12756	0.346387281	0.999994594	0.94442346	Soltu.DM.02G016870/Soltu.DM.08G001740	2
801	GO:0009963	positive regulation of flavonoid biosynthetic process	13/12756	0.346387281	0.999994594	0.94442346	Soltu.DM.03G031830/Soltu.DM.01G024670	2
802	GO:0010018	far-red light signaling pathway	13/1275	0.3463872	0.9999945	0.9444234	Soltu.DM.07G028470/Soltu.DM.02G011380	2

			6	81	94	6		
803	GO:0010100	negative regulation of photomorphogenesis	13/12756	0.346387281	0.999994594	0.94442346	Soltu.DM.02G002480/Soltu.DM.06G002140	2
804	GO:0018874	benzoate metabolic process	13/12756	0.346387281	0.999994594	0.94442346	Soltu.DM.05G007640/Soltu.DM.05G007630	2
805	GO:0019388	galactose catabolic process	13/12756	0.346387281	0.999994594	0.94442346	Soltu.DM.02G031920/Soltu.DM.02G031890	2
806	GO:0033275	actin-myosin filament sliding	13/12756	0.346387281	0.999994594	0.94442346	Soltu.DM.09G018720/Soltu.DM.01G028770	2
807	GO:0048574	long-day photoperiodism, flowering	13/12756	0.346387281	0.999994594	0.94442346	Soltu.DM.01G041980/Soltu.DM.01G024340	2
808	GO:1902358	sulfate transmembrane transport	13/12756	0.346387281	0.999994594	0.94442346	Soltu.DM.09G020160/Soltu.DM.05G021160	2
809	GO:0003013	circulatory system process	32/12756	0.350269818	0.999994594	0.94442346	Soltu.DM.03G027330/Soltu.DM.02G017970/Soltu.DM.10G004300/Soltu.DM.03G027340	4
810	GO:0009593	detection of chemical stimulus	32/12756	0.350269818	0.999994594	0.94442346	Soltu.DM.10G026220/Soltu.DM.03G037290/Soltu.DM.12G021010/Soltu.DM.07G022640	4
811	GO:0009861	jasmonic acid and ethylene-dependent systemic resistance	32/12756	0.350269818	0.999994594	0.94442346	Soltu.DM.02G014980/Soltu.DM.02G015010/Soltu.DM.02G014950/Soltu.DM.03G008510	4
812	GO:0045596	negative regulation of cell differentiation	32/12756	0.350269818	0.999994594	0.94442346	Soltu.DM.12G020370/Soltu.DM.12G020350/Soltu.DM.07G020410/Soltu.DM.12G020340	4
813	GO:0070988	demethylation	32/12756	0.350269818	0.999994594	0.94442346	Soltu.DM.08G022190/Soltu.DM.04G006870/Soltu.DM.10G024770/Soltu.DM.06G001230	4
814	GO:0042542	response to hydrogen peroxide	122/12756	0.351931551	0.999994594	0.94442346	Soltu.DM.02G025590/Soltu.DM.06G026960/Soltu.DM.07G011880/Soltu.DM.12G007510/Soltu.DM.01G040550/Soltu.DM.03G034540/Soltu.DM.02G022700/Soltu.DM.04G027760/Soltu.DM.08G024250/Soltu.DM.08G006060/Soltu.DM.04G007430/Soltu.DM.01G028770/Soltu.DM.06G020260	13
815	GO:0010563	negative regulation of phosphorus metabolic process	42/12756	0.355507822	0.999994594	0.94442346	Soltu.DM.07G012130/Soltu.DM.11G010230/Soltu.DM.11G010220/Soltu.DM.06G028580/Soltu.DM.04G038280	5
816	GO:0045936	negative regulation of phosphate metabolic process	42/12756	0.355507822	0.999994594	0.94442346	Soltu.DM.07G012130/Soltu.DM.11G010230/Soltu.DM.11G010220/Soltu.DM.06G028580/Soltu.DM.04G038280	5
817	GO:0071218	cellular response to misfolded protein	42/12756	0.355507822	0.999994594	0.94442346	Soltu.DM.03G016820/Soltu.DM.06G012970/Soltu.DM.10G004220/Soltu.DM.05G005120/Soltu.DM.06G009790	5
818	GO:0030833	regulation of actin filament polymerization	52/12756	0.357878106	0.999994594	0.94442346	Soltu.DM.06G022970/Soltu.DM.01G028770/Soltu.DM.09G015150/Soltu.DM.12G009990/Soltu.DM.10G004310/Soltu.DM.09G027230	6
819	GO:0051338	regulation of transferase activity	143/12756	0.358476985	0.999994594	0.94442346	Soltu.DM.06G026960/Soltu.DM.07G017210/Soltu.DM.07G017190/Soltu.DM.07G017200/Soltu.DM.07G017180/Soltu.DM.07G012130/Soltu.DM.06G018320/Soltu.DM.11G009630/Soltu.DM.11G010230/Soltu.DM.02G028740/Soltu.DM.08G027160/Soltu.DM.11G010220/Soltu.DM.06G028580/Soltu.DM.04G038280/Soltu.DM.02G013390	15
820	GO:0034219	carbohydrate transmembrane transport	72/12	0.3585	0.9999	0.9444	Soltu.DM.02G014590/Soltu.DM.09G024150/Soltu.DM.01G008290/Soltu.DM.02G006700/Soltu.DM.03G020090/Soltu.DM.02G018010/Soltu.DM.03G031200/Soltu.DM.01G039610	8

			756	38351	94594	2346		
821	GO:0010088	phloem development	23/12756	0.365485259	0.999994594	0.94442346	Soltu.DM.05G010240/Soltu.DM.06G019150/Soltu.DM.05G010260	3
822	GO:0034620	cellular response to unfolded protein	23/12756	0.365485259	0.999994594	0.94442346	Soltu.DM.07G011880/Soltu.DM.08G029860/Soltu.DM.08G019590	3
823	GO:0080022	primary root development	23/12756	0.365485259	0.999994594	0.94442346	Soltu.DM.05G006190/Soltu.DM.10G000600/Soltu.DM.06G020260	3
824	GO:0048573	photoperiodism, flowering	83/12756	0.37145933	0.999994594	0.94442346	Soltu.DM.02G025590/Soltu.DM.11G001010/Soltu.DM.06G012790/Soltu.DM.04G006870/Soltu.DM.01G041980/Soltu.DM.02G002480/Soltu.DM.12G026560/Soltu.DM.05G012040/Soltu.DM.01G024340	9
825	GO:2000280	regulation of root development	83/12756	0.37145933	0.999994594	0.94442346	Soltu.DM.03G017570/Soltu.DM.07G020920/Soltu.DM.10G024780/Soltu.DM.03G037290/Soltu.DM.05G024870/Soltu.DM.08G013420/Soltu.DM.04G022240/Soltu.DM.06G000240/Soltu.DM.10G022070	9
826	GO:0010099	regulation of photomorphogenesis	33/12756	0.372283142	0.999994594	0.94442346	Soltu.DM.10G026220/Soltu.DM.02G002480/Soltu.DM.01G024340/Soltu.DM.06G002140	4
827	GO:0010223	secondary shoot formation	33/12756	0.372283142	0.999994594	0.94442346	Soltu.DM.03G037070/Soltu.DM.06G022310/Soltu.DM.06G025210/Soltu.DM.03G034300	4
828	GO:0010346	shoot axis formation	33/12756	0.372283142	0.999994594	0.94442346	Soltu.DM.03G037070/Soltu.DM.06G022310/Soltu.DM.06G025210/Soltu.DM.03G034300	4
829	GO:0018107	peptidyl-threonine phosphorylation	33/12756	0.372283142	0.999994594	0.94442346	Soltu.DM.06G002800/Soltu.DM.05G023210/Soltu.DM.06G032750/Soltu.DM.06G010880	4
830	GO:0018210	peptidyl-threonine modification	33/12756	0.372283142	0.999994594	0.94442346	Soltu.DM.06G002800/Soltu.DM.05G023210/Soltu.DM.06G032750/Soltu.DM.06G010880	4
831	GO:0050829	defense response to Gram-negative bacterium	33/12756	0.372283142	0.999994594	0.94442346	Soltu.DM.07G006430/Soltu.DM.12G002640/Soltu.DM.09G023660/Soltu.DM.09G029240	4
832	GO:1901570	fatty acid derivative biosynthetic process	33/12756	0.372283142	0.999994594	0.94442346	Soltu.DM.10G005260/Soltu.DM.02G031030/Soltu.DM.02G019940/Soltu.DM.10G005430	4
833	GO:0031122	cytoplasmic microtubule organization	63/12756	0.374442868	0.999994594	0.94442346	Soltu.DM.04G027320/Soltu.DM.12G007610/Soltu.DM.02G028740/Soltu.DM.01G008180/Soltu.DM.04G022240/Soltu.DM.05G009320/Soltu.DM.11G018040	7
834	GO:0030031	cell projection assembly	43/12756	0.374712663	0.999994594	0.94442346	Soltu.DM.01G028770/Soltu.DM.09G019870/Soltu.DM.01G020880/Soltu.DM.07G024370/Soltu.DM.09G027230	5
835	GO:0010927	cellular component assembly involved in morphogenesis	53/12756	0.375117518	0.999994594	0.94442346	Soltu.DM.07G000410/Soltu.DM.03G020790/Soltu.DM.05G025440/Soltu.DM.04G022240/Soltu.DM.02G011180/Soltu.DM.02G031030	6
836	GO:0019318	hexose metabolic process	53/12756	0.375117518	0.999994594	0.94442346	Soltu.DM.09G018910/Soltu.DM.01G040570/Soltu.DM.02G031920/Soltu.DM.04G031580/Soltu.DM.02G031890/Soltu.DM.02G024820	6
837	GO:0070417	cellular response to cold	53/12756	0.375117518	0.999994594	0.94442346	Soltu.DM.07G003530/Soltu.DM.01G040570/Soltu.DM.07G003550/Soltu.DM.03G021360/Soltu.DM.05G011970/Soltu.DM.01G024860	6
83	GO:00	regulation of gene expression by	14/	0.3	0.9	0.9	Soltu.DM.01G024940/Soltu.DM.03G020440	2

8	06349	genomic imprinting	12756	81385426	99994594	4442346		
839	GO:0007097	nuclear migration	14/12756	0.381385426	0.999994594	0.94442346	Soltu.DM.06G022970/Soltu.DM.10G001460	2
840	GO:0009864	induced systemic resistance, jasmonic acid mediated signaling pathway	14/12756	0.381385426	0.999994594	0.94442346	Soltu.DM.02G025590/Soltu.DM.02G022460	2
841	GO:0009866	induced systemic resistance, ethylene mediated signaling pathway	14/12756	0.381385426	0.999994594	0.94442346	Soltu.DM.07G028550/Soltu.DM.10G000640	2
842	GO:0010044	response to aluminum ion	14/12756	0.381385426	0.999994594	0.94442346	Soltu.DM.06G020020/Soltu.DM.03G032030	2
843	GO:0019048	modulation by virus of host process	14/12756	0.381385426	0.999994594	0.94442346	Soltu.DM.02G012280/Soltu.DM.05G026810	2
844	GO:0033260	nuclear DNA replication	14/12756	0.381385426	0.999994594	0.94442346	Soltu.DM.08G027160/Soltu.DM.12G029710	2
845	GO:0035335	peptidyl-tyrosine dephosphorylation	14/12756	0.381385426	0.999994594	0.94442346	Soltu.DM.11G010230/Soltu.DM.11G010220	2
846	GO:0042058	regulation of epidermal growth factor receptor signaling pathway	14/12756	0.381385426	0.999994594	0.94442346	Soltu.DM.08G027150/Soltu.DM.09G002090	2
847	GO:0044773	mitotic DNA damage checkpoint signaling	14/12756	0.381385426	0.999994594	0.94442346	Soltu.DM.11G016820/Soltu.DM.02G033290	2
848	GO:0051647	nucleus localization	14/12756	0.381385426	0.999994594	0.94442346	Soltu.DM.06G022970/Soltu.DM.10G001460	2
849	GO:0061158	3'-UTR-mediated mRNA destabilization	14/12756	0.381385426	0.999994594	0.94442346	Soltu.DM.05G004270/Soltu.DM.08G013620	2
850	GO:0071514	genomic imprinting	14/12756	0.381385426	0.999994594	0.94442346	Soltu.DM.01G024940/Soltu.DM.03G020440	2
851	GO:0071901	negative regulation of protein serine/threonine kinase activity	14/12756	0.381385426	0.999994594	0.94442346	Soltu.DM.11G010230/Soltu.DM.11G010220	2
852	GO:1901184	regulation of ERBB signaling pathway	14/12756	0.381385426	0.999994594	0.94442346	Soltu.DM.08G027150/Soltu.DM.09G002090	2
853	GO:0009968	negative regulation of signal transduction	197/12756	0.382433337	0.999994594	0.94442346	Soltu.DM.07G021000/Soltu.DM.07G012130/Soltu.DM.01G046820/Soltu.DM.03G034960/Soltu.DM.11G010230/Soltu.DM.07G020920/Soltu.DM.10G024780/Soltu.DM.11G010220/Soltu.DM.06G014700/Soltu.DM.06G017300/Soltu.DM.01G000060/Soltu.DM.08G013420/Soltu.DM.04G024100/Soltu.DM.08G011890/Soltu.DM.08G013440/Soltu.DM.07G002580/Soltu.DM.04G005970/Soltu.DM.10G022070/Soltu.DM.07G022640/Soltu.DM.05G025620	20
854	GO:0000255	allantoin metabolic process	5/12756	0.38774441	0.999994594	0.94442346	Soltu.DM.02G017810	1
855	GO:0000290	deadenylation-dependent decapping of nuclear-transcribed mRNA	5/12756	0.3877444	0.9999945	0.9444234	Soltu.DM.05G026810	1

				1	94	6		
856	GO:0000472	endonucleolytic cleavage to generate mature 5'-end of SSU-rRNA from (SSU-rRNA, 5.8S rRNA, LSU-rRNA)	5/12756	0.38774441	0.999994594	0.94442346	Soltu.DM.01G051600	1
857	GO:0000480	endonucleolytic cleavage in 5'-ETS of tricistronic rRNA transcript (SSU-rRNA, 5.8S rRNA, LSU-rRNA)	5/12756	0.38774441	0.999994594	0.94442346	Soltu.DM.01G051600	1
858	GO:0001523	retinoid metabolic process	5/12756	0.38774441	0.999994594	0.94442346	Soltu.DM.03G018850	1
859	GO:0001541	ovarian follicle development	5/12756	0.38774441	0.999994594	0.94442346	Soltu.DM.12G019270	1
860	GO:0002188	translation reinitiation	5/12756	0.38774441	0.999994594	0.94442346	Soltu.DM.08G019530	1
861	GO:0002532	production of molecular mediator involved in inflammatory response	5/12756	0.38774441	0.999994594	0.94442346	Soltu.DM.02G017970	1
862	GO:0002538	arachidonic acid metabolite production involved in inflammatory response	5/12756	0.38774441	0.999994594	0.94442346	Soltu.DM.02G017970	1
863	GO:0002539	prostaglandin production involved in inflammatory response	5/12756	0.38774441	0.999994594	0.94442346	Soltu.DM.02G017970	1
864	GO:0006004	fucose metabolic process	5/12756	0.38774441	0.999994594	0.94442346	Soltu.DM.09G018910	1
865	GO:0006065	UDP-glucuronate biosynthetic process	5/12756	0.38774441	0.999994594	0.94442346	Soltu.DM.02G031050	1
866	GO:0006501	C-terminal protein lipidation	5/12756	0.38774441	0.999994594	0.94442346	Soltu.DM.11G022310	1
867	GO:0006538	glutamate catabolic process	5/12756	0.38774441	0.999994594	0.94442346	Soltu.DM.12G024030	1
868	GO:0006540	glutamate decarboxylation to succinate	5/12756	0.38774441	0.999994594	0.94442346	Soltu.DM.12G024030	1
869	GO:0006977	DNA damage response, signal transduction by p53 class mediator resulting in cell cycle arrest	5/12756	0.38774441	0.999994594	0.94442346	Soltu.DM.11G016820	1
870	GO:0007095	mitotic G2 DNA damage checkpoint signaling	5/12756	0.38774441	0.999994594	0.94442346	Soltu.DM.02G033290	1
871	GO:0007096	regulation of exit from mitosis	5/12756	0.38774441	0.999994594	0.94442346	Soltu.DM.12G023230	1
872	GO:0007269	neurotransmitter secretion	5/12756	0.38774441	0.999994594	0.94442346	Soltu.DM.11G026460	1
873	GO:0009187	cyclic nucleotide metabolic process	5/127	0.3877	0.9999	0.9444	Soltu.DM.03G021730	1

			56	4441	94594	2346		
874	GO:0009413	response to flooding	5/12756	0.38774441	0.999994594	0.94442346	Soltu.DM.07G013360	1
875	GO:0009448	gamma-aminobutyric acid metabolic process	5/12756	0.38774441	0.999994594	0.94442346	Soltu.DM.12G024030	1
876	GO:0009450	gamma-aminobutyric acid catabolic process	5/12756	0.38774441	0.999994594	0.94442346	Soltu.DM.12G024030	1
877	GO:0009643	photosynthetic acclimation	5/12756	0.38774441	0.999994594	0.94442346	Soltu.DM.01G008290	1
878	GO:0009727	detection of ethylene stimulus	5/12756	0.38774441	0.999994594	0.94442346	Soltu.DM.07G022640	1
879	GO:0009810	stilbene metabolic process	5/12756	0.38774441	0.999994594	0.94442346	Soltu.DM.10G004300	1
880	GO:0010068	protoderm histogenesis	5/12756	0.38774441	0.999994594	0.94442346	Soltu.DM.07G020980	1
881	GO:0010097	specification of stamen identity	5/12756	0.38774441	0.999994594	0.94442346	Soltu.DM.05G003100	1
882	GO:0010390	histone monoubiquitination	5/12756	0.38774441	0.999994594	0.94442346	Soltu.DM.01G001590	1
883	GO:0010973	positive regulation of division septum assembly	5/12756	0.38774441	0.999994594	0.94442346	Soltu.DM.12G023230	1
884	GO:0015713	phosphoglycerate transmembrane transport	5/12756	0.38774441	0.999994594	0.94442346	Soltu.DM.01G008290	1
885	GO:0016079	synaptic vesicle exocytosis	5/12756	0.38774441	0.999994594	0.94442346	Soltu.DM.11G026460	1
886	GO:0016255	attachment of GPI anchor to protein	5/12756	0.38774441	0.999994594	0.94442346	Soltu.DM.02G010490	1
887	GO:0017156	calcium-ion regulated exocytosis	5/12756	0.38774441	0.999994594	0.94442346	Soltu.DM.11G026460	1
888	GO:0017157	regulation of exocytosis	5/12756	0.38774441	0.999994594	0.94442346	Soltu.DM.02G022620	1
889	GO:0018410	C-terminal protein amino acid modification	5/12756	0.38774441	0.999994594	0.94442346	Soltu.DM.11G022310	1
890	GO:0018904	ether metabolic process	5/12756	0.38774441	0.999994594	0.94442346	Soltu.DM.10G004300	1
89	GO:00	butyrate metabolic process	5/1	0.3	0.9	0.9	Soltu.DM.02G020870	1

1	19605		2756	8774441	99994594	4442346		
892	GO:0019878	lysine biosynthetic process via aminoadipic acid	5/12756	0.38774441	0.999994594	0.94442346	Soltu.DM.07G006550	1
893	GO:0023061	signal release	5/12756	0.38774441	0.999994594	0.94442346	Soltu.DM.11G026460	1
894	GO:0030850	prostate gland development	5/12756	0.38774441	0.999994594	0.94442346	Soltu.DM.10G022360	1
895	GO:0031028	septation initiation signaling	5/12756	0.38774441	0.999994594	0.94442346	Soltu.DM.12G023230	1
896	GO:0031991	regulation of actomyosin contractile ring contraction	5/12756	0.38774441	0.999994594	0.94442346	Soltu.DM.12G023230	1
897	GO:0032211	negative regulation of telomere maintenance via telomerase	5/12756	0.38774441	0.999994594	0.94442346	Soltu.DM.05G006310	1
898	GO:0032955	regulation of division septum assembly	5/12756	0.38774441	0.999994594	0.94442346	Soltu.DM.12G023230	1
899	GO:0033481	galacturonate biosynthetic process	5/12756	0.38774441	0.999994594	0.94442346	Soltu.DM.12G002640	1
900	GO:0034756	regulation of iron ion transport	5/12756	0.38774441	0.999994594	0.94442346	Soltu.DM.05G011970	1
901	GO:0035150	regulation of tube size	5/12756	0.38774441	0.999994594	0.94442346	Soltu.DM.10G004300	1
902	GO:0035296	regulation of tube diameter	5/12756	0.38774441	0.999994594	0.94442346	Soltu.DM.10G004300	1
903	GO:0035567	non-canonical Wnt signaling pathway	5/12756	0.38774441	0.999994594	0.94442346	Soltu.DM.07G002580	1
904	GO:0035627	ceramide transport	5/12756	0.38774441	0.999994594	0.94442346	Soltu.DM.10G022360	1
905	GO:0042572	retinol metabolic process	5/12756	0.38774441	0.999994594	0.94442346	Soltu.DM.03G018850	1
906	GO:0042632	cholesterol homeostasis	5/12756	0.38774441	0.999994594	0.94442346	Soltu.DM.10G004300	1
907	GO:0042771	intrinsic apoptotic signaling pathway in response to DNA damage by p53 class mediator	5/12756	0.38774441	0.999994594	0.94442346	Soltu.DM.12G025260	1
908	GO:0044375	regulation of peroxisome size	5/12756	0.38774441	0.999994594	0.94442346	Soltu.DM.10G007000	1

909	GO:0045026	plasma membrane fusion	5/12756	0.38774441	0.999994594	0.94442346	Soltu.DM.04G000190	1
910	GO:0045038	protein import into chloroplast thylakoid membrane	5/12756	0.38774441	0.999994594	0.94442346	Soltu.DM.02G032340	1
911	GO:0045765	regulation of angiogenesis	5/12756	0.38774441	0.999994594	0.94442346	Soltu.DM.02G018520	1
912	GO:0045777	positive regulation of blood pressure	5/12756	0.38774441	0.999994594	0.94442346	Soltu.DM.02G017970	1
913	GO:0046272	stilbene catabolic process	5/12756	0.38774441	0.999994594	0.94442346	Soltu.DM.10G004300	1
914	GO:0046439	L-cysteine metabolic process	5/12756	0.38774441	0.999994594	0.94442346	Soltu.DM.12G002620	1
915	GO:0046498	S-adenosylhomocysteine metabolic process	5/12756	0.38774441	0.999994594	0.94442346	Soltu.DM.12G002620	1
916	GO:0048145	regulation of fibroblast proliferation	5/12756	0.38774441	0.999994594	0.94442346	Soltu.DM.02G018520	1
917	GO:0048445	carpel morphogenesis	5/12756	0.38774441	0.999994594	0.94442346	Soltu.DM.05G003100	1
918	GO:0048530	fruit morphogenesis	5/12756	0.38774441	0.999994594	0.94442346	Soltu.DM.09G022610	1
919	GO:0050686	negative regulation of mRNA processing	5/12756	0.38774441	0.999994594	0.94442346	Soltu.DM.10G001400	1
920	GO:0050764	regulation of phagocytosis	5/12756	0.38774441	0.999994594	0.94442346	Soltu.DM.10G004310	1
921	GO:0051764	actin crosslink formation	5/12756	0.38774441	0.999994594	0.94442346	Soltu.DM.07G026780	1
922	GO:0055092	sterol homeostasis	5/12756	0.38774441	0.999994594	0.94442346	Soltu.DM.10G004300	1
923	GO:0060742	epithelial cell differentiation involved in prostate gland development	5/12756	0.38774441	0.999994594	0.94442346	Soltu.DM.10G022360	1
924	GO:0061088	regulation of sequestering of zinc ion	5/12756	0.38774441	0.999994594	0.94442346	Soltu.DM.07G002440	1
925	GO:0061936	fusion of sperm to egg plasma membrane involved in double fertilization forming a zygote and endosperm	5/12756	0.38774441	0.999994594	0.94442346	Soltu.DM.04G000190	1
926	GO:0070574	cadmium ion transmembrane transport	5/12756	0.3877444	0.9999945	0.9444234	Soltu.DM.03G035710	1

				1	94	6		
92 7	GO:00 71366	cellular response to indolebutyric acid stimulus	5/1 27 56	0.3 877 444 1	0.9 999 945 94	0.9 444 234 6	Soltu.DM.03G035710	1
92 8	GO:00 71452	cellular response to singlet oxygen	5/1 27 56	0.3 877 444 1	0.9 999 945 94	0.9 444 234 6	Soltu.DM.06G026560	1
92 9	GO:00 72089	stem cell proliferation	5/1 27 56	0.3 877 444 1	0.9 999 945 94	0.9 444 234 6	Soltu.DM.07G002580	1
93 0	GO:00 72334	UDP-galactose transmembrane transport	5/1 27 56	0.3 877 444 1	0.9 999 945 94	0.9 444 234 6	Soltu.DM.12G004060	1
93 1	GO:00 90669	telomerase RNA stabilization	5/1 27 56	0.3 877 444 1	0.9 999 945 94	0.9 444 234 6	Soltu.DM.01G002690	1
93 2	GO:00 97176	epoxide metabolic process	5/1 27 56	0.3 877 444 1	0.9 999 945 94	0.9 444 234 6	Soltu.DM.10G004300	1
93 3	GO:00 97746	blood vessel diameter maintenance	5/1 27 56	0.3 877 444 1	0.9 999 945 94	0.9 444 234 6	Soltu.DM.10G004300	1
93 4	GO:00 99643	signal release from synapse	5/1 27 56	0.3 877 444 1	0.9 999 945 94	0.9 444 234 6	Soltu.DM.11G026460	1
93 5	GO:19 00871	chloroplast mRNA modification	5/1 27 56	0.3 877 444 1	0.9 999 945 94	0.9 444 234 6	Soltu.DM.03G033060	1
93 6	GO:19 01031	regulation of response to reactive oxygen species	5/1 27 56	0.3 877 444 1	0.9 999 945 94	0.9 444 234 6	Soltu.DM.07G015200	1
93 7	GO:19 01342	regulation of vasculature development	5/1 27 56	0.3 877 444 1	0.9 999 945 94	0.9 444 234 6	Soltu.DM.02G018520	1
93 8	GO:19 01891	regulation of cell septum assembly	5/1 27 56	0.3 877 444 1	0.9 999 945 94	0.9 444 234 6	Soltu.DM.12G023230	1
93 9	GO:19 01893	positive regulation of cell septum assembly	5/1 27 56	0.3 877 444 1	0.9 999 945 94	0.9 444 234 6	Soltu.DM.12G023230	1
94 0	GO:19 01999	homogentisate metabolic process	5/1 27 56	0.3 877 444 1	0.9 999 945 94	0.9 444 234 6	Soltu.DM.04G009170	1
94 1	GO:19 02000	homogentisate catabolic process	5/1 27 56	0.3 877 444 1	0.9 999 945 94	0.9 444 234 6	Soltu.DM.04G009170	1
94 2	GO:19 02932	positive regulation of alcohol biosynthetic process	5/1 27 56	0.3 877 444 1	0.9 999 945 94	0.9 444 234 6	Soltu.DM.02G020950	1
94 3	GO:19 03438	positive regulation of mitotic cytokinetic process	5/1 27 56	0.3 877 444 1	0.9 999 945 94	0.9 444 234 6	Soltu.DM.12G023230	1
94 4	GO:19 03490	positive regulation of mitotic cytokinesis	5/1 27	0.3 877	0.9 999	0.9 444	Soltu.DM.12G023230	1

			56	4441	94594	2346		
945	GO:1904502	regulation of lipophagy	5/12756	0.38774441	0.999994594	0.94442346	Soltu.DM.08G014180	1
946	GO:1904504	positive regulation of lipophagy	5/12756	0.38774441	0.999994594	0.94442346	Soltu.DM.08G014180	1
947	GO:0009756	carbohydrate mediated signaling	74/12756	0.387787232	0.999994594	0.94442346	Soltu.DM.06G026960/Soltu.DM.01G006210/Soltu.DM.07G020920/Soltu.DM.10G024780/Soltu.DM.08G013420/Soltu.DM.02G002480/Soltu.DM.10G022070/Soltu.DM.07G022640	8
948	GO:0010182	sugar mediated signaling pathway	74/12756	0.387787232	0.999994594	0.94442346	Soltu.DM.06G026960/Soltu.DM.01G006210/Soltu.DM.07G020920/Soltu.DM.10G024780/Soltu.DM.08G013420/Soltu.DM.02G002480/Soltu.DM.10G022070/Soltu.DM.07G022640	8
949	GO:0009165	nucleotide biosynthetic process	146/12756	0.389530743	0.999994594	0.94442346	Soltu.DM.05G011440/Soltu.DM.12G004480/Soltu.DM.03G037170/Soltu.DM.07G009580/Soltu.DM.09G006670/Soltu.DM.11G007630/Soltu.DM.10G027910/Soltu.DM.06G013720/Soltu.DM.03G021730/Soltu.DM.01G017170/Soltu.DM.02G031030/Soltu.DM.11G025570/Soltu.DM.05G023970/Soltu.DM.11G010590/Soltu.DM.01G019520	15
950	GO:1901293	nucleoside phosphate biosynthetic process	146/12756	0.389530743	0.999994594	0.94442346	Soltu.DM.05G011440/Soltu.DM.12G004480/Soltu.DM.03G037170/Soltu.DM.07G009580/Soltu.DM.09G006670/Soltu.DM.11G007630/Soltu.DM.10G027910/Soltu.DM.06G013720/Soltu.DM.03G021730/Soltu.DM.01G017170/Soltu.DM.02G031030/Soltu.DM.11G025570/Soltu.DM.05G023970/Soltu.DM.11G010590/Soltu.DM.01G019520	15
951	GO:0019932	second-messenger-mediated signaling	64/12756	0.390239928	0.999994594	0.94442346	Soltu.DM.02G018520/Soltu.DM.01G051900/Soltu.DM.10G026210/Soltu.DM.01G039230/Soltu.DM.02G028750/Soltu.DM.08G015350/Soltu.DM.02G022460	7
952	GO:0042455	ribonucleoside biosynthetic process	24/12756	0.391817521	0.999994594	0.94442346	Soltu.DM.03G037170/Soltu.DM.10G027910/Soltu.DM.01G017170	3
953	GO:0051961	negative regulation of nervous system development	24/12756	0.391817521	0.999994594	0.94442346	Soltu.DM.12G020370/Soltu.DM.12G020350/Soltu.DM.12G020340	3
954	GO:0062014	negative regulation of small molecule metabolic process	24/12756	0.391817521	0.999994594	0.94442346	Soltu.DM.03G035070/Soltu.DM.03G035080/Soltu.DM.11G018040	3
955	GO:1900673	olefin metabolic process	24/12756	0.391817521	0.999994594	0.94442346	Soltu.DM.11G009500/Soltu.DM.02G004480/Soltu.DM.10G004300	3
956	GO:0009119	ribonucleoside metabolic process	44/12756	0.393910183	0.999994594	0.94442346	Soltu.DM.03G037170/Soltu.DM.10G027910/Soltu.DM.12G003790/Soltu.DM.12G002620/Soltu.DM.01G017170	5
957	GO:0048508	embryonic meristem development	44/12756	0.393910183	0.999994594	0.94442346	Soltu.DM.02G003130/Soltu.DM.05G026810/Soltu.DM.07G020980/Soltu.DM.06G034230/Soltu.DM.03G034800	5
958	GO:0051865	protein autoubiquitination	44/12756	0.393910183	0.999994594	0.94442346	Soltu.DM.04G033430/Soltu.DM.06G031440/Soltu.DM.06G015770/Soltu.DM.09G005140/Soltu.DM.05G005120	5
959	GO:0120255	olefinic compound biosynthetic process	44/12756	0.393910183	0.999994594	0.94442346	Soltu.DM.11G024450/Soltu.DM.11G009500/Soltu.DM.02G004480/Soltu.DM.06G029640/Soltu.DM.06G020450	5
960	GO:1901616	organic hydroxy compound catabolic process	44/12756	0.393910183	0.999994594	0.94442346	Soltu.DM.06G028410/Soltu.DM.08G020150/Soltu.DM.07G013940/Soltu.DM.07G013900/Soltu.DM.04G009170	5
961	GO:0006986	response to unfolded protein	34/12756	0.394236774	0.999994594	0.94442346	Soltu.DM.07G011880/Soltu.DM.04G037150/Soltu.DM.08G029860/Soltu.DM.08G019590	4
96	GO:00	regulation of cell size	34/	0.3	0.9	0.9	Soltu.DM.12G020370/Soltu.DM.12G020350/Soltu.DM.05G027000/Soltu.DM.12G020340	4

2	08361		12756	94236774	99994594	4442346		
963	GO:0009851	auxin biosynthetic process	34/12756	0.394236774	0.999994594	0.94442346	Soltu.DM.11G024450/Soltu.DM.03G035070/Soltu.DM.06G019760/Soltu.DM.03G035080	4
964	GO:0010720	positive regulation of cell development	34/12756	0.394236774	0.999994594	0.94442346	Soltu.DM.12G020370/Soltu.DM.12G020350/Soltu.DM.12G025260/Soltu.DM.12G020340	4
965	GO:2000069	regulation of post-embryonic root development	34/12756	0.394236774	0.999994594	0.94442346	Soltu.DM.07G020920/Soltu.DM.10G024780/Soltu.DM.08G013420/Soltu.DM.10G022070	4
966	GO:0010941	regulation of cell death	157/12756	0.396202185	0.999994594	0.94442346	Soltu.DM.09G024260/Soltu.DM.02G018520/Soltu.DM.08G014180/Soltu.DM.05G022160/Soltu.DM.08G022900/Soltu.DM.06G028580/Soltu.DM.06G024610/Soltu.DM.08G028440/Soltu.DM.03G032350/Soltu.DM.06G026400/Soltu.DM.01G044520/Soltu.DM.04G022240/Soltu.DM.07G014680/Soltu.DM.07G024240/Soltu.DM.09G024270/Soltu.DM.06G024530	16
967	GO:0008643	carbohydrate transport	75/12756	0.402452122	0.999994594	0.94442346	Soltu.DM.02G014590/Soltu.DM.09G024150/Soltu.DM.01G008290/Soltu.DM.02G006700/Soltu.DM.03G020090/Soltu.DM.02G018010/Soltu.DM.03G031200/Soltu.DM.01G039610	8
968	GO:0006972	hyperosmotic response	96/12756	0.408385515	0.999994594	0.94442346	Soltu.DM.03G020450/Soltu.DM.03G017570/Soltu.DM.02G020550/Soltu.DM.08G019590/Soltu.DM.01G046560/Soltu.DM.05G018830/Soltu.DM.05G009320/Soltu.DM.08G007450/Soltu.DM.05G018810/Soltu.DM.07G014750	10
969	GO:0032271	regulation of protein polymerization	55/12756	0.409618685	0.999994594	0.94442346	Soltu.DM.06G022970/Soltu.DM.01G028770/Soltu.DM.09G015150/Soltu.DM.12G009990/Soltu.DM.10G004310/Soltu.DM.09G027230	6
970	GO:0034293	sexual sporulation	55/12756	0.409618685	0.999994594	0.94442346	Soltu.DM.06G009270/Soltu.DM.09G031520/Soltu.DM.05G022790/Soltu.DM.02G011180/Soltu.DM.09G031510/Soltu.DM.12G023840	6
971	GO:0051788	response to misfolded protein	45/12756	0.413055601	0.999994594	0.94442346	Soltu.DM.03G016820/Soltu.DM.06G012970/Soltu.DM.10G004220/Soltu.DM.05G005120/Soltu.DM.06G009790	5
972	GO:0002238	response to molecule of fungal origin	15/12756	0.415555765	0.999994594	0.94442346	Soltu.DM.01G047750/Soltu.DM.06G010530	2
973	GO:0006513	protein monoubiquitination	15/12756	0.415555765	0.999994594	0.94442346	Soltu.DM.01G001590/Soltu.DM.02G033290	2
974	GO:0007035	vacuolar acidification	15/12756	0.415555765	0.999994594	0.94442346	Soltu.DM.02G024200/Soltu.DM.07G009580	2
975	GO:0009554	megasporogenesis	15/12756	0.415555765	0.999994594	0.94442346	Soltu.DM.09G031520/Soltu.DM.09G031510	2
976	GO:0010232	vascular transport	15/12756	0.415555765	0.999994594	0.94442346	Soltu.DM.03G027330/Soltu.DM.03G027340	2
977	GO:0010233	phloem transport	15/12756	0.415555765	0.999994594	0.94442346	Soltu.DM.03G027330/Soltu.DM.03G027340	2
978	GO:0010929	positive regulation of auxin mediated signaling pathway	15/12756	0.415555765	0.999994594	0.94442346	Soltu.DM.04G033180/Soltu.DM.03G036780	2
979	GO:0032210	regulation of telomere maintenance via telomerase	15/12756	0.415555765	0.999994594	0.94442346	Soltu.DM.05G006310/Soltu.DM.02G013390	2

980	GO:0035067	negative regulation of histone acetylation	15/12756	0.415555765	0.999994594	0.944423466	Soltu.DM.08G022190/Soltu.DM.04G038280	2
981	GO:0043489	RNA stabilization	15/12756	0.415555765	0.999994594	0.944423466	Soltu.DM.11G016820/Soltu.DM.01G002690	2
982	GO:0046482	para-aminobenzoic acid metabolic process	15/12756	0.415555765	0.999994594	0.944423466	Soltu.DM.05G007640/Soltu.DM.05G007630	2
983	GO:0046520	sphingoid biosynthetic process	15/12756	0.415555765	0.999994594	0.944423466	Soltu.DM.02G018520/Soltu.DM.08G014180	2
984	GO:0051365	cellular response to potassium ion starvation	15/12756	0.415555765	0.999994594	0.944423466	Soltu.DM.07G028550/Soltu.DM.10G000640	2
985	GO:0051452	intracellular pH reduction	15/12756	0.415555765	0.999994594	0.944423466	Soltu.DM.02G024200/Soltu.DM.07G009580	2
986	GO:0072529	pyrimidine-containing compound catabolic process	15/12756	0.415555765	0.999994594	0.944423466	Soltu.DM.10G012990/Soltu.DM.03G019550	2
987	GO:1901984	negative regulation of protein acetylation	15/12756	0.415555765	0.999994594	0.944423466	Soltu.DM.08G022190/Soltu.DM.04G038280	2
988	GO:1902369	negative regulation of RNA catabolic process	15/12756	0.415555765	0.999994594	0.944423466	Soltu.DM.11G016820/Soltu.DM.01G002690	2
989	GO:2000757	negative regulation of peptidyl-lysine acetylation	15/12756	0.415555765	0.999994594	0.944423466	Soltu.DM.08G022190/Soltu.DM.04G038280	2
990	GO:0006637	acyl-CoA metabolic process	35/12756	0.416066494	0.999994594	0.944423466	Soltu.DM.01G033530/Soltu.DM.08G026810/Soltu.DM.02G031030/Soltu.DM.01G019520	4
991	GO:0035383	thioester metabolic process	35/12756	0.416066494	0.999994594	0.944423466	Soltu.DM.01G033530/Soltu.DM.08G026810/Soltu.DM.02G031030/Soltu.DM.01G019520	4
992	GO:0051046	regulation of secretion	35/12756	0.416066494	0.999994594	0.944423466	Soltu.DM.08G027150/Soltu.DM.01G002690/Soltu.DM.09G002090/Soltu.DM.02G022620	4
993	GO:1903530	regulation of secretion by cell	35/12756	0.416066494	0.999994594	0.944423466	Soltu.DM.08G027150/Soltu.DM.01G002690/Soltu.DM.09G002090/Soltu.DM.02G022620	4
994	GO:0051701	biological process involved in interaction with host	76/12756	0.417116451	0.999994594	0.944423466	Soltu.DM.01G024680/Soltu.DM.02G012280/Soltu.DM.05G026810/Soltu.DM.07G014680/Soltu.DM.01G005590/Soltu.DM.03G024130/Soltu.DM.01G010020/Soltu.DM.11G004150	8
995	GO:0009403	toxin biosynthetic process	25/12756	0.417860988	0.999994594	0.944423466	Soltu.DM.12G022190/Soltu.DM.06G018840/Soltu.DM.07G014750	3
996	GO:0009944	polarity specification of adaxial/abaxial axis	25/12756	0.417860988	0.999994594	0.944423466	Soltu.DM.02G003130/Soltu.DM.07G026690/Soltu.DM.08G012010	3
997	GO:0032104	regulation of response to extracellular stimulus	25/1275	0.4178609	0.9999945	0.9444234	Soltu.DM.01G008040/Soltu.DM.12G025260/Soltu.DM.05G001260	3

			6	88	94	6		
998	GO:0032107	regulation of response to nutrient levels	25/12756	0.417860988	0.999994594	0.94442346	Soltu.DM.01G008040/Soltu.DM.12G025260/Soltu.DM.05G001260	3
999	GO:0043467	regulation of generation of precursor metabolites and energy	25/12756	0.417860988	0.999994594	0.94442346	Soltu.DM.07G012130/Soltu.DM.07G000550/Soltu.DM.04G037460	3
1000	GO:0043967	histone H4 acetylation	25/12756	0.417860988	0.999994594	0.94442346	Soltu.DM.06G019850/Soltu.DM.04G033160/Soltu.DM.09G019870	3
1001	GO:0046839	phospholipid dephosphorylation	25/12756	0.417860988	0.999994594	0.94442346	Soltu.DM.02G017970/Soltu.DM.10G004300/Soltu.DM.04G034770	3
1002	GO:0006164	purine nucleotide biosynthetic process	97/12756	0.421365654	0.999994594	0.94442346	Soltu.DM.12G004480/Soltu.DM.03G037170/Soltu.DM.07G009580/Soltu.DM.10G027910/Soltu.DM.06G013720/Soltu.DM.03G021730/Soltu.DM.02G031030/Soltu.DM.11G025570/Soltu.DM.11G010590/Soltu.DM.01G019520	10
1003	GO:0008064	regulation of actin polymerization or depolymerization	56/12756	0.426815028	0.999994594	0.94442346	Soltu.DM.06G022970/Soltu.DM.01G028770/Soltu.DM.09G015150/Soltu.DM.12G009990/Soltu.DM.10G004310/Soltu.DM.09G027230	6
1004	GO:0019722	calcium-mediated signaling	56/12756	0.426815028	0.999994594	0.94442346	Soltu.DM.02G018520/Soltu.DM.01G051900/Soltu.DM.10G026210/Soltu.DM.01G039230/Soltu.DM.02G028750/Soltu.DM.02G022460	6
1005	GO:0030832	regulation of actin filament length	56/12756	0.426815028	0.999994594	0.94442346	Soltu.DM.06G022970/Soltu.DM.01G028770/Soltu.DM.09G015150/Soltu.DM.12G009990/Soltu.DM.10G004310/Soltu.DM.09G027230	6
1006	GO:0006643	membrane lipid metabolic process	108/12756	0.428971642	0.999994594	0.94442346	Soltu.DM.08G001900/Soltu.DM.04G008710/Soltu.DM.01G042210/Soltu.DM.02G018520/Soltu.DM.08G014180/Soltu.DM.01G047750/Soltu.DM.10G022360/Soltu.DM.07G015200/Soltu.DM.02G010490/Soltu.DM.10G020020/Soltu.DM.05G012150	11
1007	GO:0045859	regulation of protein kinase activity	108/12756	0.428971642	0.999994594	0.94442346	Soltu.DM.07G017210/Soltu.DM.07G017190/Soltu.DM.07G017200/Soltu.DM.07G017180/Soltu.DM.07G012130/Soltu.DM.06G018320/Soltu.DM.11G010230/Soltu.DM.02G028740/Soltu.DM.11G010220/Soltu.DM.06G028580/Soltu.DM.04G038280	11
1008	GO:0009553	embryo sac development	150/12756	0.431311754	0.999994594	0.94442346	Soltu.DM.06G026960/Soltu.DM.06G009270/Soltu.DM.02G020320/Soltu.DM.05G023990/Soltu.DM.09G031520/Soltu.DM.02G012680/Soltu.DM.08G006060/Soltu.DM.01G002850/Soltu.DM.03G022850/Soltu.DM.05G022790/Soltu.DM.12G002630/Soltu.DM.02G020340/Soltu.DM.10G014590/Soltu.DM.08G026840/Soltu.DM.09G031510	15
1009	GO:0000096	sulfur amino acid metabolic process	46/12756	0.43210657	0.999994594	0.94442346	Soltu.DM.03G035070/Soltu.DM.08G030020/Soltu.DM.03G035080/Soltu.DM.12G002620/Soltu.DM.12G025770	5
1010	GO:0034605	cellular response to heat	46/12756	0.43210657	0.999994594	0.94442346	Soltu.DM.07G011880/Soltu.DM.09G025070/Soltu.DM.06G018840/Soltu.DM.01G024860/Soltu.DM.08G012010	5
1011	GO:0006633	fatty acid biosynthetic process	98/12756	0.43433737	0.999994594	0.94442346	Soltu.DM.06G009560/Soltu.DM.04G008710/Soltu.DM.07G003270/Soltu.DM.01G047750/Soltu.DM.10G004300/Soltu.DM.10G026590/Soltu.DM.07G026100/Soltu.DM.07G000550/Soltu.DM.10G026480/Soltu.DM.02G019940	10
1012	GO:0009259	ribonucleotide metabolic process	161/12756	0.436618227	0.999994594	0.94442346	Soltu.DM.12G004480/Soltu.DM.01G033530/Soltu.DM.02G018700/Soltu.DM.03G037170/Soltu.DM.07G009580/Soltu.DM.11G007630/Soltu.DM.10G027910/Soltu.DM.06G013720/Soltu.DM.03G021730/Soltu.DM.01G017170/Soltu.DM.08G026810/Soltu.DM.02G031030/Soltu.DM.11G025570/Soltu.DM.01G023860/Soltu.DM.11G010590/Soltu.DM.01G019520	16
1013	GO:0001763	morphogenesis of a branching structure	36/12756	0.437712973	0.999994594	0.94442346	Soltu.DM.03G037070/Soltu.DM.06G022310/Soltu.DM.06G025210/Soltu.DM.03G034300	4
1014	GO:0016998	cell wall macromolecule catabolic process	36/12756	0.437712973	0.999994594	0.94442346	Soltu.DM.06G006590/Soltu.DM.11G000740/Soltu.DM.09G005280/Soltu.DM.06G006580	4
1015	GO:0019693	ribose phosphate metabolic process	172/1	0.4415	0.9999	0.9444	Soltu.DM.08G014620/Soltu.DM.12G004480/Soltu.DM.01G033530/Soltu.DM.02G018700/Soltu.DM.03G037170/Soltu.DM.07G009580/Soltu.DM.11G007630/Soltu.DM.10G027910/Sol	17

			2756	22894	94594	2346	tu.DM.06G013720/Soltu.DM.03G021730/Soltu.DM.01G017170/Soltu.DM.08G026810/Soltu.DM.02G031030/Soltu.DM.11G025570/Soltu.DM.01G023860/Soltu.DM.11G010590/Soltu.DM.01G019520	
1016	GO:0005983	starch catabolic process	26/12756	0.443525294	0.999994594	0.94442346	Soltu.DM.09G027770/Soltu.DM.05G006330/Soltu.DM.04G037250	3
1017	GO:0009163	nucleoside biosynthetic process	26/12756	0.443525294	0.999994594	0.94442346	Soltu.DM.03G037170/Soltu.DM.10G027910/Soltu.DM.01G017170	3
1018	GO:0010345	suberin biosynthetic process	26/12756	0.443525294	0.999994594	0.94442346	Soltu.DM.05G025440/Soltu.DM.10G026590/Soltu.DM.03G000410	3
1019	GO:0019432	triglyceride biosynthetic process	26/12756	0.443525294	0.999994594	0.94442346	Soltu.DM.12G002120/Soltu.DM.01G034820/Soltu.DM.10G005430	3
1020	GO:0032204	regulation of telomere maintenance	26/12756	0.443525294	0.999994594	0.94442346	Soltu.DM.05G006310/Soltu.DM.01G002690/Soltu.DM.02G013390	3
1021	GO:0071732	cellular response to nitric oxide	26/12756	0.443525294	0.999994594	0.94442346	Soltu.DM.07G028550/Soltu.DM.10G000640/Soltu.DM.02G030410	3
1022	GO:0000395	mRNA 5'-splice site recognition	6/12756	0.444979816	0.999994594	0.94442346	Soltu.DM.10G001400	1
1023	GO:0001516	prostaglandin biosynthetic process	6/12756	0.444979816	0.999994594	0.94442346	Soltu.DM.02G019940	1
1024	GO:0002576	platelet degranulation	6/12756	0.444979816	0.999994594	0.94442346	Soltu.DM.10G022360	1
1025	GO:0006097	glyoxylate cycle	6/12756	0.444979816	0.999994594	0.94442346	Soltu.DM.07G017900	1
1026	GO:0006285	base-excision repair, AP site formation	6/12756	0.444979816	0.999994594	0.94442346	Soltu.DM.05G007660	1
1027	GO:0006388	tRNA splicing, via endonucleolytic cleavage and ligation	6/12756	0.444979816	0.999994594	0.94442346	Soltu.DM.01G006380	1
1028	GO:0006552	leucine catabolic process	6/12756	0.444979816	0.999994594	0.94442346	Soltu.DM.01G047450	1
1029	GO:0006771	riboflavin metabolic process	6/12756	0.444979816	0.999994594	0.94442346	Soltu.DM.01G044760	1
1030	GO:0006880	intracellular sequestering of iron ion	6/12756	0.444979816	0.999994594	0.94442346	Soltu.DM.04G003430	1
1031	GO:0008585	female gonad development	6/12756	0.444979816	0.999994594	0.94442346	Soltu.DM.12G019270	1
1032	GO:0008630	intrinsic apoptotic signaling pathway in response to DNA damage	6/12756	0.444979816	0.999994594	0.94442346	Soltu.DM.12G025260	1

1033	GO:0009231	riboflavin biosynthetic process	6/12756	0.444979816	0.999994594	0.94442346	Soltu.DM.01G044760	1
1034	GO:0009865	pollen tube adhesion	6/12756	0.444979816	0.999994594	0.94442346	Soltu.DM.12G024030	1
1035	GO:0009957	epidermal cell fate specification	6/12756	0.444979816	0.999994594	0.94442346	Soltu.DM.10G023760	1
1036	GO:0009988	cell-cell recognition	6/12756	0.444979816	0.999994594	0.94442346	Soltu.DM.04G000190	1
1037	GO:0010117	photoprotection	6/12756	0.444979816	0.999994594	0.94442346	Soltu.DM.09G025070	1
1038	GO:0010376	stomatal complex formation	6/12756	0.444979816	0.999994594	0.94442346	Soltu.DM.12G010960	1
1039	GO:0015717	triose phosphate transport	6/12756	0.444979816	0.999994594	0.94442346	Soltu.DM.01G008290	1
1040	GO:0016055	Wnt signaling pathway	6/12756	0.444979816	0.999994594	0.94442346	Soltu.DM.07G002580	1
1041	GO:0016078	tRNA catabolic process	6/12756	0.444979816	0.999994594	0.94442346	Soltu.DM.11G004920	1
1042	GO:0016239	positive regulation of macroautophagy	6/12756	0.444979816	0.999994594	0.94442346	Soltu.DM.08G014180	1
1043	GO:0019253	reductive pentose-phosphate cycle	6/12756	0.444979816	0.999994594	0.94442346	Soltu.DM.01G024860	1
1044	GO:0019685	photosynthesis, dark reaction	6/12756	0.444979816	0.999994594	0.94442346	Soltu.DM.01G024860	1
1045	GO:0030026	cellular manganese ion homeostasis	6/12756	0.444979816	0.999994594	0.94442346	Soltu.DM.04G003430	1
1046	GO:0030038	contractile actin filament bundle assembly	6/12756	0.444979816	0.999994594	0.94442346	Soltu.DM.01G028770	1
1047	GO:0030643	cellular phosphate ion homeostasis	6/12756	0.444979816	0.999994594	0.94442346	Soltu.DM.02G034460	1
1048	GO:0031270	pseudopodium retraction	6/12756	0.444979816	0.999994594	0.94442346	Soltu.DM.01G028770	1
1049	GO:0032060	bleb assembly	6/12756	0.444979816	0.999994594	0.94442346	Soltu.DM.01G028770	1
1050	GO:0032954	regulation of cytokinetic process	6/12756	0.4449798	0.9999945	0.9444234	Soltu.DM.12G023230	1

				16	94	6		
1051	GO:0033298	contractile vacuole organization	6/12756	0.444979816	0.999994594	0.94442346	Soltu.DM.01G028770	1
1052	GO:0034085	establishment of sister chromatid cohesion	6/12756	0.444979816	0.999994594	0.94442346	Soltu.DM.06G019850	1
1053	GO:0034086	maintenance of sister chromatid cohesion	6/12756	0.444979816	0.999994594	0.94442346	Soltu.DM.09G014080	1
1054	GO:0034314	Arp2/3 complex-mediated actin nucleation	6/12756	0.444979816	0.999994594	0.94442346	Soltu.DM.09G027230	1
1055	GO:0035436	triose phosphate transmembrane transport	6/12756	0.444979816	0.999994594	0.94442346	Soltu.DM.01G008290	1
1056	GO:0035445	borate transmembrane transport	6/12756	0.444979816	0.999994594	0.94442346	Soltu.DM.03G031200	1
1057	GO:0042344	indole glucosinolate catabolic process	6/12756	0.444979816	0.999994594	0.94442346	Soltu.DM.03G035710	1
1058	GO:0042873	aldonate transmembrane transport	6/12756	0.444979816	0.999994594	0.94442346	Soltu.DM.01G008290	1
1059	GO:0045604	regulation of epidermal cell differentiation	6/12756	0.444979816	0.999994594	0.94442346	Soltu.DM.10G024000	1
1060	GO:0046136	positive regulation of vitamin metabolic process	6/12756	0.444979816	0.999994594	0.94442346	Soltu.DM.03G008510	1
1061	GO:0046398	UDP-glucuronate metabolic process	6/12756	0.444979816	0.999994594	0.94442346	Soltu.DM.02G031050	1
1062	GO:0046456	icosanoid biosynthetic process	6/12756	0.444979816	0.999994594	0.94442346	Soltu.DM.02G019940	1
1063	GO:0046457	prostanoid biosynthetic process	6/12756	0.444979816	0.999994594	0.94442346	Soltu.DM.02G019940	1
1064	GO:0046545	development of primary female sexual characteristics	6/12756	0.444979816	0.999994594	0.94442346	Soltu.DM.12G019270	1
1065	GO:0046713	borate transport	6/12756	0.444979816	0.999994594	0.94442346	Soltu.DM.03G031200	1
1066	GO:0046836	glycolipid transport	6/12756	0.444979816	0.999994594	0.94442346	Soltu.DM.10G022360	1
1067	GO:0046847	filopodium assembly	6/12756	0.444979816	0.999994594	0.94442346	Soltu.DM.01G028770	1
1068	GO:0048026	positive regulation of mRNA splicing, via spliceosome	6/127	0.4449	0.9999	0.9444	Soltu.DM.12G025260	1

			56	798 16	945 94	234 6		
1069	GO:0051639	actin filament network formation	6/12756	0.444979816	0.999994594	0.94442346	Soltu.DM.07G026780	1
1070	GO:0051974	negative regulation of telomerase activity	6/12756	0.444979816	0.999994594	0.94442346	Soltu.DM.02G013390	1
1071	GO:0060070	canonical Wnt signaling pathway	6/12756	0.444979816	0.999994594	0.94442346	Soltu.DM.07G002580	1
1072	GO:0060327	cytoplasmic actin-based contraction involved in cell motility	6/12756	0.444979816	0.999994594	0.94442346	Soltu.DM.01G028770	1
1073	GO:0060328	cytoplasmic actin-based contraction involved in forward cell motility	6/12756	0.444979816	0.999994594	0.94442346	Soltu.DM.01G028770	1
1074	GO:0070613	regulation of protein processing	6/12756	0.444979816	0.999994594	0.94442346	Soltu.DM.06G018040	1
1075	GO:0071324	cellular response to disaccharide stimulus	6/12756	0.444979816	0.999994594	0.94442346	Soltu.DM.05G011970	1
1076	GO:0071329	cellular response to sucrose stimulus	6/12756	0.444979816	0.999994594	0.94442346	Soltu.DM.05G011970	1
1077	GO:0071486	cellular response to high light intensity	6/12756	0.444979816	0.999994594	0.94442346	Soltu.DM.09G025070	1
1078	GO:0072708	response to sorbitol	6/12756	0.444979816	0.999994594	0.94442346	Soltu.DM.07G013360	1
1079	GO:0080005	photosystem stoichiometry adjustment	6/12756	0.444979816	0.999994594	0.94442346	Soltu.DM.06G028040	1
1080	GO:0080110	sporopollenin biosynthetic process	6/12756	0.444979816	0.999994594	0.94442346	Soltu.DM.02G031030	1
1081	GO:0080117	secondary growth	6/12756	0.444979816	0.999994594	0.94442346	Soltu.DM.05G011970	1
1082	GO:0080154	regulation of fertilization	6/12756	0.444979816	0.999994594	0.94442346	Soltu.DM.09G022610	1
1083	GO:0080190	lateral growth	6/12756	0.444979816	0.999994594	0.94442346	Soltu.DM.05G011970	1
1084	GO:0090100	positive regulation of transmembrane receptor protein serine/threonine kinase signaling pathway	6/12756	0.444979816	0.999994594	0.94442346	Soltu.DM.12G025260	1
1085	GO:0097577	sequestering of iron ion	6/12756	0.444979816	0.999994594	0.94442346	Soltu.DM.04G003430	1
10	GO:01	cell-cell signaling by wnt	6/1	0.4	0.9	0.9	Soltu.DM.07G002580	1

86	98738		2756	44979816	99994594	4442346		
1087	GO:1900036	positive regulation of cellular response to heat	6/12756	0.444979816	0.999994594	0.94442346	Soltu.DM.07G019630	1
1088	GO:1902022	L-lysine transport	6/12756	0.444979816	0.999994594	0.94442346	Soltu.DM.05G003990	1
1089	GO:1902412	regulation of mitotic cytokinesis	6/12756	0.444979816	0.999994594	0.94442346	Soltu.DM.12G023230	1
1090	GO:1903317	regulation of protein maturation	6/12756	0.444979816	0.999994594	0.94442346	Soltu.DM.06G018040	1
1091	GO:1903401	L-lysine transmembrane transport	6/12756	0.444979816	0.999994594	0.94442346	Soltu.DM.05G003990	1
1092	GO:1903436	regulation of mitotic cytokinetic process	6/12756	0.444979816	0.999994594	0.94442346	Soltu.DM.12G023230	1
1093	GO:1904965	regulation of vitamin E biosynthetic process	6/12756	0.444979816	0.999994594	0.94442346	Soltu.DM.03G008510	1
1094	GO:1904966	positive regulation of vitamin E biosynthetic process	6/12756	0.444979816	0.999994594	0.94442346	Soltu.DM.03G008510	1
1095	GO:1905114	cell surface receptor signaling pathway involved in cell-cell signaling	6/12756	0.444979816	0.999994594	0.94442346	Soltu.DM.07G002580	1
1096	GO:2000067	regulation of root morphogenesis	6/12756	0.444979816	0.999994594	0.94442346	Soltu.DM.04G022240	1
1097	GO:0008283	cell population proliferation	78/12756	0.446364231	0.999994594	0.94442346	Soltu.DM.08G027150/Soltu.DM.06G018130/Soltu.DM.04G006620/Soltu.DM.11G007750/Soltu.DM.02G029740/Soltu.DM.09G002090/Soltu.DM.07G002580/Soltu.DM.03G020440	8
1098	GO:0006839	mitochondrial transport	99/12756	0.447287046	0.999994594	0.94442346	Soltu.DM.04G037150/Soltu.DM.01G038470/Soltu.DM.06G024610/Soltu.DM.03G032350/Soltu.DM.10G028240/Soltu.DM.01G034680/Soltu.DM.06G018560/Soltu.DM.05G021160/Soltu.DM.06G025410/Soltu.DM.12G021450	10
1099	GO:0006636	unsaturated fatty acid biosynthetic process	16/12756	0.448747395	0.999994594	0.94442346	Soltu.DM.07G026100/Soltu.DM.02G019940	2
1100	GO:0006787	porphyrin-containing compound catabolic process	16/12756	0.448747395	0.999994594	0.94442346	Soltu.DM.07G010570/Soltu.DM.12G026560	2
1101	GO:0009218	pyrimidine ribonucleotide metabolic process	16/12756	0.448747395	0.999994594	0.94442346	Soltu.DM.11G007630/Soltu.DM.01G017170	2
1102	GO:0009220	pyrimidine ribonucleotide biosynthetic process	16/12756	0.448747395	0.999994594	0.94442346	Soltu.DM.11G007630/Soltu.DM.01G017170	2
1103	GO:0010412	mannan metabolic process	16/12756	0.448747395	0.999994594	0.94442346	Soltu.DM.11G000740/Soltu.DM.06G021870	2

1104	GO:0016074	sno(s)RNA metabolic process	16/12756	0.448747395	0.999994594	0.94442346	Soltu.DM.02G021990/Soltu.DM.06G029830	2
1105	GO:0018202	peptidyl-histidine modification	16/12756	0.448747395	0.999994594	0.94442346	Soltu.DM.05G011970/Soltu.DM.07G022640	2
1106	GO:0019319	hexose biosynthetic process	16/12756	0.448747395	0.999994594	0.94442346	Soltu.DM.09G018910/Soltu.DM.04G031580	2
1107	GO:0019320	hexose catabolic process	16/12756	0.448747395	0.999994594	0.94442346	Soltu.DM.02G031920/Soltu.DM.02G031890	2
1108	GO:0019321	pentose metabolic process	16/12756	0.448747395	0.999994594	0.94442346	Soltu.DM.09G023470/Soltu.DM.02G012570	2
1109	GO:0032206	positive regulation of telomere maintenance	16/12756	0.448747395	0.999994594	0.94442346	Soltu.DM.01G002690/Soltu.DM.02G013390	2
1110	GO:0032481	positive regulation of type I interferon production	16/12756	0.448747395	0.999994594	0.94442346	Soltu.DM.08G020460/Soltu.DM.01G002690	2
1111	GO:0033015	tetrapyrrole catabolic process	16/12756	0.448747395	0.999994594	0.94442346	Soltu.DM.07G010570/Soltu.DM.12G026560	2
1112	GO:0045851	pH reduction	16/12756	0.448747395	0.999994594	0.94442346	Soltu.DM.02G024200/Soltu.DM.07G009580	2
1113	GO:0070252	actin-mediated cell contraction	16/12756	0.448747395	0.999994594	0.94442346	Soltu.DM.09G018720/Soltu.DM.01G028770	2
1114	GO:0072331	signal transduction by p53 class mediator	16/12756	0.448747395	0.999994594	0.94442346	Soltu.DM.12G025260/Soltu.DM.11G016820	2
1115	GO:0090691	formation of plant organ boundary	16/12756	0.448747395	0.999994594	0.94442346	Soltu.DM.08G013580/Soltu.DM.02G016680	2
1116	GO:1903312	negative regulation of mRNA metabolic process	16/12756	0.448747395	0.999994594	0.94442346	Soltu.DM.11G016820/Soltu.DM.10G001400	2
1117	GO:0010431	seed maturation	47/12756	0.45102328	0.999994594	0.94442346	Soltu.DM.07G013360/Soltu.DM.08G011330/Soltu.DM.01G001590/Soltu.DM.03G013100/Soltu.DM.07G024500	5
1118	GO:0015850	organic hydroxy compound transport	47/12756	0.45102328	0.999994594	0.94442346	Soltu.DM.03G035710/Soltu.DM.09G028710/Soltu.DM.06G005370/Soltu.DM.03G031200/Soltu.DM.11G011430	5
1119	GO:0033157	regulation of intracellular protein transport	47/12756	0.45102328	0.999994594	0.94442346	Soltu.DM.12G020370/Soltu.DM.12G020350/Soltu.DM.07G002400/Soltu.DM.08G001690/Soltu.DM.12G020340	5
1120	GO:0055082	cellular chemical homeostasis	173/12756	0.451322594	0.999994594	0.94442346	Soltu.DM.02G034460/Soltu.DM.09G002620/Soltu.DM.07G002440/Soltu.DM.03G017590/Soltu.DM.08G029860/Soltu.DM.08G008380/Soltu.DM.02G024200/Soltu.DM.05G021830/Soltu.DM.04G003430/Soltu.DM.11G000570/Soltu.DM.11G011180/Soltu.DM.07G009580/Soltu.DM.05G027000/Soltu.DM.10G000600/Soltu.DM.10G004300/Soltu.DM.10G025890/Soltu.DM.08G001690	17
1121	GO:0009110	vitamin biosynthetic process	68/12	0.4532	0.9999	0.9444	Soltu.DM.04G025250/Soltu.DM.09G006800/Soltu.DM.02G025970/Soltu.DM.06G009750/Soltu.DM.01G045760/Soltu.DM.06G003240/Soltu.DM.01G044760	7

			756	47139	94594	42346		
1122	GO:0071368	cellular response to cytokinin stimulus	68/12756	0.453247139	0.999994594	0.94442346	Soltu.DM.03G027640/Soltu.DM.10G027680/Soltu.DM.05G024870/Soltu.DM.05G011970/Soltu.DM.06G011930/Soltu.DM.10G027810/Soltu.DM.06G011620	7
1123	GO:0043549	regulation of kinase activity	110/12756	0.453587633	0.999994594	0.94442346	Soltu.DM.07G017210/Soltu.DM.07G017190/Soltu.DM.07G017200/Soltu.DM.07G017180/Soltu.DM.07G012130/Soltu.DM.06G018320/Soltu.DM.11G010230/Soltu.DM.02G028740/Soltu.DM.11G010220/Soltu.DM.06G028580/Soltu.DM.04G038280	11
1124	GO:0000018	regulation of DNA recombination	37/12756	0.459121823	0.999994594	0.94442346	Soltu.DM.04G034330/Soltu.DM.02G001620/Soltu.DM.02G001630/Soltu.DM.05G006310	4
1125	GO:0000723	telomere maintenance	37/12756	0.459121823	0.999994594	0.94442346	Soltu.DM.08G027160/Soltu.DM.11G017980/Soltu.DM.05G023970/Soltu.DM.02G013390	4
1126	GO:0098739	import across plasma membrane	37/12756	0.459121823	0.999994594	0.94442346	Soltu.DM.04G031760/Soltu.DM.12G006380/Soltu.DM.12G024710/Soltu.DM.02G032050	4
1127	GO:0042430	indole-containing compound metabolic process	79/12756	0.460909694	0.999994594	0.94442346	Soltu.DM.03G035710/Soltu.DM.01G038470/Soltu.DM.03G035070/Soltu.DM.12G022190/Soltu.DM.06G018840/Soltu.DM.12G026120/Soltu.DM.03G035080/Soltu.DM.07G014750	8
1128	GO:0009201	ribonucleoside triphosphate biosynthetic process	58/12756	0.460946403	0.999994594	0.94442346	Soltu.DM.12G004480/Soltu.DM.07G009580/Soltu.DM.10G027910/Soltu.DM.06G013720/Soltu.DM.01G017170/Soltu.DM.11G025570	6
1129	GO:0110053	regulation of actin filament organization	58/12756	0.460946403	0.999994594	0.94442346	Soltu.DM.06G022970/Soltu.DM.01G028770/Soltu.DM.09G015150/Soltu.DM.12G009990/Soltu.DM.10G004310/Soltu.DM.09G027230	6
1130	GO:0009267	cellular response to starvation	174/12756	0.461112635	0.999994594	0.94442346	Soltu.DM.02G017810/Soltu.DM.04G025250/Soltu.DM.01G049900/Soltu.DM.01G049890/Soltu.DM.07G028550/Soltu.DM.08G030020/Soltu.DM.04G001370/Soltu.DM.10G000640/Soltu.DM.03G000340/Soltu.DM.07G020410/Soltu.DM.04G031580/Soltu.DM.11G007630/Soltu.DM.01G028770/Soltu.DM.05G011970/Soltu.DM.11G022310/Soltu.DM.01G040220/Soltu.DM.10G002820	17
1131	GO:0007178	transmembrane receptor protein serine/threonine kinase signaling pathway	153/12756	0.462669722	0.999994594	0.94442346	Soltu.DM.04G024310/Soltu.DM.08G023130/Soltu.DM.12G001970/Soltu.DM.01G002930/Soltu.DM.02G026780/Soltu.DM.02G020600/Soltu.DM.09G026810/Soltu.DM.03G031770/Soltu.DM.01G031880/Soltu.DM.02G019190/Soltu.DM.09G029750/Soltu.DM.03G001740/Soltu.DM.03G037530/Soltu.DM.07G020980/Soltu.DM.01G051120	15
1132	GO:0008652	amino acid biosynthetic process	153/12756	0.462669722	0.999994594	0.94442346	Soltu.DM.04G023360/Soltu.DM.04G018630/Soltu.DM.02G031330/Soltu.DM.03G005810/Soltu.DM.08G030020/Soltu.DM.06G018090/Soltu.DM.07G023080/Soltu.DM.09G006670/Soltu.DM.02G020220/Soltu.DM.08G011890/Soltu.DM.12G025770/Soltu.DM.11G003850/Soltu.DM.07G006550/Soltu.DM.08G007450/Soltu.DM.07G014750	15
1133	GO:0051493	regulation of cytoskeleton organization	90/12756	0.467414685	0.999994594	0.94442346	Soltu.DM.03G003730/Soltu.DM.06G022970/Soltu.DM.01G028770/Soltu.DM.09G015150/Soltu.DM.04G022240/Soltu.DM.12G009990/Soltu.DM.10G004310/Soltu.DM.09G027230/Soltu.DM.12G023230	9
1134	GO:0006206	pyrimidine nucleobase metabolic process	27/12756	0.468731453	0.999994594	0.94442346	Soltu.DM.10G012990/Soltu.DM.03G019550/Soltu.DM.01G017170	3
1135	GO:0006534	cysteine metabolic process	27/12756	0.468731453	0.999994594	0.94442346	Soltu.DM.08G030020/Soltu.DM.12G002620/Soltu.DM.12G025770	3
1136	GO:0015695	organic cation transport	27/12756	0.468731453	0.999994594	0.94442346	Soltu.DM.12G010360/Soltu.DM.06G018020/Soltu.DM.08G003390	3
1137	GO:0031396	regulation of protein ubiquitination	27/12756	0.468731453	0.999994594	0.94442346	Soltu.DM.02G018520/Soltu.DM.06G015770/Soltu.DM.09G005140	3
1138	GO:0043066	negative regulation of apoptotic process	27/12756	0.468731453	0.999994594	0.94442346	Soltu.DM.02G018520/Soltu.DM.04G022240/Soltu.DM.06G024530	3

1139	GO:1905268	negative regulation of chromatin organization	27/12756	0.468731453	0.999994594	0.94442346	Soltu.DM.08G022190/Soltu.DM.04G038280/Soltu.DM.10G024770	3
1140	GO:2000012	regulation of auxin polar transport	27/12756	0.468731453	0.999994594	0.94442346	Soltu.DM.07G022680/Soltu.DM.08G013590/Soltu.DM.11G024760	3
1141	GO:0042176	regulation of protein catabolic process	69/12756	0.468834388	0.999994594	0.94442346	Soltu.DM.08G027150/Soltu.DM.12G005510/Soltu.DM.06G015770/Soltu.DM.04G034740/Soltu.DM.09G005140/Soltu.DM.09G002090/Soltu.DM.06G024530	7
1142	GO:0060548	negative regulation of cell death	69/12756	0.468834388	0.999994594	0.94442346	Soltu.DM.02G018520/Soltu.DM.05G022160/Soltu.DM.08G022900/Soltu.DM.06G028580/Soltu.DM.08G028440/Soltu.DM.04G022240/Soltu.DM.06G024530	7
1143	GO:0009116	nucleoside metabolic process	48/12756	0.469768541	0.999994594	0.94442346	Soltu.DM.03G037170/Soltu.DM.10G027910/Soltu.DM.12G003790/Soltu.DM.12G002620/Soltu.DM.01G017170	5
1144	GO:0010286	heat acclimation	48/12756	0.469768541	0.999994594	0.94442346	Soltu.DM.07G011880/Soltu.DM.06G018840/Soltu.DM.08G012010/Soltu.DM.05G022450/Soltu.DM.12G021450	5
1145	GO:0035195	miRNA-mediated gene silencing	48/12756	0.469768541	0.999994594	0.94442346	Soltu.DM.12G005490/Soltu.DM.12G026070/Soltu.DM.11G025410/Soltu.DM.01G010020/Soltu.DM.04G031030	5
1146	GO:0031329	regulation of cellular catabolic process	186/12756	0.474582543	0.999994594	0.94442346	Soltu.DM.05G004270/Soltu.DM.08G014180/Soltu.DM.08G027150/Soltu.DM.12G005510/Soltu.DM.09G014740/Soltu.DM.06G018840/Soltu.DM.08G013620/Soltu.DM.10G022360/Soltu.DM.05G026810/Soltu.DM.06G015770/Soltu.DM.05G011970/Soltu.DM.11G016820/Soltu.DM.04G034740/Soltu.DM.09G005140/Soltu.DM.01G002690/Soltu.DM.09G002090/Soltu.DM.06G024530/Soltu.DM.04G031030	18
1147	GO:0090627	plant epidermal cell differentiation	165/12756	0.476999858	0.999994594	0.94442346	Soltu.DM.08G027650/Soltu.DM.08G029290/Soltu.DM.04G001370/Soltu.DM.12G008450/Soltu.DM.12G004060/Soltu.DM.12G005490/Soltu.DM.08G023170/Soltu.DM.12G024710/Soltu.DM.05G021390/Soltu.DM.09G026810/Soltu.DM.09G031790/Soltu.DM.03G001740/Soltu.DM.01G042120/Soltu.DM.01G047090/Soltu.DM.03G018740/Soltu.DM.04G002690	16
1148	GO:0048764	trichoblast maturation	112/12756	0.478069738	0.999994594	0.94442346	Soltu.DM.08G027650/Soltu.DM.04G001370/Soltu.DM.12G004060/Soltu.DM.12G005490/Soltu.DM.08G023170/Soltu.DM.12G024710/Soltu.DM.09G026810/Soltu.DM.03G001740/Soltu.DM.01G042120/Soltu.DM.03G018740/Soltu.DM.04G002690	11
1149	GO:0048765	root hair cell differentiation	112/12756	0.478069738	0.999994594	0.94442346	Soltu.DM.08G027650/Soltu.DM.04G001370/Soltu.DM.12G004060/Soltu.DM.12G005490/Soltu.DM.08G023170/Soltu.DM.12G024710/Soltu.DM.09G026810/Soltu.DM.03G001740/Soltu.DM.01G042120/Soltu.DM.03G018740/Soltu.DM.04G002690	11
1150	GO:0002832	negative regulation of response to biotic stimulus	38/12756	0.480243586	0.999994594	0.94442346	Soltu.DM.08G022900/Soltu.DM.06G015770/Soltu.DM.02G023840/Soltu.DM.05G023030	4
1151	GO:0010506	regulation of autophagy	38/12756	0.480243586	0.999994594	0.94442346	Soltu.DM.08G014180/Soltu.DM.09G014740/Soltu.DM.06G018840/Soltu.DM.10G022360	4
1152	GO:0032200	telomere organization	38/12756	0.480243586	0.999994594	0.94442346	Soltu.DM.08G027160/Soltu.DM.11G017980/Soltu.DM.05G023970/Soltu.DM.02G013390	4
1153	GO:0048586	regulation of long-day photoperiodism, flowering	38/12756	0.480243586	0.999994594	0.94442346	Soltu.DM.12G007510/Soltu.DM.04G027760/Soltu.DM.04G006870/Soltu.DM.01G024340	4
1154	GO:0090421	embryonic meristem initiation	38/12756	0.480243586	0.999994594	0.94442346	Soltu.DM.02G003130/Soltu.DM.05G026810/Soltu.DM.06G034230/Soltu.DM.03G034800	4
1155	GO:0120031	plasma membrane bounded cell projection assembly	38/12756	0.480243586	0.999994594	0.94442346	Soltu.DM.01G028770/Soltu.DM.09G019870/Soltu.DM.01G020880/Soltu.DM.07G024370	4
1156	GO:0001578	microtubule bundle formation	17/12	0.4808	0.9999	0.9444	Soltu.DM.02G030630/Soltu.DM.01G020880	2

			756	44795	94594	2346		
1157	GO:0001819	positive regulation of cytokine production	17/12756	0.480844795	0.999994594	0.94442346	Soltu.DM.08G020460/Soltu.DM.01G002690	2
1158	GO:0006560	proline metabolic process	17/12756	0.480844795	0.999994594	0.94442346	Soltu.DM.08G007450/Soltu.DM.07G014750	2
1159	GO:0008015	blood circulation	17/12756	0.480844795	0.999994594	0.94442346	Soltu.DM.02G017970/Soltu.DM.10G004300	2
1160	GO:0010216	maintenance of DNA methylation	17/12756	0.480844795	0.999994594	0.94442346	Soltu.DM.04G006870/Soltu.DM.11G004150	2
1161	GO:0015985	energy coupled proton transport, down electrochemical gradient	17/12756	0.480844795	0.999994594	0.94442346	Soltu.DM.07G009580/Soltu.DM.11G025570	2
1162	GO:0015986	proton motive force-driven ATP synthesis	17/12756	0.480844795	0.999994594	0.94442346	Soltu.DM.07G009580/Soltu.DM.11G025570	2
1163	GO:0030705	cytoskeleton-dependent intracellular transport	17/12756	0.480844795	0.999994594	0.94442346	Soltu.DM.07G024370/Soltu.DM.10G001460	2
1164	GO:0045824	negative regulation of innate immune response	17/12756	0.480844795	0.999994594	0.94442346	Soltu.DM.08G022900/Soltu.DM.05G023030	2
1165	GO:0045910	negative regulation of DNA recombination	17/12756	0.480844795	0.999994594	0.94442346	Soltu.DM.04G034330/Soltu.DM.05G006310	2
1166	GO:0046365	monosaccharide catabolic process	17/12756	0.480844795	0.999994594	0.94442346	Soltu.DM.02G031920/Soltu.DM.02G031890	2
1167	GO:0048571	long-day photoperiodism	17/12756	0.480844795	0.999994594	0.94442346	Soltu.DM.01G041980/Soltu.DM.01G024340	2
1168	GO:0048587	regulation of short-day photoperiodism, flowering	17/12756	0.480844795	0.999994594	0.94442346	Soltu.DM.05G012040/Soltu.DM.01G024940	2
1169	GO:0051047	positive regulation of secretion	17/12756	0.480844795	0.999994594	0.94442346	Soltu.DM.01G002690/Soltu.DM.02G022620	2
1170	GO:0051291	protein heterooligomerization	17/12756	0.480844795	0.999994594	0.94442346	Soltu.DM.03G012810/Soltu.DM.02G032340	2
1171	GO:0071712	ER-associated misfolded protein catabolic process	17/12756	0.480844795	0.999994594	0.94442346	Soltu.DM.10G004220/Soltu.DM.06G009790	2
1172	GO:1903532	positive regulation of secretion by cell	17/12756	0.480844795	0.999994594	0.94442346	Soltu.DM.01G002690/Soltu.DM.02G022620	2
1173	GO:0009648	photoperiodism	91/12756	0.480976055	0.999994594	0.94442346	Soltu.DM.02G025590/Soltu.DM.11G001010/Soltu.DM.06G012790/Soltu.DM.04G006870/Soltu.DM.01G041980/Soltu.DM.02G002480/Soltu.DM.12G026560/Soltu.DM.05G012040/Soltu.DM.01G024340	9
11	GO:00	aspartate family amino acid metabolic	49/	0.4	0.9	0.9	Soltu.DM.03G035070/Soltu.DM.08G030020/Soltu.DM.03G035080/Soltu.DM.12G002620/S	5

74	09066	process	12756	88307828	99994594	4442346	oltu.DM.07G006550	
1175	GO:0045732	positive regulation of protein catabolic process	49/12756	0.488307828	0.999994594	0.944423346	Soltu.DM.12G005510/Soltu.DM.06G015770/Soltu.DM.04G034740/Soltu.DM.09G005140/Soltu.DM.06G024530	5
1176	GO:0042538	hyperosmotic salinity response	81/12756	0.4897541	0.999994594	0.944423346	Soltu.DM.03G017570/Soltu.DM.08G019590/Soltu.DM.01G046560/Soltu.DM.05G018830/Soltu.DM.05G009320/Soltu.DM.08G007450/Soltu.DM.05G018810/Soltu.DM.07G014750	8
1177	GO:0010054	trichoblast differentiation	113/12756	0.490233256	0.999994594	0.944423346	Soltu.DM.08G027650/Soltu.DM.04G001370/Soltu.DM.12G004060/Soltu.DM.12G005490/Soltu.DM.08G023170/Soltu.DM.12G024710/Soltu.DM.09G026810/Soltu.DM.03G001740/Soltu.DM.01G042120/Soltu.DM.03G018740/Soltu.DM.04G002690	11
1178	GO:0010267	ta-siRNA processing	28/12756	0.49341127	0.999994594	0.944423346	Soltu.DM.01G010020/Soltu.DM.04G031030/Soltu.DM.11G004150	3
1179	GO:0016109	tetraterpenoid biosynthetic process	28/12756	0.49341127	0.999994594	0.944423346	Soltu.DM.08G028310/Soltu.DM.06G029640/Soltu.DM.12G026560	3
1180	GO:0016117	carotenoid biosynthetic process	28/12756	0.49341127	0.999994594	0.944423346	Soltu.DM.08G028310/Soltu.DM.06G029640/Soltu.DM.12G026560	3
1181	GO:0042133	neurotransmitter metabolic process	28/12756	0.49341127	0.999994594	0.944423346	Soltu.DM.08G013400/Soltu.DM.12G024030/Soltu.DM.06G034310	3
1182	GO:0050821	protein stabilization	28/12756	0.49341127	0.999994594	0.944423346	Soltu.DM.03G030480/Soltu.DM.04G037380/Soltu.DM.04G037150	3
1183	GO:0000967	rRNA 5'-end processing	7/12756	0.496868762	0.999994594	0.944423346	Soltu.DM.01G051600	1
1184	GO:0002230	positive regulation of defense response to virus by host	7/12756	0.496868762	0.999994594	0.944423346	Soltu.DM.03G021360	1
1185	GO:0006063	uronic acid metabolic process	7/12756	0.496868762	0.999994594	0.944423346	Soltu.DM.12G002640	1
1186	GO:0006102	isocitrate metabolic process	7/12756	0.496868762	0.999994594	0.944423346	Soltu.DM.07G017750	1
1187	GO:0006241	CTP biosynthetic process	7/12756	0.496868762	0.999994594	0.944423346	Soltu.DM.01G017170	1
1188	GO:0006265	DNA topological change	7/12756	0.496868762	0.999994594	0.944423346	Soltu.DM.12G016140	1
1189	GO:0006283	transcription-coupled nucleotide-excision repair	7/12756	0.496868762	0.999994594	0.944423346	Soltu.DM.08G027160	1
1190	GO:0006692	prostanoid metabolic process	7/12756	0.496868762	0.999994594	0.944423346	Soltu.DM.02G019940	1
1191	GO:0006693	prostaglandin metabolic process	7/12756	0.496868762	0.999994594	0.944423346	Soltu.DM.02G019940	1

1192	GO:0006772	thiamine metabolic process	7/12756	0.496868762	0.999994594	0.94442346	Soltu.DM.06G003240	1
1193	GO:0006937	regulation of muscle contraction	7/12756	0.496868762	0.999994594	0.94442346	Soltu.DM.02G018520	1
1194	GO:0009071	serine family amino acid catabolic process	7/12756	0.496868762	0.999994594	0.94442346	Soltu.DM.08G013400	1
1195	GO:0009085	lysine biosynthetic process	7/12756	0.496868762	0.999994594	0.94442346	Soltu.DM.07G006550	1
1196	GO:0009208	pyrimidine ribonucleoside triphosphate metabolic process	7/12756	0.496868762	0.999994594	0.94442346	Soltu.DM.01G017170	1
1197	GO:0009209	pyrimidine ribonucleoside triphosphate biosynthetic process	7/12756	0.496868762	0.999994594	0.94442346	Soltu.DM.01G017170	1
1198	GO:0009609	response to symbiotic bacterium	7/12756	0.496868762	0.999994594	0.94442346	Soltu.DM.03G017570	1
1199	GO:0009720	detection of hormone stimulus	7/12756	0.496868762	0.999994594	0.94442346	Soltu.DM.07G022640	1
1200	GO:0009726	detection of endogenous stimulus	7/12756	0.496868762	0.999994594	0.94442346	Soltu.DM.07G022640	1
1201	GO:0010023	proanthocyanidin biosynthetic process	7/12756	0.496868762	0.999994594	0.94442346	Soltu.DM.08G026700	1
1202	GO:0010375	stomatal complex patterning	7/12756	0.496868762	0.999994594	0.94442346	Soltu.DM.12G010960	1
1203	GO:0014074	response to purine-containing compound	7/12756	0.496868762	0.999994594	0.94442346	Soltu.DM.01G028770	1
1204	GO:0015793	glycerol transmembrane transport	7/12756	0.496868762	0.999994594	0.94442346	Soltu.DM.03G031200	1
1205	GO:0015977	carbon fixation	7/12756	0.496868762	0.999994594	0.94442346	Soltu.DM.01G024860	1
1206	GO:0019586	galacturonate metabolic process	7/12756	0.496868762	0.999994594	0.94442346	Soltu.DM.12G002640	1
1207	GO:0030174	regulation of DNA-templated DNA replication initiation	7/12756	0.496868762	0.999994594	0.94442346	Soltu.DM.03G032560	1
1208	GO:0031034	myosin filament assembly	7/12756	0.496868762	0.999994594	0.94442346	Soltu.DM.01G028770	1
1209	GO:0031408	oxylipin biosynthetic process	7/12756	0.4968687	0.9999945	0.9444234	Soltu.DM.07G003270	1

				62	94	6		
1210	GO:0031571	mitotic G1 DNA damage checkpoint signaling	7/12756	0.496868762	0.999994594	0.94442346	Soltu.DM.11G016820	1
1211	GO:0032467	positive regulation of cytokinesis	7/12756	0.496868762	0.999994594	0.94442346	Soltu.DM.12G023230	1
1212	GO:0032648	regulation of interferon-beta production	7/12756	0.496868762	0.999994594	0.94442346	Soltu.DM.08G020460	1
1213	GO:0032728	positive regulation of interferon-beta production	7/12756	0.496868762	0.999994594	0.94442346	Soltu.DM.08G020460	1
1214	GO:0034051	negative regulation of plant-type hypersensitive response	7/12756	0.496868762	0.999994594	0.94442346	Soltu.DM.08G022900	1
1215	GO:0043112	receptor metabolic process	7/12756	0.496868762	0.999994594	0.94442346	Soltu.DM.07G002580	1
1216	GO:0043968	histone H2A acetylation	7/12756	0.496868762	0.999994594	0.94442346	Soltu.DM.04G033160	1
1217	GO:0043982	histone H4-K8 acetylation	7/12756	0.496868762	0.999994594	0.94442346	Soltu.DM.09G019870	1
1218	GO:0043984	histone H4-K16 acetylation	7/12756	0.496868762	0.999994594	0.94442346	Soltu.DM.09G019870	1
1219	GO:0044819	mitotic G1/S transition checkpoint signaling	7/12756	0.496868762	0.999994594	0.94442346	Soltu.DM.11G016820	1
1220	GO:0046036	CTP metabolic process	7/12756	0.496868762	0.999994594	0.94442346	Soltu.DM.01G017170	1
1221	GO:0046337	phosphatidylethanolamine metabolic process	7/12756	0.496868762	0.999994594	0.94442346	Soltu.DM.02G019940	1
1222	GO:0046459	short-chain fatty acid metabolic process	7/12756	0.496868762	0.999994594	0.94442346	Soltu.DM.02G020870	1
1223	GO:0046473	phosphatidic acid metabolic process	7/12756	0.496868762	0.999994594	0.94442346	Soltu.DM.04G037130	1
1224	GO:0046660	female sex differentiation	7/12756	0.496868762	0.999994594	0.94442346	Soltu.DM.12G019270	1
1225	GO:0046931	pore complex assembly	7/12756	0.496868762	0.999994594	0.94442346	Soltu.DM.04G011330	1
1226	GO:0051016	barbed-end actin filament capping	7/12756	0.496868762	0.999994594	0.94442346	Soltu.DM.09G015150	1
1227	GO:0051292	nuclear pore complex assembly	7/127	0.4968	0.9999	0.9444	Soltu.DM.04G011330	1

			56	687 62	945 94	234 6		
1228	GO:0051591	response to cAMP	7/127 56	0.4968 68762	0.9999 94594	0.9444 2346	Soltu.DM.01G028770	1
1229	GO:0060359	response to ammonium ion	7/127 56	0.4968 68762	0.9999 94594	0.9444 2346	Soltu.DM.06G009750	1
1230	GO:0071461	cellular response to redox state	7/127 56	0.4968 68762	0.9999 94594	0.9444 2346	Soltu.DM.06G028040	1
1231	GO:0080029	cellular response to boron-containing substance levels	7/127 56	0.4968 68762	0.9999 94594	0.9444 2346	Soltu.DM.03G031200	1
1232	GO:0090069	regulation of ribosome biogenesis	7/127 56	0.4968 68762	0.9999 94594	0.9444 2346	Soltu.DM.11G024760	1
1233	GO:0090175	regulation of establishment of planar polarity	7/127 56	0.4968 68762	0.9999 94594	0.9444 2346	Soltu.DM.07G002580	1
1234	GO:0110020	regulation of actomyosin structure organization	7/127 56	0.4968 68762	0.9999 94594	0.9444 2346	Soltu.DM.12G023230	1
1235	GO:0140027	contractile vacuole localization	7/127 56	0.4968 68762	0.9999 94594	0.9444 2346	Soltu.DM.09G018720	1
1236	GO:1902807	negative regulation of cell cycle G1/S phase transition	7/127 56	0.4968 68762	0.9999 94594	0.9444 2346	Soltu.DM.11G016820	1
1237	GO:1903426	regulation of reactive oxygen species biosynthetic process	7/127 56	0.4968 68762	0.9999 94594	0.9444 2346	Soltu.DM.08G013620	1
1238	GO:1905897	regulation of response to endoplasmic reticulum stress	7/127 56	0.4968 68762	0.9999 94594	0.9444 2346	Soltu.DM.03G021360	1
1239	GO:1990849	vacuolar localization	7/127 56	0.4968 68762	0.9999 94594	0.9444 2346	Soltu.DM.09G018720	1
1240	GO:2000050	regulation of non-canonical Wnt signaling pathway	7/127 56	0.4968 68762	0.9999 94594	0.9444 2346	Soltu.DM.07G002580	1
1241	GO:2000134	negative regulation of G1/S transition of mitotic cell cycle	7/127 56	0.4968 68762	0.9999 94594	0.9444 2346	Soltu.DM.11G016820	1
1242	GO:2000306	positive regulation of photomorphogenesis	7/127 56	0.4968 68762	0.9999 94594	0.9444 2346	Soltu.DM.01G024340	1
1243	GO:0006766	vitamin metabolic process	71/12756	0.4996 58515	0.9999 94594	0.9444 2346	Soltu.DM.04G025250/Soltu.DM.09G006800/Soltu.DM.02G025970/Soltu.DM.06G009750/Soltu.DM.01G045760/Soltu.DM.06G003240/Soltu.DM.01G044760	7
1244	GO:0010083	regulation of vegetative meristem growth	71/12756	0.4996 58515	0.9999 94594	0.9444 2346	Soltu.DM.04G011110/Soltu.DM.10G027470/Soltu.DM.04G011320/Soltu.DM.04G011240/Soltu.DM.10G028070/Soltu.DM.04G011370/Soltu.DM.11G021090	7
12	GO:19	regulation of developmental vegetative	71/	0.4	0.9	0.9	Soltu.DM.04G011110/Soltu.DM.10G027470/Soltu.DM.04G011320/Soltu.DM.04G011240/S	7

45	05613	growth	12756	99658515	99994594	4442346	oltu.DM.10G028070/Soltu.DM.04G011370/Soltu.DM.11G021090	
1246	GO:2000243	positive regulation of reproductive process	71/12756	0.499658515	0.999994594	0.94442346	Soltu.DM.12G007510/Soltu.DM.04G027760/Soltu.DM.09G022610/Soltu.DM.06G019760/Soltu.DM.01G040220/Soltu.DM.08G001470/Soltu.DM.03G024000	7
1247	GO:0048439	flower morphogenesis	82/12756	0.504019406	0.999994594	0.94442346	Soltu.DM.08G022190/Soltu.DM.04G011110/Soltu.DM.10G027470/Soltu.DM.04G011320/Soltu.DM.04G011240/Soltu.DM.10G028070/Soltu.DM.04G011370/Soltu.DM.11G021090	8
1248	GO:1901136	carbohydrate derivative catabolic process	50/12756	0.506609301	0.999994594	0.94442346	Soltu.DM.03G035710/Soltu.DM.01G042210/Soltu.DM.12G003790/Soltu.DM.02G008560/Soltu.DM.02G008550	5
1249	GO:0006568	tryptophan metabolic process	18/12756	0.511762542	0.999994594	0.94442346	Soltu.DM.03G035070/Soltu.DM.03G035080	2
1250	GO:0006586	indolalkylamine metabolic process	18/12756	0.511762542	0.999994594	0.94442346	Soltu.DM.03G035070/Soltu.DM.03G035080	2
1251	GO:0019344	cysteine biosynthetic process	18/12756	0.511762542	0.999994594	0.94442346	Soltu.DM.08G030020/Soltu.DM.12G025770	2
1252	GO:0032479	regulation of type I interferon production	18/12756	0.511762542	0.999994594	0.94442346	Soltu.DM.08G020460/Soltu.DM.01G002690	2
1253	GO:0032781	positive regulation of ATP-dependent activity	18/12756	0.511762542	0.999994594	0.94442346	Soltu.DM.05G006580/Soltu.DM.07G024370	2
1254	GO:0043462	regulation of ATP-dependent activity	18/12756	0.511762542	0.999994594	0.94442346	Soltu.DM.05G006580/Soltu.DM.07G024370	2
1255	GO:0046189	phenol-containing compound biosynthetic process	18/12756	0.511762542	0.999994594	0.94442346	Soltu.DM.08G026700/Soltu.DM.09G023400	2
1256	GO:0070507	regulation of microtubule cytoskeleton organization	18/12756	0.511762542	0.999994594	0.94442346	Soltu.DM.03G003730/Soltu.DM.04G022240	2
1257	GO:0080113	regulation of seed growth	18/12756	0.511762542	0.999994594	0.94442346	Soltu.DM.05G024870/Soltu.DM.03G036780	2
1258	GO:1900425	negative regulation of defense response to bacterium	18/12756	0.511762542	0.999994594	0.94442346	Soltu.DM.06G015770/Soltu.DM.02G023840	2
1259	GO:1902584	positive regulation of response to water deprivation	18/12756	0.511762542	0.999994594	0.94442346	Soltu.DM.06G017300/Soltu.DM.09G023300	2
1260	GO:1904356	regulation of telomere maintenance via telomere lengthening	18/12756	0.511762542	0.999994594	0.94442346	Soltu.DM.05G006310/Soltu.DM.02G013390	2
1261	GO:1905421	regulation of plant organ morphogenesis	18/12756	0.511762542	0.999994594	0.94442346	Soltu.DM.04G022240/Soltu.DM.08G012010	2
1262	GO:0002252	immune effector process	115/12756	0.514353512	0.999994594	0.94442346	Soltu.DM.02G025590/Soltu.DM.10G025390/Soltu.DM.07G028550/Soltu.DM.10G000640/Soltu.DM.02G012280/Soltu.DM.01G003040/Soltu.DM.10G022360/Soltu.DM.08G028440/Soltu.DM.02G022460/Soltu.DM.01G010020/Soltu.DM.11G004150	11

1263	GO:0031331	positive regulation of cellular catabolic process	115/12756	0.514353512	0.999994594	0.94442346	Soltu.DM.05G004270/Soltu.DM.08G014180/Soltu.DM.12G005510/Soltu.DM.06G018840/Soltu.DM.08G013620/Soltu.DM.05G026810/Soltu.DM.06G015770/Soltu.DM.04G034740/Soltu.DM.09G005140/Soltu.DM.06G024530/Soltu.DM.04G031030	11
1264	GO:0032956	regulation of actin cytoskeleton organization	72/12756	0.514856071	0.999994594	0.94442346	Soltu.DM.06G022970/Soltu.DM.01G028770/Soltu.DM.09G015150/Soltu.DM.12G009990/Soltu.DM.10G004310/Soltu.DM.09G027230/Soltu.DM.12G023230	7
1265	GO:0046165	alcohol biosynthetic process	72/12756	0.514856071	0.999994594	0.94442346	Soltu.DM.02G018520/Soltu.DM.11G024450/Soltu.DM.08G014180/Soltu.DM.06G029640/Soltu.DM.03G008510/Soltu.DM.06G003240/Soltu.DM.10G022710	7
1266	GO:0009827	plant-type cell wall modification	29/12756	0.517506698	0.999994594	0.94442346	Soltu.DM.09G007590/Soltu.DM.01G025270/Soltu.DM.01G040720	3
1267	GO:0009943	adaxial/abaxial axis specification	29/12756	0.517506698	0.999994594	0.94442346	Soltu.DM.02G003130/Soltu.DM.07G026690/Soltu.DM.08G012010	3
1268	GO:0060271	cilium assembly	29/12756	0.517506698	0.999994594	0.94442346	Soltu.DM.09G019870/Soltu.DM.01G020880/Soltu.DM.07G024370	3
1269	GO:0051606	detection of stimulus	83/12756	0.518159207	0.999994594	0.94442346	Soltu.DM.10G026220/Soltu.DM.08G027160/Soltu.DM.03G037290/Soltu.DM.01G028770/Soltu.DM.07G015980/Soltu.DM.12G021010/Soltu.DM.02G011380/Soltu.DM.07G022640	8
1270	GO:0055076	transition metal ion homeostasis	83/12756	0.518159207	0.999994594	0.94442346	Soltu.DM.07G002440/Soltu.DM.01G035900/Soltu.DM.03G017590/Soltu.DM.05G021830/Soltu.DM.04G003430/Soltu.DM.07G009580/Soltu.DM.07G015200/Soltu.DM.01G035910	8
1271	GO:0080147	root hair cell development	83/12756	0.518159207	0.999994594	0.94442346	Soltu.DM.08G027650/Soltu.DM.04G001370/Soltu.DM.12G004060/Soltu.DM.12G005490/Soltu.DM.08G023170/Soltu.DM.12G024710/Soltu.DM.09G026810/Soltu.DM.04G002690	8
1272	GO:0032880	regulation of protein localization	94/12756	0.52111601	0.999994594	0.94442346	Soltu.DM.12G020370/Soltu.DM.12G020350/Soltu.DM.08G027150/Soltu.DM.07G002400/Soltu.DM.01G002690/Soltu.DM.08G001690/Soltu.DM.09G002090/Soltu.DM.12G020340/Soltu.DM.02G022620	9
1273	GO:0009251	glucan catabolic process	40/12756	0.521452236	0.999994594	0.94442346	Soltu.DM.09G027770/Soltu.DM.05G006330/Soltu.DM.12G007130/Soltu.DM.04G037250	4
1274	GO:0009556	microsporogenesis	40/12756	0.521452236	0.999994594	0.94442346	Soltu.DM.06G009270/Soltu.DM.05G022790/Soltu.DM.02G011180/Soltu.DM.12G023840	4
1275	GO:0009145	purine nucleoside triphosphate biosynthetic process	51/12756	0.524643803	0.999994594	0.94442346	Soltu.DM.12G004480/Soltu.DM.07G009580/Soltu.DM.10G027910/Soltu.DM.06G013720/Soltu.DM.11G025570	5
1276	GO:0009206	purine ribonucleoside triphosphate biosynthetic process	51/12756	0.524643803	0.999994594	0.94442346	Soltu.DM.12G004480/Soltu.DM.07G009580/Soltu.DM.10G027910/Soltu.DM.06G013720/Soltu.DM.11G025570	5
1277	GO:0002239	response to oomycetes	62/12756	0.527407195	0.999994594	0.94442346	Soltu.DM.06G028410/Soltu.DM.07G003530/Soltu.DM.09G028710/Soltu.DM.07G003550/Soltu.DM.01G044600/Soltu.DM.05G023040	6
1278	GO:0009142	nucleoside triphosphate biosynthetic process	62/12756	0.527407195	0.999994594	0.94442346	Soltu.DM.12G004480/Soltu.DM.07G009580/Soltu.DM.10G027910/Soltu.DM.06G013720/Soltu.DM.01G017170/Soltu.DM.11G025570	6
1279	GO:0072522	purine-containing compound biosynthetic process	106/12756	0.536255444	0.999994594	0.94442346	Soltu.DM.12G004480/Soltu.DM.03G037170/Soltu.DM.07G009580/Soltu.DM.10G027910/Soltu.DM.06G013720/Soltu.DM.03G021730/Soltu.DM.02G031030/Soltu.DM.11G025570/Soltu.DM.11G010590/Soltu.DM.01G019520	10
1280	GO:0090351	seedling development	128/127	0.5399213	0.9999945	0.9444234	Soltu.DM.02G018520/Soltu.DM.01G038470/Soltu.DM.06G012350/Soltu.DM.02G022700/Soltu.DM.07G017750/Soltu.DM.08G011890/Soltu.DM.02G004510/Soltu.DM.08G011330/Soltu.DM.01G035240/Soltu.DM.10G005360/Soltu.DM.12G028510/Soltu.DM.06G002320	12

			56 72 94 6		
12<br>81	GO:00<br>06898	receptor-mediated endocytosis	30/ 0.5 0.9 0.9<br>12 409 999 444<br>75 691 945 234<br>6 48 94 6	Soltu.DM.09G000440/Soltu.DM.10G023680/Soltu.DM.01G042120	3
12<br>82	GO:00<br>06020	inositol metabolic process	19/ 0.5 0.9 0.9<br>12 414 999 444<br>75 407 945 234<br>6 01 94 6	Soltu.DM.12G024030/Soltu.DM.03G008510	2
12<br>83	GO:00<br>06379	mRNA cleavage	19/ 0.5 0.9 0.9<br>12 414 999 444<br>75 407 945 234<br>6 01 94 6	Soltu.DM.01G006380/Soltu.DM.04G031030	2
12<br>84	GO:00<br>06825	copper ion transport	19/ 0.5 0.9 0.9<br>12 414 999 444<br>75 407 945 234<br>6 01 94 6	Soltu.DM.03G020090/Soltu.DM.08G012080	2
12<br>85	GO:00<br>06904	vesicle docking involved in exocytosis	19/ 0.5 0.9 0.9<br>12 414 999 444<br>75 407 945 234<br>6 01 94 6	Soltu.DM.06G005370/Soltu.DM.11G026460	2
12<br>86	GO:00<br>06995	cellular response to nitrogen starvation	19/ 0.5 0.9 0.9<br>12 414 999 444<br>75 407 945 234<br>6 01 94 6	Soltu.DM.02G017810/Soltu.DM.11G022310	2
12<br>87	GO:00<br>09692	ethylene metabolic process	19/ 0.5 0.9 0.9<br>12 414 999 444<br>75 407 945 234<br>6 01 94 6	Soltu.DM.11G009500/Soltu.DM.02G004480	2
12<br>88	GO:00<br>09693	ethylene biosynthetic process	19/ 0.5 0.9 0.9<br>12 414 999 444<br>75 407 945 234<br>6 01 94 6	Soltu.DM.11G009500/Soltu.DM.02G004480	2
12<br>89	GO:00<br>16598	protein arginylation	19/ 0.5 0.9 0.9<br>12 414 999 444<br>75 407 945 234<br>6 01 94 6	Soltu.DM.10G020990/Soltu.DM.05G026870	2
12<br>90	GO:00<br>43449	cellular alkene metabolic process	19/ 0.5 0.9 0.9<br>12 414 999 444<br>75 407 945 234<br>6 01 94 6	Soltu.DM.11G009500/Soltu.DM.02G004480	2
12<br>91	GO:00<br>43450	alkene biosynthetic process	19/ 0.5 0.9 0.9<br>12 414 999 444<br>75 407 945 234<br>6 01 94 6	Soltu.DM.11G009500/Soltu.DM.02G004480	2
12<br>92	GO:00<br>44057	regulation of system process	19/ 0.5 0.9 0.9<br>12 414 999 444<br>75 407 945 234<br>6 01 94 6	Soltu.DM.02G018520/Soltu.DM.10G027270	2
12<br>93	GO:00<br>45010	actin nucleation	19/ 0.5 0.9 0.9<br>12 414 999 444<br>75 407 945 234<br>6 01 94 6	Soltu.DM.09G015150/Soltu.DM.09G027230	2
12<br>94	GO:19<br>00378	positive regulation of secondary metabolite biosynthetic process	19/ 0.5 0.9 0.9<br>12 414 999 444<br>75 407 945 234<br>6 01 94 6	Soltu.DM.12G022190/Soltu.DM.02G019030	2
12<br>95	GO:19<br>00674	olefin biosynthetic process	19/ 0.5 0.9 0.9<br>12 414 999 444<br>75 407 945 234<br>6 01 94 6	Soltu.DM.11G009500/Soltu.DM.02G004480	2
12<br>96	GO:00<br>09112	nucleobase metabolic process	41/ 0.5 0.9 0.9<br>12 414 999 444<br>75 640 945 234<br>6 75 94 6	Soltu.DM.02G017810/Soltu.DM.10G012990/Soltu.DM.03G019550/Soltu.DM.01G017170	4
12<br>97	GO:00<br>35196	miRNA processing	41/ 0.5 0.9 0.9<br>12 414 999 444<br>75 640 945 234<br>6 75 94 6	Soltu.DM.12G005490/Soltu.DM.12G026070/Soltu.DM.11G025410/Soltu.DM.04G031030	4
12<br>98	GO:00<br>90329	regulation of DNA-templated DNA replication	41/ 0.5 0.9 0.9<br>12 414 999 444	Soltu.DM.11G009630/Soltu.DM.08G027160/Soltu.DM.03G032560/Soltu.DM.12G030150	4

			756	64075	94594	2346		
1299	GO:1900150	regulation of defense response to fungus	41/12756	0.541464075	0.999994594	0.94442346	Soltu.DM.06G017300/Soltu.DM.12G026070/Soltu.DM.12G010960/Soltu.DM.03G008510	4
1300	GO:0051054	positive regulation of DNA metabolic process	52/12756	0.542384834	0.999994594	0.94442346	Soltu.DM.06G026960/Soltu.DM.11G009630/Soltu.DM.08G027160/Soltu.DM.01G002690/Soltu.DM.02G013390	5
1301	GO:0000398	mRNA splicing, via spliceosome	150/12756	0.543274417	0.999994594	0.94442346	Soltu.DM.02G018020/Soltu.DM.04G029350/Soltu.DM.12G005490/Soltu.DM.02G025260/Soltu.DM.12G025260/Soltu.DM.11G014750/Soltu.DM.02G021990/Soltu.DM.11G021400/Soltu.DM.09G001490/Soltu.DM.10G028250/Soltu.DM.11G025410/Soltu.DM.10G001400/Soltu.DM.12G025710/Soltu.DM.07G006530	14
1302	GO:0010928	regulation of auxin mediated signaling pathway	63/12756	0.543508703	0.999994594	0.94442346	Soltu.DM.04G033180/Soltu.DM.02G032050/Soltu.DM.10G026500/Soltu.DM.03G036780/Soltu.DM.02G022410/Soltu.DM.04G002690	6
1303	GO:0045787	positive regulation of cell cycle	63/12756	0.543508703	0.999994594	0.94442346	Soltu.DM.02G018520/Soltu.DM.03G003730/Soltu.DM.11G016820/Soltu.DM.02G029580/Soltu.DM.12G023230/Soltu.DM.03G020440	6
1304	GO:0048767	root hair elongation	63/12756	0.543508703	0.999994594	0.94442346	Soltu.DM.08G027650/Soltu.DM.12G005490/Soltu.DM.08G023170/Soltu.DM.12G024710/Soltu.DM.09G026810/Soltu.DM.04G002690	6
1305	GO:0000098	sulfur amino acid catabolic process	8/12756	0.54391029	0.999994594	0.94442346	Soltu.DM.08G030020	1
1306	GO:0000393	spliceosomal conformational changes to generate catalytic conformation	8/12756	0.54391029	0.999994594	0.94442346	Soltu.DM.12G025260	1
1307	GO:0006105	succinate metabolic process	8/12756	0.54391029	0.999994594	0.94442346	Soltu.DM.12G024030	1
1308	GO:0006432	phenylalanyl-tRNA aminoacylation	8/12756	0.54391029	0.999994594	0.94442346	Soltu.DM.07G027760	1
1309	GO:0006549	isoleucine metabolic process	8/12756	0.54391029	0.999994594	0.94442346	Soltu.DM.11G003850	1
1310	GO:0006553	lysine metabolic process	8/12756	0.54391029	0.999994594	0.94442346	Soltu.DM.07G006550	1
1311	GO:0006651	diacylglycerol biosynthetic process	8/12756	0.54391029	0.999994594	0.94442346	Soltu.DM.02G024810	1
1312	GO:0006734	NADH metabolic process	8/12756	0.54391029	0.999994594	0.94442346	Soltu.DM.02G018700	1
1313	GO:0007268	chemical synaptic transmission	8/12756	0.54391029	0.999994594	0.94442346	Soltu.DM.11G026460	1
1314	GO:0009097	isoleucine biosynthetic process	8/12756	0.54391029	0.999994594	0.94442346	Soltu.DM.11G003850	1
1315	GO:0009823	cytokinin catabolic process	8/12756	0.54391029	0.999994594	0.94442346	Soltu.DM.04G011550	1
13	GO:00	response to boron-containing	8/1	0.5	0.9	0.9	Soltu.DM.03G031200	1

16	10036	substance	27<br>56	439<br>102<br>9	999<br>945<br>94	444<br>234<br>6		
13<br>17	GO:00<br>10219	regulation of vernalization response	8/1<br>27<br>56	0.5<br>439<br>102<br>9	0.9<br>999<br>945<br>94	0.9<br>444<br>234<br>6	Soltu.DM.05G012040	1
13<br>18	GO:00<br>10244	response to low fluence blue light stimulus by blue low-fluence system	8/1<br>27<br>56	0.5<br>439<br>102<br>9	0.9<br>999<br>945<br>94	0.9<br>444<br>234<br>6	Soltu.DM.08G011890	1
13<br>19	GO:00<br>10310	regulation of hydrogen peroxide metabolic process	8/1<br>27<br>56	0.5<br>439<br>102<br>9	0.9<br>999<br>945<br>94	0.9<br>444<br>234<br>6	Soltu.DM.06G026400	1
13<br>20	GO:00<br>10581	regulation of starch biosynthetic process	8/1<br>27<br>56	0.5<br>439<br>102<br>9	0.9<br>999<br>945<br>94	0.9<br>444<br>234<br>6	Soltu.DM.08G008380	1
13<br>21	GO:00<br>16094	polyprenol biosynthetic process	8/1<br>27<br>56	0.5<br>439<br>102<br>9	0.9<br>999<br>945<br>94	0.9<br>444<br>234<br>6	Soltu.DM.10G022710	1
13<br>22	GO:00<br>16145	S-glycoside catabolic process	8/1<br>27<br>56	0.5<br>439<br>102<br>9	0.9<br>999<br>945<br>94	0.9<br>444<br>234<br>6	Soltu.DM.03G035710	1
13<br>23	GO:00<br>19322	pentose biosynthetic process	8/1<br>27<br>56	0.5<br>439<br>102<br>9	0.9<br>999<br>945<br>94	0.9<br>444<br>234<br>6	Soltu.DM.02G012570	1
13<br>24	GO:00<br>19566	arabinose metabolic process	8/1<br>27<br>56	0.5<br>439<br>102<br>9	0.9<br>999<br>945<br>94	0.9<br>444<br>234<br>6	Soltu.DM.02G012570	1
13<br>25	GO:00<br>19567	arabinose biosynthetic process	8/1<br>27<br>56	0.5<br>439<br>102<br>9	0.9<br>999<br>945<br>94	0.9<br>444<br>234<br>6	Soltu.DM.02G012570	1
13<br>26	GO:00<br>19759	glycosinolate catabolic process	8/1<br>27<br>56	0.5<br>439<br>102<br>9	0.9<br>999<br>945<br>94	0.9<br>444<br>234<br>6	Soltu.DM.03G035710	1
13<br>27	GO:00<br>19762	glucosinolate catabolic process	8/1<br>27<br>56	0.5<br>439<br>102<br>9	0.9<br>999<br>945<br>94	0.9<br>444<br>234<br>6	Soltu.DM.03G035710	1
13<br>28	GO:00<br>19919	peptidyl-arginine methylation, to asymmetrical-dimethyl arginine	8/1<br>27<br>56	0.5<br>439<br>102<br>9	0.9<br>999<br>945<br>94	0.9<br>444<br>234<br>6	Soltu.DM.06G017810	1
13<br>29	GO:00<br>21953	central nervous system neuron differentiation	8/1<br>27<br>56	0.5<br>439<br>102<br>9	0.9<br>999<br>945<br>94	0.9<br>444<br>234<br>6	Soltu.DM.01G042210	1
13<br>30	GO:00<br>30330	DNA damage response, signal transduction by p53 class mediator	8/1<br>27<br>56	0.5<br>439<br>102<br>9	0.9<br>999<br>945<br>94	0.9<br>444<br>234<br>6	Soltu.DM.11G016820	1
13<br>31	GO:00<br>30856	regulation of epithelial cell differentiation	8/1<br>27<br>56	0.5<br>439<br>102<br>9	0.9<br>999<br>945<br>94	0.9<br>444<br>234<br>6	Soltu.DM.10G024000	1
13<br>32	GO:00<br>31086	nuclear-transcribed mRNA catabolic process, deadenylation-independent decay	8/1<br>27<br>56	0.5<br>439<br>102<br>9	0.9<br>999<br>945<br>94	0.9<br>444<br>234<br>6	Soltu.DM.05G026810	1
13<br>33	GO:00<br>31087	deadenylation-independent decapping of nuclear-transcribed mRNA	8/1<br>27<br>56	0.5<br>439<br>102<br>9	0.9<br>999<br>945<br>94	0.9<br>444<br>234<br>6	Soltu.DM.05G026810	1

1334	GO:0032042	mitochondrial DNA metabolic process	8/12756	0.54391029	0.999994594	0.94442346	Soltu.DM.08G023320	1
1335	GO:0032205	negative regulation of telomere maintenance	8/12756	0.54391029	0.999994594	0.94442346	Soltu.DM.05G006310	1
1336	GO:0034517	ribophagy	8/12756	0.54391029	0.999994594	0.94442346	Soltu.DM.02G030830	1
1337	GO:0035973	aggrephagy	8/12756	0.54391029	0.999994594	0.94442346	Soltu.DM.02G030830	1
1338	GO:0042276	error-prone translesion synthesis	8/12756	0.54391029	0.999994594	0.94442346	Soltu.DM.08G027160	1
1339	GO:0042723	thiamine-containing compound metabolic process	8/12756	0.54391029	0.999994594	0.94442346	Soltu.DM.06G003240	1
1340	GO:0042724	thiamine-containing compound biosynthetic process	8/12756	0.54391029	0.999994594	0.94442346	Soltu.DM.06G003240	1
1341	GO:0043271	negative regulation of monoatomic ion transport	8/12756	0.54391029	0.999994594	0.94442346	Soltu.DM.05G011970	1
1342	GO:0043617	cellular response to sucrose starvation	8/12756	0.54391029	0.999994594	0.94442346	Soltu.DM.04G025250	1
1343	GO:0043620	regulation of DNA-templated transcription in response to stress	8/12756	0.54391029	0.999994594	0.94442346	Soltu.DM.06G020020	1
1344	GO:0045682	regulation of epidermis development	8/12756	0.54391029	0.999994594	0.94442346	Soltu.DM.10G024000	1
1345	GO:0046487	glyoxylate metabolic process	8/12756	0.54391029	0.999994594	0.94442346	Soltu.DM.07G017900	1
1346	GO:0046605	regulation of centrosome cycle	8/12756	0.54391029	0.999994594	0.94442346	Soltu.DM.03G003730	1
1347	GO:0046683	response to organophosphorus	8/12756	0.54391029	0.999994594	0.94442346	Soltu.DM.01G028770	1
1348	GO:0048255	mRNA stabilization	8/12756	0.54391029	0.999994594	0.94442346	Soltu.DM.11G016820	1
1349	GO:0048759	xylem vessel member cell differentiation	8/12756	0.54391029	0.999994594	0.94442346	Soltu.DM.11G011390	1
1350	GO:0050691	regulation of defense response to virus by host	8/12756	0.54391029	0.999994594	0.94442346	Soltu.DM.03G021360	1
1351	GO:0051092	positive regulation of NF-kappaB transcription factor activity	8/12756	0.5439102	0.9999945	0.9444234	Soltu.DM.02G018520	1

				9	94	6		
1352	GO:0051176	positive regulation of sulfur metabolic process	8/12756	0.54391029	0.999994594	0.94442346	Soltu.DM.12G022190	1
1353	GO:0051290	protein heterotetramerization	8/12756	0.54391029	0.999994594	0.94442346	Soltu.DM.03G012810	1
1354	GO:0051785	positive regulation of nuclear division	8/12756	0.54391029	0.999994594	0.94442346	Soltu.DM.03G003730	1
1355	GO:0060919	auxin import into cell	8/12756	0.54391029	0.999994594	0.94442346	Soltu.DM.02G032050	1
1356	GO:0090257	regulation of muscle system process	8/12756	0.54391029	0.999994594	0.94442346	Soltu.DM.02G018520	1
1357	GO:0098916	anterograde trans-synaptic signaling	8/12756	0.54391029	0.999994594	0.94442346	Soltu.DM.11G026460	1
1358	GO:0099536	synaptic signaling	8/12756	0.54391029	0.999994594	0.94442346	Soltu.DM.11G026460	1
1359	GO:0099537	trans-synaptic signaling	8/12756	0.54391029	0.999994594	0.94442346	Soltu.DM.11G026460	1
1360	GO:1902373	negative regulation of mRNA catabolic process	8/12756	0.54391029	0.999994594	0.94442346	Soltu.DM.11G016820	1
1361	GO:1902476	chloride transmembrane transport	8/12756	0.54391029	0.999994594	0.94442346	Soltu.DM.02G010790	1
1362	GO:1904357	negative regulation of telomere maintenance via telomere lengthening	8/12756	0.54391029	0.999994594	0.94442346	Soltu.DM.05G006310	1
1363	GO:2000045	regulation of G1/S transition of mitotic cell cycle	8/12756	0.54391029	0.999994594	0.94442346	Soltu.DM.11G016820	1
1364	GO:0071166	ribonucleoprotein complex localization	74/12756	0.544732493	0.999994594	0.94442346	Soltu.DM.12G005490/Soltu.DM.03G003730/Soltu.DM.12G024350/Soltu.DM.09G005370/Soltu.DM.11G024760/Soltu.DM.04G011330/Soltu.DM.07G006510	7
1365	GO:0090056	regulation of chlorophyll metabolic process	96/12756	0.547293854	0.999994594	0.94442346	Soltu.DM.04G011110/Soltu.DM.09G025070/Soltu.DM.10G027470/Soltu.DM.04G011320/Soltu.DM.04G011240/Soltu.DM.10G028070/Soltu.DM.05G011970/Soltu.DM.04G011370/Soltu.DM.11G021090	9
1366	GO:0045089	positive regulation of innate immune response	118/12756	0.549872934	0.999994594	0.94442346	Soltu.DM.02G025590/Soltu.DM.07G028550/Soltu.DM.01G024680/Soltu.DM.10G000640/Soltu.DM.04G018070/Soltu.DM.08G011890/Soltu.DM.02G022460/Soltu.DM.01G051770/Soltu.DM.03G008510/Soltu.DM.08G020460/Soltu.DM.02G026820	11
1367	GO:0050778	positive regulation of immune response	129/12756	0.551146903	0.999994594	0.94442346	Soltu.DM.02G025590/Soltu.DM.07G028550/Soltu.DM.01G024680/Soltu.DM.10G000640/Soltu.DM.04G018070/Soltu.DM.08G011890/Soltu.DM.07G014680/Soltu.DM.02G022460/Soltu.DM.01G051770/Soltu.DM.03G008510/Soltu.DM.08G020460/Soltu.DM.02G026820	12
1368	GO:0007167	enzyme-linked receptor protein signaling pathway	162/12756	0.55486978	0.999994594	0.94442346	Soltu.DM.04G024310/Soltu.DM.08G023130/Soltu.DM.12G001970/Soltu.DM.01G002930/Soltu.DM.02G026780/Soltu.DM.02G020600/Soltu.DM.09G026810/Soltu.DM.03G031770/Soltu.DM.01G031880/Soltu.DM.02G019190/Soltu.DM.09G029750/Soltu.DM.03G001740/Soltu.DM.03G037530/Soltu.DM.07G020980/Soltu.DM.01G051120	15
1369	GO:0031348	negative regulation of defense response	75/12	0.5593	0.9999	0.9444	Soltu.DM.03G035710/Soltu.DM.11G010230/Soltu.DM.08G022900/Soltu.DM.11G010220/Soltu.DM.06G015770/Soltu.DM.02G023840/Soltu.DM.05G023030	7

			756	79001	94594	2346		
1370	GO:0032970	regulation of actin filament-based process	75/12756	0.559379001	0.999994594	0.94442346	Soltu.DM.06G022970/Soltu.DM.01G028770/Soltu.DM.09G015150/Soltu.DM.12G009990/Soltu.DM.10G004310/Soltu.DM.09G027230/Soltu.DM.12G023230	7
1371	GO:2001251	negative regulation of chromosome organization	53/12756	0.559808513	0.999994594	0.94442346	Soltu.DM.08G022190/Soltu.DM.10G020590/Soltu.DM.04G038280/Soltu.DM.05G006310/Soltu.DM.10G024770	5
1372	GO:0010639	negative regulation of organelle organization	108/12756	0.560792359	0.999994594	0.94442346	Soltu.DM.08G022190/Soltu.DM.10G020590/Soltu.DM.06G022970/Soltu.DM.01G028770/Soltu.DM.04G038280/Soltu.DM.09G015150/Soltu.DM.05G006310/Soltu.DM.12G009990/Soltu.DM.10G024770/Soltu.DM.10G004310	10
1373	GO:0072530	purine-containing compound transmembrane transport	42/12756	0.561038417	0.999994594	0.94442346	Soltu.DM.04G030440/Soltu.DM.07G000710/Soltu.DM.06G012690/Soltu.DM.02G014120	4
1374	GO:0031349	positive regulation of defense response	141/12756	0.563087868	0.999994594	0.94442346	Soltu.DM.02G025590/Soltu.DM.07G028550/Soltu.DM.01G024680/Soltu.DM.10G000640/Soltu.DM.04G018070/Soltu.DM.08G011890/Soltu.DM.02G022460/Soltu.DM.01G051770/Soltu.DM.03G008510/Soltu.DM.08G020460/Soltu.DM.04G000670/Soltu.DM.09G026500/Soltu.DM.02G026820	13
1375	GO:0044782	cilium organization	31/12756	0.563758793	0.999994594	0.94442346	Soltu.DM.09G019870/Soltu.DM.01G020880/Soltu.DM.07G024370	3
1376	GO:0071731	response to nitric oxide	31/12756	0.563758793	0.999994594	0.94442346	Soltu.DM.07G028550/Soltu.DM.10G000640/Soltu.DM.02G030410	3
1377	GO:0097366	response to bronchodilator	31/12756	0.563758793	0.999994594	0.94442346	Soltu.DM.07G028550/Soltu.DM.10G000640/Soltu.DM.02G030410	3
1378	GO:1903320	regulation of protein modification by small protein conjugation or removal	31/12756	0.563758793	0.999994594	0.94442346	Soltu.DM.02G018520/Soltu.DM.06G015770/Soltu.DM.09G005140	3
1379	GO:1905037	autophagosome organization	31/12756	0.563758793	0.999994594	0.94442346	Soltu.DM.01G039130/Soltu.DM.11G022310/Soltu.DM.09G025980	3
1380	GO:0071695	anatomical structure maturation	174/12756	0.565690746	0.999994594	0.94442346	Soltu.DM.07G013360/Soltu.DM.08G027650/Soltu.DM.04G001370/Soltu.DM.12G004060/Soltu.DM.12G005490/Soltu.DM.08G023170/Soltu.DM.12G024710/Soltu.DM.09G026810/Soltu.DM.08G011330/Soltu.DM.03G001740/Soltu.DM.01G042120/Soltu.DM.01G001590/Soltu.DM.03G013100/Soltu.DM.07G024500/Soltu.DM.03G018740/Soltu.DM.04G002690	16
1381	GO:0000077	DNA damage checkpoint signaling	20/12756	0.569840814	0.999994594	0.94442346	Soltu.DM.11G016820/Soltu.DM.02G033290	2
1382	GO:0031365	N-terminal protein amino acid modification	20/12756	0.569840814	0.999994594	0.94442346	Soltu.DM.06G019850/Soltu.DM.01G002490	2
1383	GO:0032507	maintenance of protein location in cell	20/12756	0.569840814	0.999994594	0.94442346	Soltu.DM.06G022970/Soltu.DM.12G009990	2
1384	GO:0043289	apocarotenoid biosynthetic process	20/12756	0.569840814	0.999994594	0.94442346	Soltu.DM.11G024450/Soltu.DM.06G029640	2
1385	GO:0044774	mitotic DNA integrity checkpoint signaling	20/12756	0.569840814	0.999994594	0.94442346	Soltu.DM.11G016820/Soltu.DM.02G033290	2
1386	GO:0046864	isoprenoid transport	20/12756	0.569840814	0.999994594	0.94442346	Soltu.DM.09G028710/Soltu.DM.11G011430	2
13	GO:00	terpenoid transport	20/	0.5	0.9	0.9	Soltu.DM.09G028710/Soltu.DM.11G011430	2

87	46865		12756	69840814	99994594	4442346		
1388	GO:0048859	formation of anatomical boundary	20/12756	0.569840814	0.999994594	0.94442346	Soltu.DM.08G013580/Soltu.DM.02G016680	2
1389	GO:0051972	regulation of telomerase activity	20/12756	0.569840814	0.999994594	0.94442346	Soltu.DM.06G026960/Soltu.DM.02G013390	2
1390	GO:0140029	exocytic process	20/12756	0.569840814	0.999994594	0.94442346	Soltu.DM.06G005370/Soltu.DM.11G026460	2
1391	GO:1900457	regulation of brassinosteroid mediated signaling pathway	20/12756	0.569840814	0.999994594	0.94442346	Soltu.DM.07G021000/Soltu.DM.01G003190	2
1392	GO:1902645	tertiary alcohol biosynthetic process	20/12756	0.569840814	0.999994594	0.94442346	Soltu.DM.11G024450/Soltu.DM.06G029640	2
1393	GO:1903008	organelle disassembly	20/12756	0.569840814	0.999994594	0.94442346	Soltu.DM.01G039130/Soltu.DM.11G022310	2
1394	GO:2000034	regulation of seed maturation	20/12756	0.569840814	0.999994594	0.94442346	Soltu.DM.07G010930/Soltu.DM.01G040220	2
1395	GO:1901401	regulation of tetrapyrrole metabolic process	98/12756	0.572897813	0.999994594	0.94442346	Soltu.DM.04G011110/Soltu.DM.09G025070/Soltu.DM.10G027470/Soltu.DM.04G011320/Soltu.DM.04G011240/Soltu.DM.10G028070/Soltu.DM.05G011970/Soltu.DM.04G011370/Soltu.DM.11G021090	9
1396	GO:0009150	purine ribonucleotide metabolic process	142/12756	0.573672777	0.999994594	0.94442346	Soltu.DM.12G004480/Soltu.DM.01G033530/Soltu.DM.02G018700/Soltu.DM.03G037170/Soltu.DM.07G009580/Soltu.DM.10G027910/Soltu.DM.06G013720/Soltu.DM.03G021730/Soltu.DM.08G026810/Soltu.DM.02G031030/Soltu.DM.11G025570/Soltu.DM.11G010590/Soltu.DM.01G019520	13
1397	GO:0006611	protein export from nucleus	76/12756	0.573811434	0.999994594	0.94442346	Soltu.DM.12G005490/Soltu.DM.03G003730/Soltu.DM.12G024350/Soltu.DM.09G005370/Soltu.DM.11G024760/Soltu.DM.04G011330/Soltu.DM.07G006510	7
1398	GO:0022411	cellular component disassembly	65/12756	0.574956	0.999994594	0.94442346	Soltu.DM.07G001240/Soltu.DM.08G019530/Soltu.DM.01G039130/Soltu.DM.01G028770/Soltu.DM.11G010650/Soltu.DM.11G022310	6
1399	GO:1902903	regulation of supramolecular fiber organization	65/12756	0.574956	0.999994594	0.94442346	Soltu.DM.06G022970/Soltu.DM.01G028770/Soltu.DM.09G015150/Soltu.DM.12G009990/Soltu.DM.10G004310/Soltu.DM.09G027230	6
1400	GO:0031124	mRNA 3'-end processing	43/12756	0.580148751	0.999994594	0.94442346	Soltu.DM.01G006380/Soltu.DM.12G005490/Soltu.DM.03G003370/Soltu.DM.03G019650	4
1401	GO:0048870	cell motility	43/12756	0.580148751	0.999994594	0.94442346	Soltu.DM.08G027150/Soltu.DM.01G028770/Soltu.DM.09G002090/Soltu.DM.10G004310	4
1402	GO:0051674	localization of cell	43/12756	0.580148751	0.999994594	0.94442346	Soltu.DM.08G027150/Soltu.DM.01G028770/Soltu.DM.09G002090/Soltu.DM.10G004310	4
1403	GO:0051129	negative regulation of cellular component organization	143/12756	0.584154084	0.999994594	0.94442346	Soltu.DM.12G020370/Soltu.DM.08G022190/Soltu.DM.12G020350/Soltu.DM.10G020590/Soltu.DM.06G022970/Soltu.DM.01G028770/Soltu.DM.04G038280/Soltu.DM.09G015150/Soltu.DM.05G006310/Soltu.DM.12G009990/Soltu.DM.10G024770/Soltu.DM.10G004310/Soltu.DM.12G020340	13
1404	GO:0018105	peptidyl-serine phosphorylation	99/12756	0.585458889	0.999994594	0.94442346	Soltu.DM.06G002800/Soltu.DM.05G023210/Soltu.DM.06G032750/Soltu.DM.06G010880/Soltu.DM.03G021780/Soltu.DM.02G023840/Soltu.DM.07G026270/Soltu.DM.03G016650/Soltu.DM.11G004950	9

1405	GO:0010103	stomatal complex morphogenesis	32/12756	0.585843859	0.999994594	0.94442346	Soltu.DM.05G021390/Soltu.DM.12G010960/Soltu.DM.08G012010	3
1406	GO:0046173	polyol biosynthetic process	32/12756	0.585843859	0.999994594	0.94442346	Soltu.DM.02G018520/Soltu.DM.08G014180/Soltu.DM.03G008510	3
1407	GO:0050777	negative regulation of immune response	32/12756	0.585843859	0.999994594	0.94442346	Soltu.DM.08G022900/Soltu.DM.04G022240/Soltu.DM.05G023030	3
1408	GO:0065001	specification of axis polarity	32/12756	0.585843859	0.999994594	0.94442346	Soltu.DM.02G003130/Soltu.DM.07G026690/Soltu.DM.08G012010	3
1409	GO:0000056	ribosomal small subunit export from nucleus	9/12756	0.586556896	0.999994594	0.94442346	Soltu.DM.07G006510	1
1410	GO:0001503	ossification	9/12756	0.586556896	0.999994594	0.94442346	Soltu.DM.01G002690	1
1411	GO:0006085	acetyl-CoA biosynthetic process	9/12756	0.586556896	0.999994594	0.94442346	Soltu.DM.01G019520	1
1412	GO:0006152	purine nucleoside catabolic process	9/12756	0.586556896	0.999994594	0.94442346	Soltu.DM.12G003790	1
1413	GO:0006572	tyrosine catabolic process	9/12756	0.586556896	0.999994594	0.94442346	Soltu.DM.04G009170	1
1414	GO:0007632	visual behavior	9/12756	0.586556896	0.999994594	0.94442346	Soltu.DM.06G005370	1
1415	GO:0007634	optokinetic behavior	9/12756	0.586556896	0.999994594	0.94442346	Soltu.DM.06G005370	1
1416	GO:0009147	pyrimidine nucleoside triphosphate metabolic process	9/12756	0.586556896	0.999994594	0.94442346	Soltu.DM.01G017170	1
1417	GO:0009148	pyrimidine nucleoside triphosphate biosynthetic process	9/12756	0.586556896	0.999994594	0.94442346	Soltu.DM.01G017170	1
1418	GO:0009649	entrainment of circadian clock	9/12756	0.586556896	0.999994594	0.94442346	Soltu.DM.11G001010	1
1419	GO:0009939	positive regulation of gibberellic acid mediated signaling pathway	9/12756	0.586556896	0.999994594	0.94442346	Soltu.DM.09G022610	1
1420	GO:0010050	vegetative phase change	9/12756	0.586556896	0.999994594	0.94442346	Soltu.DM.01G010020	1
1421	GO:0010071	root meristem specification	9/12756	0.586556896	0.999994594	0.94442346	Soltu.DM.03G034800	1
1422	GO:0010559	regulation of glycoprotein biosynthetic process	9/12756	0.5865568	0.9999945	0.9444234	Soltu.DM.01G033530	1

				96	94	6		
1423	GO:0015721	bile acid and bile salt transport	9/12756	0.586556896	0.999994594	0.94442346	Soltu.DM.06G005370	1
1424	GO:0016574	histone ubiquitination	9/12756	0.586556896	0.999994594	0.94442346	Soltu.DM.01G001590	1
1425	GO:0019430	removal of superoxide radicals	9/12756	0.586556896	0.999994594	0.94442346	Soltu.DM.06G012170	1
1426	GO:0030002	cellular monoatomic anion homeostasis	9/12756	0.586556896	0.999994594	0.94442346	Soltu.DM.02G034460	1
1427	GO:0030518	intracellular steroid hormone receptor signaling pathway	9/12756	0.586556896	0.999994594	0.94442346	Soltu.DM.04G038280	1
1428	GO:0030903	notochord development	9/12756	0.586556896	0.999994594	0.94442346	Soltu.DM.06G005370	1
1429	GO:0031119	tRNA pseudouridine synthesis	9/12756	0.586556896	0.999994594	0.94442346	Soltu.DM.01G046920	1
1430	GO:0031268	pseudopodium organization	9/12756	0.586556896	0.999994594	0.94442346	Soltu.DM.01G028770	1
1431	GO:0032796	uropod organization	9/12756	0.586556896	0.999994594	0.94442346	Soltu.DM.01G028770	1
1432	GO:0034389	lipid droplet organization	9/12756	0.586556896	0.999994594	0.94442346	Soltu.DM.09G023300	1
1433	GO:0034461	uropod retraction	9/12756	0.586556896	0.999994594	0.94442346	Soltu.DM.01G028770	1
1434	GO:0035247	peptidyl-arginine omega-N-methylation	9/12756	0.586556896	0.999994594	0.94442346	Soltu.DM.06G017810	1
1435	GO:0035435	phosphate ion transmembrane transport	9/12756	0.586556896	0.999994594	0.94442346	Soltu.DM.05G021160	1
1436	GO:0036065	fucosylation	9/12756	0.586556896	0.999994594	0.94442346	Soltu.DM.03G037510	1
1437	GO:0042343	indole glucosinolate metabolic process	9/12756	0.586556896	0.999994594	0.94442346	Soltu.DM.03G035710	1
1438	GO:0042727	flavin-containing compound biosynthetic process	9/12756	0.586556896	0.999994594	0.94442346	Soltu.DM.01G044760	1
1439	GO:0043485	endosome to pigment granule transport	9/12756	0.586556896	0.999994594	0.94442346	Soltu.DM.06G005370	1
1440	GO:0048069	eye pigmentation	9/127	0.5865	0.9999	0.9444	Soltu.DM.06G005370	1

			56	56896	94594	2346		
1441	GO:0048489	synaptic vesicle transport	9/12756	0.586556896	0.999994594	0.94442346	Soltu.DM.11G026460	1
1442	GO:0048757	pigment granule maturation	9/12756	0.586556896	0.999994594	0.94442346	Soltu.DM.06G005370	1
1443	GO:0050685	positive regulation of mRNA processing	9/12756	0.586556896	0.999994594	0.94442346	Soltu.DM.12G025260	1
1444	GO:0051055	negative regulation of lipid biosynthetic process	9/12756	0.586556896	0.999994594	0.94442346	Soltu.DM.08G029860	1
1445	GO:0051775	response to redox state	9/12756	0.586556896	0.999994594	0.94442346	Soltu.DM.06G028040	1
1446	GO:0051924	regulation of calcium ion transport	9/12756	0.586556896	0.999994594	0.94442346	Soltu.DM.02G020550	1
1447	GO:0055071	manganese ion homeostasis	9/12756	0.586556896	0.999994594	0.94442346	Soltu.DM.04G003430	1
1448	GO:0060034	notochord cell differentiation	9/12756	0.586556896	0.999994594	0.94442346	Soltu.DM.06G005370	1
1449	GO:0060035	notochord cell development	9/12756	0.586556896	0.999994594	0.94442346	Soltu.DM.06G005370	1
1450	GO:0060036	notochord cell vacuolation	9/12756	0.586556896	0.999994594	0.94442346	Soltu.DM.06G005370	1
1451	GO:0070734	histone H3-K27 methylation	9/12756	0.586556896	0.999994594	0.94442346	Soltu.DM.01G024940	1
1452	GO:0071450	cellular response to oxygen radical	9/12756	0.586556896	0.999994594	0.94442346	Soltu.DM.06G012170	1
1453	GO:0071451	cellular response to superoxide	9/12756	0.586556896	0.999994594	0.94442346	Soltu.DM.06G012170	1
1454	GO:0071484	cellular response to light intensity	9/12756	0.586556896	0.999994594	0.94442346	Soltu.DM.09G025070	1
1455	GO:0072332	intrinsic apoptotic signaling pathway by p53 class mediator	9/12756	0.586556896	0.999994594	0.94442346	Soltu.DM.12G025260	1
1456	GO:0080111	DNA demethylation	9/12756	0.586556896	0.999994594	0.94442346	Soltu.DM.10G024770	1
1457	GO:0097479	synaptic vesicle localization	9/12756	0.586556896	0.999994594	0.94442346	Soltu.DM.11G026460	1
14	GO:00	establishment of synaptic vesicle	9/1	0.5	0.9	0.9	Soltu.DM.11G026460	1

58	97480	localization	27 56	865 568 96	999 945 94	444 234 6		
1459	GO:0099504	synaptic vesicle cycle	9/12756	0.586556896	0.99994594	0.94442346	Soltu.DM.11G026460	1
1460	GO:1903018	regulation of glycoprotein metabolic process	9/12756	0.586556896	0.99994594	0.94442346	Soltu.DM.01G033530	1
1461	GO:1905330	regulation of morphogenesis of an epithelium	9/12756	0.586556896	0.99994594	0.94442346	Soltu.DM.07G002580	1
1462	GO:2000904	regulation of starch metabolic process	9/12756	0.586556896	0.99994594	0.94442346	Soltu.DM.08G008380	1
1463	GO:0010476	gibberellin mediated signaling pathway	77/12756	0.588016551	0.99994594	0.94442346	Soltu.DM.02G011120/Soltu.DM.09G022610/Soltu.DM.06G012790/Soltu.DM.08G011890/Soltu.DM.01G024340/Soltu.DM.06G002140/Soltu.DM.06G023440	7
1464	GO:0031098	stress-activated protein kinase signaling cascade	55/12756	0.593620997	0.99994594	0.94442346	Soltu.DM.07G017210/Soltu.DM.07G017190/Soltu.DM.07G017200/Soltu.DM.07G017180/Soltu.DM.02G028740	5
1465	GO:0072527	pyrimidine-containing compound metabolic process	55/12756	0.593620997	0.99994594	0.94442346	Soltu.DM.11G007630/Soltu.DM.10G012990/Soltu.DM.03G019550/Soltu.DM.01G017170/Soltu.DM.06G003240	5
1466	GO:0002684	positive regulation of immune system process	133/12756	0.59497609	0.99994594	0.94442346	Soltu.DM.02G025590/Soltu.DM.07G028550/Soltu.DM.01G024680/Soltu.DM.10G000640/Soltu.DM.04G018070/Soltu.DM.08G011890/Soltu.DM.07G014680/Soltu.DM.02G022460/Soltu.DM.01G051770/Soltu.DM.03G008510/Soltu.DM.08G020460/Soltu.DM.02G026820	12
1467	GO:0000479	endonucleolytic cleavage of tricistronic rRNA transcript (SSU-rRNA, 5.8S rRNA, LSU-rRNA)	21/12756	0.596942416	0.99994594	0.94442346	Soltu.DM.12G024350/Soltu.DM.01G051600	2
1468	GO:0007155	cell adhesion	21/12756	0.596942416	0.99994594	0.94442346	Soltu.DM.12G024030/Soltu.DM.08G001690	2
1469	GO:0010959	regulation of metal ion transport	21/12756	0.596942416	0.99994594	0.94442346	Soltu.DM.02G020550/Soltu.DM.05G011970	2
1470	GO:0015743	malate transport	21/12756	0.596942416	0.99994594	0.94442346	Soltu.DM.11G000570/Soltu.DM.05G021160	2
1471	GO:0031057	negative regulation of histone modification	21/12756	0.596942416	0.99994594	0.94442346	Soltu.DM.08G022190/Soltu.DM.04G038280	2
1472	GO:0035065	regulation of histone acetylation	21/12756	0.596942416	0.99994594	0.94442346	Soltu.DM.08G022190/Soltu.DM.04G038280	2
1473	GO:0045185	maintenance of protein location	21/12756	0.596942416	0.99994594	0.94442346	Soltu.DM.06G022970/Soltu.DM.12G009990	2
1474	GO:0046519	sphingoid metabolic process	21/12756	0.596942416	0.99994594	0.94442346	Soltu.DM.02G018520/Soltu.DM.08G014180	2
1475	GO:0071423	malate transmembrane transport	21/12756	0.596942416	0.99994594	0.94442346	Soltu.DM.11G000570/Soltu.DM.05G021160	2

1476	GO:0080186	developmental vegetative growth	21/12756	0.596942416	0.999994594	0.94442346	Soltu.DM.02G025590/Soltu.DM.09G023300	2
1477	GO:1901983	regulation of protein acetylation	21/12756	0.596942416	0.999994594	0.94442346	Soltu.DM.08G022190/Soltu.DM.04G038280	2
1478	GO:1903959	regulation of monoatomic anion transmembrane transport	21/12756	0.596942416	0.999994594	0.94442346	Soltu.DM.02G030340/Soltu.DM.11G004950	2
1479	GO:2000756	regulation of peptidyl-lysine acetylation	21/12756	0.596942416	0.999994594	0.94442346	Soltu.DM.08G022190/Soltu.DM.04G038280	2
1480	GO:0018209	peptidyl-serine modification	100/12756	0.597846991	0.999994594	0.94442346	Soltu.DM.06G002800/Soltu.DM.05G023210/Soltu.DM.06G032750/Soltu.DM.06G010880/Soltu.DM.03G021780/Soltu.DM.02G023840/Soltu.DM.07G026270/Soltu.DM.03G016650/Soltu.DM.11G004950	9
1481	GO:0051656	establishment of organelle localization	100/12756	0.597846991	0.999994594	0.94442346	Soltu.DM.06G022970/Soltu.DM.09G018720/Soltu.DM.12G024350/Soltu.DM.09G005370/Soltu.DM.11G024760/Soltu.DM.11G026460/Soltu.DM.01G043730/Soltu.DM.10G001460/Soltu.DM.07G006510	9
1482	GO:0050767	regulation of neurogenesis	44/12756	0.598772608	0.999994594	0.94442346	Soltu.DM.12G020370/Soltu.DM.12G020350/Soltu.DM.12G025260/Soltu.DM.12G020340	4
1483	GO:0006405	RNA export from nucleus	78/12756	0.601982273	0.999994594	0.94442346	Soltu.DM.12G005490/Soltu.DM.03G003730/Soltu.DM.12G024350/Soltu.DM.09G005370/Soltu.DM.11G024760/Soltu.DM.04G011330/Soltu.DM.07G006510	7
1484	GO:0016036	cellular response to phosphate starvation	78/12756	0.601982273	0.999994594	0.94442346	Soltu.DM.01G049900/Soltu.DM.01G049890/Soltu.DM.04G001370/Soltu.DM.04G031580/Soltu.DM.11G007630/Soltu.DM.05G011970/Soltu.DM.10G002820	7
1485	GO:0071370	cellular response to gibberellin stimulus	78/12756	0.601982273	0.999994594	0.94442346	Soltu.DM.02G011120/Soltu.DM.09G022610/Soltu.DM.06G012790/Soltu.DM.08G011890/Soltu.DM.01G024340/Soltu.DM.06G002140/Soltu.DM.06G023440	7
1486	GO:0031400	negative regulation of protein modification process	67/12756	0.605288141	0.999994594	0.94442346	Soltu.DM.08G022190/Soltu.DM.07G012130/Soltu.DM.11G010230/Soltu.DM.11G010220/Soltu.DM.06G028580/Soltu.DM.04G038280	6
1487	GO:0009896	positive regulation of catabolic process	123/12756	0.606742844	0.999994594	0.94442346	Soltu.DM.05G004270/Soltu.DM.08G014180/Soltu.DM.12G005510/Soltu.DM.06G018840/Soltu.DM.08G013620/Soltu.DM.05G026810/Soltu.DM.06G015770/Soltu.DM.04G034740/Soltu.DM.09G005140/Soltu.DM.06G024530/Soltu.DM.04G031030	11
1488	GO:0045926	negative regulation of growth	123/12756	0.606742844	0.999994594	0.94442346	Soltu.DM.04G011110/Soltu.DM.01G024670/Soltu.DM.01G039760/Soltu.DM.04G033430/Soltu.DM.01G039230/Soltu.DM.10G027470/Soltu.DM.04G011320/Soltu.DM.04G011240/Soltu.DM.10G028070/Soltu.DM.04G011370/Soltu.DM.11G021090	11
1489	GO:0048469	cell maturation	123/12756	0.606742844	0.999994594	0.94442346	Soltu.DM.08G027650/Soltu.DM.04G001370/Soltu.DM.12G004060/Soltu.DM.12G005490/Soltu.DM.08G023170/Soltu.DM.12G024710/Soltu.DM.09G026810/Soltu.DM.03G001740/Soltu.DM.01G042120/Soltu.DM.03G018740/Soltu.DM.04G002690	11
1490	GO:0031647	regulation of protein stability	33/12756	0.607199931	0.999994594	0.94442346	Soltu.DM.03G030480/Soltu.DM.04G037380/Soltu.DM.04G037150	3
1491	GO:0034765	regulation of monoatomic ion transmembrane transport	33/12756	0.607199931	0.999994594	0.94442346	Soltu.DM.02G030340/Soltu.DM.07G024250/Soltu.DM.11G004950	3
1492	GO:0051495	positive regulation of cytoskeleton organization	33/12756	0.607199931	0.999994594	0.94442346	Soltu.DM.03G003730/Soltu.DM.09G015150/Soltu.DM.09G027230	3
1493	GO:2000070	regulation of response to water deprivation	33/1275	0.6071999	0.9999945	0.9444234	Soltu.DM.06G017300/Soltu.DM.07G019630/Soltu.DM.09G023300	3

			6	31	94	6		
1494	GO:0009741	response to brassinosteroid	112/12756	0.608184387	0.999994594	0.94442346	Soltu.DM.08G022190/Soltu.DM.03G017780/Soltu.DM.03G017800/Soltu.DM.12G024720/Soltu.DM.04G030010/Soltu.DM.08G011890/Soltu.DM.11G011390/Soltu.DM.04G029270/Soltu.DM.04G030040/Soltu.DM.04G021540	10
1495	GO:0016236	macroautophagy	56/12756	0.609974523	0.999994594	0.94442346	Soltu.DM.01G039130/Soltu.DM.11G022310/Soltu.DM.09G025980/Soltu.DM.10G011110/Soltu.DM.02G030830	5
1496	GO:0071470	cellular response to osmotic stress	56/12756	0.609974523	0.999994594	0.94442346	Soltu.DM.02G020550/Soltu.DM.07G020410/Soltu.DM.08G028440/Soltu.DM.03G022850/Soltu.DM.01G008180	5
1497	GO:0050657	nucleic acid transport	79/12756	0.615697662	0.999994594	0.94442346	Soltu.DM.12G005490/Soltu.DM.03G003730/Soltu.DM.12G024350/Soltu.DM.09G005370/Soltu.DM.11G024760/Soltu.DM.04G011330/Soltu.DM.07G006510	7
1498	GO:0050658	RNA transport	79/12756	0.615697662	0.999994594	0.94442346	Soltu.DM.12G005490/Soltu.DM.03G003730/Soltu.DM.12G024350/Soltu.DM.09G005370/Soltu.DM.11G024760/Soltu.DM.04G011330/Soltu.DM.07G006510	7
1499	GO:0051236	establishment of RNA localization	79/12756	0.615697662	0.999994594	0.94442346	Soltu.DM.12G005490/Soltu.DM.03G003730/Soltu.DM.12G024350/Soltu.DM.09G005370/Soltu.DM.11G024760/Soltu.DM.04G011330/Soltu.DM.07G006510	7
1500	GO:0002833	positive regulation of response to biotic stimulus	135/12756	0.616156222	0.999994594	0.94442346	Soltu.DM.02G025590/Soltu.DM.07G028550/Soltu.DM.01G024680/Soltu.DM.10G000640/Soltu.DM.04G018070/Soltu.DM.08G011890/Soltu.DM.02G022460/Soltu.DM.01G051770/Soltu.DM.03G008510/Soltu.DM.08G020460/Soltu.DM.04G000670/Soltu.DM.02G026820	12
1501	GO:0006275	regulation of DNA replication	45/12756	0.61689134	0.999994594	0.94442346	Soltu.DM.11G009630/Soltu.DM.08G027160/Soltu.DM.03G032560/Soltu.DM.12G030150	4
1502	GO:0006352	DNA-templated transcription initiation	45/12756	0.61689134	0.999994594	0.94442346	Soltu.DM.06G028040/Soltu.DM.03G022850/Soltu.DM.04G038280/Soltu.DM.11G015370	4
1503	GO:0006885	regulation of pH	45/12756	0.61689134	0.999994594	0.94442346	Soltu.DM.02G024200/Soltu.DM.11G000570/Soltu.DM.07G009580/Soltu.DM.08G001690	4
1504	GO:0009850	auxin metabolic process	68/12756	0.620001787	0.999994594	0.94442346	Soltu.DM.11G024450/Soltu.DM.01G038470/Soltu.DM.03G035070/Soltu.DM.02G011120/Soltu.DM.06G019760/Soltu.DM.03G035080	6
1505	GO:0032386	regulation of intracellular transport	68/12756	0.620001787	0.999994594	0.94442346	Soltu.DM.12G020370/Soltu.DM.12G020350/Soltu.DM.07G002400/Soltu.DM.08G001690/Soltu.DM.12G020340/Soltu.DM.02G030830	6
1506	GO:0000466	maturation of 5.8S rRNA from tricistronic rRNA transcript (SSU-rRNA, 5.8S rRNA, LSU-rRNA)	22/12756	0.622740013	0.999994594	0.94442346	Soltu.DM.12G024350/Soltu.DM.01G051600	2
1507	GO:0000478	endonucleolytic cleavage involved in rRNA processing	22/12756	0.622740013	0.999994594	0.94442346	Soltu.DM.12G024350/Soltu.DM.01G051600	2
1508	GO:0000720	pyrimidine dimer repair by nucleotide-excision repair	22/12756	0.622740013	0.999994594	0.94442346	Soltu.DM.07G017530/Soltu.DM.05G018370	2
1509	GO:0001817	regulation of cytokine production	22/12756	0.622740013	0.999994594	0.94442346	Soltu.DM.08G020460/Soltu.DM.01G002690	2
1510	GO:0006596	polyamine biosynthetic process	22/12756	0.622740013	0.999994594	0.94442346	Soltu.DM.06G014480/Soltu.DM.01G050280	2
1511	GO:0007602	phototransduction	22/ 12	0.6 227	0.9 999	0.9 444	Soltu.DM.07G015980/Soltu.DM.02G011380	2

			75 6	400 13	945 94	234 6		
15 12	GO:00 09585	red, far-red light phototransduction	22/ 12 75 6	0.6 227 400 13	0.9 999 945 94	0.9 444 234 6	Soltu.DM.07G015980/Soltu.DM.02G011380	2
15 13	GO:00 30150	protein import into mitochondrial matrix	22/ 12 75 6	0.6 227 400 13	0.9 999 945 94	0.9 444 234 6	Soltu.DM.04G037150/Soltu.DM.12G021450	2
15 14	GO:00 30838	positive regulation of actin filament polymerization	22/ 12 75 6	0.6 227 400 13	0.9 999 945 94	0.9 444 234 6	Soltu.DM.09G015150/Soltu.DM.09G027230	2
15 15	GO:00 48579	negative regulation of long-day photoperiodism, flowering	22/ 12 75 6	0.6 227 400 13	0.9 999 945 94	0.9 444 234 6	Soltu.DM.04G006870/Soltu.DM.01G024340	2
15 16	GO:00 51017	actin filament bundle assembly	22/ 12 75 6	0.6 227 400 13	0.9 999 945 94	0.9 444 234 6	Soltu.DM.07G026780/Soltu.DM.01G028770	2
15 17	GO:00 61572	actin filament bundle organization	22/ 12 75 6	0.6 227 400 13	0.9 999 945 94	0.9 444 234 6	Soltu.DM.07G026780/Soltu.DM.01G028770	2
15 18	GO:00 00103	sulfate assimilation	10/ 12 75 6	0.6 252 188 69	0.9 999 945 94	0.9 444 234 6	Soltu.DM.03G000340	1
15 19	GO:00 00303	response to superoxide	10/ 12 75 6	0.6 252 188 69	0.9 999 945 94	0.9 444 234 6	Soltu.DM.06G012170	1
15 20	GO:00 00394	RNA splicing, via endonucleolytic cleavage and ligation	10/ 12 75 6	0.6 252 188 69	0.9 999 945 94	0.9 444 234 6	Soltu.DM.01G006380	1
15 21	GO:00 01655	urogenital system development	10/ 12 75 6	0.6 252 188 69	0.9 999 945 94	0.9 444 234 6	Soltu.DM.10G022360	1
15 22	GO:00 06023	aminoglycan biosynthetic process	10/ 12 75 6	0.6 252 188 69	0.9 999 945 94	0.9 444 234 6	Soltu.DM.02G031050	1
15 23	GO:00 06024	glycosaminoglycan biosynthetic process	10/ 12 75 6	0.6 252 188 69	0.9 999 945 94	0.9 444 234 6	Soltu.DM.02G031050	1
15 24	GO:00 06145	purine nucleobase catabolic process	10/ 12 75 6	0.6 252 188 69	0.9 999 945 94	0.9 444 234 6	Soltu.DM.02G017810	1
15 25	GO:00 06474	N-terminal protein amino acid acetylation	10/ 12 75 6	0.6 252 188 69	0.9 999 945 94	0.9 444 234 6	Soltu.DM.06G019850	1
15 26	GO:00 06547	histidine metabolic process	10/ 12 75 6	0.6 252 188 69	0.9 999 945 94	0.9 444 234 6	Soltu.DM.07G019380	1
15 27	GO:00 06903	vesicle targeting	10/ 12 75 6	0.6 252 188 69	0.9 999 945 94	0.9 444 234 6	Soltu.DM.11G026460	1
15 28	GO:00 08406	gonad development	10/ 12 75 6	0.6 252 188 69	0.9 999 945 94	0.9 444 234 6	Soltu.DM.12G019270	1
15	GO:00	pentose-phosphate shunt, oxidative	10/	0.6	0.9	0.9	Soltu.DM.08G014620	1

29	09051	branch	12756	25218869	99994594	4442346		
1530	GO:0009098	leucine biosynthetic process	10/12756	0.625218869	0.999994594	0.944423446	Soltu.DM.06G018090	1
1531	GO:0009164	nucleoside catabolic process	10/12756	0.625218869	0.999994594	0.944423446	Soltu.DM.12G003790	1
1532	GO:0009445	putrescine metabolic process	10/12756	0.625218869	0.999994594	0.944423446	Soltu.DM.01G050280	1
1533	GO:0009446	putrescine biosynthetic process	10/12756	0.625218869	0.999994594	0.944423446	Soltu.DM.01G050280	1
1534	GO:0009697	salicylic acid biosynthetic process	10/12756	0.625218869	0.999994594	0.944423446	Soltu.DM.09G023400	1
1535	GO:0010962	regulation of glucan biosynthetic process	10/12756	0.625218869	0.999994594	0.944423446	Soltu.DM.08G008380	1
1536	GO:0015791	polyol transmembrane transport	10/12756	0.625218869	0.999994594	0.944423446	Soltu.DM.03G031200	1
1537	GO:0015936	coenzyme A metabolic process	10/12756	0.625218869	0.999994594	0.944423446	Soltu.DM.11G010590	1
1538	GO:0015937	coenzyme A biosynthetic process	10/12756	0.625218869	0.999994594	0.944423446	Soltu.DM.11G010590	1
1539	GO:0019233	sensory perception of pain	10/12756	0.625218869	0.999994594	0.944423446	Soltu.DM.02G017970	1
1540	GO:0019343	cysteine biosynthetic process via cystathionine	10/12756	0.625218869	0.999994594	0.944423446	Soltu.DM.08G030020	1
1541	GO:0019346	transsulfuration	10/12756	0.625218869	0.999994594	0.944423446	Soltu.DM.08G030020	1
1542	GO:0031445	regulation of heterochromatin formation	10/12756	0.625218869	0.999994594	0.944423446	Soltu.DM.03G020440	1
1543	GO:0032212	positive regulation of telomere maintenance via telomerase	10/12756	0.625218869	0.999994594	0.944423446	Soltu.DM.02G013390	1
1544	GO:0032885	regulation of polysaccharide biosynthetic process	10/12756	0.625218869	0.999994594	0.944423446	Soltu.DM.08G008380	1
1545	GO:0033059	cellular pigmentation	10/12756	0.625218869	0.999994594	0.944423446	Soltu.DM.06G005370	1
1546	GO:0035082	axoneme assembly	10/12756	0.625218869	0.999994594	0.944423446	Soltu.DM.01G020880	1

15 47	GO:00 35246	peptidyl-arginine N-methylation	10/ 12 75 6	0.6 252 188 69	0.9 999 945 94	0.9 444 234 6	Soltu.DM.06G017810	1
15 48	GO:00 35434	copper ion transmembrane transport	10/ 12 75 6	0.6 252 188 69	0.9 999 945 94	0.9 444 234 6	Soltu.DM.08G012080	1
15 49	GO:00 42304	regulation of fatty acid biosynthetic process	10/ 12 75 6	0.6 252 188 69	0.9 999 945 94	0.9 444 234 6	Soltu.DM.07G024240	1
15 50	GO:00 42454	ribonucleoside catabolic process	10/ 12 75 6	0.6 252 188 69	0.9 999 945 94	0.9 444 234 6	Soltu.DM.12G003790	1
15 51	GO:00 43482	cellular pigment accumulation	10/ 12 75 6	0.6 252 188 69	0.9 999 945 94	0.9 444 234 6	Soltu.DM.06G005370	1
15 52	GO:00 43981	histone H4-K5 acetylation	10/ 12 75 6	0.6 252 188 69	0.9 999 945 94	0.9 444 234 6	Soltu.DM.09G019870	1
15 53	GO:00 45137	development of primary sexual characteristics	10/ 12 75 6	0.6 252 188 69	0.9 999 945 94	0.9 444 234 6	Soltu.DM.12G019270	1
15 54	GO:00 45833	negative regulation of lipid metabolic process	10/ 12 75 6	0.6 252 188 69	0.9 999 945 94	0.9 444 234 6	Soltu.DM.08G029860	1
15 55	GO:00 46471	phosphatidylglycerol metabolic process	10/ 12 75 6	0.6 252 188 69	0.9 999 945 94	0.9 444 234 6	Soltu.DM.07G026100	1
15 56	GO:00 48066	developmental pigmentation	10/ 12 75 6	0.6 252 188 69	0.9 999 945 94	0.9 444 234 6	Soltu.DM.06G005370	1
15 57	GO:00 50931	pigment cell differentiation	10/ 12 75 6	0.6 252 188 69	0.9 999 945 94	0.9 444 234 6	Soltu.DM.06G005370	1
15 58	GO:00 51214	RNAi-mediated antiviral immunity against RNA virus	10/ 12 75 6	0.6 252 188 69	0.9 999 945 94	0.9 444 234 6	Soltu.DM.11G004150	1
15 59	GO:00 52803	imidazole-containing compound metabolic process	10/ 12 75 6	0.6 252 188 69	0.9 999 945 94	0.9 444 234 6	Soltu.DM.07G019380	1
15 60	GO:00 61615	glycolytic process through fructose-6-phosphate	10/ 12 75 6	0.6 252 188 69	0.9 999 945 94	0.9 444 234 6	Soltu.DM.12G004480	1
15 61	GO:00 61912	selective autophagy	10/ 12 75 6	0.6 252 188 69	0.9 999 945 94	0.9 444 234 6	Soltu.DM.02G030830	1
15 62	GO:00 71266	'de novo' L-methionine biosynthetic process	10/ 12 75 6	0.6 252 188 69	0.9 999 945 94	0.9 444 234 6	Soltu.DM.08G030020	1
15 63	GO:00 71421	manganese ion transmembrane transport	10/ 12 75 6	0.6 252 188 69	0.9 999 945 94	0.9 444 234 6	Soltu.DM.04G003430	1
15 64	GO:00 80060	integument development	10/ 12 75	0.6 252 188	0.9 999 945	0.9 444 234	Soltu.DM.02G003130	1

			6	69	94	6		
1565	GO:0120261	regulation of heterochromatin organization	10/12756	0.625218869	0.999994594	0.94442346	Soltu.DM.03G020440	1
1566	GO:1900034	regulation of cellular response to heat	10/12756	0.625218869	0.999994594	0.94442346	Soltu.DM.07G019630	1
1567	GO:1904358	positive regulation of telomere maintenance via telomere lengthening	10/12756	0.625218869	0.999994594	0.94442346	Soltu.DM.02G013390	1
1568	GO:1905177	tracheary element differentiation	10/12756	0.625218869	0.999994594	0.94442346	Soltu.DM.11G011390	1
1569	GO:1990573	potassium ion import across plasma membrane	10/12756	0.625218869	0.999994594	0.94442346	Soltu.DM.12G024710	1
1570	GO:2000114	regulation of establishment of cell polarity	10/12756	0.625218869	0.999994594	0.94442346	Soltu.DM.01G042120	1
1571	GO:0043269	regulation of monoatomic ion transport	57/12756	0.625939963	0.999994594	0.94442346	Soltu.DM.02G020550/Soltu.DM.05G011970/Soltu.DM.02G030340/Soltu.DM.07G024250/Soltu.DM.11G004950	5
1572	GO:0006006	glucose metabolic process	34/12756	0.627809268	0.999994594	0.94442346	Soltu.DM.02G031920/Soltu.DM.04G031580/Soltu.DM.02G031890	3
1573	GO:0008645	hexose transmembrane transport	34/12756	0.627809268	0.999994594	0.94442346	Soltu.DM.09G024150/Soltu.DM.02G006700/Soltu.DM.02G018010	3
1574	GO:0010975	regulation of neuron projection development	34/12756	0.627809268	0.999994594	0.94442346	Soltu.DM.12G020370/Soltu.DM.12G020350/Soltu.DM.12G020340	3
1575	GO:0016575	histone deacetylation	34/12756	0.627809268	0.999994594	0.94442346	Soltu.DM.04G033160/Soltu.DM.02G001620/Soltu.DM.02G001630	3
1576	GO:0034762	regulation of transmembrane transport	34/12756	0.627809268	0.999994594	0.94442346	Soltu.DM.02G030340/Soltu.DM.07G024250/Soltu.DM.11G004950	3
1577	GO:0046323	glucose import	34/12756	0.627809268	0.999994594	0.94442346	Soltu.DM.09G024150/Soltu.DM.02G006700/Soltu.DM.02G018010	3
1578	GO:1904659	glucose transmembrane transport	34/12756	0.627809268	0.999994594	0.94442346	Soltu.DM.09G024150/Soltu.DM.02G006700/Soltu.DM.02G018010	3
1579	GO:0043067	regulation of programmed cell death	125/12756	0.628506696	0.999994594	0.94442346	Soltu.DM.09G024260/Soltu.DM.02G018520/Soltu.DM.08G014180/Soltu.DM.05G022160/Soltu.DM.08G022900/Soltu.DM.08G028440/Soltu.DM.01G044520/Soltu.DM.04G022240/Soltu.DM.07G014680/Soltu.DM.09G024270/Soltu.DM.06G024530	11
1580	GO:0009069	serine family amino acid metabolic process	46/12756	0.634489889	0.999994594	0.94442346	Soltu.DM.08G013400/Soltu.DM.08G030020/Soltu.DM.12G002620/Soltu.DM.12G025770	4
1581	GO:0032102	negative regulation of response to external stimulus	46/12756	0.634489889	0.999994594	0.94442346	Soltu.DM.08G022900/Soltu.DM.06G015770/Soltu.DM.02G023840/Soltu.DM.05G023030	4
1582	GO:0043200	response to amino acid	46/12	0.6344	0.9999	0.9444	Soltu.DM.02G024660/Soltu.DM.10G000560/Soltu.DM.01G051900/Soltu.DM.02G022460	4

			756	89889	94594	2346		
1583	GO:0043401	steroid hormone mediated signaling pathway	46/12756	0.634489889	0.999945994	0.94442346	Soltu.DM.04G038280/Soltu.DM.08G011890/Soltu.DM.04G029270/Soltu.DM.04G021540	4
1584	GO:0071383	cellular response to steroid hormone stimulus	46/12756	0.634489889	0.999945994	0.94442346	Soltu.DM.04G038280/Soltu.DM.08G011890/Soltu.DM.04G029270/Soltu.DM.04G021540	4
1585	GO:0016052	carbohydrate catabolic process	137/12756	0.636786134	0.999945994	0.94442346	Soltu.DM.09G027770/Soltu.DM.12G004480/Soltu.DM.05G006330/Soltu.DM.01G018690/Soltu.DM.01G040550/Soltu.DM.02G031920/Soltu.DM.02G031890/Soltu.DM.11G000740/Soltu.DM.12G007130/Soltu.DM.04G037250/Soltu.DM.06G013720/Soltu.DM.06G020260	12
1586	GO:0071446	cellular response to salicylic acid stimulus	58/12756	0.641505393	0.999945994	0.94442346	Soltu.DM.09G018910/Soltu.DM.09G029050/Soltu.DM.02G006310/Soltu.DM.04G028540/Soltu.DM.07G024240	5
1587	GO:0031503	protein-containing complex localization	81/12756	0.642339277	0.999945994	0.94442346	Soltu.DM.12G005490/Soltu.DM.03G003730/Soltu.DM.12G024350/Soltu.DM.09G005370/Soltu.DM.11G024760/Soltu.DM.04G011330/Soltu.DM.07G006510	7
1588	GO:0034249	negative regulation of amide metabolic process	81/12756	0.642339277	0.999945994	0.94442346	Soltu.DM.05G004270/Soltu.DM.08G029860/Soltu.DM.04G001110/Soltu.DM.08G013620/Soltu.DM.05G006430/Soltu.DM.05G026810/Soltu.DM.04G031030	7
1589	GO:0006084	acetyl-CoA metabolic process	23/12756	0.647240465	0.999945994	0.94442346	Soltu.DM.08G026810/Soltu.DM.01G019520	2
1590	GO:0006221	pyrimidine nucleotide biosynthetic process	23/12756	0.647240465	0.999945994	0.94442346	Soltu.DM.11G007630/Soltu.DM.01G017170	2
1591	GO:0006536	glutamate metabolic process	23/12756	0.647240465	0.999945994	0.94442346	Soltu.DM.12G024030/Soltu.DM.08G007450	2
1592	GO:0009083	branched-chain amino acid catabolic process	23/12756	0.647240465	0.999945994	0.94442346	Soltu.DM.04G025250/Soltu.DM.01G047450	2
1593	GO:0009616	RNAi-mediated antiviral immune response	23/12756	0.647240465	0.999945994	0.94442346	Soltu.DM.01G010020/Soltu.DM.11G004150	2
1594	GO:0010048	vernalization response	23/12756	0.647240465	0.999945994	0.94442346	Soltu.DM.02G002210/Soltu.DM.05G012040	2
1595	GO:0010143	cutin biosynthetic process	23/12756	0.647240465	0.999945994	0.94442346	Soltu.DM.07G022050/Soltu.DM.01G033720	2
1596	GO:0030497	fatty acid elongation	23/12756	0.647240465	0.999945994	0.94442346	Soltu.DM.04G008710/Soltu.DM.10G026480	2
1597	GO:0051304	chromosome separation	23/12756	0.647240465	0.999945994	0.94442346	Soltu.DM.02G001620/Soltu.DM.02G001630	2
1598	GO:1901661	quinone metabolic process	23/12756	0.647240465	0.999945994	0.94442346	Soltu.DM.06G032850/Soltu.DM.06G032860	2
1599	GO:1901663	quinone biosynthetic process	23/12756	0.647240465	0.999945994	0.94442346	Soltu.DM.06G032850/Soltu.DM.06G032860	2
16	GO:00	mRNA polyadenylation	35/	0.6	0.9	0.9	Soltu.DM.01G006380/Soltu.DM.03G003370/Soltu.DM.03G019650	3

00	06378		12756	47660154	99994594	4442346		
1601	GO:0015749	monosaccharide transmembrane transport	35/12756	0.647660154	0.999994594	0.94442346	Soltu.DM.09G024150/Soltu.DM.02G006700/Soltu.DM.02G018010	3
1602	GO:0046164	alcohol catabolic process	35/12756	0.647660154	0.999994594	0.94442346	Soltu.DM.08G020150/Soltu.DM.07G013940/Soltu.DM.07G013900	3
1603	GO:0010051	xylem and phloem pattern formation	70/12756	0.648469347	0.999994594	0.94442346	Soltu.DM.12G005490/Soltu.DM.09G020190/Soltu.DM.04G029270/Soltu.DM.03G024040/Soltu.DM.04G021540/Soltu.DM.04G031030	6
1604	GO:0010380	regulation of chlorophyll biosynthetic process	93/12756	0.649827772	0.999994594	0.94442346	Soltu.DM.04G011110/Soltu.DM.09G025070/Soltu.DM.10G027470/Soltu.DM.04G011320/Soltu.DM.04G011240/Soltu.DM.10G028070/Soltu.DM.04G011370/Soltu.DM.11G021090	8
1605	GO:0009749	response to glucose	47/12756	0.651556551	0.999994594	0.94442346	Soltu.DM.07G013360/Soltu.DM.08G008380/Soltu.DM.04G024100/Soltu.DM.08G011890	4
1606	GO:0010942	positive regulation of cell death	47/12756	0.651556551	0.999994594	0.94442346	Soltu.DM.06G026400/Soltu.DM.01G044520/Soltu.DM.07G014680/Soltu.DM.07G024240	4
1607	GO:0044247	cellular polysaccharide catabolic process	47/12756	0.651556551	0.999994594	0.94442346	Soltu.DM.09G027770/Soltu.DM.05G006330/Soltu.DM.11G000740/Soltu.DM.04G037250	4
1608	GO:0048545	response to steroid hormone	47/12756	0.651556551	0.999994594	0.94442346	Soltu.DM.04G038280/Soltu.DM.08G011890/Soltu.DM.04G029270/Soltu.DM.04G021540	4
1609	GO:0080036	regulation of cytokinin-activated signaling pathway	47/12756	0.651556551	0.999994594	0.94442346	Soltu.DM.03G034960/Soltu.DM.04G011320/Soltu.DM.04G011240/Soltu.DM.04G011370	4
1610	GO:1900424	regulation of defense response to bacterium	47/12756	0.651556551	0.999994594	0.94442346	Soltu.DM.06G015770/Soltu.DM.02G023840/Soltu.DM.04G000670/Soltu.DM.02G026820	4
1611	GO:0006066	alcohol metabolic process	150/12756	0.654175838	0.999994594	0.94442346	Soltu.DM.02G018520/Soltu.DM.08G020150/Soltu.DM.11G024450/Soltu.DM.08G014180/Soltu.DM.07G013940/Soltu.DM.03G018850/Soltu.DM.07G017750/Soltu.DM.12G024030/Soltu.DM.06G029640/Soltu.DM.07G013900/Soltu.DM.03G008510/Soltu.DM.06G003240/Soltu.DM.10G022710	13
1612	GO:0002218	activation of innate immune response	82/12756	0.655249127	0.999994594	0.94442346	Soltu.DM.02G025590/Soltu.DM.07G028550/Soltu.DM.10G000640/Soltu.DM.04G018070/Soltu.DM.08G011890/Soltu.DM.02G022460/Soltu.DM.01G051770	7
1613	GO:0051168	nuclear export	82/12756	0.655249127	0.999994594	0.94442346	Soltu.DM.12G005490/Soltu.DM.03G003730/Soltu.DM.12G024350/Soltu.DM.09G005370/Soltu.DM.11G024760/Soltu.DM.04G011330/Soltu.DM.07G006510	7
1614	GO:0071417	cellular response to organonitrogen compound	59/12756	0.656660992	0.999994594	0.94442346	Soltu.DM.01G008040/Soltu.DM.03G035710/Soltu.DM.01G051900/Soltu.DM.02G022460/Soltu.DM.05G001260	5
1615	GO:0032103	positive regulation of response to external stimulus	139/12756	0.656827521	0.999994594	0.94442346	Soltu.DM.02G025590/Soltu.DM.07G028550/Soltu.DM.01G024680/Soltu.DM.10G000640/Soltu.DM.04G018070/Soltu.DM.08G011890/Soltu.DM.02G022460/Soltu.DM.01G051770/Soltu.DM.03G008510/Soltu.DM.08G020460/Soltu.DM.04G000670/Soltu.DM.02G026820	12
1616	GO:0001824	blastocyst development	11/12756	0.660268227	0.999994594	0.94442346	Soltu.DM.02G029740	1
1617	GO:0006098	pentose-phosphate shunt	11/12756	0.660268227	0.999994594	0.94442346	Soltu.DM.08G014620	1

1618	GO:0006376	mRNA splice site selection	11/12756	0.660268227	0.999994594	0.94442346	Soltu.DM.10G001400	1
1619	GO:0006541	glutamine metabolic process	11/12756	0.660268227	0.999994594	0.94442346	Soltu.DM.12G024030	1
1620	GO:0006544	glycine metabolic process	11/12756	0.660268227	0.999994594	0.94442346	Soltu.DM.08G013400	1
1621	GO:0008216	spermidine metabolic process	11/12756	0.660268227	0.999994594	0.94442346	Soltu.DM.06G014480	1
1622	GO:0008295	spermidine biosynthetic process	11/12756	0.660268227	0.999994594	0.94442346	Soltu.DM.06G014480	1
1623	GO:0009855	determination of bilateral symmetry	11/12756	0.660268227	0.999994594	0.94442346	Soltu.DM.02G003130	1
1624	GO:0010019	chloroplast-nucleus signaling pathway	11/12756	0.660268227	0.999994594	0.94442346	Soltu.DM.12G026560	1
1625	GO:0010586	miRNA metabolic process	11/12756	0.660268227	0.999994594	0.94442346	Soltu.DM.08G013620	1
1626	GO:0030186	melatonin metabolic process	11/12756	0.660268227	0.999994594	0.94442346	Soltu.DM.12G026120	1
1627	GO:0030187	melatonin biosynthetic process	11/12756	0.660268227	0.999994594	0.94442346	Soltu.DM.12G026120	1
1628	GO:0030203	glycosaminoglycan metabolic process	11/12756	0.660268227	0.999994594	0.94442346	Soltu.DM.02G031050	1
1629	GO:0031054	pre-miRNA processing	11/12756	0.660268227	0.999994594	0.94442346	Soltu.DM.04G031030	1
1630	GO:0031152	aggregation involved in sorocarp development	11/12756	0.660268227	0.999994594	0.94442346	Soltu.DM.01G028770	1
1631	GO:0032527	protein exit from endoplasmic reticulum	11/12756	0.660268227	0.999994594	0.94442346	Soltu.DM.10G004220	1
1632	GO:0033169	histone H3-K9 demethylation	11/12756	0.660268227	0.999994594	0.94442346	Soltu.DM.08G022190	1
1633	GO:0034969	histone arginine methylation	11/12756	0.660268227	0.999994594	0.94442346	Soltu.DM.06G017810	1
1634	GO:0035265	organ growth	11/12756	0.660268227	0.999994594	0.94442346	Soltu.DM.10G022360	1
1635	GO:0035510	DNA dealkylation	11/1275	0.6602682	0.9999945	0.9444234	Soltu.DM.10G024770	1

			6	27	94	6		
1636	GO:0036473	cell death in response to oxidative stress	11/12756	0.660268227	0.999994594	0.94442346	Soltu.DM.06G026560	1
1637	GO:0043393	regulation of protein binding	11/12756	0.660268227	0.999994594	0.94442346	Soltu.DM.01G039760	1
1638	GO:0044818	mitotic G2/M transition checkpoint	11/12756	0.660268227	0.999994594	0.94442346	Soltu.DM.02G033290	1
1639	GO:0048732	gland development	11/12756	0.660268227	0.999994594	0.94442346	Soltu.DM.10G022360	1
1640	GO:0050667	homocysteine metabolic process	11/12756	0.660268227	0.999994594	0.94442346	Soltu.DM.08G030020	1
1641	GO:0090090	negative regulation of canonical Wnt signaling pathway	11/12756	0.660268227	0.999994594	0.94442346	Soltu.DM.07G002580	1
1642	GO:0090376	seed trichome differentiation	11/12756	0.660268227	0.999994594	0.94442346	Soltu.DM.05G005120	1
1643	GO:0090378	seed trichome elongation	11/12756	0.660268227	0.999994594	0.94442346	Soltu.DM.05G005120	1
1644	GO:0097468	programmed cell death in response to reactive oxygen species	11/12756	0.660268227	0.999994594	0.94442346	Soltu.DM.06G026560	1
1645	GO:0098609	cell-cell adhesion	11/12756	0.660268227	0.999994594	0.94442346	Soltu.DM.12G024030	1
1646	GO:0098630	aggregation of unicellular organisms	11/12756	0.660268227	0.999994594	0.94442346	Soltu.DM.01G028770	1
1647	GO:0098743	cell aggregation	11/12756	0.660268227	0.999994594	0.94442346	Soltu.DM.01G028770	1
1648	GO:1900458	negative regulation of brassinosteroid mediated signaling pathway	11/12756	0.660268227	0.999994594	0.94442346	Soltu.DM.07G021000	1
1649	GO:0046467	membrane lipid biosynthetic process	94/12756	0.661800959	0.999994594	0.94442346	Soltu.DM.08G001900/Soltu.DM.04G008710/Soltu.DM.02G018520/Soltu.DM.08G014180/Soltu.DM.01G047750/Soltu.DM.07G015200/Soltu.DM.02G010490/Soltu.DM.10G020020	8
1650	GO:1901463	regulation of tetrapyrrole biosynthetic process	94/12756	0.661800959	0.999994594	0.94442346	Soltu.DM.04G011110/Soltu.DM.09G025070/Soltu.DM.10G027470/Soltu.DM.04G011320/Soltu.DM.04G011240/Soltu.DM.10G028070/Soltu.DM.04G011370/Soltu.DM.11G021090	8
1651	GO:0043934	sporulation	71/12756	0.662205731	0.999994594	0.94442346	Soltu.DM.06G009270/Soltu.DM.09G031520/Soltu.DM.05G022790/Soltu.DM.02G011180/Soltu.DM.09G031510/Soltu.DM.12G023840	6
1652	GO:1901699	cellular response to nitrogen compound	196/12756	0.663627329	0.999994594	0.94442346	Soltu.DM.01G008040/Soltu.DM.03G035710/Soltu.DM.07G028550/Soltu.DM.09G024260/Soltu.DM.01G051900/Soltu.DM.10G000640/Soltu.DM.02G030410/Soltu.DM.12G005490/Soltu.DM.09G024860/Soltu.DM.12G026070/Soltu.DM.02G022460/Soltu.DM.11G025410/Soltu.DM.05G001260/Soltu.DM.09G024270/Soltu.DM.01G010020/Soltu.DM.04G031030/Soltu.DM.11G004150	17
16	GO:00	organelle localization	15	0.6	0.9	0.9	Soltu.DM.06G005370/Soltu.DM.03G003730/Soltu.DM.06G022970/Soltu.DM.09G018720/S	13

53	51640		1/12756	63645976	99994594	4442346	oltu.DM.12G024350/Soltu.DM.08G015350/Soltu.DM.09G005370/Soltu.DM.11G024760/Soltu.DM.11G026460/Soltu.DM.01G043730/Soltu.DM.08G007450/Soltu.DM.10G001460/Soltu.DM.07G006510	
1654	GO:0006497	protein lipidation	36/12756	0.666746261	0.999994594	0.94442346	Soltu.DM.01G039130/Soltu.DM.02G010490/Soltu.DM.11G022310	3
1655	GO:0006641	triglyceride metabolic process	36/12756	0.666746261	0.999994594	0.94442346	Soltu.DM.12G002120/Soltu.DM.01G034820/Soltu.DM.10G005430	3
1656	GO:0042158	lipoprotein biosynthetic process	36/12756	0.666746261	0.999994594	0.94442346	Soltu.DM.01G039130/Soltu.DM.02G010490/Soltu.DM.11G022310	3
1657	GO:0043903	regulation of biological process involved in symbiotic interaction	36/12756	0.666746261	0.999994594	0.94442346	Soltu.DM.08G022900/Soltu.DM.09G003770/Soltu.DM.05G026140	3
1658	GO:1902170	cellular response to reactive nitrogen species	36/12756	0.666746261	0.999994594	0.94442346	Soltu.DM.07G028550/Soltu.DM.10G000640/Soltu.DM.02G030410	3
1659	GO:0042435	indole-containing compound biosynthetic process	48/12756	0.668082734	0.999994594	0.94442346	Soltu.DM.12G022190/Soltu.DM.06G018840/Soltu.DM.12G026120/Soltu.DM.07G014750	4
1660	GO:0006650	glycerophospholipid metabolic process	106/12756	0.668115839	0.999994594	0.94442346	Soltu.DM.02G034460/Soltu.DM.04G037130/Soltu.DM.03G030800/Soltu.DM.02G010490/Soltu.DM.04G034770/Soltu.DM.05G001470/Soltu.DM.07G026100/Soltu.DM.06G018040/Soltu.DM.02G019940	9
1661	GO:0009583	detection of light stimulus	24/12756	0.670460733	0.999994594	0.94442346	Soltu.DM.07G015980/Soltu.DM.02G011380	2
1662	GO:0032273	positive regulation of protein polymerization	24/12756	0.670460733	0.999994594	0.94442346	Soltu.DM.09G015150/Soltu.DM.09G027230	2
1663	GO:0035672	oligopeptide transmembrane transport	24/12756	0.670460733	0.999994594	0.94442346	Soltu.DM.08G029920/Soltu.DM.04G031480	2
1664	GO:0042181	ketone biosynthetic process	24/12756	0.670460733	0.999994594	0.94442346	Soltu.DM.06G032850/Soltu.DM.06G032860	2
1665	GO:0043405	regulation of MAP kinase activity	24/12756	0.670460733	0.999994594	0.94442346	Soltu.DM.11G010230/Soltu.DM.11G010220	2
1666	GO:0043473	pigmentation	24/12756	0.670460733	0.999994594	0.94442346	Soltu.DM.06G005370/Soltu.DM.09G025040	2
1667	GO:0072523	purine-containing compound catabolic process	24/12756	0.670460733	0.999994594	0.94442346	Soltu.DM.02G017810/Soltu.DM.12G003790	2
1668	GO:0032147	activation of protein kinase activity	60/12756	0.671398926	0.999994594	0.94442346	Soltu.DM.07G017210/Soltu.DM.07G017190/Soltu.DM.07G017200/Soltu.DM.07G017180/Soltu.DM.02G028740	5
1669	GO:0046916	cellular transition metal ion homeostasis	60/12756	0.671398926	0.999994594	0.94442346	Soltu.DM.07G002440/Soltu.DM.03G017590/Soltu.DM.05G021830/Soltu.DM.04G003430/Soltu.DM.07G009580	5
1670	GO:0051604	protein maturation	60/12756	0.671398926	0.999994594	0.94442346	Soltu.DM.02G020320/Soltu.DM.06G019850/Soltu.DM.08G023320/Soltu.DM.02G020340/Soltu.DM.06G018040	5

1671	GO:0006869	lipid transport	107/12756	0.679093002	0.999994594	0.94442346	Soltu.DM.09G028710/Soltu.DM.08G016090/Soltu.DM.06G005370/Soltu.DM.10G022360/Soltu.DM.06G022490/Soltu.DM.01G034820/Soltu.DM.03G024040/Soltu.DM.11G011430/Soltu.DM.02G019940	9
1672	GO:0009914	hormone transport	130/12756	0.680061494	0.999994594	0.94442346	Soltu.DM.04G030440/Soltu.DM.01G035900/Soltu.DM.02G032050/Soltu.DM.06G012690/Soltu.DM.10G026500/Soltu.DM.01G035910/Soltu.DM.03G036780/Soltu.DM.11G001500/Soltu.DM.08G001470/Soltu.DM.02G022410/Soltu.DM.04G002690	11
1673	GO:0055065	metal ion homeostasis	153/12756	0.682149113	0.999994594	0.94442346	Soltu.DM.07G002440/Soltu.DM.01G035900/Soltu.DM.07G028550/Soltu.DM.03G017590/Soltu.DM.10G010160/Soltu.DM.10G000640/Soltu.DM.05G021830/Soltu.DM.04G003430/Soltu.DM.07G009580/Soltu.DM.07G015200/Soltu.DM.10G004300/Soltu.DM.12G021010/Soltu.DM.01G035910	13
1674	GO:0006754	ATP biosynthetic process	49/12756	0.684062725	0.999994594	0.94442346	Soltu.DM.12G004480/Soltu.DM.07G009580/Soltu.DM.06G013720/Soltu.DM.11G025570	4
1675	GO:0010014	meristem initiation	49/12756	0.684062725	0.999994594	0.94442346	Soltu.DM.02G003130/Soltu.DM.05G026810/Soltu.DM.06G034230/Soltu.DM.03G034800	4
1676	GO:0009845	seed germination	119/12756	0.684538308	0.999994594	0.94442346	Soltu.DM.02G018520/Soltu.DM.06G012350/Soltu.DM.02G022700/Soltu.DM.08G011890/Soltu.DM.02G004510/Soltu.DM.08G011330/Soltu.DM.01G035240/Soltu.DM.10G005360/Soltu.DM.12G028510/Soltu.DM.06G002320	10
1677	GO:0005977	glycogen metabolic process	37/12756	0.685066064	0.999994594	0.94442346	Soltu.DM.01G040570/Soltu.DM.02G024820/Soltu.DM.11G008240	3
1678	GO:0006112	energy reserve metabolic process	37/12756	0.685066064	0.999994594	0.94442346	Soltu.DM.01G040570/Soltu.DM.02G024820/Soltu.DM.11G008240	3
1679	GO:0009742	brassinosteroid mediated signaling pathway	37/12756	0.685066064	0.999994594	0.94442346	Soltu.DM.08G011890/Soltu.DM.04G029270/Soltu.DM.04G021540	3
1680	GO:0043631	RNA polyadenylation	37/12756	0.685066064	0.999994594	0.94442346	Soltu.DM.01G006380/Soltu.DM.03G003370/Soltu.DM.03G019650	3
1681	GO:0002237	response to molecule of bacterial origin	61/12756	0.685713239	0.999994594	0.94442346	Soltu.DM.07G028550/Soltu.DM.10G000640/Soltu.DM.02G006310/Soltu.DM.03G021360/Soltu.DM.07G022640	5
1682	GO:0048443	stamen development	142/12756	0.685716175	0.999994594	0.94442346	Soltu.DM.03G024660/Soltu.DM.03G024680/Soltu.DM.03G037120/Soltu.DM.07G003270/Soltu.DM.03G024670/Soltu.DM.06G023410/Soltu.DM.05G003100/Soltu.DM.03G024690/Soltu.DM.10G000600/Soltu.DM.04G022240/Soltu.DM.03G000400/Soltu.DM.11G001500	12
1683	GO:0048466	androecium development	142/12756	0.685716175	0.999994594	0.94442346	Soltu.DM.03G024660/Soltu.DM.03G024680/Soltu.DM.03G037120/Soltu.DM.07G003270/Soltu.DM.03G024670/Soltu.DM.06G023410/Soltu.DM.05G003100/Soltu.DM.03G024690/Soltu.DM.10G000600/Soltu.DM.04G022240/Soltu.DM.03G000400/Soltu.DM.11G001500	12
1684	GO:0046434	organophosphate catabolic process	73/12756	0.688650245	0.999994594	0.94442346	Soltu.DM.02G034460/Soltu.DM.12G004480/Soltu.DM.06G013720/Soltu.DM.01G005590/Soltu.DM.03G024130/Soltu.DM.02G019940	6
1685	GO:0045017	glycerolipid biosynthetic process	108/12756	0.689847606	0.999994594	0.94442346	Soltu.DM.02G024810/Soltu.DM.03G030800/Soltu.DM.12G002120/Soltu.DM.02G010490/Soltu.DM.01G034820/Soltu.DM.04G034770/Soltu.DM.05G001470/Soltu.DM.06G018040/Soltu.DM.10G005430	9
1686	GO:0000305	response to oxygen radical	12/12756	0.692042277	0.999994594	0.94442346	Soltu.DM.06G012170	1
1687	GO:0006270	DNA replication initiation	12/12756	0.692042277	0.999994594	0.94442346	Soltu.DM.12G029710	1
1688	GO:0006297	nucleotide-excision repair, DNA gap filling	12/1275	0.6920422	0.9999945	0.9444234	Soltu.DM.08G027160	1

			6	77	94	6		
1689	GO:0006356	regulation of transcription by RNA polymerase I	12/12756	0.692042277	0.999994594	0.94442346	Soltu.DM.07G026610	1
1690	GO:0006368	transcription elongation by RNA polymerase II	12/12756	0.692042277	0.999994594	0.94442346	Soltu.DM.11G008820	1
1691	GO:0006740	NADPH regeneration	12/12756	0.692042277	0.999994594	0.94442346	Soltu.DM.08G014620	1
1692	GO:0006828	manganese ion transport	12/12756	0.692042277	0.999994594	0.94442346	Soltu.DM.04G003430	1
1693	GO:0006896	Golgi to vacuole transport	12/12756	0.692042277	0.999994594	0.94442346	Soltu.DM.01G042120	1
1694	GO:0007006	mitochondrial membrane organization	12/12756	0.692042277	0.999994594	0.94442346	Soltu.DM.06G025410	1
1695	GO:0007029	endoplasmic reticulum organization	12/12756	0.692042277	0.999994594	0.94442346	Soltu.DM.06G021170	1
1696	GO:0007548	sex differentiation	12/12756	0.692042277	0.999994594	0.94442346	Soltu.DM.12G019270	1
1697	GO:0009704	de-etiolation	12/12756	0.692042277	0.999994594	0.94442346	Soltu.DM.06G023410	1
1698	GO:0009799	specification of symmetry	12/12756	0.692042277	0.999994594	0.94442346	Soltu.DM.02G003130	1
1699	GO:0010047	fruit dehiscence	12/12756	0.692042277	0.999994594	0.94442346	Soltu.DM.08G005070	1
1700	GO:0010207	photosystem II assembly	12/12756	0.692042277	0.999994594	0.94442346	Soltu.DM.03G022850	1
1701	GO:0010970	transport along microtubule	12/12756	0.692042277	0.999994594	0.94442346	Soltu.DM.07G024370	1
1702	GO:0015692	lead ion transport	12/12756	0.692042277	0.999994594	0.94442346	Soltu.DM.09G028710	1
1703	GO:0016925	protein sumoylation	12/12756	0.692042277	0.999994594	0.94442346	Soltu.DM.03G003730	1
1704	GO:0018195	peptidyl-arginine modification	12/12756	0.692042277	0.999994594	0.94442346	Soltu.DM.06G017810	1
1705	GO:0018216	peptidyl-arginine methylation	12/12756	0.692042277	0.999994594	0.94442346	Soltu.DM.06G017810	1
1706	GO:0019856	pyrimidine nucleobase biosynthetic process	12/12	0.6920	0.9999	0.9444	Soltu.DM.01G017170	1

			75 6	422 77	945 94	234 6		
17 07	GO:00 30104	water homeostasis	12/ 12 75 6	0.6 920 422 77	0.9 999 945 94	0.9 444 234 6	Soltu.DM.05G027000	1
17 08	GO:00 30178	negative regulation of Wnt signaling pathway	12/ 12 75 6	0.6 920 422 77	0.9 999 945 94	0.9 444 234 6	Soltu.DM.07G002580	1
17 09	GO:00 31033	myosin filament organization	12/ 12 75 6	0.6 920 422 77	0.9 999 945 94	0.9 444 234 6	Soltu.DM.01G028770	1
17 10	GO:00 31154	culmination involved in sorocarp development	12/ 12 75 6	0.6 920 422 77	0.9 999 945 94	0.9 444 234 6	Soltu.DM.01G028770	1
17 11	GO:00 31505	fungal-type cell wall organization	12/ 12 75 6	0.6 920 422 77	0.9 999 945 94	0.9 444 234 6	Soltu.DM.02G025970	1
17 12	GO:00 34656	nucleobase-containing small molecule catabolic process	12/ 12 75 6	0.6 920 422 77	0.9 999 945 94	0.9 444 234 6	Soltu.DM.12G003790	1
17 13	GO:00 45332	phospholipid translocation	12/ 12 75 6	0.6 920 422 77	0.9 999 945 94	0.9 444 234 6	Soltu.DM.06G022490	1
17 14	GO:00 45912	negative regulation of carbohydrate metabolic process	12/ 12 75 6	0.6 920 422 77	0.9 999 945 94	0.9 444 234 6	Soltu.DM.01G024860	1
17 15	GO:00 45943	positive regulation of transcription by RNA polymerase I	12/ 12 75 6	0.6 920 422 77	0.9 999 945 94	0.9 444 234 6	Soltu.DM.07G026610	1
17 16	GO:00 46621	negative regulation of organ growth	12/ 12 75 6	0.6 920 422 77	0.9 999 945 94	0.9 444 234 6	Soltu.DM.04G033430	1
17 17	GO:00 48564	photosystem I assembly	12/ 12 75 6	0.6 920 422 77	0.9 999 945 94	0.9 444 234 6	Soltu.DM.04G037460	1
17 18	GO:00 48829	root cap development	12/ 12 75 6	0.6 920 422 77	0.9 999 945 94	0.9 444 234 6	Soltu.DM.03G034800	1
17 19	GO:01 10154	RNA decapping	12/ 12 75 6	0.6 920 422 77	0.9 999 945 94	0.9 444 234 6	Soltu.DM.05G026810	1
17 20	GO:01 10156	methylguanosine-cap decapping	12/ 12 75 6	0.6 920 422 77	0.9 999 945 94	0.9 444 234 6	Soltu.DM.05G026810	1
17 21	GO:19 01038	cyanidin 3-O-glucoside metabolic process	12/ 12 75 6	0.6 920 422 77	0.9 999 945 94	0.9 444 234 6	Soltu.DM.09G025040	1
17 22	GO:19 01804	beta-glucoside metabolic process	12/ 12 75 6	0.6 920 422 77	0.9 999 945 94	0.9 444 234 6	Soltu.DM.09G025040	1
17 23	GO:19 04062	regulation of monoatomic cation transmembrane transport	12/ 12 75 6	0.6 920 422 77	0.9 999 945 94	0.9 444 234 6	Soltu.DM.07G024250	1
17	GO:00	autophagosome assembly	25/	0.6	0.9	0.9	Soltu.DM.01G039130/Soltu.DM.11G022310	2

24	00045		12756	92425928	99994594	4442346		
1725	GO:0009292	horizontal gene transfer	25/12756	0.692425928	0.999994594	0.94442346	Soltu.DM.07G020410/Soltu.DM.03G002260	2
1726	GO:0009294	DNA-mediated transformation	25/12756	0.692425928	0.999994594	0.94442346	Soltu.DM.07G020410/Soltu.DM.03G002260	2
1727	GO:0045003	double-strand break repair via synthesis-dependent strand annealing	25/12756	0.692425928	0.999994594	0.94442346	Soltu.DM.09G025170/Soltu.DM.03G002260	2
1728	GO:0045055	regulated exocytosis	25/12756	0.692425928	0.999994594	0.94442346	Soltu.DM.10G022360/Soltu.DM.11G026460	2
1729	GO:0048572	short-day photoperiodism	25/12756	0.692425928	0.999994594	0.94442346	Soltu.DM.06G012790/Soltu.DM.02G002480	2
1730	GO:0048575	short-day photoperiodism, flowering	25/12756	0.692425928	0.999994594	0.94442346	Soltu.DM.06G012790/Soltu.DM.02G002480	2
1731	GO:2000031	regulation of salicylic acid mediated signaling pathway	25/12756	0.692425928	0.999994594	0.94442346	Soltu.DM.04G033180/Soltu.DM.12G026070	2
1732	GO:0006163	purine nucleotide metabolic process	177/12756	0.693285802	0.999994594	0.94442346	Soltu.DM.08G014620/Soltu.DM.05G011440/Soltu.DM.12G004480/Soltu.DM.01G033530/Soltu.DM.02G018700/Soltu.DM.03G037170/Soltu.DM.07G009580/Soltu.DM.10G027910/Soltu.DM.06G013720/Soltu.DM.03G021730/Soltu.DM.08G026810/Soltu.DM.02G031030/Soltu.DM.11G025570/Soltu.DM.11G010590/Soltu.DM.01G019520	15
1733	GO:0021700	developmental maturation	200/12756	0.695876887	0.999994594	0.94442346	Soltu.DM.07G013360/Soltu.DM.08G027650/Soltu.DM.04G001370/Soltu.DM.06G005370/Soltu.DM.12G004060/Soltu.DM.12G005490/Soltu.DM.08G023170/Soltu.DM.12G024710/Soltu.DM.09G026810/Soltu.DM.08G011330/Soltu.DM.03G001740/Soltu.DM.01G042120/Soltu.DM.01G001590/Soltu.DM.03G013100/Soltu.DM.07G024500/Soltu.DM.03G018740/Soltu.DM.04G002690	17
1734	GO:0009615	response to virus	97/12756	0.696224394	0.999994594	0.94442346	Soltu.DM.01G035490/Soltu.DM.10G025390/Soltu.DM.02G012280/Soltu.DM.01G003040/Soltu.DM.04G007430/Soltu.DM.03G021360/Soltu.DM.01G010020/Soltu.DM.11G004150	8
1735	GO:0009863	salicylic acid mediated signaling pathway	50/12756	0.699493458	0.999994594	0.94442346	Soltu.DM.09G018910/Soltu.DM.02G006310/Soltu.DM.04G028540/Soltu.DM.07G024240	4
1736	GO:0060249	anatomical structure homeostasis	50/12756	0.699493458	0.999994594	0.94442346	Soltu.DM.08G027160/Soltu.DM.11G017980/Soltu.DM.05G023970/Soltu.DM.02G013390	4
1737	GO:0009740	gibberellic acid mediated signaling pathway	74/12756	0.70134887	0.999994594	0.94442346	Soltu.DM.02G011120/Soltu.DM.06G012790/Soltu.DM.08G011890/Soltu.DM.01G024340/Soltu.DM.06G002140/Soltu.DM.06G023440	6
1738	GO:0006476	protein deacetylation	38/12756	0.702622277	0.999994594	0.94442346	Soltu.DM.04G033160/Soltu.DM.02G001620/Soltu.DM.02G001630	3
1739	GO:0031330	negative regulation of cellular catabolic process	38/12756	0.702622277	0.999994594	0.94442346	Soltu.DM.09G014740/Soltu.DM.11G016820/Soltu.DM.01G002690	3
1740	GO:0072528	pyrimidine-containing compound biosynthetic process	38/12756	0.702622277	0.999994594	0.94442346	Soltu.DM.11G007630/Soltu.DM.01G017170/Soltu.DM.06G003240	3
1741	GO:0002253	activation of immune response	86/1275	0.7040055	0.9999945	0.9444234	Soltu.DM.02G025590/Soltu.DM.07G028550/Soltu.DM.10G000640/Soltu.DM.04G018070/Soltu.DM.08G011890/Soltu.DM.02G022460/Soltu.DM.01G051770	7

			6	57	94	6		
1742	GO:0000377	RNA splicing, via transesterification reactions with bulged adenosine as nucleophile	167/12756	0.704943951	0.999994594	0.94442346	Soltu.DM.02G018020/Soltu.DM.04G029350/Soltu.DM.12G005490/Soltu.DM.02G025260/Soltu.DM.12G025260/Soltu.DM.11G014750/Soltu.DM.02G021990/Soltu.DM.11G021400/Soltu.DM.09G001490/Soltu.DM.10G028250/Soltu.DM.11G025410/Soltu.DM.10G001400/Soltu.DM.12G025710/Soltu.DM.07G006530	14
1743	GO:0000209	protein polyubiquitination	98/12756	0.707186757	0.999994594	0.94442346	Soltu.DM.12G005510/Soltu.DM.09G006890/Soltu.DM.04G034740/Soltu.DM.05G005120/Soltu.DM.02G002480/Soltu.DM.02G033290/Soltu.DM.07G026100/Soltu.DM.06G024530	8
1744	GO:0009561	megagametogenesis	63/12756	0.713055858	0.999994594	0.94442346	Soltu.DM.05G023990/Soltu.DM.02G012680/Soltu.DM.01G002850/Soltu.DM.12G002630/Soltu.DM.10G014590	5
1745	GO:0001932	regulation of protein phosphorylation	145/12756	0.713121265	0.999994594	0.94442346	Soltu.DM.07G017210/Soltu.DM.07G017190/Soltu.DM.07G017200/Soltu.DM.02G018520/Soltu.DM.07G017180/Soltu.DM.07G012130/Soltu.DM.06G018320/Soltu.DM.11G010230/Soltu.DM.02G028740/Soltu.DM.11G010220/Soltu.DM.06G028580/Soltu.DM.04G038280	12
1746	GO:0006220	pyrimidine nucleotide metabolic process	26/12756	0.713167648	0.999994594	0.94442346	Soltu.DM.11G007630/Soltu.DM.01G017170	2
1747	GO:0006857	oligopeptide transport	26/12756	0.713167648	0.999994594	0.94442346	Soltu.DM.08G029920/Soltu.DM.04G031480	2
1748	GO:0009084	glutamine family amino acid biosynthetic process	26/12756	0.713167648	0.999994594	0.94442346	Soltu.DM.08G007450/Soltu.DM.07G014750	2
1749	GO:0010025	wax biosynthetic process	26/12756	0.713167648	0.999994594	0.94442346	Soltu.DM.10G005260/Soltu.DM.10G005430	2
1750	GO:0034661	ncRNA catabolic process	26/12756	0.713167648	0.999994594	0.94442346	Soltu.DM.09G014740/Soltu.DM.11G004920	2
1751	GO:0042770	signal transduction in response to DNA damage	26/12756	0.713167648	0.999994594	0.94442346	Soltu.DM.11G016820/Soltu.DM.02G033290	2
1752	GO:0046854	phosphatidylinositol phosphate biosynthetic process	26/12756	0.713167648	0.999994594	0.94442346	Soltu.DM.04G034770/Soltu.DM.05G001470	2
1753	GO:0048640	negative regulation of developmental growth	26/12756	0.713167648	0.999994594	0.94442346	Soltu.DM.01G024670/Soltu.DM.04G033430	2
1754	GO:0051648	vesicle localization	26/12756	0.713167648	0.999994594	0.94442346	Soltu.DM.09G018720/Soltu.DM.11G026460	2
1755	GO:0071577	zinc ion transmembrane transport	26/12756	0.713167648	0.999994594	0.94442346	Soltu.DM.07G002440/Soltu.DM.07G027100	2
1756	GO:0098586	cellular response to virus	26/12756	0.713167648	0.999994594	0.94442346	Soltu.DM.01G010020/Soltu.DM.11G004150	2
1757	GO:0005982	starch metabolic process	51/12756	0.714374279	0.999994594	0.94442346	Soltu.DM.09G027770/Soltu.DM.05G006330/Soltu.DM.04G037250/Soltu.DM.01G051470	4
1758	GO:0016032	viral process	51/12756	0.714374279	0.999994594	0.94442346	Soltu.DM.02G012280/Soltu.DM.03G003730/Soltu.DM.03G021360/Soltu.DM.05G026810	4
1759	GO:0044743	protein transmembrane import into intracellular organelle	51/12	0.7143	0.9999	0.9444	Soltu.DM.04G037150/Soltu.DM.08G023320/Soltu.DM.02G032340/Soltu.DM.12G021450	4

			756	74279	94594	2346		
1760	GO:0042325	regulation of phosphorylation	157/12756	0.717330622	0.999994594	0.944423456	Soltu.DM.07G017210/Soltu.DM.07G017190/Soltu.DM.07G017200/Soltu.DM.02G018520/Soltu.DM.07G017180/Soltu.DM.07G012130/Soltu.DM.06G018320/Soltu.DM.11G010230/Soltu.DM.02G028740/Soltu.DM.11G010220/Soltu.DM.06G028580/Soltu.DM.04G038280/Soltu.DM.01G051340	13
1761	GO:0045664	regulation of neuron differentiation	39/12756	0.719421327	0.999994594	0.944423456	Soltu.DM.12G020370/Soltu.DM.12G020350/Soltu.DM.12G020340	3
1762	GO:0071367	cellular response to brassinosteroid stimulus	39/12756	0.719421327	0.999994594	0.944423456	Soltu.DM.08G011890/Soltu.DM.04G029270/Soltu.DM.04G021540	3
1763	GO:0000387	spliceosomal snRNP assembly	13/12756	0.72084686	0.999994594	0.944423456	Soltu.DM.10G028250	1
1764	GO:0006108	malate metabolic process	13/12756	0.72084686	0.999994594	0.944423456	Soltu.DM.08G014620	1
1765	GO:0006414	translational elongation	13/12756	0.72084686	0.999994594	0.944423456	Soltu.DM.12G028920	1
1766	GO:0006821	chloride transport	13/12756	0.72084686	0.999994594	0.944423456	Soltu.DM.02G010790	1
1767	GO:0006999	nuclear pore organization	13/12756	0.72084686	0.999994594	0.944423456	Soltu.DM.04G011330	1
1768	GO:0009092	homoserine metabolic process	13/12756	0.72084686	0.999994594	0.944423456	Soltu.DM.08G030020	1
1769	GO:0009645	response to low light intensity stimulus	13/12756	0.72084686	0.999994594	0.944423456	Soltu.DM.08G011890	1
1770	GO:0009717	isoflavonoid biosynthetic process	13/12756	0.72084686	0.999994594	0.944423456	Soltu.DM.05G021610	1
1771	GO:0009942	longitudinal axis specification	13/12756	0.72084686	0.999994594	0.944423456	Soltu.DM.04G002690	1
1772	GO:0009954	proximal/distal pattern formation	13/12756	0.72084686	0.999994594	0.944423456	Soltu.DM.04G031030	1
1773	GO:0010206	photosystem II repair	13/12756	0.72084686	0.999994594	0.944423456	Soltu.DM.04G037460	1
1774	GO:0016093	polyprenol metabolic process	13/12756	0.72084686	0.999994594	0.944423456	Soltu.DM.10G022710	1
1775	GO:0016123	xanthophyll biosynthetic process	13/12756	0.72084686	0.999994594	0.944423456	Soltu.DM.06G029640	1
1776	GO:0018023	peptidyl-lysine trimethylation	13/12756	0.72084686	0.999994594	0.944423456	Soltu.DM.05G000070	1
17	GO:00	establishment of cell polarity	13/	0.7	0.9	0.9	Soltu.DM.02G016680	1

77	30010		12756	2084686	99994594	4442346		
1778	GO:0030335	positive regulation of cell migration	13/12756	0.72084686	0.999994594	0.94442346	Soltu.DM.02G018520	1
1779	GO:0032881	regulation of polysaccharide metabolic process	13/12756	0.72084686	0.999994594	0.94442346	Soltu.DM.08G008380	1
1780	GO:0034204	lipid translocation	13/12756	0.72084686	0.999994594	0.94442346	Soltu.DM.06G022490	1
1781	GO:0034755	iron ion transmembrane transport	13/12756	0.72084686	0.999994594	0.94442346	Soltu.DM.04G003430	1
1782	GO:0042136	neurotransmitter biosynthetic process	13/12756	0.72084686	0.999994594	0.94442346	Soltu.DM.06G034310	1
1783	GO:0046287	isoflavonoid metabolic process	13/12756	0.72084686	0.999994594	0.94442346	Soltu.DM.05G021610	1
1784	GO:0050688	regulation of defense response to virus	13/12756	0.72084686	0.999994594	0.94442346	Soltu.DM.03G021360	1
1785	GO:0051123	RNA polymerase II preinitiation complex assembly	13/12756	0.72084686	0.999994594	0.94442346	Soltu.DM.04G038280	1
1786	GO:0051315	attachment of mitotic spindle microtubules to kinetochore	13/12756	0.72084686	0.999994594	0.94442346	Soltu.DM.01G043730	1
1787	GO:0060628	regulation of ER to Golgi vesicle-mediated transport	13/12756	0.72084686	0.999994594	0.94442346	Soltu.DM.02G030830	1
1788	GO:0070601	centromeric sister chromatid cohesion	13/12756	0.72084686	0.999994594	0.94442346	Soltu.DM.06G019850	1
1789	GO:0071333	cellular response to glucose stimulus	13/12756	0.72084686	0.999994594	0.94442346	Soltu.DM.08G008380	1
1790	GO:0071852	fungal-type cell wall organization or biogenesis	13/12756	0.72084686	0.999994594	0.94442346	Soltu.DM.02G025970	1
1791	GO:0080026	response to indolebutyric acid	13/12756	0.72084686	0.999994594	0.94442346	Soltu.DM.03G035710	1
1792	GO:0080051	cutin transport	13/12756	0.72084686	0.999994594	0.94442346	Soltu.DM.03G024040	1
1793	GO:0090181	regulation of cholesterol metabolic process	13/12756	0.72084686	0.999994594	0.94442346	Soltu.DM.10G004300	1
1794	GO:0090287	regulation of cellular response to growth factor stimulus	13/12756	0.72084686	0.999994594	0.94442346	Soltu.DM.12G025260	1

1795	GO:0097035	regulation of membrane lipid distribution	13/12756	0.72084686	0.999994594	0.94442346	Soltu.DM.06G022490	1
1796	GO:0099003	vesicle-mediated transport in synapse	13/12756	0.72084686	0.999994594	0.94442346	Soltu.DM.11G026460	1
1797	GO:2000027	regulation of animal organ morphogenesis	13/12756	0.72084686	0.999994594	0.94442346	Soltu.DM.07G002580	1
1798	GO:2000147	positive regulation of cell motility	13/12756	0.72084686	0.999994594	0.94442346	Soltu.DM.02G018520	1
1799	GO:0000375	RNA splicing, via transesterification reactions	169/12756	0.721486996	0.999994594	0.94442346	Soltu.DM.02G018020/Soltu.DM.04G029350/Soltu.DM.12G005490/Soltu.DM.02G025260/Soltu.DM.12G025260/Soltu.DM.11G014750/Soltu.DM.02G021990/Soltu.DM.11G021400/Soltu.DM.09G001490/Soltu.DM.10G028250/Soltu.DM.11G025410/Soltu.DM.10G001400/Soltu.DM.12G025710/Soltu.DM.07G006530	14
1800	GO:0022603	regulation of anatomical structure morphogenesis	192/12756	0.72036819	0.999994594	0.94442346	Soltu.DM.12G020370/Soltu.DM.02G018520/Soltu.DM.12G020350/Soltu.DM.06G025210/Soltu.DM.01G028770/Soltu.DM.04G022240/Soltu.DM.02G023840/Soltu.DM.02G030780/Soltu.DM.02G017390/Soltu.DM.07G002580/Soltu.DM.08G012010/Soltu.DM.06G018040/Soltu.DM.06G023200/Soltu.DM.12G020340/Soltu.DM.09G027230/Soltu.DM.03G034300	16
1801	GO:0000041	transition metal ion transport	76/12756	0.725684017	0.999994594	0.94442346	Soltu.DM.03G035710/Soltu.DM.07G002440/Soltu.DM.04G003430/Soltu.DM.03G020090/Soltu.DM.08G012080/Soltu.DM.07G027100	6
1802	GO:0006403	RNA localization	88/12756	0.72659796	0.999994594	0.94442346	Soltu.DM.12G005490/Soltu.DM.03G003730/Soltu.DM.12G024350/Soltu.DM.09G005370/Soltu.DM.11G024760/Soltu.DM.04G011330/Soltu.DM.07G006510	7
1803	GO:0009199	ribonucleoside triphosphate metabolic process	88/12756	0.72659796	0.999994594	0.94442346	Soltu.DM.12G004480/Soltu.DM.02G018700/Soltu.DM.07G009580/Soltu.DM.10G027910/Soltu.DM.06G013720/Soltu.DM.01G017170/Soltu.DM.11G025570	7
1804	GO:0006406	mRNA export from nucleus	52/12756	0.728706733	0.999994594	0.94442346	Soltu.DM.12G005490/Soltu.DM.03G003730/Soltu.DM.04G011330/Soltu.DM.07G006510	4
1805	GO:0045597	positive regulation of cell differentiation	52/12756	0.728706733	0.999994594	0.94442346	Soltu.DM.12G020370/Soltu.DM.12G020350/Soltu.DM.12G025260/Soltu.DM.12G020340	4
1806	GO:0051028	mRNA transport	52/12756	0.728706733	0.999994594	0.94442346	Soltu.DM.12G005490/Soltu.DM.03G003730/Soltu.DM.04G011330/Soltu.DM.07G006510	4
1807	GO:0000469	cleavage involved in rRNA processing	27/12756	0.732722543	0.999994594	0.94442346	Soltu.DM.12G024350/Soltu.DM.01G051600	2
1808	GO:0006290	pyrimidine dimer repair	27/12756	0.732722543	0.999994594	0.94442346	Soltu.DM.07G017530/Soltu.DM.05G018370	2
1809	GO:0006367	transcription initiation at RNA polymerase II promoter	27/12756	0.732722543	0.999994594	0.94442346	Soltu.DM.04G038280/Soltu.DM.11G015370	2
1810	GO:0009567	double fertilization forming a zygote and endosperm	27/12756	0.732722543	0.999994594	0.94442346	Soltu.DM.04G000190/Soltu.DM.05G004430	2
1811	GO:0010093	specification of floral organ identity	27/12756	0.732722543	0.999994594	0.94442346	Soltu.DM.10G023790/Soltu.DM.05G003100	2
1812	GO:0042398	cellular modified amino acid biosynthetic process	27/1275	0.7327225	0.9999945	0.9444234	Soltu.DM.04G025250/Soltu.DM.09G006800	2

			6	43	94	6		
1813	GO:0044273	sulfur compound catabolic process	27/12756	0.732722543	0.999994594	0.94442346	Soltu.DM.03G035710/Soltu.DM.08G030020	2
1814	GO:0090701	specification of plant organ identity	27/12756	0.732722543	0.999994594	0.94442346	Soltu.DM.10G023790/Soltu.DM.05G003100	2
1815	GO:1900426	positive regulation of defense response to bacterium	27/12756	0.732722543	0.999994594	0.94442346	Soltu.DM.04G000670/Soltu.DM.02G026820	2
1816	GO:0009668	plastid membrane organization	40/12756	0.735472875	0.999994594	0.94442346	Soltu.DM.08G001900/Soltu.DM.08G011890/Soltu.DM.10G022710	3
1817	GO:0016108	tetraterpenoid metabolic process	40/12756	0.735472875	0.999994594	0.94442346	Soltu.DM.08G028310/Soltu.DM.06G029640/Soltu.DM.12G026560	3
1818	GO:0016116	carotenoid metabolic process	40/12756	0.735472875	0.999994594	0.94442346	Soltu.DM.08G028310/Soltu.DM.06G029640/Soltu.DM.12G026560	3
1819	GO:0032984	protein-containing complex disassembly	40/12756	0.735472875	0.999994594	0.94442346	Soltu.DM.07G001240/Soltu.DM.08G019530/Soltu.DM.11G010650	3
1820	GO:0044772	mitotic cell cycle phase transition	40/12756	0.735472875	0.999994594	0.94442346	Soltu.DM.03G003730/Soltu.DM.02G029740/Soltu.DM.01G047090	3
1821	GO:0090068	positive regulation of cell cycle process	40/12756	0.735472875	0.999994594	0.94442346	Soltu.DM.03G003730/Soltu.DM.11G016820/Soltu.DM.12G023230	3
1822	GO:0090501	RNA phosphodiester bond hydrolysis	136/12756	0.736004699	0.999994594	0.94442346	Soltu.DM.02G018270/Soltu.DM.01G006380/Soltu.DM.09G014740/Soltu.DM.12G024350/Soltu.DM.01G051600/Soltu.DM.11G004920/Soltu.DM.07G006530/Soltu.DM.09G002100/Soltu.DM.04G031030/Soltu.DM.06G029830/Soltu.DM.11G004150	11
1823	GO:1901607	alpha-amino acid biosynthetic process	136/12756	0.736004699	0.999994594	0.94442346	Soltu.DM.03G005810/Soltu.DM.08G030020/Soltu.DM.06G018090/Soltu.DM.07G023080/Soltu.DM.02G020220/Soltu.DM.08G011890/Soltu.DM.12G025770/Soltu.DM.11G003850/Soltu.DM.07G006550/Soltu.DM.08G007450/Soltu.DM.07G014750	11
1824	GO:0055080	monoatomic cation homeostasis	194/12756	0.737045773	0.999994594	0.94442346	Soltu.DM.07G002440/Soltu.DM.01G035900/Soltu.DM.07G028550/Soltu.DM.03G017590/Soltu.DM.10G010160/Soltu.DM.02G024200/Soltu.DM.10G000640/Soltu.DM.05G021830/Soltu.DM.04G003430/Soltu.DM.11G000570/Soltu.DM.07G009580/Soltu.DM.07G015200/Soltu.DM.10G004300/Soltu.DM.12G021010/Soltu.DM.01G035910/Soltu.DM.08G001690	16
1825	GO:0048581	negative regulation of post-embryonic development	148/12756	0.738982046	0.999994594	0.94442346	Soltu.DM.02G016290/Soltu.DM.02G016300/Soltu.DM.04G006870/Soltu.DM.02G016770/Soltu.DM.02G002480/Soltu.DM.02G016380/Soltu.DM.05G012040/Soltu.DM.01G024340/Soltu.DM.07G020080/Soltu.DM.02G016780/Soltu.DM.06G002140/Soltu.DM.04G033440	12
1826	GO:0010374	stomatal complex development	53/12756	0.742494343	0.999994594	0.94442346	Soltu.DM.05G021390/Soltu.DM.12G010960/Soltu.DM.08G012010/Soltu.DM.01G047090	4
1827	GO:0019751	polyol metabolic process	53/12756	0.742494343	0.999994594	0.94442346	Soltu.DM.02G018520/Soltu.DM.08G014180/Soltu.DM.12G024030/Soltu.DM.03G008510	4
1828	GO:0002274	myeloid leukocyte activation	14/12756	0.746959278	0.999994594	0.94442346	Soltu.DM.10G022360	1
1829	GO:0002275	myeloid cell activation involved in immune response	14/12756	0.746959278	0.999994594	0.94442346	Soltu.DM.10G022360	1
1830	GO:0002283	neutrophil activation involved in immune response	14/12	0.7469	0.9999	0.9444	Soltu.DM.10G022360	1

			756	59278	94594	2346	
1831	GO:0002444	myeloid leukocyte mediated immunity	14/12756	0.746959278	0.999994594	0.94442346	Soltu.DM.10G022360	1
1832	GO:0002446	neutrophil mediated immunity	14/12756	0.746959278	0.999994594	0.94442346	Soltu.DM.10G022360	1
1833	GO:0006094	gluconeogenesis	14/12756	0.746959278	0.999994594	0.94442346	Soltu.DM.04G031580	1
1834	GO:0006144	purine nucleobase metabolic process	14/12756	0.746959278	0.999994594	0.94442346	Soltu.DM.02G017810	1
1835	GO:0006525	arginine metabolic process	14/12756	0.746959278	0.999994594	0.94442346	Soltu.DM.08G007450	1
1836	GO:0006591	ornithine metabolic process	14/12756	0.746959278	0.999994594	0.94442346	Soltu.DM.08G007450	1
1837	GO:0009903	chloroplast avoidance movement	14/12756	0.746959278	0.999994594	0.94442346	Soltu.DM.10G001460	1
1838	GO:0010497	plasmodesmata-mediated intercellular transport	14/12756	0.746959278	0.999994594	0.94442346	Soltu.DM.01G047440	1
1839	GO:0015693	magnesium ion transport	14/12756	0.746959278	0.999994594	0.94442346	Soltu.DM.02G034690	1
1840	GO:0019217	regulation of fatty acid metabolic process	14/12756	0.746959278	0.999994594	0.94442346	Soltu.DM.07G024240	1
1841	GO:0019287	isopentenyl diphosphate biosynthetic process, mevalonate pathway	14/12756	0.746959278	0.999994594	0.94442346	Soltu.DM.08G026810	1
1842	GO:0030091	protein repair	14/12756	0.746959278	0.999994594	0.94442346	Soltu.DM.04G037460	1
1843	GO:0034260	negative regulation of GTPase activity	14/12756	0.746959278	0.999994594	0.94442346	Soltu.DM.08G011890	1
1844	GO:0035542	regulation of SNARE complex assembly	14/12756	0.746959278	0.999994594	0.94442346	Soltu.DM.06G005370	1
1845	GO:0036230	granulocyte activation	14/12756	0.746959278	0.999994594	0.94442346	Soltu.DM.10G022360	1
1846	GO:0042119	neutrophil activation	14/12756	0.746959278	0.999994594	0.94442346	Soltu.DM.10G022360	1
1847	GO:0043299	leukocyte degranulation	14/12756	0.746959278	0.999994594	0.94442346	Soltu.DM.10G022360	1
18	GO:00	neutrophil degranulation	14/	0.7	0.9	0.9	Soltu.DM.10G022360	1

48	43312		12756	46959278	99994594	4442346		
1849	GO:0046132	pyrimidine ribonucleoside biosynthetic process	14/12756	0.746959278	0.999994594	0.94442346	Soltu.DM.01G017170	1
1850	GO:0046355	mannan catabolic process	14/12756	0.746959278	0.999994594	0.94442346	Soltu.DM.11G000740	1
1851	GO:0048768	root hair cell tip growth	14/12756	0.746959278	0.999994594	0.94442346	Soltu.DM.09G026810	1
1852	GO:0050982	detection of mechanical stimulus	14/12756	0.746959278	0.999994594	0.94442346	Soltu.DM.01G028770	1
1853	GO:0051156	glucose 6-phosphate metabolic process	14/12756	0.746959278	0.999994594	0.94442346	Soltu.DM.08G014620	1
1854	GO:0051973	positive regulation of telomerase activity	14/12756	0.746959278	0.999994594	0.94442346	Soltu.DM.06G026960	1
1855	GO:0071326	cellular response to monosaccharide stimulus	14/12756	0.746959278	0.999994594	0.94442346	Soltu.DM.08G008380	1
1856	GO:0071331	cellular response to hexose stimulus	14/12756	0.746959278	0.999994594	0.94442346	Soltu.DM.08G008380	1
1857	GO:0090332	stomatal closure	14/12756	0.746959278	0.999994594	0.94442346	Soltu.DM.09G026500	1
1858	GO:2000117	negative regulation of cysteine-type endopeptidase activity	14/12756	0.746959278	0.999994594	0.94442346	Soltu.DM.03G020450	1
1859	GO:0043487	regulation of RNA stability	78/12756	0.748595855	0.999994594	0.94442346	Soltu.DM.05G004270/Soltu.DM.08G013620/Soltu.DM.05G026810/Soltu.DM.11G016820/Soltu.DM.01G002690/Soltu.DM.04G031030	6
1860	GO:0008284	positive regulation of cell population proliferation	41/12756	0.750789359	0.999994594	0.94442346	Soltu.DM.12G026600/Soltu.DM.02G018520/Soltu.DM.02G016680	3
1861	GO:0042157	lipoprotein metabolic process	41/12756	0.750789359	0.999994594	0.94442346	Soltu.DM.01G039130/Soltu.DM.02G010490/Soltu.DM.11G022310	3
1862	GO:0006829	zinc ion transport	28/12756	0.751131103	0.999994594	0.94442346	Soltu.DM.07G002440/Soltu.DM.07G027100	2
1863	GO:0009612	response to mechanical stimulus	28/12756	0.751131103	0.999994594	0.94442346	Soltu.DM.07G020410/Soltu.DM.01G028770	2
1864	GO:0010166	wax metabolic process	28/12756	0.751131103	0.999994594	0.94442346	Soltu.DM.10G005260/Soltu.DM.10G005430	2
1865	GO:0010492	maintenance of shoot apical meristem identity	28/12756	0.751131103	0.999994594	0.94442346	Soltu.DM.02G027330/Soltu.DM.01G010020	2

1866	GO:0031570	DNA integrity checkpoint signaling	28/12756	0.751131103	0.999994594	0.94442346	Soltu.DM.11G016820/Soltu.DM.02G033290	2
1867	GO:0034308	primary alcohol metabolic process	28/12756	0.751131103	0.999994594	0.94442346	Soltu.DM.03G018850/Soltu.DM.06G003240	2
1868	GO:1902905	positive regulation of supramolecular fiber organization	28/12756	0.751131103	0.999994594	0.94442346	Soltu.DM.09G015150/Soltu.DM.09G027230	2
1869	GO:0042391	regulation of membrane potential	54/12756	0.755742409	0.999994594	0.94442346	Soltu.DM.12G024710/Soltu.DM.08G018550/Soltu.DM.02G013580/Soltu.DM.06G013710	4
1870	GO:0051336	regulation of hydrolase activity	162/12756	0.757729185	0.999994594	0.94442346	Soltu.DM.09G031320/Soltu.DM.03G020450/Soltu.DM.04G034360/Soltu.DM.04G034390/Soltu.DM.04G034380/Soltu.DM.04G003450/Soltu.DM.08G011890/Soltu.DM.05G006580/Soltu.DM.09G031340/Soltu.DM.07G024370/Soltu.DM.04G034280/Soltu.DM.02G026820/Soltu.DM.02G022620	13
1871	GO:0051347	positive regulation of transferase activity	103/12756	0.758052895	0.999994594	0.94442346	Soltu.DM.06G026960/Soltu.DM.07G017210/Soltu.DM.07G017190/Soltu.DM.07G017200/Soltu.DM.07G017180/Soltu.DM.11G009630/Soltu.DM.02G028740/Soltu.DM.08G027160	8
1872	GO:0044087	regulation of cellular component biogenesis	197/12756	0.758572134	0.999994594	0.94442346	Soltu.DM.07G028550/Soltu.DM.08G029860/Soltu.DM.10G000640/Soltu.DM.06G005370/Soltu.DM.06G022970/Soltu.DM.06G028040/Soltu.DM.01G028770/Soltu.DM.03G022850/Soltu.DM.09G015150/Soltu.DM.11G011390/Soltu.DM.11G024760/Soltu.DM.12G009990/Soltu.DM.01G027520/Soltu.DM.10G004310/Soltu.DM.09G027230/Soltu.DM.12G023230	16
1873	GO:0002683	negative regulation of immune system process	42/12756	0.765385586	0.999994594	0.94442346	Soltu.DM.08G022900/Soltu.DM.04G022240/Soltu.DM.05G023030	3
1874	GO:0006081	cellular aldehyde metabolic process	42/12756	0.765385586	0.999994594	0.94442346	Soltu.DM.08G014620/Soltu.DM.03G018850/Soltu.DM.07G017900	3
1875	GO:0031344	regulation of cell projection organization	42/12756	0.765385586	0.999994594	0.94442346	Soltu.DM.12G020370/Soltu.DM.12G020350/Soltu.DM.12G020340	3
1876	GO:0051053	negative regulation of DNA metabolic process	42/12756	0.765385586	0.999994594	0.94442346	Soltu.DM.04G034330/Soltu.DM.05G006310/Soltu.DM.02G013390	3
1877	GO:0120035	regulation of plasma membrane bounded cell projection organization	42/12756	0.765385586	0.999994594	0.94442346	Soltu.DM.12G020370/Soltu.DM.12G020350/Soltu.DM.12G020340	3
1878	GO:0000097	sulfur amino acid biosynthetic process	29/12756	0.768436614	0.999994594	0.94442346	Soltu.DM.08G030020/Soltu.DM.12G025770	2
1879	GO:0009625	response to insect	29/12756	0.768436614	0.999994594	0.94442346	Soltu.DM.02G025590/Soltu.DM.07G022640	2
1880	GO:0009768	photosynthesis, light harvesting in photosystem I	29/12756	0.768436614	0.999994594	0.94442346	Soltu.DM.03G000900/Soltu.DM.07G024910	2
1881	GO:0016477	cell migration	29/12756	0.768436614	0.999994594	0.94442346	Soltu.DM.08G027150/Soltu.DM.09G002090	2
1882	GO:0033500	carbohydrate homeostasis	29/12756	0.768436614	0.999994594	0.94442346	Soltu.DM.08G008380/Soltu.DM.08G028440	2
1883	GO:0048826	cotyledon morphogenesis	29/1275	0.7684366	0.9999945	0.9444234	Soltu.DM.05G006190/Soltu.DM.03G024040	2

			6	14	94	6		
1884	GO:0055075	potassium ion homeostasis	29/12756	0.768436614	0.999994594	0.94442346	Soltu.DM.10G010160/Soltu.DM.12G021010	2
1885	GO:1901002	positive regulation of response to salt stress	29/12756	0.768436614	0.999994594	0.94442346	Soltu.DM.06G017300/Soltu.DM.02G026820	2
1886	GO:2001023	regulation of response to drug	29/12756	0.768436614	0.999994594	0.94442346	Soltu.DM.04G033180/Soltu.DM.12G026070	2
1887	GO:2001038	regulation of cellular response to drug	29/12756	0.768436614	0.999994594	0.94442346	Soltu.DM.04G033180/Soltu.DM.12G026070	2
1888	GO:0006090	pyruvate metabolic process	55/12756	0.768457814	0.999994594	0.94442346	Soltu.DM.08G014620/Soltu.DM.12G004480/Soltu.DM.06G013720/Soltu.DM.12G002000	4
1889	GO:0010311	lateral root formation	55/12756	0.768457814	0.999994594	0.94442346	Soltu.DM.02G006700/Soltu.DM.11G018040/Soltu.DM.06G020260/Soltu.DM.02G022410	4
1890	GO:0140352	export from cell	140/12756	0.769495986	0.999994594	0.94442346	Soltu.DM.09G002620/Soltu.DM.03G017520/Soltu.DM.06G005370/Soltu.DM.11G011180/Soltu.DM.10G022360/Soltu.DM.01G000060/Soltu.DM.02G032050/Soltu.DM.10G026500/Soltu.DM.11G026460/Soltu.DM.02G022410/Soltu.DM.04G002690	11
1891	GO:0017148	negative regulation of translation	80/12756	0.770089218	0.999994594	0.94442346	Soltu.DM.05G004270/Soltu.DM.04G001110/Soltu.DM.08G013620/Soltu.DM.05G006430/Soltu.DM.05G026810/Soltu.DM.04G031030	6
1892	GO:0048532	anatomical structure arrangement	80/12756	0.770089218	0.999994594	0.94442346	Soltu.DM.10G025140/Soltu.DM.09G002750/Soltu.DM.02G003130/Soltu.DM.05G026810/Soltu.DM.06G034230/Soltu.DM.03G034800	6
1893	GO:0001701	in utero embryonic development	15/12756	0.770630958	0.999994594	0.94442346	Soltu.DM.02G029740	1
1894	GO:0006493	protein O-linked glycosylation	15/12756	0.770630958	0.999994594	0.94442346	Soltu.DM.03G037510	1
1895	GO:0006801	superoxide metabolic process	15/12756	0.770630958	0.999994594	0.94442346	Soltu.DM.06G012170	1
1896	GO:0006909	phagocytosis	15/12756	0.770630958	0.999994594	0.94442346	Soltu.DM.08G001690	1
1897	GO:0007094	mitotic spindle assembly checkpoint signaling	15/12756	0.770630958	0.999994594	0.94442346	Soltu.DM.10G020590	1
1898	GO:0009959	negative gravitropism	15/12756	0.770630958	0.999994594	0.94442346	Soltu.DM.06G002140	1
1899	GO:0010496	intercellular transport	15/12756	0.770630958	0.999994594	0.94442346	Soltu.DM.01G047440	1
1900	GO:0015996	chlorophyll catabolic process	15/12756	0.770630958	0.999994594	0.94442346	Soltu.DM.07G010570	1
1901	GO:0019674	NAD metabolic process	15/12	0.7706	0.9999	0.9444	Soltu.DM.02G018700	1

			756	30958	94594	2346		
1902	GO:0030835	negative regulation of actin filament depolymerization	15/12756	0.770630958	0.999994594	0.94442346	Soltu.DM.09G015150	1
1903	GO:0031577	spindle checkpoint signaling	15/12756	0.770630958	0.999994594	0.94442346	Soltu.DM.10G020590	1
1904	GO:0042726	flavin-containing compound metabolic process	15/12756	0.770630958	0.999994594	0.94442346	Soltu.DM.01G044760	1
1905	GO:0042753	positive regulation of circadian rhythm	15/12756	0.770630958	0.999994594	0.94442346	Soltu.DM.02G011380	1
1906	GO:0045841	negative regulation of mitotic metaphase/anaphase transition	15/12756	0.770630958	0.999994594	0.94442346	Soltu.DM.10G020590	1
1907	GO:0046112	nucleobase biosynthetic process	15/12756	0.770630958	0.999994594	0.94442346	Soltu.DM.01G017170	1
1908	GO:0046149	pigment catabolic process	15/12756	0.770630958	0.999994594	0.94442346	Soltu.DM.07G010570	1
1909	GO:0048455	stamen formation	15/12756	0.770630958	0.999994594	0.94442346	Soltu.DM.05G003100	1
1910	GO:0051693	actin filament capping	15/12756	0.770630958	0.999994594	0.94442346	Soltu.DM.09G015150	1
1911	GO:0071173	spindle assembly checkpoint signaling	15/12756	0.770630958	0.999994594	0.94442346	Soltu.DM.10G020590	1
1912	GO:0071174	mitotic spindle checkpoint signaling	15/12756	0.770630958	0.999994594	0.94442346	Soltu.DM.10G020590	1
1913	GO:0080009	mRNA methylation	15/12756	0.770630958	0.999994594	0.94442346	Soltu.DM.01G032530	1
1914	GO:1902100	negative regulation of metaphase/anaphase transition of cell cycle	15/12756	0.770630958	0.999994594	0.94442346	Soltu.DM.10G020590	1
1915	GO:1902806	regulation of cell cycle G1/S phase transition	15/12756	0.770630958	0.999994594	0.94442346	Soltu.DM.11G016820	1
1916	GO:1905819	negative regulation of chromosome separation	15/12756	0.770630958	0.999994594	0.94442346	Soltu.DM.10G020590	1
1917	GO:2000033	regulation of seed dormancy process	15/12756	0.770630958	0.999994594	0.94442346	Soltu.DM.07G010930	1
1918	GO:2000816	negative regulation of mitotic sister chromatid separation	15/12756	0.770630958	0.999994594	0.94442346	Soltu.DM.10G020590	1
19	GO:00	chloroplast organization	16	0.7	0.9	0.9	Soltu.DM.06G018090/Soltu.DM.09G019220/Soltu.DM.01G047730/Soltu.DM.07G015200/S	13

19	09658		4/12756	72747836	99994594	4442346	oltu.DM.10G016030/Soltu.DM.03G022850/Soltu.DM.03G033060/Soltu.DM.06G021830/Soltu.DM.10G013520/Soltu.DM.02G027620/Soltu.DM.10G001460/Soltu.DM.09G002100/Soltu.DM.03G021590	
1920	GO:0034404	nucleobase-containing small molecule biosynthetic process	68/12756	0.773892103	0.999994594	0.94442346	Soltu.DM.12G004480/Soltu.DM.03G037170/Soltu.DM.10G027910/Soltu.DM.06G013720/Soltu.DM.01G017170	5
1921	GO:0034614	cellular response to reactive oxygen species	68/12756	0.773892103	0.999994594	0.94442346	Soltu.DM.07G028550/Soltu.DM.10G000640/Soltu.DM.02G030410/Soltu.DM.06G012170/Soltu.DM.06G026560	5
1922	GO:1903313	positive regulation of mRNA metabolic process	68/12756	0.773892103	0.999994594	0.94442346	Soltu.DM.05G004270/Soltu.DM.12G025260/Soltu.DM.08G013620/Soltu.DM.05G026810/Soltu.DM.04G031030	5
1923	GO:1905393	plant organ formation	141/12756	0.777386224	0.999994594	0.94442346	Soltu.DM.03G037070/Soltu.DM.06G022310/Soltu.DM.06G025210/Soltu.DM.10G023790/Soltu.DM.02G006700/Soltu.DM.05G003100/Soltu.DM.11G018040/Soltu.DM.06G020260/Soltu.DM.07G020980/Soltu.DM.02G022410/Soltu.DM.03G034300	11
1924	GO:0071359	cellular response to dsRNA	93/12756	0.777774159	0.999994594	0.94442346	Soltu.DM.12G005490/Soltu.DM.09G024860/Soltu.DM.12G026070/Soltu.DM.11G025410/Soltu.DM.01G010020/Soltu.DM.04G031030/Soltu.DM.11G004150	7
1925	GO:0006486	protein glycosylation	153/12756	0.778563466	0.999994594	0.94442346	Soltu.DM.04G011110/Soltu.DM.02G025970/Soltu.DM.02G024810/Soltu.DM.03G037510/Soltu.DM.10G027470/Soltu.DM.04G011320/Soltu.DM.04G011240/Soltu.DM.10G028070/Soltu.DM.04G000320/Soltu.DM.04G011370/Soltu.DM.05G001020/Soltu.DM.11G021090	12
1926	GO:0043413	macromolecule glycosylation	153/12756	0.778563466	0.999994594	0.94442346	Soltu.DM.04G011110/Soltu.DM.02G025970/Soltu.DM.02G024810/Soltu.DM.03G037510/Soltu.DM.10G027470/Soltu.DM.04G011320/Soltu.DM.04G011240/Soltu.DM.10G028070/Soltu.DM.04G000320/Soltu.DM.04G011370/Soltu.DM.05G001020/Soltu.DM.11G021090	12
1927	GO:0007088	regulation of mitotic nuclear division	43/12756	0.779278348	0.999994594	0.94442346	Soltu.DM.03G003730/Soltu.DM.10G020590/Soltu.DM.12G023230	3
1928	GO:0009765	photosynthesis, light harvesting	43/12756	0.779278348	0.999994594	0.94442346	Soltu.DM.03G000900/Soltu.DM.04G037460/Soltu.DM.07G024910	3
1929	GO:0035601	protein deacylation	43/12756	0.779278348	0.999994594	0.94442346	Soltu.DM.04G033160/Soltu.DM.02G001620/Soltu.DM.02G001630	3
1930	GO:0098732	macromolecule deacylation	43/12756	0.779278348	0.999994594	0.94442346	Soltu.DM.04G033160/Soltu.DM.02G001620/Soltu.DM.02G001630	3
1931	GO:0009205	purine ribonucleoside triphosphate metabolic process	81/12756	0.780309213	0.999994594	0.94442346	Soltu.DM.12G004480/Soltu.DM.02G018700/Soltu.DM.07G009580/Soltu.DM.10G027910/Soltu.DM.06G013720/Soltu.DM.11G025570	6
1932	GO:0071805	potassium ion transmembrane transport	81/12756	0.780309213	0.999994594	0.94442346	Soltu.DM.01G035900/Soltu.DM.10G010160/Soltu.DM.12G024710/Soltu.DM.08G018550/Soltu.DM.01G035910/Soltu.DM.02G013580	6
1933	GO:0010769	regulation of cell morphogenesis involved in differentiation	56/12756	0.780648837	0.999994594	0.94442346	Soltu.DM.12G020370/Soltu.DM.12G020350/Soltu.DM.06G023200/Soltu.DM.12G020340	4
1934	GO:0050779	RNA destabilization	56/12756	0.780648837	0.999994594	0.94442346	Soltu.DM.05G004270/Soltu.DM.08G013620/Soltu.DM.05G026810/Soltu.DM.04G031030	4
1935	GO:0061157	mRNA destabilization	56/12756	0.780648837	0.999994594	0.94442346	Soltu.DM.05G004270/Soltu.DM.08G013620/Soltu.DM.05G026810/Soltu.DM.04G031030	4
1936	GO:0060918	auxin transport	118/12756	0.784653684	0.999994594	0.94442346	Soltu.DM.01G035900/Soltu.DM.02G032050/Soltu.DM.10G026500/Soltu.DM.01G035910/Soltu.DM.03G036780/Soltu.DM.11G001500/Soltu.DM.08G001470/Soltu.DM.02G022410/Soltu.DM.04G002690	9

1937	GO:0009067	aspartate family amino acid biosynthetic process	30/12756	0.78468429	0.999994594	0.94442346	Soltu.DM.08G030020/Soltu.DM.07G006550	2
1938	GO:0016485	protein processing	30/12756	0.78468429	0.999994594	0.94442346	Soltu.DM.08G023320/Soltu.DM.06G018040	2
1939	GO:0006914	autophagy	69/12756	0.784790027	0.999994594	0.94442346	Soltu.DM.01G039130/Soltu.DM.11G022310/Soltu.DM.09G025980/Soltu.DM.10G011110/Soltu.DM.02G030830	5
1940	GO:0051262	protein tetramerization	69/12756	0.784790027	0.999994594	0.94442346	Soltu.DM.03G012810/Soltu.DM.07G013360/Soltu.DM.02G020550/Soltu.DM.08G030020/Soltu.DM.10G002820	5
1941	GO:0061919	process utilizing autophagic mechanism	69/12756	0.784790027	0.999994594	0.94442346	Soltu.DM.01G039130/Soltu.DM.11G022310/Soltu.DM.09G025980/Soltu.DM.10G011110/Soltu.DM.02G030830	5
1942	GO:0070918	regulatory ncRNA processing	94/12756	0.787105336	0.999994594	0.94442346	Soltu.DM.12G005490/Soltu.DM.09G024860/Soltu.DM.12G026070/Soltu.DM.11G025410/Soltu.DM.01G010020/Soltu.DM.04G031030/Soltu.DM.11G004150	7
1943	GO:0009144	purine nucleoside triphosphate metabolic process	82/12756	0.790182378	0.999994594	0.94442346	Soltu.DM.12G004480/Soltu.DM.02G018700/Soltu.DM.07G009580/Soltu.DM.10G027910/Soltu.DM.06G013720/Soltu.DM.11G025570	6
1944	GO:0048699	generation of neurons	82/12756	0.790182378	0.999994594	0.94442346	Soltu.DM.12G020370/Soltu.DM.01G042210/Soltu.DM.12G020350/Soltu.DM.12G025260/Soltu.DM.09G019870/Soltu.DM.12G020340	6
1945	GO:0002263	cell activation involved in immune response	16/12756	0.792089862	0.999994594	0.94442346	Soltu.DM.10G022360	1
1946	GO:0002366	leukocyte activation involved in immune response	16/12756	0.792089862	0.999994594	0.94442346	Soltu.DM.10G022360	1
1947	GO:0002443	leukocyte mediated immunity	16/12756	0.792089862	0.999994594	0.94442346	Soltu.DM.10G022360	1
1948	GO:0002793	positive regulation of peptide secretion	16/12756	0.792089862	0.999994594	0.94442346	Soltu.DM.01G002690	1
1949	GO:0010065	primary meristem tissue development	16/12756	0.792089862	0.999994594	0.94442346	Soltu.DM.07G020980	1
1950	GO:0010507	negative regulation of autophagy	16/12756	0.792089862	0.999994594	0.94442346	Soltu.DM.09G014740	1
1951	GO:0015691	cadmium ion transport	16/12756	0.792089862	0.999994594	0.94442346	Soltu.DM.03G035710	1
1952	GO:0016241	regulation of macroautophagy	16/12756	0.792089862	0.999994594	0.94442346	Soltu.DM.08G014180	1
1953	GO:0016558	protein import into peroxisome matrix	16/12756	0.792089862	0.999994594	0.94442346	Soltu.DM.08G023320	1
1954	GO:0016973	poly(A)+ mRNA export from nucleus	16/1275	0.7920898	0.9999945	0.9444234	Soltu.DM.04G011330	1

			6	62	94	6		
1955	GO:0030656	regulation of vitamin metabolic process	16/12756	0.792089862	0.999994594	0.94442346	Soltu.DM.03G008510	1
1956	GO:0032878	regulation of establishment or maintenance of cell polarity	16/12756	0.792089862	0.999994594	0.94442346	Soltu.DM.01G042120	1
1957	GO:0032958	inositol phosphate biosynthetic process	16/12756	0.792089862	0.999994594	0.94442346	Soltu.DM.03G008510	1
1958	GO:0033048	negative regulation of mitotic sister chromatid segregation	16/12756	0.792089862	0.999994594	0.94442346	Soltu.DM.10G020590	1
1959	GO:0046134	pyrimidine nucleoside biosynthetic process	16/12756	0.792089862	0.999994594	0.94442346	Soltu.DM.01G017170	1
1960	GO:0048194	Golgi vesicle budding	16/12756	0.792089862	0.999994594	0.94442346	Soltu.DM.06G022490	1
1961	GO:0050714	positive regulation of protein secretion	16/12756	0.792089862	0.999994594	0.94442346	Soltu.DM.01G002690	1
1962	GO:0051026	chiasma assembly	16/12756	0.792089862	0.999994594	0.94442346	Soltu.DM.09G025170	1
1963	GO:0051571	positive regulation of histone H3-K4 methylation	16/12756	0.792089862	0.999994594	0.94442346	Soltu.DM.12G025260	1
1964	GO:0060828	regulation of canonical Wnt signaling pathway	16/12756	0.792089862	0.999994594	0.94442346	Soltu.DM.07G002580	1
1965	GO:0070848	response to growth factor	16/12756	0.792089862	0.999994594	0.94442346	Soltu.DM.03G028660	1
1966	GO:0070897	transcription preinitiation complex assembly	16/12756	0.792089862	0.999994594	0.94442346	Soltu.DM.04G038280	1
1967	GO:0071265	L-methionine biosynthetic process	16/12756	0.792089862	0.999994594	0.94442346	Soltu.DM.08G030020	1
1968	GO:0071363	cellular response to growth factor stimulus	16/12756	0.792089862	0.999994594	0.94442346	Soltu.DM.03G028660	1
1969	GO:0090092	regulation of transmembrane receptor protein serine/threonine kinase signaling pathway	16/12756	0.792089862	0.999994594	0.94442346	Soltu.DM.12G025260	1
1970	GO:0097190	apoptotic signaling pathway	16/12756	0.792089862	0.999994594	0.94442346	Soltu.DM.12G025260	1
1971	GO:0097193	intrinsic apoptotic signaling pathway	16/12756	0.792089862	0.999994594	0.94442346	Soltu.DM.12G025260	1
1972	GO:1902652	secondary alcohol metabolic process	16/12	0.7920	0.9999	0.9444	Soltu.DM.07G017750	1

			756	89862	94594	2346		
1973	GO:0009127	purine nucleoside monophosphate biosynthetic process	57/12756	0.792324978	0.999994594	0.944423446	Soltu.DM.12G004480/Soltu.DM.07G009580/Soltu.DM.06G013720/Soltu.DM.11G025570	4
1974	GO:0009168	purine ribonucleoside monophosphate biosynthetic process	57/12756	0.792324978	0.999994594	0.944423446	Soltu.DM.12G004480/Soltu.DM.07G009580/Soltu.DM.06G013720/Soltu.DM.11G025570	4
1975	GO:0051960	regulation of nervous system development	57/12756	0.792324978	0.999994594	0.944423446	Soltu.DM.12G020370/Soltu.DM.12G020350/Soltu.DM.12G025260/Soltu.DM.12G020340	4
1976	GO:0060341	regulation of cellular localization	131/12756	0.792441043	0.999994594	0.944423446	Soltu.DM.12G020370/Soltu.DM.12G020350/Soltu.DM.08G027150/Soltu.DM.07G002400/Soltu.DM.01G002690/Soltu.DM.08G001690/Soltu.DM.09G002090/Soltu.DM.12G020340/Soltu.DM.02G022620/Soltu.DM.02G030830	10
1977	GO:0009895	negative regulation of catabolic process	44/12756	0.792486085	0.999994594	0.944423446	Soltu.DM.09G014740/Soltu.DM.11G016820/Soltu.DM.01G002690	3
1978	GO:0044770	cell cycle phase transition	44/12756	0.792486085	0.999994594	0.944423446	Soltu.DM.03G003730/Soltu.DM.02G029740/Soltu.DM.01G047090	3
1979	GO:1901991	negative regulation of mitotic cell cycle phase transition	44/12756	0.792486085	0.999994594	0.944423446	Soltu.DM.10G020590/Soltu.DM.11G016820/Soltu.DM.02G033290	3
1980	GO:0030030	cell projection organization	70/12756	0.795276233	0.999994594	0.944423446	Soltu.DM.01G028770/Soltu.DM.09G019870/Soltu.DM.01G020880/Soltu.DM.07G024370/Soltu.DM.09G027230	5
1981	GO:0043488	regulation of mRNA stability	70/12756	0.795276233	0.999994594	0.944423446	Soltu.DM.05G004270/Soltu.DM.08G013620/Soltu.DM.05G026810/Soltu.DM.11G016820/Soltu.DM.04G031030	5
1982	GO:0051607	defense response to virus	70/12756	0.795276233	0.999994594	0.944423446	Soltu.DM.10G025390/Soltu.DM.02G012280/Soltu.DM.01G003040/Soltu.DM.01G010020/Soltu.DM.11G004150	5
1983	GO:0140546	defense response to symbiont	70/12756	0.795276233	0.999994594	0.944423446	Soltu.DM.10G025390/Soltu.DM.02G012280/Soltu.DM.01G003040/Soltu.DM.01G010020/Soltu.DM.11G004150	5
1984	GO:0009141	nucleoside triphosphate metabolic process	95/12756	0.79613991	0.999994594	0.944423446	Soltu.DM.12G004480/Soltu.DM.02G018700/Soltu.DM.07G009580/Soltu.DM.10G027910/Soltu.DM.06G013720/Soltu.DM.01G017170/Soltu.DM.11G025570	7
1985	GO:0006813	potassium ion transport	83/12756	0.799712758	0.999994594	0.944423446	Soltu.DM.01G035900/Soltu.DM.10G010160/Soltu.DM.12G024710/Soltu.DM.08G018550/Soltu.DM.01G035910/Soltu.DM.02G013580	6
1986	GO:0000460	maturation of 5.8S rRNA	31/12756	0.799920532	0.999994594	0.944423446	Soltu.DM.12G024350/Soltu.DM.01G051600	2
1987	GO:0030855	epithelial cell differentiation	31/12756	0.799920532	0.999994594	0.944423446	Soltu.DM.10G022360/Soltu.DM.10G023760	2
1988	GO:0044070	regulation of monoatomic anion transport	31/12756	0.799920532	0.999994594	0.944423446	Soltu.DM.02G030340/Soltu.DM.11G004950	2
1989	GO:0046834	lipid phosphorylation	31/12756	0.799920532	0.999994594	0.944423446	Soltu.DM.02G018520/Soltu.DM.05G001470	2
19	GO:00	regulation of mRNA splicing, via	31/	0.7	0.9	0.9	Soltu.DM.12G025260/Soltu.DM.10G001400	2

90	48024	spliceosome	12756	99920532	99994594	4442346			
1991	GO:0051052	regulation of DNA metabolic process	156/12756	0.800188811	0.999994594	0.94442346		Soltu.DM.06G026960/Soltu.DM.11G009630/Soltu.DM.08G027160/Soltu.DM.04G034330/Soltu.DM.09G024860/Soltu.DM.02G001620/Soltu.DM.02G001630/Soltu.DM.03G032560/Soltu.DM.05G006310/Soltu.DM.01G002690/Soltu.DM.12G030150/Soltu.DM.02G013390	12
1992	GO:0003002	regionalization	180/12756	0.801722966	0.999994594	0.94442346		Soltu.DM.10G023790/Soltu.DM.12G005490/Soltu.DM.02G003130/Soltu.DM.05G003100/Soltu.DM.04G006620/Soltu.DM.09G020190/Soltu.DM.11G007750/Soltu.DM.12G010960/Soltu.DM.04G029270/Soltu.DM.03G024040/Soltu.DM.07G026690/Soltu.DM.08G012010/Soltu.DM.04G021540/Soltu.DM.04G031030	14
1993	GO:0016114	terpenoid biosynthetic process	192/12756	0.802840076	0.999994594	0.94442346		Soltu.DM.11G024450/Soltu.DM.11G004760/Soltu.DM.07G020920/Soltu.DM.02G011120/Soltu.DM.10G024780/Soltu.DM.08G028310/Soltu.DM.06G012790/Soltu.DM.08G013420/Soltu.DM.06G004460/Soltu.DM.06G029640/Soltu.DM.12G026560/Soltu.DM.10G003570/Soltu.DM.10G022070/Soltu.DM.06G023440/Soltu.DM.10G003550	15
1994	GO:0009798	axis specification	58/12756	0.803496799	0.999994594	0.94442346		Soltu.DM.02G003130/Soltu.DM.07G026690/Soltu.DM.08G012010/Soltu.DM.04G002690	4
1995	GO:0043331	response to dsRNA	96/12756	0.804880964	0.999994594	0.94442346		Soltu.DM.12G005490/Soltu.DM.09G024860/Soltu.DM.12G026070/Soltu.DM.11G025410/Soltu.DM.01G010020/Soltu.DM.04G031030/Soltu.DM.11G004150	7
1996	GO:0009911	positive regulation of flower development	45/12756	0.805028567	0.999994594	0.94442346		Soltu.DM.06G019760/Soltu.DM.08G001470/Soltu.DM.03G024000	3
1997	GO:0061013	regulation of mRNA catabolic process	71/12756	0.805357332	0.999994594	0.94442346		Soltu.DM.05G004270/Soltu.DM.08G013620/Soltu.DM.05G026810/Soltu.DM.11G016820/Soltu.DM.04G031030	5
1998	GO:0048527	lateral root development	109/12756	0.810393245	0.999994594	0.94442346		Soltu.DM.02G006700/Soltu.DM.12G004060/Soltu.DM.08G023320/Soltu.DM.11G018040/Soltu.DM.06G020260/Soltu.DM.12G029710/Soltu.DM.02G022410/Soltu.DM.06G034230	8
1999	GO:0001522	pseudouridine synthesis	17/12756	0.811542679	0.999994594	0.94442346		Soltu.DM.01G046920	1
2000	GO:0001678	cellular glucose homeostasis	17/12756	0.811542679	0.999994594	0.94442346		Soltu.DM.08G008380	1
2001	GO:0006354	DNA-templated transcription elongation	17/12756	0.811542679	0.999994594	0.94442346		Soltu.DM.11G008820	1
2002	GO:0007009	plasma membrane organization	17/12756	0.811542679	0.999994594	0.94442346		Soltu.DM.04G000190	1
2003	GO:0009804	coumarin metabolic process	17/12756	0.811542679	0.999994594	0.94442346		Soltu.DM.10G029960	1
2004	GO:0009805	coumarin biosynthetic process	17/12756	0.811542679	0.999994594	0.94442346		Soltu.DM.10G029960	1
2005	GO:0009902	chloroplast relocation	17/12756	0.811542679	0.999994594	0.94442346		Soltu.DM.10G001460	1
2006	GO:0010037	response to carbon dioxide	17/12756	0.811542679	0.999994594	0.94442346		Soltu.DM.07G013360	1
2007	GO:0010192	mucilage biosynthetic process	17/12756	0.811542679	0.999994594	0.94442346		Soltu.DM.02G027540	1

2008	GO:0010449	root meristem growth	17/12756	0.811542679	0.999994594	0.94442346	Soltu.DM.11G017980	1
2009	GO:0015919	peroxisomal membrane transport	17/12756	0.811542679	0.999994594	0.94442346	Soltu.DM.08G023320	1
2010	GO:0019218	regulation of steroid metabolic process	17/12756	0.811542679	0.999994594	0.94442346	Soltu.DM.10G004300	1
2011	GO:0030111	regulation of Wnt signaling pathway	17/12756	0.811542679	0.999994594	0.94442346	Soltu.DM.07G002580	1
2012	GO:0030834	regulation of actin filament depolymerization	17/12756	0.811542679	0.999994594	0.94442346	Soltu.DM.09G015150	1
2013	GO:0042775	mitochondrial ATP synthesis coupled electron transport	17/12756	0.811542679	0.999994594	0.94442346	Soltu.DM.02G018700	1
2014	GO:0045321	leukocyte activation	17/12756	0.811542679	0.999994594	0.94442346	Soltu.DM.10G022360	1
2015	GO:0048448	stamen morphogenesis	17/12756	0.811542679	0.999994594	0.94442346	Soltu.DM.05G003100	1
2016	GO:0050792	regulation of viral process	17/12756	0.811542679	0.999994594	0.94442346	Soltu.DM.05G026140	1
2017	GO:0051091	positive regulation of DNA-binding transcription factor activity	17/12756	0.811542679	0.999994594	0.94442346	Soltu.DM.02G018520	1
2018	GO:0051569	regulation of histone H3-K4 methylation	17/12756	0.811542679	0.999994594	0.94442346	Soltu.DM.12G025260	1
2019	GO:0051667	establishment of plastid localization	17/12756	0.811542679	0.999994594	0.94442346	Soltu.DM.10G001460	1
2020	GO:0072347	response to anesthetic	17/12756	0.811542679	0.999994594	0.94442346	Soltu.DM.07G013360	1
2021	GO:0099111	microtubule-based transport	17/12756	0.811542679	0.999994594	0.94442346	Soltu.DM.07G024370	1
2022	GO:1901880	negative regulation of protein depolymerization	17/12756	0.811542679	0.999994594	0.94442346	Soltu.DM.09G015150	1
2023	GO:0061014	positive regulation of mRNA catabolic process	59/12756	0.814175767	0.999994594	0.94442346	Soltu.DM.05G004270/Soltu.DM.08G013620/Soltu.DM.05G026810/Soltu.DM.04G031030	4
2024	GO:0006096	glycolytic process	32/12756	0.81419231	0.999994594	0.94442346	Soltu.DM.12G004480/Soltu.DM.06G013720	2
2025	GO:0006757	ATP generation from ADP	32/1275	0.8141923	0.9999945	0.9444234	Soltu.DM.12G004480/Soltu.DM.06G013720	2

			6	1	94	6		
2026	GO:0007062	sister chromatid cohesion	32/12756	0.81419231	0.999994594	0.94442346	Soltu.DM.06G019850/Soltu.DM.09G014080	2
2027	GO:0009070	serine family amino acid biosynthetic process	32/12756	0.81419231	0.999994594	0.94442346	Soltu.DM.08G030020/Soltu.DM.12G025770	2
2028	GO:0043255	regulation of carbohydrate biosynthetic process	32/12756	0.81419231	0.999994594	0.94442346	Soltu.DM.08G008380/Soltu.DM.01G024860	2
2029	GO:0055072	iron ion homeostasis	32/12756	0.81419231	0.999994594	0.94442346	Soltu.DM.04G003430/Soltu.DM.07G015200	2
2030	GO:0071230	cellular response to amino acid stimulus	32/12756	0.81419231	0.999994594	0.94442346	Soltu.DM.01G051900/Soltu.DM.02G022460	2
2031	GO:0009581	detection of external stimulus	46/12756	0.816926615	0.999994594	0.94442346	Soltu.DM.01G028770/Soltu.DM.07G015980/Soltu.DM.02G011380	3
2032	GO:0009582	detection of abiotic stimulus	46/12756	0.816926615	0.999994594	0.94442346	Soltu.DM.01G028770/Soltu.DM.07G015980/Soltu.DM.02G011380	3
2033	GO:2000377	regulation of reactive oxygen species metabolic process	46/12756	0.816926615	0.999994594	0.94442346	Soltu.DM.05G026160/Soltu.DM.08G013620/Soltu.DM.06G026400	3
2034	GO:0007346	regulation of mitotic cell cycle	159/12756	0.820239806	0.999994594	0.94442346	Soltu.DM.07G017210/Soltu.DM.07G017190/Soltu.DM.07G017200/Soltu.DM.02G018520/Soltu.DM.07G017180/Soltu.DM.02G028740/Soltu.DM.03G003730/Soltu.DM.10G020590/Soltu.DM.11G016820/Soltu.DM.02G033290/Soltu.DM.12G023230/Soltu.DM.03G034800	12
2035	GO:0006473	protein acetylation	60/12756	0.824374113	0.999994594	0.94442346	Soltu.DM.06G019850/Soltu.DM.04G033160/Soltu.DM.09G019870/Soltu.DM.02G027780	4
2036	GO:0006664	glycolipid metabolic process	60/12756	0.824374113	0.999994594	0.94442346	Soltu.DM.08G001900/Soltu.DM.01G042210/Soltu.DM.10G022360/Soltu.DM.02G010490	4
2037	GO:1903509	liposaccharide metabolic process	60/12756	0.824374113	0.999994594	0.94442346	Soltu.DM.08G001900/Soltu.DM.01G042210/Soltu.DM.10G022360/Soltu.DM.02G010490	4
2038	GO:0009123	nucleoside monophosphate metabolic process	111/12756	0.825784691	0.999994594	0.94442346	Soltu.DM.05G011440/Soltu.DM.12G004480/Soltu.DM.02G018700/Soltu.DM.07G009580/Soltu.DM.09G006670/Soltu.DM.06G013720/Soltu.DM.11G025570/Soltu.DM.05G023970	8
2039	GO:0006714	sesquiterpenoid metabolic process	86/12756	0.826296135	0.999994594	0.94442346	Soltu.DM.08G020150/Soltu.DM.11G024450/Soltu.DM.11G004760/Soltu.DM.07G013940/Soltu.DM.06G029640/Soltu.DM.07G013900	6
2040	GO:0006312	mitotic recombination	33/12756	0.827546657	0.999994594	0.94442346	Soltu.DM.09G025170/Soltu.DM.05G023970	2
2041	GO:0046031	ADP metabolic process	33/12756	0.827546657	0.999994594	0.94442346	Soltu.DM.12G004480/Soltu.DM.06G013720	2
2042	GO:0046898	response to cycloheximide	33/12756	0.827546657	0.999994594	0.94442346	Soltu.DM.10G020990/Soltu.DM.05G026870	2
2043	GO:0007093	mitotic cell cycle checkpoint signaling	47/12	0.8282	0.9999	0.9444	Soltu.DM.10G020590/Soltu.DM.11G016820/Soltu.DM.02G033290	3

			756	01846	94594	2346		
2044	GO:0009064	glutamine family amino acid metabolic process	47/12756	0.828201846	0.999994594	0.944423466	Soltu.DM.12G024030/Soltu.DM.08G007450/Soltu.DM.07G014750	3
2045	GO:0080086	stamen filament development	47/12756	0.828201846	0.999994594	0.944423466	Soltu.DM.03G037120/Soltu.DM.06G023410/Soltu.DM.11G001500	3
2046	GO:0000266	mitochondrial fission	18/12756	0.829176804	0.999994594	0.944423466	Soltu.DM.01G042120	1
2047	GO:0006101	citrate metabolic process	18/12756	0.829176804	0.999994594	0.944423466	Soltu.DM.07G017750	1
2048	GO:0009240	isopentenyl diphosphate biosynthetic process	18/12756	0.829176804	0.999994594	0.944423466	Soltu.DM.08G026810	1
2049	GO:0009862	systemic acquired resistance, salicylic acid mediated signaling pathway	18/12756	0.829176804	0.999994594	0.944423466	Soltu.DM.04G028540	1
2050	GO:0010222	stem vascular tissue pattern formation	18/12756	0.829176804	0.999994594	0.944423466	Soltu.DM.03G024040	1
2051	GO:0010227	floral organ abscission	18/12756	0.829176804	0.999994594	0.944423466	Soltu.DM.08G013580	1
2052	GO:0010359	regulation of anion channel activity	18/12756	0.829176804	0.999994594	0.944423466	Soltu.DM.11G004950	1
2053	GO:0010363	regulation of plant-type hypersensitive response	18/12756	0.829176804	0.999994594	0.944423466	Soltu.DM.08G022900	1
2054	GO:0010444	guard mother cell differentiation	18/12756	0.829176804	0.999994594	0.944423466	Soltu.DM.01G047090	1
2055	GO:0033046	negative regulation of sister chromatid segregation	18/12756	0.829176804	0.999994594	0.944423466	Soltu.DM.10G020590	1
2056	GO:0035266	meristem growth	18/12756	0.829176804	0.999994594	0.944423466	Soltu.DM.11G017980	1
2057	GO:0040017	positive regulation of locomotion	18/12756	0.829176804	0.999994594	0.944423466	Soltu.DM.02G018520	1
2058	GO:0043476	pigment accumulation	18/12756	0.829176804	0.999994594	0.944423466	Soltu.DM.06G005370	1
2059	GO:0046490	isopentenyl diphosphate metabolic process	18/12756	0.829176804	0.999994594	0.944423466	Soltu.DM.08G026810	1
2060	GO:0046856	phosphatidylinositol dephosphorylation	18/12756	0.829176804	0.999994594	0.944423466	Soltu.DM.04G034770	1
20	GO:00	negative regulation of chromosome	18/	0.8	0.9	0.9	Soltu.DM.10G020590	1

61	51985	segregation	12756	29176804	99994594	4442346		
2062	GO:0071474	cellular hyperosmotic response	18/12756	0.829176804	0.999994594	0.94442346	Soltu.DM.02G020550	1
2063	GO:1990778	protein localization to cell periphery	18/12756	0.829176804	0.999994594	0.94442346	Soltu.DM.04G022240	1
2064	GO:2000116	regulation of cysteine-type endopeptidase activity	18/12756	0.829176804	0.999994594	0.94442346	Soltu.DM.03G020450	1
2065	GO:0046474	glycerophospholipid biosynthetic process	74/12756	0.833246008	0.999994594	0.94442346	Soltu.DM.03G030800/Soltu.DM.02G010490/Soltu.DM.04G034770/Soltu.DM.05G001470/Soltu.DM.06G018040	5
2066	GO:0045814	negative regulation of gene expression, epigenetic	87/12756	0.834506752	0.999994594	0.94442346	Soltu.DM.04G033160/Soltu.DM.04G006870/Soltu.DM.10G024770/Soltu.DM.01G024940/Soltu.DM.06G001230/Soltu.DM.03G020440	6
2067	GO:0006109	regulation of carbohydrate metabolic process	48/12756	0.838876454	0.999994594	0.94442346	Soltu.DM.08G008380/Soltu.DM.07G019030/Soltu.DM.01G024860	3
2068	GO:0048481	plant ovule development	48/12756	0.838876454	0.999994594	0.94442346	Soltu.DM.10G023790/Soltu.DM.02G003130/Soltu.DM.01G000910	3
2069	GO:0070588	calcium ion transmembrane transport	48/12756	0.838876454	0.999994594	0.94442346	Soltu.DM.01G051900/Soltu.DM.02G022460/Soltu.DM.06G033030	3
2070	GO:0006625	protein targeting to peroxisome	34/12756	0.840030244	0.999994594	0.94442346	Soltu.DM.08G023320/Soltu.DM.10G004300	2
2071	GO:0008356	asymmetric cell division	34/12756	0.840030244	0.999994594	0.94442346	Soltu.DM.12G010960/Soltu.DM.10G024000	2
2072	GO:0009566	fertilization	34/12756	0.840030244	0.999994594	0.94442346	Soltu.DM.04G000190/Soltu.DM.05G004430	2
2073	GO:0016579	protein deubiquitination	34/12756	0.840030244	0.999994594	0.94442346	Soltu.DM.06G018150/Soltu.DM.02G030830	2
2074	GO:0043687	post-translational protein modification	34/12756	0.840030244	0.999994594	0.94442346	Soltu.DM.11G022310/Soltu.DM.01G013260	2
2075	GO:0051289	protein homotetramerization	34/12756	0.840030244	0.999994594	0.94442346	Soltu.DM.03G012810/Soltu.DM.08G030020	2
2076	GO:0060992	response to fungicide	34/12756	0.840030244	0.999994594	0.94442346	Soltu.DM.10G020990/Soltu.DM.05G026870	2
2077	GO:0072662	protein localization to peroxisome	34/12756	0.840030244	0.999994594	0.94442346	Soltu.DM.08G023320/Soltu.DM.10G004300	2
2078	GO:0072663	establishment of protein localization to peroxisome	34/12756	0.840030244	0.999994594	0.94442346	Soltu.DM.08G023320/Soltu.DM.10G004300	2

2079	GO:2000142	regulation of DNA-templated transcription initiation	34/12756	0.840030244	0.999994594	0.94442346	Soltu.DM.06G028040/Soltu.DM.03G022850	2
2080	GO:0000272	polysaccharide catabolic process	75/12756	0.841785431	0.999994594	0.94442346	Soltu.DM.09G027770/Soltu.DM.05G006330/Soltu.DM.11G000740/Soltu.DM.12G007130/Soltu.DM.04G037250	5
2081	GO:0009630	gravitropism	88/12756	0.842402518	0.999994594	0.94442346	Soltu.DM.01G035900/Soltu.DM.09G006890/Soltu.DM.01G035910/Soltu.DM.03G036780/Soltu.DM.06G002140/Soltu.DM.04G002690	6
2082	GO:0048871	multicellular organismal homeostasis	62/12756	0.843380905	0.999994594	0.94442346	Soltu.DM.08G027160/Soltu.DM.11G017980/Soltu.DM.05G023970/Soltu.DM.02G013390	4
2083	GO:0070085	glycosylation	175/12756	0.84375335	0.999994594	0.94442346	Soltu.DM.04G011110/Soltu.DM.02G025970/Soltu.DM.02G024810/Soltu.DM.03G037510/Soltu.DM.10G027470/Soltu.DM.04G011320/Soltu.DM.04G011240/Soltu.DM.10G028070/Soltu.DM.04G000320/Soltu.DM.04G011370/Soltu.DM.05G001020/Soltu.DM.06G021870/Soltu.DM.11G021090	13
2084	GO:0010638	positive regulation of organelle organization	101/12756	0.844320617	0.999994594	0.94442346	Soltu.DM.12G025260/Soltu.DM.03G003730/Soltu.DM.09G015150/Soltu.DM.01G002690/Soltu.DM.06G018040/Soltu.DM.09G027230/Soltu.DM.02G013390	7
2085	GO:0000578	embryonic axis specification	19/12756	0.845162143	0.999994594	0.94442346	Soltu.DM.04G002690	1
2086	GO:0002183	cytoplasmic translational initiation	19/12756	0.845162143	0.999994594	0.94442346	Soltu.DM.08G019530	1
2087	GO:0002697	regulation of immune effector process	19/12756	0.845162143	0.999994594	0.94442346	Soltu.DM.03G021360	1
2088	GO:0006817	phosphate ion transport	19/12756	0.845162143	0.999994594	0.94442346	Soltu.DM.05G021160	1
2089	GO:0008037	cell recognition	19/12756	0.845162143	0.999994594	0.94442346	Soltu.DM.04G000190	1
2090	GO:0015770	sucrose transport	19/12756	0.845162143	0.999994594	0.94442346	Soltu.DM.03G020090	1
2091	GO:0015914	phospholipid transport	19/12756	0.845162143	0.999994594	0.94442346	Soltu.DM.06G022490	1
2092	GO:0016075	rRNA catabolic process	19/12756	0.845162143	0.999994594	0.94442346	Soltu.DM.09G014740	1
2093	GO:0019682	glyceraldehyde-3-phosphate metabolic process	19/12756	0.845162143	0.999994594	0.94442346	Soltu.DM.08G014620	1
2094	GO:0019750	chloroplast localization	19/12756	0.845162143	0.999994594	0.94442346	Soltu.DM.10G001460	1
2095	GO:0030968	endoplasmic reticulum unfolded protein response	19/12756	0.845162143	0.999994594	0.94442346	Soltu.DM.08G019590	1
2096	GO:0031537	regulation of anthocyanin metabolic process	19/1275	0.8451621	0.9999945	0.9444234	Soltu.DM.12G026600	1

			6	43	94	6		
2097	GO:0032875	regulation of DNA endoreduplication	19/12756	0.845162143	0.999994594	0.94442346	Soltu.DM.12G030150	1
2098	GO:0045839	negative regulation of mitotic nuclear division	19/12756	0.845162143	0.999994594	0.94442346	Soltu.DM.10G020590	1
2099	GO:0051644	plastid localization	19/12756	0.845162143	0.999994594	0.94442346	Soltu.DM.10G001460	1
2100	GO:0051650	establishment of vesicle localization	19/12756	0.845162143	0.999994594	0.94442346	Soltu.DM.11G026460	1
2101	GO:0071345	cellular response to cytokine stimulus	19/12756	0.845162143	0.999994594	0.94442346	Soltu.DM.08G013620	1
2102	GO:1902930	regulation of alcohol biosynthetic process	19/12756	0.845162143	0.999994594	0.94442346	Soltu.DM.02G020950	1
2103	GO:0000070	mitotic sister chromatid segregation	49/12756	0.848973001	0.999994594	0.94442346	Soltu.DM.06G019850/Soltu.DM.09G014080/Soltu.DM.01G043730	3
2104	GO:0006661	phosphatidylinositol biosynthetic process	49/12756	0.848973001	0.999994594	0.94442346	Soltu.DM.02G010490/Soltu.DM.04G034770/Soltu.DM.05G001470	3
2105	GO:0071900	regulation of protein serine/threonine kinase activity	49/12756	0.848973001	0.999994594	0.94442346	Soltu.DM.06G018320/Soltu.DM.11G010230/Soltu.DM.11G010220	3
2106	GO:0044089	positive regulation of cellular component biogenesis	89/12756	0.849990185	0.999994594	0.94442346	Soltu.DM.09G015150/Soltu.DM.11G011390/Soltu.DM.11G024760/Soltu.DM.01G027520/Soltu.DM.09G027230/Soltu.DM.12G023230	6
2107	GO:0007610	behavior	35/12756	0.851689037	0.999994594	0.94442346	Soltu.DM.06G005370/Soltu.DM.10G016030	2
2108	GO:0042866	pyruvate biosynthetic process	35/12756	0.851689037	0.999994594	0.94442346	Soltu.DM.12G004480/Soltu.DM.06G013720	2
2109	GO:0090316	positive regulation of intracellular protein transport	35/12756	0.851689037	0.999994594	0.94442346	Soltu.DM.07G002400/Soltu.DM.08G001690	2
2110	GO:0048588	developmental cell growth	165/12756	0.855766424	0.999994594	0.94442346	Soltu.DM.10G024410/Soltu.DM.02G031090/Soltu.DM.08G027650/Soltu.DM.09G005320/Soltu.DM.12G005490/Soltu.DM.08G023170/Soltu.DM.12G024710/Soltu.DM.09G026810/Soltu.DM.12G024030/Soltu.DM.10G002320/Soltu.DM.06G023200/Soltu.DM.04G002690	12
2111	GO:1902531	regulation of intracellular signal transduction	165/12756	0.855766424	0.999994594	0.94442346	Soltu.DM.02G018520/Soltu.DM.11G010230/Soltu.DM.07G020920/Soltu.DM.10G024780/Soltu.DM.11G010220/Soltu.DM.06G014700/Soltu.DM.08G013420/Soltu.DM.08G013440/Soltu.DM.10G022070/Soltu.DM.11G015370/Soltu.DM.07G022640/Soltu.DM.05G025620	12
2112	GO:0001558	regulation of cell growth	128/12756	0.856418304	0.999994594	0.94442346	Soltu.DM.12G020370/Soltu.DM.02G018520/Soltu.DM.12G020350/Soltu.DM.01G039230/Soltu.DM.02G030780/Soltu.DM.12G026380/Soltu.DM.08G012010/Soltu.DM.06G023200/Soltu.DM.12G020340	9
2113	GO:0043254	regulation of protein-containing complex assembly	128/12756	0.856418304	0.999994594	0.94442346	Soltu.DM.06G005370/Soltu.DM.06G022970/Soltu.DM.06G028040/Soltu.DM.01G028770/Soltu.DM.03G022850/Soltu.DM.09G015150/Soltu.DM.12G009990/Soltu.DM.10G004310/Soltu.DM.09G027230	9
2114	GO:0022008	neurogenesis	90/12	0.8572	0.9999	0.9444	Soltu.DM.12G020370/Soltu.DM.01G042210/Soltu.DM.12G020350/Soltu.DM.12G025260/Soltu.DM.09G019870/Soltu.DM.12G020340	6

			756	76786	94594	2346		
2115	GO:0046034	ATP metabolic process	77/12756	0.857784067	0.999994594	0.94442346	Soltu.DM.12G004480/Soltu.DM.02G018700/Soltu.DM.07G009580/Soltu.DM.06G013720/Soltu.DM.11G025570	5
2116	GO:0048284	organelle fusion	77/12756	0.857784067	0.999994594	0.94442346	Soltu.DM.03G017520/Soltu.DM.05G023990/Soltu.DM.02G012680/Soltu.DM.01G002850/Soltu.DM.12G002630	5
2117	GO:0009606	tropism	103/12756	0.85819343	0.999994594	0.94442346	Soltu.DM.01G035900/Soltu.DM.07G020410/Soltu.DM.09G006890/Soltu.DM.01G035910/Soltu.DM.03G036780/Soltu.DM.06G002140/Soltu.DM.04G002690	7
2118	GO:0051302	regulation of cell division	103/12756	0.85819343	0.999994594	0.94442346	Soltu.DM.07G020920/Soltu.DM.10G024780/Soltu.DM.08G013420/Soltu.DM.10G022070/Soltu.DM.08G012010/Soltu.DM.10G024000/Soltu.DM.12G023230	7
2119	GO:0035670	plant-type ovary development	50/12756	0.858514249	0.999994594	0.94442346	Soltu.DM.10G023790/Soltu.DM.02G003130/Soltu.DM.01G000910	3
2120	GO:0006873	cellular monoatomic ion homeostasis	141/12756	0.859538317	0.999994594	0.94442346	Soltu.DM.02G034460/Soltu.DM.07G002440/Soltu.DM.03G017590/Soltu.DM.02G024200/Soltu.DM.05G021830/Soltu.DM.04G003430/Soltu.DM.11G000570/Soltu.DM.07G009580/Soltu.DM.10G004300/Soltu.DM.08G001690	10
2121	GO:0000028	ribosomal small subunit assembly	20/12756	0.85965274	0.999994594	0.94442346	Soltu.DM.01G051600	1
2122	GO:0006739	NADP metabolic process	20/12756	0.85965274	0.999994594	0.94442346	Soltu.DM.08G014620	1
2123	GO:0008544	epidermis development	20/12756	0.85965274	0.999994594	0.94442346	Soltu.DM.10G023760	1
2124	GO:0009913	epidermal cell differentiation	20/12756	0.85965274	0.999994594	0.94442346	Soltu.DM.10G023760	1
2125	GO:0010052	guard cell differentiation	20/12756	0.85965274	0.999994594	0.94442346	Soltu.DM.05G021390	1
2126	GO:0010972	negative regulation of G2/M transition of mitotic cell cycle	20/12756	0.85965274	0.999994594	0.94442346	Soltu.DM.02G033290	1
2127	GO:0015766	disaccharide transport	20/12756	0.85965274	0.999994594	0.94442346	Soltu.DM.03G020090	1
2128	GO:0015772	oligosaccharide transport	20/12756	0.85965274	0.999994594	0.94442346	Soltu.DM.03G020090	1
2129	GO:0019252	starch biosynthetic process	20/12756	0.85965274	0.999994594	0.94442346	Soltu.DM.01G051470	1
2130	GO:0030155	regulation of cell adhesion	20/12756	0.85965274	0.999994594	0.94442346	Soltu.DM.08G012010	1
2131	GO:0030308	negative regulation of cell growth	20/12756	0.85965274	0.999994594	0.94442346	Soltu.DM.01G039230	1
21	GO:00	mitochondrial translation	20/	0.8	0.9	0.9	Soltu.DM.12G028920	1

32	32543		12756	5965274	99994594	4442346		
2133	GO:0043242	negative regulation of protein-containing complex disassembly	20/12756	0.85965274	0.999994594	0.94442346	Soltu.DM.09G015150	1
2134	GO:0098659	inorganic cation import across plasma membrane	20/12756	0.85965274	0.999994594	0.94442346	Soltu.DM.12G024710	1
2135	GO:0099587	inorganic ion import across plasma membrane	20/12756	0.85965274	0.999994594	0.94442346	Soltu.DM.12G024710	1
2136	GO:1901879	regulation of protein depolymerization	20/12756	0.85965274	0.999994594	0.94442346	Soltu.DM.09G015150	1
2137	GO:1902750	negative regulation of cell cycle G2/M phase transition	20/12756	0.85965274	0.999994594	0.94442346	Soltu.DM.02G033290	1
2138	GO:0009156	ribonucleoside monophosphate biosynthetic process	64/12756	0.860625562	0.999994594	0.94442346	Soltu.DM.12G004480/Soltu.DM.07G009580/Soltu.DM.06G013720/Soltu.DM.11G025570	4
2139	GO:0046496	nicotinamide nucleotide metabolic process	64/12756	0.860625562	0.999994594	0.94442346	Soltu.DM.08G014620/Soltu.DM.12G004480/Soltu.DM.02G018700/Soltu.DM.06G013720	4
2140	GO:0051170	import into nucleus	64/12756	0.860625562	0.999994594	0.94442346	Soltu.DM.07G020410/Soltu.DM.03G003730/Soltu.DM.04G011330/Soltu.DM.07G006510	4
2141	GO:0030003	cellular monoatomic cation homeostasis	129/12756	0.86240712	0.999994594	0.94442346	Soltu.DM.07G002440/Soltu.DM.03G017590/Soltu.DM.02G024200/Soltu.DM.05G021830/Soltu.DM.04G003430/Soltu.DM.11G000570/Soltu.DM.07G009580/Soltu.DM.10G004300/Soltu.DM.08G001690	9
2142	GO:0008360	regulation of cell shape	36/12756	0.862568024	0.999994594	0.94442346	Soltu.DM.01G028770/Soltu.DM.09G027230	2
2143	GO:0009135	purine nucleoside diphosphate metabolic process	36/12756	0.862568024	0.999994594	0.94442346	Soltu.DM.12G004480/Soltu.DM.06G013720	2
2144	GO:0009179	purine ribonucleoside diphosphate metabolic process	36/12756	0.862568024	0.999994594	0.94442346	Soltu.DM.12G004480/Soltu.DM.06G013720	2
2145	GO:0009185	ribonucleoside diphosphate metabolic process	36/12756	0.862568024	0.999994594	0.94442346	Soltu.DM.12G004480/Soltu.DM.06G013720	2
2146	GO:0043574	peroxisomal transport	36/12756	0.862568024	0.999994594	0.94442346	Soltu.DM.08G023320/Soltu.DM.10G004300	2
2147	GO:0009926	auxin polar transport	104/12756	0.864743266	0.999994594	0.94442346	Soltu.DM.01G035900/Soltu.DM.02G032050/Soltu.DM.10G026500/Soltu.DM.01G035910/Soltu.DM.11G001500/Soltu.DM.08G001470/Soltu.DM.04G002690	7
2148	GO:0016573	histone acetylation	51/12756	0.867522997	0.999994594	0.94442346	Soltu.DM.06G019850/Soltu.DM.04G033160/Soltu.DM.09G019870	3
2149	GO:0051222	positive regulation of protein transport	51/12756	0.867522997	0.999994594	0.94442346	Soltu.DM.07G002400/Soltu.DM.01G002690/Soltu.DM.08G001690	3

2150	GO:0019362	pyridine nucleotide metabolic process	65/12756	0.868622366	0.999994594	0.94442346	Soltu.DM.08G014620/Soltu.DM.12G004480/Soltu.DM.02G018700/Soltu.DM.06G013720	4
2151	GO:0043543	protein acylation	65/12756	0.868622366	0.999994594	0.94442346	Soltu.DM.06G019850/Soltu.DM.04G033160/Soltu.DM.09G019870/Soltu.DM.02G027780	4
2152	GO:0120036	plasma membrane bounded cell projection organization	65/12756	0.868622366	0.999994594	0.94442346	Soltu.DM.01G028770/Soltu.DM.09G019870/Soltu.DM.01G020880/Soltu.DM.07G024370	4
2153	GO:0009101	glycoprotein biosynthetic process	168/12756	0.871346231	0.999994594	0.94442346	Soltu.DM.04G011110/Soltu.DM.02G025970/Soltu.DM.02G024810/Soltu.DM.03G037510/Soltu.DM.10G027470/Soltu.DM.04G011320/Soltu.DM.04G011240/Soltu.DM.10G028070/Soltu.DM.04G000320/Soltu.DM.04G011370/Soltu.DM.05G001020/Soltu.DM.11G021090	12
2154	GO:0098657	import into cell	118/12756	0.872087062	0.999994594	0.94442346	Soltu.DM.04G031760/Soltu.DM.12G006380/Soltu.DM.12G024710/Soltu.DM.09G000440/Soltu.DM.02G032050/Soltu.DM.10G023680/Soltu.DM.01G042120/Soltu.DM.08G001690	8
2155	GO:0009880	embryonic pattern specification	37/12756	0.872710993	0.999994594	0.94442346	Soltu.DM.02G003130/Soltu.DM.04G002690	2
2156	GO:0010027	thylakoid membrane organization	37/12756	0.872710993	0.999994594	0.94442346	Soltu.DM.08G001900/Soltu.DM.08G011890	2
2157	GO:0006022	aminoglycan metabolic process	21/12756	0.872788256	0.999994594	0.94442346	Soltu.DM.02G031050	1
2158	GO:0006284	base-excision repair	21/12756	0.872788256	0.999994594	0.94442346	Soltu.DM.05G007660	1
2159	GO:0009086	methionine biosynthetic process	21/12756	0.872788256	0.999994594	0.94442346	Soltu.DM.08G030020	1
2160	GO:0009875	pollen-pistil interaction	21/12756	0.872788256	0.999994594	0.94442346	Soltu.DM.12G024030	1
2161	GO:0010082	regulation of root meristem growth	21/12756	0.872788256	0.999994594	0.94442346	Soltu.DM.05G024870	1
2162	GO:0017062	respiratory chain complex III assembly	21/12756	0.872788256	0.999994594	0.94442346	Soltu.DM.02G006310	1
2163	GO:0034551	mitochondrial respiratory chain complex III assembly	21/12756	0.872788256	0.999994594	0.94442346	Soltu.DM.02G006310	1
2164	GO:0044030	regulation of DNA methylation	21/12756	0.872788256	0.999994594	0.94442346	Soltu.DM.09G024860	1
2165	GO:0055062	phosphate ion homeostasis	21/12756	0.872788256	0.999994594	0.94442346	Soltu.DM.02G034460	1
2166	GO:0072350	tricarboxylic acid metabolic process	21/12756	0.872788256	0.999994594	0.94442346	Soltu.DM.07G017750	1
2167	GO:0080024	indolebutyric acid metabolic process	21/1275	0.8727882	0.9999945	0.9444234	Soltu.DM.01G038470	1

			6	56	94	6		
2168	GO:1901796	regulation of signal transduction by p53 class mediator	21/12756	0.872788256	0.999994594	0.94442346	Soltu.DM.11G015370	1
2169	GO:0006816	calcium ion transport	52/12756	0.876021952	0.999994594	0.94442346	Soltu.DM.01G051900/Soltu.DM.02G022460/Soltu.DM.06G033030	3
2170	GO:0018393	internal peptidyl-lysine acetylation	52/12756	0.876021952	0.999994594	0.94442346	Soltu.DM.06G019850/Soltu.DM.04G033160/Soltu.DM.09G019870	3
2171	GO:0018394	peptidyl-lysine acetylation	52/12756	0.876021952	0.999994594	0.94442346	Soltu.DM.06G019850/Soltu.DM.04G033160/Soltu.DM.09G019870	3
2172	GO:0006289	nucleotide-excision repair	66/12756	0.876221313	0.999994594	0.94442346	Soltu.DM.08G027160/Soltu.DM.05G007660/Soltu.DM.07G017530/Soltu.DM.05G018370	4
2173	GO:0009933	meristem structural organization	66/12756	0.876221313	0.999994594	0.94442346	Soltu.DM.02G003130/Soltu.DM.05G026810/Soltu.DM.06G034230/Soltu.DM.03G034800	4
2174	GO:0045860	positive regulation of protein kinase activity	80/12756	0.87922878	0.999994594	0.94442346	Soltu.DM.07G017210/Soltu.DM.07G017190/Soltu.DM.07G017200/Soltu.DM.07G017180/Soltu.DM.02G028740	5
2175	GO:1901654	response to ketone	38/12756	0.882160366	0.999994594	0.94442346	Soltu.DM.10G020990/Soltu.DM.05G026870	2
2176	GO:0009860	pollen tube growth	107/12756	0.88291666	0.999994594	0.94442346	Soltu.DM.10G024410/Soltu.DM.02G031090/Soltu.DM.09G005320/Soltu.DM.09G026810/Soltu.DM.12G024030/Soltu.DM.10G002320/Soltu.DM.06G023200	7
2177	GO:0001934	positive regulation of protein phosphorylation	94/12756	0.88356046	0.999994594	0.94442346	Soltu.DM.07G017210/Soltu.DM.07G017190/Soltu.DM.07G017200/Soltu.DM.02G018520/Soltu.DM.07G017180/Soltu.DM.02G028740	6
2178	GO:0006475	internal protein amino acid acetylation	53/12756	0.884033608	0.999994594	0.94442346	Soltu.DM.06G019850/Soltu.DM.04G033160/Soltu.DM.09G019870	3
2179	GO:0044786	cell cycle DNA replication	53/12756	0.884033608	0.999994594	0.94442346	Soltu.DM.08G027160/Soltu.DM.12G029710/Soltu.DM.03G018740	3
2180	GO:0045930	negative regulation of mitotic cell cycle	53/12756	0.884033608	0.999994594	0.94442346	Soltu.DM.10G020590/Soltu.DM.11G016820/Soltu.DM.02G033290	3
2181	GO:1904951	positive regulation of establishment of protein localization	53/12756	0.884033608	0.999994594	0.94442346	Soltu.DM.07G002400/Soltu.DM.01G002690/Soltu.DM.08G001690	3
2182	GO:2001252	positive regulation of chromosome organization	53/12756	0.884033608	0.999994594	0.94442346	Soltu.DM.12G025260/Soltu.DM.01G002690/Soltu.DM.02G013390	3
2183	GO:0000730	DNA recombinase assembly	22/12756	0.884695315	0.999994594	0.94442346	Soltu.DM.09G025170	1
2184	GO:0006826	iron ion transport	22/12756	0.884695315	0.999994594	0.94442346	Soltu.DM.04G003430	1
2185	GO:0006879	cellular iron ion homeostasis	22/12	0.8846	0.9999	0.9444	Soltu.DM.04G003430	1

			756	95315	94594	2346		
2186	GO:0010588	cotyledon vascular tissue pattern formation	22/12756	0.884695315	0.999994594	0.94442346	Soltu.DM.03G024040	1
2187	GO:0016122	xanthophyll metabolic process	22/12756	0.884695315	0.999994594	0.94442346	Soltu.DM.06G029640	1
2188	GO:0043009	chordate embryonic development	22/12756	0.884695315	0.999994594	0.94442346	Soltu.DM.02G029740	1
2189	GO:0046131	pyrimidine ribonucleoside metabolic process	22/12756	0.884695315	0.999994594	0.94442346	Soltu.DM.01G017170	1
2190	GO:0090615	mitochondrial mRNA processing	22/12756	0.884695315	0.999994594	0.94442346	Soltu.DM.06G002320	1
2191	GO:0090735	DNA repair complex assembly	22/12756	0.884695315	0.999994594	0.94442346	Soltu.DM.09G025170	1
2192	GO:0097502	mannosylation	22/12756	0.884695315	0.999994594	0.94442346	Soltu.DM.06G021870	1
2193	GO:1900056	negative regulation of leaf senescence	22/12756	0.884695315	0.999994594	0.94442346	Soltu.DM.07G014750	1
2194	GO:1905622	negative regulation of leaf development	22/12756	0.884695315	0.999994594	0.94442346	Soltu.DM.07G014750	1
2195	GO:0006997	nucleus organization	81/12756	0.885736646	0.999994594	0.94442346	Soltu.DM.05G023990/Soltu.DM.02G012680/Soltu.DM.01G002850/Soltu.DM.12G002630/Soltu.DM.04G011330	5
2196	GO:0033674	positive regulation of kinase activity	81/12756	0.885736646	0.999994594	0.94442346	Soltu.DM.07G017210/Soltu.DM.07G017190/Soltu.DM.07G017200/Soltu.DM.07G017180/Soltu.DM.02G028740	5
2197	GO:0019220	regulation of phosphate metabolic process	184/12756	0.888075846	0.999994594	0.94442346	Soltu.DM.07G017210/Soltu.DM.07G017190/Soltu.DM.07G017200/Soltu.DM.02G018520/Soltu.DM.07G017180/Soltu.DM.07G012130/Soltu.DM.06G018320/Soltu.DM.11G010230/Soltu.DM.02G028740/Soltu.DM.11G010220/Soltu.DM.06G028580/Soltu.DM.04G038280/Soltu.DM.01G051340	13
2198	GO:0051174	regulation of phosphorus metabolic process	184/12756	0.888075846	0.999994594	0.94442346	Soltu.DM.07G017210/Soltu.DM.07G017190/Soltu.DM.07G017200/Soltu.DM.02G018520/Soltu.DM.07G017180/Soltu.DM.07G012130/Soltu.DM.06G018320/Soltu.DM.11G010230/Soltu.DM.02G028740/Soltu.DM.11G010220/Soltu.DM.06G028580/Soltu.DM.04G038280/Soltu.DM.01G051340	13
2199	GO:0016441	post-transcriptional gene silencing	108/12756	0.888502664	0.999994594	0.94442346	Soltu.DM.12G005490/Soltu.DM.05G026810/Soltu.DM.12G026070/Soltu.DM.11G025410/Soltu.DM.01G010020/Soltu.DM.04G031030/Soltu.DM.11G004150	7
2200	GO:0007033	vacuole organization	95/12756	0.88945398	0.999994594	0.94442346	Soltu.DM.06G005370/Soltu.DM.01G039130/Soltu.DM.01G028770/Soltu.DM.11G022310/Soltu.DM.09G025980/Soltu.DM.08G026700	6
2201	GO:0042327	positive regulation of phosphorylation	95/12756	0.88945398	0.999994594	0.94442346	Soltu.DM.07G017210/Soltu.DM.07G017190/Soltu.DM.07G017200/Soltu.DM.02G018520/Soltu.DM.07G017180/Soltu.DM.02G028740	6
2202	GO:0000226	microtubule cytoskeleton organization	172/12756	0.889999322	0.999994594	0.94442346	Soltu.DM.04G027320/Soltu.DM.12G007610/Soltu.DM.01G039760/Soltu.DM.02G028740/Soltu.DM.03G003730/Soltu.DM.01G008180/Soltu.DM.02G030630/Soltu.DM.04G022240/Soltu.DM.01G020880/Soltu.DM.05G009320/Soltu.DM.11G018040/Soltu.DM.03G035510	12
22	GO:00	nucleoside monophosphate	68/	0.8	0.9	0.9	Soltu.DM.12G004480/Soltu.DM.07G009580/Soltu.DM.06G013720/Soltu.DM.11G025570	4

03	09124	biosynthetic process	12756	902833 95	999945 94	444234 6		
2204	GO:0010101	post-embryonic root morphogenesis	68/12756	0.890283395	0.999994594	0.94442346	Soltu.DM.02G006700/Soltu.DM.11G018040/Soltu.DM.06G020260/Soltu.DM.02G022410	4
2205	GO:0010102	lateral root morphogenesis	68/12756	0.890283395	0.999994594	0.94442346	Soltu.DM.02G006700/Soltu.DM.11G018040/Soltu.DM.06G020260/Soltu.DM.02G022410	4
2206	GO:0072524	pyridine-containing compound metabolic process	68/12756	0.890283395	0.999994594	0.94442346	Soltu.DM.08G014620/Soltu.DM.12G004480/Soltu.DM.02G018700/Soltu.DM.06G013720	4
2207	GO:0006165	nucleoside diphosphate phosphorylation	39/12756	0.890957071	0.999994594	0.94442346	Soltu.DM.12G004480/Soltu.DM.06G013720	2
2208	GO:0043068	positive regulation of programmed cell death	39/12756	0.890957071	0.999994594	0.94442346	Soltu.DM.01G044520/Soltu.DM.07G014680	2
2209	GO:0048666	neuron development	39/12756	0.890957071	0.999994594	0.94442346	Soltu.DM.01G042210/Soltu.DM.09G019870	2
2210	GO:0002229	defense response to oomycetes	54/12756	0.891580149	0.999994594	0.94442346	Soltu.DM.06G028410/Soltu.DM.09G028710/Soltu.DM.05G023040	3
2211	GO:0002758	innate immune response-activating signaling pathway	54/12756	0.891580149	0.999994594	0.94442346	Soltu.DM.04G018070/Soltu.DM.08G011890/Soltu.DM.01G051770	3
2212	GO:0009629	response to gravity	96/12756	0.895092181	0.999994594	0.94442346	Soltu.DM.01G035900/Soltu.DM.09G006890/Soltu.DM.01G035910/Soltu.DM.03G036780/Soltu.DM.06G002140/Soltu.DM.04G002690	6
2213	GO:0010562	positive regulation of phosphorus metabolic process	96/12756	0.895092181	0.999994594	0.94442346	Soltu.DM.07G017210/Soltu.DM.07G017190/Soltu.DM.07G017200/Soltu.DM.02G018520/Soltu.DM.07G017180/Soltu.DM.02G028740	6
2214	GO:0045937	positive regulation of phosphate metabolic process	96/12756	0.895092181	0.999994594	0.94442346	Soltu.DM.07G017210/Soltu.DM.07G017190/Soltu.DM.07G017200/Soltu.DM.02G018520/Soltu.DM.07G017180/Soltu.DM.02G028740	6
2215	GO:0071466	cellular response to xenobiotic stimulus	148/12756	0.895307755	0.999994594	0.94442346	Soltu.DM.09G018910/Soltu.DM.07G028550/Soltu.DM.09G029050/Soltu.DM.10G000640/Soltu.DM.02G030410/Soltu.DM.02G006310/Soltu.DM.10G004300/Soltu.DM.04G028540/Soltu.DM.07G024240/Soltu.DM.06G026560	10
2216	GO:0001775	cell activation	23/12756	0.895488717	0.999994594	0.94442346	Soltu.DM.10G022360	1
2217	GO:0006505	GPI anchor metabolic process	23/12756	0.895488717	0.999994594	0.94442346	Soltu.DM.02G010490	1
2218	GO:0006506	GPI anchor biosynthetic process	23/12756	0.895488717	0.999994594	0.94442346	Soltu.DM.02G010490	1
2219	GO:0006607	NLS-bearing protein import into nucleus	23/12756	0.895488717	0.999994594	0.94442346	Soltu.DM.03G003730	1
2220	GO:0006722	triterpenoid metabolic process	23/12756	0.895488717	0.999994594	0.94442346	Soltu.DM.05G015440	1

2221	GO:0007600	sensory perception	23/12756	0.895488717	0.999994594	0.94442346	Soltu.DM.02G017970	1
2222	GO:0009937	regulation of gibberellic acid mediated signaling pathway	23/12756	0.895488717	0.999994594	0.94442346	Soltu.DM.09G022610	1
2223	GO:0010440	stomatal lineage progression	23/12756	0.895488717	0.999994594	0.94442346	Soltu.DM.01G047090	1
2224	GO:0010675	regulation of cellular carbohydrate metabolic process	23/12756	0.895488717	0.999994594	0.94442346	Soltu.DM.08G008380	1
2225	GO:0010965	regulation of mitotic sister chromatid separation	23/12756	0.895488717	0.999994594	0.94442346	Soltu.DM.10G020590	1
2226	GO:0030071	regulation of mitotic metaphase/anaphase transition	23/12756	0.895488717	0.999994594	0.94442346	Soltu.DM.10G020590	1
2227	GO:0031425	chloroplast RNA processing	23/12756	0.895488717	0.999994594	0.94442346	Soltu.DM.09G019220	1
2228	GO:0042148	strand invasion	23/12756	0.895488717	0.999994594	0.94442346	Soltu.DM.09G025170	1
2229	GO:0042593	glucose homeostasis	23/12756	0.895488717	0.999994594	0.94442346	Soltu.DM.08G008380	1
2230	GO:0042761	very long-chain fatty acid biosynthetic process	23/12756	0.895488717	0.999994594	0.94442346	Soltu.DM.10G026590	1
2231	GO:0042773	ATP synthesis coupled electron transport	23/12756	0.895488717	0.999994594	0.94442346	Soltu.DM.02G018700	1
2232	GO:0043692	monoterpene metabolic process	23/12756	0.895488717	0.999994594	0.94442346	Soltu.DM.07G026340	1
2233	GO:0043693	monoterpene biosynthetic process	23/12756	0.895488717	0.999994594	0.94442346	Soltu.DM.07G026340	1
2234	GO:0048568	embryonic organ development	23/12756	0.895488717	0.999994594	0.94442346	Soltu.DM.06G005370	1
2235	GO:0051781	positive regulation of cell division	23/12756	0.895488717	0.999994594	0.94442346	Soltu.DM.12G023230	1
2236	GO:1902099	regulation of metaphase/anaphase transition of cell cycle	23/12756	0.895488717	0.999994594	0.94442346	Soltu.DM.10G020590	1
2237	GO:1905818	regulation of chromosome separation	23/12756	0.895488717	0.999994594	0.94442346	Soltu.DM.10G020590	1
2238	GO:1901990	regulation of mitotic cell cycle phase transition	69/1275	0.8967752	0.9999945	0.9444234	Soltu.DM.10G020590/Soltu.DM.11G016820/Soltu.DM.02G033290/Soltu.DM.12G023230	4

			6	94	94	6		
2239	GO:0000075	cell cycle checkpoint signaling	55/12756	0.898683361	0.999994594	0.94442346	Soltu.DM.10G020590/Soltu.DM.11G016820/Soltu.DM.02G033290	3
2240	GO:0048444	floral organ morphogenesis	55/12756	0.898683361	0.999994594	0.94442346	Soltu.DM.10G023790/Soltu.DM.09G022610/Soltu.DM.05G003100	3
2241	GO:0009166	nucleotide catabolic process	40/12756	0.89914045	0.999994594	0.94442346	Soltu.DM.12G004480/Soltu.DM.06G013720	2
2242	GO:0009309	amine biosynthetic process	40/12756	0.89914045	0.999994594	0.94442346	Soltu.DM.06G014480/Soltu.DM.01G050280	2
2243	GO:0042401	biogenic amine biosynthetic process	40/12756	0.89914045	0.999994594	0.94442346	Soltu.DM.06G014480/Soltu.DM.01G050280	2
2244	GO:0050684	regulation of mRNA processing	40/12756	0.89914045	0.999994594	0.94442346	Soltu.DM.12G025260/Soltu.DM.10G001400	2
2245	GO:1901926	cadinene metabolic process	70/12756	0.902926752	0.999994594	0.94442346	Soltu.DM.06G017120/Soltu.DM.06G017230/Soltu.DM.06G017100/Soltu.DM.07G004480	4
2246	GO:1901928	cadinene biosynthetic process	70/12756	0.902926752	0.999994594	0.94442346	Soltu.DM.06G017120/Soltu.DM.06G017230/Soltu.DM.06G017100/Soltu.DM.07G004480	4
2247	GO:0008654	phospholipid biosynthetic process	124/12756	0.903281783	0.999994594	0.94442346	Soltu.DM.08G014180/Soltu.DM.03G030800/Soltu.DM.02G010490/Soltu.DM.04G034770/Soltu.DM.01G017170/Soltu.DM.05G001470/Soltu.DM.08G026810/Soltu.DM.06G018040	8
2248	GO:1902275	regulation of chromatin organization	84/12756	0.903483222	0.999994594	0.94442346	Soltu.DM.08G022190/Soltu.DM.12G025260/Soltu.DM.04G038280/Soltu.DM.10G024770/Soltu.DM.03G020440	5
2249	GO:0022604	regulation of cell morphogenesis	111/12756	0.903932545	0.999994594	0.94442346	Soltu.DM.12G020370/Soltu.DM.12G020350/Soltu.DM.01G028770/Soltu.DM.02G030780/Soltu.DM.06G023200/Soltu.DM.12G020340/Soltu.DM.09G027230	7
2250	GO:1903311	regulation of mRNA metabolic process	111/12756	0.903932545	0.999994594	0.94442346	Soltu.DM.05G004270/Soltu.DM.12G025260/Soltu.DM.08G013620/Soltu.DM.05G026810/Soltu.DM.11G016820/Soltu.DM.10G001400/Soltu.DM.04G031030	7
2251	GO:0006119	oxidative phosphorylation	24/12756	0.905272543	0.999994594	0.94442346	Soltu.DM.02G018700	1
2252	GO:0006892	post-Golgi vesicle-mediated transport	24/12756	0.905272543	0.999994594	0.94442346	Soltu.DM.01G042120	1
2253	GO:0007040	lysosome organization	24/12756	0.905272543	0.999994594	0.94442346	Soltu.DM.06G005370	1
2254	GO:0051784	negative regulation of nuclear division	24/12756	0.905272543	0.999994594	0.94442346	Soltu.DM.10G020590	1
2255	GO:1902882	regulation of response to oxidative stress	24/12756	0.905272543	0.999994594	0.94442346	Soltu.DM.07G015200	1
2256	GO:1901988	negative regulation of cell cycle phase transition	56/12	0.9053	0.9999	0.9444	Soltu.DM.10G020590/Soltu.DM.11G016820/Soltu.DM.02G033290	3

			756	6456	94594	2346		
2257	GO:0006626	protein targeting to mitochondrion	41/12756	0.906748198	0.999994594	0.94442346	Soltu.DM.04G037150/Soltu.DM.12G021450	2
2258	GO:0019359	nicotinamide nucleotide biosynthetic process	41/12756	0.906748198	0.999994594	0.94442346	Soltu.DM.12G004480/Soltu.DM.06G013720	2
2259	GO:0032388	positive regulation of intracellular transport	41/12756	0.906748198	0.999994594	0.94442346	Soltu.DM.07G002400/Soltu.DM.08G001690	2
2260	GO:0042180	cellular ketone metabolic process	41/12756	0.906748198	0.999994594	0.94442346	Soltu.DM.06G032850/Soltu.DM.06G032860	2
2261	GO:0070585	protein localization to mitochondrion	41/12756	0.906748198	0.999994594	0.94442346	Soltu.DM.04G037150/Soltu.DM.12G021450	2
2262	GO:0072655	establishment of protein localization to mitochondrion	41/12756	0.906748198	0.999994594	0.94442346	Soltu.DM.04G037150/Soltu.DM.12G021450	2
2263	GO:1901000	regulation of response to salt stress	41/12756	0.906748198	0.999994594	0.94442346	Soltu.DM.06G017300/Soltu.DM.02G026820	2
2264	GO:0031123	RNA 3'-end processing	71/12756	0.908751792	0.999994594	0.94442346	Soltu.DM.01G006380/Soltu.DM.12G005490/Soltu.DM.03G003370/Soltu.DM.03G019650	4
2265	GO:0035194	RNA-mediated post-transcriptional gene silencing	99/12756	0.91055382	0.999994594	0.94442346	Soltu.DM.12G005490/Soltu.DM.12G026070/Soltu.DM.11G025410/Soltu.DM.01G010020/Soltu.DM.04G031030/Soltu.DM.11G004150	6
2266	GO:0051301	cell division	190/12756	0.911529513	0.999994594	0.94442346	Soltu.DM.04G027320/Soltu.DM.12G007610/Soltu.DM.07G000930/Soltu.DM.02G015410/Soltu.DM.01G028770/Soltu.DM.04G022240/Soltu.DM.01G047760/Soltu.DM.12G010960/Soltu.DM.03G014570/Soltu.DM.10G024000/Soltu.DM.12G023230/Soltu.DM.03G014580/Soltu.DM.07G022640	13
2267	GO:0031056	regulation of histone modification	57/12756	0.91164453 7	0.999994594	0.94442346	Soltu.DM.08G022190/Soltu.DM.12G025260/Soltu.DM.04G038280	3
2268	GO:0034248	regulation of amide metabolic process	139/12756	0.911852555	0.999994594	0.94442346	Soltu.DM.05G004270/Soltu.DM.10G027270/Soltu.DM.08G029860/Soltu.DM.12G015690/Soltu.DM.04G001110/Soltu.DM.08G013620/Soltu.DM.05G006430/Soltu.DM.05G026810/Soltu.DM.04G031030	9
2269	GO:0048528	post-embryonic root development	126/12756	0.912137831	0.999994594	0.94442346	Soltu.DM.02G006700/Soltu.DM.12G004060/Soltu.DM.08G023320/Soltu.DM.11G018040/Soltu.DM.06G020260/Soltu.DM.12G029710/Soltu.DM.02G022410/Soltu.DM.06G034230	8
2270	GO:0019363	pyridine nucleotide biosynthetic process	42/12756	0.913816329	0.999994594	0.94442346	Soltu.DM.12G004480/Soltu.DM.06G013720	2
2271	GO:0048449	floral organ formation	42/12756	0.913816329	0.999994594	0.94442346	Soltu.DM.10G023790/Soltu.DM.05G003100	2
2272	GO:1901292	nucleoside phosphate catabolic process	42/12756	0.913816329	0.999994594	0.94442346	Soltu.DM.12G004480/Soltu.DM.06G013720	2
2273	GO:0009126	purine nucleoside monophosphate metabolic process	86/12756	0.913936	0.999994594	0.94442346	Soltu.DM.12G004480/Soltu.DM.02G018700/Soltu.DM.07G009580/Soltu.DM.06G013720/Soltu.DM.11G025570	5
22	GO:00	purine ribonucleoside monophosphate	86/	0.9	0.9	0.9	Soltu.DM.12G004480/Soltu.DM.02G018700/Soltu.DM.07G009580/Soltu.DM.06G013720/S	5

74	09167	metabolic process	12756	13936	99994594	4442346	oltu.DM.11G025570	
2275	GO:0007080	mitotic metaphase plate congression	25/12756	0.914141152	0.999994594	0.94442346	Soltu.DM.01G043730	1
2276	GO:0007163	establishment or maintenance of cell polarity	25/12756	0.914141152	0.999994594	0.94442346	Soltu.DM.02G016680	1
2277	GO:0008608	attachment of spindle microtubules to kinetochore	25/12756	0.914141152	0.999994594	0.94442346	Soltu.DM.01G043730	1
2278	GO:0010582	floral meristem determinacy	25/12756	0.914141152	0.999994594	0.94442346	Soltu.DM.06G018130	1
2279	GO:0022898	regulation of transmembrane transporter activity	25/12756	0.914141152	0.999994594	0.94442346	Soltu.DM.11G004950	1
2280	GO:0032409	regulation of transporter activity	25/12756	0.914141152	0.999994594	0.94442346	Soltu.DM.11G004950	1
2281	GO:0032412	regulation of monoatomic ion transmembrane transporter activity	25/12756	0.914141152	0.999994594	0.94442346	Soltu.DM.11G004950	1
2282	GO:0033047	regulation of mitotic sister chromatid segregation	25/12756	0.914141152	0.999994594	0.94442346	Soltu.DM.10G020590	1
2283	GO:0046688	response to copper ion	25/12756	0.914141152	0.999994594	0.94442346	Soltu.DM.05G022440	1
2284	GO:0051098	regulation of binding	25/12756	0.914141152	0.999994594	0.94442346	Soltu.DM.01G039760	1
2285	GO:0080171	lytic vacuole organization	25/12756	0.914141152	0.999994594	0.94442346	Soltu.DM.06G005370	1
2286	GO:0003008	system process	72/12756	0.914264214	0.999994594	0.94442346	Soltu.DM.03G027330/Soltu.DM.02G017970/Soltu.DM.10G004300/Soltu.DM.03G027340	4
2287	GO:0007059	chromosome segregation	127/12756	0.916300142	0.999994594	0.94442346	Soltu.DM.10G020590/Soltu.DM.06G019850/Soltu.DM.09G014080/Soltu.DM.02G001620/Soltu.DM.02G001630/Soltu.DM.12G016140/Soltu.DM.09G025170/Soltu.DM.01G043730	8
2288	GO:0009100	glycoprotein metabolic process	179/12756	0.917304725	0.999994594	0.94442346	Soltu.DM.04G011110/Soltu.DM.02G025970/Soltu.DM.02G024810/Soltu.DM.03G037510/Soltu.DM.10G027470/Soltu.DM.04G011320/Soltu.DM.04G011240/Soltu.DM.10G028070/Soltu.DM.04G000320/Soltu.DM.04G011370/Soltu.DM.05G001020/Soltu.DM.11G021090	12
2289	GO:0002757	immune response-activating signaling pathway	58/12756	0.917543505	0.999994594	0.94442346	Soltu.DM.04G018070/Soltu.DM.08G011890/Soltu.DM.01G051770	3
2290	GO:0002764	immune response-regulating signaling pathway	58/12756	0.917543505	0.999994594	0.94442346	Soltu.DM.04G018070/Soltu.DM.08G011890/Soltu.DM.01G051770	3
2291	GO:1903829	positive regulation of protein localization	58/12756	0.917543505	0.999994594	0.94442346	Soltu.DM.07G002400/Soltu.DM.01G002690/Soltu.DM.08G001690	3

2292	GO:0000122	negative regulation of transcription by RNA polymerase II	43/12756	0.920379157	0.999994594	0.94442346	Soltu.DM.12G025260/Soltu.DM.04G038280	2
2293	GO:0048278	vesicle docking	43/12756	0.920379157	0.999994594	0.94442346	Soltu.DM.06G005370/Soltu.DM.11G026460	2
2294	GO:0000038	very long-chain fatty acid metabolic process	26/12756	0.922180092	0.999994594	0.94442346	Soltu.DM.10G026590	1
2295	GO:0006213	pyrimidine nucleoside metabolic process	26/12756	0.922180092	0.999994594	0.94442346	Soltu.DM.01G017170	1
2296	GO:0007129	homologous chromosome pairing at meiosis	26/12756	0.922180092	0.999994594	0.94442346	Soltu.DM.09G025170	1
2297	GO:0031062	positive regulation of histone methylation	26/12756	0.922180092	0.999994594	0.94442346	Soltu.DM.12G025260	1
2298	GO:0031146	SCF-dependent proteasomal ubiquitin-dependent protein catabolic process	26/12756	0.922180092	0.999994594	0.94442346	Soltu.DM.06G033930	1
2299	GO:0090503	RNA phosphodiester bond hydrolysis, exonucleolytic	26/12756	0.922180092	0.999994594	0.94442346	Soltu.DM.09G002100	1
2300	GO:0030422	siRNA processing	59/12756	0.923081062	0.999994594	0.94442346	Soltu.DM.01G010020/Soltu.DM.04G031030/Soltu.DM.11G004150	3
2301	GO:0051783	regulation of nuclear division	59/12756	0.923081062	0.999994594	0.94442346	Soltu.DM.03G003730/Soltu.DM.10G020590/Soltu.DM.12G023230	3
2302	GO:0097435	supramolecular fiber organization	168/12756	0.923416539	0.999994594	0.94442346	Soltu.DM.07G026780/Soltu.DM.04G027320/Soltu.DM.12G007610/Soltu.DM.02G028740/Soltu.DM.01G028770/Soltu.DM.01G008180/Soltu.DM.09G015150/Soltu.DM.04G022240/Soltu.DM.05G009320/Soltu.DM.11G018040/Soltu.DM.09G027230	11
2303	GO:0006875	cellular metal ion homeostasis	102/12756	0.924000677	0.999994594	0.94442346	Soltu.DM.07G002440/Soltu.DM.03G017590/Soltu.DM.05G021830/Soltu.DM.04G003430/Soltu.DM.07G009580/Soltu.DM.10G004300	6
2304	GO:0007015	actin filament organization	74/12756	0.924405074	0.999994594	0.94442346	Soltu.DM.07G026780/Soltu.DM.01G028770/Soltu.DM.09G015150/Soltu.DM.09G027230	4
2305	GO:0065002	intracellular protein transmembrane transport	74/12756	0.924405074	0.999994594	0.94442346	Soltu.DM.04G037150/Soltu.DM.08G023320/Soltu.DM.02G032340/Soltu.DM.12G021450	4
2306	GO:0006305	DNA alkylation	44/12756	0.926469303	0.999994594	0.94442346	Soltu.DM.09G024860/Soltu.DM.10G024770	2
2307	GO:0006306	DNA methylation	44/12756	0.926469303	0.999994594	0.94442346	Soltu.DM.09G024860/Soltu.DM.10G024770	2
2308	GO:0022406	membrane docking	44/12756	0.926469303	0.999994594	0.94442346	Soltu.DM.06G005370/Soltu.DM.11G026460	2
2309	GO:0071472	cellular response to salt stress	44/1275	0.9264693	0.9999945	0.9444234	Soltu.DM.03G022850/Soltu.DM.01G008180	2

			6	03	94	6		
2310	GO:0140056	organelle localization by membrane tethering	44/12756	0.926469303	0.999994594	0.94442346	Soltu.DM.06G005370/Soltu.DM.11G026460	2
2311	GO:0007005	mitochondrion organization	143/12756	0.926916743	0.999994594	0.94442346	Soltu.DM.04G037150/Soltu.DM.02G006310/Soltu.DM.04G034330/Soltu.DM.01G039130/Soltu.DM.08G023320/Soltu.DM.11G022310/Soltu.DM.01G042120/Soltu.DM.06G025410/Soltu.DM.12G021450	9
2312	GO:0006913	nucleocytoplasmic transport	130/12756	0.927786639	0.999994594	0.94442346	Soltu.DM.07G020410/Soltu.DM.12G005490/Soltu.DM.03G003730/Soltu.DM.12G024350/Soltu.DM.09G005370/Soltu.DM.11G024760/Soltu.DM.04G011330/Soltu.DM.07G006510	8
2313	GO:0051169	nuclear transport	130/12756	0.927786639	0.999994594	0.94442346	Soltu.DM.07G020410/Soltu.DM.12G005490/Soltu.DM.03G003730/Soltu.DM.12G024350/Soltu.DM.09G005370/Soltu.DM.11G024760/Soltu.DM.04G011330/Soltu.DM.07G006510	8
2314	GO:0015979	photosynthesis	117/12756	0.929383031	0.999994594	0.94442346	Soltu.DM.03G037170/Soltu.DM.10G018880/Soltu.DM.03G022850/Soltu.DM.01G024860/Soltu.DM.03G000900/Soltu.DM.04G037460/Soltu.DM.07G024910	7
2315	GO:0010022	meristem determinacy	27/12756	0.929466921	0.999994594	0.94442346	Soltu.DM.06G018130	1
2316	GO:0031167	rRNA methylation	27/12756	0.929466921	0.999994594	0.94442346	Soltu.DM.01G032530	1
2317	GO:0043244	regulation of protein-containing complex disassembly	27/12756	0.929466921	0.999994594	0.94442346	Soltu.DM.09G015150	1
2318	GO:0048658	anther wall tapetum development	27/12756	0.929466921	0.999994594	0.94442346	Soltu.DM.10G000600	1
2319	GO:0051090	regulation of DNA-binding transcription factor activity	27/12756	0.929466921	0.999994594	0.94442346	Soltu.DM.02G018520	1
2320	GO:0051310	metaphase plate congression	27/12756	0.929466921	0.999994594	0.94442346	Soltu.DM.01G043730	1
2321	GO:1900865	chloroplast RNA modification	27/12756	0.929466921	0.999994594	0.94442346	Soltu.DM.03G033060	1
2322	GO:0090696	post-embryonic plant organ development	183/12756	0.930177114	0.999994594	0.94442346	Soltu.DM.10G023790/Soltu.DM.02G006700/Soltu.DM.12G004060/Soltu.DM.09G022610/Soltu.DM.05G003100/Soltu.DM.08G023320/Soltu.DM.02G024210/Soltu.DM.11G018040/Soltu.DM.06G020260/Soltu.DM.12G029710/Soltu.DM.02G022410/Soltu.DM.06G034230	12
2323	GO:0009909	regulation of flower development	196/12756	0.930743036	0.999994594	0.94442346	Soltu.DM.03G022540/Soltu.DM.06G019760/Soltu.DM.05G012760/Soltu.DM.05G011970/Soltu.DM.02G004510/Soltu.DM.04G006870/Soltu.DM.06G021830/Soltu.DM.07G028470/Soltu.DM.02G002210/Soltu.DM.05G012040/Soltu.DM.08G001470/Soltu.DM.03G024000/Soltu.DM.03G020440	13
2324	GO:0009132	nucleoside diphosphate metabolic process	45/12756	0.932117709	0.999994594	0.94442346	Soltu.DM.12G004480/Soltu.DM.06G013720	2
2325	GO:0072525	pyridine-containing compound biosynthetic process	45/12756	0.932117709	0.999994594	0.94442346	Soltu.DM.12G004480/Soltu.DM.06G013720	2
2326	GO:0051130	positive regulation of cellular component organization	171/12756	0.932911946	0.999994594	0.94442346	Soltu.DM.12G020370/Soltu.DM.12G020350/Soltu.DM.12G025260/Soltu.DM.03G003730/Soltu.DM.09G015150/Soltu.DM.01G002690/Soltu.DM.06G018040/Soltu.DM.12G020340/Soltu.DM.09G027230/Soltu.DM.02G013390/Soltu.DM.12G023230	11
2327	GO:0006606	protein import into nucleus	61/12	0.9331	0.9999	0.9444	Soltu.DM.03G003730/Soltu.DM.04G011330/Soltu.DM.07G006510	3

			756	47103	94594	2346		
2328	GO:0018022	peptidyl-lysine methylation	61/12756	0.933147103	0.999994594	0.94442346	Soltu.DM.09G019870/Soltu.DM.05G000070/Soltu.DM.01G024940	3
2329	GO:0033044	regulation of chromosome organization	132/12756	0.93466191	0.999994594	0.94442346	Soltu.DM.08G022190/Soltu.DM.12G025260/Soltu.DM.10G020590/Soltu.DM.04G038280/Soltu.DM.05G006310/Soltu.DM.01G002690/Soltu.DM.10G024770/Soltu.DM.02G013390	8
2330	GO:0006401	RNA catabolic process	105/12756	0.935639684	0.999994594	0.94442346	Soltu.DM.09G014740/Soltu.DM.08G013620/Soltu.DM.05G026810/Soltu.DM.10G012880/Soltu.DM.11G004920/Soltu.DM.09G002100	6
2331	GO:0007399	nervous system development	105/12756	0.935639684	0.999994594	0.94442346	Soltu.DM.12G020370/Soltu.DM.01G042210/Soltu.DM.12G020350/Soltu.DM.12G025260/Soltu.DM.09G019870/Soltu.DM.12G020340	6
2332	GO:0071236	cellular response to antibiotic	91/12756	0.935825777	0.999994594	0.94442346	Soltu.DM.09G018910/Soltu.DM.09G029050/Soltu.DM.02G006310/Soltu.DM.04G028540/Soltu.DM.07G024240	5
2333	GO:0007135	meiosis II	28/12756	0.936071951	0.999994594	0.94442346	Soltu.DM.12G023840	1
2334	GO:0007417	central nervous system development	28/12756	0.936071951	0.999994594	0.94442346	Soltu.DM.01G042210	1
2335	GO:0009792	embryo development ending in birth or egg hatching	28/12756	0.936071951	0.999994594	0.94442346	Soltu.DM.02G029740	1
2336	GO:0043647	inositol phosphate metabolic process	28/12756	0.936071951	0.999994594	0.94442346	Soltu.DM.03G008510	1
2337	GO:0051303	establishment of chromosome localization	28/12756	0.936071951	0.999994594	0.94442346	Soltu.DM.01G043730	1
2338	GO:0061983	meiosis II cell cycle process	28/12756	0.936071951	0.999994594	0.94442346	Soltu.DM.12G023840	1
2339	GO:0000910	cytokinesis	119/12756	0.93644617	0.999994594	0.94442346	Soltu.DM.04G027320/Soltu.DM.12G007610/Soltu.DM.07G000930/Soltu.DM.02G015410/Soltu.DM.01G028770/Soltu.DM.04G022240/Soltu.DM.12G023230	7
2340	GO:0031401	positive regulation of protein modification process	146/12756	0.936708261	0.999994594	0.94442346	Soltu.DM.07G017210/Soltu.DM.07G017190/Soltu.DM.07G017200/Soltu.DM.02G018520/Soltu.DM.07G017180/Soltu.DM.02G028740/Soltu.DM.12G025260/Soltu.DM.06G015770/Soltu.DM.09G005140	9
2341	GO:0007051	spindle organization	46/12756	0.937353668	0.999994594	0.94442346	Soltu.DM.03G003730/Soltu.DM.03G035510	2
2342	GO:0030182	neuron differentiation	46/12756	0.937353668	0.999994594	0.94442346	Soltu.DM.01G042210/Soltu.DM.09G019870	2
2343	GO:0046939	nucleotide phosphorylation	46/12756	0.937353668	0.999994594	0.94442346	Soltu.DM.12G004480/Soltu.DM.06G013720	2
2344	GO:1990542	mitochondrial transmembrane transport	46/12756	0.937353668	0.999994594	0.94442346	Soltu.DM.04G037150/Soltu.DM.12G021450	2
23	GO:00	heterochromatin formation	77/	0.9	0.9	0.9	Soltu.DM.04G033160/Soltu.DM.10G024770/Soltu.DM.01G024940/Soltu.DM.03G020440	4

45	31507		12756	37600342	99994594	4442346		
2346	GO:0051050	positive regulation of transport	77/12756	0.937600342	0.999994594	0.94442346	Soltu.DM.07G002400/Soltu.DM.01G002690/Soltu.DM.08G001690/Soltu.DM.02G022620	4
2347	GO:0006417	regulation of translation	133/12756	0.937879946	0.999994594	0.94442346	Soltu.DM.05G004270/Soltu.DM.10G027270/Soltu.DM.12G015690/Soltu.DM.04G001110/Soltu.DM.08G013620/Soltu.DM.05G006430/Soltu.DM.05G026810/Soltu.DM.04G031030	8
2348	GO:0051260	protein homooligomerization	92/12756	0.939552774	0.999994594	0.94442346	Soltu.DM.03G012810/Soltu.DM.08G014620/Soltu.DM.08G030020/Soltu.DM.02G001620/Soltu.DM.02G001630	5
2349	GO:0006897	endocytosis	78/12756	0.941510484	0.999994594	0.94442346	Soltu.DM.09G000440/Soltu.DM.10G023680/Soltu.DM.01G042120/Soltu.DM.08G001690	4
2350	GO:0070828	heterochromatin organization	78/12756	0.941510484	0.999994594	0.94442346	Soltu.DM.04G033160/Soltu.DM.10G024770/Soltu.DM.01G024940/Soltu.DM.03G020440	4
2351	GO:0034655	nucleobase-containing compound catabolic process	161/12756	0.941777581	0.999994594	0.94442346	Soltu.DM.12G004480/Soltu.DM.09G014740/Soltu.DM.08G013620/Soltu.DM.05G003030/Soltu.DM.06G013720/Soltu.DM.12G003790/Soltu.DM.05G026810/Soltu.DM.10G012880/Soltu.DM.11G004920/Soltu.DM.09G002100	10
2352	GO:0000963	mitochondrial RNA processing	29/12756	0.942058927	0.999994594	0.94442346	Soltu.DM.06G002320	1
2353	GO:0006334	nucleosome assembly	29/12756	0.942058927	0.999994594	0.94442346	Soltu.DM.02G029740	1
2354	GO:0010389	regulation of G2/M transition of mitotic cell cycle	29/12756	0.942058927	0.999994594	0.94442346	Soltu.DM.02G033290	1
2355	GO:0030100	regulation of endocytosis	29/12756	0.942058927	0.999994594	0.94442346	Soltu.DM.10G004310	1
2356	GO:0033108	mitochondrial respiratory chain complex assembly	29/12756	0.942058927	0.999994594	0.94442346	Soltu.DM.02G006310	1
2357	GO:0034097	response to cytokine	29/12756	0.942058927	0.999994594	0.94442346	Soltu.DM.08G013620	1
2358	GO:0030029	actin filament-based process	107/12756	0.942492855	0.999994594	0.94442346	Soltu.DM.07G026780/Soltu.DM.09G018720/Soltu.DM.01G028770/Soltu.DM.09G015150/Soltu.DM.10G001460/Soltu.DM.09G027230	6
2359	GO:0046903	secretion	107/12756	0.942492855	0.999994594	0.94442346	Soltu.DM.03G017520/Soltu.DM.06G005370/Soltu.DM.10G022360/Soltu.DM.01G000060/Soltu.DM.11G026460/Soltu.DM.02G019940	6
2360	GO:0009161	ribonucleoside monophosphate metabolic process	93/12756	0.943083968	0.999994594	0.94442346	Soltu.DM.12G004480/Soltu.DM.02G018700/Soltu.DM.07G009580/Soltu.DM.06G013720/Soltu.DM.11G025570	5
2361	GO:0007018	microtubule-based movement	79/12756	0.945195974	0.999994594	0.94442346	Soltu.DM.12G012260/Soltu.DM.01G050230/Soltu.DM.07G024370/Soltu.DM.03G035510	4
2362	GO:0006303	double-strand break repair via nonhomologous end joining	30/12756	0.947485639	0.999994594	0.94442346	Soltu.DM.05G007660	1

2363	GO:0006874	cellular calcium ion homeostasis	30/12756	0.947485639	0.999994594	0.94442346	Soltu.DM.10G004300	1
2364	GO:0007032	endosome organization	30/12756	0.947485639	0.999994594	0.94442346	Soltu.DM.06G005370	1
2365	GO:0022904	respiratory electron transport chain	30/12756	0.947485639	0.999994594	0.94442346	Soltu.DM.02G018700	1
2366	GO:0031175	neuron projection development	30/12756	0.947485639	0.999994594	0.94442346	Soltu.DM.09G019870	1
2367	GO:0032465	regulation of cytokinesis	30/12756	0.947485639	0.999994594	0.94442346	Soltu.DM.12G023230	1
2368	GO:0033045	regulation of sister chromatid segregation	30/12756	0.947485639	0.999994594	0.94442346	Soltu.DM.10G020590	1
2369	GO:0048235	pollen sperm cell differentiation	30/12756	0.947485639	0.999994594	0.94442346	Soltu.DM.07G022050	1
2370	GO:0051568	histone H3-K4 methylation	30/12756	0.947485639	0.999994594	0.94442346	Soltu.DM.09G019870	1
2371	GO:0055074	calcium ion homeostasis	30/12756	0.947485639	0.999994594	0.94442346	Soltu.DM.10G004300	1
2372	GO:0048868	pollen tube development	150/12756	0.947974032	0.999994594	0.94442346	Soltu.DM.10G024410/Soltu.DM.02G031090/Soltu.DM.09G005320/Soltu.DM.09G026810/Soltu.DM.12G024030/Soltu.DM.02G012570/Soltu.DM.04G022240/Soltu.DM.10G002320/Soltu.DM.06G023200	9
2373	GO:0006338	chromatin remodeling	164/12756	0.949506977	0.999994594	0.94442346	Soltu.DM.08G022190/Soltu.DM.04G033160/Soltu.DM.06G017030/Soltu.DM.11G010650/Soltu.DM.02G001620/Soltu.DM.02G001630/Soltu.DM.02G029740/Soltu.DM.10G024770/Soltu.DM.01G024940/Soltu.DM.03G020440	10
2374	GO:0051259	protein complex oligomerization	164/12756	0.949506977	0.999994594	0.94442346	Soltu.DM.03G012810/Soltu.DM.08G014620/Soltu.DM.07G013360/Soltu.DM.02G020550/Soltu.DM.08G030020/Soltu.DM.03G000340/Soltu.DM.02G001620/Soltu.DM.02G001630/Soltu.DM.10G002820/Soltu.DM.02G032340	10
2375	GO:0016106	sesquiterpenoid biosynthetic process	65/12756	0.949731057	0.999994594	0.94442346	Soltu.DM.11G024450/Soltu.DM.11G004760/Soltu.DM.06G029640	3
2376	GO:0047484	regulation of response to osmotic stress	49/12756	0.950855885	0.999994594	0.94442346	Soltu.DM.06G017300/Soltu.DM.02G026820	2
2377	GO:0070192	chromosome organization involved in meiotic cell cycle	49/12756	0.950855885	0.999994594	0.94442346	Soltu.DM.09G025170/Soltu.DM.05G023970	2
2378	GO:0090697	post-embryonic plant organ morphogenesis	124/12756	0.951446876	0.999994594	0.94442346	Soltu.DM.10G023790/Soltu.DM.02G006700/Soltu.DM.09G022610/Soltu.DM.05G003100/Soltu.DM.11G018040/Soltu.DM.06G020260/Soltu.DM.02G022410	7
2379	GO:0000281	mitotic cytokinesis	110/12756	0.951552836	0.999994594	0.94442346	Soltu.DM.04G027320/Soltu.DM.12G007610/Soltu.DM.07G000930/Soltu.DM.02G015410/Soltu.DM.01G028770/Soltu.DM.04G022240	6
2380	GO:0019827	stem cell population maintenance	81/1275	0.9519370	0.9999945	0.9444234	Soltu.DM.02G027330/Soltu.DM.03G027640/Soltu.DM.11G017980/Soltu.DM.01G010020	4

			6	89	94	6		
2381	GO:0098727	maintenance of cell number	81/12756	0.951937089	0.999994594	0.944423446	Soltu.DM.02G027330/Soltu.DM.03G027640/Soltu.DM.11G017980/Soltu.DM.01G010020	4
2382	GO:0006612	protein targeting to membrane	31/12756	0.952404476	0.999994594	0.944423446	Soltu.DM.02G032340	1
2383	GO:0006900	vesicle budding from membrane	31/12756	0.952404476	0.999994594	0.944423446	Soltu.DM.06G022490	1
2384	GO:0009838	abscission	31/12756	0.952404476	0.999994594	0.944423446	Soltu.DM.08G013580	1
2385	GO:0010191	mucilage metabolic process	31/12756	0.952404476	0.999994594	0.944423446	Soltu.DM.02G027540	1
2386	GO:0016126	sterol biosynthetic process	31/12756	0.952404476	0.999994594	0.944423446	Soltu.DM.02G007460	1
2387	GO:0044347	cell wall polysaccharide catabolic process	31/12756	0.952404476	0.999994594	0.944423446	Soltu.DM.11G000740	1
2388	GO:0006479	protein methylation	96/12756	0.952587707	0.999994594	0.944423446	Soltu.DM.09G019870/Soltu.DM.04G006870/Soltu.DM.05G000070/Soltu.DM.01G024940/Soltu.DM.06G017810	5
2389	GO:0008213	protein alkylation	96/12756	0.952587707	0.999994594	0.944423446	Soltu.DM.09G019870/Soltu.DM.04G006870/Soltu.DM.05G000070/Soltu.DM.01G024940/Soltu.DM.06G017810	5
2390	GO:0042981	regulation of apoptotic process	66/12756	0.953231521	0.999994594	0.944423446	Soltu.DM.02G018520/Soltu.DM.04G022240/Soltu.DM.06G024530	3
2391	GO:0034728	nucleosome organization	50/12756	0.954703453	0.999994594	0.944423446	Soltu.DM.11G010650/Soltu.DM.02G029740	2
2392	GO:0044728	DNA methylation or demethylation	50/12756	0.954703453	0.999994594	0.944423446	Soltu.DM.09G024860/Soltu.DM.10G024770	2
2393	GO:0018205	peptidyl-lysine modification	140/12756	0.956755447	0.999994594	0.944423446	Soltu.DM.02G020320/Soltu.DM.03G003730/Soltu.DM.06G019850/Soltu.DM.04G033160/Soltu.DM.09G019870/Soltu.DM.05G000070/Soltu.DM.02G020340/Soltu.DM.01G024940	8
2394	GO:0000291	nuclear-transcribed mRNA catabolic process, exonucleolytic	32/12756	0.956862933	0.999994594	0.944423446	Soltu.DM.05G026810	1
2395	GO:0043408	regulation of MAPK cascade	51/12756	0.958261839	0.999994594	0.944423446	Soltu.DM.11G010230/Soltu.DM.11G010220	2
2396	GO:0140053	mitochondrial gene expression	51/12756	0.958261839	0.999994594	0.944423446	Soltu.DM.12G028920/Soltu.DM.06G002320	2
2397	GO:0017038	protein import	127/12756	0.958845726	0.999994594	0.944423446	Soltu.DM.04G037150/Soltu.DM.03G003730/Soltu.DM.08G023320/Soltu.DM.04G011330/Soltu.DM.02G032340/Soltu.DM.07G006510/Soltu.DM.12G021450	7
2398	GO:0015980	energy derivation by oxidation of organic compounds	99/12	0.9606	0.9999	0.9444	Soltu.DM.01G040570/Soltu.DM.02G018700/Soltu.DM.03G037730/Soltu.DM.02G024820/Soltu.DM.11G008240	5

			756	25033	94594	2346		
2399	GO:0000245	spliceosomal complex assembly	33/12756	0.960904066	0.999994594	0.94442346	Soltu.DM.10G001400	1
2400	GO:0006915	apoptotic process	33/12756	0.960904066	0.999994594	0.94442346	Soltu.DM.12G025260	1
2401	GO:0007140	male meiotic nuclear division	33/12756	0.960904066	0.999994594	0.94442346	Soltu.DM.12G023840	1
2402	GO:0010091	trichome branching	33/12756	0.960904066	0.999994594	0.94442346	Soltu.DM.08G030100	1
2403	GO:0010183	pollen tube guidance	33/12756	0.960904066	0.999994594	0.94442346	Soltu.DM.12G024030	1
2404	GO:0050000	chromosome localization	33/12756	0.960904066	0.999994594	0.94442346	Soltu.DM.01G043730	1
2405	GO:0050918	positive chemotaxis	33/12756	0.960904066	0.999994594	0.94442346	Soltu.DM.12G024030	1
2406	GO:1902749	regulation of cell cycle G2/M phase transition	33/12756	0.960904066	0.999994594	0.94442346	Soltu.DM.02G033290	1
2407	GO:0009247	glycolipid biosynthetic process	52/12756	0.961551434	0.999994594	0.94442346	Soltu.DM.08G001900/Soltu.DM.02G010490	2
2408	GO:0046620	regulation of organ growth	52/12756	0.961551434	0.999994594	0.94442346	Soltu.DM.04G033430/Soltu.DM.11G001500	2
2409	GO:0061640	cytoskeleton-dependent cytokinesis	114/12756	0.961628016	0.999994594	0.94442346	Soltu.DM.04G027320/Soltu.DM.12G007610/Soltu.DM.07G000930/Soltu.DM.02G015410/Soltu.DM.01G028770/Soltu.DM.04G022240	6
2410	GO:0071806	protein transmembrane transport	85/12756	0.963186673	0.999994594	0.94442346	Soltu.DM.04G037150/Soltu.DM.08G023320/Soltu.DM.02G032340/Soltu.DM.12G021450	4
2411	GO:0008333	endosome to lysosome transport	34/12756	0.964566909	0.999994594	0.94442346	Soltu.DM.06G005370	1
2412	GO:0009958	positive gravitropism	34/12756	0.964566909	0.999994594	0.94442346	Soltu.DM.04G002690	1
2413	GO:0017004	cytochrome complex assembly	34/12756	0.964566909	0.999994594	0.94442346	Soltu.DM.02G006310	1
2414	GO:0031058	positive regulation of histone modification	34/12756	0.964566909	0.999994594	0.94442346	Soltu.DM.12G025260	1
2415	GO:0045324	late endosome to vacuole transport	34/12756	0.964566909	0.999994594	0.94442346	Soltu.DM.01G000060	1
24	GO:00	carbohydrate phosphorylation	34/	0.9	0.9	0.9	Soltu.DM.12G004480	1

16	46835		12756	64566909	99994594	4442346		
2417	GO:0060429	epithelium development	53/12756	0.964591349	0.999994594	0.94442346	Soltu.DM.10G022360/Soltu.DM.10G023760	2
2418	GO:0022900	electron transport chain	70/12756	0.965076843	0.999994594	0.94442346	Soltu.DM.02G018700/Soltu.DM.10G025910/Soltu.DM.02G030630	3
2419	GO:0034504	protein localization to nucleus	70/12756	0.965076843	0.999994594	0.94442346	Soltu.DM.03G003730/Soltu.DM.04G011330/Soltu.DM.07G006510	3
2420	GO:0046488	phosphatidylinositol metabolic process	70/12756	0.965076843	0.999994594	0.94442346	Soltu.DM.02G010490/Soltu.DM.04G034770/Soltu.DM.05G001470	3
2421	GO:0016571	histone methylation	86/12756	0.965589145	0.999994594	0.94442346	Soltu.DM.09G019870/Soltu.DM.04G006870/Soltu.DM.01G024940/Soltu.DM.06G017810	4
2422	GO:0000462	maturation of SSU-rRNA from tricistronic rRNA transcript (SSU-rRNA, 5.8S rRNA, LSU-rRNA)	54/12756	0.967399472	0.999994594	0.94442346	Soltu.DM.12G024350/Soltu.DM.01G051600	2
2423	GO:0006304	DNA modification	54/12756	0.967399472	0.999994594	0.94442346	Soltu.DM.09G024860/Soltu.DM.10G024770	2
2424	GO:0010501	RNA secondary structure unwinding	54/12756	0.967399472	0.999994594	0.94442346	Soltu.DM.08G021950/Soltu.DM.01G002690	2
2425	GO:0034968	histone lysine methylation	54/12756	0.967399472	0.999994594	0.94442346	Soltu.DM.09G019870/Soltu.DM.01G024940	2
2426	GO:0000165	MAPK cascade	35/12756	0.967886847	0.999994594	0.94442346	Soltu.DM.07G023200	1
2427	GO:0045036	protein targeting to chloroplast	35/12756	0.967886847	0.999994594	0.94442346	Soltu.DM.02G032340	1
2428	GO:0051983	regulation of chromosome segregation	35/12756	0.967886847	0.999994594	0.94442346	Soltu.DM.10G020590	1
2429	GO:0072596	establishment of protein localization to chloroplast	35/12756	0.967886847	0.999994594	0.94442346	Soltu.DM.02G032340	1
2430	GO:1905269	positive regulation of chromatin organization	35/12756	0.967886847	0.999994594	0.94442346	Soltu.DM.12G025260	1
2431	GO:0040011	locomotion	103/12756	0.969401045	0.999994594	0.94442346	Soltu.DM.08G027150/Soltu.DM.12G024030/Soltu.DM.01G028770/Soltu.DM.09G002090/Soltu.DM.10G004310	5
2432	GO:0000819	sister chromatid segregation	72/12756	0.969879057	0.999994594	0.94442346	Soltu.DM.06G019850/Soltu.DM.09G014080/Soltu.DM.01G043730	3
2433	GO:0120252	hydrocarbon metabolic process	161/12756	0.970354751	0.999994594	0.94442346	Soltu.DM.06G017120/Soltu.DM.11G004760/Soltu.DM.06G017230/Soltu.DM.06G017100/Soltu.DM.07G026340/Soltu.DM.11G009500/Soltu.DM.02G004480/Soltu.DM.10G004300/Soltu.DM.07G004480	9

2434	GO:0000288	nuclear-transcribed mRNA catabolic process, deadenylation-dependent decay	36/12756	0.970895956	0.999994594	0.94442346	Soltu.DM.05G026810	1
2435	GO:0007052	mitotic spindle organization	36/12756	0.970895956	0.999994594	0.94442346	Soltu.DM.03G035510	1
2436	GO:0045338	farnesyl diphosphate metabolic process	104/12756	0.97129259	0.999994594	0.94442346	Soltu.DM.06G017120/Soltu.DM.11G004760/Soltu.DM.06G017230/Soltu.DM.06G017100/Soltu.DM.07G004480	5
2437	GO:0010948	negative regulation of cell cycle process	73/12756	0.972039121	0.999994594	0.94442346	Soltu.DM.10G020590/Soltu.DM.11G016820/Soltu.DM.02G033290	3
2438	GO:0009626	plant-type hypersensitive response	134/12756	0.972307628	0.999994594	0.94442346	Soltu.DM.04G018070/Soltu.DM.02G006310/Soltu.DM.06G026400/Soltu.DM.01G051770/Soltu.DM.08G007450/Soltu.DM.09G023400/Soltu.DM.04G006280	7
2439	GO:0070646	protein modification by small protein removal	56/12756	0.972386162	0.999994594	0.94442346	Soltu.DM.06G018150/Soltu.DM.02G030830	2
2440	GO:0032940	secretion by cell	105/12756	0.973075235	0.999994594	0.94442346	Soltu.DM.03G017520/Soltu.DM.06G005370/Soltu.DM.10G022360/Soltu.DM.01G000060/Soltu.DM.11G026460	5
2441	GO:0120251	hydrocarbon biosynthetic process	149/12756	0.973416534	0.999994594	0.94442346	Soltu.DM.06G017120/Soltu.DM.11G004760/Soltu.DM.06G017230/Soltu.DM.06G017100/Soltu.DM.07G026340/Soltu.DM.11G009500/Soltu.DM.02G004480/Soltu.DM.07G004480	8
2442	GO:0031060	regulation of histone methylation	37/12756	0.973623316	0.999994594	0.94442346	Soltu.DM.12G025260	1
2443	GO:0072598	protein localization to chloroplast	37/12756	0.973623316	0.999994594	0.94442346	Soltu.DM.02G032340	1
2444	GO:0030036	actin cytoskeleton organization	90/12756	0.973811957	0.999994594	0.94442346	Soltu.DM.07G026780/Soltu.DM.01G028770/Soltu.DM.09G015150/Soltu.DM.09G027230	4
2445	GO:0032508	DNA duplex unwinding	90/12756	0.973811957	0.999994594	0.94442346	Soltu.DM.03G020310/Soltu.DM.07G017530/Soltu.DM.05G023970/Soltu.DM.05G018370	4
2446	GO:1901987	regulation of cell cycle phase transition	90/12756	0.973811957	0.999994594	0.94442346	Soltu.DM.10G020590/Soltu.DM.11G016820/Soltu.DM.02G033290/Soltu.DM.12G023230	4
2447	GO:0009932	cell tip growth	135/12756	0.973861096	0.999994594	0.94442346	Soltu.DM.10G024410/Soltu.DM.02G031090/Soltu.DM.09G005320/Soltu.DM.09G026810/Soltu.DM.12G024030/Soltu.DM.10G002320/Soltu.DM.06G023200	7
2448	GO:0034050	programmed cell death induced by symbiont	135/12756	0.973861096	0.999994594	0.94442346	Soltu.DM.04G018070/Soltu.DM.02G006310/Soltu.DM.06G026400/Soltu.DM.01G051770/Soltu.DM.08G007450/Soltu.DM.09G023400/Soltu.DM.04G006280	7
2449	GO:0140014	mitotic nuclear division	74/12756	0.974051828	0.999994594	0.94442346	Soltu.DM.06G019850/Soltu.DM.09G014080/Soltu.DM.01G043730	3
2450	GO:0006935	chemotaxis	57/12756	0.974594948	0.999994594	0.94442346	Soltu.DM.12G024030/Soltu.DM.01G028770	2
2451	GO:0065004	protein-DNA complex assembly	91/1275	0.9755584	0.999945	0.9444234	Soltu.DM.04G038280/Soltu.DM.02G029740/Soltu.DM.09G025170/Soltu.DM.12G029710	4

			6	48	94	6		
2452	GO:0001708	cell fate specification	38/12756	0.976095288	0.999994594	0.94442346	Soltu.DM.10G023760	1
2453	GO:0030334	regulation of cell migration	38/12756	0.976095288	0.999994594	0.94442346	Soltu.DM.02G018520	1
2454	GO:0031338	regulation of vesicle fusion	38/12756	0.976095288	0.999994594	0.94442346	Soltu.DM.06G005370	1
2455	GO:0045143	homologous chromosome segregation	38/12756	0.976095288	0.999994594	0.94442346	Soltu.DM.09G025170	1
2456	GO:1902850	microtubule cytoskeleton organization involved in mitosis	38/12756	0.976095288	0.999994594	0.94442346	Soltu.DM.03G035510	1
2457	GO:2000145	regulation of cell motility	38/12756	0.976095288	0.999994594	0.94442346	Soltu.DM.02G018520	1
2458	GO:0042330	taxis	58/12756	0.9766325	0.999994594	0.94442346	Soltu.DM.12G024030/Soltu.DM.01G028770	2
2459	GO:0006887	exocytosis	92/12756	0.977195069	0.999994594	0.94442346	Soltu.DM.03G017520/Soltu.DM.06G005370/Soltu.DM.10G022360/Soltu.DM.11G026460	4
2460	GO:0032392	DNA geometric change	92/12756	0.977195069	0.999994594	0.94442346	Soltu.DM.03G020310/Soltu.DM.07G017530/Soltu.DM.05G023970/Soltu.DM.05G018370	4
2461	GO:0000154	rRNA modification	39/12756	0.978335767	0.999994594	0.94442346	Soltu.DM.01G032530	1
2462	GO:0042023	DNA endoreduplication	39/12756	0.978335767	0.999994594	0.94442346	Soltu.DM.03G018740	1
2463	GO:0009910	negative regulation of flower development	59/12756	0.978511497	0.999994594	0.94442346	Soltu.DM.04G006870/Soltu.DM.05G012040	2
2464	GO:0010074	maintenance of meristem identity	59/12756	0.978511497	0.999994594	0.94442346	Soltu.DM.02G027330/Soltu.DM.01G010020	2
2465	GO:0051702	biological process involved in interaction with symbiont	139/12756	0.979307151	0.999994594	0.94442346	Soltu.DM.04G018070/Soltu.DM.02G006310/Soltu.DM.06G026400/Soltu.DM.01G051770/Soltu.DM.08G007450/Soltu.DM.09G023400/Soltu.DM.04G006280	7
2466	GO:0019684	photosynthesis, light reaction	94/12756	0.980163716	0.999994594	0.94442346	Soltu.DM.03G022850/Soltu.DM.03G000900/Soltu.DM.04G037460/Soltu.DM.07G024910	4
2467	GO:0051345	positive regulation of hydrolase activity	94/12756	0.980163716	0.999994594	0.94442346	Soltu.DM.05G006580/Soltu.DM.07G024370/Soltu.DM.02G026820/Soltu.DM.02G022620	4
2468	GO:0030433	ubiquitin-dependent ERAD pathway	40/12756	0.980366416	0.999994594	0.94442346	Soltu.DM.07G026100	1
2469	GO:0019953	sexual reproduction	183/1	0.9806	0.9999	0.9444	Soltu.DM.06G009270/Soltu.DM.04G000190/Soltu.DM.06G012350/Soltu.DM.07G022050/Soltu.DM.09G031520/Soltu.DM.05G022790/Soltu.DM.02G011180/Soltu.DM.05G004430/Sol	10

			2756	10884	94594	2346	tu.DM.09G031510/Soltu.DM.12G023840	
2470	GO:0044703	multi-organism reproductive process	197/12756	0.980702331	0.999994594	0.94442346	Soltu.DM.06G009270/Soltu.DM.04G000190/Soltu.DM.06G012350/Soltu.DM.07G022050/Soltu.DM.09G031520/Soltu.DM.12G024030/Soltu.DM.05G022790/Soltu.DM.02G011180/Soltu.DM.05G004430/Soltu.DM.09G031510/Soltu.DM.12G023840	11
2471	GO:0060627	regulation of vesicle-mediated transport	95/12756	0.981507474	0.999994594	0.94442346	Soltu.DM.06G005370/Soltu.DM.10G004310/Soltu.DM.02G022620/Soltu.DM.02G030830	4
2472	GO:0006888	endoplasmic reticulum to Golgi vesicle-mediated transport	61/12756	0.981840247	0.999994594	0.94442346	Soltu.DM.10G020590/Soltu.DM.08G024020	2
2473	GO:0045333	cellular respiration	61/12756	0.981840247	0.999994594	0.94442346	Soltu.DM.02G018700/Soltu.DM.03G037730	2
2474	GO:0051510	regulation of unidimensional cell growth	61/12756	0.981840247	0.999994594	0.94442346	Soltu.DM.02G030780/Soltu.DM.06G023200	2
2475	GO:0007131	reciprocal meiotic recombination	79/12756	0.982213641	0.999994594	0.94442346	Soltu.DM.12G023260/Soltu.DM.09G025170/Soltu.DM.05G023970	3
2476	GO:0140527	reciprocal homologous recombination	79/12756	0.982213641	0.999994594	0.94442346	Soltu.DM.12G023260/Soltu.DM.09G025170/Soltu.DM.05G023970	3
2477	GO:0031047	RNA-mediated gene silencing	142/12756	0.982680982	0.999994594	0.94442346	Soltu.DM.12G005490/Soltu.DM.09G024860/Soltu.DM.12G026070/Soltu.DM.11G025410/Soltu.DM.01G010020/Soltu.DM.04G031030/Soltu.DM.11G004150	7
2478	GO:0071824	protein-DNA complex subunit organization	112/12756	0.982947287	0.999994594	0.94442346	Soltu.DM.04G038280/Soltu.DM.11G010650/Soltu.DM.02G029740/Soltu.DM.09G025170/Soltu.DM.12G029710	5
2479	GO:0048598	embryonic morphogenesis	62/12756	0.983311205	0.999994594	0.94442346	Soltu.DM.05G006190/Soltu.DM.03G024040	2
2480	GO:0048513	animal organ development	80/12756	0.983520616	0.999994594	0.94442346	Soltu.DM.06G005370/Soltu.DM.10G022360/Soltu.DM.12G019270	3
2481	GO:0046246	terpene biosynthetic process	128/12756	0.983653879	0.999994594	0.94442346	Soltu.DM.06G017120/Soltu.DM.11G004760/Soltu.DM.06G017230/Soltu.DM.06G017100/Soltu.DM.07G026340/Soltu.DM.07G004480	6
2482	GO:0007041	lysosomal transport	42/12756	0.983874934	0.999994594	0.94442346	Soltu.DM.06G005370	1
2483	GO:0090150	establishment of protein localization to membrane	42/12756	0.983874934	0.999994594	0.94442346	Soltu.DM.02G032340	1
2484	GO:0045944	positive regulation of transcription by RNA polymerase II	158/12756	0.984005753	0.999994594	0.94442346	Soltu.DM.09G014750/Soltu.DM.08G019590/Soltu.DM.12G025260/Soltu.DM.04G038280/Soltu.DM.06G020510/Soltu.DM.12G026070/Soltu.DM.08G001050/Soltu.DM.01G002690	8
2485	GO:0031334	positive regulation of protein-containing complex assembly	63/12756	0.984666122	0.999994594	0.94442346	Soltu.DM.09G015150/Soltu.DM.09G027230	2
2486	GO:0043547	positive regulation of GTPase activity	63/12756	0.984666122	0.999994594	0.94442346	Soltu.DM.02G026820/Soltu.DM.02G022620	2
24	GO:00	ERAD pathway	81/	0.9	0.9	0.9	Soltu.DM.10G004220/Soltu.DM.06G009790/Soltu.DM.07G026100	3

87	36503		12756	84735368	99994594	4442346		
2488	GO:0043087	regulation of GTPase activity	81/12756	0.984735368	0.999994594	0.94442346	Soltu.DM.08G011890/Soltu.DM.02G026820/Soltu.DM.02G022620	3
2489	GO:0045786	negative regulation of cell cycle	81/12756	0.984735368	0.999994594	0.94442346	Soltu.DM.10G020590/Soltu.DM.11G016820/Soltu.DM.02G033290	3
2490	GO:0050877	nervous system process	43/12756	0.985386738	0.999994594	0.94442346	Soltu.DM.02G017970	1
2491	GO:0060284	regulation of cell development	115/12756	0.986035459	0.999994594	0.94442346	Soltu.DM.12G020370/Soltu.DM.12G020350/Soltu.DM.12G025260/Soltu.DM.06G023200/Soltu.DM.12G020340	5
2492	GO:0006418	tRNA aminoacylation for protein translation	44/12756	0.986756911	0.999994594	0.94442346	Soltu.DM.07G027760	1
2493	GO:0040012	regulation of locomotion	44/12756	0.986756911	0.999994594	0.94442346	Soltu.DM.02G018520	1
2494	GO:0080027	response to herbivore	44/12756	0.986756911	0.999994594	0.94442346	Soltu.DM.11G004760	1
2495	GO:0006402	mRNA catabolic process	83/12756	0.986912387	0.999994594	0.94442346	Soltu.DM.08G013620/Soltu.DM.05G026810/Soltu.DM.10G012880	3
2496	GO:0016556	mRNA modification	83/12756	0.986912387	0.999994594	0.94442346	Soltu.DM.03G033060/Soltu.DM.01G032530/Soltu.DM.06G002320	3
2497	GO:0035825	homologous recombination	83/12756	0.986912387	0.999994594	0.94442346	Soltu.DM.12G023260/Soltu.DM.09G025170/Soltu.DM.05G023970	3
2498	GO:0034976	response to endoplasmic reticulum stress	116/12756	0.986941819	0.999994594	0.94442346	Soltu.DM.08G019590/Soltu.DM.03G035420/Soltu.DM.10G004220/Soltu.DM.06G009790/Soltu.DM.07G026100	5
2499	GO:0080092	regulation of pollen tube growth	45/12756	0.987998711	0.999994594	0.94442346	Soltu.DM.06G023200	1
2500	GO:0030490	maturation of SSU-rRNA	66/12756	0.988119694	0.999994594	0.94442346	Soltu.DM.12G024350/Soltu.DM.01G051600	2
2501	GO:0009060	aerobic respiration	46/12756	0.989124156	0.999994594	0.94442346	Soltu.DM.02G018700	1
2502	GO:0043038	amino acid activation	46/12756	0.989124156	0.999994594	0.94442346	Soltu.DM.07G027760	1
2503	GO:0043039	tRNA aminoacylation	46/12756	0.989124156	0.999994594	0.94442346	Soltu.DM.07G027760	1
2504	GO:0051761	sesquiterpene metabolic process	119/12756	0.989338765	0.999994594	0.94442346	Soltu.DM.06G017120/Soltu.DM.11G004760/Soltu.DM.06G017230/Soltu.DM.06G017100/Soltu.DM.07G004480	5

2505	GO:0051762	sesquiterpene biosynthetic process	119/12756	0.989338765	0.999994594	0.94442346	Soltu.DM.06G017120/Soltu.DM.11G004760/Soltu.DM.06G017230/Soltu.DM.06G017100/Soltu.DM.07G004480	5
2506	GO:0042214	terpene metabolic process	135/12756	0.989537117	0.999994594	0.94442346	Soltu.DM.06G017120/Soltu.DM.11G004760/Soltu.DM.06G017230/Soltu.DM.06G017100/Soltu.DM.07G026340/Soltu.DM.07G004480	6
2507	GO:0061025	membrane fusion	68/12756	0.989987452	0.999994594	0.94442346	Soltu.DM.04G000190/Soltu.DM.03G017520	2
2508	GO:0010026	trichome differentiation	120/12756	0.99040445	0.999994594	0.94442346	Soltu.DM.08G030100/Soltu.DM.07G003440/Soltu.DM.12G010960/Soltu.DM.05G005120/Soltu.DM.03G020440	5
2509	GO:0006413	translational initiation	47/12756	0.990144141	0.999994594	0.94442346	Soltu.DM.08G019530	1
2510	GO:0006623	protein targeting to vacuole	47/12756	0.990144141	0.999994594	0.94442346	Soltu.DM.01G039100	1
2511	GO:1902533	positive regulation of intracellular signal transduction	47/12756	0.990144141	0.999994594	0.94442346	Soltu.DM.02G018520	1
2512	GO:0016050	vesicle organization	104/12756	0.990277462	0.999994594	0.94442346	Soltu.DM.03G017520/Soltu.DM.06G005370/Soltu.DM.06G022490/Soltu.DM.01G028770	4
2513	GO:0048609	multicellular organismal reproductive process	196/12756	0.990412798	0.999994594	0.94442346	Soltu.DM.07G013360/Soltu.DM.03G037120/Soltu.DM.07G022050/Soltu.DM.04G022240/Soltu.DM.08G011330/Soltu.DM.01G001590/Soltu.DM.08G005070/Soltu.DM.03G013100/Soltu.DM.07G024500/Soltu.DM.12G023840	10
2514	GO:0006366	transcription by RNA polymerase II	137/12756	0.990809221	0.999994594	0.94442346	Soltu.DM.11G008820/Soltu.DM.04G038280/Soltu.DM.06G020510/Soltu.DM.12G026070/Soltu.DM.08G001050/Soltu.DM.11G015370	6
2515	GO:0007034	vacuolar transport	105/12756	0.990958738	0.999994594	0.94442346	Soltu.DM.06G005370/Soltu.DM.01G000060/Soltu.DM.01G039100/Soltu.DM.01G042120	4
2516	GO:2000242	negative regulation of reproductive process	88/12756	0.991127534	0.999994594	0.94442346	Soltu.DM.04G006870/Soltu.DM.05G012040/Soltu.DM.01G024340	3
2517	GO:0071103	DNA conformation change	153/12756	0.991150776	0.999994594	0.94442346	Soltu.DM.03G020310/Soltu.DM.02G029740/Soltu.DM.12G016140/Soltu.DM.07G017530/Soltu.DM.05G023970/Soltu.DM.05G018370/Soltu.DM.03G020440	7
2518	GO:1903046	meiotic cell cycle process	183/12756	0.991215475	0.999994594	0.94442346	Soltu.DM.06G009270/Soltu.DM.09G031520/Soltu.DM.12G023260/Soltu.DM.05G022790/Soltu.DM.02G011180/Soltu.DM.09G025170/Soltu.DM.05G023970/Soltu.DM.09G031510/Soltu.DM.12G023840	9
2519	GO:0006906	vesicle fusion	49/12756	0.991906304	0.999994594	0.94442346	Soltu.DM.03G017520	1
2520	GO:0010256	endomembrane system organization	90/12756	0.992416713	0.999994594	0.94442346	Soltu.DM.09G018910/Soltu.DM.06G005370/Soltu.DM.06G021170	3
2521	GO:0090174	organelle membrane fusion	50/12756	0.992665545	0.999994594	0.94442346	Soltu.DM.03G017520	1
2522	GO:0022412	cellular process involved in reproduction in multicellular organism	108/127	0.9927391	0.9999945	0.9444234	Soltu.DM.04G000190/Soltu.DM.07G022050/Soltu.DM.05G005120/Soltu.DM.12G023840	4

			56	28	94	6		
2523	GO:0006457	protein folding	141/12756	0.992927573	0.999994594	0.94442346	Soltu.DM.04G037150/Soltu.DM.02G018520/Soltu.DM.11G019990/Soltu.DM.03G035420/Soltu.DM.04G018070/Soltu.DM.05G027000	6
2524	GO:0055046	microgametogenesis	51/12756	0.99335362	0.999994594	0.94442346	Soltu.DM.07G022050	1
2525	GO:0080156	mitochondrial mRNA modification	51/12756	0.99335362	0.999994594	0.94442346	Soltu.DM.06G002320	1
2526	GO:0090626	plant epidermis morphogenesis	110/12756	0.993733571	0.999994594	0.94442346	Soltu.DM.08G030100/Soltu.DM.05G021390/Soltu.DM.12G010960/Soltu.DM.08G012010	4
2527	GO:0006487	protein N-linked glycosylation	52/12756	0.993977192	0.999994594	0.94442346	Soltu.DM.02G024810	1
2528	GO:0010118	stomatal movement	74/12756	0.994030474	0.999994594	0.94442346	Soltu.DM.09G026500/Soltu.DM.02G013580	2
2529	GO:0008033	tRNA processing	190/12756	0.994167486	0.999994594	0.94442346	Soltu.DM.01G006380/Soltu.DM.06G028580/Soltu.DM.02G028320/Soltu.DM.01G046920/Soltu.DM.12G016740/Soltu.DM.12G030020/Soltu.DM.11G004920/Soltu.DM.06G029830/Soltu.DM.09G019340	9
2530	GO:0006302	double-strand break repair	144/12756	0.994202645	0.999994594	0.94442346	Soltu.DM.05G007660/Soltu.DM.02G001620/Soltu.DM.02G001630/Soltu.DM.09G025170/Soltu.DM.05G023970/Soltu.DM.03G002260	6
2531	GO:0051321	meiotic cell cycle	193/12756	0.995121149	0.999994594	0.94442346	Soltu.DM.06G009270/Soltu.DM.09G031520/Soltu.DM.12G023260/Soltu.DM.05G022790/Soltu.DM.02G011180/Soltu.DM.09G025170/Soltu.DM.05G023970/Soltu.DM.09G031510/Soltu.DM.12G023840	9
2532	GO:0045595	regulation of cell differentiation	178/12756	0.995165883	0.999994594	0.94442346	Soltu.DM.12G020370/Soltu.DM.12G020350/Soltu.DM.07G020410/Soltu.DM.08G022900/Soltu.DM.12G025260/Soltu.DM.06G023200/Soltu.DM.12G020340/Soltu.DM.10G024000	8
2533	GO:0072665	protein localization to vacuole	55/12756	0.995518542	0.999994594	0.94442346	Soltu.DM.01G039100	1
2534	GO:0072666	establishment of protein localization to vacuole	55/12756	0.995518542	0.999994594	0.94442346	Soltu.DM.01G039100	1
2535	GO:0045165	cell fate commitment	56/12756	0.995939131	0.999994594	0.94442346	Soltu.DM.10G023760	1
2536	GO:0007127	meiosis I	98/12756	0.995985112	0.999994594	0.94442346	Soltu.DM.12G023260/Soltu.DM.09G025170/Soltu.DM.05G023970	3
2537	GO:0006261	DNA-templated DNA replication	116/12756	0.995991717	0.999994594	0.94442346	Soltu.DM.11G009630/Soltu.DM.08G027160/Soltu.DM.12G029710/Soltu.DM.03G018740	4
2538	GO:0048193	Golgi vesicle transport	116/12756	0.995991717	0.999994594	0.94442346	Soltu.DM.10G020590/Soltu.DM.06G022490/Soltu.DM.08G024020/Soltu.DM.01G042120	4
2539	GO:0098813	nuclear chromosome segregation	116/12756	0.995991717	0.999994594	0.94442346	Soltu.DM.06G019850/Soltu.DM.09G014080/Soltu.DM.09G025170/Soltu.DM.01G043730	4
2540	GO:0006260	DNA replication	134/1	0.9962	0.9999	0.9444	Soltu.DM.11G009630/Soltu.DM.08G027160/Soltu.DM.02G029740/Soltu.DM.12G029710/Soltu.DM.03G018740	5

			2756	48913	94594	2346		
2541	GO:0001510	RNA methylation	57/12756	0.996320277	0.999994594	0.94442346	Soltu.DM.01G032530	1
2542	GO:1900864	mitochondrial RNA modification	57/12756	0.996320277	0.999994594	0.94442346	Soltu.DM.06G002320	1
2543	GO:0042255	ribosome assembly	80/12756	0.996460267	0.999994594	0.94442346	Soltu.DM.01G051600/Soltu.DM.03G021590	2
2544	GO:0009856	pollination	199/12756	0.996603845	0.999994594	0.94442346	Soltu.DM.10G024410/Soltu.DM.02G031090/Soltu.DM.09G005320/Soltu.DM.09G026810/Soltu.DM.12G024030/Soltu.DM.02G012570/Soltu.DM.04G022240/Soltu.DM.10G002320/Soltu.DM.06G023200	9
2545	GO:0044706	multi-multicellular organism process	199/12756	0.996603845	0.999994594	0.94442346	Soltu.DM.10G024410/Soltu.DM.02G031090/Soltu.DM.09G005320/Soltu.DM.09G026810/Soltu.DM.12G024030/Soltu.DM.02G012570/Soltu.DM.04G022240/Soltu.DM.10G002320/Soltu.DM.06G023200	9
2546	GO:0006458	'de novo' protein folding	58/12756	0.996665677	0.999994594	0.94442346	Soltu.DM.05G027000	1
2547	GO:0072657	protein localization to membrane	58/12756	0.996665677	0.999994594	0.94442346	Soltu.DM.02G032340	1
2548	GO:0010090	trichome morphogenesis	81/12756	0.9967571	0.999994594	0.94442346	Soltu.DM.08G030100/Soltu.DM.12G010960	2
2549	GO:0042274	ribosomal small subunit biogenesis	81/12756	0.9967571	0.999994594	0.94442346	Soltu.DM.12G024350/Soltu.DM.01G051600	2
2550	GO:0000724	double-strand break repair via homologous recombination	101/12756	0.9968465	0.999994594	0.94442346	Soltu.DM.09G025170/Soltu.DM.05G023970/Soltu.DM.03G002260	3
2551	GO:0048232	male gamete generation	82/12756	0.997029427	0.999994594	0.94442346	Soltu.DM.07G022050/Soltu.DM.12G023840	2
2552	GO:0010564	regulation of cell cycle process	154/12756	0.997051274	0.999994594	0.94442346	Soltu.DM.03G003730/Soltu.DM.10G020590/Soltu.DM.11G016820/Soltu.DM.02G033290/Soltu.DM.12G030150/Soltu.DM.12G023230	6
2553	GO:0043414	macromolecule methylation	187/12756	0.997253169	0.999994594	0.94442346	Soltu.DM.09G024860/Soltu.DM.09G019870/Soltu.DM.04G006870/Soltu.DM.05G000070/Soltu.DM.01G032530/Soltu.DM.10G024770/Soltu.DM.01G024940/Soltu.DM.06G017810	8
2554	GO:0000725	recombinational repair	103/12756	0.997317721	0.999994594	0.94442346	Soltu.DM.09G025170/Soltu.DM.05G023970/Soltu.DM.03G002260	3
2555	GO:0061982	meiosis I cell cycle process	103/12756	0.997317721	0.999994594	0.94442346	Soltu.DM.12G023260/Soltu.DM.09G025170/Soltu.DM.05G023970	3
2556	GO:0061077	chaperone-mediated protein folding	84/12756	0.997508337	0.999994594	0.94442346	Soltu.DM.11G019990/Soltu.DM.05G027000	2
2557	GO:0010212	response to ionizing radiation	61/12756	0.997519357	0.999994594	0.94442346	Soltu.DM.09G025170	1
25	GO:00	meiotic chromosome segregation	61/	0.9	0.9	0.9	Soltu.DM.09G025170	1

58	45132		12756	97519357	99994594	4442346		
2559	GO:0022618	ribonucleoprotein complex assembly	157/12756	0.99760162	0.999994594	0.94442346	Soltu.DM.08G019530/Soltu.DM.12G025260/Soltu.DM.10G028250/Soltu.DM.01G051600/Soltu.DM.10G001400/Soltu.DM.03G021590	6
2560	GO:0071826	ribonucleoprotein complex subunit organization	161/12756	0.998183681	0.999994594	0.94442346	Soltu.DM.08G019530/Soltu.DM.12G025260/Soltu.DM.10G028250/Soltu.DM.01G051600/Soltu.DM.10G001400/Soltu.DM.03G021590	6
2561	GO:0051668	localization within membrane	88/12756	0.998249512	0.999994594	0.94442346	Soltu.DM.10G022360/Soltu.DM.02G032340	2
2562	GO:0000959	mitochondrial RNA metabolic process	66/12756	0.998484971	0.999994594	0.94442346	Soltu.DM.06G002320	1
2563	GO:0002181	cytoplasmic translation	66/12756	0.998484971	0.999994594	0.94442346	Soltu.DM.08G019530	1
2564	GO:0061024	membrane organization	181/12756	0.998572711	0.999994594	0.94442346	Soltu.DM.08G001900/Soltu.DM.04G000190/Soltu.DM.03G017520/Soltu.DM.06G022490/Soltu.DM.08G011890/Soltu.DM.10G022710/Soltu.DM.06G025410	7
2565	GO:0032506	cytokinetic process	67/12756	0.998627281	0.999994594	0.94442346	Soltu.DM.12G023230	1
2566	GO:0016197	endosomal transport	68/12756	0.998756234	0.999994594	0.94442346	Soltu.DM.01G000060	1
2567	GO:0000956	nuclear-transcribed mRNA catabolic process	70/12756	0.998978961	0.999994594	0.94442346	Soltu.DM.05G026810	1
2568	GO:0140013	meiotic nuclear division	135/12756	0.99906747	0.999994594	0.94442346	Soltu.DM.12G023260/Soltu.DM.09G025170/Soltu.DM.05G023970/Soltu.DM.12G023840	4
2569	GO:0080135	regulation of cellular response to stress	118/12756	0.999218569	0.999994594	0.94442346	Soltu.DM.08G022900/Soltu.DM.07G019630/Soltu.DM.03G021360	3
2570	GO:0070925	organelle assembly	193/12756	0.999374279	0.999994594	0.94442346	Soltu.DM.01G039130/Soltu.DM.09G019870/Soltu.DM.11G022310/Soltu.DM.01G020880/Soltu.DM.07G024370/Soltu.DM.01G051600/Soltu.DM.03G021590	7
2571	GO:0006364	rRNA processing	198/12756	0.999559122	0.999994594	0.94442346	Soltu.DM.12G024350/Soltu.DM.10G013520/Soltu.DM.12G016740/Soltu.DM.11G024760/Soltu.DM.01G032530/Soltu.DM.01G051600/Soltu.DM.09G002100	7
2572	GO:0006605	protein targeting	182/12756	0.999595817	0.999994594	0.94442346	Soltu.DM.04G037150/Soltu.DM.08G023320/Soltu.DM.10G004300/Soltu.DM.01G039100/Soltu.DM.02G032340/Soltu.DM.12G021450	6
2573	GO:0007276	gamete generation	106/12756	0.999649884	0.999994594	0.94442346	Soltu.DM.07G022050/Soltu.DM.12G023840	2
2574	GO:0006400	tRNA modification	169/12756	0.999720481	0.999994594	0.94442346	Soltu.DM.06G028580/Soltu.DM.02G028320/Soltu.DM.01G046920/Soltu.DM.12G030020/Soltu.DM.09G019340	5
2575	GO:0042273	ribosomal large subunit biogenesis	99/12756	0.99994181	0.999994594	0.94442346	Soltu.DM.12G024350	1

2576	GO:0140694	non-membrane-bounded organelle assembly	129/12756	0.999956914	0.999994594	0.94442346	Soltu.DM.01G051600/Soltu.DM.03G021590	2
2577	GO:0006310	DNA recombination	177/12756	0.999968879	0.999994594	0.94442346	Soltu.DM.12G023260/Soltu.DM.09G025170/Soltu.DM.05G023970/Soltu.DM.03G002260	4
2578	GO:0009691	cytokinin biosynthetic process	123/12756	0.999994594	0.999994594	0.94442346	Soltu.DM.09G019340	1

gene_name	B-1	B-2	B-3	R-1	R-2	R-3	WT-1	WT-2	WT-3
Soltu.DM.11G016910 (StABI5)	28.91831	38.15633	36.86945	27.98239	29.93919	22.28341	16.39488	18.47707	12.57584
Soltu.DM.09G003280(StBZIP)	25.16174	20.65486	28.81482	1.554847	0.607411	0.400219	11.01687	10.66526	10.39916
Soltu.DM.08G023690(StSnRK2.2)	41.66855	45.11056	38.08873	16.9054	18.78535	17.7728	27.02692	22.04523	27.80897
Soltu.DM.05G027280(StSnRK2.6)	0	0.282686	1.506023	2.648978	0.715622	0.096159	1.888815	1.20208	1.915941
Soltu.DM.04G033590(StABI3)	29.18924	47.40131	49.275	17.42572	20.3852	10.80749	26.36792	22.9478	25.28316
Soltu.DM.04G027660(StCCJ9)	5.10529	10.81565	10.25877	111.5086	116.9468	91.0164	34.98297	35.9588	30.96926
Soltu.DM.04G027650(StDWF)	150.5452	159.8994	139.2419	5117.504	4836.666	3827.298	340.2366	82.48277	288.5564
Soltu.DM.02G006820(StBZR1)	25.96371	28.44888	24.56399	45.84542	43.12583	36.05467	39.83897	42.91159	32.77914
Soltu.DM.01G044300(StPOD)	12.70915	11.82047	11.40855	346.3388	237.1165	138.7865	32.19373	15.54571	12.85476

Table S7. Primer sequence

Gene	Purpose	Forward/Reverse
StBIN2	Cloning of RNAi	GAGAGAACACGGGGGACCCGGGAGTTGCGATAAAGAAGGT<br>GAGAAAAACTAGACCTGCAGGCCCGGGTTGGTTCATATTGCT
StBIN2	Cloning of OE	GGATCCATGGCTGAACGTATTGTGGG<br>GGATCCTTATGCCAAGGGTGTGCTT
StBIN2	luciferase complementary	GGAGCTCGGTACCCTCGAGGGATGGATCGGACGGCAGTGA<br>TTAAGCAGAGATTACCTATCTAGATTACATTGCATAGACAATCTCT
StSnRK2.2	luciferase complementary	TTGGAGAGAACACGGGGGACGAGCTCATGGAGGAAAAGTATGA<br>CGGGACGCGTACGAGATCTGGTCGACGACATAAACAGCAAAGT
StBIN2	qPCR	CTGCTCACGGTTTTATCGGG<br>TCCTCTCTCGTTGGTGTTCC
StPOD	qPCR	CTGCTCGTGACTCTGTTGCT<br>CCTACTGTA TGGGACCCGAC
StCCJ9	qPCR	GCTGCTCGTGACTCTGTTTT<br>GAAGGTGGTGGAAGATTGGC
StSnRK2.2	qPCR	TTATGGAGTACGCAGCAGGT<br>CCCACAGTCGACTTTGGTTG
StBZR1	qPCR	CCCTTTCTATTTCCAGGTTTG<br>TTCAAGATTATTCAATGGGCA
StABI3	qPCR	CCATGGTTCCATTGGCTGAT<br>TGCCAACATCACTTTGCTTC
StABI5	qPCR	GAACTGGAGAACAAGGTTTCAC<br>GACATAACGAACACTACGCATC
